# Synthesis of γ-Hydroxy-α-amino Acid Derivatives by Enzymatic Tandem Aldol Addition–Transamination Reactions

**DOI:** 10.1021/acscatal.1c00210

**Published:** 2021-04-02

**Authors:** Carlos J. Moreno, Karel Hernández, Simon J. Charnok, Samantha Gittings, Michael Bolte, Jesús Joglar, Jordi Bujons, Teodor Parella, Pere Clapés

**Affiliations:** † Institute for Advanced Chemistry of Catalonia, Department of Biological Chemistry, IQAC-CSIC, Jordi Girona 18-24, Barcelona 08034, Spain; ‡ Prozomix Ltd. West End Industrial Estate, Haltwhistle, Northumberland NE49 9HA, U.K.; § Institut für Anorganische Chemie, J.-W.-Goethe-Universität, Frankfurt/Main, Germany; ∥ Servei de Ressonància Magnètica Nuclear, Universitat Autònoma de Barcelona, Bellaterra, Spain

**Keywords:** biocatalysis, 2-oxoacid aldolase, transaminases, aldol addition, reductive amination, γ-hydroxy-α-amino acids

## Abstract

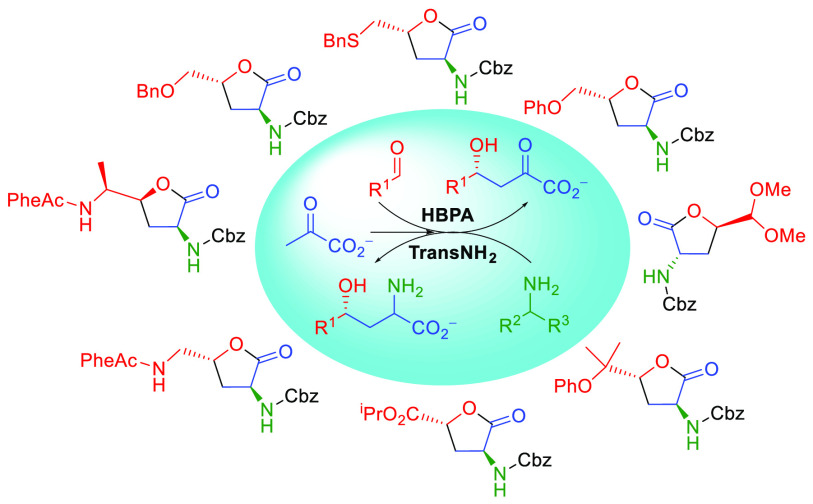

Three enzymatic routes toward γ-hydroxy-α-amino acids by tandem aldol addition–transamination one-pot two-step reactions are reported. The approaches feature an enantioselective aldol addition of pyruvate to various nonaromatic aldehydes catalyzed by *trans*-*o*-hydroxybenzylidene pyruvate hydratase-aldolase (HBPA) from *Pseudomonas putida*. This affords chiral 4-hydroxy-2-oxo acids, which were subsequently enantioselectively aminated using *S*-selective transaminases. Three transamination processes were investigated involving different amine donors and transaminases: (i) l-Ala as an amine donor with pyruvate recycling, (ii) a benzylamine donor using benzaldehyde lyase from *Pseudomonas fluorescens* Biovar I (BAL) to transform the benzaldehyde formed into benzoin, minimizing equilibrium limitations, and (iii) l-Glu as an amine donor with a double cascade comprising branched-chain α-amino acid aminotransferase (BCAT) and aspartate amino transferase (AspAT), both from *E. coli*, using l-Asp as a substrate to regenerate l-Glu. The γ-hydroxy-α-amino acids thus obtained were transformed into chiral α-amino-γ-butyrolactones, structural motifs found in many biologically active compounds and valuable intermediates for the synthesis of pharmaceutical agents.

## Introduction

**Figure 1:**
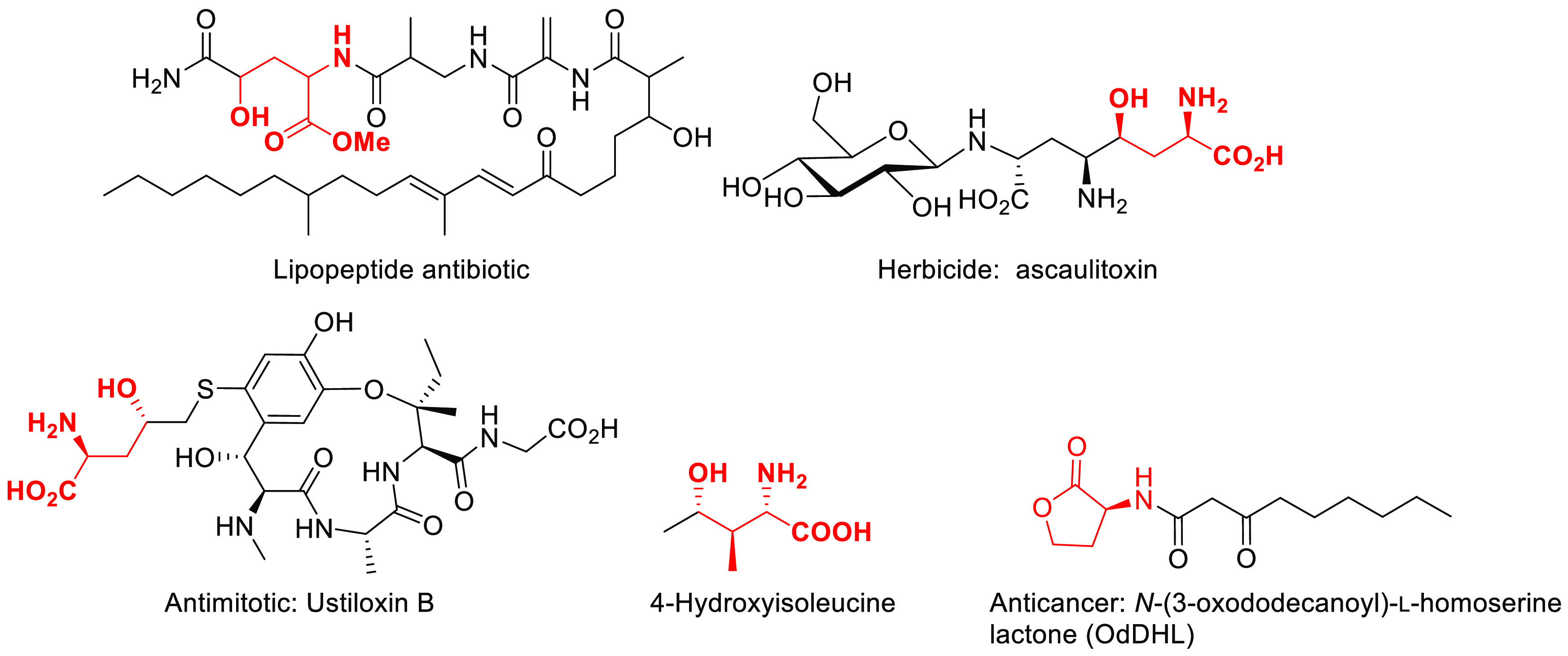
Examples of bioactive compounds bearing a γ-hydroxy-α-amino acid moiety or α-amino-γ-butyrolactone.

γ-Hydroxy-α-amino acids represent compounds with relevant biological and pharmacological importance.^1^ Examples include antidiabetics such as (2*S*,3*R*,4*S*)-4-hydroxyisoleucine, 4-hydroxy-l-norvaline, and 4-hydroxypipecolic acid and nutritional supplements in the food industry, e.g. *trans*-4-hydroxy-l-proline.^1a,2^ Moreover, they constitute structural motifs of more complex naturally occurring and synthetic molecules, namely antibiotics,^3^ antimitotics,^4^ and (bio)herbicides,^5^ as well as chiral building blocks for the production of active ingredients, e.g. α-amino-γ-butyrolactones, 4,5-dihydroxynorvaline, and 4-hydroxypyroglutamic acid and derivatives (Figure 1).^2b,2d,6^

**Figure 2:**
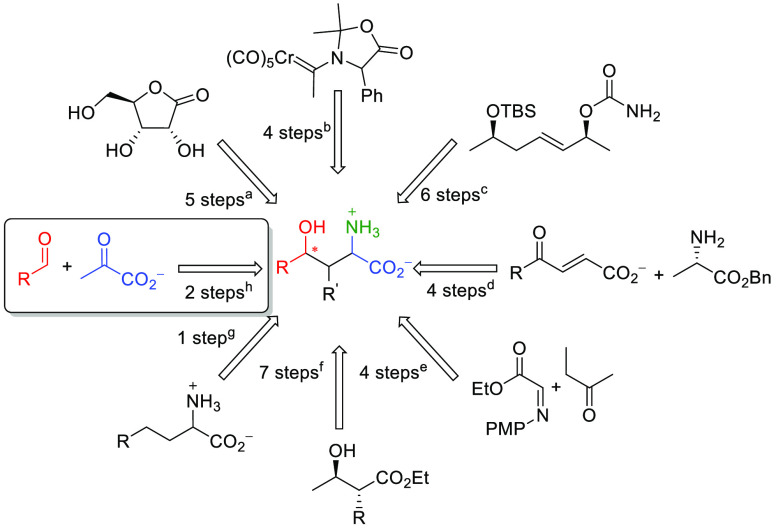
Examples of strategies for the synthesis γ-hydroxy-α-amino acids: (a) synthesis from d-ribonolactone as a chiral precursor and stereocontrolled transformation;^7d^ (b) aldol reaction with oxazolidonyl chromium carbene complex and photocyclization;^7c^ (c) cyanate to isocyanate rearrangement and enzymatic kinetic resolution;^1a^ (d) aza-Michael additions controlled by a crystallization-induced asymmetric transformation;^7f^ (e) Mannich condensation and catalytic epimerization;^7b^ (f) palladium(II)-catalyzed aza Claisen rearrangements;^7a^ (g) direct β or γ-hydroxylation of amino acids via α-ketoglutarate-dependent dioxygenases;^2c,8a^ (h) enzymatic synthesis via carboligase-transaminase reactions.^2e,8b,8e,9^

Therefore, substantial research efforts have been devoted to their synthesis using multistep catalytic or stoichiometric chemical approaches (Figure 2).^1a,2g,7^ Biocatalytic access to these compounds is regarded as a powerful strategy because of their simplicity and stereoselectivity starting from simple achiral materials (Figure 2).^2c,2e,8^

In this sense, the sequential combination of carboligases and transaminases in one-pot one-step or one-pot two-step reactions with or without substrate recycling offers broad synthetic possibilities. Using this route, chiral l- and d-homoserine, d-*anti*-dihydroxynorvaline, 4-hydroxyisoleucine, norpseudoephedrine, and norephedrine were prepared.^2e,8b,8e,9^ However, the number of examples is scarce and, although they represent a remarkable achievement, they are often limited by the poor stereoselectivity profile of pyruvate aldolases as catalysts to generate the corresponding 4-hydroxy-2-oxo acid precursors or the lack of transaminase selectivity toward the aldol adduct.

Herein, we report three strategies for the biocatalytic diastereoselective synthesis of γ-hydroxy-α-amino acids by combining an enantioselective pyruvate-aldolase and *S*-selective transaminases,^8b^ starting from pyruvate and diverse aldehyde substrates.

## Results and Discussion

### Stereoselective Aldol Addition of Pyruvate to Aldehydes Catalyzed by *trans*-*o*-Hydroxybenzylidene Pyruvate Hydratase-Aldolase (HBPA)

**Scheme 1:**
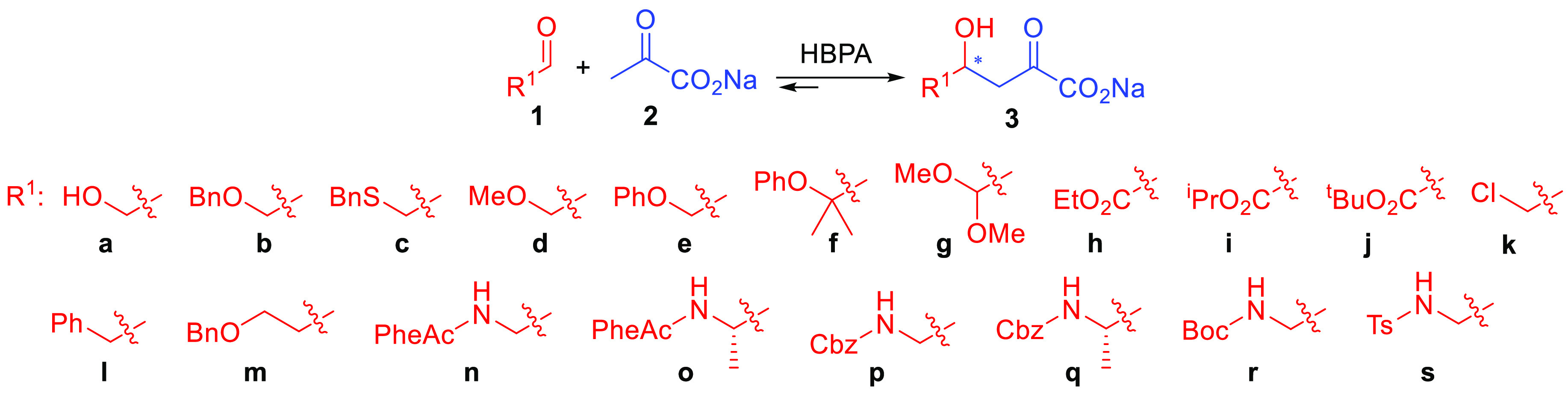
Panel of Aldehyde Substrates **1** Assayed in the Aldol Addition of Pyruvate **2** Catalyzed by Wild-Type HBPA and Its H205A Variant

The *trans*-*o*-hydroxybenzylidene pyruvate hydratase-aldolase (HBPA, EC 4.1.2.45) from *Pseudomonas putida* was discovered in the metabolic degradative pathway of naphthalene and naphthalenesulfonates.^10^*In vivo*, HBPA catalyzes the reversible aldol condensation of pyruvate to salicylaldehyde in two steps: an aldol addition and a subsequent dehydration, both steps being catalyzed by the enzyme. Interestingly, it has been reported that HBPA catalyzes the aldol addition of fluoropyruvate to aromatic aldehydes with high stereoselectivity, in which the dehydration products were not detected, likely because the fluorine atom precludes the dehydration step by the enzyme.^11^ We envisioned that HBPA could catalyze aldol additions of pyruvate to nonaromatic electrophiles, in which the dehydration activity could be largely minimized or even suppressed, rendering aldol adducts with high enantioselectivity. We assayed various nonaromatic electrophiles (**1a**–**s**; Scheme 1) as substrates of HBPA in the aldol addition of pyruvate.

**Scheme 2:**
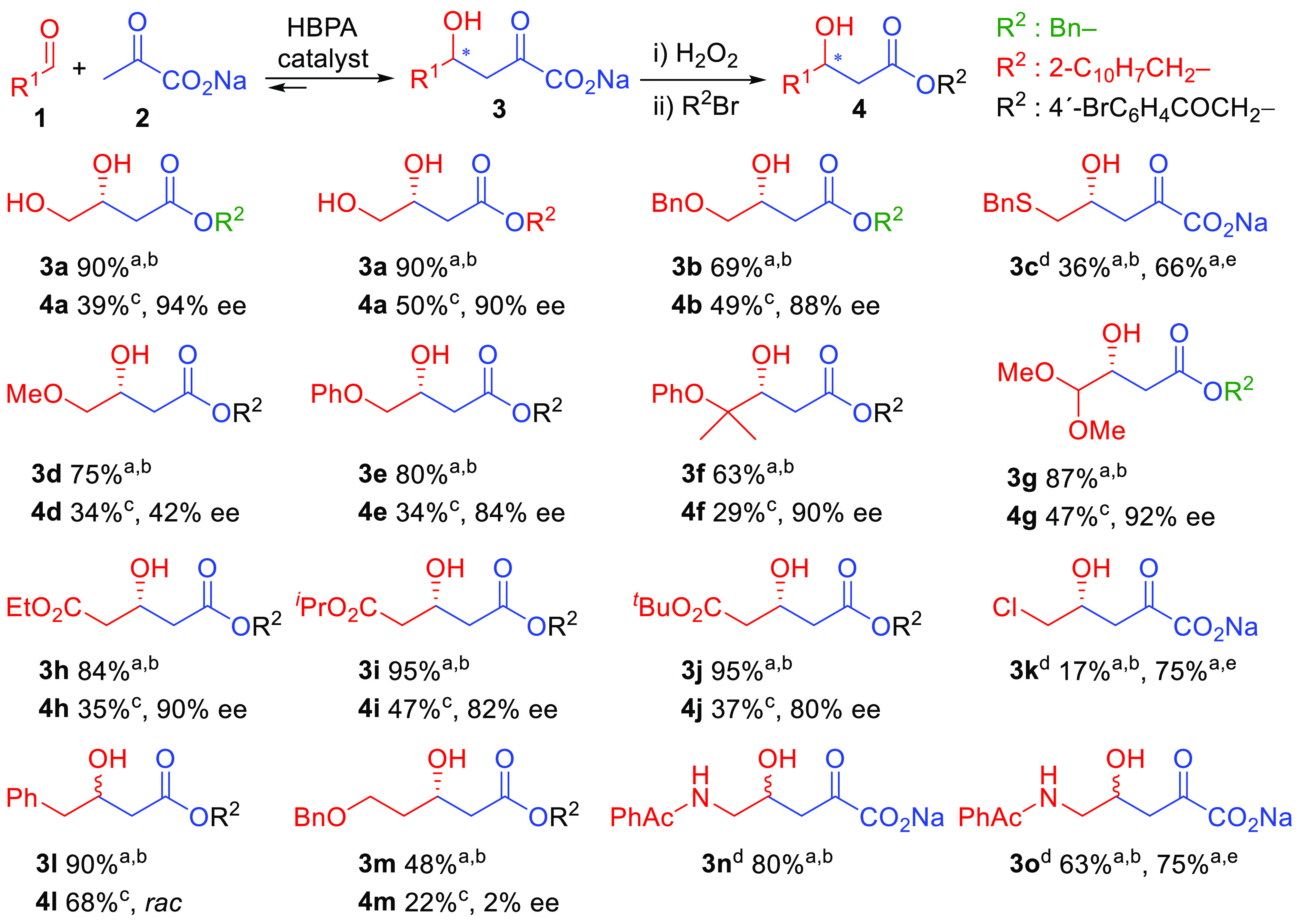
HBPA Wild-Type and H205A Variant Catalyzed Aldol Addition of Pyruvate to Aldehydes **1** and Transformation of Aldol Adducts **3** into 3-Hydroxy Ester Derivatives **4**, for Enantiomeric Ratio Measurement Conversions measured by HPLC. HBPA wild-type. Isolated yield. ee not determined; the material was submitted directly to the enzymatic transamination reaction and the stereochemistry inferred from the corresponding **14c** (for **3c**), **15k** (for **3k**), **14n** (for **3n**), and **14o** (for **3o**) derivatives; see Schemes 7 and 8. HBPA H205A variant. The ee values were determined by HPLC on a chiral stationary phase.

Good to excellent conversions to 4-hydroxy-2-oxo acids **3** (70–95%) were achieved for most of the electrophiles, indicating an excellent structural substrate tolerance of HBPA (Scheme 2). A single residue mutation, the HBPA H205A variant, greatly improved the catalyst efficiency toward **1c**,**k**,**o**, yielding **3c**,**k**,**o**, respectively, in 66–75% conversion.

**Figure 3:**
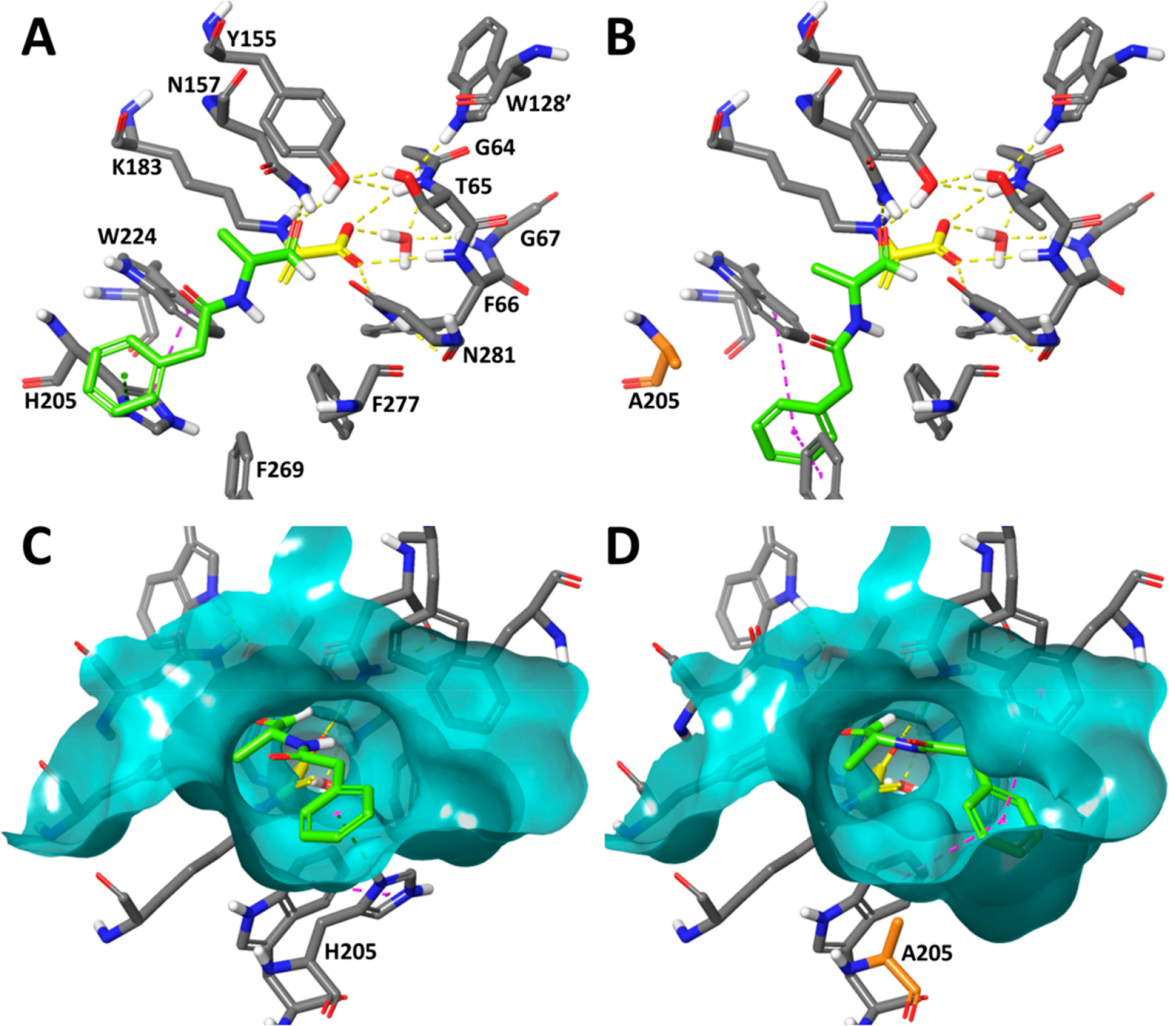
Models of the prereactive complexes of wild-type HBPA (A, C) and HBPA H205A (B, D) with the covalently bound pyruvate enamine (yellow C atoms) and aldehyde **1o** (green C atoms). The mutated residue is highlighted in orange. Interactions are shown with dashed lines: H bonds in yellow, π-stacking in magenta, and π-cation in dark green. A comparison of the surface of the active sites of both proteins (C, D) (transparent cyan) reveals that the H205A mutation generates an expanded cavity near residue A205, which can be occupied by the phenyl moiety of **1o**. These models were obtained by QM/MM optimization of the structure of the complexes at the DFT (B3LYP/6-31G**) level of theory, as detailed in the Supporting Information.

**Figure 4:**
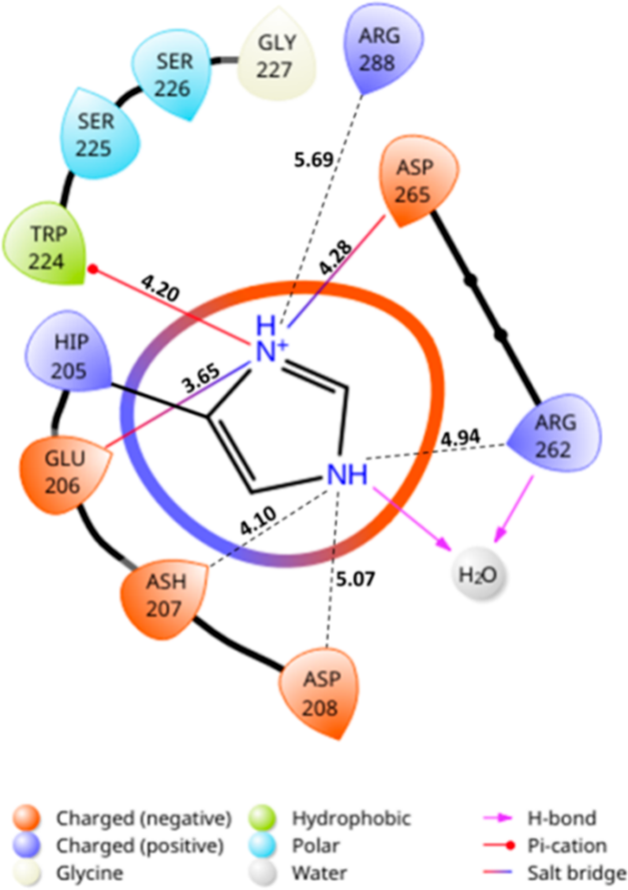
Scheme showing the interactions established by the imidazole group of residue His205 of HBPA. Distances (dashed lines) to nearby residues are indicated.

Molecular models of the complex of pyruvate-enamine-bound HBPA with aldehydes **1c**,**o** suggest that the H205A mutation generates a new cavity in the HBPA active site, which reduces the steric hindrance. This cavity can be occupied by the phenyl moiety of the incoming aldehyde (Figure 3 and Figure S93), thus facilitating the interactions with residues Trp224 and Phe269. Moreover, an analysis of the HBPA structure shows that the carboxylic groups of Glu206, Asp207, Asp208, and Asp265 are within 5 Å of the δ and ε-N atoms of the imidazole group of His205, stabilizing its protonated state (Figure 4). This positive charge is also stabilized by a π-cation interaction with the aromatic moiety of Trp224. Therefore, removal of this protonated imidazole, which is ∼8 Å from the ε-amino group of the essential Lys183, by the H205A mutation modifies the electrostatic environment of the active site, which could also contribute to the enhanced activity of this mutant toward the selected substrates.

Enantiomeric ratios were determined by HPLC on a chiral stationary phase after transforming the aldol adducts **3** into 3-hydroxy ester derivatives **4** (Scheme 2 and Figures S74–S85). The corresponding authentic racemic samples were produced by employing 2-oxo-3-deoxy-l-rhamnonate aldolase (YfaU), which yielded aldol adducts **3** as racemic mixtures with the selected electrophiles (see the Supporting Information). Excellent levels of enantioselectivity were achieved for **3a**,**f**–**h** (96–90% ee), while they were good to moderate for **3b**,**e**,**i**,**j** (88–80% ee). This is significant considering the low stereoselective profile of wild-type pyruvate aldolases toward low-molecular-weight electrophiles.^12^ Low enantioselectivy was attained with the methoxy derivative **3d** (42% ee), whereas racemates were obtained with phenylacetaldehyde (**3l**) and 3-(benzyloxy)propanal (**3m**). Cbz-, Boc-, and Ts-protected aminoethanal compounds **1p**,**r**,**s**, respectively, and (*S*)-Cbz-2-aminopropanal (**1q**) were not converted. Thus, PhAc was the amino protecting group of choice for aminoaldehydes in the planned HBPA catalysis. Interestingly, PheAc has the advantage that it can be removed by penicillin G acylase, a mild and orthogonal protection compatible with most common amino masking groups.^13^

**Figure 5:**
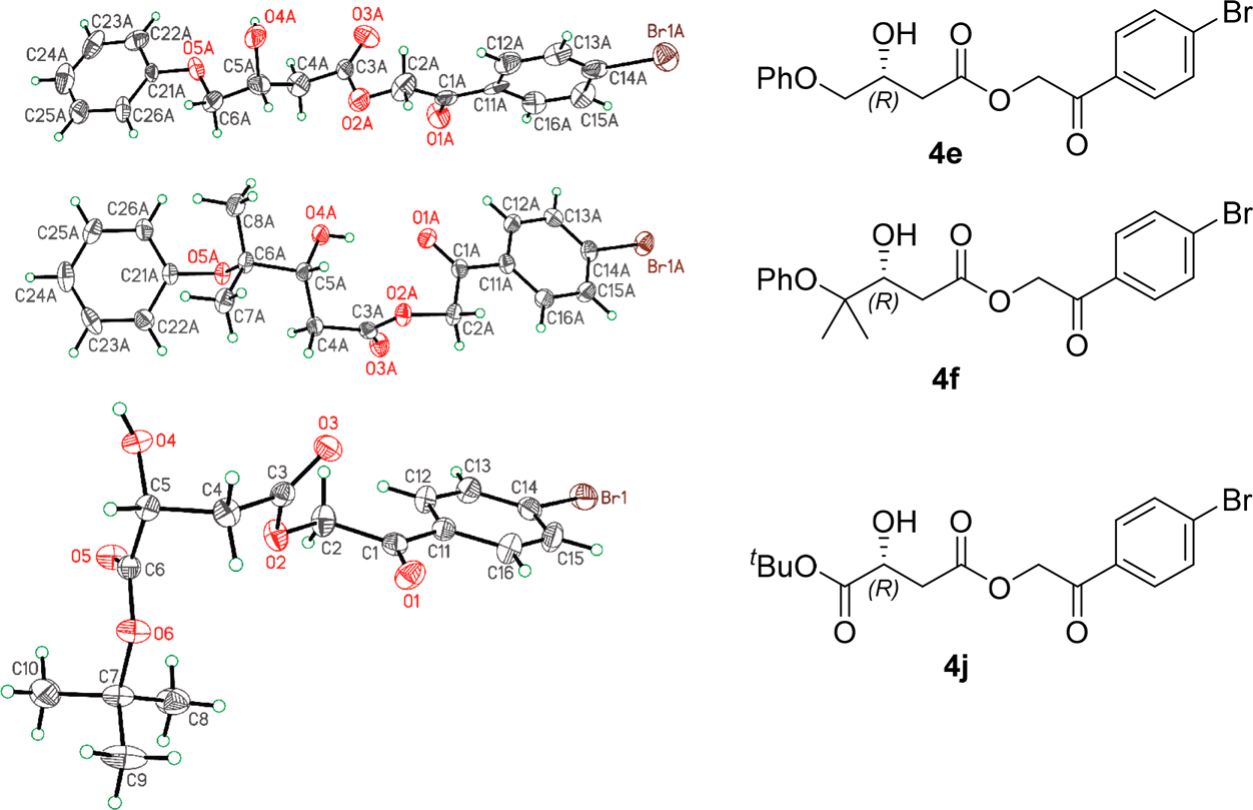
X-ray structures of (*R*)-**4e**, (*R*)-**4f**, and (*R*)-**4j** as ORTEP-type plots displaying one molecule with 50% probability ellipsoids. The data can be obtained free of charge from The Cambridge Crystallographic Data Centre via www.ccdc.cam.ac.uk/data_request/cif.

Single-crystal X-ray diffraction studies of compounds **4e**,**f**,**j** indicate that HBPA renders aldol adducts having an *R* configuration as the major products (Figure 5). This is consistent with the reported stereochemical outcome of the reactions between fluoropyruvate and (hetero)aromatic aldehydes.^11^ Therefore, an *R* configuration may safely be assumed for the major enantiomers of **3a**–**k** (Scheme 2).

Molecular models of the complexes of HBPA were obtained with (i) the covalently bound pyruvate enamine and the electrophile molecules (Figures S88–S93) and (ii) the imines derived from the aldol adducts (Figures S94–S99). These models suggest that there is no steric restriction to the *re*- or *si*-face approach of the electrophile to the enamine. Thus, it is not clear why there is a preference for the *re*-face approach, which would generate the *R*-aldol adducts as major products of the reaction. A more in depth theoretical study would be required to determine the source of this preference; however, this is beyond the scope of this paper.

### Synthesis of γ-Hydroxy-α-amino Acid Derivatives Using a Biocatalytic One-Pot Cyclic Cascade Approach with l-Ala as an Amine Donor

**Scheme 3:**
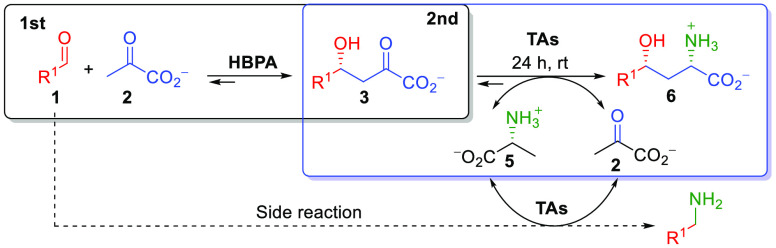
One-Pot Two-Step Screening Reaction for the Synthesis of γ-Hydroxy-α-amino Acid Derivatives **6**, Using l-Ala (**5**) as an Amine Donor for the Enzymatic Transamination Reaction The dotted line indicates an enzymatic transamination of the aldehyde remaining in the system.

We began to screen a transaminase panel (T001–T050) provided by Prozomix Ltd. toward the selected aldol adduct examples **3a**,**b**,**e**,**g**,**h**, produced with good conversions and enantioselectivities by HBPA catalysis (Figures S8–S12). We used l-Ala (**5**) as an amino donor in a one-pot two-step reaction sequence (Scheme 3). Thus, for the screening, the enzymatic aldol addition was run first and, once the formation of **3** reached a maximum, l-Ala (**5**) (500 mM, ≥ 10 equiv with respect to **3**) and the transaminase (T###) were added, allowing the reaction to proceed for 24 h. Since unpurified aldol adducts **3** were supplied for the screening reactions, HPLC analysis was the method of choice to detect false positives caused by transamination of the remaining unreacted aldehyde **1**. Compounds **6b**,**e**, bearing aromatic moieties, were directly detected by HPLC. The percentages of **6a**,**g**,**h** formed were estimated by measuring the consumption of **3a**,**g**,**h** by HPLC, after precolumn derivatization of the carbonyl group via oxime formation (see the Supporting Information).

**Scheme 4:**
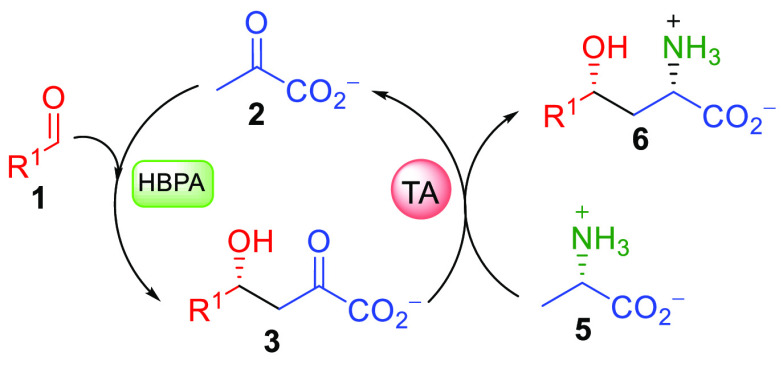
One-Pot Biocatalytic Cascade Synthesis of γ-Hydroxy-α-amino Acid Derivatives **6** Starting from Aldehydes **1** and an Amine Donor **5** with Pyruvate (**2**) Recycling

Roughly, 4 out of the 50 different transaminases were selected as promising candidates for the transamination reaction (Figures S8–S12). For scale-up experiments, we capitalize on a strategy developed by our group consisting of a one-pot reaction recycling of pyruvate **2**, formed in the transamination reaction, into the aldol reaction (Scheme 4).^8b^ This approach effectively shifts the equilibrium of the transamination to the product, since the pyruvate is continuously removed by the aldol reaction.

**Figure 6:**
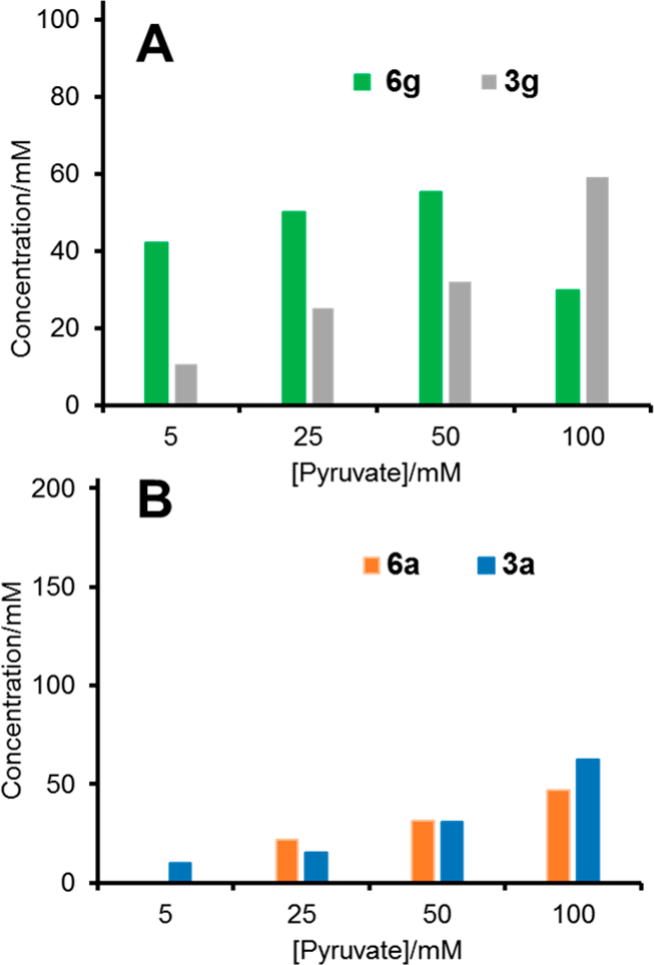
One-pot biocatalytic synthesis of **6g** (A) and **6a** (B), starting from aldehydes **1g** (100 mM), **1a** (200 mM), and **5** (100 mM) in both experiments: concentration of the components after 24 h of reaction as a function of the initial concentration of pyruvate using HBPA/T039 catalysts.

To successfully perform this strategy, the transamination of the aldehydes **1** must be largely avoided or minimized. To evaluate the degree of conversion for each aldehyde and establish suitable reaction conditions for the one-pot cascade process, we ran control experiments incubating aldehydes **1a**,**b**,**e**,**g**,**h** (100 mM) with selected transaminases (Figures S13–S18). Having established the suitable conditions, we started testing a range of starting pyruvate (**2**) concentrations between 5 and 100 mM, against 100 mM of l-Ala. Using **1g** (100 mM) as the electrophile and HBPA/T039 catalysts, 42 mM of **6g** (42% yield) was formed at 5 mM pyruvate concentration, indicating pyruvate recycling (Figure 6A and Figure S21). At 50 mM of pyruvate, **6g** reached a maximum (55 mM, 55% yield), with no effective pyruvate recycling. Further increasing the initial pyruvate concentration (i.e., up to 100 mM) caused an increase in **3g** production, but **6g** decreased, likely due to an equilibrium limitation of the transamination. Using **1a** (200 mM) and HBPA/T039, the best initial pyruvate concentration was 100 mM (Figure 6B and Figure S19). Under these conditions, 47 mM of **6a** (47% yield, with respect to the limiting substrate **5**) was formed, but no pyruvate recycling occurred. Aldol adduct **3a** was most probably in an equilibrium between the cyclic hemiketal and the acyclic form. It is likely that the hemiketal was not a substrate for the transaminase and its activity was limited by the actual concentration of the acyclic adduct. Disappointingly, this strategy failed to produce the corresponding γ-hydroxy-α-amino acid derivatives **6** for the rest of the aldehydes with the selected transaminases and aldol adducts **3** were the only species detected (see Figure S20 for an example).

One problem that jeopardizes the pyruvate recycling strategy was that the aldolase and transaminase were not sufficiently active toward the aldehyde and aldol adduct, respectively, in comparison with the system reported by us for the synthesis of homoserine.^8b^ This makes a rapid conversion difficult for both the initial pyruvate and the aldol adduct formed, compromising the efficiency of the recycling process. Therefore, the one-pot two-step reaction sequence appears to be the most convenient route in dealing with aldolases and/or transaminases with kinetic and thermodynamic limitations. In this case, however, an effective method to overcome the equilibrium limitations of the transaminase must be implemented.

### Synthesis of γ-Hydroxy-α-amino Acid Derivatives Using a Biocatalytic One-Pot Two-Step Approach Using Benzylamine as an Amine Donor

**Scheme 5:**
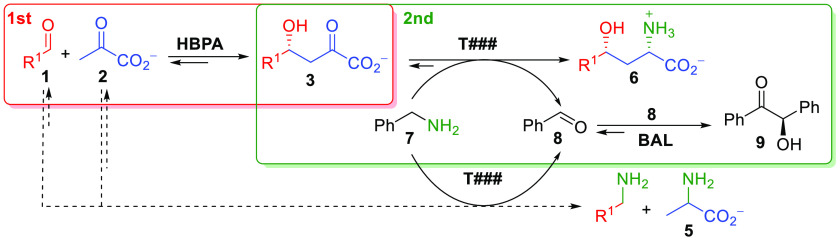
One-Pot Two-Step Stereoselective Synthesis of γ-Hydroxy-α-amino Acids **6**, Using Benzylamine (**7**) as an Amine Donor for the Enzymatic Transamination Reaction and BAL to Transform the Benzaldehyde Formed (**8**) into Benzoin (**9**) Dotted lines indicate a transamination reaction of the aldehydes **1** and pyruvate **2**, favoring the retroaldolysis of **3** catalyzed by the HBPA present in the system.

Another strategy was devised for the synthesis of the selected target γ-hydroxy-α-amino acid derivatives using benzylamine (**7**) as an alternative amine donor (Scheme 5). The effective shift of the equilibrium of the transamination was accomplished by converting the benzaldehyde (**8**) formed into benzoin (**9**) by benzaldehyde lyase from *Pseudomonas fluorescens* Biovar I (BAL) (Scheme 5). BAL is highly selective toward benzaldehyde, ensuring an almost quantitative conversion into the mostly insoluble benzoin, which benefits the reaction equilibrium.^14^ Benzylamine has been reported to be a suitable amine donor, and different strategies to eliminate the benzaldehyde were proposed: e.g. conversion into benzoic acid under 1 atm of oxygen,^15^ extraction with hexane in an aqueous–organic two-phase system,^16^ and reduction to benzyl alcohol during a cascade synthesis of ω-amino fatty acids and α,ω-diamines from ω-hydroxy fatty acids and α,ω-diols, respectively.^17^

For this strategy, an extended panel of 194 transaminases from Prozomix Ltd. was screened in a one-pot two-step sequence (Figure S22 and Table S7) using the crude reaction products of adducts **3a**,**b**,**e**,**g**,**h** as starting materials.

Next, we rescreened 27 positive hits from the first screening in the two-step sequence, removing the HBPA before starting the transamination reaction aldolase to avoid retroaldolysis (see the Supporting Information). In this case, the aldol adducts **3**, products **6**, benzaldehyde (**8**), and benzoin (**9**) were analyzed and quantified by HPLC. Only transaminase T039 was able to convert the aldol adducts **3a**,**b**,**e**,**g** into the corresponding products **6** (Figure S24). The reaction with aldehyde **1h** resulted in a false positive, because no product could be detected in scale-up experiments.

**Figure 7:**
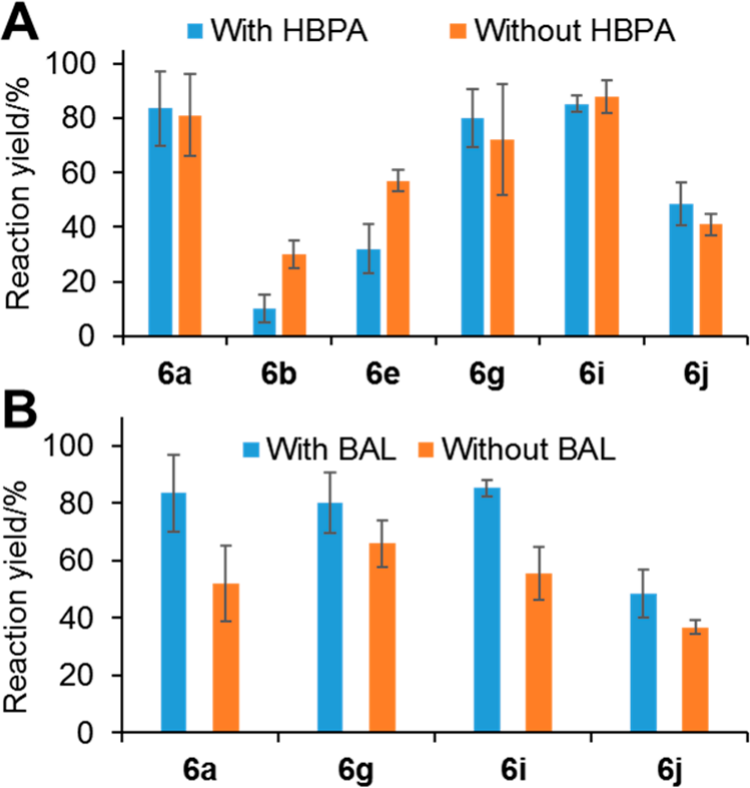
One-pot two-step stereoselective synthesis of γ-hydroxy-α-amino acids (**6a**,**b**,**e**,**g**,**i**,**j**), using the HBPA/T039 system and benzylamine (**7**) as amine donor: influence of the presence of HBPA (A) and BAL (B) on the reaction yield of products **6**. Error bars are the values of the estimated standard error of the mean of two independent experiments under the same reaction conditions.

The same two-step reaction sequence was then repeated with and without removing HBPA, using T039 and including aldehydes **1i**,**j** (Figure 7A). The HBPA did not affect the yield of **6a**,**g**,**i**,**j**, indicating that T039 was rather selective toward the corresponding aldol adducts and that no retroaldolysis was taking place. On the other hand, elimination of HBPA benefited **6b**,**e** in comparison with those products without aromatic substituents. Next, we investigated the effect of BAL on the yield of **6a**,**g**,**i**,**j** (Figure 7B). The addition of BAL was largely positive for **6a**,**i** and less so for **6g**,**j**. Overall, this depends on each individual case and should be considered for establishing the optimal operational conditions. Moreover, no aldol condensation of pyruvate to benzaldehyde catalyzed by HBPA was detected.

The low conversion of aldol adducts with aromatic substituents, **3b**,**e**, and the need for a highly selective transaminase for the 4-hydroxy-2-oxo acids **3** prompted us to explore another methodology based on branched-chain α-amino acid aminotransferases (BCATs).^18^

### Synthesis of γ-Hydroxy-α-amino acid Derivatives Using a Biocatalytic One-Pot Two-Step Approach with the PLP-Dependent Branched-Chain Amino Acid Aminotransferase (BCAT) from *E. coli*

**Scheme 6:**
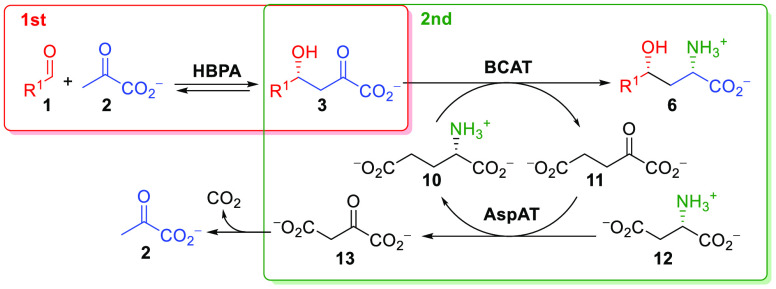
One-Pot Two-Step Stereoselective Synthesis of γ-Hydroxy-α-amino Acids **6** using l-Glu (**10**) as an Amine Donor The 2-oxoglutarate (**11**) formed was transaminated to l-Glu (**10**) by AspAT using l-Asp (**12**) as an amine donor. The oxaloacetate (**13**) decomposes into CO_2_ and pyruvate, shifting the equilibrium of the transamination to γ-hydroxy-α-amino acids **6**.

The branched-chain α-amino acid aminotransferase (BCAT) from *E. coli* was selected to convert **3** into **6**, employing l-Glu (**10**) as an amine donor and delivering 2-oxoglutarate (**11**), which is a strong inhibitor of BCAT (e.g., 10 mM of **11** reduces the activity up to 80%).^19^ Thus, the regeneration of l-Glu was needed, which was accomplished by coupling with aspartate aminotransferase (AspAT) from *E. coli* that employed l-Asp (**12**) as the substrate (Scheme 6). The resulting oxaloacetate **13** spontaneously decomposes into CO_2_ and pyruvate, shifting the transamination equilibrium to the formation of γ-hydroxy-α-amino acids **6**.

**Scheme 7:**
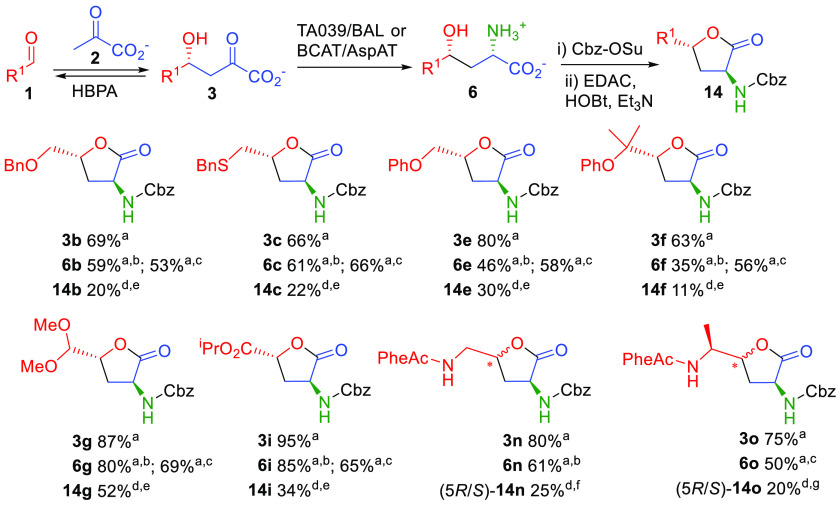
Synthesis of γ-Hydroxy-α-amino Acids **6** by Tandem HBPA/Transaminase Catalyzed Reactions and Conversion to α-Amino-γ-butyrolactone Derivatives **14** Conversions determined by HPLC. Transaminase T039/BAL (see Scheme 5). BCAT/AspAT system (see Scheme 6). Isolated yield. dr >95:5 as measured by NMR; no other diastereomers were detected. dr 50:50 as measured by NMR. dr 60:40 (*S*:*R*) as measured by NMR.

**Scheme 8:**
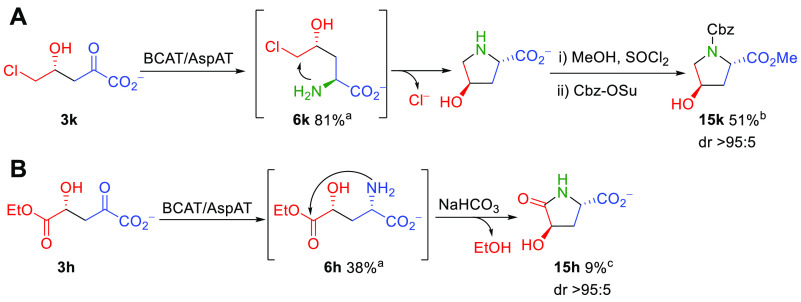
(A) Formation of (2*S*,4*R*)-(−)-*trans*-4-Hydroxyproline Derivative **15k** by Tandem Enzymatic Aldol Reaction/Transamination and Intramolecular Nucleophilic Substitution with the Amine Group and (B) Formation of γ-Hydroxypyroglutamic Acid (**15h**) by Intramolecular Aminolysis of the Ethyl Ester Group under Basic Conditions Percentage of product formed determined by HPLC. Isolated yield from **1k**. Isolated yield from **1h**. The material contained l-Asp and l-Glu as major impurities. The stereochemistry of **15k** was established unequivocally by a comparison with authentic samples (see Figure S26).

The reaction system worked successfully with the aldol adducts **3**, with better performances for some examples in comparison to those with the benzylamine/T039 system (Scheme 7). For instance, this reaction system converted the aldol adduct **3k**, which was not a substrate for T039 (Scheme 8). On the other hand, benzylamine/T039 afforded product **6n**, whereas its precursor **3n** was not converted by the BCAT/AspAT system. Therefore, both methodologies are somehow complementary. Despite the fact that pyruvate is released because of the decarboxylation of oxaloacetate **13**, attempts to run the reaction with one-pot pyruvate recycling failed to provide the corresponding γ-hydroxy-α-amino acids **6** (Figure S25).

Finally, for the laboratory-scale preparation of γ-hydroxy-α-amino acids **6** we chose a system with better performance for conversion, isolation, and product purification. Products **6** were converted into the corresponding Cbz-*N*^α^-γ-butyrolactone derivatives **14** (Scheme 7). α-Amino-γ-butyrolactones are structural motifs found in biologically active compounds in addition to being valuable chiral intermediates for the synthesis of pharmaceutical agents.^6a,20^ The optimal lactonization conditions were established, and particular attention was paid to avoid eroding the enantiopurity of **14** (Table S7). The *R* configuration for aldol adducts **3b**,**c**,**e**–**g**,**i** (Scheme 2) and the *S* configuration generated by the transaminases^8b,19c^ afforded the expected 2*S*,4*R*-configured hydroxy amino acid derivatives **6**, which were confirmed by a NMR diastereochemical analysis of products **14**. Exceptions were the products **6n**,**o**. In this case, the aldol addition reaction was not stereoselective, yielding a mixture of diastereoisomers (5*R*/5*S*)-**14n** and (5*R*/5*S*)-**14o**, respectively.

Interestingly, the enzymatic transamination reaction of aldol adducts **3k**,**h** led to the formation of (2*S*,4*R*)-(−)-*trans*-4-hydroxyproline (**15k**) (Scheme 8 A) and γ-hydroxypyroglutamic acid (**15h**) (Scheme 8 B), respectively. The first product could be formed by an intramolecular nucleophilic substitution of the terminal Cl at C5 by the amine group after the transamination reaction.

The γ-hydroxypyroglutamic acid **15h** was probably formed by intramolecular aminolysis of the ethyl ester group during the attempts to protect the amino group by Cbz. The basic conditions necessary to conduct the reaction with CbzOSu likely favored the reaction (Scheme 8 B). On the other hand, the corresponding isopropyl ester derivative of Cbz-*N*^α^-γ-butyrolactone **14i** could be isolated, most likely due to the steric hindrance imposed by the isopropyl group, which precluded an intramolecular aminolysis.

**Scheme 9:**
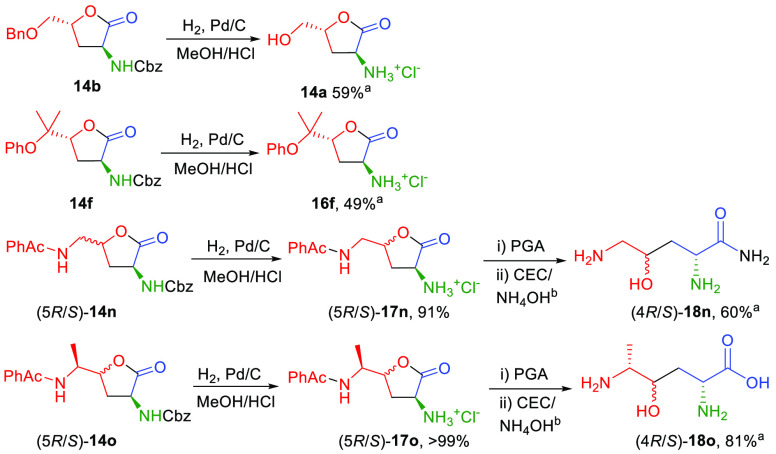
Protection Group Removal of Selected α-Amino-γ-butyrolactone Derivatives **14** Isolated yield. Cation exchange chromatography (CEC) eluted with an aqueous solution of NH_4_OH.

Deprotection of the Cbz group of butyrolactones **14** was accomplished by catalytic hydrogenolysis with H_2_ in the presence of Pd/C, while PheAc was removed by enzymatic hydrolysis mediated by penicillin G acylase (PGA) (Scheme 9). Hydrogenolysis of (5*R*/5*S*)-**14n** and (5*R*/5*S*)-**14o** furnished the lactones (5*R*/5*S*)-**17n** and (5*R*/5*S*)-**17o**, which upon treatment with PGA and purification by cation exchange chromatography, with NH_4_OH as eluent, led to the corresponding amide derivatives (4*R*/4*S*)-**18n** and carboxylates (4*R*/4*S*)-**18o**.

## Conclusions

A tandem enantioselective aldol addition–transamination approach was established for the production of chiral γ-hydroxy-α-amino acids. The *trans*-*o*-hydroxybenzylidene pyruvate hydratase-aldolase afforded chiral 4-hydroxy-2-oxo acids with a remarkable efficiency, broad substrate tolerance, and unparalleled stereoselectivity, far beyond those of the pyruvate aldolases hitherto reported. Thus, the HBPA/benzylamine/T039 and HBPA/Glu/BCAT/AspAT systems are adequate complementary approaches for the asymmetric synthesis of chiral γ-hydroxy-α-amino acids **6**. Overall, the HBPA/Glu/BCAT/AspAT system renders, in some instances, better results mainly due to the selectivity of the α-transaminase BCAT for the 2-oxo acids and its inability to catalyze the transamination of the remaining aldehyde from the aldol addition. Moreover, the reaction can be carried out in whole cells. The HBPA/benzylamine/T039 system is more unspecific, allowing the conversion of various structurally different substrates, such as that of the aldehydes **1** into primary amines. The HBPA/l-Ala/T039 approach with pyruvate recycling is not straightforward and needs an optimization of the activities of the aldolase and transaminase involved to develop an effective process.

Using the strategy developed in this work, an unprecedented number of chiral γ-hydroxy-α-amino acids and the corresponding α-amino-γ-butyrolactones were constructed in two steps with high stereoselectivity from small functionalized aldehydes.

## Supporting Information Available

General methods and protocols for the screening and synthesis, activity determinations, synthesis of starting materials, synthesis of γ-hydroxy-α-amino acids and α-amino-γ-butyrolactones, NMR spectra, and computation methods and modeling (PDF)
