# Supplementary material for: Synthesis of γ-Hydroxy-α-amino Acid Derivatives by Enzymatic Tandem Aldol Addition–Transamination Reactions
Source: ACS Catal. 2021 Apr 2;11(8):4660–9. doi: 10.1021/acscatal.1c00210 (PMC8482765; doi:10.1021/acscatal.1c00210)
Supplement: Supplementary file 1 — cs1c00210_si_001.pdf [file cs1c00210_si_001.pdf]

Supporting Information

Synthesis of γ-Hydroxy-α-Amino Acid Derivatives by Enzymatic Tandem Aldol Addition-Transamination Reactions

Carlos J. Moreno[a], Karel Hernández[a], Simon J. Charnok[b], Samantha Gittings[b], Michael Bolte[c], Jesús Joglar[a], Jordi Bujons[a], Teodor Parella[d], Pere Clapés[a*]

[a]Institute for Advanced Chemistry of Catalonia, Dept. of Biological Chemistry, IQAC-CSIC, Spain.

*Email: pere.clapes@iqac.csic.es

[b]Prozomix Ltd. West End Industrial Estate, Haltwhistle, Northumberland, NE49 9HA, UK.

[c]Institut für Anorganische Chemie, J.-W.-Goethe-Universität, Frankfurt/Main, Germany.

[d]Servei de Ressonància Magnètica Nuclear. Universitat Autònoma de Barcelona, Bellaterra, Spain.

Table of Contents

Materials. 3

Methods. 4

Protein production and purification 8

TLC analysis. 14

Specific rotation. 14

HPLC analysis. 14

NMR analysis. 15

Activity determination of HBPA. 15

Activity determination of (*S*)-selective transaminases. 16

Synthesis of starting material (1c-f, 1i-j, 1l-s). 17

General procedure for the aldol addition of sodium pyruvate to aldehydes (1a-s) catalyzed by HBPA 22

Synthesis of 4-hydroxyesters (*R*-4a-m). 23

X-Ray structures of *R*-4e, *R*-4f and *R*-4j 34

Enzymatic transamination of 4-hydroxy-2-oxoacids (3). Screening of transaminases from Prozomix, using L-Ala and benzyl amine as amino donors. 37

Assay of one-pot biocatalytic cascade synthesis of 4-hydroxy-amino with substrate recycling. .. 45

Assay of one-pot two steps 4-hydroxy-amino acids derivatives using a biocatalytic one-pot two-steps approach using the PLP-Dependent branched-chain amino acid aminotransferase (BCATs) from *Escherichia coli*. **51**

Assay of one-pot biocatalytic cascade synthesis of 4-hydroxy-amino acid derivatives with substrate recycling BCAT/L-Glu/L-Asp/AspAT. **52**

Synthesis of 4-hydroxy-amino acids by tandem HBPA/transaminase and conversion to α-amino-γ-butyrolactone derivatives. **53**

Chemical synthesis of 1-Benzyl 2-methyl (2*S*,4*R*)- and (2*S*,4*S*)-4-hydroxypyrrolidine-1,2-dicarboxylate ((2*S*,4*R*)-15k and (2*S*,4*S*)-15k). **64**

Chemoenzymatic synthesis of 1-benzyl 2-methyl (2*S*,4*R*)-4-hydroxypyrrolidine-1,2-dicarboxylate (15k). **65**

Analysis of the diasteromeric excess of 15k. **66**

NMR spectra. **68**

Chromatograms of the HPLC analysis on chiral stationary phases. **173**

Computational Modeling. **180**

Computational Methods. **195**

References. **196**

Materials.

Glycolaldehyde dimer, sodium pyruvate, benzaldehyde, 2,2-dimethoxyacetaldehyde, chloroacetaldehyde, ethyl glyoxylate, indole-3-carboxaldehyde, L-Ala, L-Asp, L-Glu, L-Val, benzylamine, 2,4′-dibromoacetophenone, benzyl bromide, and 2-(bromomethyl)naphthalene were purchased from Sigma-Aldrich. (*S*)-Cbz-*N*-2-Aminopropanal (**1q**) used in this study was synthesized in our lab using procedures published in previous works.[1] Synthetic oligonucleotides were purchased from Eurofins Genomics. All reagents for molecular biology were from Life Thermo Scientific. Culture media components for *E. coli* were from Pronadisa (Madrid, Spain). Antibiotics, IPTG and L-arabinose were from Carl Roth. High-density IDA-Agarose 6BCL nickel charged was from GE Healthcare Life Science. Water for analytical HPLC was obtained from an Arium Pro ultrapure water purification system (Sartorius Stedim Biotech) and the rest of solvents used in this work were of analytical or HPLC grade. Bacterial strains, oligonucleotides, and plasmids used in this study are listed in **Table S1**.

Table S1. Strains, plasmids, and oligonucleotides used in this study.

**Strains**	**Relevant genotype**
E. coli Nova Blue (used for plasmid preparation)	*end*A1, *hsd*R17 (rB^+, mB^+), *sup*E44, *thi*1, *rec*A1, *gyr*A96, *rel*A1, *lac* F′ [proA+ B+, *lac*I^q ZΔM15::Tn10] (Tet^R).
E. coli Bl21-AI (used for protein expression)	F^- *ompT gal dcm lon hsdS*$_B$($r_B^- m_B^-$)[$malB^+$]$_{K\text{-}12}$(λ^S) *araB*::*T7RNAP-tetA*
E. coli M15 [pREP4] (used for protein expression)	nal^s , str^s , rif^s , thi^- , lac^- , ara^+ , gal^+ , mtl^- , F^- , $recA^+$, uvr^+, lon^+, KmR.
**Oligonucleotides**	**Sequences**
Primer 1 (HBPA)	Forward: 5’ATGTCGT**CATATG**AGAGGATCGC 3’
Primer 2 (HBPA)	Reverse:5’GAACCGAT**GGTACC**TTATTTGCTATACTTCG 3’
Primer 3 (HBPA)	Forward: 5’ATGAGAGGATCGCATCACC 3’
Primer 4 (HBPA)	Reverse:5’TTATTTGCTATACTTCG 3’
HBPA H205A	CAC(H)→GCG(A) Forward: 5’CGAACATCCGTTTTCTGCCG**GCG**GAGGACGATTACTATGCGG3’
BCAT(53)	Forward: 5’ ATGCATG**CCATGG**GAATGACCACGAAGAAAGCTG 3’
BCAT(35)	Reverse:5’ATACGAT**AGATCT**TTGATTAACTTGATCTAACCAGCCCC 3’
AspAT(53)	Forward: 5’ AGTCAGCT**GGATCC**ATGTTTGAGAACATTACCGC 3’
AspAT(35)	Reverse: 5’CATACT**AAGCTT**AGTGATGGTGATGGTGATGAGATCTCAGCACTG CCACAATCGC 3’
**Plasmids**	**Relevant genetic characteristics**
pETDuet-1	The vector encodes two multiple cloning sites, each of which is preceded by a T7 promoter, *lac* operator and ribosome binding site. The vector also

	carries the pBR322-derived ColE1 replicon, *lacI* gene and ampicillin resistance gene.
pETDuet-1- *HBPA*	Plasmid containing the synthetic gene *nahE* (1008 bp), codes for *trans-O*-hydroxybenzylidenepyruvate hydratase-aldolase (HBPA , EC 4.1.2.45) from *Pseudomonas putida* .This study**.**
pETDuet-1-*HBPA H205A*	Plasmid containing the *nahE* (CAC→GCG) gene, codes for HBPA H205A. This study.
pQE-60	The vector encodes one multiple cloning site preceded by a T5 promoter, *lac* operator and ribosome binding site. The vector also carries the ColE1 replicon and ampicillin resistance gene.
pQE60 *BCAT*	Plasmid containing the *ilvE* gene (960 bp), codes for branched-chain amino acid aminotransferase (BCAT EC 2.6.1.42) from *E. coli* K-12. This study.
pQE60 *AspAT*	Plasmid containing the *aspC* gene (1227 bp), code for aspartate amino transferase (AspAT EC 2.6.1.1) from *E. coli* K-12. This study.
pQE60 *BAL*	Previous work.[2]
pQE40 MBP-YfaU	Previous work.[3]

Methods.

Enzymes cloning.

HBPA: The optimized synthetic gene *nahE* (**Figure S1**) insert in a pQE 40 (GenScript's gene synthesis service) was re-cloned into the second cloning site of a pETDuet-1 plasmid, using the restriction enzymes *Nde*I and *Kpn*I (**Figure S2**). All DNA manipulation were performed using routine procedures of molecular biology.[3]

A)

```
ATGCTGAACAAGGTGATCAAAACCACCCGTCTGACCGCGGAGGACATCAACGGTGCGTGGACCATTATGCCGACC
CCGAGCACCCCGGATGCGAGCGATTGGCGTAGCACCAACACCGTTGACCTGGATGAAACCGCGCGTATCGTGGAG
GAACTGATTGCGGCGGGTGTTAACGGCATTCTGAGCATGGGCACCTTTGGCGAGTGCGCGACCCTGACCTGGGAG
GAAAAGCGTGACTACGTGAGCACCGTGGTTGAAACCATCCGTGGTCGTGTTCCGTATTTCTGCGGCACCACCGCG
CTGAACACCCGTGAGGTGATCCGTCAGACCCGTGAACTGATCGATATTGGTGCGAACGGCACCATGCTGGGTGTG
CCGATGTGGGTTAAGATGGACCTGCCGACCGCGGTTCAATTTTACCGTGATGTTGCGGGTGCGGTTCCGGAGGCG
GCGATCGCGATTTATGCGAACCCGGAAGCGTTCAAATTTGATTTCCCGCGTCCGTTCTGGGCGGAGATGAGCAAG
ATCCCGCAGGTGGTTACCGCGAAATACCTGGGTATTGGCATGCTGGACCTGGATCTGAAACTGGCGCCGAACATC
CGTTTTCTGCCGCACGAGGACGATTACTATGCGGCGGCGCGTATCAACCCGGAACGTATTACCGCGTTCTGGAGC
AGCGGTGCGATGTGCGGTCCGGCGACCGCGATTATGCTGCGTGACGAGGTGGAACGTGCGAAGAGCACCGGTGAT
TGGATCAAGGCGAAAGCGATTAGCGACGATATGCGTGCGGCGGACAGCACCCTGTTTCCGCGTGGTGATTTTAGC
GAGTTCAGCAAGTACAACATCGGCCTGGAAAAAGCGCGTATGGATGCGGCGGGTTGGCTGAAAGCGGGTCCGTGC
CGTCCGCCGTACAACCTGGTGCCGGAAGATTATCTGGTTGGTGCGCAAAAGAGCGGTAAAGCGTGGGCGGCGCTG
CACGCGAAGTATAGCAAA
```

B)

MLNKVIKTTRLTAEDINGAWTIMPTPSTPDASDWRSTNTVDLDETARIVEELIAAGVNGILSMGTFGECATLTWEEKRDYV
STVVETIRGRVPYFCGTTALNTREVIRQTRELIDIGANGTMLGVPMWVKMDLPTAVQFYRDVAGAVPEAAIAIYANPEAFK
FDFPRPFWAEMSKIPQVVTAKYLGIGMLDLDLKLAPNIRFLPHEDDYYAAARINPERITAFWSSGAMCGPATAIMLRDEVE
RAKSTGDWIKAKAISDDMRAADSTLFPRGDFSEFSKYNIGLEKARMDAAGWLKAGPCRPPYNLVPEDYLVGAQKSGKAW
AALHAKYSK

Figure S1. **A)** Optimized gene sequence (GenScript's gene synthesis service) and **B)** amino acid sequence of HBPA (NCBI database accession number: WP_011475383.1).

A)

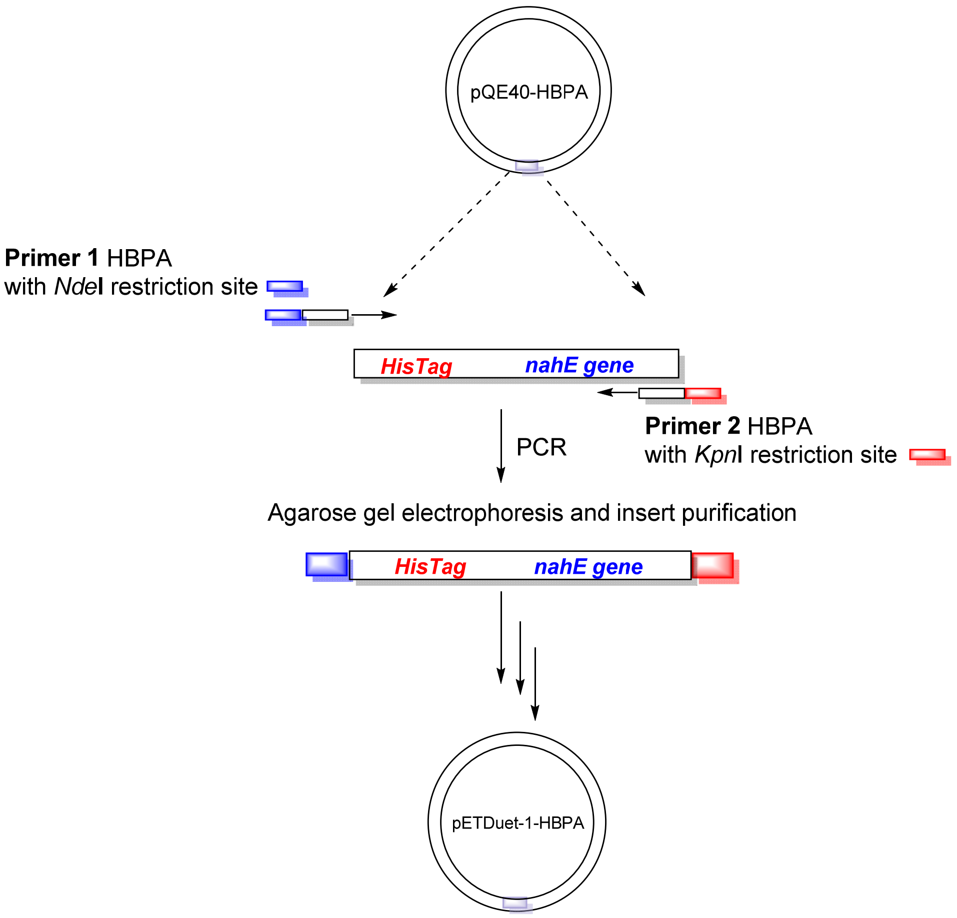

B)

MRGSHHHHHHGSMLNKVIKTTRLTAEDINGAWTIMPTPSTPDASDWRSTNTVDLDETARIVEELIAAGVNGILSMGTFGEC
ATLTWEEKRDYVSTVVETIRGRVPYFCGTTALNTREVIRQTRELIDIGANGTMLGVPMWVKMDLPTAVQFYRDVAGAVP
EAAIAIYANPEAFKFDFPRPFWAEMSKIPQVVTAKYLGIGMLDLDLKLAPNIRFLPHEDDYYAAARINPERITAFWSSGAMC
GPATAIMLRDEVERAKSTGDWIKAKAISDDMRAADSTLFPRGDFSEFSKYNIGLEKARMDAAGWLKAGPCRPPYNLVPED
YLVGAQKSGKAWAALHAKYSK

Figure S2. **A)** Schematic representation of the cloning method of HBPA in the pETDuet-1 plasmid. **B)** Protein sequence of His tag-HBPA. 6x His tag (red) and HBPA (blue).

BCAT and **AspAT** (NCBI database accession number ABD20288.1 and NP_415448 respectively). The genes *ilvE* and *aspC* from *E. coli* K-12 were amplified by PCR from genomic DNA and cloned into pQE 60 using *Nco*I/*Bgl*II restriction enzymes (primers BCAT(53) and BCAT(35)) and *Bam*HI/*Hind*III restriction enzymes (primers AspAT(53)

and AspAT(35)) respectively (**Table S3**). All DNA manipulation were performed using routine procedures of molecular biology.[3]

A)

MGMTTKKADYIWFNGEMVRWEDAKVHVMSHALHYGTSVFEGIRCYDSHKGPVVFRHREHMQRLHDSAKIYRFPVSQSI
DELMEACRDVIRKNNLTSAYIRPLIFVGDVGMGVNPPAGYSTDVIIAAFPWGAYLGAEALEQGIDAMVSSWNRAAPNTIPT
AAKAGGNYLSSLLVGSEARRHGYQEGIALDVNGYISEGAGENLFEVKDGVLFTPPFTSSALPGITRDAIIKLAKELGIEVRE
QVLSRESLYLADEVFMSGTAAEITPVRSVDGIQVGEGRCGPVTKRIQQAFFGLFTGETEDKWGWLDQVNQRSHHHHHH

B)

MGGSMFENITAAPADPILGLADLFRADERPGKINLGIGVYKDETGKTPVLTSVKKAEQYLLENETTKNYLGIDGIPEFGRCT
QELLFGKGSALINDKRARTAQTPGGTGALRVAADFLAKNTSVKRVWVSNPSWPNHKSVFNSAGLEVREYAYYDAENHTL
DFDALINSLNEAQAGDVVLFHGCCHNPTGIDPTLEQWQTLAQLSVEKGWLPLFDFAYQGFARGLEEDAEGLRAFAAMHK
ELIVASSYSKNFGLYNERVGACTLVAADSETVDRAFSQMKAAIRANYSNPPAHGASVVATILSNDALRAIWEQELTDMRQ
RIQRMRQLFVNTLQEKGANRDFSFIIKQNGMFSFSGLTKEQVLRLREEFGVYAVASGRVNVAGMTPDNMAPLCEAIVAVL
RSHHHHHH

Figure S3. **A)** Protein sequence of BCAT-His tag and **B)** protein sequence of AspAT-His tag. BCAT (blue), AspAT (green) and 6x His tag (red).

Site-directed mutagenesis

The HBPA gene mutation H205A was introduced with the Megaprimer site-directed mutagenesis strategies (**Figure S4**) with PCR procedure described in **Table S2**.

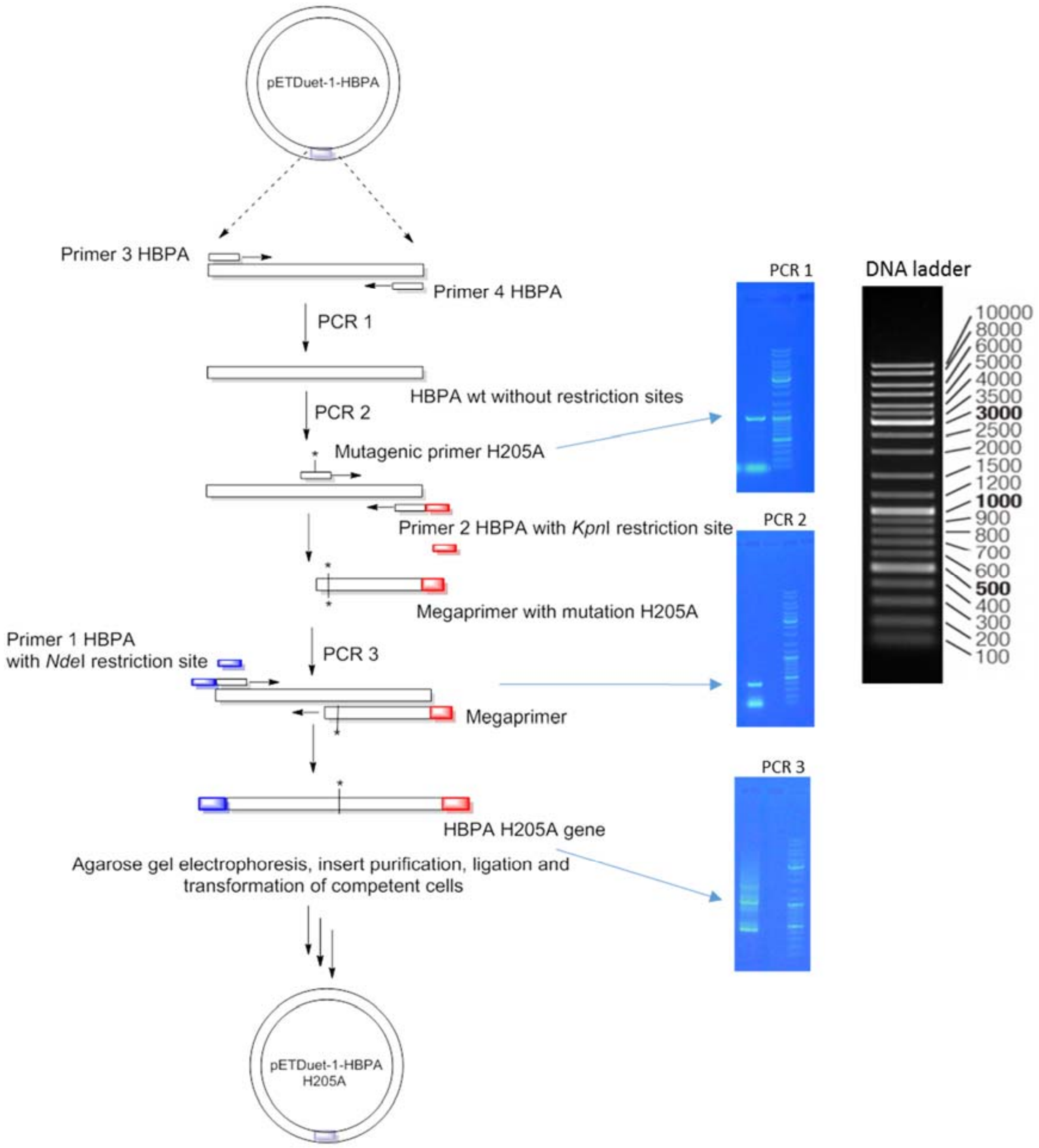

Figure S4. Schematic illustration of the megaprimer method for site-directed mutagenesis. The first-round PCR (PCR 1) was performed using external forward (Primer 3 HBPA) and reverse primer (Primer 4 HBPA), neither of one with restriction site. The second-round PCR (PCR 2) was performed using the internal mutagenic primer (Primer HPBA H205A) and external primer (Primer 2 HBPA) with *Kpn*I restriction site. The third-round PCR (PCR 3) was performed using external primer (Primer 1 HBPA) with *Nde*I restriction site and the product of PCR2 (“megaprimer”).

Table S2. Megaprimer site-directed mutagenesis PCR protocols.

a) PCR 1

**Components**	**V/μL**	**Final concentration (50 μL total volume)**	**PCR cycles**		
Water, nuclease free	35.5		Step	T/°C	Time/s
5X Green Buffer[a]	10		Initial denaturation	98	180
dNTP mix (10 mM each)	1	0.2 mM each	35 PCR cycles	98	10
F1, forward primer (25 μM)	1	0.5 μM		55	30
R, reverse primer (25 μM)	1	0.5 μM		72	20
Template DNA (1 ng μL^{-1})	1	0.01 ng μL^{-1}	Final extension	72	600
DNA Polymerase (2 U μL^{-1})[b]	0.5	0.02 U μL^{-1}		4	Hold

b) PCR 2

**Components**	**V/μL**	**Final concentration (50 μL total volume)**	**PCR cycles**		
Water, nuclease free	35.5		Step	T/°C	Time/s
5X Green Buffer[a]	10		Initial denaturation	98	180
dNTP mix (10 mM each)	1	0.2 mM each	35 PCR cycles	98	10
F1, forward primer (25 μM)	1	0.5 μM		55	30
R, reverse primer (25 μM)	1	0.5 μM		72	6
Template DNA (1 ng μL^{-1})	1	0.01 ng μL^{-1}	Final extension	72	600
DNA Polymerase (2 U μL^{-1})[b]	0.5	0.02 U μL^{-1}		4	Hold

c) PCR 3

**Components**	**V/μL**	**Final concentration (50 μL total volume)**	**PCR cycles**		
Water, nuclease free	35.5		Step	T/°C	Time/s
5X Green Buffer[a]	10		Initial denaturation	98	180
dNTP mix (10 mM each)	1	0.2 mM each	35 PCR cycles	98	10
F1, forward primer (25 μM)	1	0.5 μM		55	30
R, reverse primer (25 μM)	1	0.5 μM		72	300
Template DNA (1 ng μL^{-1})	1	0.01 ng μL^{-1}	Final extension	72	600
DNA Polymerase (2 U μL^{-1})[b]	0.5	0.02 U μL^{-1}		4	Hold

[a]5X Phusion Green HF Buffer (Thermo Scientific). [b]The concentration of Megaprimer depends on the efficiency of the PCR2. Megaprimer size: 418 bp (H205A). [c]Fusion Green High-Fidelity DNA Polymerase (Thermo Scientific).

Protein production and purification

General procedure for HBPA, BCAT and AspAT: Competent *E. coli* strain cells were transformed with the correspondent plasmid (**Table S3**) and grown in LB medium with

selected antibiotics at 37 °C on a rotary shaker at 200 rpm. A final optical density at 600 nm (OD_{600}) of 2–3 was usually achieved. An aliquot of the pre-culture (20 mL) was transferred into a baffled shaker flask (2 L) containing LB medium (1 L) plus selected antibiotics and antifoam SE-15 (0.02% (v/v)), and incubated at 37 °C with shaking at 200 rpm. During the middle exponential phase growth ($DO_{600} \approx 0.5$-0.8), the temperature was lowered to 30 °C to minimize inclusion bodies formation and then proteins expression were induced (**Table S3**). After 12-16 h, cells from the induced-culture broths (5 L, 16-22 g of cells) were centrifuged (2500 *g* for 45 min at 4 °C). The pellet was re-suspended in the lysis buffer (400 mL). Cells were lysed using a cell disrupter (Constant Systems) and cellular debris were removed by centrifugation (35000 *g* for 45 min at 4 °C). The clear supernatant was applied to a cooled HR 16/40 column (GE Healthcare) packed with Nickel Sepharose™ High Performance (50 mL bed volume, GE Healthcare) at 6 mL min^{-1} and washed with lysis buffer (400 mL) at 6 mL min^{-1}. The protein was eluted with elution buffer (200 mL) at a flow rate of 6 mL min^{-1} (**Table S3, Figure S5**). The recombinant proteins were dialyzed against 1 L of dialysis buffer (3x1 L 24 h each). The dialyzed solution obtained (90-140 mL) was stored at - 20 °C. The glycerol in dialysis buffer minimizes protein precipitation after purification. Protein concentrations were determined by absorption at 280 nm using extinction coefficients calculated by ProtParam (http://expasy.org/tools/protparam.html), HBPA *wt* and H205A variant (1.7 mg mL^{-1}), BCAT (1.40 mg mL^{-1}) and AspAT (0.98 mg mL^{-1}), assuming all Cys residues are reduced.

Table S3. Conditions for expression and purification of protein using in this work.

Enzyme	Expression conditions		Purification conditions		
	Cellular expression system	Induction conditions and selection antibiotics	Lysis buffer	Elution buffer	Dialysis buffer
HBPA	*E. coli* BL21 AI	IPTG 1 mM L-arabinose (0.2% m/v) T =30 ºC Ampicillin (100 μg mL^{-1})	50 mM NaH_2PO_4 buffer pH 8.0, NaCl (300 mM), imidazole (20 mM), glycerol (10 % v/v) and DNAse	50mM NaH_2PO_4 buffer pH 8.0, NaCl (300 mM), imidazole (250mM) and glycerol (10 % v/v).	20 mM TEA buffer pH 6.5, NaCl (50 mM), EDTA (0.5 mM) and glycerol (50% v/v).
BCAT	*E. coli* M15 [pREP-4]	IPTG 50 μM T = 20ºC Ampicillin (100 μg mL^{-1}) Kanamycin (25 μg mL^{-1})			50mM NaH_2PO_4 buffer pH 7.0, NaCl (100 mM), PLP (0.01 mM) and glycerol (50% v/v).
AspAT					

General procedure for MBP-YfaU and BAL. Protein expression and purification were performed as described in previous work.[2-3]

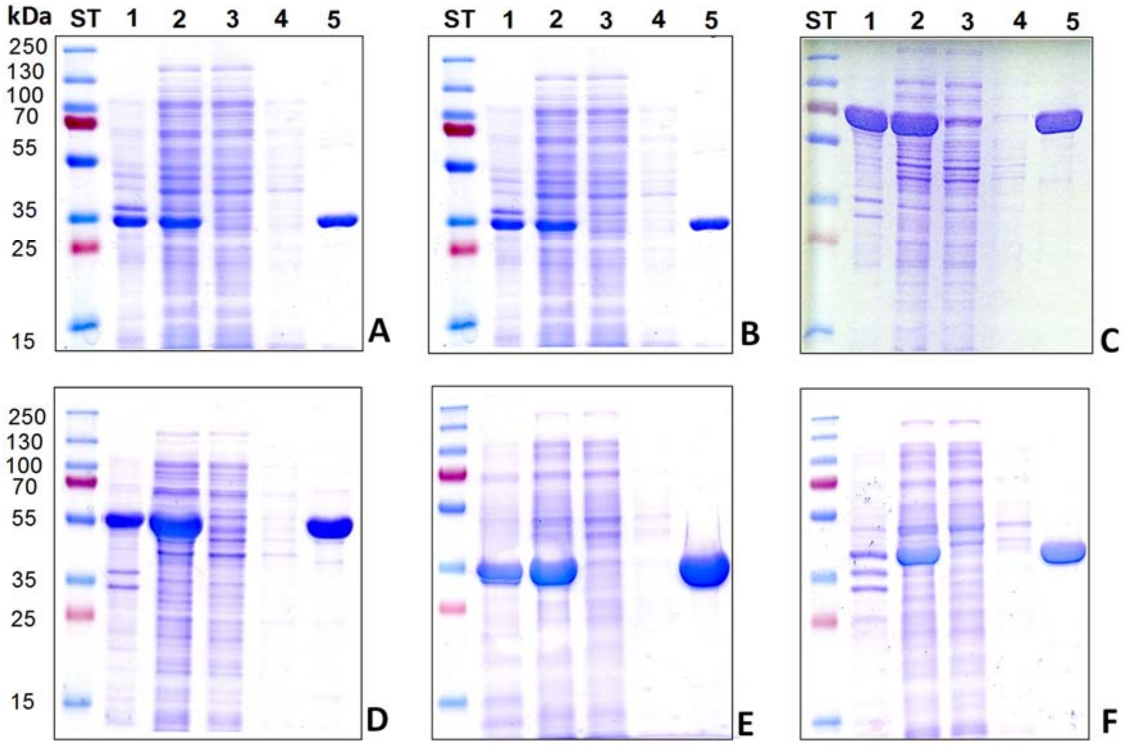

Figure S5. Analysis of enzyme purification steps by Coomassie Blue-stained SDS-PAGE. *Wild-type* HBPA (**A**), H205A variant (**B**), MBP-YfaU (**C**), BAL (**D**), BCAT (**E**),

and AspAT (**F**). In each case, the gel was loaded with samples from pellet after lysis (lane 1), supernatant of lysis (lane 2), flow-through fraction (lane 3), wash fraction (lane 4) and elution fraction (lane 5). The molecular masses of the proteins in the Standard Molecular Weight Marker (ST) are as indicated. The predicted molecular mass of HBPA *wild-type* and H205A variant is 38 kDa. MBP-YfaU (72.3 kDa), BAL (60.1 kDa), BCAT (35.3 kDa), and AspAT (45 kDa).

Table S4. Yield of enzyme after purification and inclusion bodies formation.

Enzyme	Inclusion bodies formation/(%)[a]	Yield[b]
HBPA *wild-type*	36	72
HBPA H205A	40	85
MBP-YfU	47	168
BAL	30	234
BCAT	39	396
AspAT	29	168

[a]Formation of inclusion bodies was estimated by color densitometry in SDS-PAGE using Image J version 1.53a.[4] [b]mg protein L^{-1} culture medium.

Electrospray mass spectrometry of proteins.

Glycerinated proteins were diluted in water/formic acid (0.1% v/v) (1 mg protein mL^{-1}, final concentration in the solution) and glycerol and salts were removed with PD MiniTrap G-10 of (GE Healthcare). The samples were eluted in water/formic acid (0.1% v/v, 0.5 mL) and injected (5 μL) to an Acquity UPLC BEH C4, 1.7 μm, 2.1x50 mm column connected to a mass spectrometer ESI-TOF instrument (LCT PremierWaters, Milford, MA, USA), equipped with a 4 GHz time-to-digital converter (TDC) with a dual ESI source (LockSpray). The second sprayer provided the lock mass calibration with leucine enkephalin (m/z 556.2771). The ESI-TOF was operated in the W-optics mode, thus providing a mass resolution of at least 10 000 full width at half maximum (FWHM). The acquisition time per spectrum was set to 0.2 s, and the mass range was from 500 to 1800 Da. Data were acquired using a cone voltage of 50 V, capillary voltage of 3000 V, desolvation temperature of 35 °C, and source temperature of 100 °C. The desolvation gas flow was set at 400 L h^{-1} and the cone gas flow was set at 30 L h^{-1}. The solvent system used for the elution was: solvent (A): 0.1% formic acid in CH_3CN/H_2O (70/30, v/v) and solvent (B): 0.1% (v/v) formic acid in H_2O, gradient elution from 28% A to 100% A in 25 min, 100% A for 2 min and 28% A for 18 min, flow rate 0.2 mL min^{-1}. Mass Lynx

4.1 software (Waters, Milford, MA, USA) was used for data acquisition and processing. Magtran software 4, kindly provided by Dr. Zhongqi Zhang (Amgen, Inc.; Thousand Oaks, CA), was used for molecular weight deconvolution from ESI-MS spectra of proteins (**Table S5**/**Figure S6)**.

Table S5. Molecular weight determination by ESI-MS of HBPA.

Enzyme variant	Molecular weight calculated/Da	Δm/Da (HBPA *wt* – HBPA H205A) Theoretical	Δm/Da (HBPA *wt* – HBPA H205A) Experimental
Wild-type	38045	66	66
H205A	37979		

HBPA *wild-type*

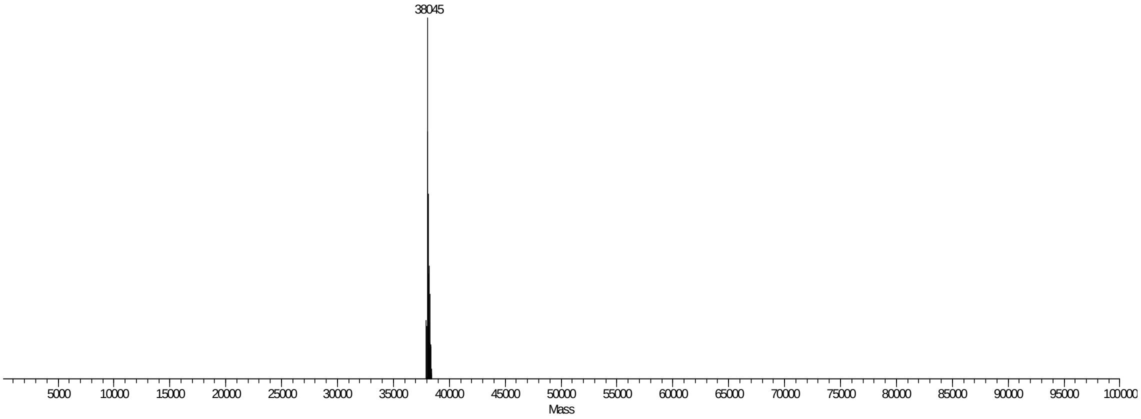

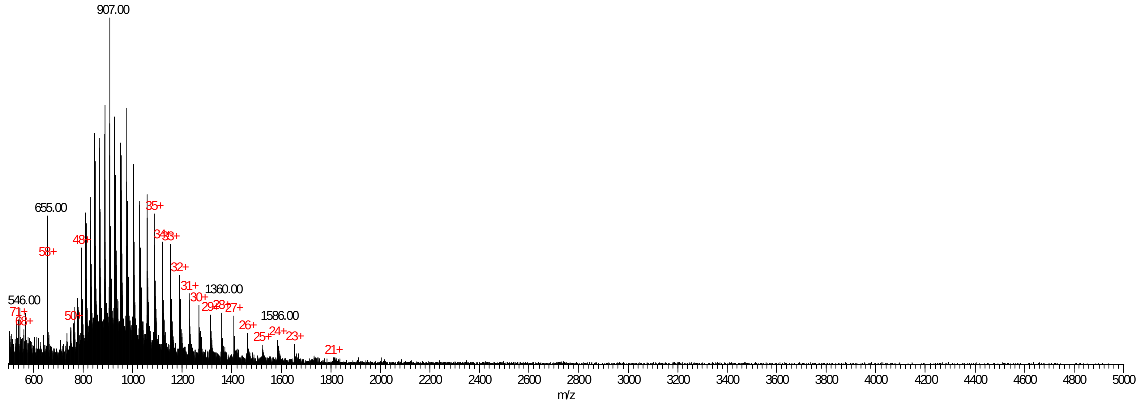

HBPA H205A

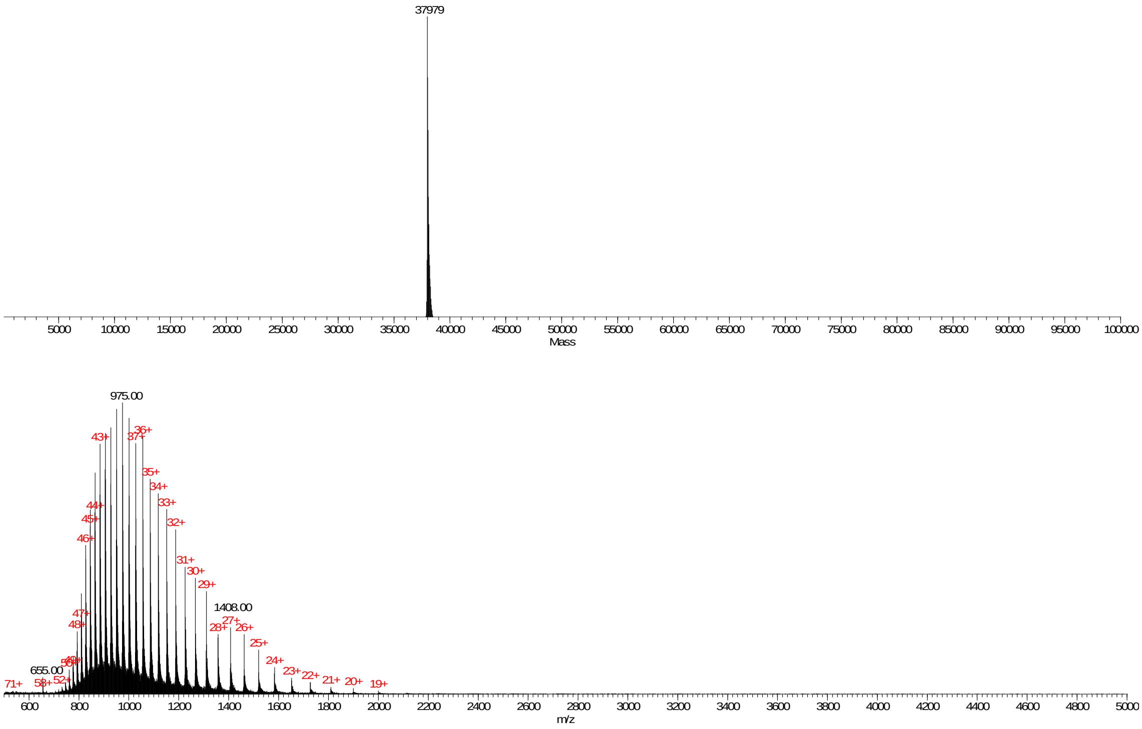

Figure S6: ESI/TOF spectra and deconvolution spectra of HBPA and H205A variant.

TLC analysis.

Thin layer chromatography was performed using precoated silica gel plates with or without fluorescent indicator UV254 (Macherey-Nagel GmbH & Co. KG, Kieselgel 60). TLC without fluorescent visualization was stained using ceric ammonium molybdate, ninhydrin or *p*-anisaldehyde.

Specific rotation.

Specific rotation values were measured with a Perkin Elmer Model 341 (Überlingen, Germany) (Na Lamp, 589 nm). Products (16 to 63 mg) were dissolved in H_2O, MeOH or $CHCl_3$ (1.5 mL) and the samples were analyzed at room temperature 1.0 dm cell with polarized light. Rotation values are described in each compound.

HPLC analysis.

HPLC analysis was performed on a RP-HPLC XBridge® C18, 5 µm, 4.6 × 250 mm column (Waters). The solvent system used was: solvent (A): 0.1% (v/v) trifluoroacetic acid (TFA) in H_2O and solvent (B): 0.095% (v/v) TFA in CH_3CN/H_2O 4:1, flow rate 1 mL min^{-1}, detection at 215 nm and column temperature at 30 °C.

The amount of products and substrates were quantified from the peak areas using an external standard methodology. Reaction monitoring for aliphatic aldehydes (**1**), sodium pyruvate (**2**) and aldol adduct (**3**) were carried out as follows:

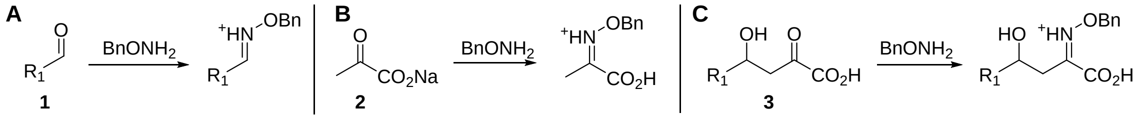

a) Analysis of the compounds bearing carbonyl groups. Samples were withdrawn from the reaction mixture (10 µL) and mixed with a solution of $BnONH_2$ (50 µL of a 0.13 mM stock solution in pyridine:methanol:water 33:15:2) during 5 min, and then diluted with methanol (500 µL). After centrifugation, they were analyzed by HPLC. Elution conditions: most samples were analyzed using a gradient elution from 10 to 100% B over 30 min.

b) Analysis of the compounds bearing aromatic moieties. Samples were withdrawn from the reaction mixture (50 µL) and diluted with methanol (500 µL). After

centrifugation, they were analyzed by HPLC. Elution conditions: most samples were analyzed with a gradient elution from 10 to 100% B over 30 min.

c) Analysis of the compounds bearing amino groups. Samples were withdrawn from the reaction mixture (10 μL) and mixed with sodium borate buffer (20 μL of a 100 mM stock solution pH 10.0). Then, a solution of *N*-(benzoyloxy)succinimide (Bz-OSu) (50 μL of a 150 mM stock solution in CH_3CN, 7.5 μmol, 7.5 eq) was added. After incubation at room temperature for 30 min, samples were diluted with AcOH:CH_3CN 10:90 (300 μL), centrifuged, and subsequently analyzed by HPLC. Elution conditions: most samples were analyzed with a gradient elution from 10 to 100% B over 30 min.

d) Chiral HPLC: Enantiomeric excesses (ee) were determined by HPLC on chiral stationary phase. Compounds **4a** and **4a'** were analyzed on a CHIRALPAK® ID, **4b**, **4e-f**, **4h-j** and **4m** on a CHIRALPAK® IB, and **4d**, **4g** and **4l** on a CHIRALPAK® IC, columns (46 x 250 mm, 5 μm). All samples were eluted with hexane/isopropanol mixtures at flow rate 1 mL min^{-1}, column temperature 30 °C and diode array detection (215-350 nm). Specific elution conditions are described in each compound.

NMR analysis.

Routine ^1H (400 MHz) and ^{13}C (101 MHz) NMR spectra of compounds were recorded with a Varian Mercury-400 spectrometer. Full characterization of the described compounds was performed using typical gradient-enhanced 2D experiments: COSY, HSQC, NOESY, HMBC, and selective 1D nOe recorded under routine conditions.

Activity determination of HBPA

The activity was determined using the aldol condensation of sodium pyruvate to indole-3-carboxyaldehyde using a continuous assay method.[5] One unit of activity was defined as the amount of HBPA that catalyzes the formation of 1 μmol sodium (*E*)-4-(1*H*-indol-3-yl)-2-oxobut-3-enoate per min at 30 °C, in 50 mM sodium phosphate buffer pH 7.0 containing 3% of MeOH (v/v). The assay was performed as follows: to a solution of sodium pyruvate (20 μL of a 2 M stock solution in H_2O pH 6.5-7.0, prepared just before use) and indole-3-carboxyaldehyde (200 μL of a 0.075 mM solution in 50 mM sodium phosphate buffer pH 7.0, and 3% of MeOH (v/v)), a solution of the enzyme (80 μL, of stock solution between 0.0625 – 4 mg mL^{-1} in 20 mM TEA buffer pH 6.8, containing

NaCl (50 mM), EDTA (0.5 mM), and 50% of glycerol (v/v)) was added. The total reaction volume was 0.3 mL. Reaction was monitoring spectrophotometrically at 367 nm during 15 min measuring each 30 s.

Activity determination of (*S*)-selective transaminases.

The activity of **T039** was determined using the transamination of α-ketoglutarate to L-Glu, using L-Ala as amine donor. Pyruvate formed was measured with a coupled assay with lactate dehydrogenase (LDH), measuring the consumption of NADH. One unit of activity was defined as the amount of transaminase that catalyzes the formation of 1 μmol of L-Glu per min at 30 °C in 50 mM sodium phosphate buffer pH 7.0, containing PLP (0.05 mM). The assay protocol was as follows: to a solution mixture of α-ketoglutarate and L-Ala (180 μL of a 16.6 mM α-ketoglutarate, and a 83.3 mM L-Ala stock solutions, containing NADH (0.16 mM) and PLP (0.08 mM) stock solution in 50 mM sodium phosphate buffer pH 7.0), and LDH (20 μL of 140 U mL^{-1}stock solution, 4.2 U in the reaction), T039 (100 μL of a 0.006 – 0.09 mg mL^{-1} stock solutions of free cell extract powder in 50 mM sodium phosphate buffer pH 7.0) was added. Total final volume was 0.3 mL. Reaction was monitoring spectrophotometrically at 340 nm during 10 min measuring each 30 s.

Activity of **AspAT** was determined using the transamination of α-ketoglutarate to L-Glu, using L-Asp as amine donor. Oxalacetic acid formed was measured with a coupled assay with lactate dehydrogenase (LDH) and Malate dehydrogenase (MDH), measuring the consumption of NADH. One unit of activity was defined as the amount of transaminase that catalyzes the formation of 1 μmol of L-Glu per min at 30 °C in 50 mM sodium phosphate buffer pH 7.0, containing PLP (0.05 mM). The assay protocol was as follows: to a solution mixture of α-ketoglutarate and L-Asp (250 μL of a 12 mM α-ketoglutarate, and 240 mM L-Asp stock solutions, containing NADH (0.19 mM) and PLP (0.12 mM) stock solution in 50 mM sodium phosphate buffer pH 7.0, LDH (2.4 μL of 9968 U mL^{-1} stock solution, 25 U in the reaction) and MDH (10 μL of 6000 U mL^{-1}stock solution, 5 U in the reaction), AspAT (50 μL, of stock solution between 0.00024 - 1 mg mL^{-1} in 50mM NaH_2PO_4 buffer pH 7.0, NaCl (100 mM), PLP (0.01 mM) and glycerol (50% v/v)) was added. Total final volume was 0.3 mL. Reaction was monitoring spectrophotometrically at 340 nm during 15 min measuring each 30 s.

Activity of **BCAT** was determined using the transamination of α-ketoglutarate to L-Glu, using L-Val as amine donor. L-Glu formed was measured with a coupled assay with Glutamate dehydrogenase (GDH) measuring the formation of NADH.[6] One unit of activity was defined as the amount of transaminase that catalyzes the formation of 1 μmol of L-Glu per min at 30 °C in 100 mM sodium phosphate buffer pH 8.0, containing PLP (0.05 mM). The assay protocol was as follows: to a solution mixture of α-ketoglutarate and L-Val (250 μL of 6 mM α-ketoglutarate, and 12 mM L-Val stock solution, containing NAD^+ (0.6 mM) and PLP (0.6 mM) in 100 mM sodium phosphate buffer pH 8.0, GDH (1.8 mg of 13 U mg^{-1}stock solution, 2 U mL^{-1} in the reaction), BCAT (50 μL, of stock solution between 0.0078 – 1 mg mL^{-1} in 50 mM NaH_2PO_4 buffer pH 7.0, NaCl (100 mM), PLP (0.01 mM) and glycerol (50% v/v)) was added. Total final volume was 0.3 mL. Reaction was monitoring spectrophotometrically at 340 nm during 15 min measuring each 30 s.

Synthesis of starting material (1c-f, 1i-j, 1l-s).

2-(Benzylthio)acetaldehyde (**1c**). Typical procedure: The title compound was prepared by oxidation the corresponding alcohol precursor. 2-(Benzylthio)ethanol (1.2 g, 7.1 mmol) was dissolved in EtOAc (250 mL) and 2-iodoxybenzoic acid (IBX) (4.0 g, 14.3 mmol, 2 eq) was added. The mixture was refluxed for 6 h. Then, the reaction mixture was allowed to cold down to room temperature (rt), filtrated and the filtrate was washed with $NaHCO_3$ 5%, brine (3 x 100 mL), dried over anhydrous $MgSO_4$ and concentrated under vacuum to afford the compound **1c** as a yellow oil, (959 mg, 81%). The spectral properties of this product agreed with those reported in the literature.[7] ^{1}H NMR (400 MHz, $CDCl_3$) δ 9.40 (t, *J* = 2x3.4 Hz, 1H), 7.34 – 7.21 (m, 5H), 3.61 (s, 2H), 3.07 (d, *J* = 3.4 Hz, 2H). ^{13}C NMR (101 MHz, $CDCl_3$) δ 193.8, 129.2, 128.6, 127.4, 40.2, 35.5.

2-Methoxyacetaldehyde (**1d**). To a solution of 1,1,2-trimethoxyethane (5.0 g, 41.6 mmol) in H_2SO_4 (20 mL, 2 M), was stirred at rt for 1 h. $CaCO_3$ solid was added until neutralization. After that, reaction mixture was filtrated through Celite®. Aldehyde **1d** was used without any further purification. The concentration of the compound in the final solution resulted to be 0.4 M, as determined by HPLC analysis using pre-column derivatization with *O*-benzylhydroxylamine.

2-Phenoxyacetaldehyde (**1e**). The title compound **1e** (oil, 1.4 g, 92%) was prepared following the procedure described for (**1c**) starting from 2-phenoxyethanol (1.5 g, 10.9 mmol). The spectral properties of this product matched those reported in the literature.[8] ^{1}H NMR (400 MHz, $CDCl_3$) δ 9.86 (t, *J* = 2x1.2 Hz, 1H), 7.34 – 7.19 (m, 2H), 7.05 – 6.78 (m, 3H), 1.55 (s, 2H). ^{13}C NMR (101 MHz, $CDCl_3$) δ 199.4, 157.6, 129.6, 122.0, 114.5, 72.6.

2-Methyl-2-phenoxypropanal (**1f**).

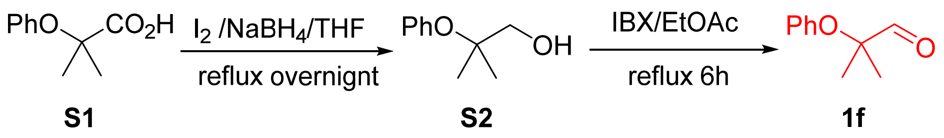

To a suspension of 2-methyl-2-phenoxypropanoic acid **S1** (2.0 g, 11.1 mmol) and $NaBH_4$ (1.3 g, 33.3 mmol, 3 eq) in anhydrous THF (100 mL), I_2 (3.4 g, 13.3 mmol, 1.2 eq) was slowly added under N_2 atmosphere. When all the added I_2 was consumed, the reaction was stirred overnight under reflux. The mixture was allowed to cold down to rt, and methanol was added until there was no gas formation. The solvent was removed under vacuum affording a white solid. To this residue, aqueous NaOH 5% (200 mL) was added and solution was stirred for 3 h, and extracted with CH_2Cl_2 (3 x 100 mL). The organic phases were combined, washed with water (3x100 mL), $NaHCO_3$ 20% (3 x 100 mL), brine (3 x 100 mL) and dried over anhydrous magnesium sulfate. The solvent removed under vacuum affording 2-methyl-2-phenoxypropanol **S2** as an oil (1.7 g, 90%). ^{1}H NMR (400 MHz, $CDCl_3$) δ 7.30 – 7.23 (m, 2H), 7.09 (t, *J* = 2x7.4 Hz, 1H), 6.99 (d, *J* = 1.4 Hz, 1H), 6.96 (d, *J* = 1.2 Hz, 1H), 3.58 (s, 2H), 1.26 (s, 6H). ^{13}C NMR (101 MHz, $CDCl_3$) δ 154.5, 129.1, 123.8, 80.6, 70.3, 23.1. The title compound **1f** (oil, 932 mg, 57%) was prepared following the procedure described for **1c** starting from **S2** (1.7 g, 10.0 mmol). ^{1}H NMR (400 MHz, $CDCl_3$) δ 9.84 (s, 1H), 7.29 – 7.19 (m, 2H), 7.02 (t, *J* = 2x7.9 Hz, 1H), 6.85 (d, *J* = 0.8 Hz, 1H), 6.83 (d, *J* = 1.0 Hz, 1H), 1.41 (s, 6H). ^{13}C NMR (101 MHz, $CDCl_3$) δ 203.4, 155.1, 129.4, 122.8, 119.8, 83.1, 21.8.

Isopropyl 2-oxoacetate (**1i**). To a solution of diisopropyl-L-(+)-tartrate (5.0 g, 21.4 mmol) in anhydrous ether (100 mL) at 4 °C, H_5IO_6 (4.9 g, 21.4 mmol, 1 eq) was added under N_2 atmosphere. After stirring at 4 °C for 2 h, the reaction mixture was filtered through Celite® and the filter cake washed with ether (3 x 100 mL). Finally, the solvent

was removed under vacuum affording the title compound (**1i**) as a yellow oil (5 g, 88%). The compound was used without any further purification. ^{1}H NMR (400 MHz, CD_3OD) δ 5.06 – 4.98 (m, 1H), 1.26 (d, J = 2.0 Hz, 3H), 1.25 (d, J = 2.0 Hz, 3H). ^{13}C NMR (101 MHz, CD_3OD) δ 168.6, 93.5, 68.9, 20.4.

***tert*-Butyl 2-oxoacetate** (**1j**). Compound **1j** was obtained as a white solid (1 g, 99%) following the procedure described for **1i** starting from di-*tert*-butyl-L-(+)-tartrate (1.0 g 3.8 mmol). ^{1}H NMR (400 MHz, CD_3OD) δ 4.70 (s, 1H), 4.34 (s, 1H), 3.29 (s, 1H), 1.47 (d, J = 3.7 Hz, 9H). ^{13}C NMR (101 MHz, CD_3OD) δ 170.8, 168.3, 93.7, 47.98, 81.8, 72.6, 26.8.

3-(Benzyloxy)propanal (**1m**). The title compound **1m** (colorless oil, 1.5 g, 99%) was prepared following the procedure described for **1c** starting from 3-(benzyloxy)propan-1-ol (1.5 g, 9.0 mmol). The NMR data agreed with that reported in the literature.[8] ^{1}H NMR (400 MHz, $CDCl_3$) δ 9.79 (t, J = 2x1.9 Hz, 1H), 7.36 – 7.24 (m, 5H), 4.52 (s, 2H), 3.80 (t, J = 2x6.1 Hz, 2H), 2.69 (td, J = 2x6.1, 1.9 Hz, 2H). ^{13}C NMR (101 MHz, $CDCl_3$) δ 201.1, 137.8, 128.4, 127.8, 127.7, 73.2, 63.8, 43.9.

***N*-(2-Oxoethyl)-2-phenylacetamide** (**1n**).

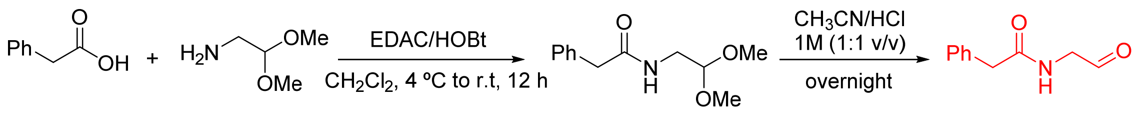

To a solution of 2-phenylacetic acid **S3** (10.0 g, 73.5 mmol) in anhydrous CH_2Cl_2 (200 mL) at 4 °C, HOBt (11.9 g 88.4 mmol, 1.2 eq) was added, followed by EDAC (16.9 g, 88.2 mmol, 1.2 eq), and 2,2-dimethoxyethanamine **S4** (9.3 g, 88.1 mmol, 1.2 eq). After stirring for 12 h from 4 °C to rt under N_2 atmosphere, the solvent was removed under vacuum. The residue was re-suspended in EtOAc (400 mL), washed with citric acid 5% (3 x 200 mL), $NaHCO_3$ 5% (3 x 200 mL), brine (3 x 200 mL) and dried over anhydrous $MgSO_4$ and concentrated under vacuum yielding *N*-(2,2-dimethoxyethyl)-2-phenylacetamide **S5** (11.5 g, 70%). ^{1}H NMR (400 MHz, $CDCl_3$) δ 7.36 – 7.21 (m, 5H), 4.29 (t, J = 2x5.4 Hz, 1H), 3.56 (s, 2H), 3.34 (t, J = 2x5.6 Hz, 2H), 3.31 (s, 6H). ^{13}C NMR (101 MHz, $CDCl_3$) δ 171.0, 134.8, 129.3, 128.9, 127.3, 102.6, 54.5, 43.7, 41.1. A solution of **S5** (3.0 g, 13.4 mmol) was dissolved in CH_3CN/HCl 1 M 1:1 (v/v) (150 mL) and the

mixture was stirred at rt overnight. After that, the solvent was evaporated under vacuum until the remaining aqueous phase. Then, solid NaCl was added until saturation, and extracted with CH_2Cl_2 (3 x 100 mL). The organic phases were combined, dried over anhydrous $MgSO_4$ and concentrated under vacuum to afford the title compound **1n** as solid (2.1 g, 87%). The NMR data agreed with that reported in the literature.[9] ^{1}H NMR (400 MHz, $CDCl_3$) δ 9.59 (s, 1H), 7.39 – 7.22 (m, 5H), 4.14 (d, *J* = 5.0 Hz, 2H), 3.62 (s, 2H). ^{13}C NMR (101 MHz, $CDCl_3$) δ 196.0, 171.3, 134.2, 129.4, 129.1, 127.5, 50.3, 43.4.

L-Alanine methyl ester hydrochloride (**S6**). To a solution of L-Ala (6.5 g, 73.0 mmol) in anhydrous MeOH (150 mL) cooled at – 80 °C, $SOCl_2$ (26.0 g, 218.9 mmol, 3 eq) was slowly added under N_2 atmosphere. After the addition, the mixture was stirred overnight allowed to warm up to rt. After that, the solvent was evaporated under vacuum and the residue was dissolved again with MeOH and removed under vacuum. This operation was repeated five times (5 x 100 mL). Finally, the excess of volatile acid was eliminated *in vacuo* over NaOH pellets.[10] L-Alanine methyl ester hydrochloride (10.4 g, 99%) was obtained as a white solid. ^{1}H NMR (400 MHz, D_2O) δ 4.06 (q, *J* = 3x7.2 Hz, 1H), 3.70 (d, *J* = 1.0 Hz, 3H), 1.41 (dd, *J* = 7.3, 1.0 Hz, 3H). ^{13}C NMR (101 MHz, D_2O) δ 171.2, 53.5, 48.7, 15.0.

(*S*)-*N*-(1-Oxopropan-2-yl)-2-phenylacetamide (**1o**).

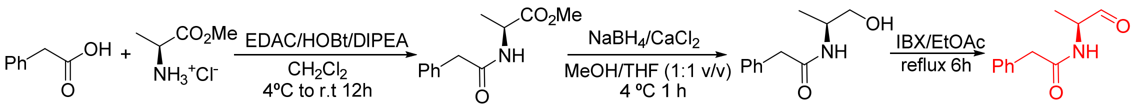

To a solution of 2-phenylacetic acid **S3** (8.3 g, 61.0 mmol, 1 eq) in anhydrous CH_2Cl_2 (200 mL) at 4 °C, HOBt (10.0 g 73.2 mmol, 1.2 eq) was added, followed by consecutive the addition of EDAC (10.2 g, 73.2 mmol, 1.2 eq), L-alanine methyl ester hydrochloride **S6** (10.2 g, 73.2 mmol, 1.2 eq) and DIPEA (11.8 g, 91.4 mmol, 1.5 eq). After stirring for 12 h from 4°C to rt under N_2 atmosphere, the reaction mixture was transferred to a separation funnel and the organic phase was washed with HCl 1 M (3 x 200 mL), $NaHCO_3$ 5% (3 x 200 mL) and brine (3 x 200 mL). Then, the organic phase was dried over anhydrous $MgSO_4$ and concentrated under vacuum yielding *N*-phenylacetyl-L-alanine methyl ester **S7** (12.6 g, 93%) as a white solid. ^{1}H NMR (400 MHz, $CDCl_3$) δ 7.39 – 7.22 (m, 5H), 4.56 (p, *J* = 2x7.2, 2x7.1Hz, 1H), 3.69 (s, 3H), 3.57 (s, 2H), 1.32 (d, *J* = 7.1 Hz,

3H). ^{13}C NMR (101 MHz, $CDCl_3$) δ 173.3, 170.3, 134.4, 127.4, 129.4, 129.0, 127.4, 52.4, 48.1, 43.6, 18.3. To a solution of **S7** (6.0 g, 27.1 mmol) in anhydrous MeOH/THF mixture (100 mL 1:1 v/v), $CaCl_2$ (15.1 g, 135.6 mmol, 5 eq) and $NaBH_4$ (5.1 g, 135.6 mmol, 5 eq) were added and the mixture was stirred for 1h at 0 °C under N_2 atmosphere. Then, HCl 1 M (300 mL) was added at 4°C and stirred until no gas formation was detected. The organic solvent was removed under vacuum, checking that the pH of the aqueous phase was acidic. The product was extracted with CH_2Cl_2 (3 x 100 mL) and the combined organic phases dried over anhydrous $MgSO_4$ and concentrated under vacuum to afford *N*-phenylacetyl-L-alaninol **S8** as a white solid (4.2 g, 81%). ^{1}H NMR (400 MHz, $CDCl_3$) δ 7.38 – 7.20 (m, 5H), 4.02 (ddt, *J* = 10.5, 6.8, 2x3.6 Hz, 1H), 3.60 (dd, *J* = 10.9, 3.4 Hz, 2H), 3.56 (s, 2H), 3.46 (dd, *J* = 11.0, 6.1 Hz, 2H), 1.07 (d, *J* = 6.8 Hz, 3H). ^{13}C NMR (101 MHz, $CDCl_3$) δ 171.9, 134.7, 129.3, 129.0, 127.4, 67.2, 48.1, 43.7, 16.8. *N*-phenylacetyl-L-alaninol **S8** (2.0 g, 10.4 mmol) was dissolved in EtOAc and oxidized with IBX (5.8 g, 20.7 mmol) using an identical procedure to that described for **1c**, affording the title compound **1o** as a white solid (1.5 g, 76%). ^{1}H NMR (400 MHz, $CDCl_3$) δ 9.48 (s, 1H), 7.39 – 7.21 (m, 5H), 4.47 (p, *J* = 4x7.4 Hz, 1H), 3.60 (s, 2H), 1.29 (d, *J* = 7.4 Hz, 3H). ^{13}C NMR (101 MHz, $CDCl_3$) δ 198.7, 170.9, 134.4, 129.3, 129.1, 127.5, 54.4, 43.4, 14.4.

***N*-Cbz-2-Aminoethanal** (**1p**). The title compound **1p** (oil, 1.8 g, 92%) was prepared following the procedure described for **1c** starting from *N*-Cbz-2-aminoethanol (2.0 g, 10.2 mmol). The spectral properties of this product matched those reported in the literature.[11] ^{1}H NMR (400 MHz, $CDCl_3$) δ 9.64 (s, 1H), 7.40 – 7.26 (m, 5H), 5.12 (s, 2H), 4.14 (d, *J* = 5.1 Hz, 2H). ^{13}C NMR (101 MHz, $CDCl_3$) δ 196.3, 156.2, 136.1, 128.6, 128.3, 128.1, 67.2, 51.7.

***tert*-Butyloxycarbonyl-2-aminoethanal** (**1r**). To a solution of 2-aminoethanol (3.0 g, 49.1 mmol) in anhydrous CH_2Cl_2 (50 mL), di-*tert*-butyldicarbonate (Boc_2O) (10.7 g, 49.1 mmol, 1eq) was added under N_2 atmosphere. After stirring at rt overnight the solvent was removed under vacuum. The residue was re-suspended in EtOAc (300 mL) and washed with citric acid 5% wt/v (3 x 100 mL), $NaHCO_3$ 5% wt/v (3 x 100 mL) and brine (3 x 100 mL). The organic phase was dried over anhydrous $MgSO_4$ and concentrated under vacuum yielding *tert*-butyl (2-hydroxyethyl)carbamate as a yellow oil (5 g, 63%). ^{1}H NMR (400 MHz, $CDCl_3$) δ 3.76 – 3.57 (m, 2H), 3.26 (q, *J* = 2x5.4, 5.3 Hz, 2H), 1.42 (s, 9H). ^{13}C NMR (101 MHz, $CDCl_3$) δ 146.7, 79.7, 62.7, 43.2, 28.3. *Tert*-butyl (2-

hydroxyethyl)carbamate (1.5 g, 10.3 mmol) was dissolved in EtOAc and oxidized with IBX (5.8 g, 52.0 mmol) using an identical procedure to that for compound **1c**, affording the title compound **1r** as an oil (1.1 g, 76%). ^{1}H NMR (400 MHz, $CDCl_3$) δ 9.63 (s, 1H), 4.06 (d, *J* = 5.1 Hz, 2H), 1.43 (s, 9H). ^{13}C NMR (101 MHz, $CDCl_3$) δ 197.0, 146.7, 85.2, 28.2. The NMR data matched that reported in the literature.[12]

4-Methyl-*N*-(2-oxoethyl)benzenesulfonamide (1s).

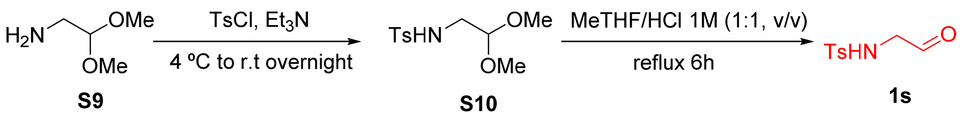

To a solution of 2,2-dimethoxyethanamine (**S9**) (2.0 g, 19.0 mmol) in anhydrous CH_2Cl_2 (100 mL), TsCl (3.6 g 19.0 mmol, 1 eq) and Et_3N (2.9 g, 28.5 mmol, 1.5 eq) were added at 4 °C under N_2 atmosphere and then left to warm up to rt. After stirring for 24 h, the reaction mixture was extracted with HCl 1 M (3 x100 mL). Then, the organic phase was washed with $NaHCO_3$ 5% (3 x 100 mL) and brine (3 x 100 mL), dried over anhydrous $MgSO_4$ and concentrated under vacuum yielding *N*-(2,2-dimethoxyethyl)-4-methylbenzenesulfonamide (**S10**) as an oil (4.7 g, 96%). **S10** (4.7 g, 18.2 mmol) was dissolved in MeTHF/HCl 1 M (200 mL 1:1 v/v). After stirring under reflux for 6 h, the mixture was then allowed to cold down to rt and the organic solvent was removed under vacuum. The residue was re-suspended in EtOAc (300 mL), washed with brine (3 x 100 mL), dried over anhydrous $MgSO_4$, and concentrated under vacuum to afford the compound **1s** as a white solid (3.4 g, 90%). ^{1}H NMR (400 MHz, $CDCl_3$) δ 9.54 (s, 1H), 7.72 (d, *J* = 8.3 Hz, 1H), 7.30 (dt, *J* = 7.9, 2x0.8 Hz, 2H), 3.91 (d, *J* = 5.1 Hz, 2H), 2.41 (s, 3H). ^{13}C NMR (101 MHz, $CDCl_3$) δ 194.7, 144.0, 136.0, 129.9, 127.2, 52.6, 21.5.

General procedure for the aldol addition of sodium pyruvate to aldehydes (1a-s) catalyzed by HBPA

Analytical scale: The reaction (500 µL total volume) was conducted in 1.5 mL Eppendorf tubes. To a solution of the aldehyde (**1a-s**) (dissolved in 50 mM sodium phosphate buffer pH 7.0, 100 mM final concentration in the reaction; in case of partial water solubility, DMF (20% v/v in the reaction was used), a solution of sodium pyruvate (**2**) (25 µL of a 2.0 M aqueous stock solution, pH 6.5-7.0, 100 mM final concentration in the reaction, *(Caution: The solution of sodium pyruvate has to be freshly prepared before use!)* was added.

The reaction was started by adding of HBPA *wild-type* (125 µL of a stock solution 0.029 U mL^{-1}, 4 mg mL^{-1} in 50 mM TEA buffer, 50 mM NaCl, 0.5 mM EDTA and 50% (v/v) of glycerol, 0.007 U mL^{-1}, 1 mg protein mL^{-1} final concentration in the reaction). The reaction mixture was placed in a vortex mixer (1000 rpm) at 25 °C for 24 h. Samples were withdrawn immediately after the enzyme addition (0 h) and after 24 h and analyzed by HPLC as described above.

Synthesis of 4-hydroxyesters (*R*-4a-m)

The aldol additions catalyzed by HBPA, oxidative decarboxylation and the subsequent esterification was carried as follows:

Benzyl (*R*)-3,4-dihydroxybutanoate (*R*-**4a**).

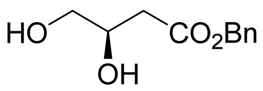

R-***4a***

The synthesis of sodium 4-hydroxy-2-oxoacid **3a** was conducted in 50 mL Erlenmeyer flask. The reaction volume was 20 mL. Sodium pyruvate **2** (1.1 g, 10 mmol, 1 eq, 0.5 M in the reaction) and glycolaldehyde dimer (600 mg, 10 mmol monomer, 1 eq, 0.5 M in the reaction) were dissolved in 15 mL of water. Finally, reaction was started by adding the enzyme (HBPA *wild-type*, 5 mL of a stock solution 0.029 U mL^{-1}, 4 mg mL^{-1} in 50 mM TEA buffer, 50 mM NaCl, 0.5 mM EDTA and 50% (v/v) of glycerol, 0.007 U mL^{-1}, 1 mg protein mL^{-1} final concentration in the reaction). The mixture was placed in an orbital shaker (250 rpm) at 25 °C for 24 h. Reaction was monitored by HPLC and samples were withdrawn immediately after the enzyme addition (0 h) and after 24 h as described above. When the aldol adduct **3a** formed was maximum, the reaction mixture was transferred in a round-bottom flask of 250 mL capacity to avoid spilling of the reaction media due to the huge amount of foam generated when adding the catalase to eliminate the excess of H_2O_2 (see below) and cooled down to 4 °C. To this mixture, H_2O_2 (1.4 mL of a stock solution 8.8 M, 1.2 eq) was added under magnetic stirring. The reaction was monitored by HPLC, and when **3a** was not detected by HPLC, catalase from bovine liver (50 mg) dissolved in 100 mM sodium phosphate buffer, pH 7.0 (1 mL) was added. When the mixture stopped bubbling, it was diluted with methanol (10 volumes), filtered through Celite® and the filter cake washed with methanol (3 x 50 mL). The filtrates were pooled and the solvent was removed under vacuum until dryness. The residue was re-suspended in anhydrous DMF (40 mL) under N_2 atmosphere and CsCl (6.7 mg, 4.0 mmol, 0.4 eq) was added. The

reaction was started by adding BnBr (3.4 g, 20 mmol, 2.0 eq) and stirred at 25 °C for 24 h. Then, EtOAc (200 mL) was added and washed with H_2O (3 x 100 mL) and brine (3 x 100 mL). The organic phase was dried over anhydrous $MgSO_4$, absorbed onto silica gel (100 mL) and loaded in a column chromatography (47x4.5 cm) packed with silica gel (100 g, 35-70 μm, 200-500 mesh). Product was eluted with a step gradient of hexane: EtOAc: 100:0, 200 mL, 80:20, 200 mL, 40:60, 200 mL, 20:80, 200 mL, 0:100, 500 mL. Pure fractions were pooled, and the solvent removed under vacuum affording the title compound *R*-**4a** as a white solid (401 mg, 19 %). $[\alpha]_{20}^{D}$ = – 11.2 (*c* = 1, in MeOH). Chiral HPLC analysis: CHIRALPACK ID, isocratic elution hexane/isopropanol 80/20 (v/v), flow rate 1 mL min^{-1}, tr (*S*) = 12.774 min, tr (*R*) = 11.293 min, 97:3 er. ^1H NMR (400 MHz, D_2O) δ 7.34 – 7.21 (m, 5H), 5.04 (s, 2H), 3.98 (ddt, *J* = 8.7, 6.3, 2x4.4, Hz, 1H), 3.43 (dd, *J* = 11.7, 4.4 Hz, 1H), 3.37 (dd, *J* = 11.7, 6.3 Hz, 1H), 2.51 (dd, *J* = 15.6, 4.4 Hz, 1H), 2.39 (dd, *J* = 15.6, 8.8 Hz, 1H). 13C NMR (101 MHz, D_2O) δ 173.4, 135.5, 128.7, 128.7, 128.5, 128.2, 127.4, 68.4, 67.0, 64.7, 38.2. ESI-TOF m/z: Calcd for [$M+Na^+$] $C_{11}H_{14}O_4Na^+$: 233.0823, found [$M+Na^+$]: 2330814.

Benzyl 3,4-dihydroxybutanoate (*rac*-4a).

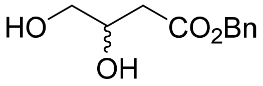

rac-**4a**

The racemic aldol adduct *rac*-**3a** was conducted in an Erlenmeyer flask (50 mL). The reaction volume was 10 mL. Sodium pyruvate **2** (1.1 g, 10 mmol, 1 eq, 1.0 M in the reaction) and glycolaldehyde dimer (0.6 g, 10 mmol monomer, 1 eq, 1.0 M in the reaction) were dissolved in 4.9 mL of water and $NiCl_2$ (100 μL of a 0.1 M stock solution, 1 mM final concentration in the reaction) was added. Finally, reaction was started by adding the enzyme (MBP-YfaU, 55.6 mg of lyophilized powder, dissolved in water (4.9 mL), 2 mg protein mL^{-1} final concentration in the reaction). The mixture was placed in an orbital shaker (250 rpm) at 25 °C for 24 h. The aldol reaction was analyzed as describe above. The subsequent oxidative decarboxylation, esterification, and purification were performed as described for *R*-**4a**, affording the compound *rac*-**4a** (579 mg, 28%). NMR spectra is indistinguishable from that of *R*-**4a**.

Naphthalen-2-ylmethyl (*R*)-3,4-dihydroxybutanoate (*R*-**4a'**).

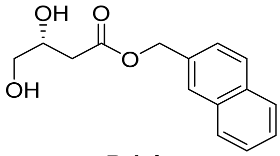

R-**4a'**

Synthesis of **3a** and decarboxylation reaction were performed as described for *R*-**4a**. The residue from the oxidative decarboxylation was suspended in anhydrous DMF (40 mL) under N_2 atmosphere, and CsCl (336.4 mg, 2.0 mmol, 0.4 eq) was added. To this suspension, 2-(bromomethyl)naphthalene (2.2 g, 10 mmol, 2 eq) was added and the reaction was stirred at 25 °C for 24 h. Work up and purification were carried out as described for *R*-**4a**. Compound *R*-**4a'** was obtained as a white solid (650 mg, 50%). $[\alpha]_{20}^{D}$ = + 10.6 (*c* = 1, in MeOH). Chiral HPLC analysis: CHIRALPACK ID, isocratic elution hexane/isopropanol 80/20 (v/v), flow rate 1 mL min^{-1}, tr (*S*) = 17.145 min, tr (*R*) = 14.157 min, 98:2 er. ^{1}H NMR (400 MHz, $CDCl_3$) δ 7.84 (d, *J* = 3.9 Hz, 1H), 7.83 – 7.80 (m, 3H), 7.51 – 7.45 (m, 2H), 7.44 (dd, *J* = 8.5, 1.7 Hz, 1H), 5.31 (s, 2H), 4.15 (ddt, *J* = 8.0, 6.1, 3.8 Hz, 1H), 3.67 (dd, *J* = 11.3, 3.6 Hz, 1H), 3.52 (dd, *J* = 11.3, 6.1 Hz, 1H), 2.63 (dd, *J* = 16.6, 8.6 Hz, 1H), 2.56 (dd, *J* = 16.6, 4.0 Hz, 1H). ^{13}C NMR (101 MHz, $CDCl_3$) δ 172.5, 133.1, 132.8, 128.5, 128.0, 127.7, 127.5, 126.4, 125.8, 68.4, 66.9, 65.7, 37.6. ESI-TOF m/z: Calcd for [2M+Na^+] $C_{30}H_{32}O_8Na^+$: 543.2015, found [2M+Na^+]: 543.1995.

Naphthalen-2-ylmethyl 3,4-dihydroxybutanoate (*rac*-**4a'**).

Synthesis of *rac*-**3a** and decarboxylation reaction were performed as described for *rac*-**4a**. Conditions for esterification and product purification were conducted as described for *R*-**4a'**. The NMR spectra of *rac*-**4a'** (237 mg, 58%) was indistinguishable from that of *R*-**4a'**.

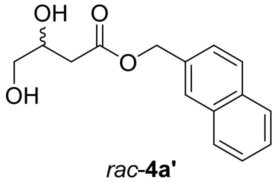

rac-**4a'**

Benzyl (*R*)-4-(benzyloxy)-3-hydroxybutanoate (*R*-**4b**).

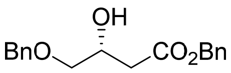

R-**4b**

The synthesis of aldol intermediate **3b** was conducted in an Erlenmeyer (50 mL). Reaction volume was 27 mL. Benzyloxyacetaldehyde (**1b**) (0.4 g, 2.7 mmol, 1 eq, 0.1 M in the reaction) was dissolved in DMF (5.4 mL, 20% (v/v) final concentration in the reaction) and sodium pyruvate **2** (293.1 mg, 2.7 mmol, 1 eq, 0.1 M final concentration in the reaction) dissolved in water (15 mL) was added. Finally, reaction was started by adding the enzyme HBPA *wild-type* (6.6 mL of a stock solution 0.029 U mL^{-1}, 4 mg mL^{-1} in 50 mM TEA buffer, 50 mM NaCl, 0.5 mM EDTA and 50% (v/v) of glycerol, 0.007 U mL^{-1}, 1 mg protein mL^{-1} final concentration in the reaction). The Erlenmeyer was placed in an orbital shaker (250 rpm) at 25 °C for 24h. Reaction was

monitored by HPLC and samples were withdrawn immediately after the enzyme addition (0 h) and after 24 h as described above. The oxidative decarboxylation and subsequent esterification were carried out as described for *R*-**4a**. Product was eluted with a step gradient of hexane: EtOAc: 100:0, 200 mL, 90:10, 200 mL, 80:20, 200 mL, 70:30, 500 mL, 60:40, 500 mL. Pure fractions were pooled and the solvent removed under vacuum affording the title compound *R*-**4b** as a yellow oil (368 mg, 49%). $[\alpha]_{20}^{D}$ = + 8.7 (*c* = 2, in MeOH). Chiral HPLC analysis: CHIRALPACK IB, isocratic elution hexane/isopropanol 80/20 (v/v), flow rate 1 mL min^{-1}, tr (*S*) = 8.292 min, tr (*R*) = 9.317 min, 94:6 er. ^{1}H NMR (400 MHz, $CDCl_3$) δ 7.36 – 7.27 (m, 10H), 5.12 (s, 2H), 4.53 (s, 2H), 4.25 (qd, *J* = 6.1, 4.4 Hz, 1H), 3.50 (dd, *J* = 9.6, 4.5 Hz, 1H), 3.46 (dd, *J* = 9.5, 5.9 Hz, 1H), 2.59 (d, *J* = 6.6 Hz, 2H). ^{13}C NMR (101 MHz, $CDCl_3$) δ 171.9, 137.8, 135.6, 128.6, 128.4, 128.3, 128.2, 127.8, 127.7, 67.2, 66.5, 38.3. ESI-TOF m/z: Calcd for [2M+Na^{+}] $C_{36}H_{40}O_8Na^{+}$: 623.2649, found [2M+Na^{+}]: 623.2621.

Benzyl 4-(benzyloxy)-3-hydroxybutanoate (*rac*-**4b**).

The synthesis of aldol intermediate *rac*-**3b** was conducted in an Erlenmeyer flask (50 mL). The reaction volume was 27 mL. Benzyloxyacetaldehyde (**1b**) (0.4 g, 2.7 mmol, 1 eq, 0.1 M in the reaction) was dissolved in DMF (5.4 mL, 20% (v/v) final concentration in the reaction) and sodium pyruvate **2** (293.1 mg, 2.7 mmol, 1 eq, 0.1 M in the reaction) dissolved in water (10.7 mL) was added. Then, $NiCl_2$ (270 µL of a 1 M stock solution, 1 mM final concentration in the reaction) was added and reaction was started by adding the enzyme (MBP-YfaU 150 mg of lyophilized powder dissolved in water (10.7 mL), 2 mg of protein mL^{-1} final concentration in the reaction). The flask was placed in an orbital shaker (250 rpm) at 25 °C for 24h. Reaction was monitored by HPLC and samples were withdrawn immediately after the enzyme addition (0 h) and after 24 h as described above. The oxidative decarboxylation, subsequent esterification, and purification were conducted as described for *R*-**4b**, affording the compound *rac*-**4b** (283 mg, 41%). NMR spectra was indistinguishable from that of *R*-**4b**.

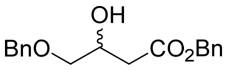

rac-**4b**

2-(4-Bromophenyl)-2-oxoethyl (*R*)-3-hydroxy-4-methoxybutanoate (*R*-**4d**).

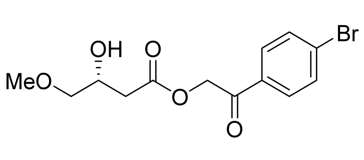

R-**4d**

The precursor **3d** was prepared following the procedure described for *R*-**4a** starting from **1d** (8.4 mL of 0.4 M aqueous solution, 3.4 mmol, 1 eq, 0.1M) and sodium pyruvate **2** (371.4 mg, 3.4 mmol, 1 eq, 0.1 M final concentration in the reaction). The oxidative decarboxylation of **3d** was performed as described for *R*-**4a**. The residue from the oxidative decarboxylation was re-suspended with anhydrous DMF (50 mL) under N_2 atmosphere in a round-bottom flask of 100 mL. To this solution, 2,4′-dibromoacetophenone (938 mg, 3.4 mmol, 1 eq) was added. The reaction mixture was stirred at 4 °C for 2 h. Work up and purification were carried out as described for *R*-**4a**. Product was eluted with a step gradient of hexane:EtOAc: 100:0, 200 mL, 95:5, 200 mL, 90:10, 500 mL 80:20, 500 mL, 70:30, 200 mL, 60:40, 200 mL, 50:50, 800 mL. Pure fractions were pooled, and the solvent removed under vacuum affording compound *R*-**4d** as a white solid (379 mg, 34%). $[\alpha]_{20}^{D}$ = + 0.3 (*c* = 2 in $CHCl_3$). Chiral HPLC analysis: CHIRALPACK IC, isocratic elution hexane/isopropanol 80/20 (v/v), flow rate 1 mL min^{-1}, tr (*S*) = 52.976 min, tr (*R*) = 58.220 min, 71:29 er. ^1H NMR (400 MHz, $CDCl_3$) δ 7.76 (d, *J* = 8.7 Hz, 2H), 7.63 (d, *J* = 8.6 Hz, 2H), 5.39 (d, *J* = 16.4 Hz, 1H), 5.29 (d, *J* = 16.5 Hz, 1H), 4.35 – 4.24 (m, 1H), 3.47 (d, *J* = 1.7 Hz, 1H), 3.45 (d, *J* = 2.7 Hz, 1H), 3.40 (s, 3H), 2.70 (d, *J* = 5.0 Hz, 1H), 2.69 (d, *J* = 7.7 Hz, 1H). ^{13}C NMR (101 MHz, $CDCl_3$) δ 191.4, 171.2, 132.6, 132.3, 129.4, 129.2, 75.4, 67.2, 65.9, 59.2, 38.5. ESI-TOF m/z: Calcd for [M+Na$^+$] $C_{13}H_{30}Br_2O_{10}Na^+$: 527.0103, found [M+Na$^+$]: 527.0106.

2-(4-Bromophenyl)-2-oxoethyl 3-hydroxy-4-methoxybutanoate (*rac*-**4d**).

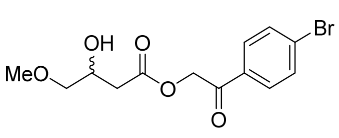

rac-**4d**

The aldol adduct precursor *rac*-**3d** was prepared following the procedure described for *rac*-**4a** (reaction volume was 27 mL) starting from **1d** (6.8 mL of 0.4 M aqueous solution, 2.7 mmol, 1 eq, 0.1M) and sodium pyruvate **2** (297.10 mg, 2.7 mmol, 1 eq). Oxidative decarboxylation, esterification, and purification were carried out as described for *R*-**4d**, affording compound *rac*-**4d** (315 mg, 35%). NMR spectra was indistinguishable from that of *R*-**4d**.

2-(4-Bromophenyl)-2-oxoethyl (*R*)-3-hydroxy-4-phenoxybutanoate (*R*-**4e**).

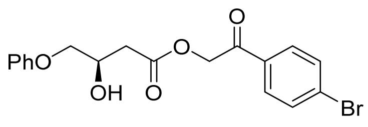

R-**4e**

The precursor **3e** was prepared following the procedure described for *R*-**4b**. Reaction volume was 37 mL. Starting from **1e** (0.5 g, 3.7 mmol, 1 eq, 0.1 M) and sodium pyruvate **2** (0.4 g, 3.7 mmol, 1 eq, 0.1 M). Oxidative decarboxylation of **3e** was conducted as described for *R*-**4a** and the esterification as described for *R*-**4d**. Product was eluted with a step gradient of hexane: EtOAc: 100:0, 200 mL, 95:5, 200 mL, 90:10, 500 mL 80:20, 1 L, 50:50, 1 L. Pure fractions were pooled, and the solvent removed under vacuum affording *R*-**4e** as a white solid (495 mg, 34 %). $[\alpha]_{20}^{D}$ = + 0.3 (*c* = 1, in $CHCl_3$). Chiral HPLC analysis: CHIRALPACK IB, isocratic elution hexane/isopropanol 95/5 (v/v), flow rate 1 mL min^{-1}, tr (*S*) = 89.648 min, tr (*R*) = 83.586 min, 92:8 er. ^{1}H NMR (400 MHz, $CDCl_3$) δ 7.78 (d, *J* = 1.8 Hz, 1H), 7.76 (d, *J* = 1.9 Hz, 1H), 7.64 (d, *J* = 2.0 Hz, 1H), 7.63 (d, *J* = 1.8 Hz, 1H), 7.33 – 7.22 (m, 2H), 6.99 – 6.89 (m, 3H), 5.42 (d, *J* = 16.4 Hz, 1H), 5.32 (d, *J* = 16.5 Hz, 1H), 4.58 – 4.47 (m, 1H), 4.08 (dd, *J* = 9.5, 5.6 Hz, 1H), 4.04 (dd, *J* = 9.5, 5.3 Hz, 1H), 2.90 (dd, *J* = 15.5, 4.2 Hz, 1H), 2.81 (dd, *J* = 15.5, 8.4 Hz, 1H). ^{13}C NMR (101 MHz, $CDCl_3$) δ 191.4, 171.1, 158.4, 132.5, 132.3, 129.5, 129.3, 121.2, 114.6, 70.5, 67.0, 65.9, 38.7. ESI-TOF m/z: Calcd for $[2M+Na^+]$ $C_{36}H_{34}Br_2O_{10}Na^+$: 807.0416, found $[2M+Na^+]$: 807.0412.

2-(4-bromophenyl)-2-oxoethyl 3-hydroxy-4-phenoxybutanoate (*rac*-**4e**).

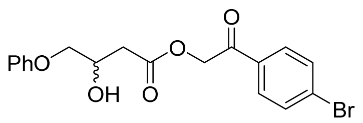

rac-**4e**

The precursor *rac*-**3e** was prepared following the procedure described for *rac*-**4b** (reaction volume was 18 mL) starting from **1d** (0.25 g, 1.8 mmol, 1 eq, 0.1 M) and sodium pyruvate **2** (202.1 mg, 1.8 mmol, 1 eq, 0.1 M). Oxidative decarboxylation, esterification, and purification were carried out as described for *R*-**4d**, affording compound *rac*-**4e** (250 mg, 35%). NMR spectra was indistinguishable from that of *R*-**4e**.

2-(4-bromophenyl)-2-oxoethyl (*R*)-3-hydroxy-4-methyl-4-phenoxypentanoate (*R*-**4f**).

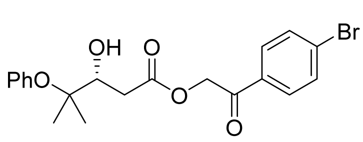

R-**4f**

The precursor **3f** was prepared following the procedure described for *R*-**4b**. Reaction volume was 21 mL, starting from **1f** (0.35 mg, 2.1 mmol, 1 eq, 0.1 M) and sodium pyruvate **2** (234.6 mg, 2.1 mmol, 1 eq, 0.1 M). Oxidative decarboxylation of **3e** was conducted as described for *R*-**4a** and the esterification as

described for *R*-**4d**. Purification by column chromatography: product was eluted with a step gradient of hexane: EtOAc: 100:0, 200 mL, 95:5, 200 mL, 90:10, 500 mL 80:20, 1 L, 50:50, 500 mL. *R*-**4f** was obtained as a solid (260 mg, 29 %). $[\alpha]_{20}^{D}$ = – 1.3 (*c* = 1 in MeOH). Chiral HPLC analysis: CHIRALPACK IB, isocratic elution hexane/isopropanol 90/10 (v/v), flow rate 1 mL min^{-1}, tr (*S*) = 23.895 min, tr (*R*) = 25.530 min, 95:5 er. ^{1}H NMR (400 MHz, $CDCl_3$) δ 7.78 (d, *J* = 1.9 Hz, 1H), 7.77 (d, *J* = 1.9 Hz, 1H), 7.64 (d, *J* = 2.0 Hz, 1H), 7.62 (d, *J* = 1.8 Hz, 1H), 7.30 – 7.21 (m, 2H), 7.08 (t, *J* = 2x7.4 Hz, 1H), 7.00 – 6.96 (m, 2H), 5.43 (d, *J* = 16.4 Hz, 1H), 5.31 (d, *J* = 16.5 Hz, 1H), 4.20 (dd, *J* = 10.2, 2.6 Hz, 1H), 2.99 (dd, *J* = 15.2, 2.6 Hz, 1H), 2.75 (dd, *J* = 15.2, 10.2 Hz, 1H), 1.31 (s, 3H), 1.28 (s, 3H). ^{13}C NMR (101 MHz, $CDCl_3$) δ 191.6, 172.1, 154.4, 132.3, 129.4, 129.3, 129.0, 124.0, 123.8, 81.7, 74.5, 65.9, 37.0, 23.1, 21.8. ESI-TOF m/z: Calcd for [2M+Na$^+$] $C_{40}H_{42}Br_2O_{10}Na^+$: 863.1042, found [2M+Na$^+$]: 863.1037.

2-(4-Bromophenyl)-2-oxoethyl 3-hydroxy-4-methyl-4-phenoxypentanoate (*rac*-**4f**).

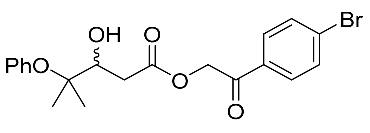

rac-**4f**

The precursor *rac*-**3f** was prepared following the procedure described for *rac*-**4b**. Reaction volume was 18 mL, starting from **1f** (0.3 g, 1.8 mmol, 1 eq, 0.1 M) and sodium pyruvate **2** (202.1 mg, 1.8 mmol, 1 eq, 0.1 M). Oxidative decarboxylation, esterification, and purification were carried out as described for *R*-**4d**, affording compound *rac*-**4f** (169 mg, 22%). NMR spectra was indistinguishable from that of *R*-**4f**.

Benzyl (*R*)-3-hydroxy-4,4-dimethoxybutanoate (*R*-**4g**).

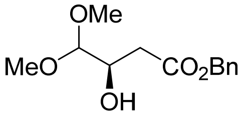

R-**4g**

The precursor **3g** was prepared following the procedure described for *R*-**4a**. Reaction volume was 15 mL starting from **1g** (873 μL of a 6.6 M commercial aqueous solution, 5.8 mmol, 1 eq, 0.1M) and sodium pyruvate **2** (0.63 g, 5.8 mmol, 1 eq, 1 M). Oxidative decarboxylation of **3g** and esterification were carried out as described for *R*-**4a**. Column chromatography purification: *R*-**4g** was eluted with a step gradient of hexane:EtOAc: 100:0, 200 mL, 90:10, 200 mL, 80:20, 200 mL, 70:30, 200 mL, 60:40, 500 mL, 50:50, 500 mL affording *R*-**4g** as a yellow oil (680 mg, 47%). $[\alpha]_{20}^{D}$ = + 1.7 (*c* = 4.2, in H_2O). Chiral HPLC analysis: CHIRALPACK IC, isocratic elution hexane/isopropanol 80/20 (v/v), flow rate 1 mL min^{-1}, tr (*S*) = 14.580 min, tr (*R*) = 17.366 min, 96:4 er. ^{1}H NMR

(400 MHz, $CDCl_3$) δ 7.37 – 7.27 (m, 5H), 5.14 (s, 2H), 4.24 (d, *J* = 5.5 Hz, 1H), 4.13 – 4.05 (m, 1H), 3.43 (s, 3H), 3.40 (s, 3H), 2.67 (dd, *J* = 16.1, 3.9 Hz, 1H), 2.54 (dd, *J* = 16.1, 8.4 Hz, 1H). ^{13}C NMR (101 MHz, $CDCl_3$) δ 171.9, 135.7, 128.5, 128.2, 128.2, 105.8, 68.3, 66.5, 55.7, 55.1, 36.6. ESI-TOF m/z: Calcd for [2M+Na^+] $C_{26}H_{36}O_{10}Na^+$: 531.2211, found [2M+Na^+]: 531.2206.

Benzyl 3-hydroxy-4,4-dimethoxybutanoate (*rac*-**4g**).

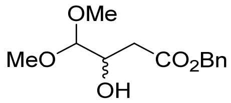

rac-**4g**

The aldol adduct precursor *rac*-**3g** was prepared following the procedure described for *rac*-**4a**. Reaction volume was 58 mL, starting from **1g** (873 μL of a 6.6 M commercial aqueous solution, 5.8 mmol, 1 eq, 0.1 M) and sodium pyruvate **2** (0.63 g, 5.8 mmol, 1 eq, 1 M). Oxidative decarboxylation of *rac*-**3g** and esterification were carried out as described for *R*-**4a**. Purification and the compound characterization were conducted as described for *R*-**4g**, affording *rac*-**4g** (1.0 g, 71%). The NMR spectra was indistinguishable from that of *R*-**4g**.

4-(2-(4-Bromophenyl)-2-oxoethyl)-1-ethyl-(*R*)-2-hydroxysuccinate (*R*-**4h**).

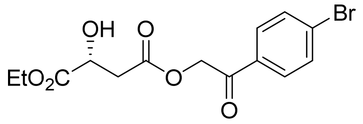

R-**4h**

Precursor **3h** was prepared following the procedure described for *R*-**4b**. Reaction volume was 34 mL, starting from **1h** (700 μL of a 4.9 M commercial aqueous solution, 3.4 mmol, 1 eq, 0.1 M) and sodium pyruvate **2** (0.38 g, 3.4 mmol, 1 eq, 0.1 M). Oxidative decarboxylation of **3h** was conducted as described for *R*-**4a** and the esterification as described for *R*-**4d**. Purification by column chromatography: product was eluted with a step gradient of hexane:EtOAc: 100:0, 200 mL, 95:5, 200 mL, 90:10, 500 mL 80:20, 2 L, 50:50, 700 mL, affording *R*-**4h** as a white solid (434 mg, 35 %). $[\alpha]_{20}^{D}$ = + 1.8 (*c* = 1, in MeOH). Chiral HPLC analysis: CHIRALPACK IB, isocratic elution hexane/isopropanol 80/20 (v/v), flow rate 1 mL min^{-1}, tr (*S*) = 18.187 min, tr (*R*) = 21.975 min, 95:5 er. ^{1}H NMR (400 MHz, $CDCl_3$) δ 7.78 – 7.71 (m, 2H), 7.65 – 7.60 (m, 2H), 5.33 (d, *J* = 0.9 Hz, 2H), 4.55 (dd, *J* = 6.3, 4.3 Hz, 1H), 4.28 (dd, *J* = 7.2, 1.9 Hz, 1H), 4.25 (dd, *J* = 7.1, 1.8 Hz, 1H), 3.05 (dd, *J* = 16.2, 4.3 Hz, 1H), 2.96 (dd, *J* = 16.2, 6.2 Hz, 1H), 1.29 (td, *J* = 2x7.1, 0.5 Hz, 3H). ^{13}C NMR (101 MHz, $CDCl_3$) δ 190.9, 173.0, 169.7, 132.6, 132.3, 129.3, 129.2, 67.3, 66.0, 62.1, 38.7, 14.1. ESI-TOF m/z: Calcd for [2M+Na^+] $C_{28}H_{30}Br_2O_{12}Na^+$: 739.0002, found [2M+$Na^+$]: 739.0008.

4-(2-(4-Bromophenyl)-2-oxoethyl) 1-ethyl -2-hydroxysuccinate (*rac*-**4h**).

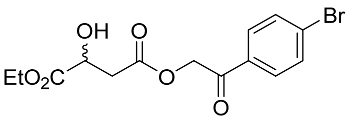

rac-**4h**

The precursor *rac*-**3h** was prepared following the procedure described for *rac*-**4b**. Reaction volume was 25 mL, starting from **1h** (500 μL of a 4.9 M commercial aqueous solution, 2.5 mmol, 1 eq, 0.1M) and sodium pyruvate **2** (0.27 g, 2.5 mmol, 1 eq, 0.1 M). Oxidative decarboxylation and esterification were carried out as described for *R*-**4d**. Purification and the compound characterization were conducted as described above, affording *rac*-**4g** (330 mg, 38%). The NMR spectra was indistinguishable from that of *R*-**4h**.

4-(2-(4-Bromophenyl)-2-oxoethyl) 1-isopropyl (*R*)-2-hydroxysuccinate (*R*-**4i**).

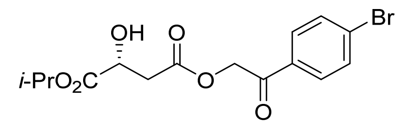

R-**4i**

The precursor **3i** was prepared following the procedure described for *R*-**4a**. Reaction volume was 35 mL starting from **1g** (0.4 g, 3.4 mmol, 1 eq, 0.1 M in the reaction) and sodium pyruvate **2** (0.38 g, 3.4 mmol, 1 eq, 0.1 M). Oxidative decarboxylation of **3i** and esterification were carried out as described for *R*-**4d**. Column chromatography purification: *R*-**4i** was eluted with a step gradient of hexane: EtOAc: 100:0, 200 mL, 95:5, 200 mL, 90:10, 500mL 80:20, 1 L, 70:30, 200 mL, 60:40, 200 mL, 50:50, 1 L. The title compound *R*-**4i** was obtained as a white solid (608 mg, 47%). $[\alpha]_{20}^{D}$ = + 2.2 (*c* = 4, in MeOH). Chiral HPLC analysis: CHIRALPACK IB, isocratic elution hexane/isopropanol 80/20 (v/v), flow rate 1 mL min^{-1}, tr (*S*) = 14.048 min, tr (*R*) = 15.680 min, 91:9 er. ^{1}H NMR (400 MHz, $CDCl_3$) δ 7.77 – 7.73 (m, 2H), 7.65 – 7.60 (m, 2H), 5.32 (d, *J* = 3.4 Hz, 2H), 5.11 (p, *J* = 4x6.3 Hz, 1H), 4.51 (dd, *J* = 6.3, 4.3 Hz, 1H), 3.04 (dd, *J* = 16.2, 4.3 Hz, 1H), 2.94 (dd, *J* = 16.1, 6.2 Hz, 1H), 1.27 (dd, *J* = 6.2, 3.6 Hz, 6H). ^{13}C NMR (101 MHz, $CDCl_3$) δ 190.9, 172.5, 169.7, 132.6, 132.3, 129.3, 129.2, 70.1, 67.3, 66.0, 38.7, 21.6. ESI-TOF m/z: Calcd for [2M+Na^+] $C_{30}H_{34}Br_2O_{12}Na^+$: 767.0315, found [2M+$Na^+$]: 767.0311.

4-(2-(4-bromophenyl)-2-oxoethyl) 1-isopropyl -2-hydroxysuccinate (*rac*-**4i**).

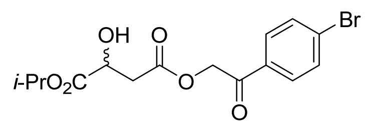

rac-**4i**

The aldol adduct precursor *rac*-**3i** was prepared following the procedure described for *rac*-**4a**. Reaction volume was 22 mL. Starting from **1i** (0.25 g, 2.2 mmol, 1 eq, 0.1 M) and sodium pyruvate **2** (0.24 g, 2.2 mmol, 1 eq, 1 M).

Oxidative decarboxylation of *rac*-**3i** and esterification were carried out as described for *R*-**4d**. Purification and the compound characterization were conducted as described for *R*-**4i**, affording *rac*-**4i** (362 mg, 45%). The NMR spectra was indistinguishable from that of *R*-**4i**.

4-(2-(4-bromophenyl)-2-oxoethyl) 1-(*tert*-butyl) (*R*)-2-hydroxysuccinate (*R*-**4j**).

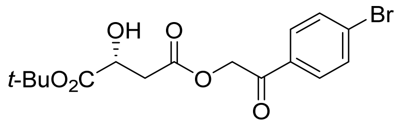

R-**4j**

The precursor **3j** was prepared following the procedure described for *R*-**4b**. Reaction volume was 27 mL. Starting from **1j** (0.35 g, 2.7 mmol, 1 eq, 0.1 M) and sodium pyruvate **2** (0.3 g, 2.7 mmol, 1 eq, 0.1 M). Oxidative decarboxylation of **3j** and esterification were conducted as described for *R*-**4d**. Column chromatography: product was eluted with a step gradient of hexane:EtOAc: 100:0, 200 mL, 95:5, 200 mL, 90:10, 3 L 40:60, 1 L, yielding *R*-**4j** as a white solid (381 mg, 37%). $[\alpha]_{20}^{D}$ = + 0.4 (*c* = 4, in MeOH). Chiral HPLC analysis: CHIRALPACK IB, isocratic elution hexane/isopropanol 80/20 (v/v), flow rate 1 mL min^{-1}, tr (*S*) = 11.661 min, tr (*R*) = 13.056 min, 90:10 er. ^{1}H NMR (400 MHz, $CDCl_3$) δ 7.75 (d, *J* = 8.7 Hz, 2H), 7.62 (d, *J* = 8.7 Hz, 2H), 5.35 (d, *J* = 16.4 Hz, 1H), 5.29 (d, *J* = 16.4 Hz, 1H), 4.42 (q, *J* = 3x5.5 Hz, 1H), 3.00 (dd, *J* = 16.1, 4.4 Hz, 1H), 2.91 (dd, *J* = 16.1, 6.2 Hz, 1H), 1.48 (s, 9H). ^{13}C NMR (101 MHz, $CDCl_3$) δ 190.9, 172.2, 169.7, 132.7, 132.2, 129.2 (d, *J* = 4.5 Hz), 83.1, 67.4, 65.9, 38.8, 27.9. ESI-TOF m/z: Calcd for [2M+Na^+] $C_{32}H_{38}Br_2O_{12}Na^+$: 795.0627, found [2M+$Na^+$]: 795.0621.

4-(2-(4-bromophenyl)-2-oxoethyl) 1-(tert-butyl) -2-hydroxysuccinate (*rac*-**4j**).

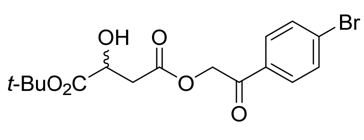

rac-**4j**

The precursor *rac*-**3j** was prepared following the procedure described for *rac*-**4b**. Reaction volume was 23 mL, starting from **1j** (0.3 g, 2.3 mmol, 1 eq, 0.1 M) and sodium pyruvate **2** (0.25 g, 2.3 mmol, 1 eq, 0.1 M). Oxidative decarboxylation and esterification were carried out as described for *R*-**4d**. Purification and the compound characterization were conducted as described above, affording *rac*-**4j** (285 mg, 32%). The NMR spectra were indistinguishable from that of *R*-**4j**.

2-(4-bromophenyl)-2-oxoethyl 3-hydroxy-4-phenylbutanoate (**4l**).

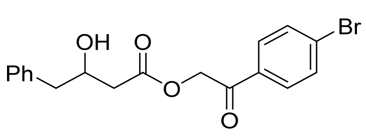

4l

Precursor **3l** was prepared following the procedure described for *R*-**4b**. Reaction volume was 34 mL, starting from **1l** (0.4 mg, 3.3 mmol, 1 eq, 0.1 M) and sodium pyruvate **2** (0.37 mg, 3.3 mmol, 1 eq, 0.1 M). Oxidative decarboxylation of **3l** and esterification were performed as described for *R*-**4d**. Column chromatography: product was eluted with a step gradient of hexane: EtOAc: 100:0, 200 mL, 95:5, 200 mL, 90:10, 500 mL, 80:20, 1 L, 50:50, 1 L, rendering **4l** as a yellow solid (337 mg, 27%). Chiral HPLC analysis: CHIRALPACK IC, isocratic elution hexane/isopropanol 80/20 (v/v), flow rate 1 mL min^{-1}, tr (*S*) = 30.090 min, tr (*R*) = 25.124 min, 50:50 er. ^{1}H NMR (400 MHz, $CDCl_3$) δ 7.78 – 7.72 (m, 2H), 7.65 – 7.60 (m, 2H), 7.35 – 7.20 (m, 5H), 5.39 (d, *J* = 16.4 Hz, 1H), 5.28 (d, *J* = 16.4 Hz, 1H), 4.36 (ddt, *J* = 13.1, 6.9, 2x3.6 Hz, 1H), 2.93 (dd, *J* = 13.6, 7.0 Hz, 1H), 2.83 (dd, *J* = 13.6, 6.3 Hz, 1H), 2.68 (dd, *J* = 15.3, 3.6 Hz, 1H), 2.59 (dd, *J* = 15.3, 8.8 Hz, 1H). ^{13}C NMR (101 MHz, $CDCl_3$) δ 132.3, 129.5, 129.3, 128.5, 126.6, 69.5, 65.8, 42.9, 41.1. Calcd. for [2M+Na^+] $C_{32}H_{36}Br_2O_8Na^+$: 729.0675, found [2M+Na^+]: 729.0679.

2-(4-Bromophenyl)-2-oxoethyl (*R*)-3-hydroxy-4-phenylbutanoate (*rac*-**4l**).

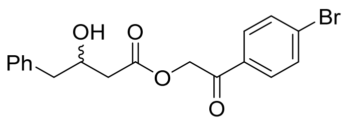

rac-**4l**

The precursor *rac*-**3l** was prepared following the procedure described for *rac*-**4b**. Reaction volume was 25 mL, starting from **1l** (0.3 g, 2.5 mmol, 1 eq, 0.1 M) and sodium pyruvate **2** (0.27 g, 2.5 mmol, 1 eq, 0.1 M). Oxidative decarboxylation and esterification, were carried out as described for *R*-**4d**. Purification and the compound characterization were conducted as described above, affording *rac*-**4l** (339 mg, 37%). The NMR spectra was indistinguishable from that of **4l**.

2-(4-Bromophenyl)-2-oxoethyl (*R*)-5-(benzyloxy)-3-hydroxypentanoate (*R*-**4m**).

The precursor **3m** was prepared following the procedure described for *R*-**4b**. Reaction

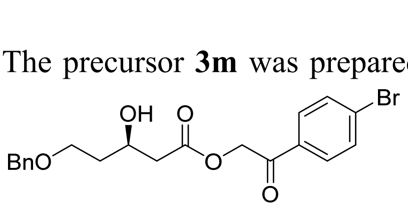

R-**4m**

volume was 30 mL. Starting from **1m** (0.5 g, 3.0 mmol, 1 eq, 0.1 M) and sodium pyruvate **2** (0.34 g, 3.0 mmol, 1 eq, 0.1 M). Oxidative decarboxylation of **3m** and esterification were performed as described for *R*-**4d**. Column chromatography: product was eluted with a step gradient of hexane: EtOAc: 100:0, 200 mL, 95:5, 200 mL, 90:10, 500 mL, 80:20, 1.5 L, 70:30, 200 mL, 50:50, 1 L. *R*-**4m** was obtained as a solid (278 mg,

22 %). $[\alpha]_{20}^{D}$ = – 1.8 (*c* = 1, in MeOH). Chiral HPLC analysis: CHIRALPACK IB, isocratic elution hexane/isopropanol 80/20 (v/v), flow rate 1 mL min^{-1}, tr (*S*) = 16.139 min, tr (*R*) = 17.517 min, 50:50 er. ^{1}H NMR (400 MHz, $CDCl_3$) δ 7.76 (d, *J* = 8.6 Hz, 2H), 7.63 (d, *J* = 8.6 Hz, 2H), 7.36 – 7.23 (m, 5H), 5.39 (d, *J* = 16.4 Hz, 1H), 5.27 (d, *J* = 16.5 Hz, 1H), 4.52 (s, 2H), 4.34 (tt, *J* = 2x7.3, 2x5.1 Hz, 1H), 3.72 (dt, *J* = 9.5, 2x5.9 Hz, 1H), 3.66 (dt, *J* = 9.4, 2x5.8 Hz, 1H), 2.67 (d, *J* = 2.1 Hz, 1H), 2.66 (d, *J* = 4.7 Hz, 1H), 1.88 (d, *J* = 7.1 Hz, 1H), 1.85 (d, *J* = 5.8 Hz, 1H). ^{13}C NMR (101 MHz, $CDCl_3$) δ 191.5, 171.4, 138.0, 132.6, 132.3, 129.4, 129.3, 128.4, 127.7, 73.2, 67.9, 67.1, 65.8, 42.0, 36.0. Calcd for [2M+Na^+] $C_{40}H_{42}Br_2O_{10}Na^+$: 863.1042, found [2M+$Na^+$]: 863.1038.

2-(4-bromophenyl)-2-oxoethyl -5-(benzyloxy)-3-hydroxypentanoate (*rac*-**4m**).

The precursor *rac*-**3m** was prepared following the procedure described for *rac*-**4b**.

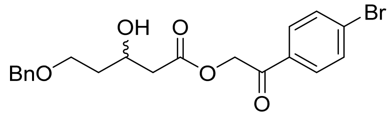

rac-**4m**

Reaction volume was 19 mL, starting from **1m** (0.3 g, 1.8 mmol, 1 eq, 0.1 M) and sodium pyruvate **2** (0.2 g, 1.8 mmol, 1 eq, 0.1 M). Oxidative decarboxylation and esterification were carried out as described for *R*-**4d**. Purification and the compound characterization were conducted as described above, affording *rac*-**4m** (176 mg, 23%). The NMR spectra were indistinguishable from that of *R*-**4m**.

X-Ray structures of *R*-4e, *R*-4f and *R*-4j

Suitable single crystals for X-ray structural analysis of *R*-**4e**, *R*-**4f**, and *R*-**4j** were obtained at room temperature. Compound *R*-**4e** (100 mg) was dissolved in MeOH:CH_2Cl_2 3:1 (v/v) (4 mL), *R*-**4f** (95 mg) in MeOH:CH_2Cl_2 1:2 (v/v) (4 mL) and *R*-**4j** (80 mg) in Et_2O:DMF 9:1 (v/v) (1 mL). Crystals were obtained by evaporation in glass vials (6 mL, 3.5 cm, Ø 1.4 cm) after 72 h at 25 °C. Based on the high enantiomeric excesses observed for the enzyme HBPA X-ray diffraction analysis indicates that HBPA render aldol adducts having *R* configuration as major products (**Figure S7**). Data were collected on a STOE IPDS II two-circle diffractometer with a Genix Microfocus tube with mirror optics using MoKα radiation (λ = 0.71073 Å). The data were scaled using the frame scaling procedure in the X-Area program system (Software X-Area - STOE & Cie GmbH. https://www.stoe.com/product/software-x-area). The structures were solved by direct methods using the program SHELXS and refined against F^2 with full-matrix least-squares techniques using the program SHELXL[13] (**Table S6**). The absolute configuration could

be unequivocally determined for all three structures. The H atoms bonded to O atoms in *R*-**4e** were geometrically allocated and refined with a riding model. In *R*-**4f**, the H atoms bonded to O atoms were freely refined. In *R*-**4j**, the H atom bonded to O was refined with a distance restraint of 0.84 (1) Å for the O-H bond.

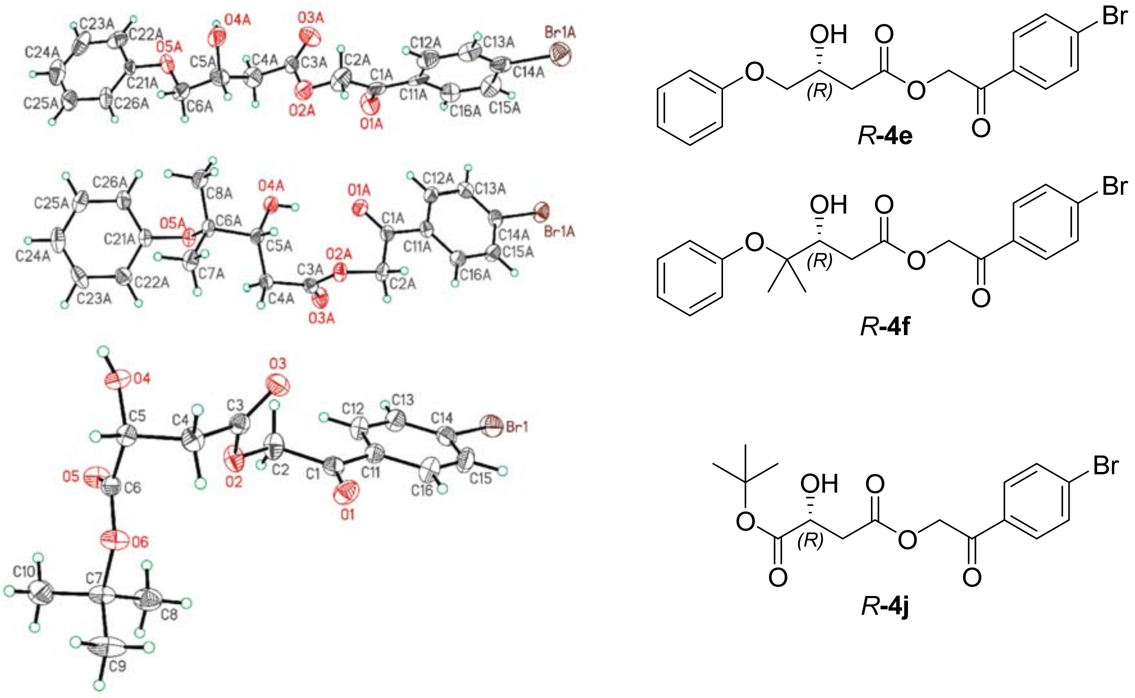

Figure S7. X-Ray structures of *R*-**4e**, *R*-**4f** and *R*-**4j**. ORTEP-type plot displaying one molecule with 50% probability ellipsoids. The data can be obtained free of charge from The Cambridge Crystallographic Data Centre via www.ccdc.cam.ac.uk/data_request/cif

Table S6. X-ray Crystallographic partial data.

Identification code	**4e**	**4f**	**4j**
CCDC number	XXX	XXX	XXX
Empirical formula	$C_{18} H_{17} Br O_5$	$C_{20}H_{21}BrO_5$	$C_{16} H_{19} Br O_6$
Formula weight	393.22	421.28	387.22
Temperature	173(2) K	173(2) K	173(2) K
Wavelength	0.71073 Å	0.71073 Å	0.71073 Å
Crystal system	Orthorhombic	Monoclinic	Orthorhombic
Space group	$P\ 2_1\ 2_1\ 2_1$	$P2_1$	$P\ 2_1\ 2_1\ 2_1$
Unit cell dimensions	a = 5.4464(3) Å, α= 90°.<br>b = 16.6460(7) Å, β= 90°.<br>c = 36.746(2) Å, γ = 90°.	a = 14.6639(7) Å, α= 90°.<br>b = 8.4782(3)Å, β= 108.740(4)°.<br>c = 15.7923(8) Å, γ = 90°.	a = 5.5586(2) Å, α= 90°.<br>b = 12.4492(7) Å, β= 90°.<br>c = 25.3738(11) Å, γ = 90°.
Volume	3331.4(3) $Å^3$	1859.27(15) $Å^3$	1755.87(14) $Å^3$
Z	8	4	4
Density (calculated)	1.568 Mg/m^3	1.505 Mg/m^3	1.465 Mg/m^3
Absorption coefficient	2.493 mm^{-1}	2.239 mm^{-1}	2.367 mm^{-1}
F(000)	1600	864	792
Crystal size	0.130 x 0.030 x 0.030 mm^3	0.230 x 0.210 x 0.160 mm^3	0.180 x 0.110 x 0.080 mm^3
Theta range for data collection	2.064 to 25.027°	3.327 to 27.605°	2.912 to 25.932°
Index ranges	-5<=h<=6, -19<=k<=19, -43<=l<=38	-19<=h<=19, -11<=k<=10, -20<=l<=20	-6<=h<=6, -15<=k<=15, -31<=l<=31
Reflections collected	9721	29057	16242
Independent reflections	5745 [R(int) = 0.0830]	8236 [R(int) = 0.0421]	3411 [R(int) = 0.0294]
Completeness to theta = 25.000°	99.6 %	99.6 %	99.8 %
Absorption correction	Semi-empirical from equivalents	Semi-empirical from equivalents	Semi-empirical from equivalents
Max. and min. transmission	1.000 and 0.657	1.000 and 0.695	1.000 and 0.671
Refinement method	Full-matrix least-squares on F^2	Full-matrix least-squares on F^2	Full-matrix least-squares on F^2
Data / restraints / parameters	5745 / 0 / 433	8236 / 1 / 477	3411 / 1 / 212
Goodness-of-fit on F^2	1.222	1.136	1.264
Final R indices [I>2sigma(I)]	R1 = 0.0915, wR2 = 0.1878	R1 = 0.0552, wR2 = 0.1299	R1 = 0.0389, wR2 = 0.0724
R indices (all data)	R1 = 0.1244, wR2 = 0.2068	R1 = 0.0606, wR2 = 0.1334	R1 = 0.0409, wR2 = 0.0731
Absolute structure parameter	0.028(19)	0.010(11)	-0.008(7)
Largest diff. peak and hole	1.124 and -0.623 e.$Å^{-3}$	0.739 and -0.558 e.$Å^{-3}$	0.423 and -0.341 e.$Å^{-3}$

Enzymatic transamination of 4-hydroxy-2-oxoacids (3). Screening of transaminases from Prozomix, using L-Ala and benzylamine as amino donors.

Using L-Ala (5) as amino donors: the reaction (500 µL total volume) was conducted in Eppendorf tubes (1.5 mL). Sodium salts of 4-hydroxy-2-oxoacids, **3a-b**, **3e**, **3h-g**, were prepared following the procedure described above. A solution (250 µL) of transaminase from Prozomix TA as cell free extracts (T01 to T050) (2-3 mg of lyophilized solid in the reaction dissolved in 50 mM sodium phosphate buffer pH 7.0) containing L-Ala (**5**) (10 eq, 0.5 M final concentration in the reaction) and 1 mM PLP in the reaction was prepared. To this solution, a portion of aldol reaction mixture (250 µL), containing 4-hydroxy-2-oxoacid (**3**) (≈ 100 mM as the basis of calculation) was added. The reaction mixture was placed in a vortex mixer (1000 rpm) at 25 °C for 24 h. Samples were withdrawn immediately after the substrate mix addition (0 h) and after 24 h and analyzed by HPLC as described above. The decrease of the aldol adducts, **3a**, **3g**, and **3h** (**Figures S8**, **S11** and **S12**, respectively) or the formation of the aminated products, **6b** and **6e** (**Figures S9** and **S10)** were indicative of potential positives.

Roughly, four out of the 50 different transaminases were selected as promising candidates for the transamination reaction: T003, T029, T038 and T039 (i.e., T029, T038 and T039 for **3a**; T003, T029, and T39 for **3b**; T039 for **3e**; T029 and T039 for **3g**; and T039 for **3h**) (Figures S8-S12).

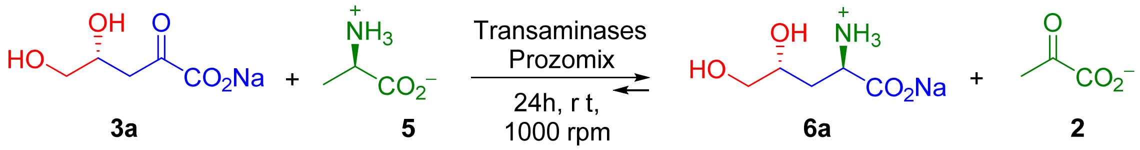

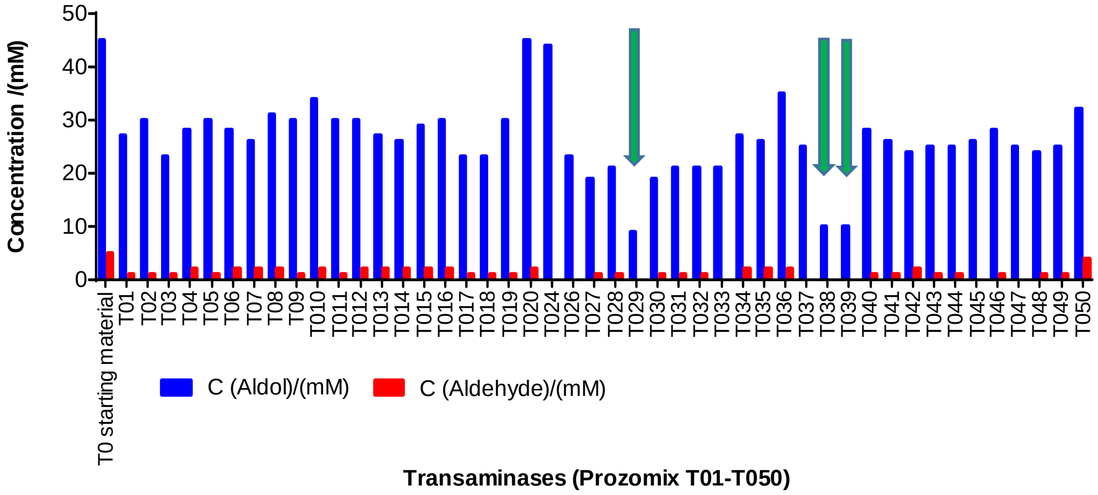

Figure S8. Screening of a panel of transaminases from Prozomix, using L-Ala (**5**) as amine donor to furnish **6a**. Conditions: Crude aldol adduct **3a** containing: **3a** (45 mM), **1a** (5 mM), and HBPA (0.5 mg mL^{-1}); transamination: L-Ala (500 mM), T01-T050 cell free extracts (2 to 3 mg). Green arrows indicate enzymes that decrease the area of aldol adduct or aldehyde.

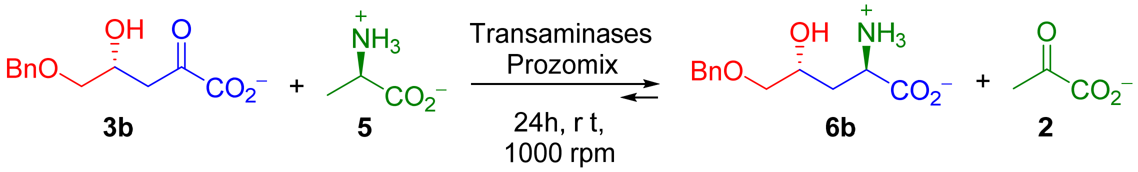

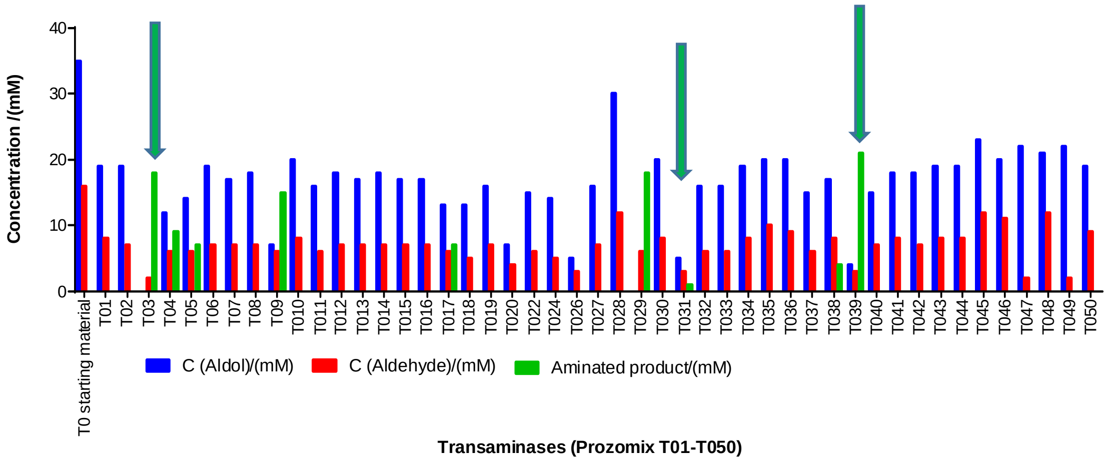

Figure S9. Screening of a panel of transaminases from Prozomix, using L-Ala (**5**) as amine donor to furnish **6b**. Conditions: Crude aldol adduct **3b** containing: **3b** (33 mM), **1b** (17 mM), and HBPA (0.5 mg mL^{-1}); transamination: L-Ala (500 mM), T01-T050 cell free extracts (2 to 3 mg). Green arrows indicate enzymes that decrease the area of aldol adduct or aldehyde.

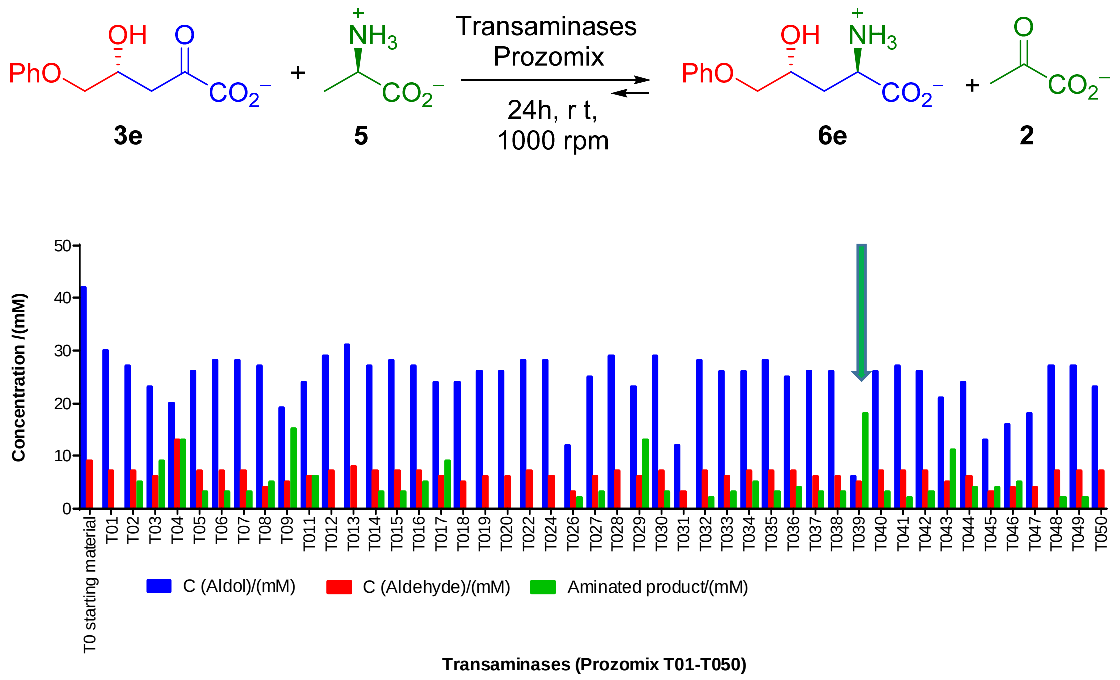

Figure S10. Screening of a panel of transaminases from Prozomix, using L-Ala (**5**) as amine donor to furnish **6e**. Conditions: Crude aldol adduct **3e** containing: **3e** (42 mM), **1e** (8 mM), and HBPA (0.5 mg mL^{-1}); transamination: L-Ala (500 mM), T01-T050 cell free extracts (2 to 3 mg). Green arrows indicate enzymes that decrease the area of aldol adduct or aldehyde.

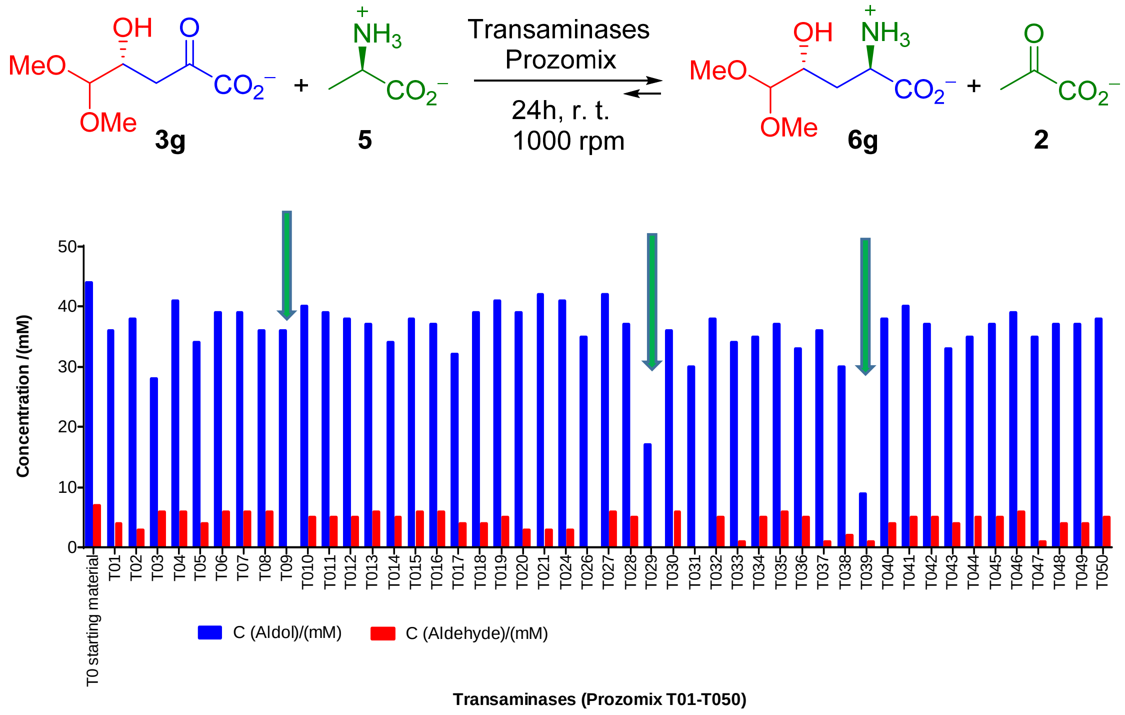

Figure S11. Screening of a panel of transaminases from Prozomix, using L-Ala (**5**) as

amine donor to furnish **6g**. Conditions: Crude aldol adduct **3g** containing: **3g** (43 mM) and **1g** (7 mM), HBPA (0.5 mg mL^{-1}); transamination: L-Ala (500 mM), T01-T050 cell free extracts (2 to 3 mg). Green arrows indicate enzymes that decrease the area of aldol adduct or aldehyde.

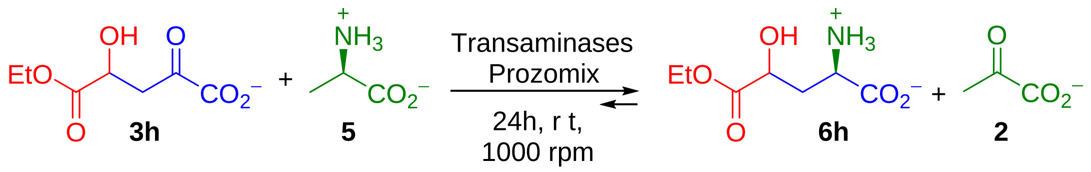

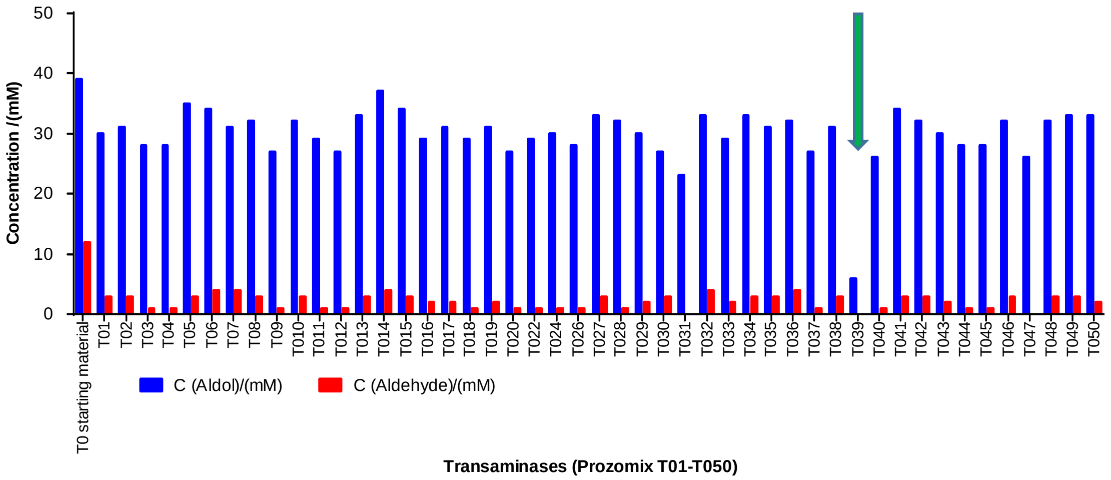

Figure S12. Screening of a panel of transaminases from Prozomix, using L-Ala (**5**) as amine donor to furnish **6h**. Conditions: Crude aldol adduct **3h** containing: **3h** (38 mM) and **1g** (12 mM), HBPA (0.5 mg mL^{-1}); transamination: L-Ala (500 mM), T01-T050 cell free extracts (2 to 3 mg). Green arrows indicate enzymes that decrease the area of aldol adduct or aldehyde.

Control experiments for aldehyde (1) transamination.

To evaluate the degree of conversion for each aldehyde and establish suitable reaction conditions for the one-pot cascade process, we run control experiments incubating aldehydes **1a-b**, **1e**, **1g** and **1h** (100 mM) with the selected transaminases. T039 converted all tested aldehydes with a conversion ranging between 23 and 72%, whereas **1a** and **1b** were not substrates for T003, T029 and T038 (Figures S13-S18). Interestingly, transamination of aldehydes **1** diminished by decreasing the L-Ala concentration to 100 mM (i.e. 2-16% conversion for **1a**, **1g** and **1h**, but still ≈50% for **1b** and **1e**) (Figure S18).

Hence, 100 mM of L-Ala was the concentration of choice to assay this reaction system with the selected substrates.

The reactions (500 µL total volume) were conducted in Eppendorf tubes (1.5 mL). Transaminases lyophilized cell free extracts from Prozomix (2-3 mg) (T029, T031, T038 and T039 for **1a**; T03, T029, T031 and T039 for **1b**; T031 and T039 for **1g**, **1h** and **1e**) were dissolved in 50 mM sodium phosphate buffer pH 7.0 containing L-Ala (250 µL of a 1 M stock solution 500 mM final concentration in the reaction, 10 eq, with 2 mM PLP, 1 mM in the reaction). To this solution, aldehydes (**1a-b**, **1e**, and **1g-h**) (250 µL of a 100 mM stock solution in 50 mM sodium phosphate buffer pH 7.0, 50 mM in each reaction, in case of water insoluble hydrophobic aldehydes DMF (20% in the stock, 10% v/v in the reaction) were added. The reaction mixtures were placed in a vortex mixer (1000 rpm) at 25 °C for 24 h. Samples were withdrawn immediately after the aldehydes addition (0 h) and after 24 h and analyzed by HPLC as described above. Pyruvate formation indicated a positive aldehyde transamination reaction showed that **1a**, **1g** and **1h** were fully converted by T031 and T039 to corresponding amine (**Figures S13**, **S16** and **S17**, respectively). Moreover, the disappearance of aldehydes **1b** and **1e** and the formation of a peak of the amine were indicative of a positive reaction (**Figures S14** and **S15)**.

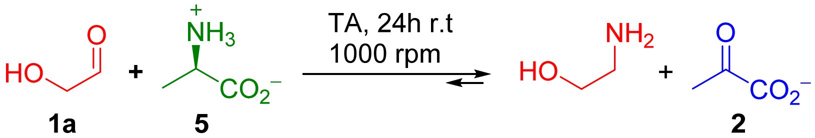

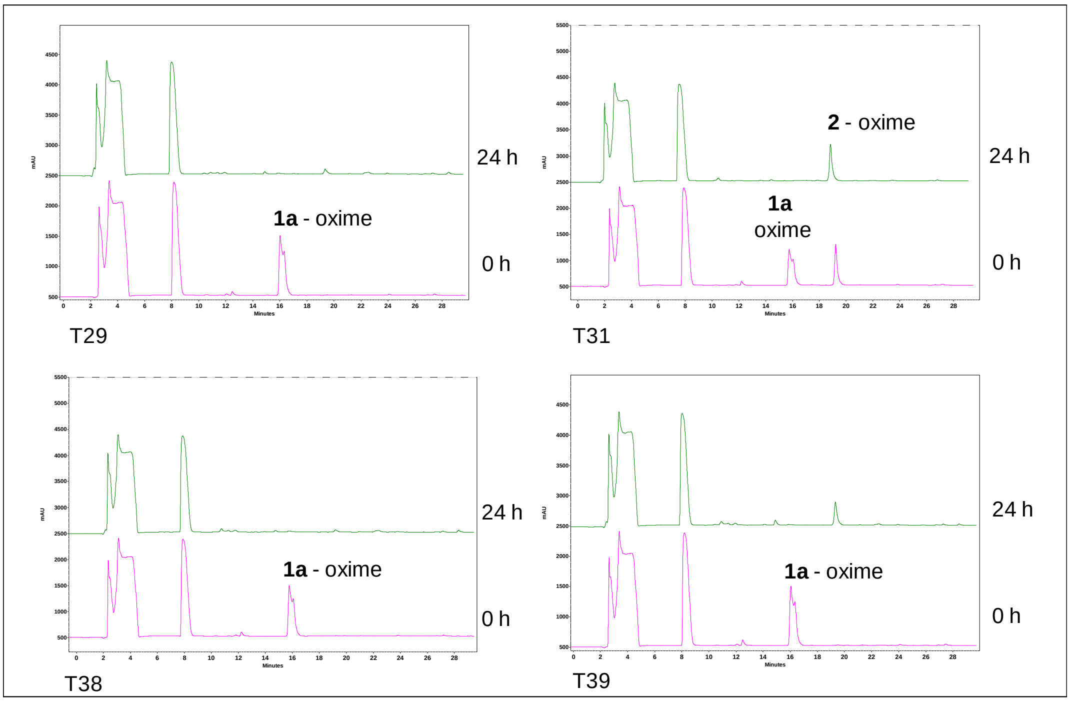

Figure S13. HPLC chromatograms of **1a** transamination catalyzed by T029, T031, T038, and T039 at 0 h and after 24 h of incubation.

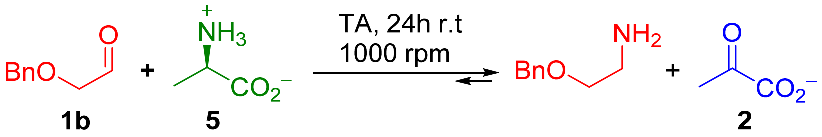

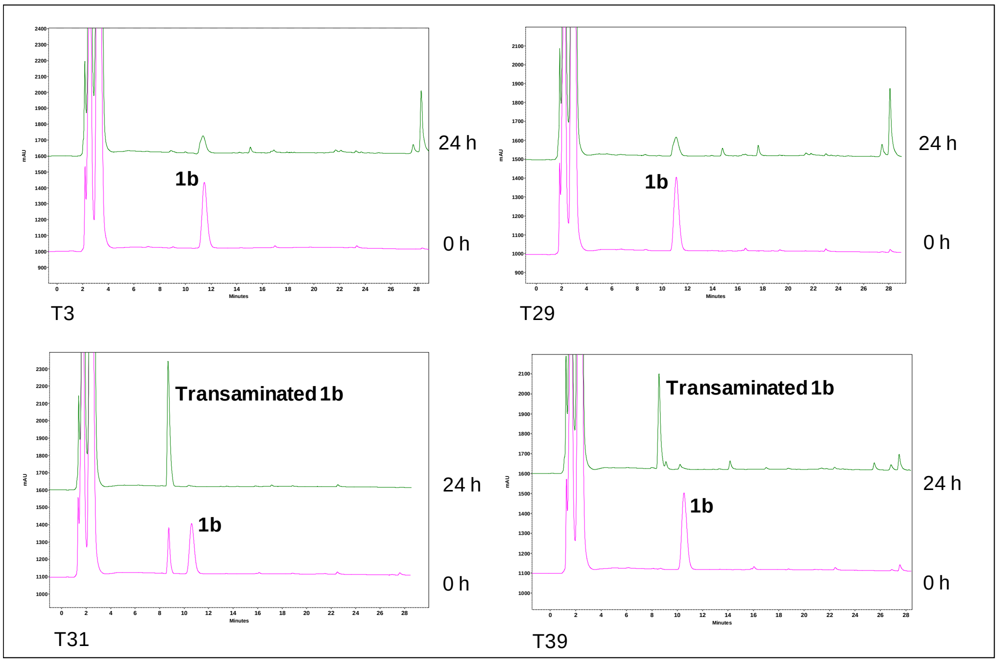

Figure S14. HPLC chromatograms of **1b** transamination catalyzed by T03, T029, T031, and T039 at 0 h and after 24 h of incubation.

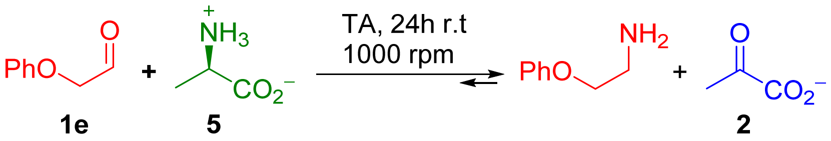

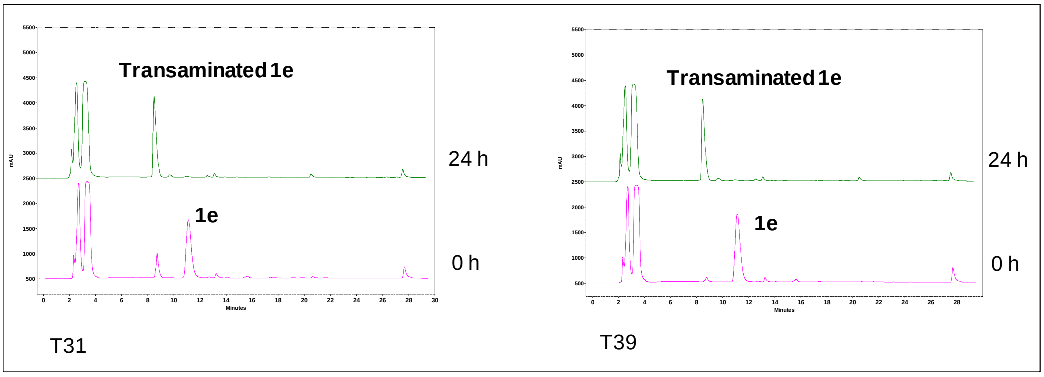

Figure S15. HPLC chromatograms of **1e** transamination catalyzed by T031, and T039 at 0 h and after 24 h of incubation.

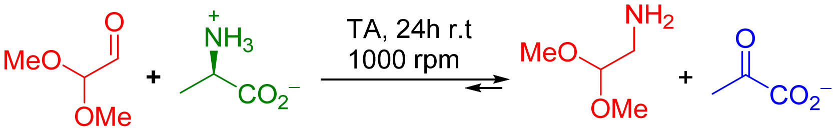

1g **5** **2**

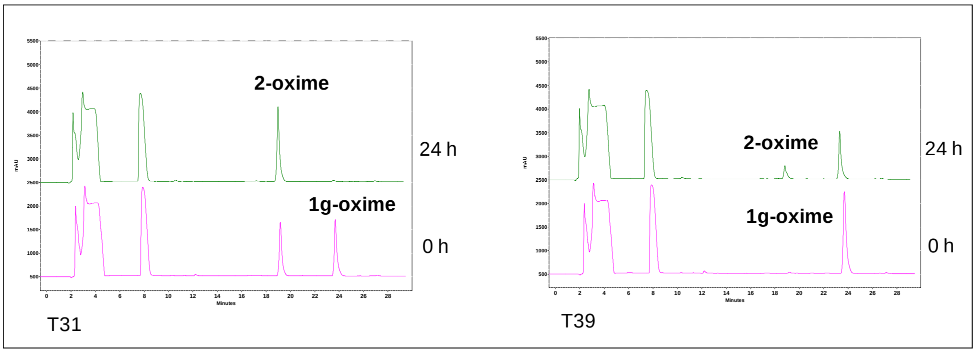

Figure S16. HPLC chromatograms of **1g** transamination catalyzed by T029, T031, and T039 24 h at 0 h and after 24 h of incubation.

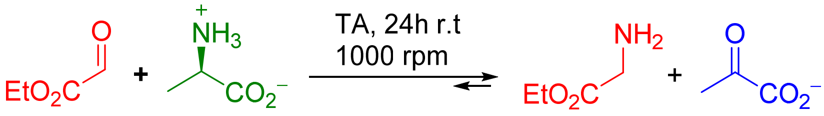

1h **5** **2**

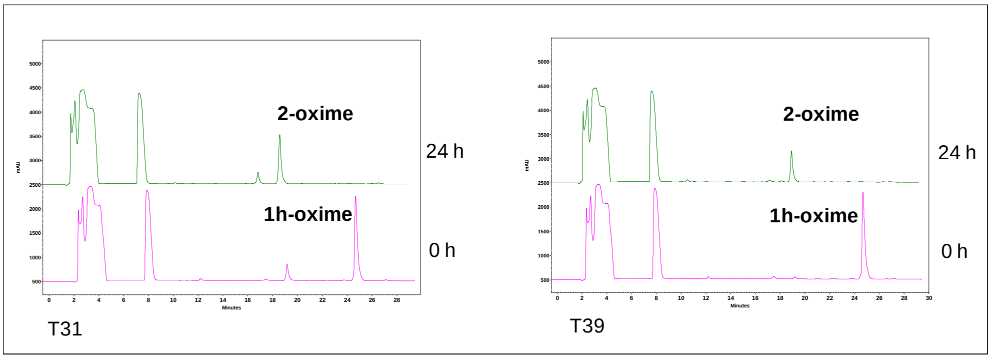

Figure S17. HPLC chromatograms of **1h** transamination catalyzed by T031 and T039 at 0 h and after 24 h of incubation.

Two enzymes (T031 and T039) were selected from this assay and evaluated under equimolar conditions of aldehydes **1a-b**, **1e**, **1h-g** (100 mM) and sodium pyruvate **2** (100 mM). Reactions (500 µL total volume) were conducted in Eppendorf tubes (1.5 mL). Transaminases lyophilized cell free extracts from Prozomix (2-3 mg) (T031 and T039) were dissolved in 50 mM sodium phosphate buffer pH 7.0 containing L-Ala (100 µL of a 0.5 M stock solution 100 mM final concentration in the reaction, 1 eq, with 5 mM PLP,

1 mM in the reaction). To this solution, aldehydes (**1a-b**, **1e**, and **1g-h**) (400 µL of a 125 mM stock solution in 50 mM sodium phosphate buffer pH 7.0, 100 mM final concentration in each reaction, in case of water insoluble hydrophobic aldehydes DMF (20% in the stock, 10% v/v in the reaction) were added. The reaction mixtures were placed in a vortex mixer (1000 rpm) at 25 °C for 24 h. The monitoring and analysis of the reactions was identical as described above. The percentage of transamination of each aldehyde catalyzed by T031 and T039 in the two experimental conditions tested is shown in **Figure S18**.

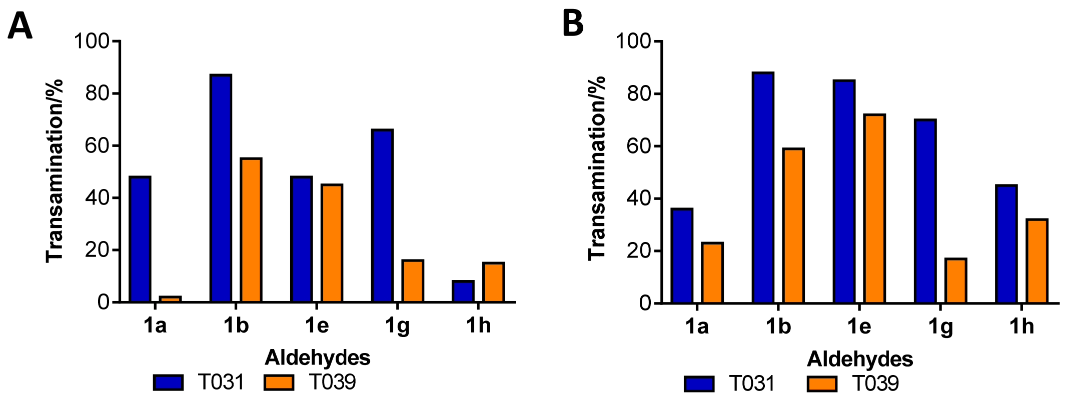

Figure S18. Control experiments for aldehyde transamination catalyzed by T031 and T039 at 24 h of incubation. **A**) Aldehyde (**1**) (100 mM) and L-Ala (**5**) (100 mM); **B**) aldehyde (**1**) (50 mM) and L-Ala (**5**) (500 mM).

Assay of one-pot biocatalytic cascade synthesis of 4-hydroxy-amino with substrate recycling.

General procedure: the reaction (500 µL total volume) was conducted in Eppendorf tubes (1.5 mL). A solution of transaminase Prozomix T039 cell free extract (2 to 3 mg of 0.12 U mg^{-1} lyophilized solid, dissolved in 50 mM sodium phosphate pH 7.0 (250 µL), 0.50 to 0.75 U mL^{-1}, 4 to 6 mg protein mL^{-1} in the reaction), L-Ala (50 µL of a stock solution 1 M in 50 mM sodium phosphate buffer pH 7.0, 100 mM final concentration in the reaction), sodium pyruvate (1.25, 6.25, 12.5, 25.0 y 50.0 µL of a 2 M stock solution in plain water pH 7.0, equivalent to 5, 25, 50, 100 and 200 mM final concentrations in the reaction), PLP (20 µL of a 25 mM stock solution in 50 mM sodium phosphate buffer pH 7.0, 1.0 mM final concentration in the reaction) and aldehydes **1** (≈ 100 mM final concentration in the reaction, as the basis of calculation) was prepared. The necessary

volume of 50 mM phosphate buffer pH 7.0 to complete 500 μL after adding the enzyme was added in each case. The reaction was started by the addition of HBPA *wild-type* (125 μL of a stock solution 0.029 U mL^{-1}, 4 mg mL^{-1} in 50 mM TEA buffer, 50 mM NaCl, 0.5 mM EDTA and 50% (v/v) of glycerol, 0.007 U mL^{-1}, 1 mg protein mL^{-1} final concentration in the reaction). The reaction mixture was placed in a vortex mixer (1000 rpm) at 25 °C for 24 h. Samples were withdrawn immediately after the aldehydes addition (0 h) and after 24 h and analyzed by HPLC as described above (**Figures S19-S21**).

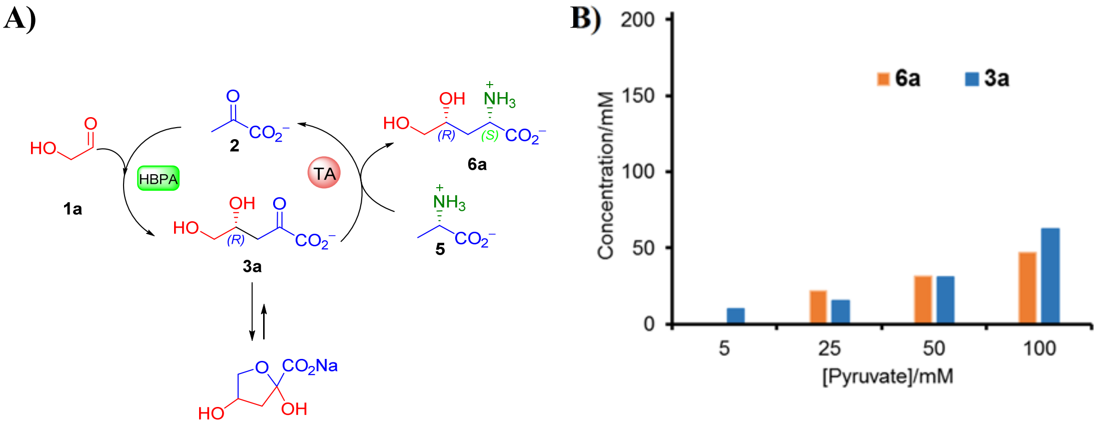

Figure S19. A) One-pot biocatalytic cascade synthesis of **6a** with substrate **2** recycling, starting from aldehyde **1a** (200 mM) and the amine donor **5** (100 mM). B) Concentration of **3a** and **6a** after 24 h of reaction as a function of the initial concentration of pyruvate.

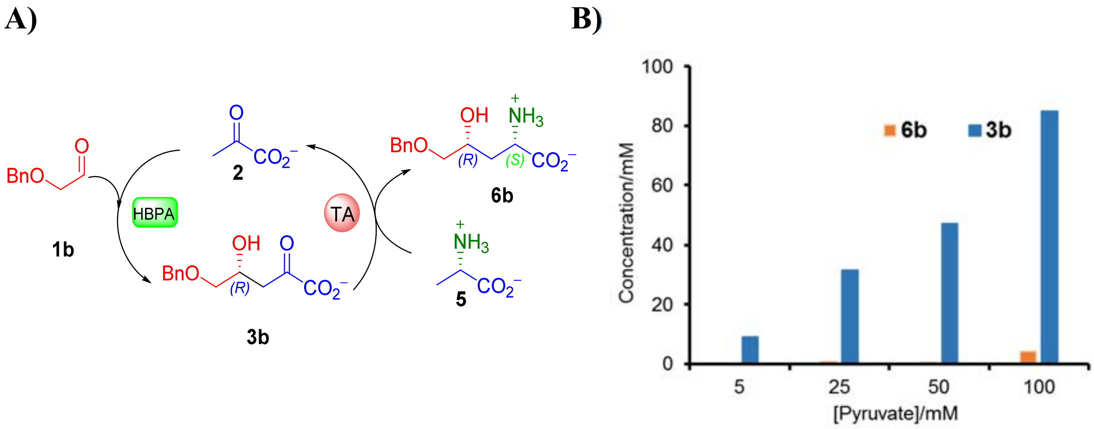

Figure S20. A) One-pot biocatalytic cascade synthesis of **6b** with substrate **2** recycling, starting from aldehyde **1b** (100 mM) and the amine donor **5** (100 mM). B) Concentration of **3b** and **6b** after 24 h of reaction as a function of the initial concentration of pyruvate.

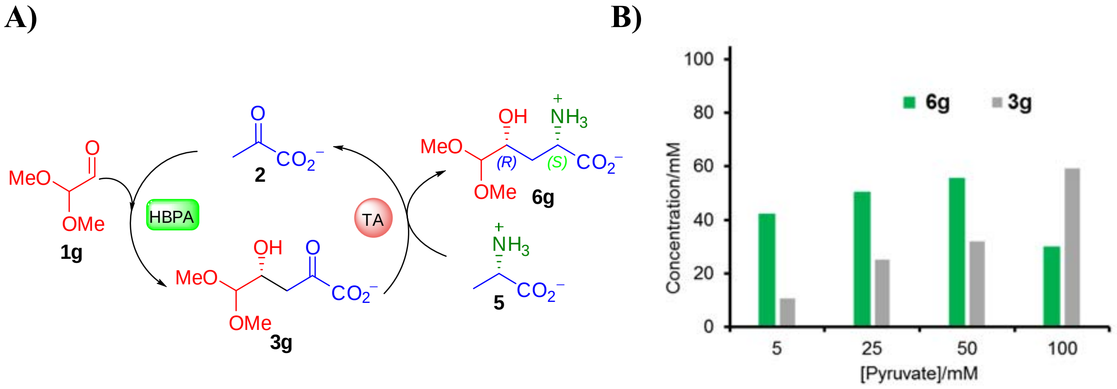

Figure S21. **A**) One-pot biocatalytic cascade synthesis of 4-hydroxy-amino acid derivative **6g** with substrate **2** recycling, starting from aldehyde **1g** (100 mM) and the amine donor **5** (100 mM). **B**) Concentration of **3g** and **6g** after 24 h of reaction as a function of the initial concentration of pyruvate.

Using benzyl amine as amino donors: an extended panel of 194 transaminases from Prozomix Ltd was screened in a one-pot two-step transformation, using benzylamine (**7**) as amine donor and BAL to transform the formed benzaldehyde (**8**) into benzoin (**9**).

To minimize the experimental efforts in the first screening round, we only measured the benzylamine consumption (**7**) (i.e. determined analyzing the benzaldehyde and benzoin (**8**+**9**) formation). This indicated the extension of the transamination reaction, but did not inform about what reaction component is converted, i.e. aldol adducts **3**, unreacted pyruvate **2** or aldehydes **1** or all of them. Moreover, in some instances, benzylamine consumption exceeded the initial amount of aldol adduct **3** in the reaction mixture (~21

µmol) (Figure S22, Table S7). This was because of the transamination of the unreacted aldehyde **1** and pyruvate **2** from the aldol reaction or that were formed by retroaldolysis of **3** (Figure S23). Considering this restriction, 27 hits were identified as potential positive transaminases (Figure S22).

The reaction (500 µL total volume) was conducted in Eppendorf tubes (1.5 mL). 4-Hydroxy-2-oxoacids (**3a-b**, **3e**, **3g-h**) were prepared following the procedure described in section "General procedure for the aldol addition of sodium pyruvate to aldehydes (**1a-s**) catalyzed by HBPA, page S23". Transaminases from Prozomix TA (T001 to T194 set) (2-3 mg of lyophilized solid) were dissolved in a solution (250 µL) containing benzylamine (**7**) (150 mM), PLP (2 mM), ThDP (0.03 mM), $MgSO_4$ (5.0 mM), and BAL (10 U). The reaction was started by the addition of the aldol reaction mixture (250 µL) containing the aldol adduct **3** and unreacted aldehyde **1** and pyruvate **2** (Table S7).

Table S7. Composition (µmol) of the crude aldol reaction mixture (250 µL) used in the screening reaction for the selected **3a**, **3b**, **3e**, **3g**, and **3h** aldol adducts

	**3** (%)	**3** (µmol)	Unreacted **2** (µmol)	Unreacted **1** (µmol)	Expected consumption benzylamine (**7**)[a] (%)	Maximum consumption benzylamine (**7**)[b] (%)
**a**	90	22.5	2.5	2.5	60.0	73.3
**b**	89	22.3	2.8	2.8	59.3	74.0
**e**	80	20.0	5.0	5.0	53.3	80.0
**g**	87	21.8	3.3	3.3	58.0	75.3
**h**	84	21.0	4.0	4.0	56.0	77.3

[a]Only transaminationof aldol adduct **3**. [b]Transamination of aldol adduct **3** plus unreacted pyruvate **2** and aldehyde **1.**

Thus, the final amounts of benzylamine and adducts **3** in the initial reaction mixture were 37.5 µmol and between 20 and 22.5 µmol, respectively (Table S7). The reaction mixture was placed in a vortex mixer (1000 rpm) at 25 °C for 24 h. Samples were withdrawn immediately after the substrate mix addition (0 h) and after 24 h and analyzed by HPLC as described above. Benzylamine (**7**) consumption was analyzed (i.e. measured analyzing the benzaldehyde and benzoin (**8**+**9**) formation). Samples (20 µL) were diluted in methanol (500 µL) and analyzed by HPLC; elution conditions: 55% B over 10 min.

Aldol adduct	T18	T19	T20	T21	T22	T23	T24	T26	T31	T33
3a	10	27	16	40	9	55	54	66	61	35
3b	4	6	17	9	36	17	11	43	59	0
3e	5	5	15	16	18	13	17	34	26	0
3g	16	6	28	20	31	21	32	58	55	48
3h	7	9	0	9	15	0	8	14	11	6

Aldol adduct	T37	T39	T45	T47	T48	T53	T59	T77	T81	T82
3a	0	44	9	58	15	22	0	12	71	0
3b	0	28	0	59	0	0	0	4	37	0
3e	0	33	10	37	0	0	0	0	27	8
3g	54	58	20	48	0	0	14	10	42	6
3h	0	31	0	16	0	0	0	4	14	0

Aldol adduct	T87	T88	T93	T130	T150	T152	T166	T168	T169	T170
3a	23	15	35	69	0	14	90	25	34	33
3b	0	0	15	23	5	0	42	16	40	17
3e	0	0	19	24	8	5	48	14	39	18
3g	0	0	39	8	2	6	43	15	33	17
3h	0	0	12	5	18	6	16	10	16	11

Aldol adduct	T171	T172	T173	T174	T176	T177	T178	T179	T180	T187
3a	0	38	12	53	73	45	23	66	67	23
3b	0	15	0	36	43	12	8	0	17	0
3e	18	0	0	37	40	17	17	7	21	0
3g	6	7	0	40	40	12	18	25	32	20
3h	0	8	0	38	16	0	8	8	14	0

Aldol adduct	T188	T189	T190	T191	T192	T193	T194
3a	78	89	15	100	49	39	94
3b	56	57	9	52	10	0	68
3e	64	65	6	68	14	16	72
3g	47	41	18	43	40	29	48
3h	17	18	9	20	11	10	19

Figure S22. Percentage of benzaldehyde/benzoin formed (i.e., benzylamine consumed) for the screening of 194 transaminases from Prozomix (T001-T194), against aldol adducts **3a-b**, **3e** and **3g-h** using benzylamine (**7**) as amine donor. Conditions: Aldol addition: **1** (100 mM), **2** (100 mM) and HBPA (1 mg mL^{-1}); transamination: **3** (~50 mM) and **7** (75 mM), and T### cell free extract (2-3 mg). The percentage of benzaldehyde/benzoin formed (i.e., benzylamine consumed) after 24 h was determined by HPLC from the peak areas by an external standard method. Transaminases not appearing on the table gave zero-conversion with all substrates. Taking into account the initial amounts of benzylamine and **3** in the reaction mixtures, a percentage >67% (red-backgrounded cells) was considered to come from partial consumption of benzylamine because of the transamination reactions of aldehydes (**1**) and pyruvate (**2**), which remained unreacted from the aldol reaction or were formed by retroaldol during the transamination reaction. The yellow-backgrounded transaminases are those that were selected as potential positive transaminases, i.e. those that showed percentages between 33% and 67% at least for one of the substrates.

Determination of the effect of the retroaldol reaction on the yield of 6a as example.

In some cases of the first screening round, an excess of benzaldehyde/benzoin production (>67%, red spots) was detected. To assess whether HBPA mediated retroaldol was favored by the transamination of the remaining aldehyde, independent experiments were performed using **3a** as example. Transamination assays were carried out with and without previously removing HBPA of the aldol reaction mixture (**Figure S23**).

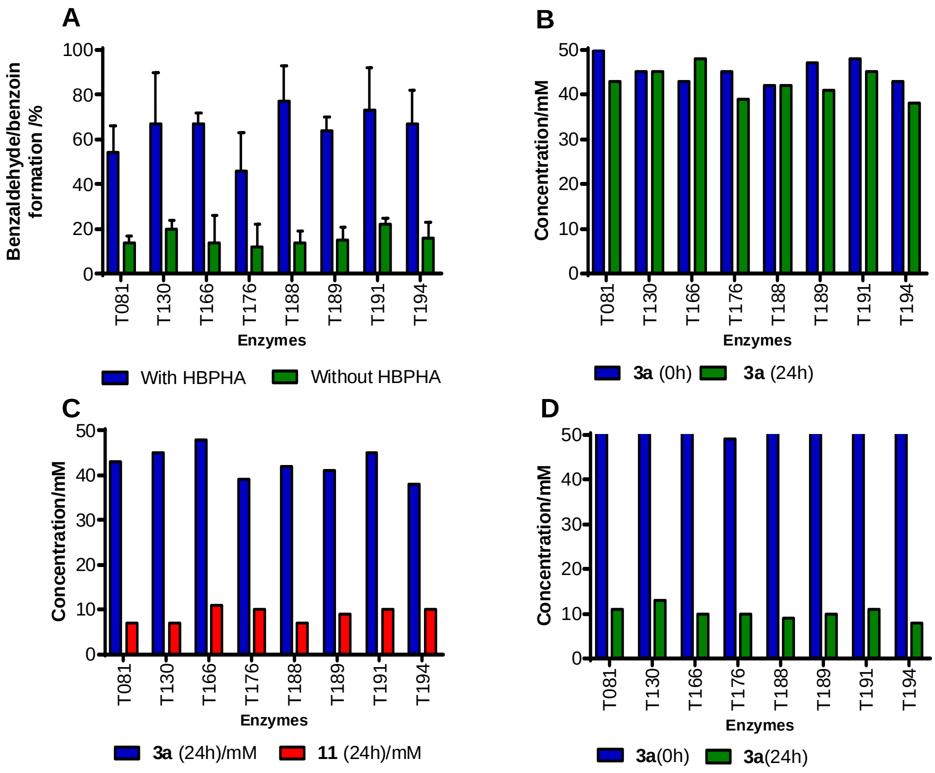

Figure S23. One-pot two-step synthesis of synthesis of **6a**, using benzylamine (**7**) as amine donor. A) Benzaldehyde/benzoin formed in transamination experiments with and without HBPA. B) Decrease of the aldol adduct at 24 h for the transamination system without HBPA. C) Concentration of the aldol adduct (**3a**) and benzaldehyde/benzoin (**11**) at 24 h for the transamination system without HBPA. D) Decrease of the aldol adduct at 24 h for the transamination system with HBPA. Example to demonstrate that the transamination of the aldehyde may activate the retroaldolisis of **3** mediated by HBPA, generating aldehyde and pyruvate and causing the excess of benzoin formation.

Second screening removing the HBPA before running the transamination reaction.

A second screening transamination round was done, removing previously the aldolase from the aldolic mixtures using amicon® Ultra-15 centrifugal filter devices (Nominal Molecular Weight Limit 3.000 Da), 7000 rpm, for 10 minutes at 4 °C. Transamination of **3** was performed following the procedure described above. The percentages of transamination for **3a**, **3g** and **3h** were determined using three measurements: a) from the aldol adducts **3** consumed with precolumn derivatization (10 µL of samples were withdrawn from the reaction mixtures and derivatized with $BnONH_2$ and analyzed by HPLC as described above), b) from the transaminated product formed (**6a**, **6g** and **6h**) previous precolumn derivatization with Bz-OSu as described above, and c) from benzoin produced (**11**) as it was described above. Transamination of **3b** and **3e** were determined

from the aldol adduct **3** consumed and **6b** and **6e** formed, respectively (50 µL of samples were withdrawn from the reaction mixtures and diluted in 500 µL of MeOH and directly analyzed with a gradient elution from 10 to 100% B over 30 min) (**Figure S24**).

Aldol addut	Enzymes																	
3a	T21	T23	T24	T26	T31	T33	T39	T47	T93	T169	T170	T172	T74	T177	T179	T192	T193	
Result	0	0	0	0	0	0	67	0	0	0	0	0	0	0	0	0	0	
3b	T22	T26	T31	T39	T47	T81	T166	T169	T174	176	T188	T189	T191	194				
Result	0	0	0	35	0	0	0	0	0	0	0	0	0	0				
3e	T26	T31	T39	T47	T81	T130	T166	T169	T174	T176	T188	T189	T191					
Result	0	0	55	0	0	0	0	0	0	0	0	0	0					
3g	T24	T26	T31	T33	T37	T39	T47	T81	T93	T166	T169	T174	T176	T188	T189	T191	T192	T194
Result	11	16	12	16	8	63	23	18	15	19	15	18	14	16	18	15	16	16
3h	T39	T174																
Result	57	45																

Figure S24. Second screening round analyzing the percentage of transamination measuring the aldol adduct consumed (**3**), the transaminated product formed (**6**) and benzoin produced (**11**).

Evaluation of the effect of HBPA and BAL in the yield of 6a-b, 6e and 6g-j

4-Hydroxy-2-oxoacids (**3a-b**, **3e**, **3h-g**) were prepared as previously described. Transamination reactions of **3** were performed as above without removing HBPA, using Prozomix T039 transaminase. To evaluate the effect of BAL on the formation of **6**, we run the same two-step reaction sequence without removing HBPA using T039, and without adding BAL in the transamination reaction. Reactions were monitored by HPLC as described for the second screening round.

Assay of one-pot two steps 4-hydroxy-α-amino acids derivatives using a biocatalytic one-pot two-steps approach using the PLP-Dependent branched-chain amino acid aminotransferase (BCATs) from *Escherichia coli.*

Reactions were conducted in Eppendorf tubes (1.5 mL) with 500 µL total reaction volume. To an aldolic reaction mixture containing aldol adduct (**3**) (250 µL, ≈ 100 mM, as the basis of calculation), L-Glu and L-Asp solution mixture (65 µL of stock solution in 50 mM sodium phosphate buffer pH 8.0 containing L-Glu **12** (77 mM), L-Asp **14** (385 mM) and PLP (8 mM)**,** 10 mM, 50 mM and 1 mM final concentration in the reaction respectively), BCAT (28 µL of a stock solution 0.0538 U mL^{-1}, 18 mg mL^{-1} in 50 mM NaH_2PO_4 buffer pH 7.0, NaCl (100 mM), PLP (0.01 mM) and glycerol (50% v/v), 0.003

U mL^{-1}, 1 mg protein mL^{-1} final concentration in the reaction), reaction was started by adding the enzyme (AspAT, 42 μL of a stock solution 2.1615 U mL^{-1}, 12 mg mL^{-1} in 50mM NaH_2PO_4 buffer pH 7.0, NaCl (100 mM), PLP (0.01 mM) and glycerol (50% v/v), 0.18 U mL^{-1}, 1 mg protein mL^{-1} final concentration in the reaction). The necessary volume of 50 mM phosphate buffer pH 7.0 to complete 500 μL was added. The reaction mixture was placed in a vortex mixer (1000 rpm) at 25 °C for 24 h. Samples were withdrawn immediately after the AspAT addition (0 h) and after 24 h and analyzed by HPLC as described above.

Assay of one-pot biocatalytic cascade synthesis of 4-hydroxy-α-amino acid derivatives with substrate recycling BCAT/L-Glu/L-Asp/AspAT.

The reaction (500 μL total volume) was conducted in Eppendorf tubes (1.5 mL). A solution of BCAT and AspAT (28 and 42 μL of stock solution 18 and 12 mg protein mL^{-1} in 50 mM sodium phosphate buffer pH 7.0 containing 100 mM NaCl, 0.1 mM PLP, and 50% (v/v) of glycerol, 0.003 U mL^{-1} of BCAT and 0.18 U mL^{-1} of AspAT. 1 mg protein mL^{-1} of each one in the reaction), containing sodium pyruvate (2.5, 12.5, 22.5, and 25.0 μL, depending on the experiment of a 2 M aqueous stock solution, adjusted to pH 7.0, 10, 50, 90, and 100 mM final concentration in the reaction), L-Asp (45, 25, 5 and 100 μL, depending on the experiment of a 1.0 M stock solution 250 mM sodium phosphate buffer pH 8.0, (90, 50, 10 and 100 mM final concentration in the reaction, respectively), L-Glu (10 μL of a 1.0 M stock solution 250 mM sodium phosphate buffer pH 8.0, containing 25 mM PLP) and aldehyde **1b** (10 μL, 100 mM in the reaction). The reaction was started by adding HBPA (125 μL of a stock solution 0.029 U mL^{-1}, 4 mg mL^{-1} in 50 mM TEA buffer, 50 mM NaCl, 0.5 mM EDTA and 50% (v/v) of glycerol, 0.007 U mL^{-1}, 1 mg protein mL^{-1} final concentration in the reaction). The necessary volume of 50 mM phosphate buffer pH 7.0 to complete 500 μL after adding the enzyme was added in each case. The reaction mixtures were placed in a vortex mixer (1000 rpm) at 25 °C for 24 h. Samples were withdrawn immediately after the HBPA addition (0 h) and after 24 h and analyzed by HPLC as described above (**Figure S25**).

A

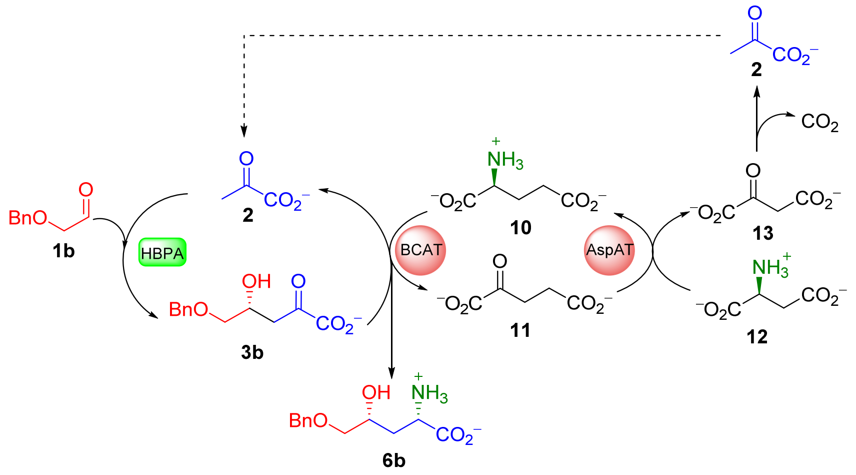

B

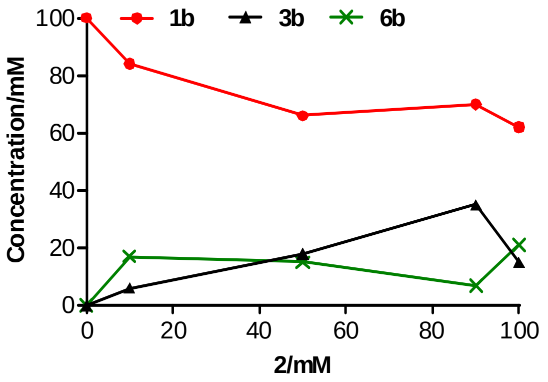

Figure S25. A) One-pot biocatalytic cascade synthesis of 4-hydroxy-α-amino acid derivative **6b** with substrate **2** recycling, starting from aldehyde **1b** (100 mM) and the amine donor **5** (100 mM). B) Concentration of the components after 24 h of reaction as a function of the initial concentration of pyruvate using HBPA/BCAT/AspAT transaminases catalysts.

Synthesis of 4-hydroxy-α-amino acids by tandem HBPA/transaminase and conversion to α-amino-γ-butyrolactone derivatives.

The aldol addition catalyzed by HBPA, transamination reaction, Cbz protection and the subsequent lactonization was carried as follows:

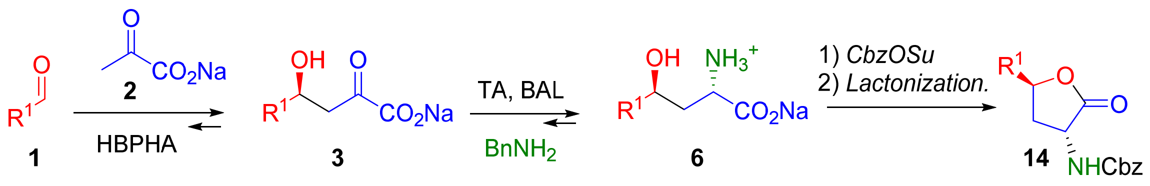

NOTE: **For convenience, the order of the compounds in the SI follows the order in which they were obtained which is different from that of the main text.**

Benzyl ((3*S*,5*R*)-5-(dimethoxymethyl)-2-oxotetrahydrofuran-3-yl)carbamate (14g);

Typical Procedure.

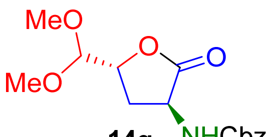

14g

The precursor **3g** was prepared following the procedure described for *R*-**4a**. Reaction volume was 29 mL. Starting from **1g** (437 µL of a 6.6 M commercial aqueous solution, 2.9 mmol, 1 eq, 0.1 M) and sodium pyruvate **2** (0.32 g, 2.9 mmol, 1 eq, 0.1 M). When the aldol adduct **3g** formed was maximum, the transamination reaction was initiated. **Transamination and Cbz protection**: to the mixture of the aldol reaction (2.9 mmol of aldol adduct in 29 mL), benzylamine (**7**) (2.2 mL, of 1 M stock solution in 50 mM sodium phosphate pH 8.0, 4.3 mmol, 1.5 eq), PLP (10.2 mg, 1 mM final concentration in the reaction), ThDP (0.3 mg, 0.015 mM final concentration in the reaction), $MgSO_4$ (24 mg, 2.5 mM final concentration in the reaction), and BAL (136 µL of 2826 U mL^{-1} stock solution in 50 mM buffer TEA containing 50 mM NaCl, 0.5 mM EDTA and 50% (v/v) of glycerol, 5.0 U mL^{-1} final concentration in the reaction) were added. Reaction was started by adding the transaminase (T039, 76 mg of 0.1243 U mg^{-1} lyophilized solid, dissolved in 50 mM sodium phosphate pH 7.0 (5 mL), 0.783 U mL^{-1}, 6 mg protein mL^{-1} final concentration in the reaction). The mixture was placed in an orbital shaker (250 rpm) at 25 °C for 24 h. Formation of **6g** was estimated by measuring the aldol adduct **3g** consumed. For both enzymatic reactions, samples were withdrawn immediately after the enzyme addition (0 h) and after 24 h and analyzed by HPLC using pre-column derivatization as described above. When the consumption of **3g** reached a maximum (24 h), methanol (10 volumes) was added. The mixture was filtered through Celite® and the filter cake washed with methanol (3 x 50 mL). The filtrates were pooled and evaporated under vacuum, until all MeOH was removed. The aqueous residue was diluted with $NaHCO_3$ 20% w/v solution (50 mL). To this solution, Cbz-OSu (1.6 g dissolved in 50 mL 2-MeTHF, 2.2 eq) was added. The reaction was stirred at 25 °C during 12 h. Then, the aqueous phase was first extracted with EtOAc (3 x 50 mL). After that, the pH of aqueous solution was adjusted to 3.5 with 5 M HCl, and extracted with EtOAc (3 x 100 mL). The organic phase was dried over anhydrous $MgSO_4$, and the solvent removed under vacuum

and the residue submitted to intramolecular lactonization using three different procedures as follows:

Procedure 1: 471 mg of **6g** (1.4 mmol, 1eq) from the previous step was suspended in anhydrous DMF (60 mL) under N_2 atmosphere. To this solution, EDAC (414.3 mg, 2.2 mmol, 1.5 eq) and DMAP (5.3 mg, 43.2 µmol, 0.03 eq) were added. The reaction was stirred from 4 °C to rt overnight. Then, EtOAc (300 mL) was added and washed with H_2O (3 x 100 mL), aqueous $NaHCO_3$ 20% w/v (3 x 100 mL) and brine (3 x 100 mL). The organic phase was dried over anhydrous $MgSO_4$, absorbed onto silica gel (100 mL) and loaded on a column (AFORA, 5880/2, 47x4.5) packed with silica gel (100 g, 35-70 µm, 200-500 mesh, Merck). Product was eluted with a step gradient of hexane:EtOAc: 100:0, 500 mL, 90:10, 200 mL, 80:20, 200 mL, 70:30, 200 mL, 60:40, 2 L. Pure fractions were pooled, and the solvent removed under vacuum affording the compound **14g** as a white solid (dr: >95:5, 121 mg, 27%) (**Table S8**). $[\alpha]_{20}^{D} = -20$ (c = 1, in $CHCl_3$). ^1H NMR (400 MHz, $CDCl_3$) δ 7.61 - 6.80 (m, 5H), 5.10 (s, 2H), 4.54 (d, J = 9.3 Hz, 1H), 4.47 (td, J = 2x10.0, 6.8 Hz, 1H), 4.38 (d, J = 2.7 Hz, 1H), 3.47 (s, 3H), 3.46 (s, 3H), 2.77 (dd, J = 13.1, 9.7 Hz, 1H), 2.19 (d, J = 10.9 Hz, 1H). ^{13}C NMR (101 MHz, $CDCl_3$) δ 175.0, 155.7, 136.8, 104.9, 76.7, 67.3, 57.4, 56.3, 50.2, 29.3. ESI-TOF m/z: Calcd for $[M+H^+]$ $C_{15}H_{20}NO_6^+$: 310.1321, found $[M+H^+]$: 310.1291.

Procedure 2: 472 mg of **6g** (1.4 mmol, 1eq) from the previous reaction was suspended in anhydrous DMF (60 mL) under N_2 atmosphere. To this solution, HOBt (292 mg, 2.2 mmol, 1.5 eq) and EDAC (414.3 mg, 2.2 mmol, 1.5 eq) were added. The reaction was stirred overnight from 4 °C to rt. Work up and purification was performed as described for **procedure 1**, affording the compound **14g** (**Table S8**).

Procedure 3: 265 mg of **6g** (810.4 µmol, 1eq) from the previous reaction was suspended in anhydrous CH_2Cl_2 (60 mL) under N_2 atmosphere. To this solution, HOBt (164.3 mg, 1.2 mmol, 1.5 eq), EDAC (233 mg, 1.2 mmol, 1.5 eq) and Et_3N (164 mg, 1.6 mmol, 2 eq) were added. The reaction was stirred overnight from 4 °C to rt. Work up and purification was performed as described for **procedure 1**, affording the compound **14g** (**Table S8**).

Table S8. Intramolecular lactonization conditions.

Procedure	Aldol Conv./(%)[a]	Transamination Conv./(%)[b]	Yield (%)[c]	[α][d]
1	80	98	27	– 20
2	86	90	35	– 21
3	87	94	52	– 20

[a] Percentage of aldol adduct (**3g**) formed after 24h. [b] Percentage of transaminated product (**6g**) formed after 24h. [c] Isolated yield (**14g**). [d]*c*=1 in $CHCl_3$.

Benzyl ((3*S*,5*R*)-5-((benzyloxy)methyl)-2-oxotetrahydrofuran-3-yl)carbamate (14b).

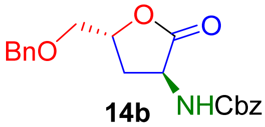

14b

Precursor **3b** was prepared following the procedure described above *R*-**4b**, starting from **1b** (0.5 g, 3.3 mmol, 0.1 M final concentration in the reaction) and sodium pyruvate **2** (0.37 g, 3.3 mmol, 1 eq). The reaction yielded 69% of **3b**. Transamination reaction was conducted in an Erlenmeyer (100 mL) using the BCAT/L-Glu/L-Asp/AspAT system. To the aldol reaction mixture (33 mL), L-Glu (1.2 mL of a 0.5 M stock solution in 250 mM sodium phosphate buffer pH 8.0, 10 mM, containing 25 mM PLP, 1 mM final concentration in the reaction), L-Asp (3 mL of a 1 M stock solution in 250 mM sodium phosphate buffer pH 8.0, 50 mM) were added. The reaction was started by adding BCAT (2.3 mL of a stock solution 0.0538 U mL^{-1}, 18 mg mL^{-1} in 50 mM NaH_2PO_4 buffer pH 7.0, NaCl (100 mM), PLP (0.01 mM), glycerol (50% v/v), 0.003 U mL^{-1}, 1 mg mL^{-1} protein final concentration in the reaction), and AspAT (3.5 mL of a stock solution 2.1615 U mL^{-1}, 12 mg mL^{-1} in 50 mM NaH_2PO_4 buffer pH 7.0, NaCl (100 mM), PLP (0.01 mM) and glycerol (50% v/v), 0.18 U mL^{-1}, 1 mg mL^{-1} protein final concentration in the reaction). The mixture was placed in an orbital shaker (250 rpm) at 25 °C for 24 h. Samples were withdrawn immediately after the enzyme addition (0 h) and after 24 h as described above, reaction was monitored by HPLC. When the reaction was completed (49% product formed), the enzymes were precipitated by adding methanol (10 volumes, 600 mL). The mixture was filtered through Celite® and the filter cake washed with methanol (3 x 50 mL). The filtrates were pooled and the solvent removed under vacuum, until all MeOH was evaporated and only the aqueous solution remained. Cbz protection and conversion to α-amino-γ-butyrolactone derivative **14b** were performed following the **procedure 3**. Product was eluted with a step gradient of hexane:EtOAc: 100:0, 500 mL, 90:10, 200 mL, 80:20, 200 mL, 70:30, 500 mL, 60:40, 1 L. The title compound **14b** was obtained as yellow oil (dr: >95:5, 222 mg, 26%). $[\alpha]_{20}^{D}$ = – 18 (*c* = 1, in $CHCl_3$). ^{1}H NMR (400 MHz,

$CDCl_3$) δ 7.39 – 7.23 (m, 10H), 5.10 (s, 2H), 4.70 (d, *J* = 9.2 Hz, 1H), 4.66 – 4.46 (m, 2H), 4.10 (q, *J* = 3x7.1, Hz, 1H), 3.70 (d, *J* = 8.0 Hz, 1H), 3.54 (d, *J* = 7.9 Hz, 1H), 2.68 (d, *J* = 11.3 Hz, 1H), 2.38 (dd, *J* = 18.1, 9.9 Hz, 1H). ^{13}C NMR (101 MHz, $CDCl_3$) δ 128.5, 76.6, 73.8, 71.1, 70.9, 67.4, 50,4, 31.8. ESI-TOF m/z: Calcd for [$M+H^+$] $C_{20}H_{22}NO_5^+$: 356.1506, found [$M+H^+$]: 356.1498.

(3*S*,5*R*)-5-(Hydroxymethyl)-2-oxotetrahydrofuran-3-aminium chloride (**14a**).

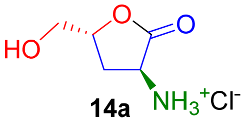

Compound (**14a**) was prepared by dissolving **14b** (50 mg, 140.7 µmol, 1 eq.) in MeOH (15 mL). Then, HCl (156 µL of 1 M stock solution, 1.1 eq) and Pd/C (1.50 mg, 10 mol%, 10% Pd, 50% humidity) were added. The suspension was stirred at room temperature for 2 hours under H_2 atmosphere. The reaction mixture was filtered through Celite® and the pellet was washed with MeOH (3x50 mL). Then, the solvent was removed *in vacuo* obtaining the title compound as a yellow oil (dr >95:5, 14 mg, 59%). (3*S*,5*R*)-5-(Hydroxymethyl)-2-oxotetrahydrofuran-3-aminium chloride, **14a**: ^1H NMR (400 MHz, CD_3OD) δ 4.82 (m, 1H), 4.45 (dd, *J* = 10.7, 9.5 Hz, 1H), 3.86 (dd, *J* = 12.4, 2.4 Hz, 1H), 3.69 (dd, *J* = 12.4, 2.4 Hz, 1H), 2.72 (dd, *J* = 13.0, 9.6 Hz, 1H), 2.47 (ddd, *J* = 13.0, 10.7, 9.1 Hz, 1H). ^{13}C NMR (101 MHz, CD_3OD) δ 172.1, 78.7, 62.7, 47.8, 28.3.

The spectra of the sample changed with the time and it was because of the cyclic lactone is hydrolyzed leading to the acyclic carboxylate compound namely **(2*S*,4*R*)-2-amino-4,5-dihydroxypentanoic acid**: ^1H NMR (400 MHz, CD_3OD) δ 4.23 (dd, *J* = 8.0, 4.8 Hz, 1H), 3.92 (dd, *J* = 5.8, 4.0 Hz, 1H), 3.51 (m, 2H), 2.23 (ddd, *J* = 14.8, 4.9, 3.0 Hz, 1H), 1.89 (ddd, *J* = 14.7, 10.2, 8.0 Hz, 1H). ^{13}C NMR (101 MHz, CD_3OD) δ 169.3, 68.8, 65.3, 50.4, 32.5.

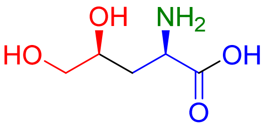

(2*S*,4*R*)-2-amino-4,5-dihydroxypentanoic acid

Benzyl ((3*S*,5*R*)-5-((benzylthio)methyl)-2-oxotetrahydrofuran-3-yl)carbamate (**14c**).

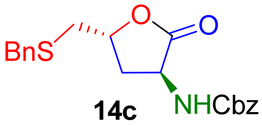

The synthesis of aldol intermediate **3c** was conducted in an Erlenmeyer flask (50 mL). The reaction volume was 15 mL. 2-(Benzylthio)acetaldehyde (**1c**) (0.25 g, 1.5 mmol, 1 eq, 0.1 M final concentration in the reaction) was dissolved in DMF (3 mL, 20% (v/v) in the reaction) and sodium pyruvate **2** (165.5 mg, 1.5 mmol, 1 eq, 0.1 M final concentration in

the reaction) dissolved in water (6.6 mL) was added. Finally, the reaction was started by adding the enzyme HBPA H205A (5.4 mL of a stock solution 0.009 U mL^{-1}, 2.8 mg mL^{-1} in 50 mM TEA buffer, 50 mM NaCl, 0.5 mM EDTA and 50% (v/v) of glycerol, 0.00324 U mL^{-1}, 1 mg mL^{-1} protein final concentration in the reaction). The reaction was placed in an orbital shaker (250 rpm) at 25 °C for 24h. Samples were withdrawn immediately after the enzyme addition (0 h) and after 24 h as described above. The reaction yielded 66% of **3c**. Enzymatic transamination of **3c** using BCAT/L-Glu/L-Asp/AspAT system yielded 66% of **6c**. Cbz protection and conversion to α-amino-γ-butyrolactone derivative **14c** were performed following the **procedure 3**. Product was eluted with a step gradient of hexane:EtOAc: 100:0, 500 mL, 90:10, 200 mL, 80:20, 200 mL, 70:30, 1.5 L, affording the compound **14c** as a solid (dr: >95:5, 124 mg, 22%). $[\alpha]_{20}^{D}$ = – 32 (*c* = 1, in $CHCl_3$). ^{1}H NMR (400 MHz, $CDCl_3$) δ 7.68 – 6.78 (m, 10H), 5.11 (s, 2H), 4.73 (s, 1H), 4.49 (d, *J* = 6.5 Hz, 1H), 3.75 (s, 2H), 2.65 (d, *J* = 5.3 Hz, 2H), 2.52 (d, *J* = 10.6 Hz, 1H), 2.31 (d, *J* = 12.9 Hz, 1H). ^{13}C NMR (101 MHz, $CDCl_3$) δ 174.4, 155.7, 128.5, 77.2, 67.6, 49.9, 36.9, 35.3, 33.4. ESI-TOF m/z: Calcd for [M+H$^+$] $C_{20}H_{22}NO_4S^+$: 372.1288, found [M+H$^+$]: 372.1270.

Benzyl ((3*S*,5*R*)-2-oxo-5-(phenoxymethyl)tetrahydrofuran-3-yl)carbamate (**14e**).

The precursor **3e** was prepared following the procedure described for *R*-**4e**. Reaction volume was 18 mL. Starting from **1e** (0.25 g, 1.8 mmol, 0.1M) and sodium pyruvate **2** (202.1 mg, 1.8 mmol, 1 eq), the reaction yielded 80% of **3e**. Transamination of **3e** using BCAT/L-Glu/L-Asp/AspAT system rendered 71% of **6e**. Cbz protection and conversion to α-amino-γ-butyrolactone derivative **14e** were performed following the **procedure 3**. Product was eluted with a step gradient of hexane:EtOAc: 100:0, 500 mL, 90:10, 200 mL, 80:20, 200 mL, 70:30, 500 mL, 60:40, 2 L affording the title compound **14e** as solid (dr: >95:5, 182 mg, 29%). $[\alpha]_{20}^{D}$ = – 35 (*c* = 1, in $CHCl_3$). ^{1}H NMR (400 MHz, $CDCl_3$) δ 7.40 – 7.22 (m, 7H), 7.02 – 6.81 (m, 3H), 5.12 (s, 2H), 4.91 (d, *J* = 8.6 Hz, 1H), 4.72 (td, *J* = 2x10.0, 6.5 Hz, 1H), 4.23 (d, *J* = 10.5 Hz, 1H), 4.05 (d, *J* = 10.3 Hz, 1H), 2.80 (d, *J* = 13.0 Hz, 1H), 2.48 (d, *J* = 11.7 Hz, 1H). ^{13}C NMR (101 MHz, CDCl3) δ 174.7, 155.4, 128.9, 121.9, 114.9, 75.4, 69.3, 67.4, 50.2. ESI-TOF m/z: Calcd for [M+H$^+$] $C_{19}H_{20}NO_5^+$: 342.1359, found [M+H$^+$]: 3421341.

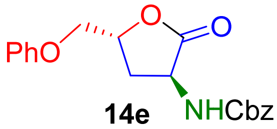

14e

Benzyl ((3*S*,5*R*)-2-oxo-5-(2-phenoxypropan-2-yl)tetrahydrofuran-3-yl)carbamate (14f).

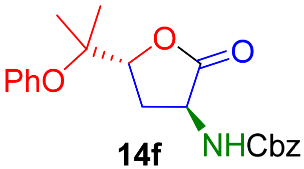

14f

The precursor **3f** was prepared following the procedure described above for *R*-**4f**. Reaction volume was 24 mL. Starting from **1f** (0.4 g, 2.4 mmol, 0.1 M) and sodium pyruvate **2** (268.1 mg, 2.4 mmol, 1 eq), the reaction afforded 63% of **3f**. Transamination of **3f** was performed using the BCAT/L-Glu/L-Asp/AspAT system, rendering 56% of **6f**. Cbz protection and conversion to α-amino-γ-butyrolactone derivative **14f** were performed following the **procedure 3**. Product was eluted with a step gradient of hexane:EtOAc: 100:0, 500 mL, 90:10, 200 mL, 80:20, 2.5 L, affording the title compound **14f** as a yellow oil (dr: >95:5, 98 mg, 11%). $[\alpha]_{20}^{D}$ = – 26 (*c* = 1, in $CHCl_3$). ^{1}H NMR (400 MHz, $CDCl_3$) δ 7.46 – 6.76 (m, 10H), 5.11 (s, 2H), 4.64 (td, *J* = 2x9.9, 6.3 Hz, 1H), 4.42 (s, 1H), 4.10 (q, *J* = 3x7.2, Hz, 1H), 3.01 (d, *J* = 10.3 Hz, 1H), 2.37 (d, *J* = 10.8 Hz, 1H), 1.31 (s, 3H), 1.29 (s, 3H). ^{13}C NMR (101 MHz, $CDCl_3$) δ 175.2, 155.7, 153.3, 129.1, 128.5, 124.2, 123.8, 83.7, 80.5, 67.3, 50.3, 30.3, 23.5, 22.6. ESI-TOF m/z: Calcd for [M+H$^+$] $C_{21}H_{24}NO_5^+$: 370.1686, found [M+H$^+$]: 370.1654.

Cbz removal of 14f. Synthesis of (3*S*,5*R*)-2-oxo-5-(2-phenoxypropan-2-yl)tetrahydrofuran-3-aminium chloride (16f).

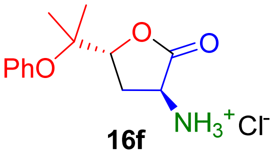

16f

To compound **14f** (50 mg, 135.4 µmol, 1 eq) in MeOH (15 mL), HCl (270 µL of 1 M stock solution, 2 eq) and Pd/C (1.34 mg, 10 mol%, 10% Pd, 50% humidity) were added. The suspension was stirred at room temperature for 1 hour under H_2 atmosphere. Reaction mixture was filtered through Celite® and the pellet was washed with MeOH (3 x 50 mL). Then, the solvent was removed *in vacuo* obtaining the title compound **16f** as a solid (18 mg, 49%) ^{1}H NMR (400 MHz, CD_3OD) δ 7.28 (t, *J* = 7.7 Hz, 2H), 7.11 (t, *J* = 7.4 Hz, 1H), 6.97 (d, *J* 1= 7.9 Hz, 2H), 4.67 (d, *J* = 9.0 Hz, 1H), 4.41 (t, *J* = 2x10.0 Hz, 1H), 3.06 (dd, *J* = 13.4, 9.5 Hz, 1H), 2.44 (dt, *J* = 13.3, 2x9.8 Hz, 1H), 1.35 (s, 3H), 1.28 (s, 3H). ^{13}C NMR (101 MHz, CD_3OD) δ 172.3, 153.5, 84.3, 81.0, 22.1, 21.5.

Isopropyl (2*R*,4*S*)-4-(((benzyloxy)carbonyl)amino)-5-oxotetrahydrofuran-2-carboxylate (14i).

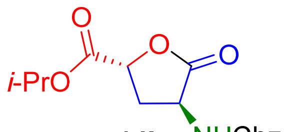

14i

The precursor **3i** was prepared following the procedure described for *R*-**4i**. Reaction volume was 16 mL. Starting from **1i** (190 mg, 1.6 mmol, 0.1M) and sodium pyruvate **2** (180.1 mg, 1.6 mmol, 1 eq), the reaction yielded 95% of **3i**. Transamination of **3i** was conducted using the T039/BnNH$_2$ system rendering 42% of **6i**. Cbz protection and conversion to α-amino-γ-butyrolactone derivative **14i** were performed following the **procedure 3**. Product was eluted with a step gradient of hexane:EtOAc: 100:0, 100 mL, 95:5, 100 mL, 90:10, 100 mL, 85:15, 100 mL, 80:20, 100 mL, 75:25, 100 mL, 70:30 1L affording the title compound **14i** as a solid (dr: >95:5, 179 mg, 34%). $[\alpha]_{20}^{D}$ = – 24.3 (*c* = 1, in CHCl$_3$). ^{1}H NMR (400 MHz, CDCl$_3$) δ 7.39 – 7.23 (m, 5H), 5.11 (s, 3H), 4.93 (d, *J* = 9.5 Hz, 1H), 4.46 (ddd, *J* = 11.4, 8.9, 6.0 Hz, 1H), 2.79 (dd, *J* = 13.0, 8.8 Hz, 1H), 2.53 (q, *J* = 3x11.6, Hz, 1H), 1.27 (dt, *J* = 6.3, 2x3.1, Hz, 6H). ^{13}C NMR (101 MHz, CDCl$_3$) δ 175.2, 168.7, 155.7, 128.7, 73.8, 70.5, 67.4, 48.9, 33.3, 21.6. ESI-TOF m/z: Calcd for [M+H$^+$] $C_{16}H_{20}NO_6^+$: 322.1284, found [M+H$^+$]: 322.1291.

(3*S*,5*S*)- and (3*S*,5*R*)-Benzyl (-2-oxo-5-((2-phenylacetamido)methyl)tetrahydrofuran-3-yl)carbamate ((5*R*/*S*)-14n) as a diasteromeric mixture.

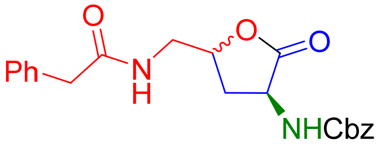

(5*R*/*S*)-**14n**

The precursor **3n** was prepared following the procedure described for **16b**. Reaction volume was 28 mL. Starting from **1n** (0.5 g, 2.8 mmol, 0.1M) and sodium pyruvate **2** (310.5 mg, 2.8 mmol, 1 eq) the reaction afforded 80% of **3n**. Transamination of **3n** was performed using the T039/BnNH$_2$ system, yielding 56% of **6n**. Cbz protection and conversion to α-amino-γ-butyrolactone derivatives (4*R*/*S*)-**14n** were performed following procedure 3. Product was eluted with a step gradient of pentane: EtOAc: 100:0, 500 mL, 90:10, 200 mL, 80:20, 200 mL, 70:30, 200 mL, 60:40, 200 mL, 50:50, 200 mL, 40:60, 200 mL, 30:70, 200 mL, 20:80, 2.2 L, affording the compound **(5*R*/*S*)-14n** as a distereomeric mixture (dr 50:50 (3*S*,5*S*):(3*S*,5*R*)), as a yellow oil (266 mg, 25%). $[\alpha]_{20}^{D}$ = – 11.2 (*c* = 1, in CHCl$_3$). **Benzyl ((3*S*,5*R*)-2-oxo-5-((2-phenylacetamido)methyl)tetrahydrofuran-3-yl)carbamate, (3*S*,5*R*)-14n)**: ^{1}H NMR (400 MHz, CD$_3$OD) δ 5.50 (s, 2H), 4.69 (dq, *J* = 9.3, 2x5.0, 4.9 Hz, 1H), 4.28 (t, *J* = 2x9.7 Hz, 1H), 3.50 (s, 2H), 3.43 (d, *J* = 5.6 Hz, 2H), 2.30 (m, 2H). ^{13}C NMR (101 MHz, CD$_3$OD) δ 176.0, 173.0, 156.9, 76.7, 66.4, 49.5 42.5, 42.4, 30.3. **Benzyl ((3*S*,5*S*)-2-oxo-5-((2-phenylacetamido)methyl)tetrahydrofuran-3-yl)carbamate, (3*S*,5*S*)-14n)**: ^{1}H

NMR (400 MHz, CD_3OD) δ 5.50 (s, 2H), 4.53 (dtd, *J* = 9.9, 2x5.9, 3.6 Hz, 1H), 4.45 (dd, *J* = 11.7, 9.1 Hz, 1H), 3.52 (d, *J* = 3,5 Hz, 1H), 3.50 (s, 2H), 3.42 (d, *J* = 5.6 Hz, 1H), 2.50 (ddd, *J* = 12.3, 9.1, 5.8 Hz, 1H), 1.97 (q, *J* = 2x11.8, 11.6 Hz, 1H). ^{13}C NMR (101 MHz, CD_3OD) δ 175.1, 173.0, 156.9, 75.9, 66.4, 48.4, 42.5, 42.4, 27.4.

Selective deprotection of (5*R*/*S*)-14n mixture.

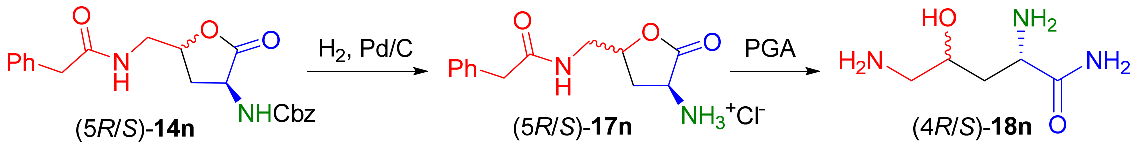

A portion of (5*R*/*S*)-**14n** mixture (100 mg, 261.5 μmol, 1 eq) in MeOH (28 mL), HCl (500 μL of 1 M stock solution) and Pd/C (2.8 mg, 10 mol%, 10% Pd, 50% humidity) were mixed. The suspension was stirred at room temperature for 1 hour under H_2 atmosphere. Reaction mixture was filtered through Celite® and the pellet was washed with MeOH (3 x 50 mL). Then, the solvent was removed *in vacuo* obtaining (5*R*/*S*)-**17n** as solid (68 mg, 91%). (5*R*)-**17n**: ^{1}H NMR (400 MHz, CD_3OD) δ 4.76 (dddd, *J* = 9.1, 6.0, 4.9, 2.1 Hz, 1H), 4.02 (t, *J* = 2x10.1 Hz, 1H), 3.52 (s, 2H), 3.49 (d, *J* = 2.3 Hz, 2H), 2.52 (ddd, *J* = 13.5, 9.7, 2.1 Hz, 1H), 2.32 (ddd, *J* = 13.5, 10.4, 9.0 Hz, 1H). ^{13}C NMR (101 MHz, CD_3OD) δ 173.6, 171.8, 78.4, 48.4, 42.4, 42.2, 28.4. (5*S*)-**17n**: ^{1}H NMR (400 MHz, CD_3OD) δ 4.64 (dddd, *J* = 13.5, 10.4, 9.0 Hz, 1H), 4.39 (dd, *J* = 12.0, 8.9 Hz, 1H), 3.59 (d, *J* = 4.0 Hz, 1H), 3.52 (s, 2H), 3.42 (d, *J* = 2.3 Hz, 1H), 2.71 (ddd, *J* = 12.5, 8.8, 5.4 Hz, 1H), 1.96 (m,1H). ^{13}C NMR (101 MHz, CD_3OD) δ 173.3, 171.5, 78.1, 50.5, 42.4, 42.1, 30.3. The hydrogenated compound obtained (60 mg, 210 μmol) was re-suspended in a volume of 155 mM sodium phosphate buffer (2.4 mL) and Penicillin G Amidase (PGA) from Roche diagnostics GmbH, Mannheim (226 μL of a stock solution 1140 U mL^{-1}, 100 U mL^{-1} final concentration in the reaction) were added. The reaction was stirred (1000 rpm) at 25 °C. Reaction monitoring was carried out by HPLC immediately after the enzyme addition (0 h) and after 24 h in the conditions described above. When the reaction was completed the enzyme was precipitate by adding 100 mM HCO_2H (3 mL) and centrifuged. The pellet was washed with 100 mM HCO_2H (3 x 3 mL). Aqueous solution was adjusted to 30 mL and was purified by ionic exchange chromatography, was performed on Macro-Prep® High S Media (25 mL, Bio-Rad) (packed into a glass column (C16/20, GE HealthcareLife Science), equilibrated with 1 M $SO_4(NH_4)_2$ (500 mL). Crude fraction was loaded onto the column at 1 mL min^{-1}. Colored impurities were washed away

with water (90 mL) at 3 mL min^{-1}. Product was eluted with a gradient of 1 M NH_4OH (420 mL) at 3 mL min^{-1}, typical fraction size was 30 mL. Fractions were lyophilized and the (4*R*/*S*)-**18n** mixture was obtained as a solid (28 mg, 60%). (4*R*)-**18n**: ^{1}H NMR (400 MHz, D_2O) δ 4.00 (dddd, *J* = 10.6, 8.8, 6.5, 3.4 Hz, 1H), 3.90 (dd, *J* = 6.2, 5.3 Hz, 1H), 3.09 (dd, *J* = 3.3, 2.3 Hz 1H), 2.90 (dd, *J* = 8.4, 4.8 Hz, 1H), 2.00 (m, 2H). ^{13}C NMR (101 MHz, D_2O) δ 173.7, 64.9, 52.1, 44.4, 33.8. (4*S*)-**18n**: ^{1}H NMR (400 MHz, D_2O) δ 4.12 (ddt, *J* = 10.4, 8.8, 2x3.1 Hz, 1H), 3.82 (t, *J* = 2x6.8 Hz, 1H), 3.09 (ddd, *J* = 3.4, 2.25 Hz, 1H), 2.90 (dd, *J* = 8.3, 4.8 Hz, 1H), 2.06 (ddd, *J* = 14.7, 6.6, 2.9 Hz, 1H), 1.80 (ddd, *J* = 14.7, 6.9, 10.4 Hz, 1H). ^{13}C NMR (101 MHz, D_2O) δ 173.7, 65.9, 52.6, 44.4, 34.7.

(3*S*,5*S*)- and (3*S*,5*R*)-Benzyl (2-oxo-5-((*S*)-1-(2-phenylacetamido)ethyl)tetrahydrofuran-3-yl)carbamate ((5*R*/*S*)-14o) as a diasteromeric mixture.

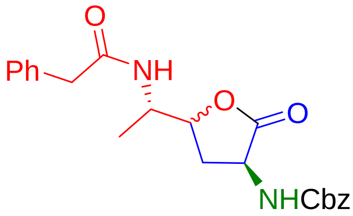

(4*R*/*S*)-**14o**

The precursor **3o** was prepared following the procedure described above **14c**. Reaction volume was 26 mL. The reaction was started by adding the enzyme HBPA H205A (9.2 mL of a stock solution 0.009 U mL^{-1}, 2.8 mg mL^{-1} in 50 mM TEA buffer, 50 mM NaCl, 0.5 mM EDTA and 50% (v/v) of glycerol, 0.00318 U mL^{-1}, 1 mg protein mL^{-1} final concentration in the reaction) to a solution of **1o** (0.5 g, 2.6 mmol, 0.1 M) and sodium pyruvate **2** (287.7 mg, 1 eq). This rendered 75% of **3o**. Transamination of **3o** was conducted using the BCAT/L-Glu/L-Asp/AspAT system, affording 50% of **6o**. Cbz protection and conversion to α-amino-γ-butyrolactone derivatives (5*R*/*S*)-**14o** were performed following procedure 3. Product was eluted with a step gradient of pentane:EtOAc: 100:0, 500 mL, 90:10, 200 mL, 80:20, 200 mL, 70:30, 200 mL, 60:40, 200 mL, 50:50, 200 mL, 40:60, 200 mL, 30:70, 3 L, affording a diastereomeric mixture (5*R*/*S*)-**14o** (dr 50:50 as a yellow oil (257 mg, 20%). $[\alpha]_{20}^{D}$ = – 22 (*c* = 1, in $CHCl_3$). (5*R*)-**14o**: ^{1}H NMR (400 MHz, $CDCl_3$) δ 5.47 (d, *J* = 8.9 Hz, 1H), 5.35 (d, *J* = 5.4 Hz, 1H), 5.10 (s, 2H), 4.54 (m, 1H), 4.22 (m, 1H), 4.14 (d, *J* = 8.6 Hz, 1H), 3.58 (s, 1H), 3.48 (s, 1H), 2.42 (t, *J* = 2x12.6 Hz, 1H), 2.29 (m, 1H), 1.08 (d, *J* = 6.8 Hz, 3H). ^{13}C NMR (101 MHz, $CDCl_3$) δ 175.1, 170.6, 155.7, 80.1, 67.5, 49.7, 47.8, 43.8, 30.8, 14.8. (5*S*)-**14o**:^{1}H NMR (400 MHz, $CDCl_3$) δ 5.71 (d, *J* = 9.2 Hz, 1H), 5.10 (s, 2H), 5.02 (d, *J* = 7.1 Hz, 1H), 4.35 (m, 1H), 4.33 (m, 1H), 4.23 (m, 1H), 3.58 (s, 1H), 3.48 (s, 1H), 2.54 (ddd, *J* =

12.9, 9.1, 5.8 Hz, 1H), 1.78 (q, *J* = 3x11.8, 1H), 1.24 (d, *J* = 7.1 Hz, 3H). ^{13}C NMR (101 MHz, $CDCl_3$) δ 173.8, 171.3, 155.7, 79.7, 67.5, 51.2, 46.0, 43.9, 31.7, 18.2.

Selective deprotection of (5*R*/*S*)-14o mixture.

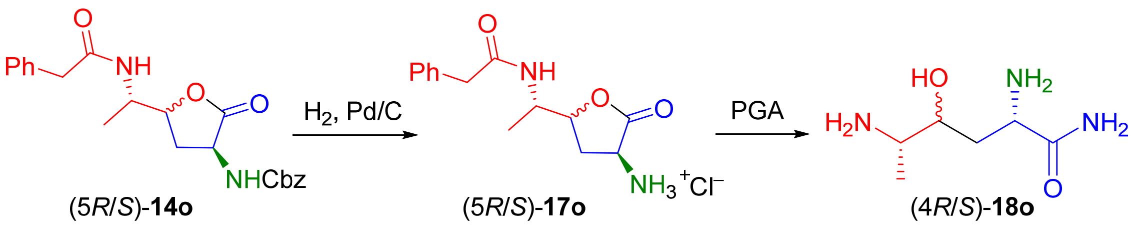

The selective deprotection of the PheAc and Cbz groups was conducted following the procedure described for (5*R*/*S*)-**14n**, starting from the (4*R*/*S*)-**14o**' mixture (120 mg, 302.3 µmol) yielding (5*R*/*S*)-**17o** as a solid (90 mg, >99%). (5*R*)-**17o**: ^{1}H NMR (400 MHz, CD_3OD) δ 4.59 (dt, *J* = 23.7, 2x6.6 Hz, 1H), 4.42 (m, 1H), 4.15 (m, 1H), 3.52 (s, 2H), 2.69 (t, *J* = 10.6 Hz, 1H), 2.03 (t, *J* = 11.2 Hz, 1H), 1.22 (m, 3H).^{13}C NMR (101 MHz, CD_3OD) δ 172.7, 171.5, 80.2, 49.4, 46.9, 42.3, 29.9, 15.3. (5*S*)-**17o**:^{1}H NMR (400 MHz, CD_3OD) δ 4.55 (dd, *J* = 15.8, 7.8 Hz, 1H), 4.24 (t, *J* = 2x9.8 Hz, 1H), 4.10 (m, 1H), 3.52 (s, 2H), 2.56 (dd, *J* = 13.4, 9.4 Hz, 1H), 2.31 (dt, *J* = 13.6, 2x10.2 Hz, 1H), 1.21 (m, 3H).^{13}C NMR (101 MHz, CD_3OD) δ 172.8, 171.8, 80.6, 47.3, 46.8, 42.3, 27.9, 15.1. After removal the PheAc as described for (4*R*/*S*)-**18n** gave (4*R*/*S*)-**18o** (58 mg, 81%). (4*R*)-**18o**: ^{1}H NMR (400 MHz, D_2O/ HCO_2H (0.1M)) δ 4.04 (t, *J* = 2x2.9 Hz, 1H), 3.77 (t, *J* = 2x6.7 Hz, 1H), 3.32 (tt, *J* = 2x6.7, 2x3.3 Hz, 1H), 1.97 (d, *J* = 4.0 Hz, 1H), 1.72 (dd, *J* = 7.3, 3.7 Hz, 1H), 1.11 (d, *J* = 6.9 Hz, 3H). ^{13}C NMR (101 MHz, D_2O) δ 173.3, 68.6, 52.5, 51.2, 32.5, 11.4. (4*S*)-**18o**: ^{1}H NMR (400 MHz, D_2O/HCO_2H (0.1M)) δ 3.89 (dd, *J* = 7.3, 3.8 Hz, 1H), 3.65 (ddd, *J* = 10.1, 7.0, 2.9 Hz, 1H), 3.19 (p, *J* = 4x6.8 Hz, 1H), 2.02 (m, 1H), 1.93 (m, 1H), 1.16 (d, *J* = 6.8 Hz, 3H). ^{13}C NMR (101 MHz, D_2O) δ 173.6, 69.2, 51.8, 32.7, 14.6.

Chemical synthesis of 1-Benzyl 2-methyl (2*S*,4*R*)- and (2*S*,4*S*)-4-hydroxypyrrolidine-1,2-dicarboxylate ((2*S*,4*R*)-15k and (2*S*,4*S*)-15k).

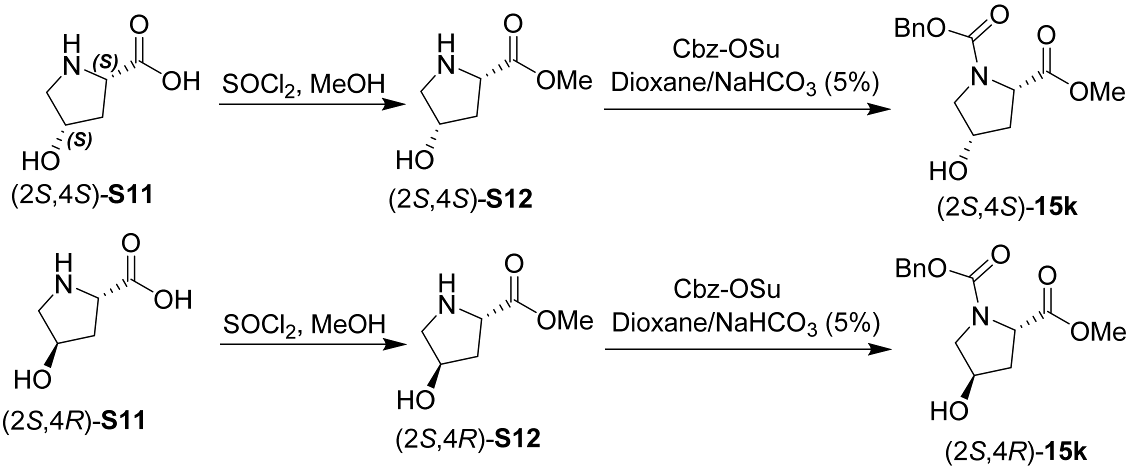

To a separate solutions of commercial diasteromers (2*S*,4*R*)-**S11** and (2*S*,4*S*)-**S11** (0.5 g, 3.8 mmol) in anhydrous MeOH (50 mL) at – 80 ºC, $SOCl_2$ (1.8 g, 15.3 mmol, 4 eq) was slowly added stirring. When the addition was finished the solution was allow to warm up to rt. After stirring for 24 h, the excess acid generated was removed under vacuum affording **S12** (0.55 g, quantitative). **S12** (3.7 mmol, 1 eq), was diluted with $NaHCO_3$ 20% w/v (60 mL) and Cbz-OSu (0.93 g, 3.7 mmol, 1 eq, dissolved in dioxane (60 mL)) was then added. The reactions were stirred at 25 °C for 12 h. The products were extracted with EtOAc (3 x 100 mL) and washed with H_2O (3 x 100 mL), brine (3 x 100 mL). Then, the organic phases were dried over anhydrous $MgSO_4$ and concentrated under vacuum yielding the compound (2*S*,4*R*)-**15k** as a solid (1.1 g, >99%) and (2*S*,4*S*)-**15k** as an oil (1.1 g, >99%). (2*S*,4*R*)-**15k**: 2-rotamers double signal NMR: ^{1}H NMR (401 MHz, $CDCl_3$) δ 7.40-7.29 (m, 5H), 5.23 – 5.02 (m, 2H), 4.52 (m, 2H), 3.77 (s, 1H), 3.56 (s, 1H), 3.73 – 3.69 (m, 3H), 2.32 (m, 1H), 2.11 (m, 1H). ^{13}C NMR (101 MHz, $CDCl_3$) δ 173.2 (rot 1), 173.0 (rot 2), 155.0 (rot 1), 154.5 (rot 2), 70.2 (rot 1), 69.5 (rot 2), 67.2 (rot 1), 67.1 (rot 2), 54.7 (rot 1), 55.3 (rot 2), 52.4 (rot 1), 52.2 (rot 2), 39.2 (rot 1), 38.4 (rot 2). 1-Benzyl 2-methyl (2*S*,4*S*)-4-hydroxypyrrolidine-1,2-dicarboxylate, (2*S*,4*S*)-**15k**: 2-rotamers double signal NMR: ^{1}H NMR (401 MHz, $CDCl_3$) δ 7.40 – 7.29 (m, 5H), 5.23-5.06 (m, 2H), 4.46 (m, 1H), 4.41 (dt, *J* = 5.7, 2x3.0 Hz, 1H) 3.82 (s, 1H), 3.79 (s, 1H), 3.79-3.60 (m, 3H), 3.60 (d, *J* = 4.4, Hz, 1H) 2.33 (tdd, *J* = 2x14.3, 9.8, 4.6 Hz, 1H), 2.16 (dd, *J* = 14.2, 5.0 Hz 1H). ^{13}C NMR (101 MHz, $CDCl_3$) δ 175.3 (rot 1), 175.0 (rot 2), 155.0 (rot 1), 154.2 (rot 2), 71.3 (rot 1), 70.3 (rot 2), 67.4 (rot 1), 67.3 (rot 2), 58.2 (rot 1), 57.7 (rot 2), 52.8 (rot 1), 52.6 (rot 2), 38.7 (rot 1), 37.8 (rot 2).

Chemoenzymatic synthesis of 1-benzyl-2-methyl (2*S*,4*R*)-4-hydroxypyrrolidine-1,2-dicarboxylate (15k).

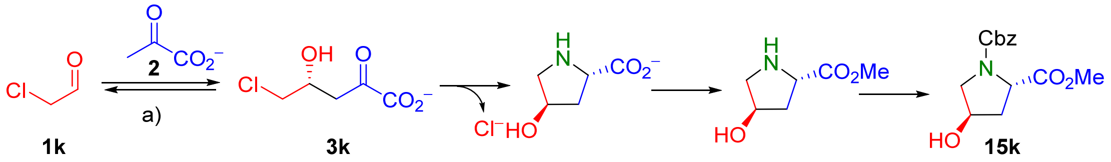

The precursor 4-hydroxy-2-oxoacid (**3k**) was prepared following the procedure described for **14c**. Reaction volume was 38 mL. To a solution of **1k** (490 µL of a 7.9 M commercial aqueous solution, 3.8 mmol, 1 eq, 0.1 M) and sodium pyruvate **2** (0.42 g, 3.8 mmol, 1 eq), HBPA H205A (14 mL of a stock solution 0.009 U mL^{-1}, 2.8 mg mL^{-1} in 50 mM TEA buffer, 50 mM NaCl, 0.5 mM EDTA and 50% (v/v) of glycerol, 0.00324 U mL^{-1}, 1 mg mL^{-1} of protein final concentration in the reaction) was added. This rendered 64% of **3k**. Transamination of **3k** was conducted using the BCAT/L-Glu/L-Asp/AspAT system, affording 81% of *trans*-4-hydroxy-L-proline. The *trans*-4-hydroxy-L-proline was formed by an intramolecular nucleophilic substitution of the terminal chloro at C5 by the amine group after the transamination reaction. The derivative **15k** (yellow oil, 611 mg, 51%) was prepared following the procedure described for the mixture (2*S*,4*R*)-**15k:**(2*S*,4*S*)-**15k**. Product **15k** was eluted with a step gradient of pentane:EtOAc: 100:0, 500 mL, 90:10, 200 mL, 80:20, 200 mL, 70:30, 200 mL, 60:40, 200 mL, 50:50, 200 mL, 40:60, 2.2 L (611 mg, 51%). The NMR spectra showed the presence of two rotamers, ^{1}H NMR (400 MHz, $CDCl_3$) δ 7.45 – 7.14 (m, 5H), 5.13 (dt, *J* = 24.0, 2x12.3 Hz, 1H), 5.00 (d, *J* = 12.4 Hz, 1H), 4.49 (m, 1H), 3.73-3.68 (m, 3H), 3.67 (m, 1H), 3.64 (dd, *J* = 4.2, 2.3 Hz, 2H), 3.53 (s, 1H), 2.30 (m, 1H), 2.28 (m, 1H), 2.09 (d, *J* = 1.7 Hz, 1H), 2.07 (d, *J* = 1.7 Hz, 1H). ^{13}C NMR (101 MHz, $CDCl_3$) δ 173.1 (rot 1), 172.3 (rot 2), 155.0 (rot 1), 154.5 (rot 1), 70.2 (rot 1), 69.4 (rot 1), 67.3, 57.9 (rot 1), 57.7 (rot 2), 55.2 (rot 1), 54.6 (rot 2), 52.4 (rot 1), 52.1 (rot 2), 39.2 (rot 1), 38.4 (rot 2). $[\alpha]_{20}^{D}$ = – 55.4 (*c* = 1, in MeOH). ESI-TOF m/z: Calcd for $[M+H^+]$ $C_{14}H_{18}NO_5^+$: 280.1193, found $[M+H^+]$: 380.1185.

Analysis of the diasteromeric excess of 15k

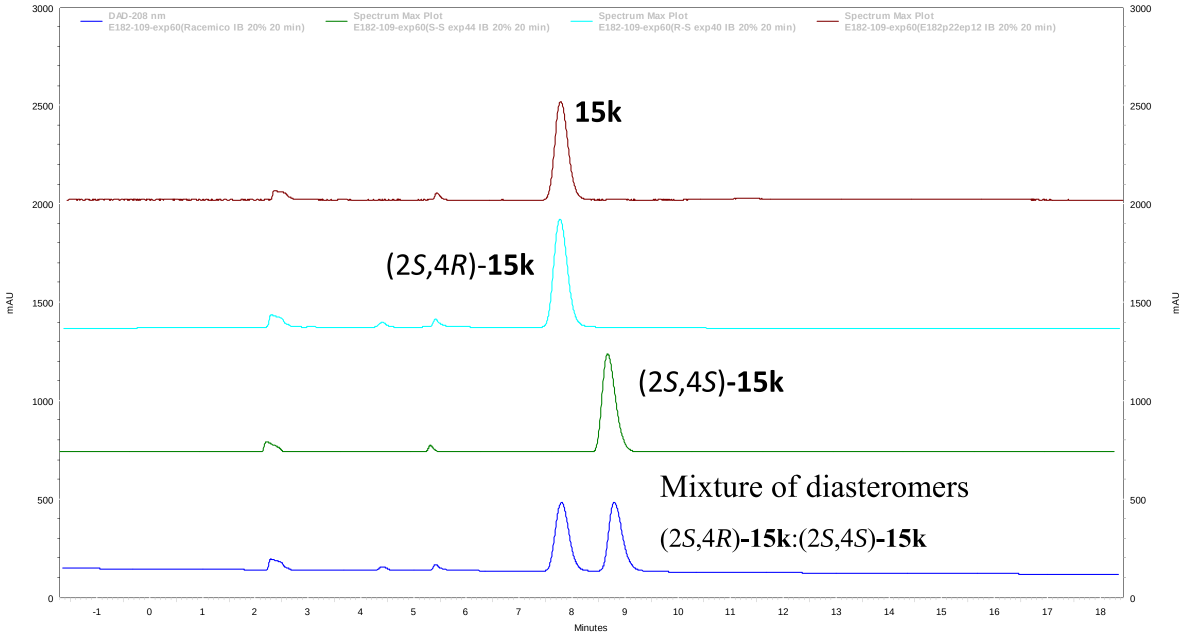

Figure S26: Chiral HPLC analysis chromatogram of **15k** synthetized by HBPA H205A and BCAT/L-Glu/L-Asp/AspAT transamination system. Conditions: CHIRALPACK® IB 46 x 250 mm column, 5 µm, flow rate 1 mL min^{-1} at 25 °C and UV detection (208 nm). Isocratic elution hexane:^{i}PrOH 80:20 (v/v).

Synthesis of *trans*-4-hydroxy-L-proline methyl ester (23k)

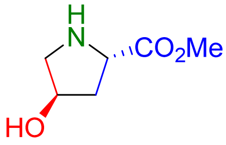

23k

A portion of (2*S*,4*R*)-**15k** (50 mg, 158.4 µmol, 1 eq.) in MeOH (16 mL), HCl (500 µL of 1 M stock solution) Pd/C (1.7 mg, 10 mol%, 10% Pd, 50% humidity) were mixed. The suspension was stirred at rt for 1 hour under H_2 atmosphere. The reaction mixture was filtered through Celite® and the pellet was washed with MeOH (3 x 50 mL). Then, the solvent was removed *in vacuo* obtaining methyl (2*S*,4*R*)-4-hydroxypyrrolidine-2-carboxylate, **23k** as an oil (38 mg, quantitative). ^{1}H NMR (400 MHz, CD_3OD) δ 4.63 – 4.54 (m, 2H), 3.43 (d, *J* = 10.0 Hz, 1H), 3.30 (d, *J* = 7.9 Hz, 1H), 2.40 (dd, *J* = 13.4, 7.5 Hz, 1H), 2.18 (t, *J* = 10.5, 10.5 Hz, 1H). ^{13}C NMR (101 MHz, CD_3OD) δ 169.2 69.2, 58.0, 52.6, 37.2.

(2*S*,4*R*)-4-Hydroxy-5-oxopyrrolidine-2-carboxylic acid (15h).

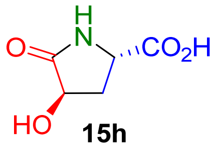

15h

The precursor **3h** was prepared following the procedure described for *R*-**4h**. The reaction volume was 20 mL. Starting from **1h** (400 µL of a 4.9 M commercial aqueous solution, 2.0 mmol, 1 eq, 0.1 M) and sodium pyruvate **2** (216 mg, 2.0 mmol, 1 eq), the reaction yielded 82% of **3h**. Transamination of **3h** using BCAT/L-Glu/L-Asp/AspAT system rendered 38% of **6h**. When the reaction was completed, the enzymes were precipitate by adding methanol (10 volumes, 260 mL). Then, the reaction was filtered through Celite® and the filter cake washed with methanol (3x50 mL). The filtrates were pooled and the solvent removed under vacuum, until all MeOH was evaporated and only the aqueous solution remained. The residue, was diluted with $NaHCO_3$ 20% w/v (50 mL). The reaction was stirred at 25 °C for 12 h. Then, the aqueous phase was concentrated up to 25 mL and after that, it was adjusted to pH 10.0 with a NaOH solution. The title compound (**15h**) was partially purified by ionic exchange chromatography, in a Macro-Prep® High Q Media (25 mL, Bio-Rad) stationary phase in H^+ form, packed into a glass column (C16/20, GE HealthcareLife Science). The stationary phase was equilibrated with 1 M HCO_2Na. Crude fraction at pH 10.0 (25 mL) was loaded onto the column at 1 mL min^{-1}. Colored impurities were washed away with water (75 mL) at 3 mL min^{-1}. Product was eluted with a gradient from 0 to 100% HCO_2H (1 M) in 50 min at 3 mL min^{-1}, typical fraction size was 25 mL. Fractions were lyophilized and the title compound **15h** was obtained as a yellow solid (65.4 mg, 9%). ^1H NMR (500 MHz, D_2O) δ 4.46 (dd, *J* = 10.1, 3.6 Hz, 1H), 3.99 (dt, *J* = 8.0, 2x4.0 Hz, 1H), 2.50 (ddd, *J* = 15.0, 5.2, 3.6 Hz, 1H), 2.06 (m, 1H). ^{13}C NMR (126 MHz, D_2O) δ 177.3, 173.2, 68.8, 52.8, 33.9. The final material contains L-Asp and L-Glu as main impurities coming from the transamination reaction.

NMR spectra.

Starting materials.

a)

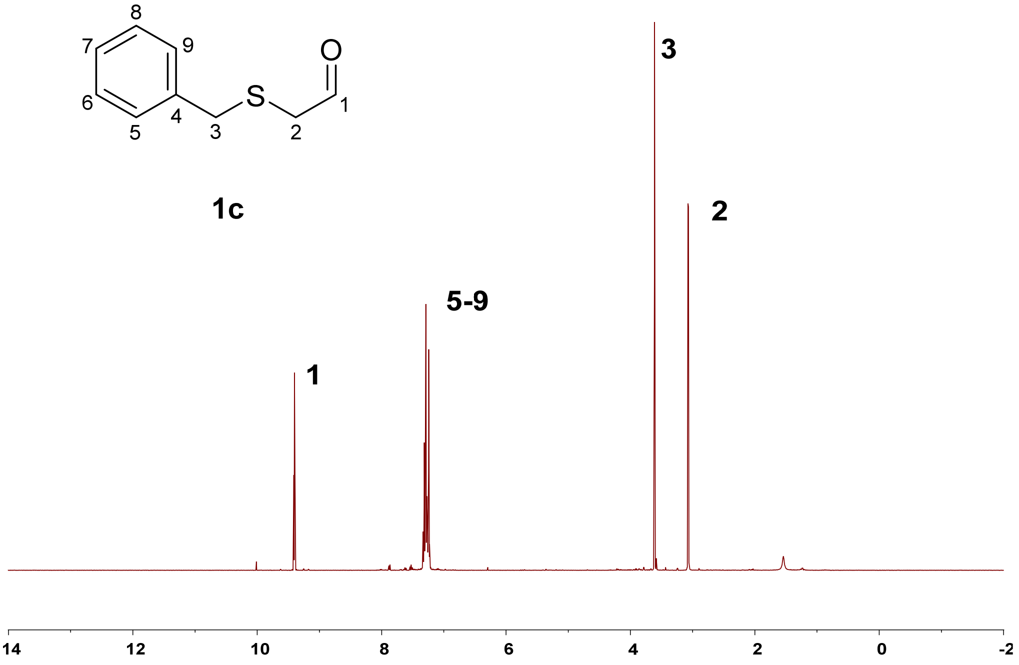

b)

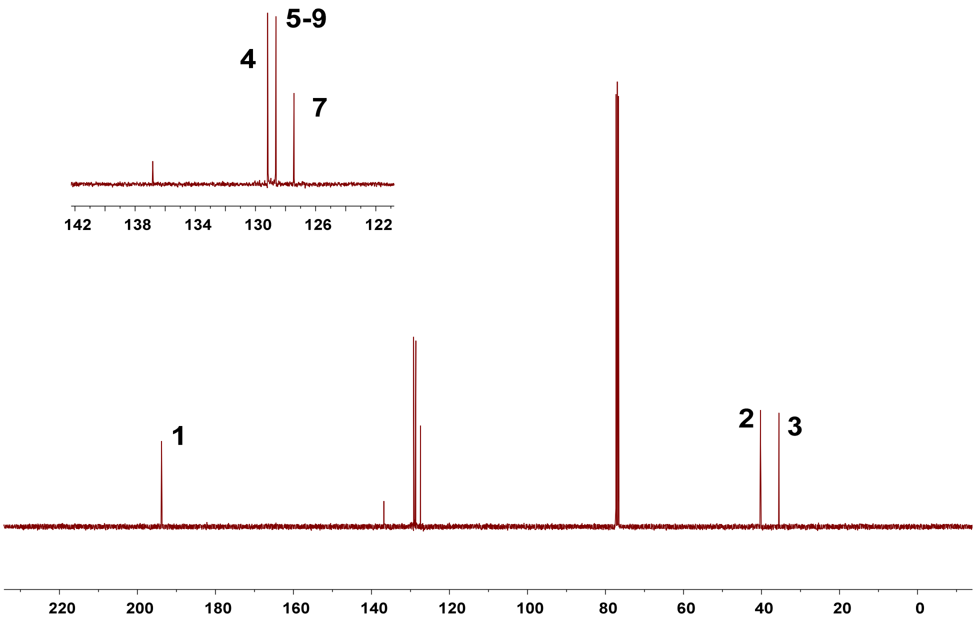

Figure S27. NMR spectra ($CDCl_3$) of **1c**: a) ^1H, b) ^{13}C.

a)

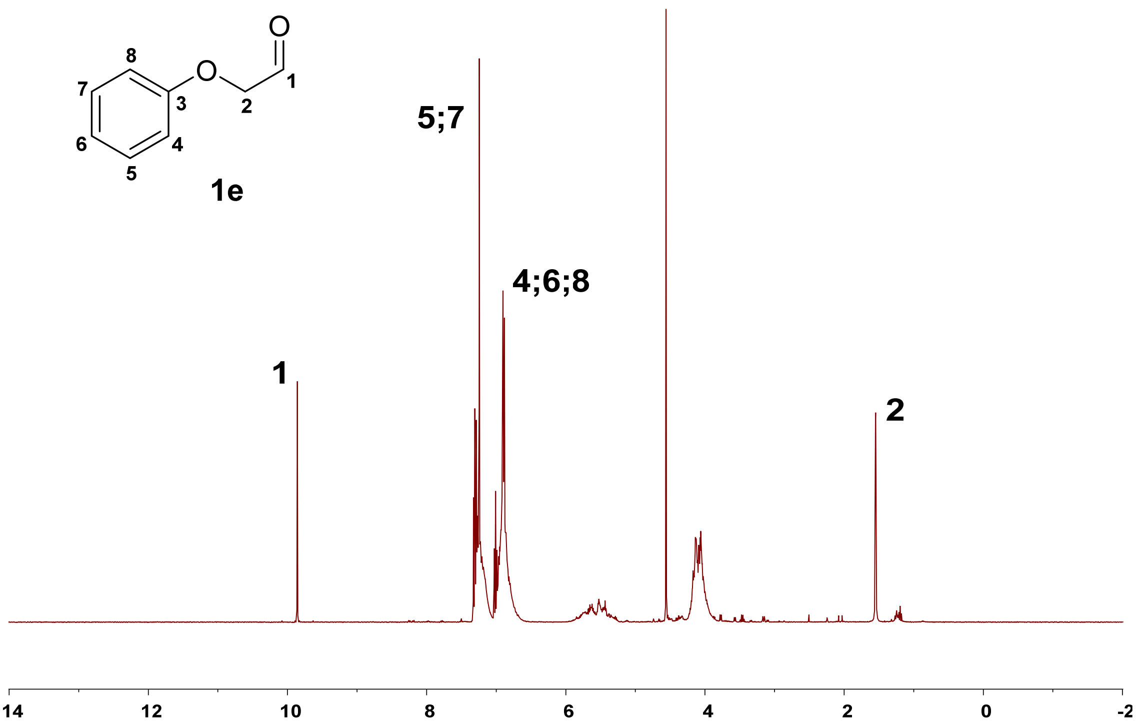

b)

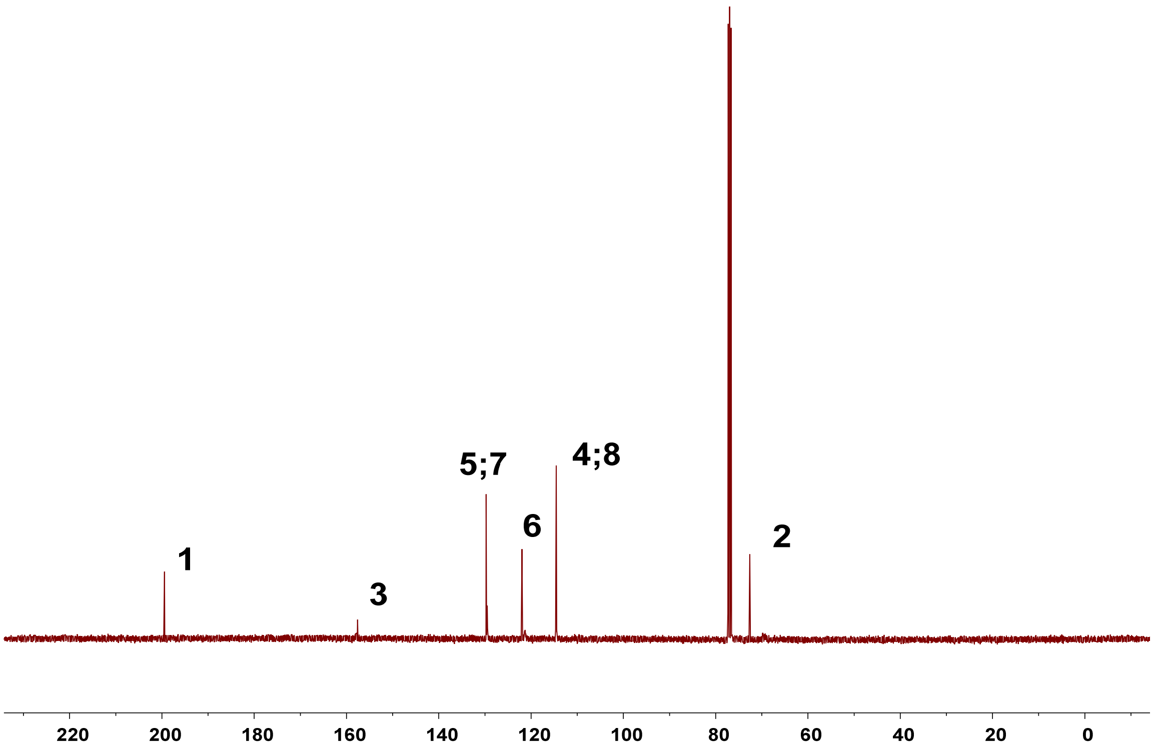

Figure S28. NMR spectra ($CDCl_3$) of **1e**: a) ^{1}H, b) ^{13}C.

a)

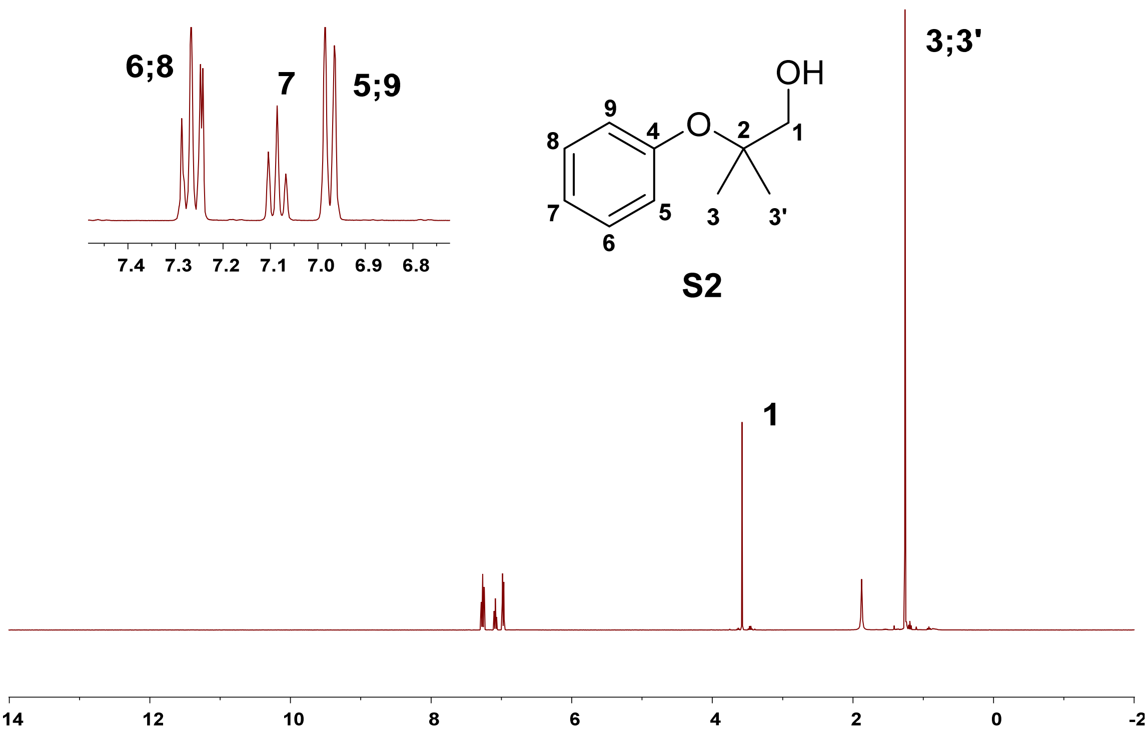

b)

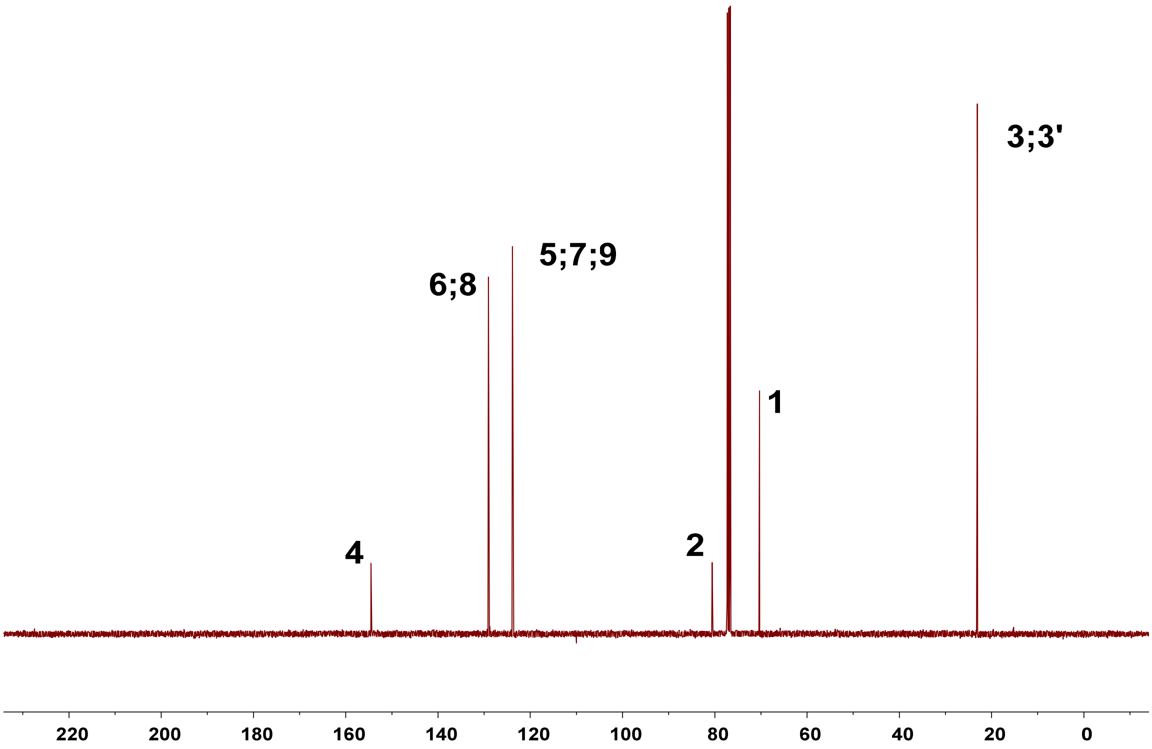

Figure S29. NMR spectra ($CDCl_3$) of **S2**: a) ^{1}H, b) ^{13}C.

a)

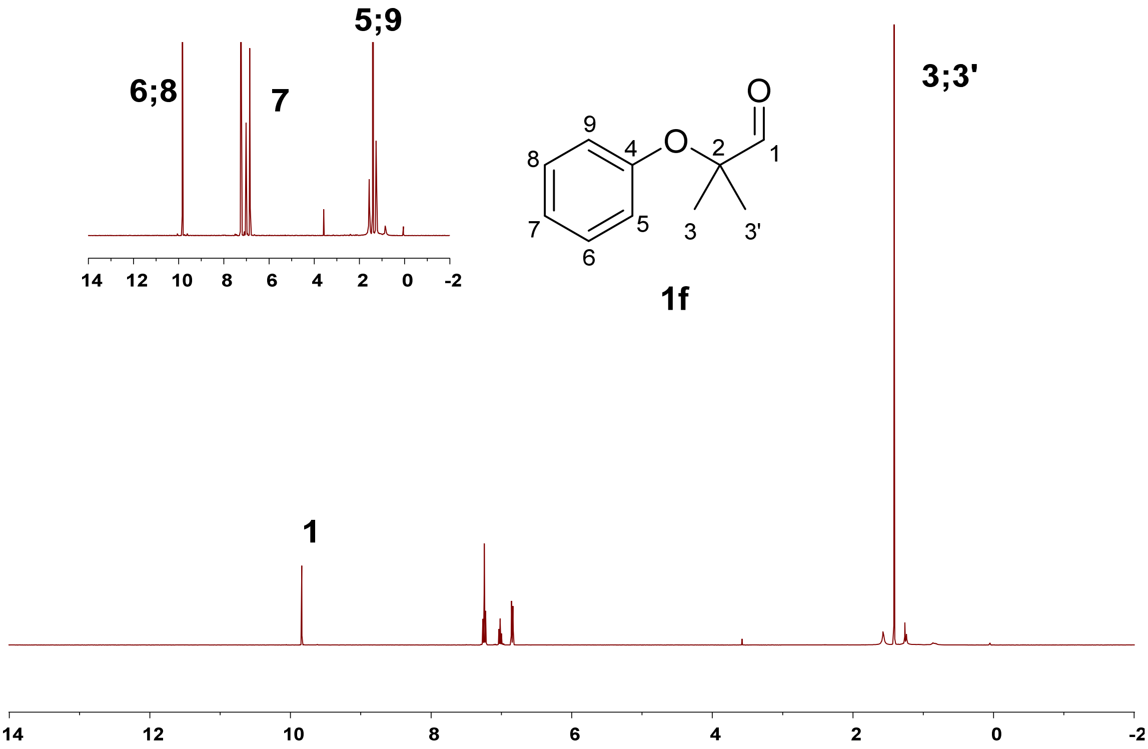

b)

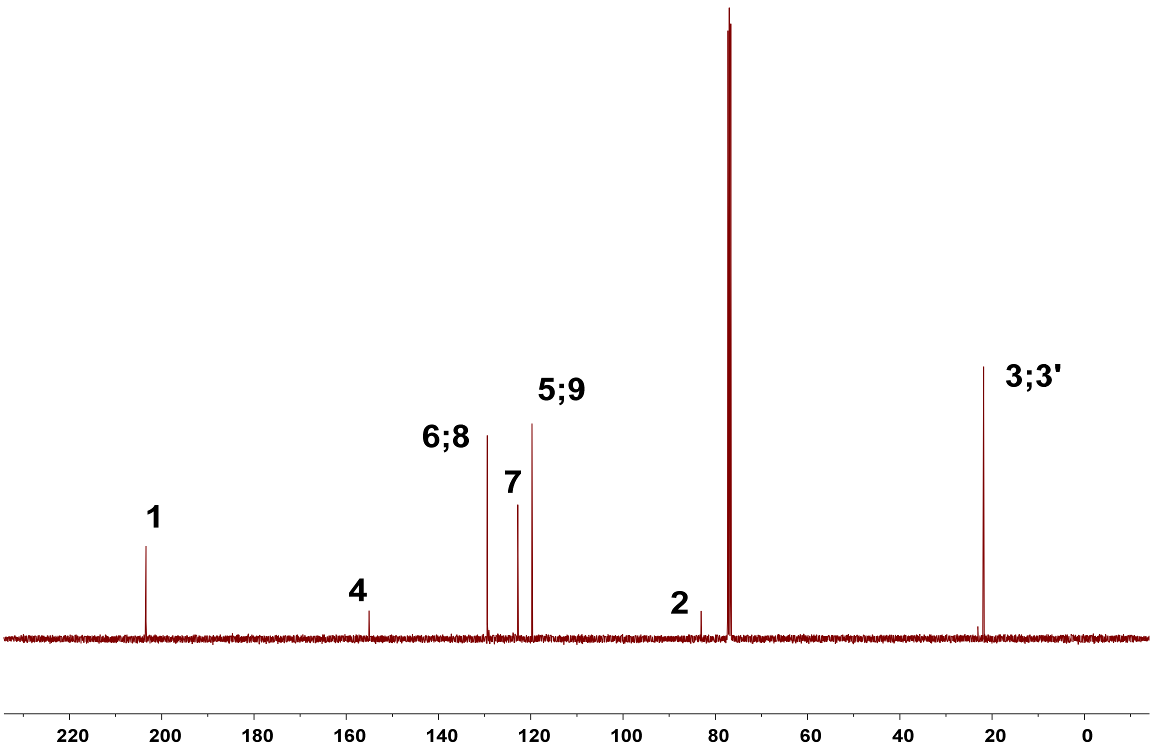

Figure S30. NMR spectra ($CDCl_3$) of **1f**: a) ^{1}H, b) ^{13}C.

a)

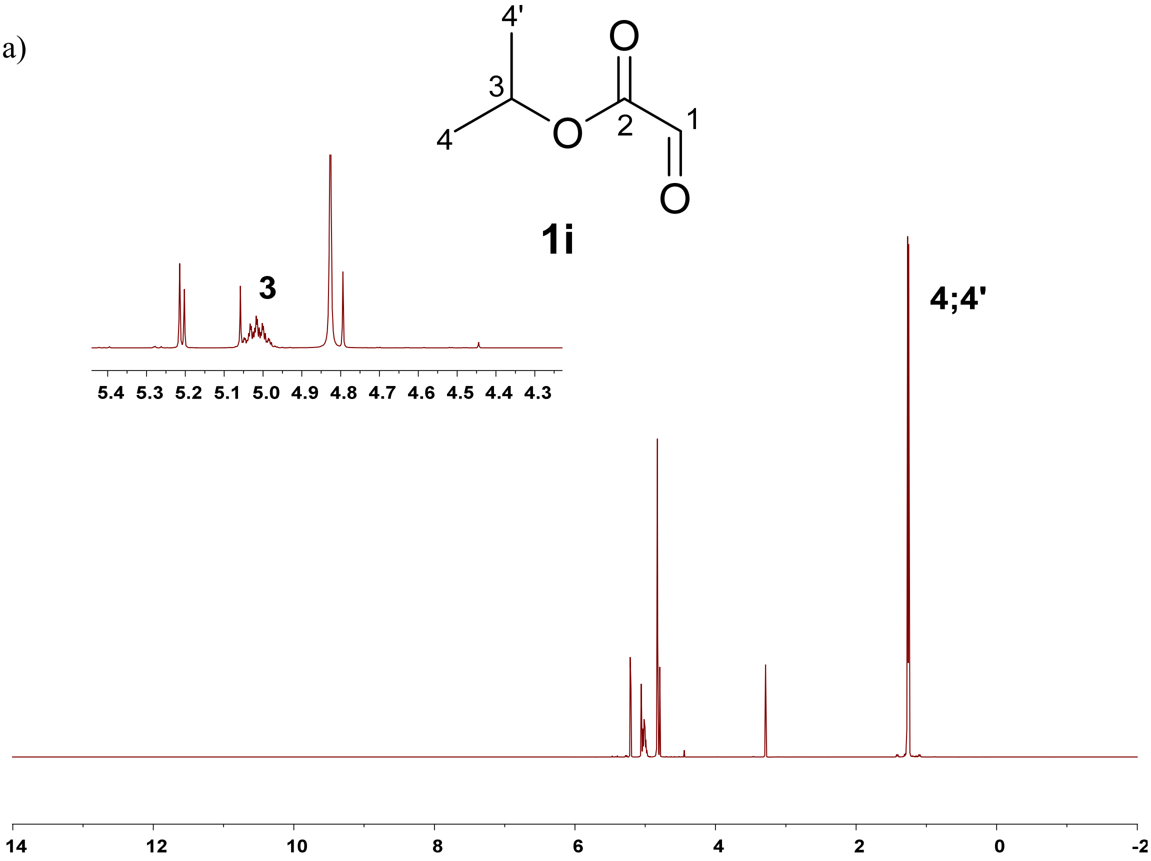
4'
3
4
O
2
1
O
O
1i
3
4;4'
5.4 5.3 5.2 5.1 5.0 4.9 4.8 4.7 4.6 4.5 4.4 4.3
14 12 10 8 6 4 2 0 -2

b)

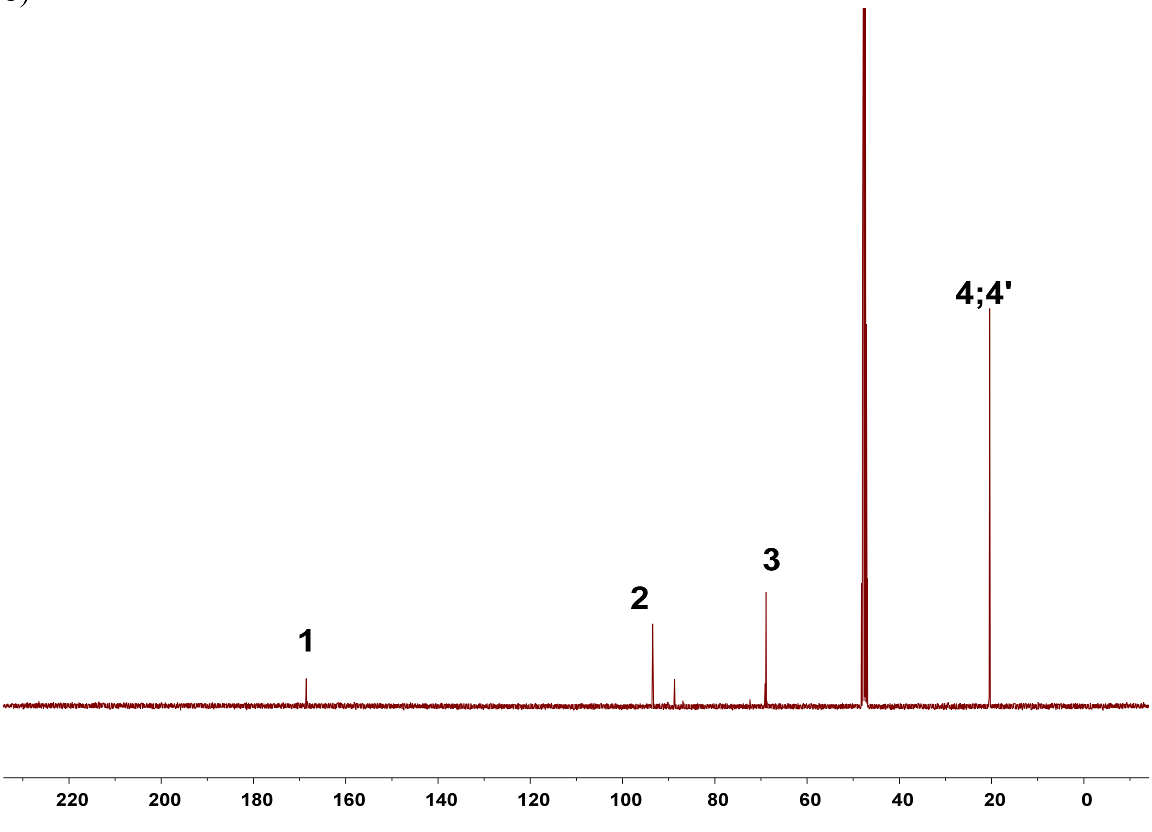
4;4'
3
2
1
220 200 180 160 140 120 100 80 60 40 20 0

c)

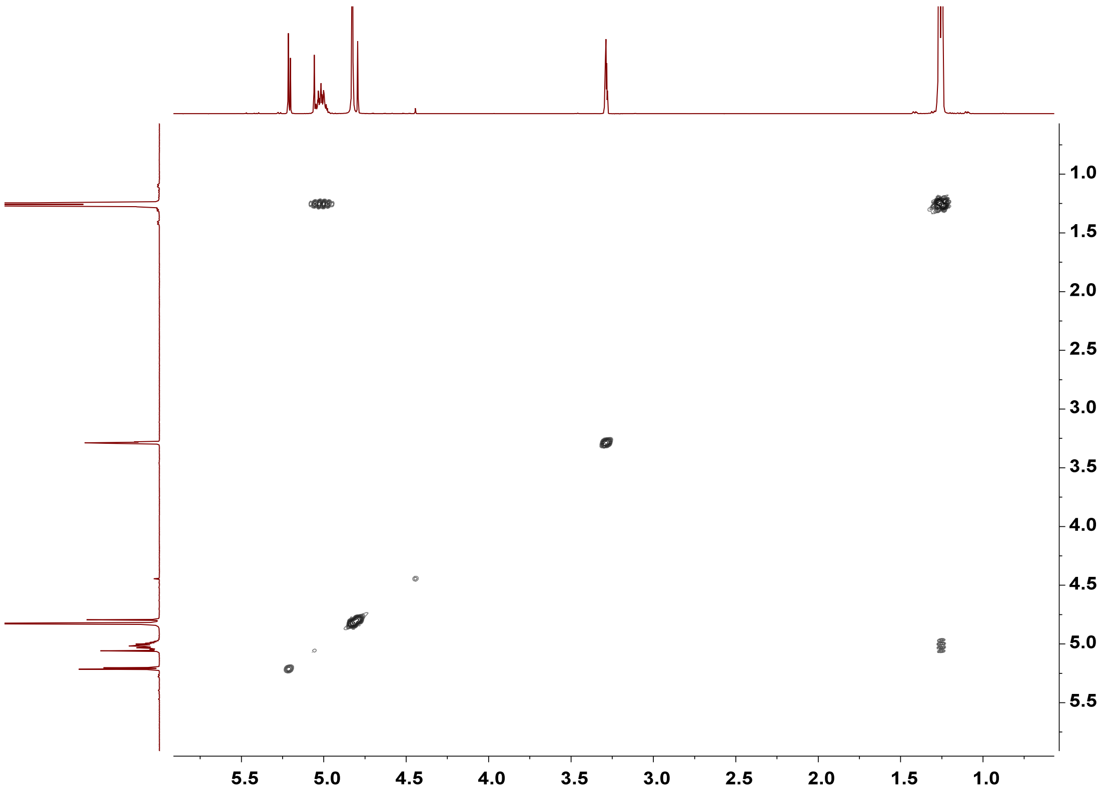

d)

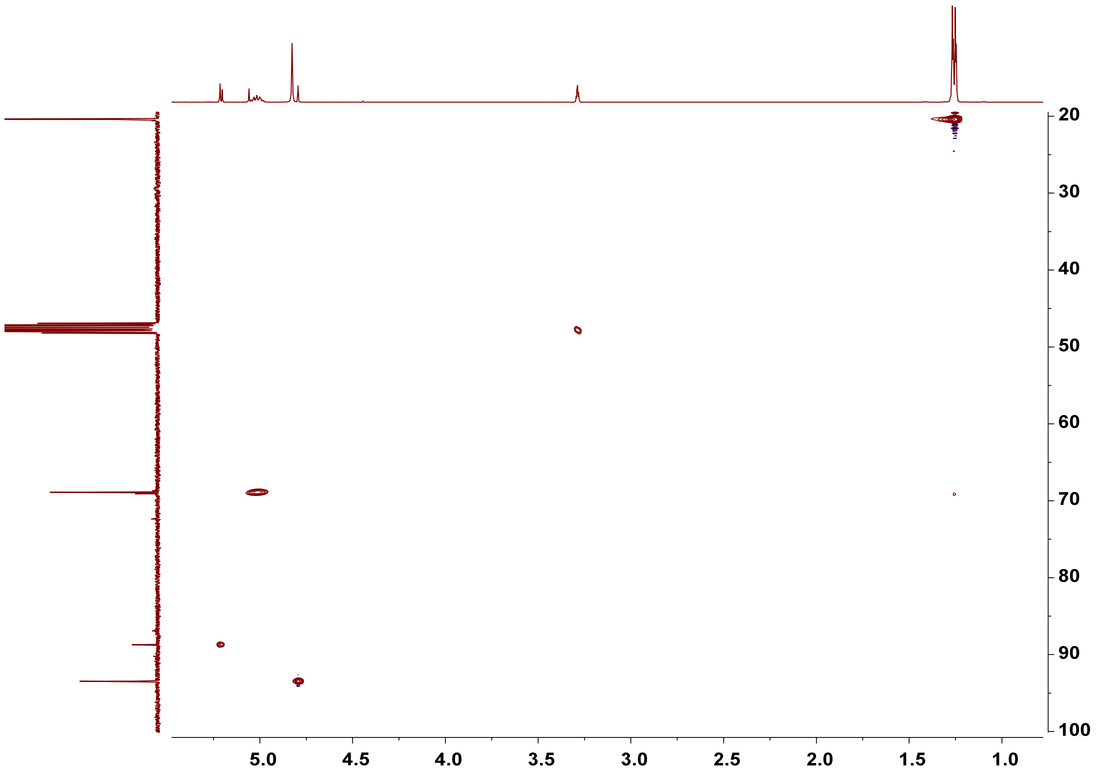

Figure S31. NMR spectra (CD3OD) of **1i**: a) ^{1}H, b) ^{13}C, c) COSY and d) HSQC.

a)

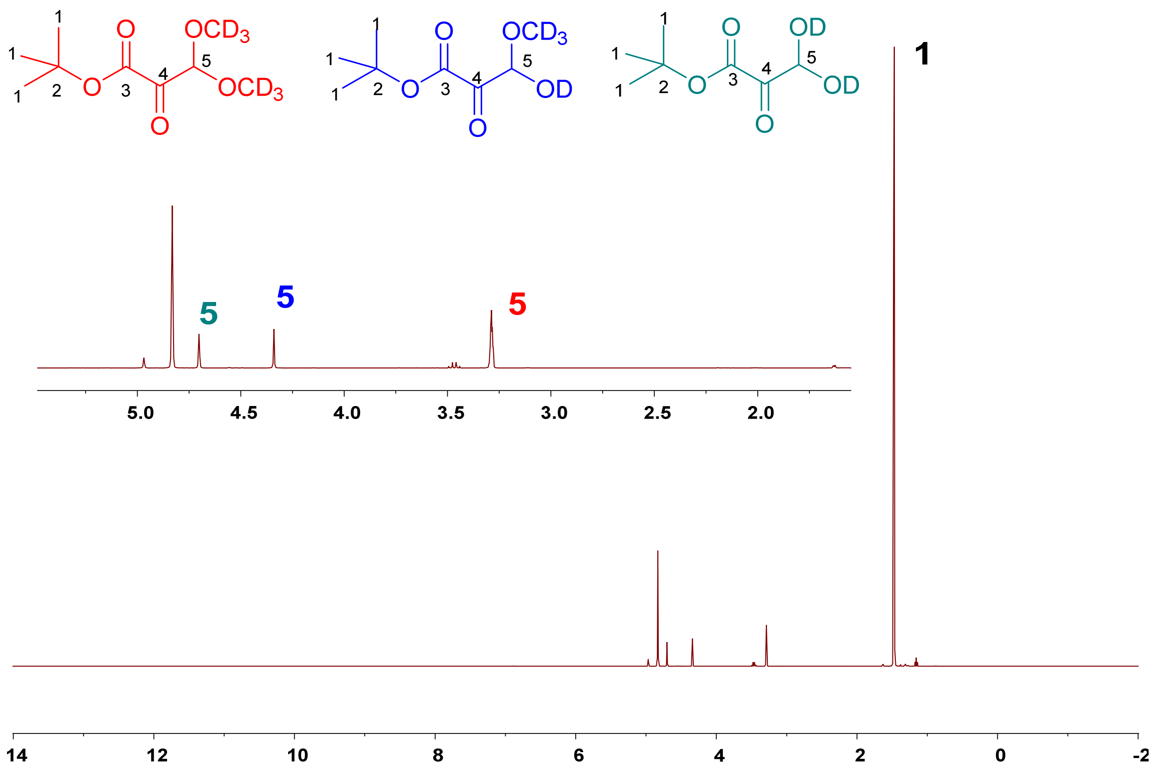
OCD3
OD
5
4
3
2
1
5.0
4.5
4.0
3.5
3.0
2.5
2.0
14
12
10
8
6
4
2
0
-2

b)

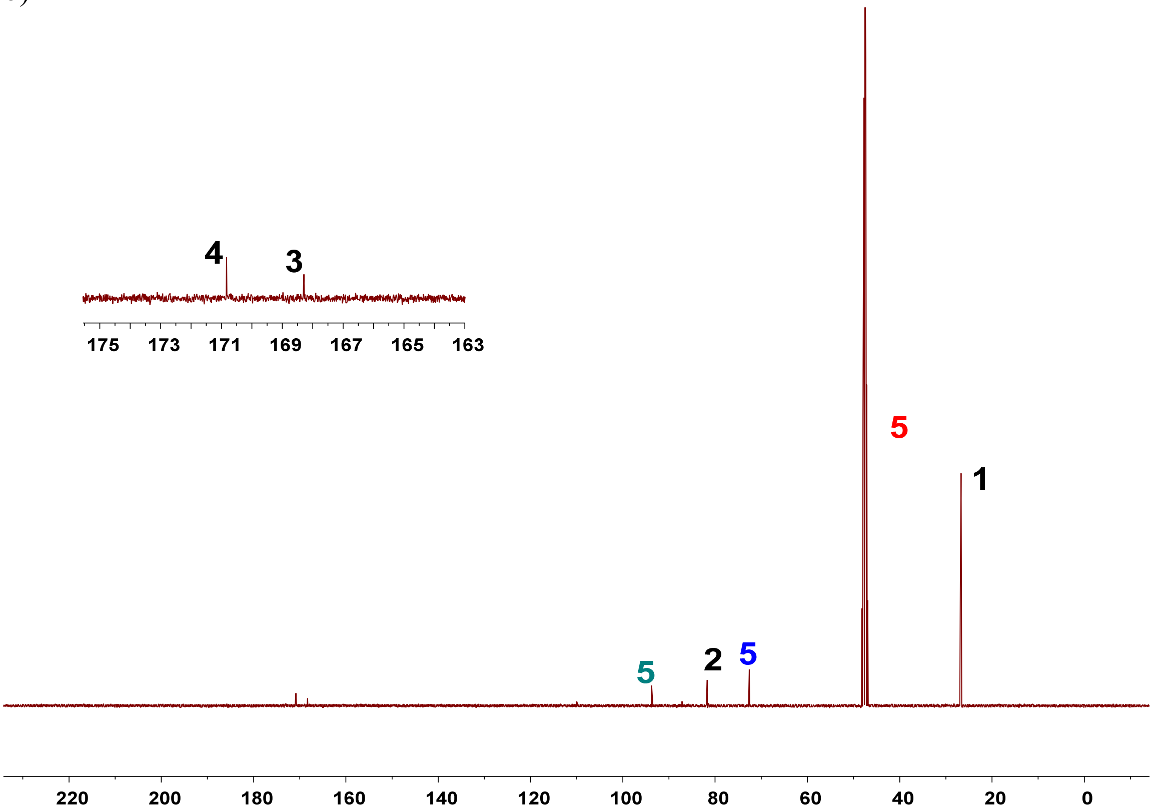
4
3
175
173
171
169
167
165
163
5
2
5
5
1
220
200
180
160
140
120
100
80
60
40
20
0

c)

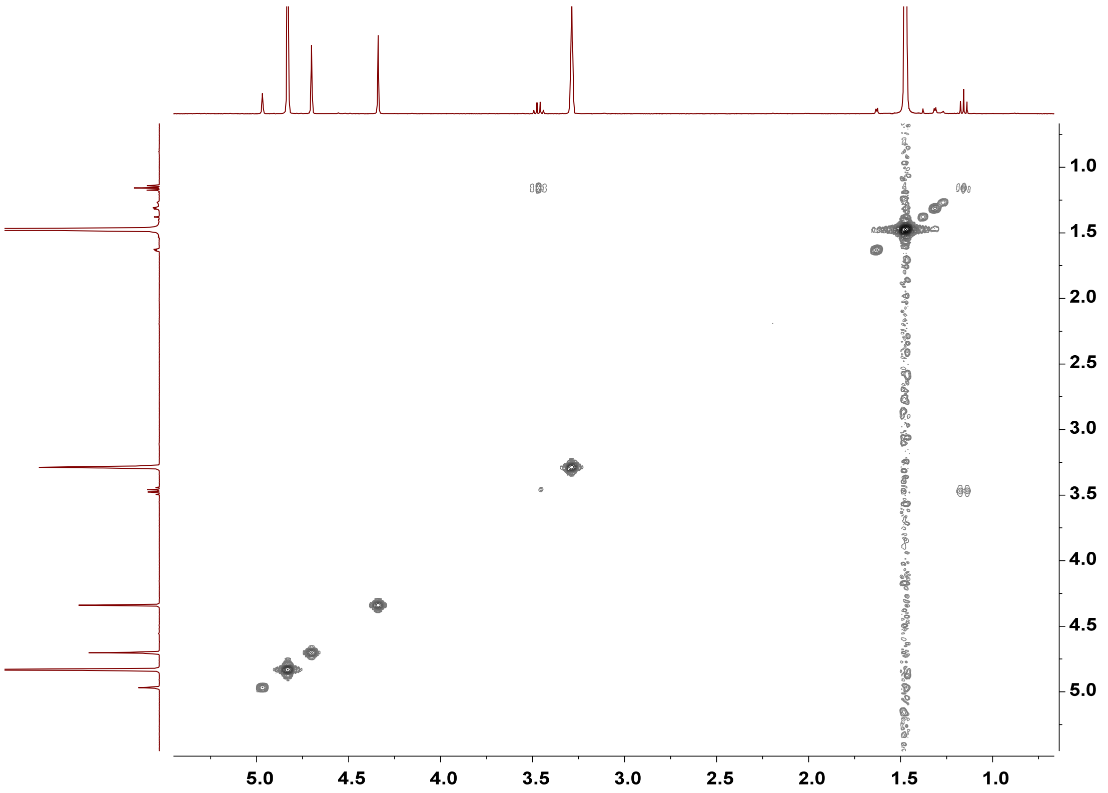

d)

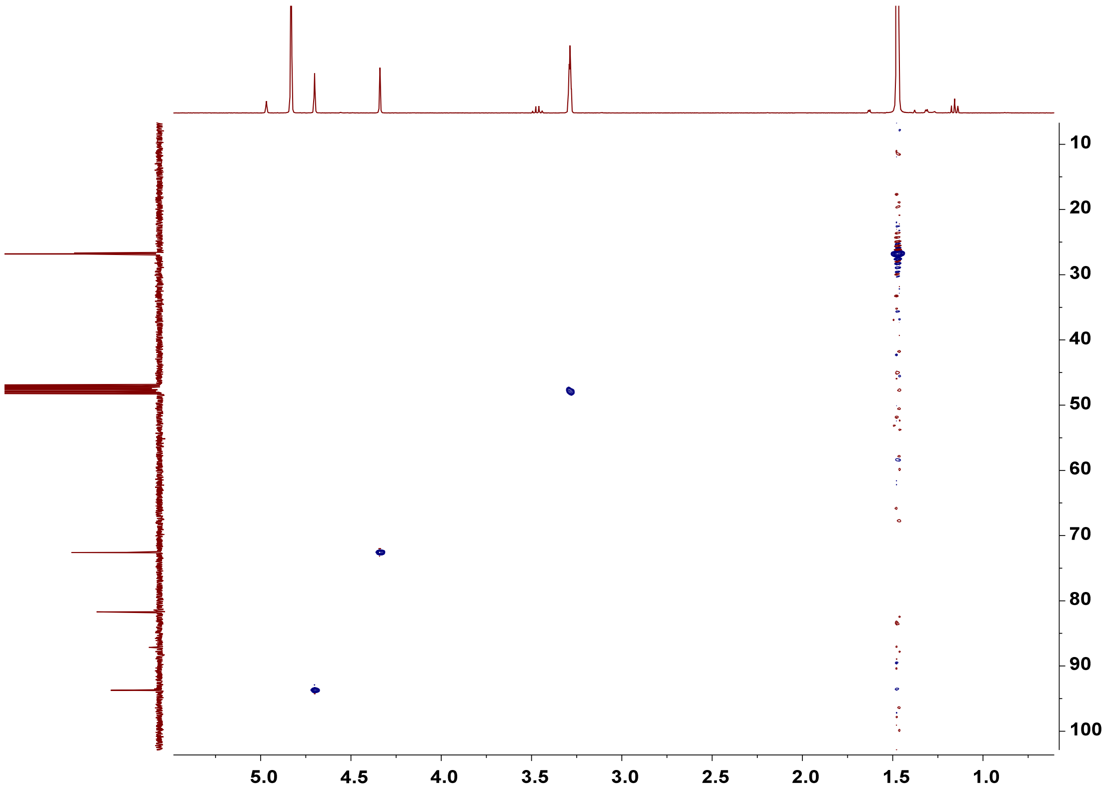

Figure S32. NMR spectra (CD_3OD) of **1j**: a) ^1H, b) ^{13}C, c) COSY and d) HSQC.

a)

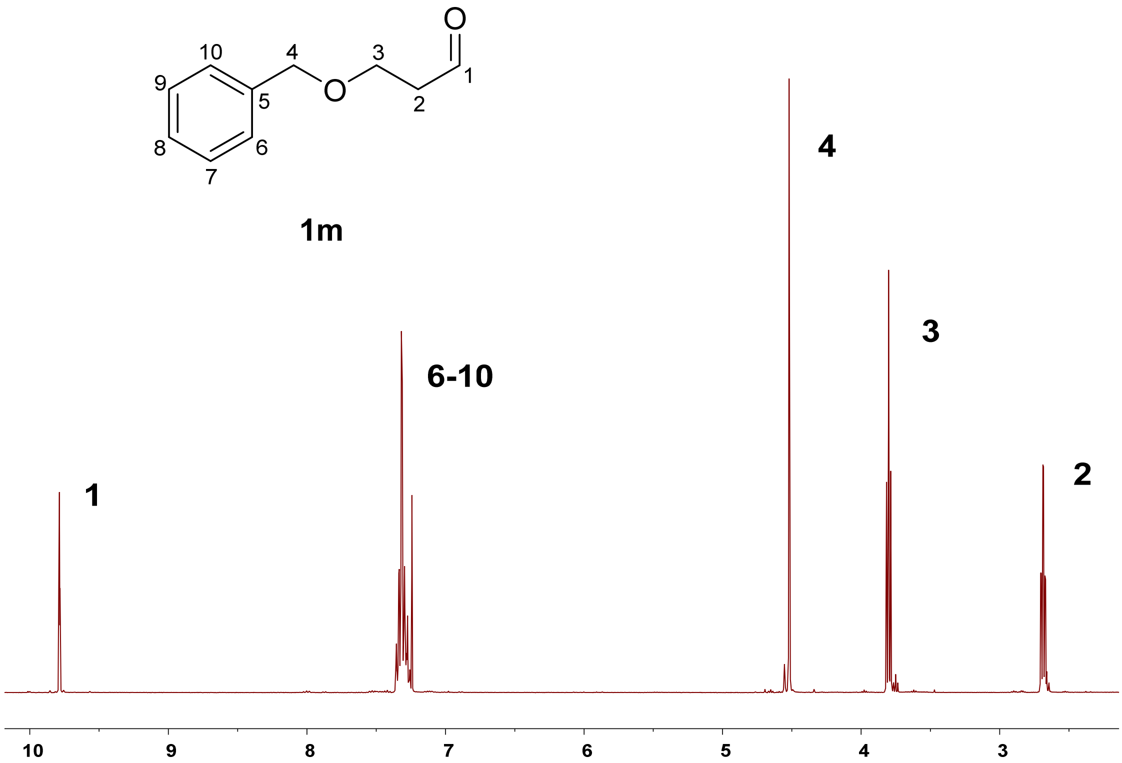

b)

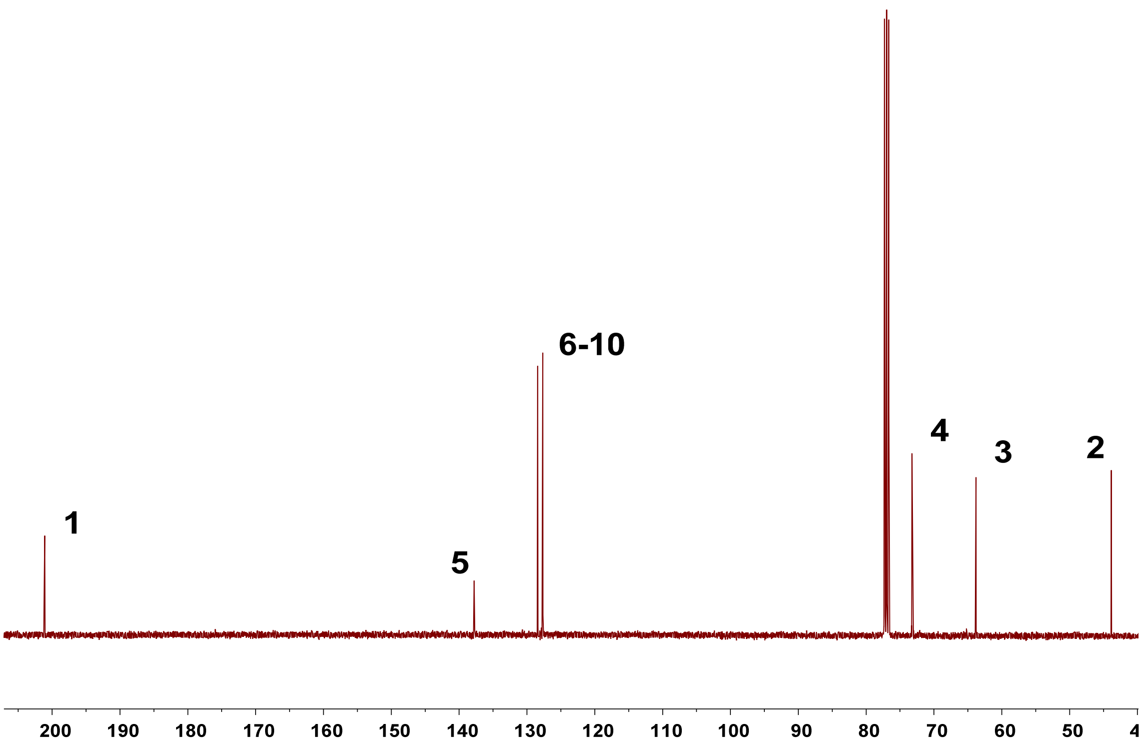

Figure S33. NMR spectra (CD_3OD) of **1m**: a) ^{1}H, b) ^{13}C.

a)

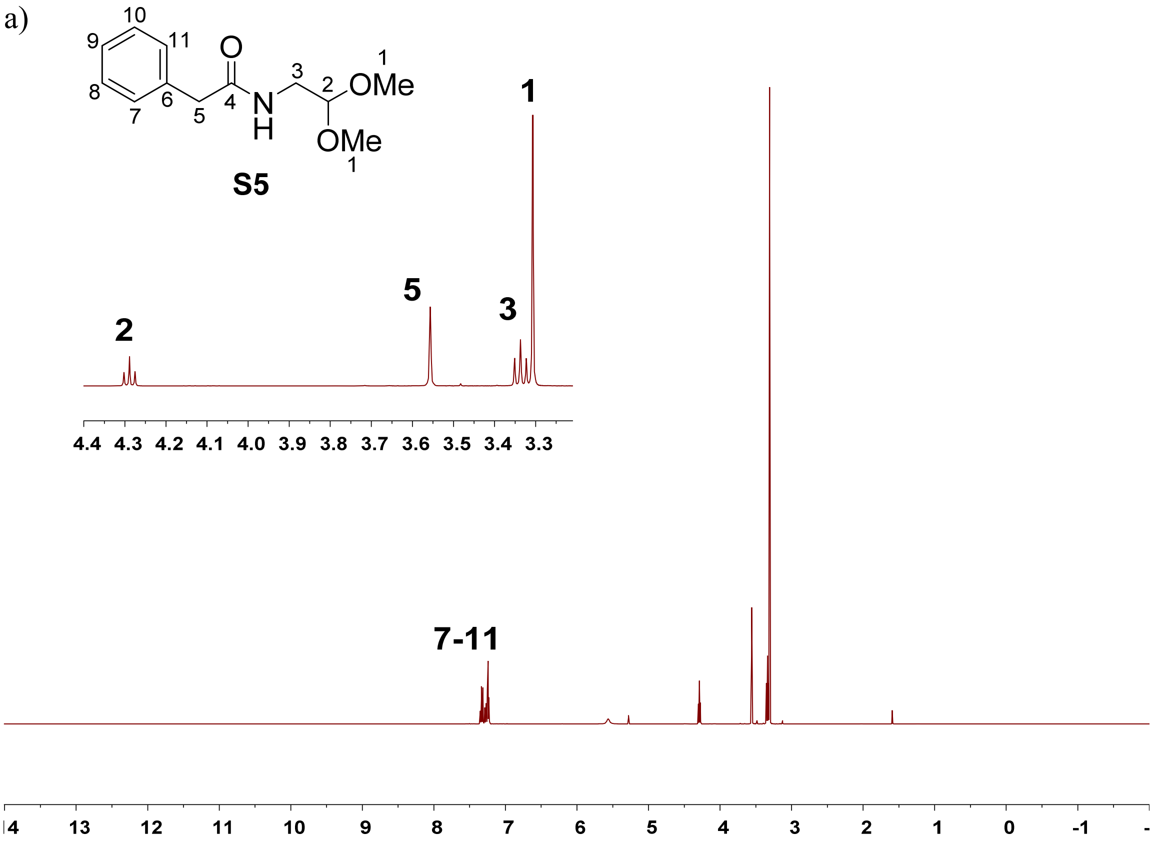

b)

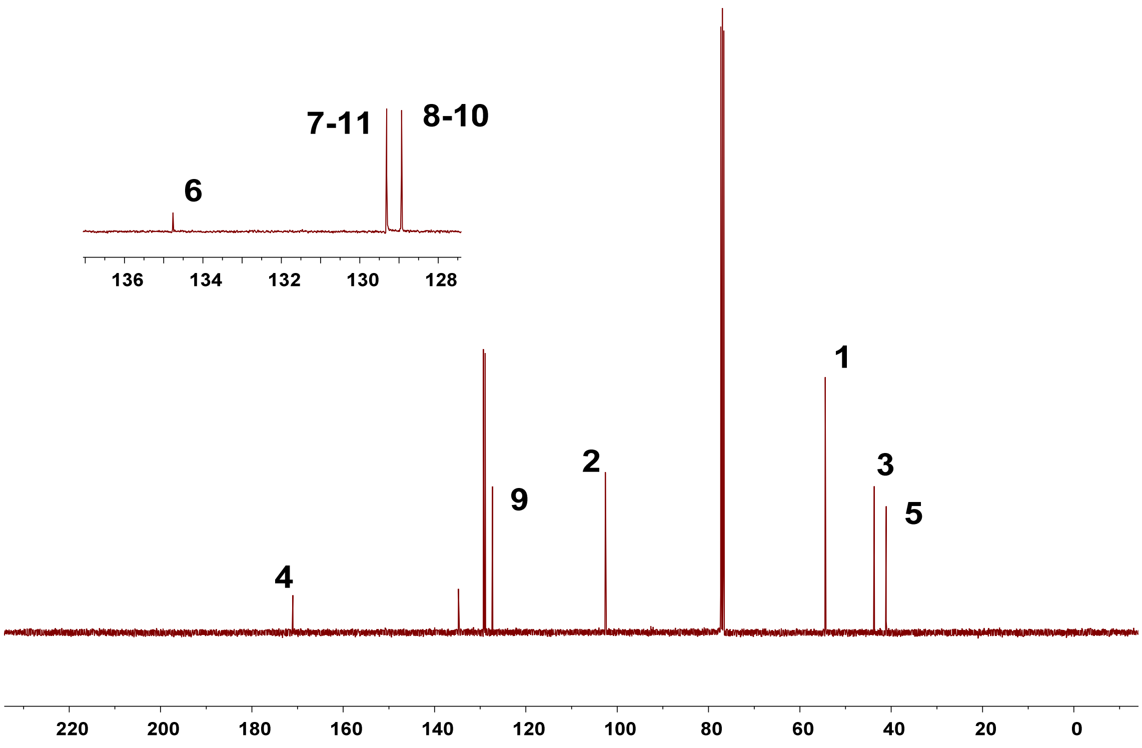

Figure S34. NMR spectra ($CDCl_3$) of **S5**: a) ^1H, b) ^{13}C.

a)

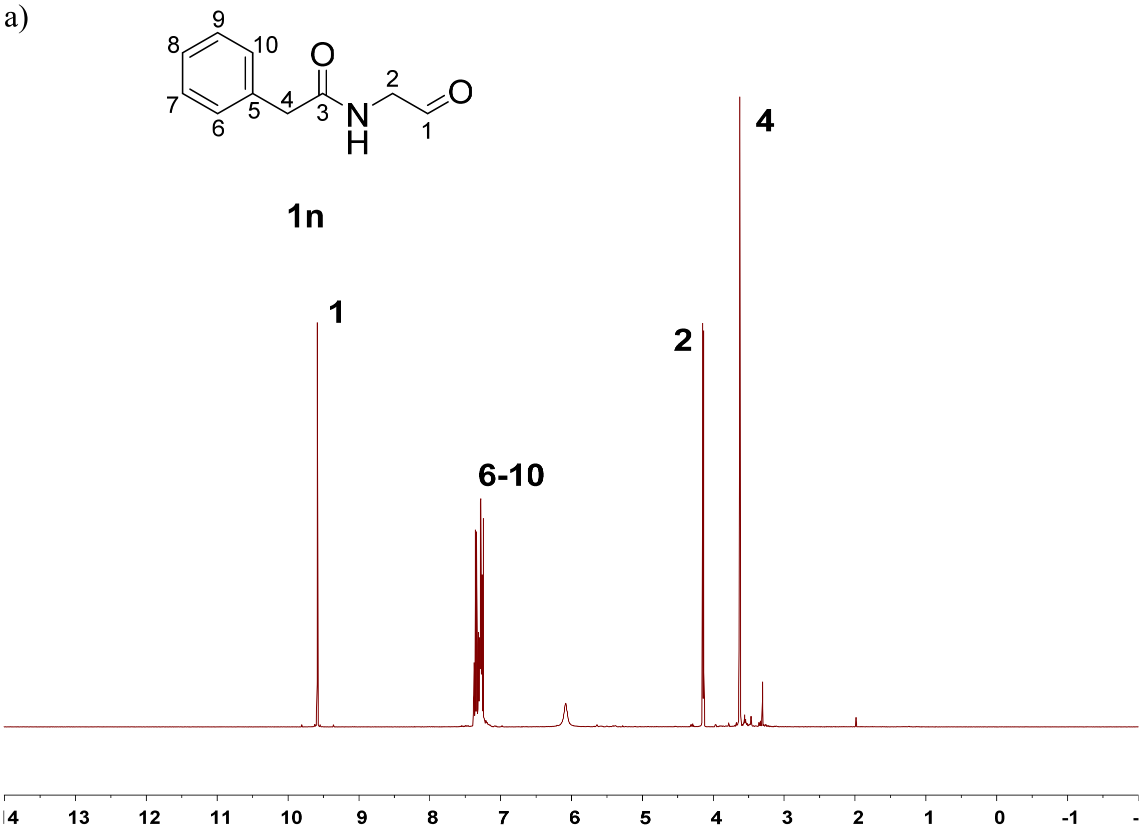

b)

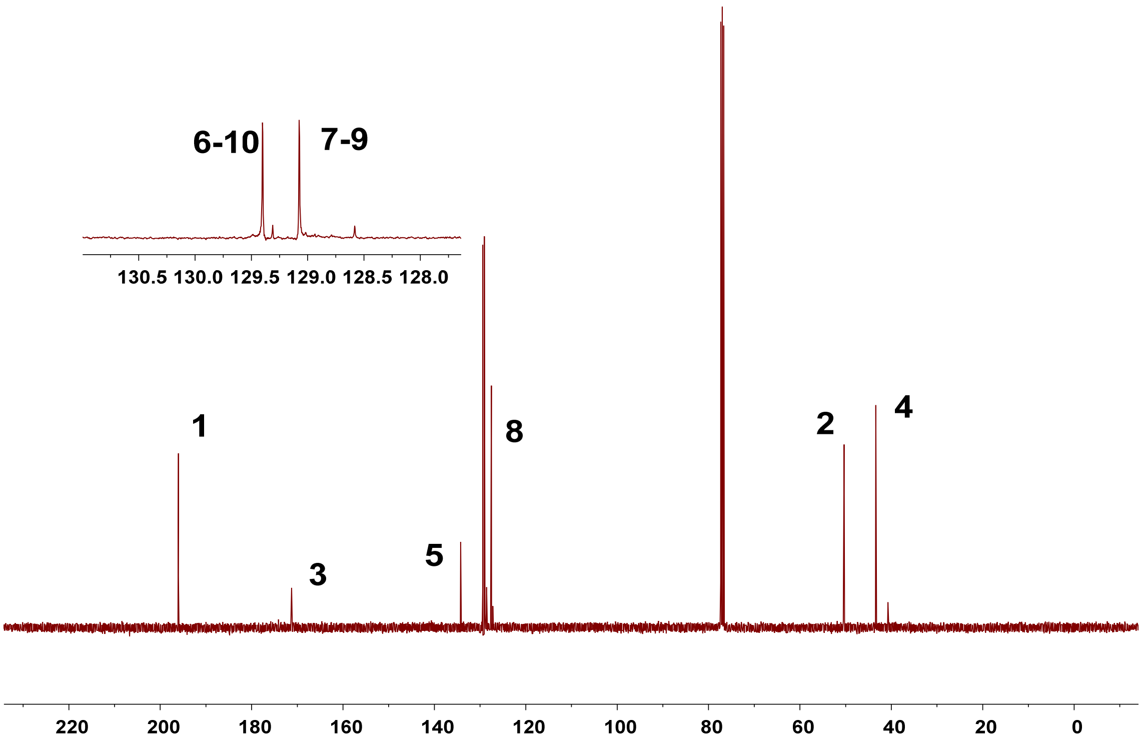

Figure S35. NMR spectra ($CDCl_3$) of **1n**: a) ^{1}H, b) ^{13}C.

a)

b)

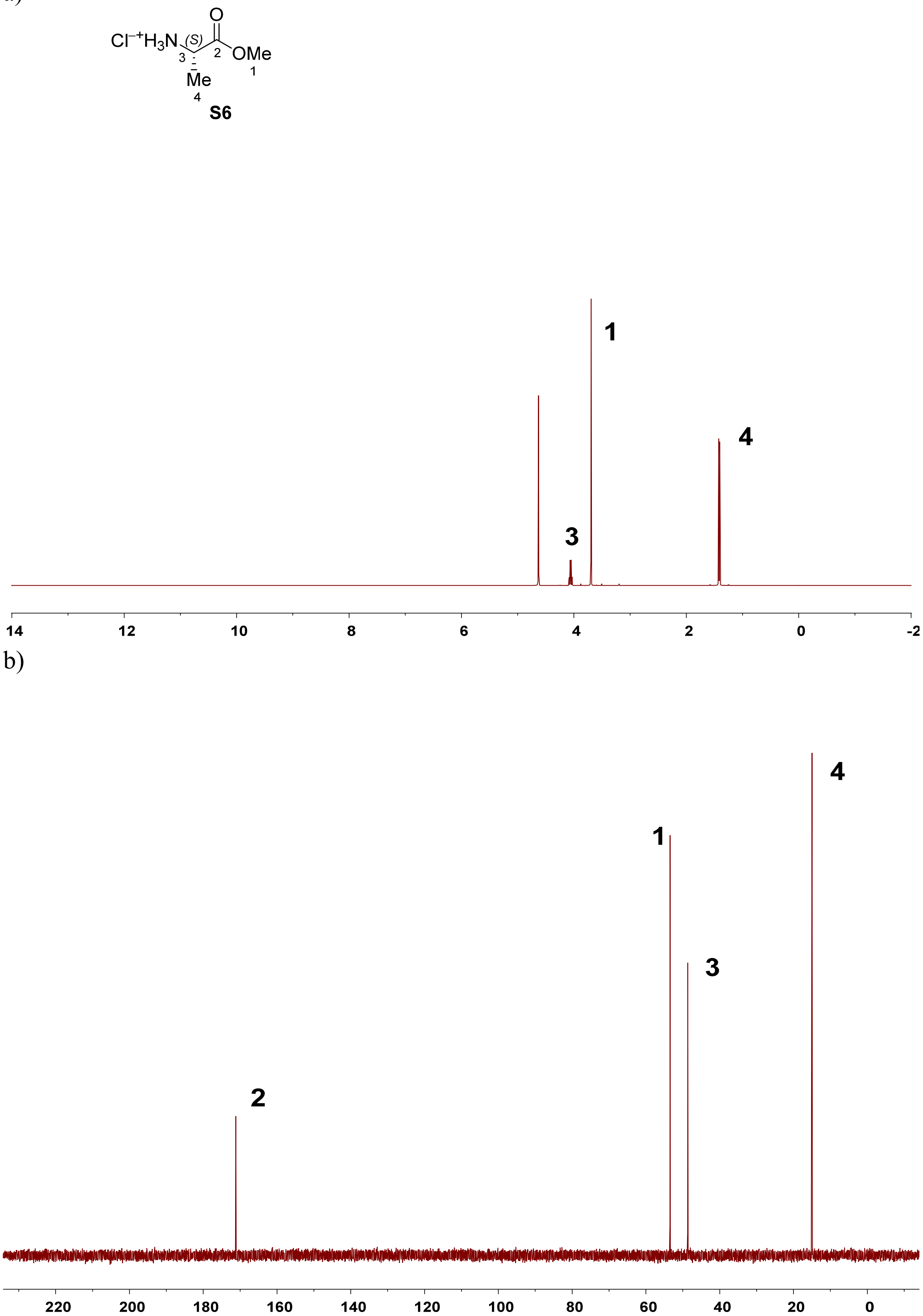

Figure S36. NMR spectra (D_2O) of **S6**: a) ^1H, b) ^{13}C.

a)

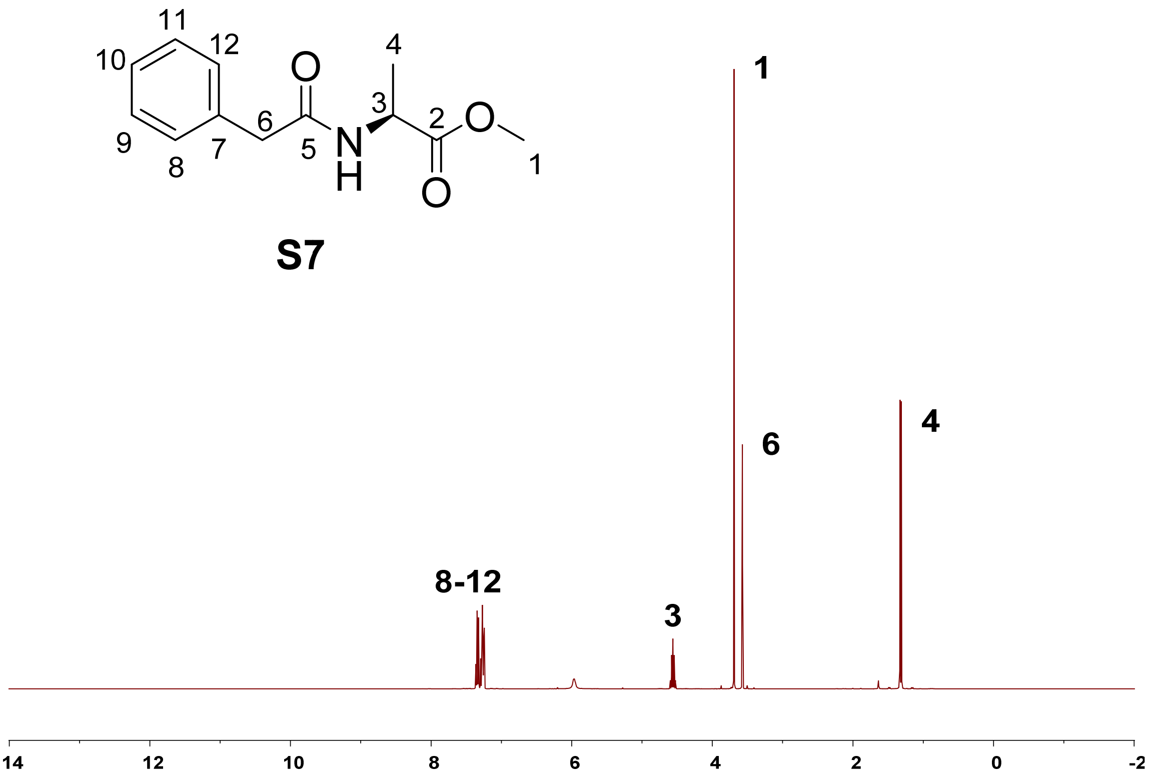

b)

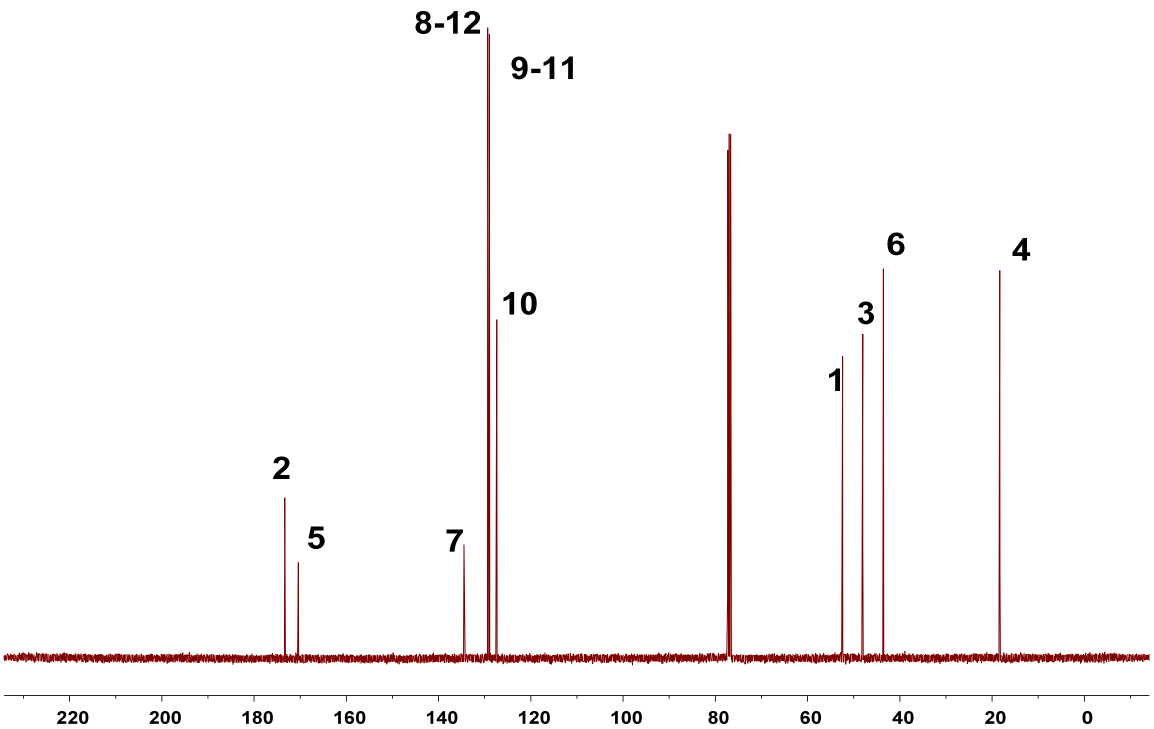

Figure S37. NMR spectra ($CDCl_3$) of **S7**: a) ^1H, b) ^{13}C

a)

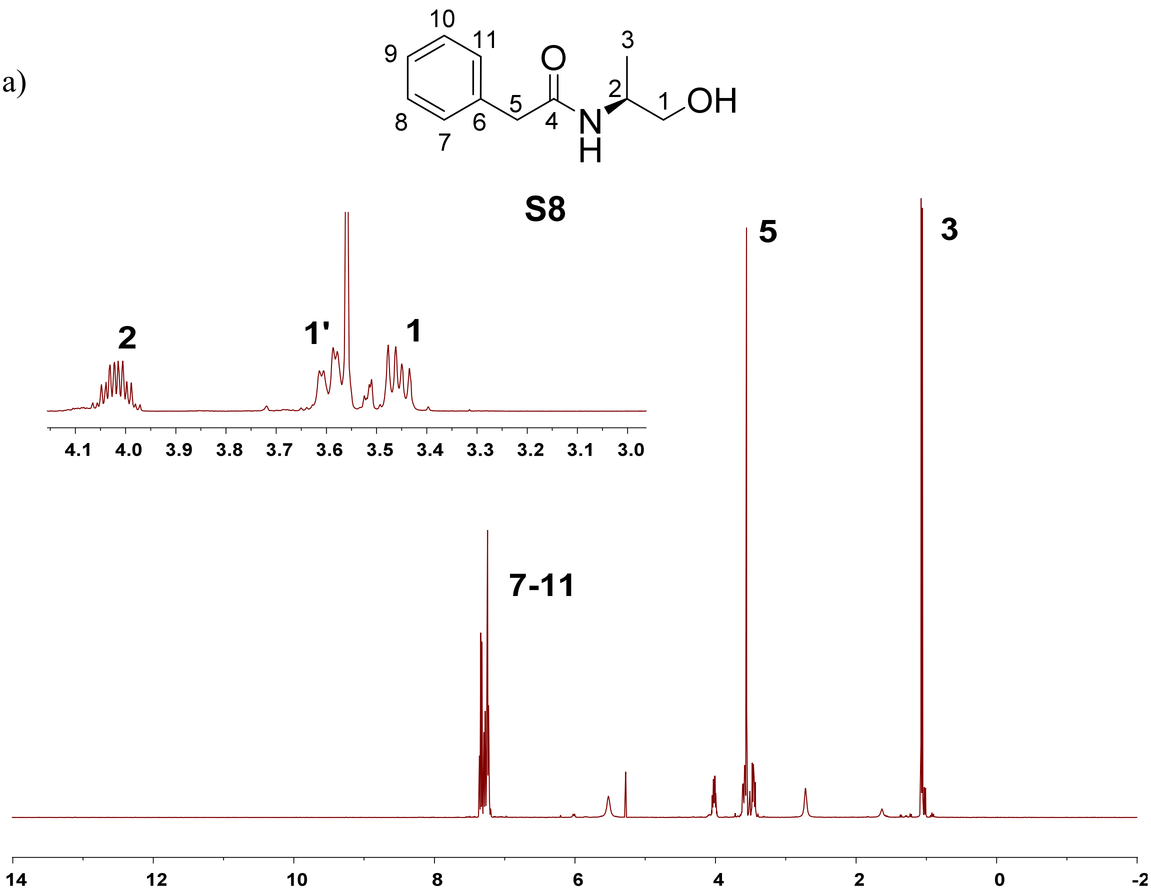

b)

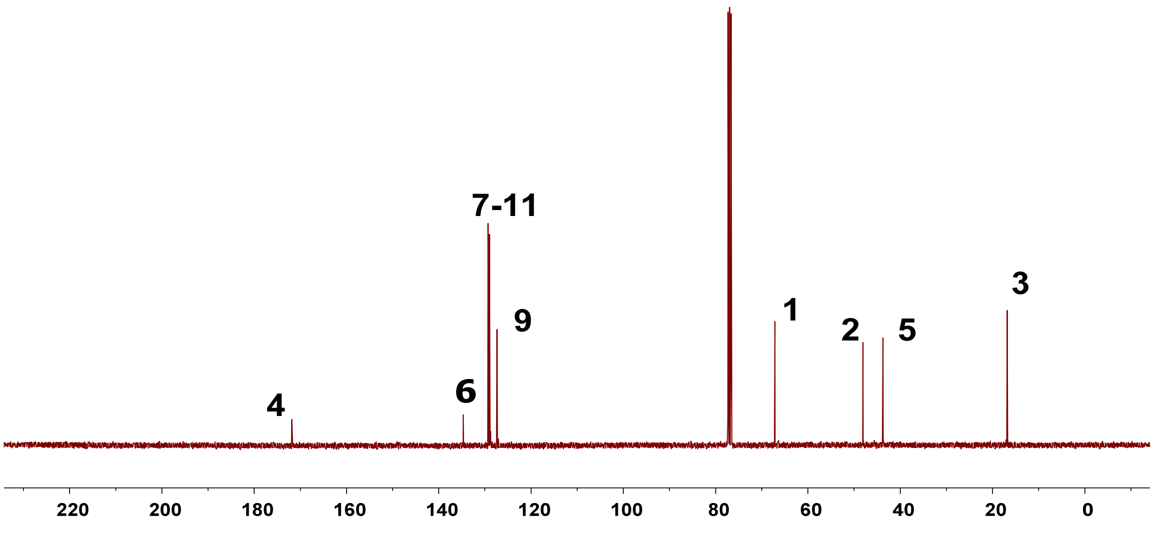

Figure S38. NMR spectra ($CDCl_3$) of **S8**: a) ^{1}H, b) ^{13}C

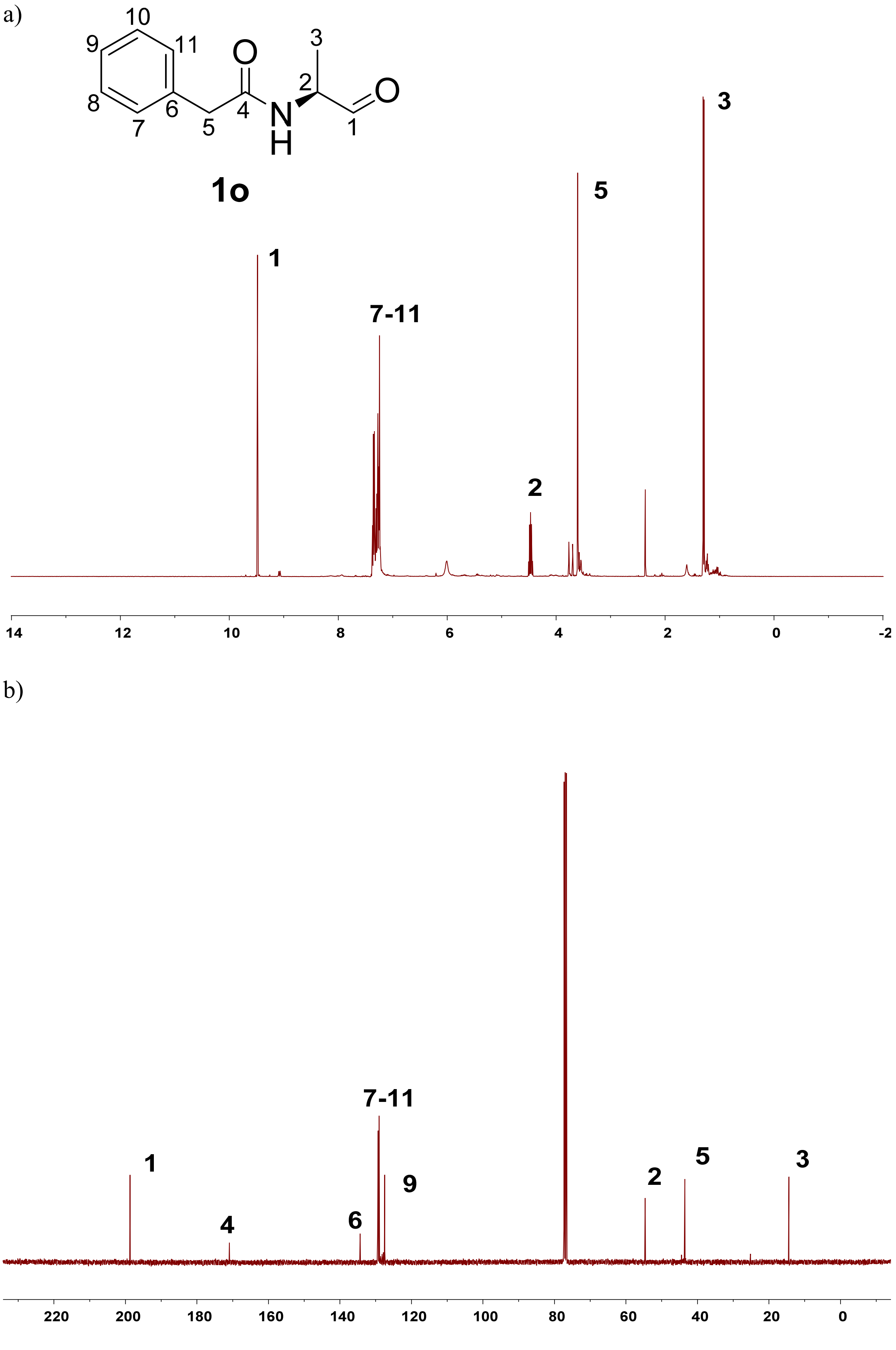

Figure S39. NMR spectra ($CDCl_3$) of **1o**: a) ^{1}H, b) ^{13}C.

a)

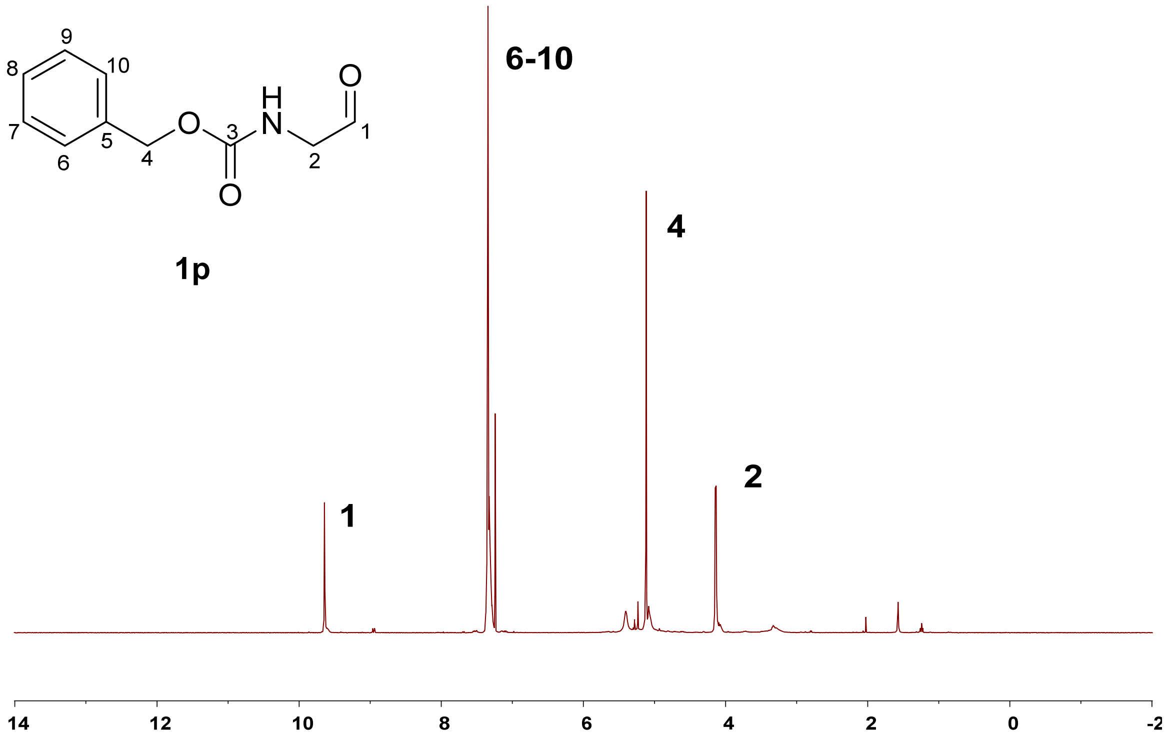

b)

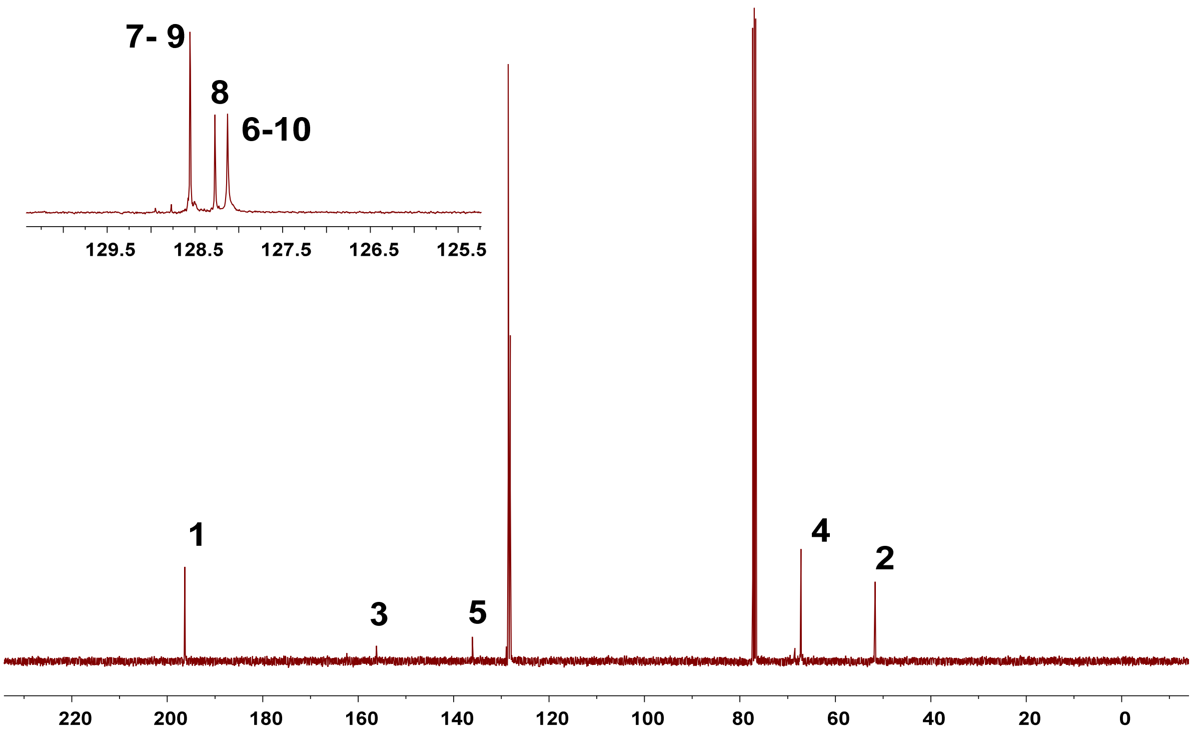

Figure S40. NMR spectra ($CDCl_3$) of **1p**: a) ^{1}H b) ^{13}C.

a)

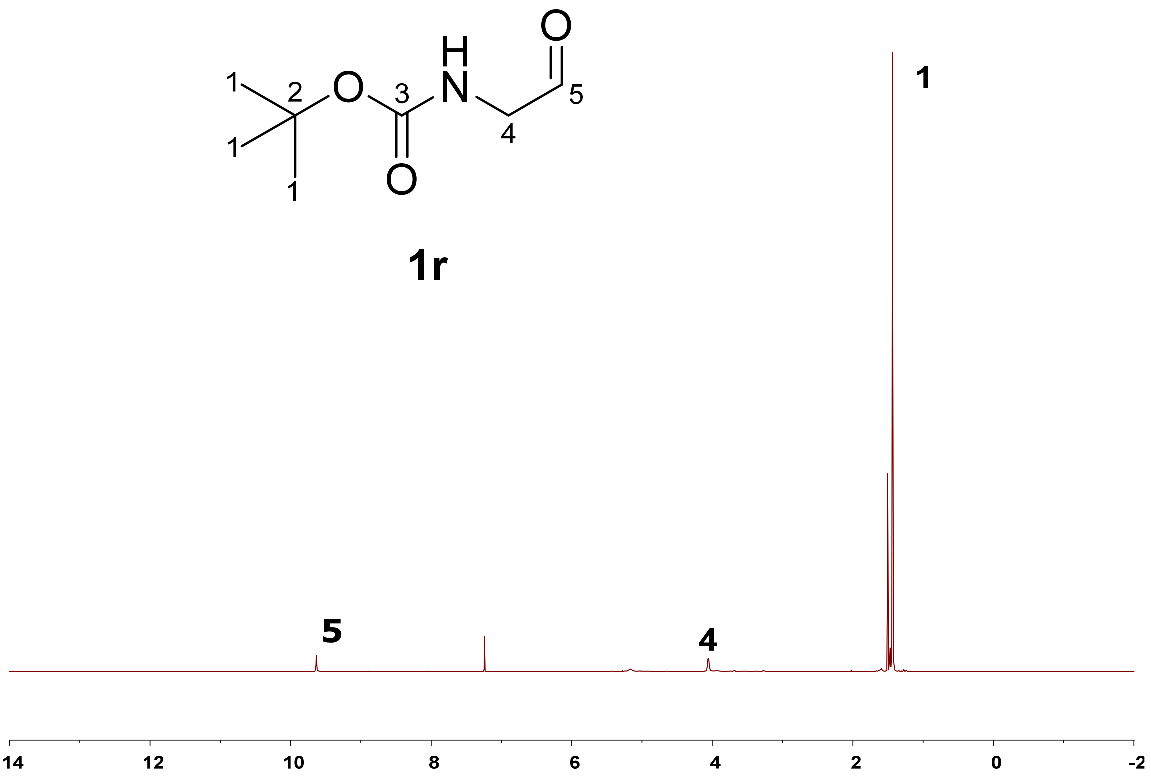

b)

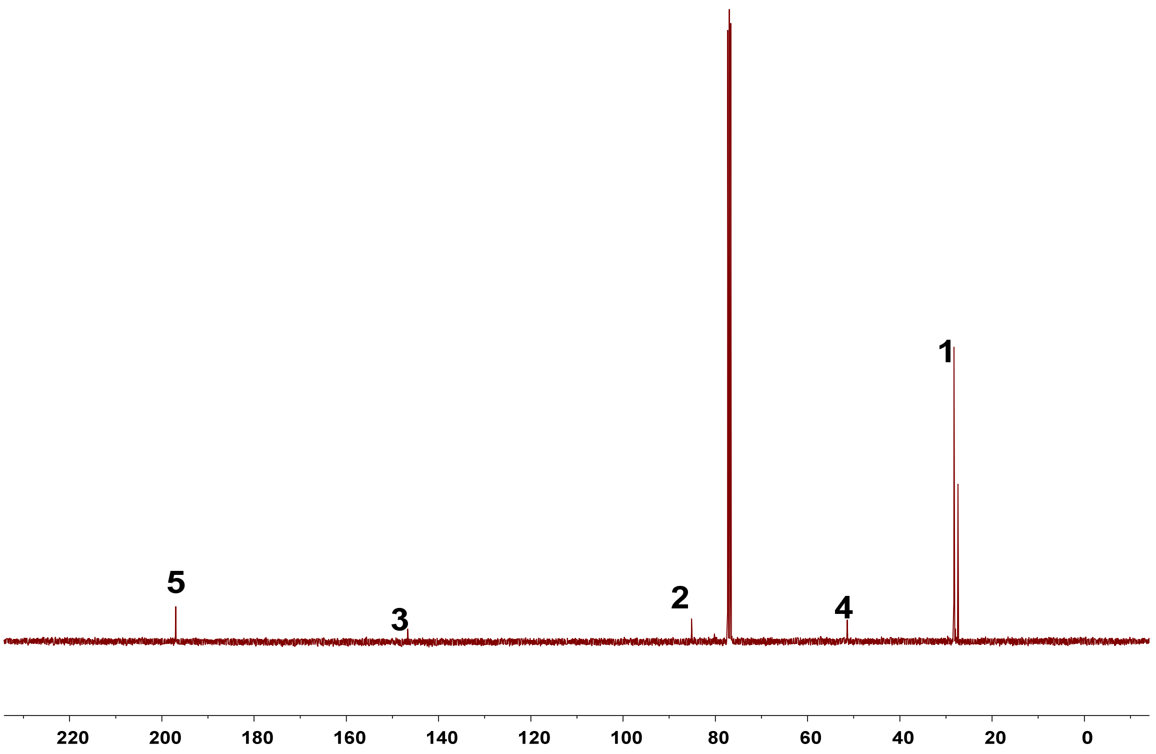

Figure S41. NMR spectra ($CDCl_3$) of **1r**: a) ^1H, b) ^{13}C.

a)

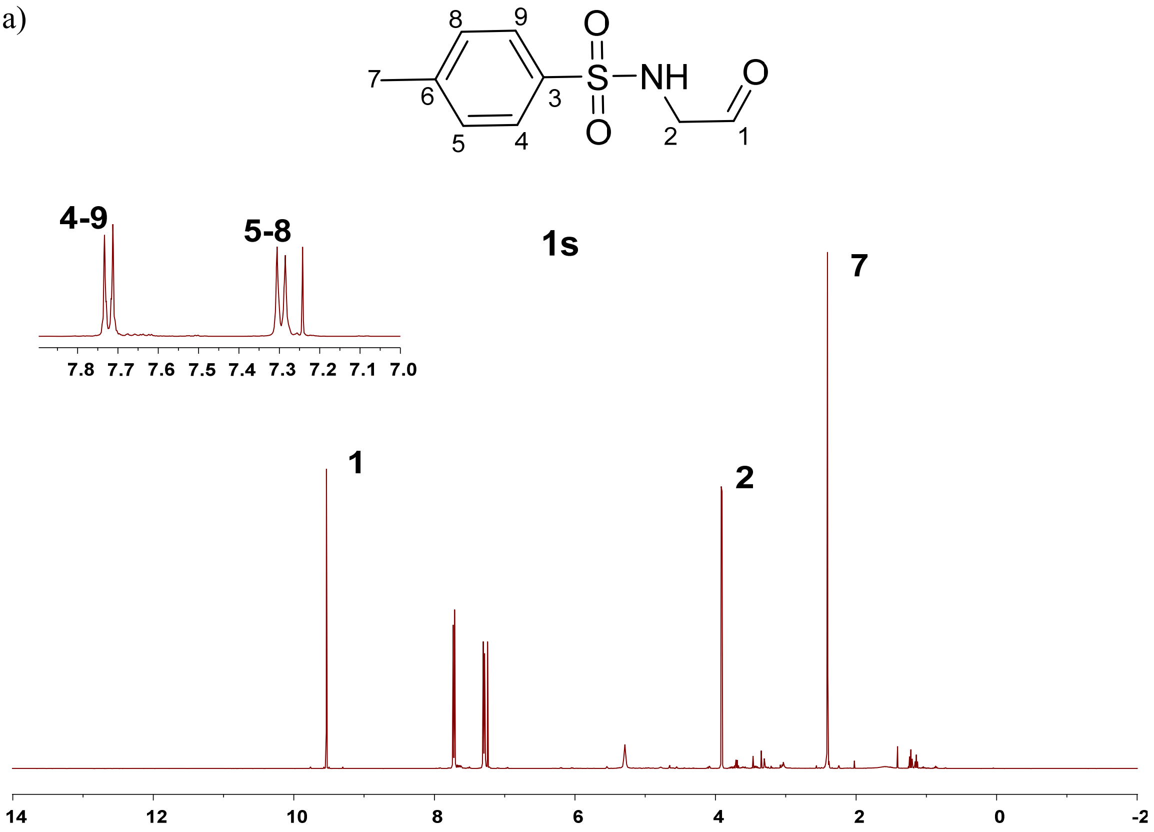

b)

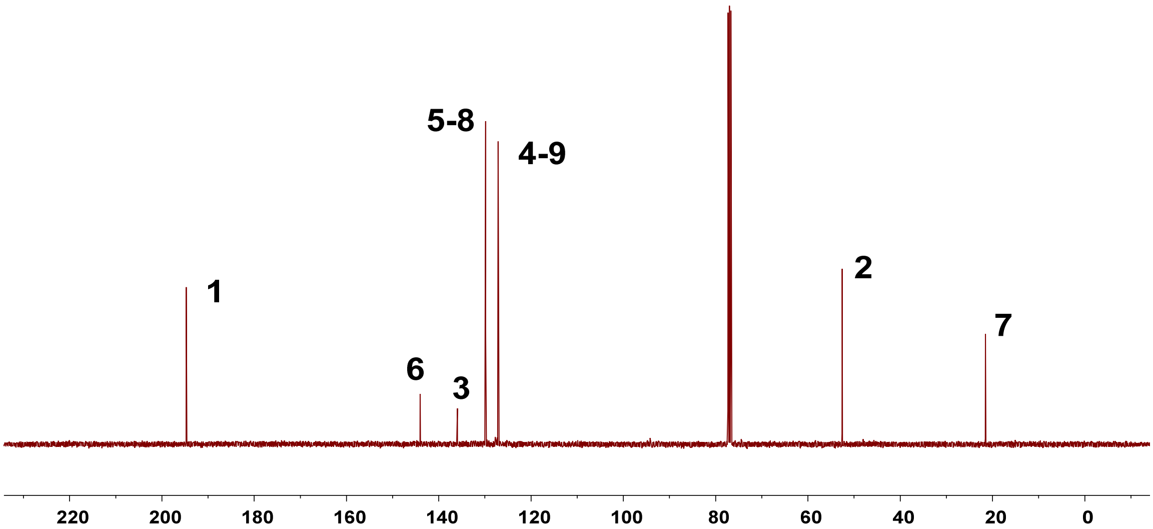

Figure S42. NMR spectra ($CDCl_3$) of **1s**: a) ^1H, b) ^{13}C.

NMR spectra of 4-hydroxyesters.

a)

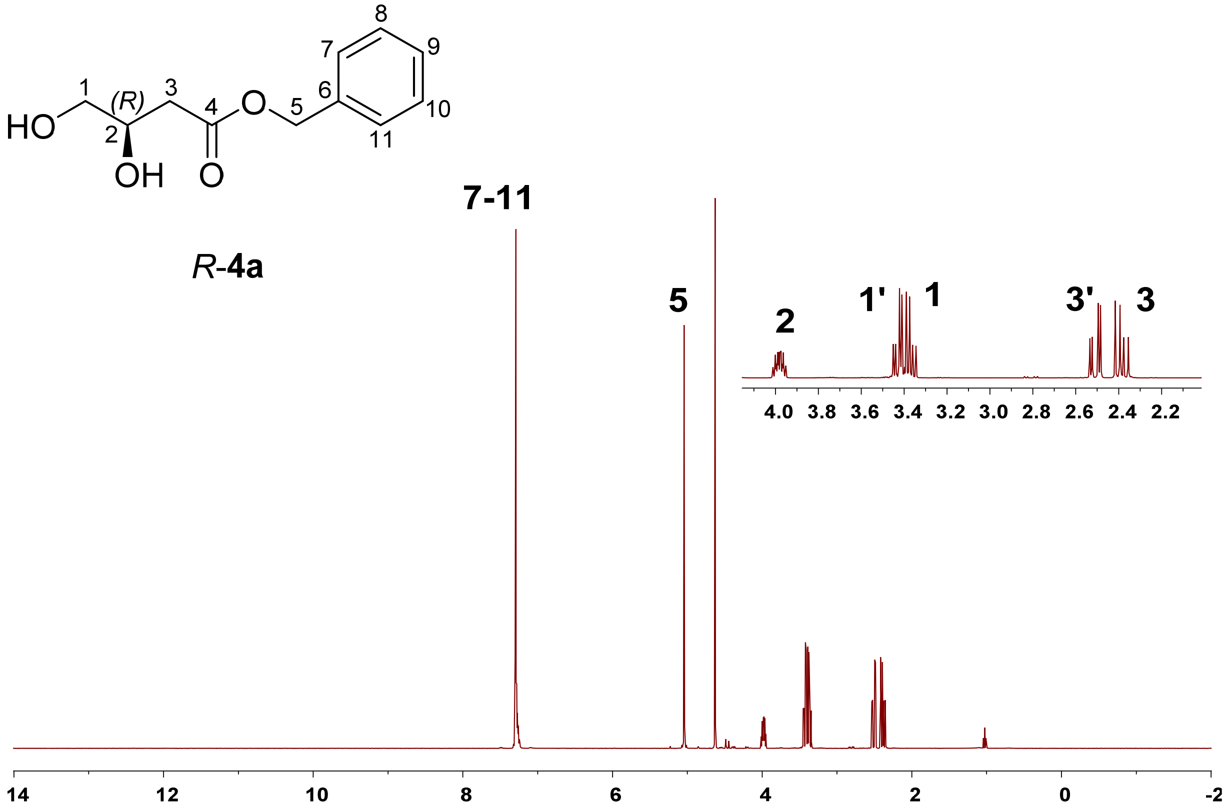

b)

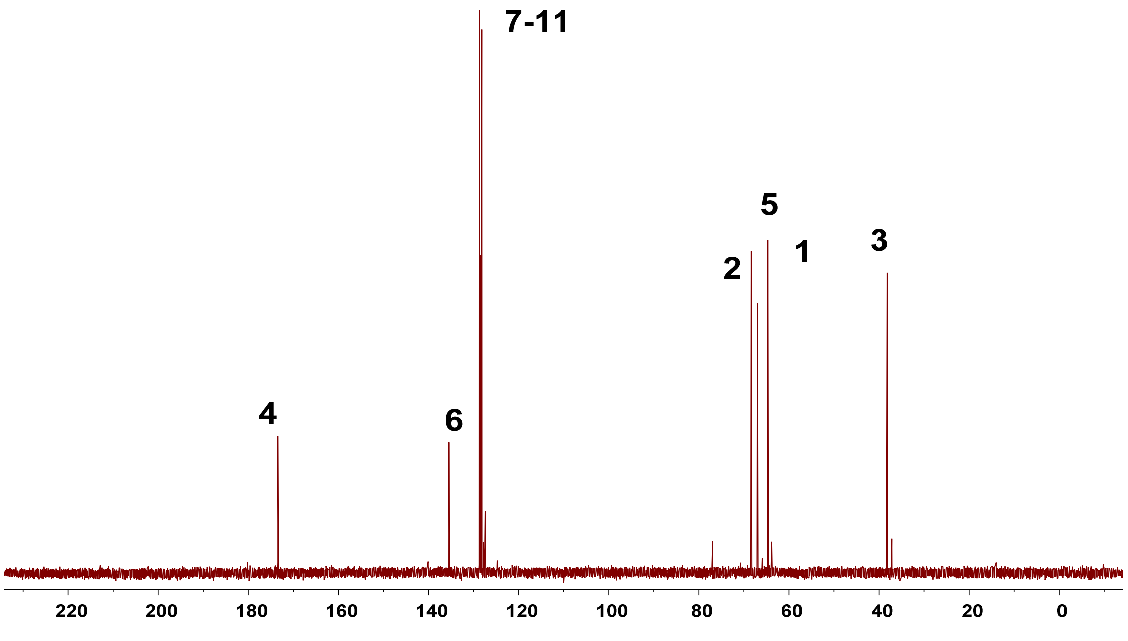

c)

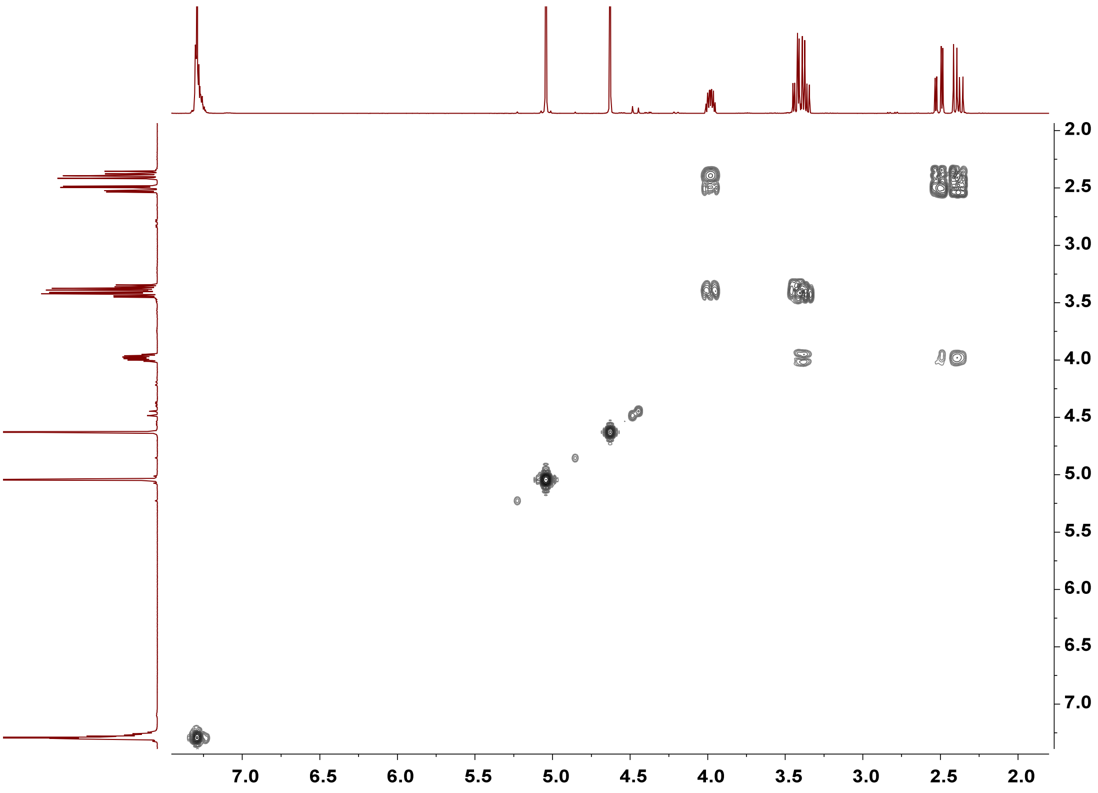

d)

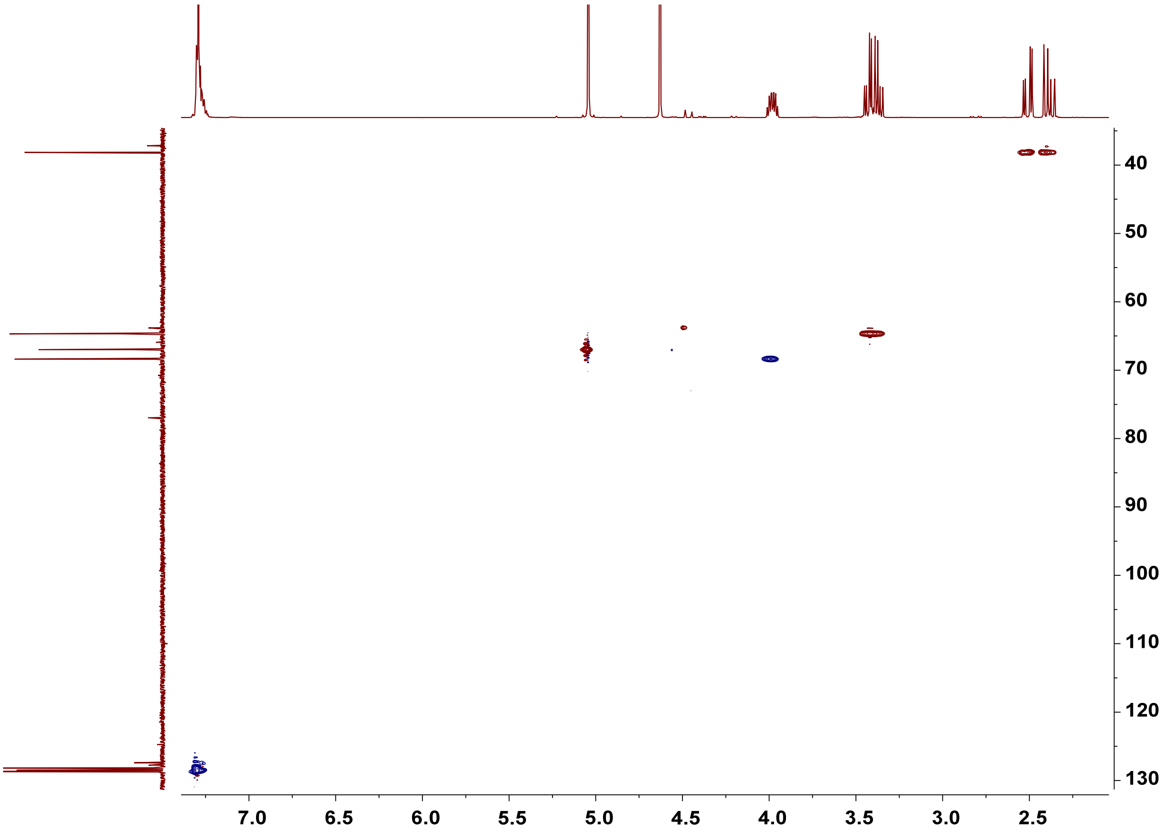

Figure S43. NMR spectra (D_2O) of *R*-**4a**: a) ^{1}H, b) ^{13}C, c) COSY and d) HSQC.

a)

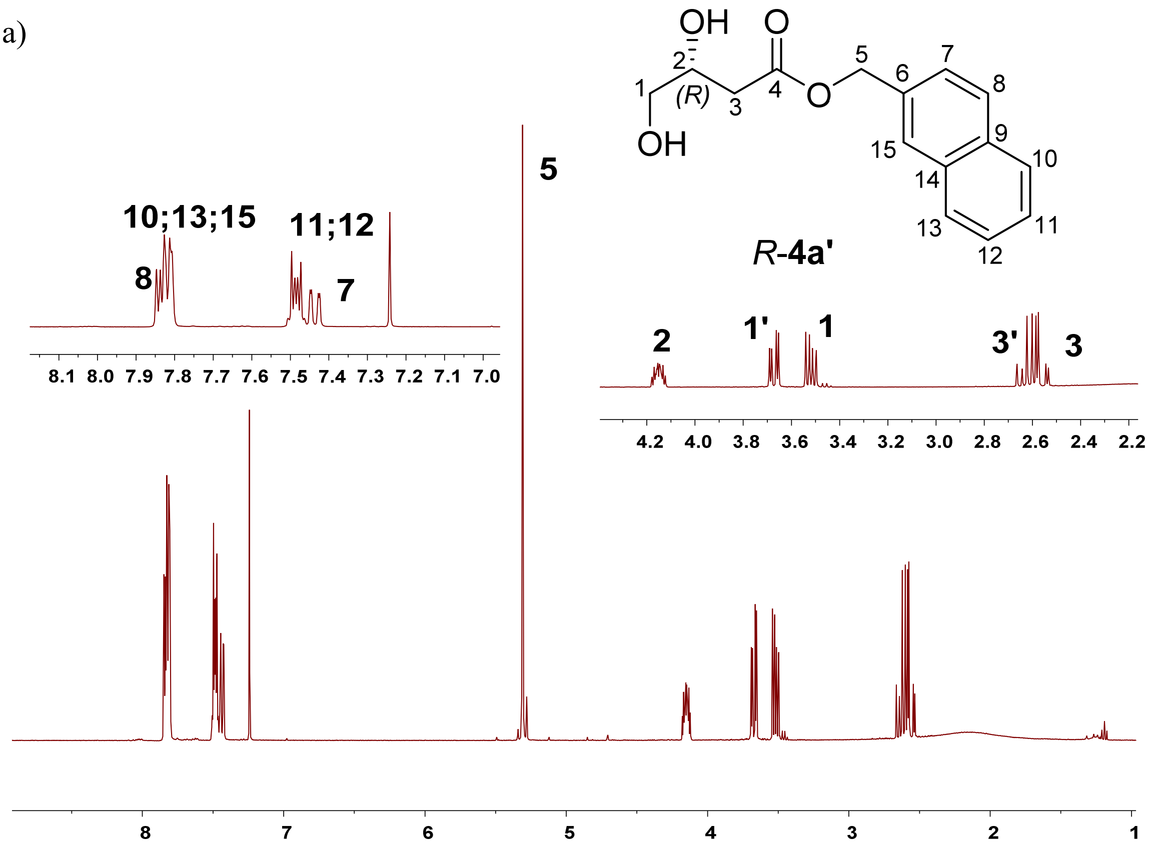
OH
O
OH
1
2
(R)
3
4
5
6
7
8
9
10
11
12
13
14
15
R-4a'
5
10;13;15
11;12
8
7
8.1 8.0 7.9 7.8 7.7 7.6 7.5 7.4 7.3 7.2 7.1 7.0
2
1'
1
3'
3
4.2 4.0 3.8 3.6 3.4 3.2 3.0 2.8 2.6 2.4 2.2
8
7
6
5
4
3
2
1

b)

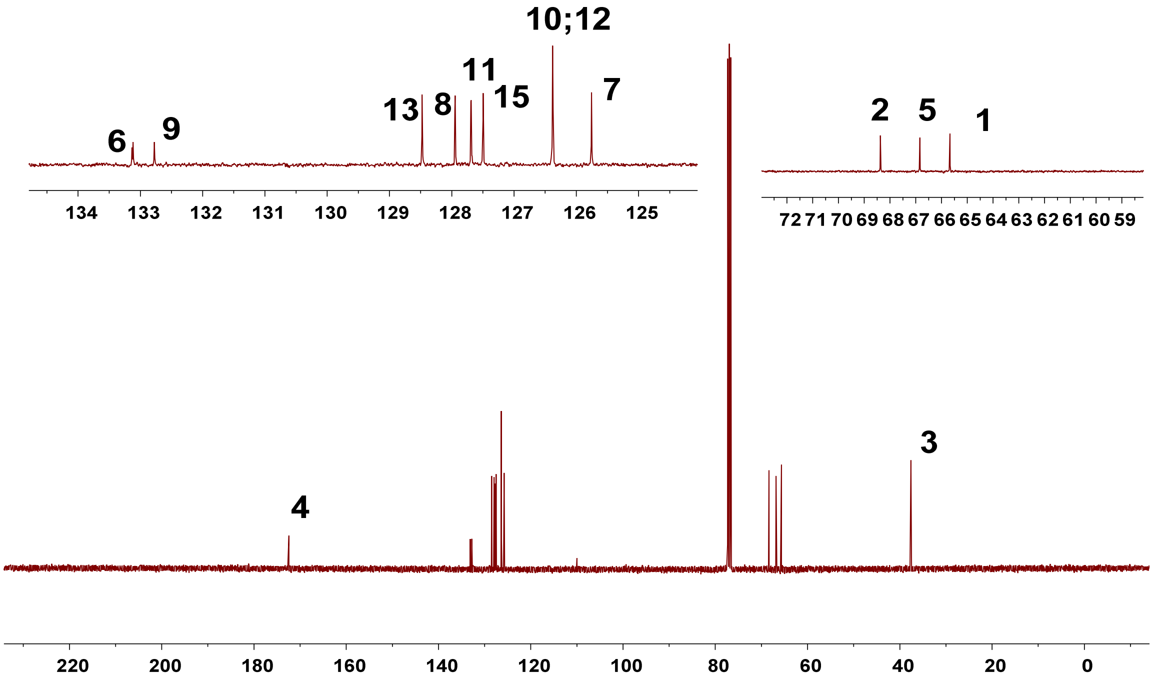
10;12
11
13
8
15
7
6
9
134 133 132 131 130 129 128 127 126 125
2
5
1
72 71 70 69 68 67 66 65 64 63 62 61 60 59
4
3
220 200 180 160 140 120 100 80 60 40 20 0

c)

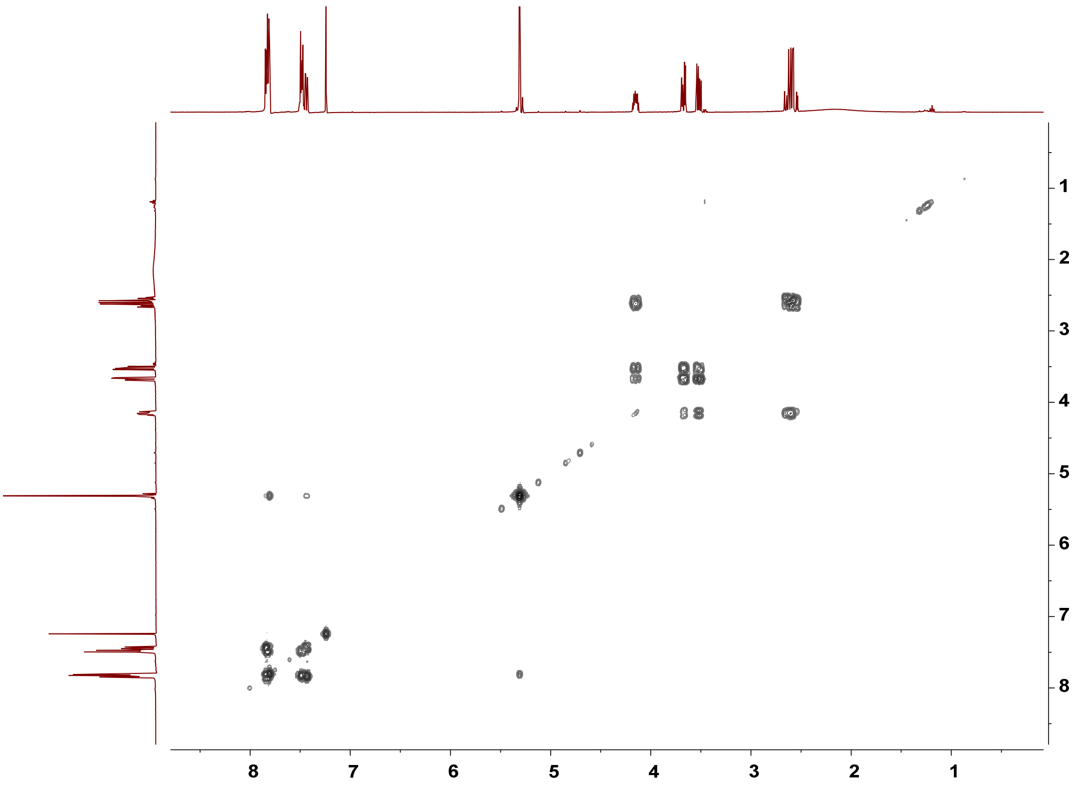

d)

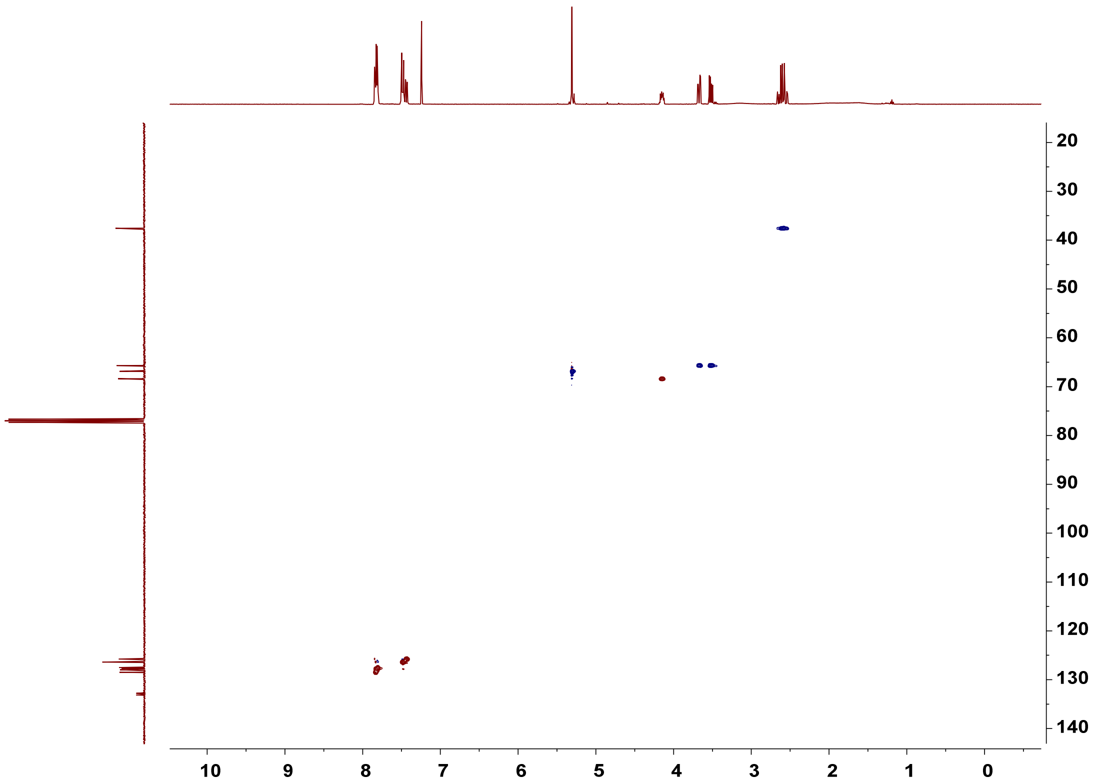

Figure S44. NMR spectra ($CDCl_3$) of *R*-**4a'**: a) ^{1}H, b) ^{13}C, c) COSY and d) HSQC.

a)

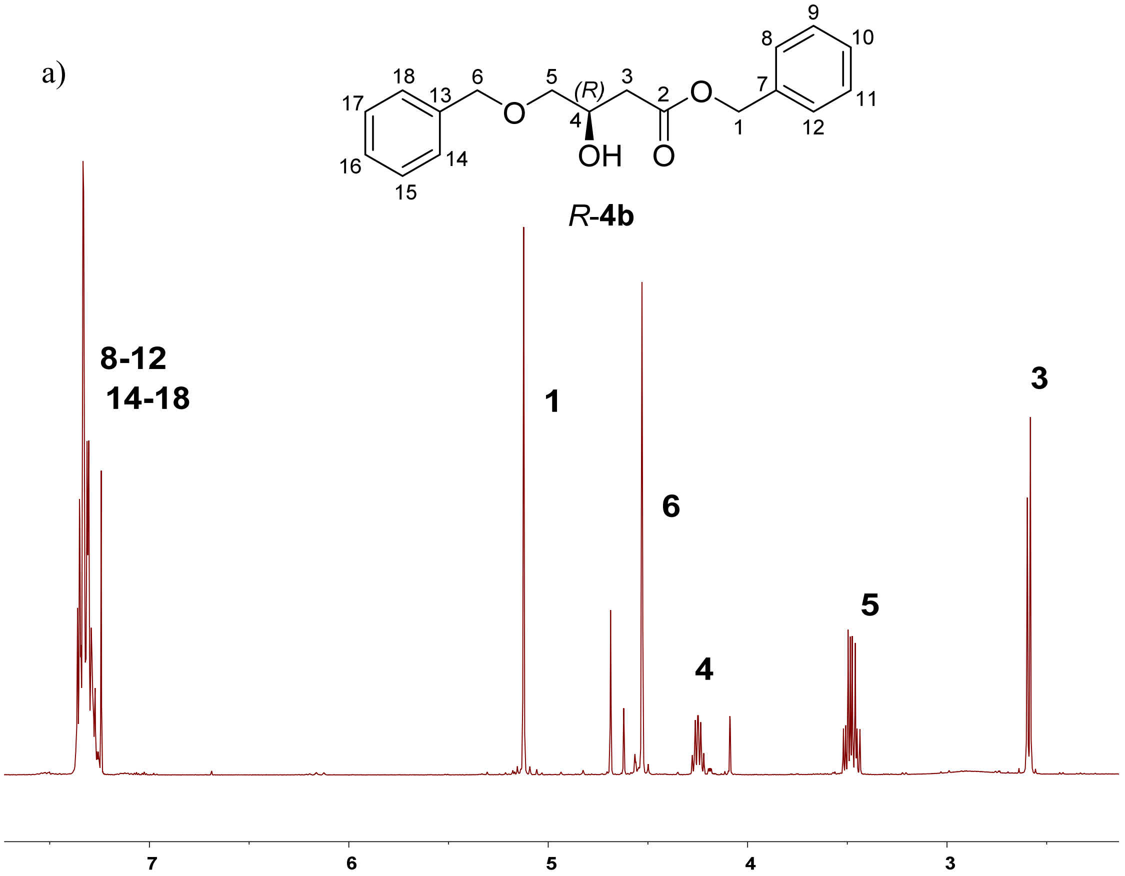

R-4b
OH
O
8-12
14-18
1
6
4
5
3
7
6
5
4
3

b)

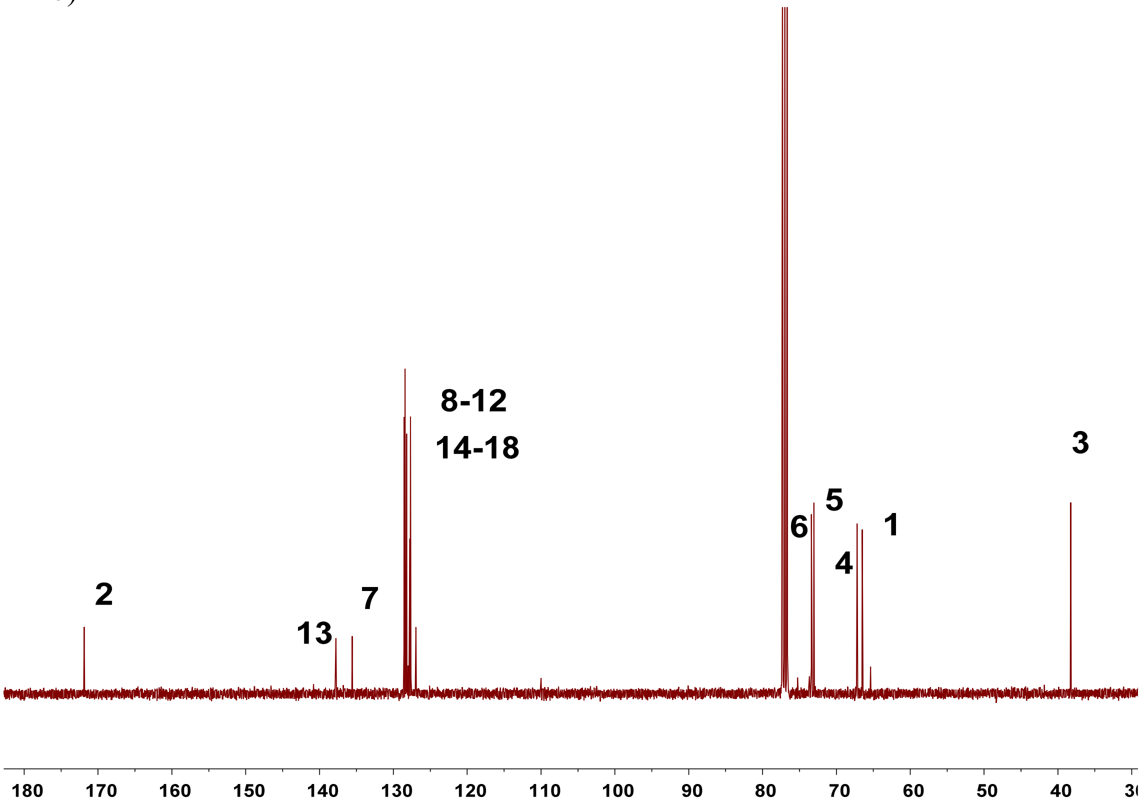

8-12
14-18
2
13
7
6
5
4
1
3
180
170
160
150
140
130
120
110
100
90
80
70
60
50
40

c)

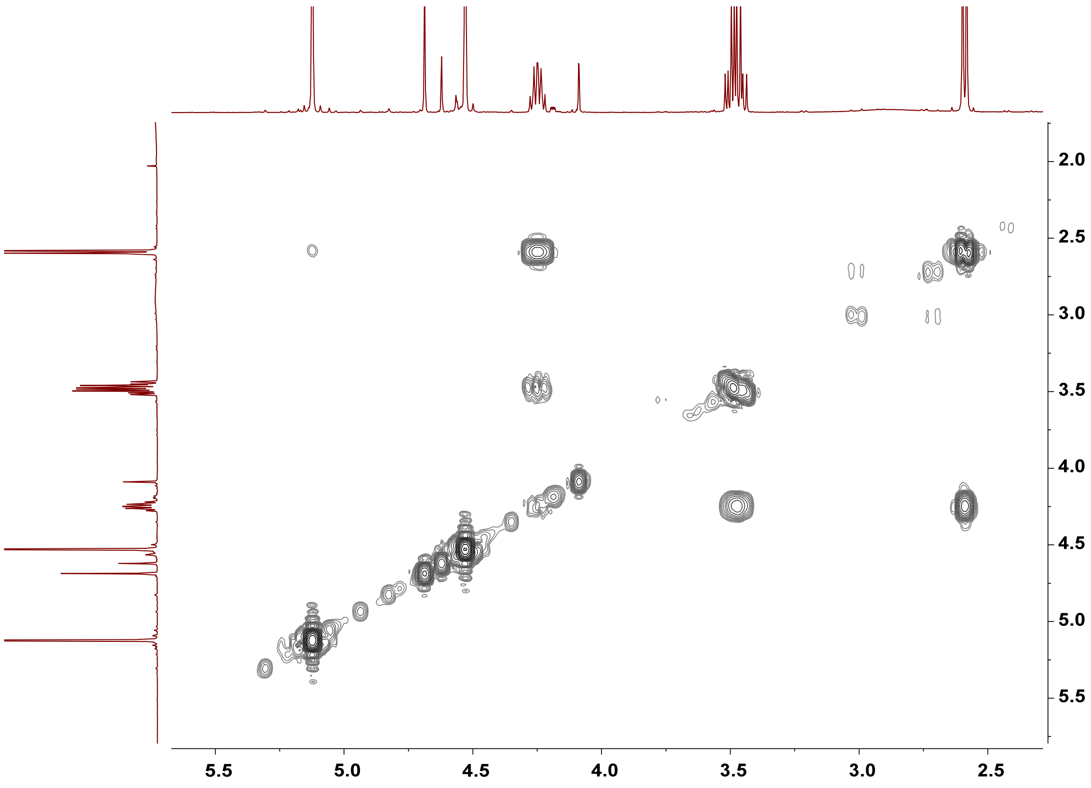

d)

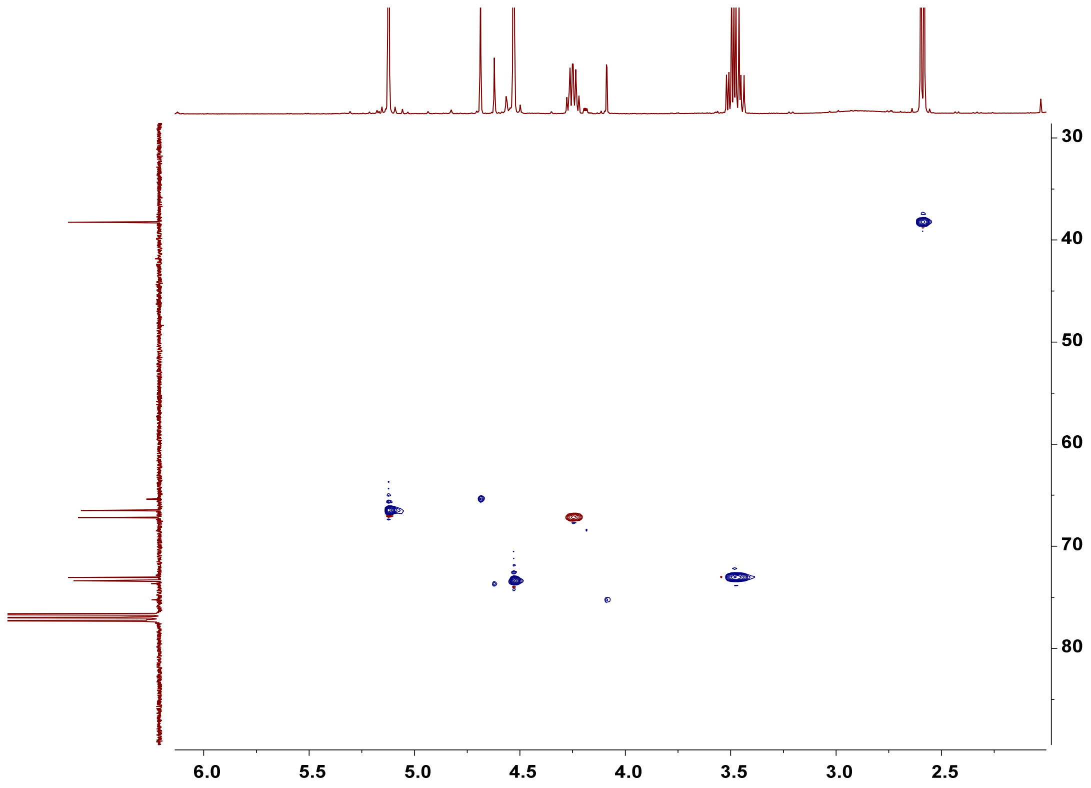

Figure S45. NMR spectra ($CDCl_3$) of *R*-**4b**: a) ^{1}H, b) ^{13}C, c) COSY and d) HSQC.

a)

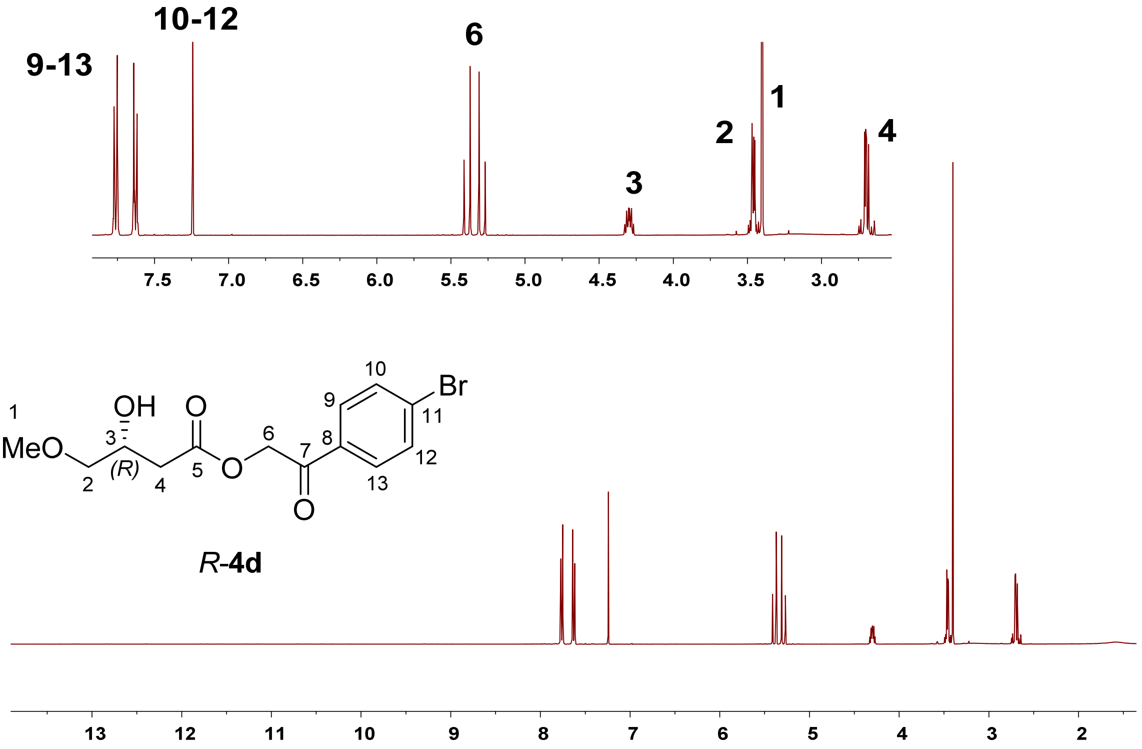

b)

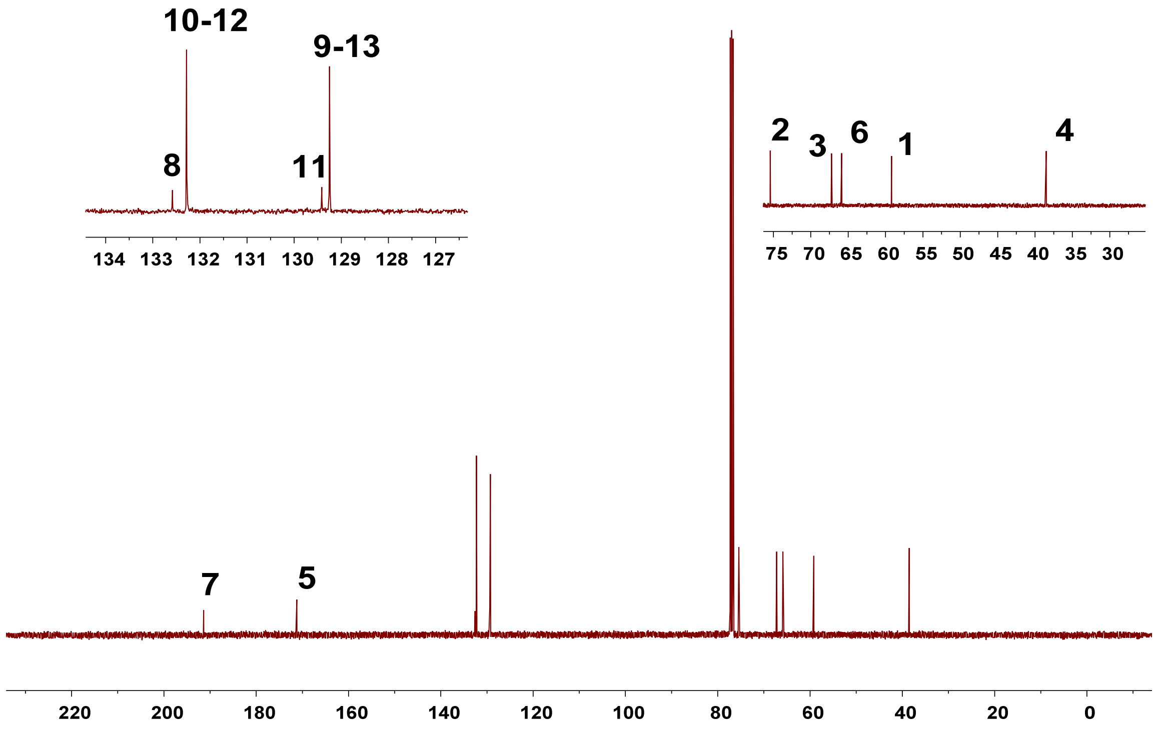

c)

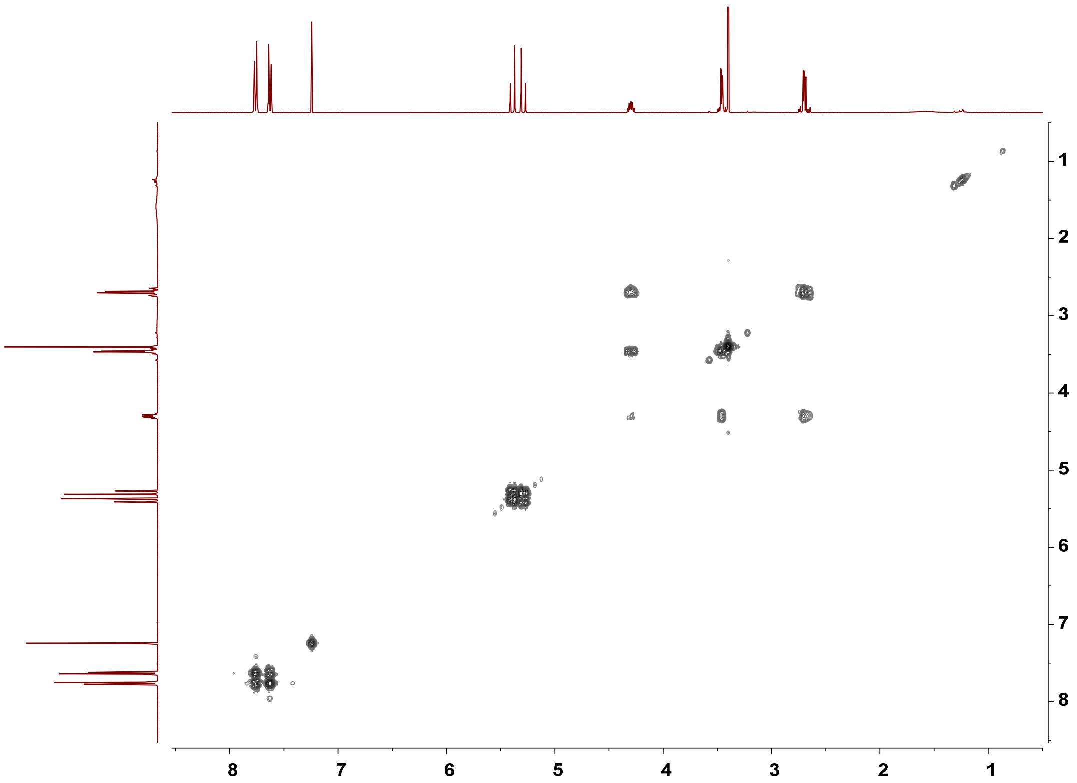

d)

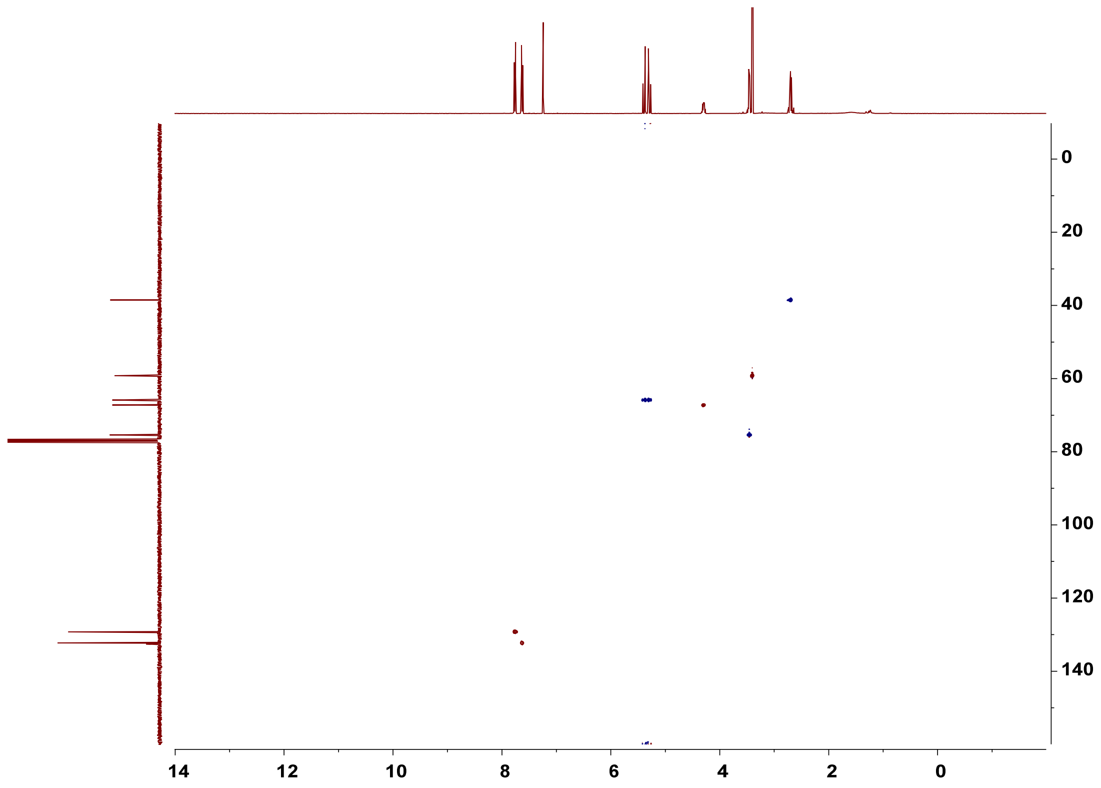

Figure S46. NMR spectra ($CDCl_3$) of *R*-**4d**: a) ^1H, b) ^{13}C, c) COSY and d) HSQC.

a)

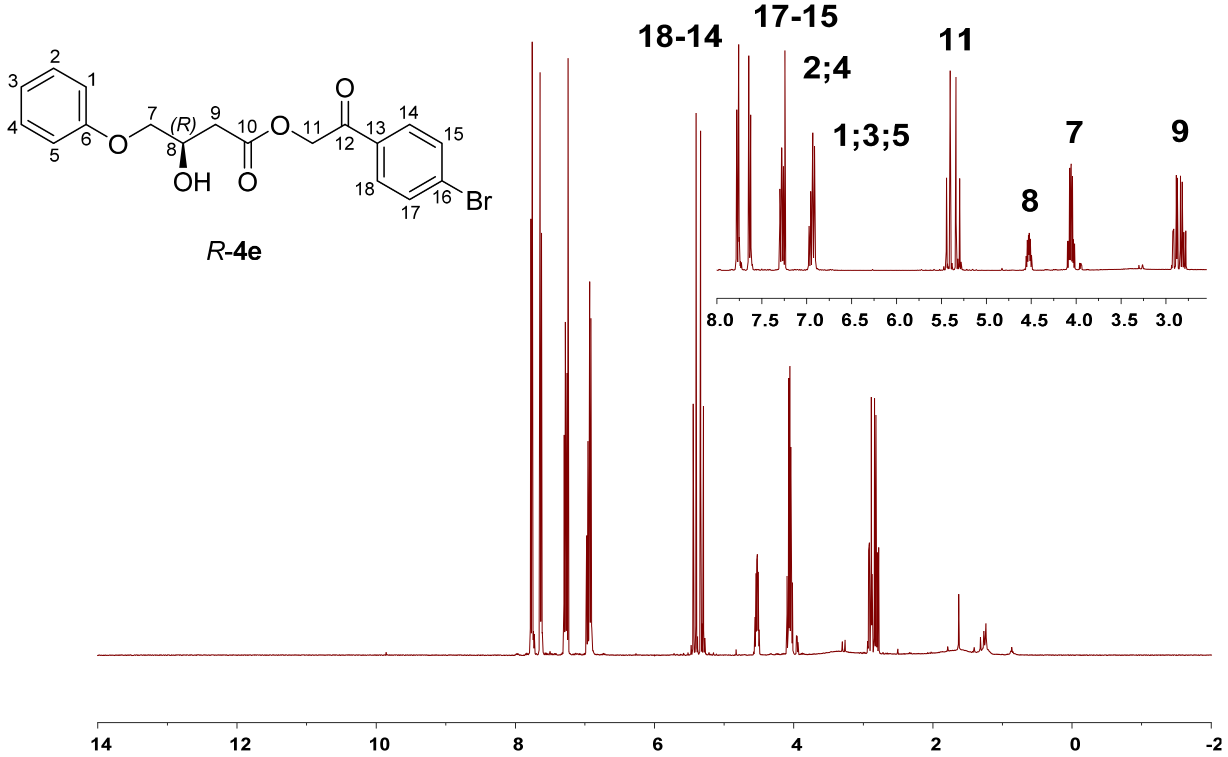
R-4e
OH
O
Br
18-14
17-15
2;4
1;3;5
11
8
7
9
8.0 7.5 7.0 6.5 6.0 5.5 5.0 4.5 4.0 3.5 3.0
14 12 10 8 6 4 2 0 -2

b)

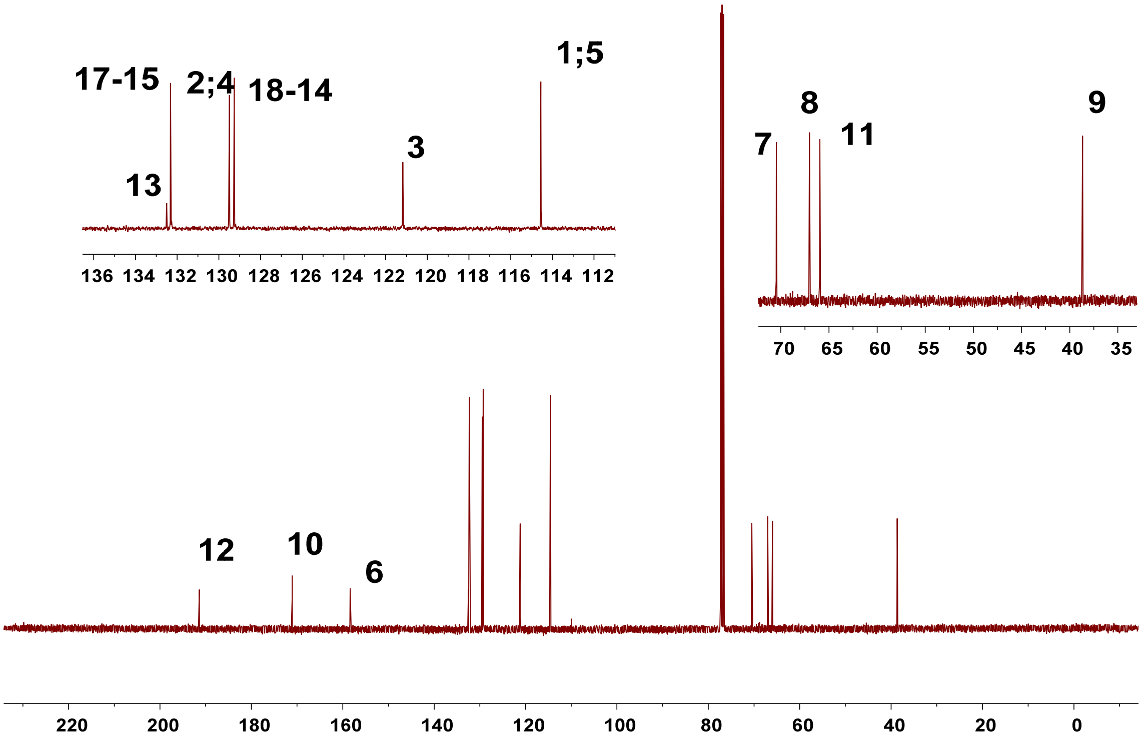
17-15
2;4
18-14
13
3
1;5
136 134 132 130 128 126 124 122 120 118 116 114 112
7
8
11
9
70 65 60 55 50 45 40 35
12
10
6
220 200 180 160 140 120 100 80 60 40 20 0

c)

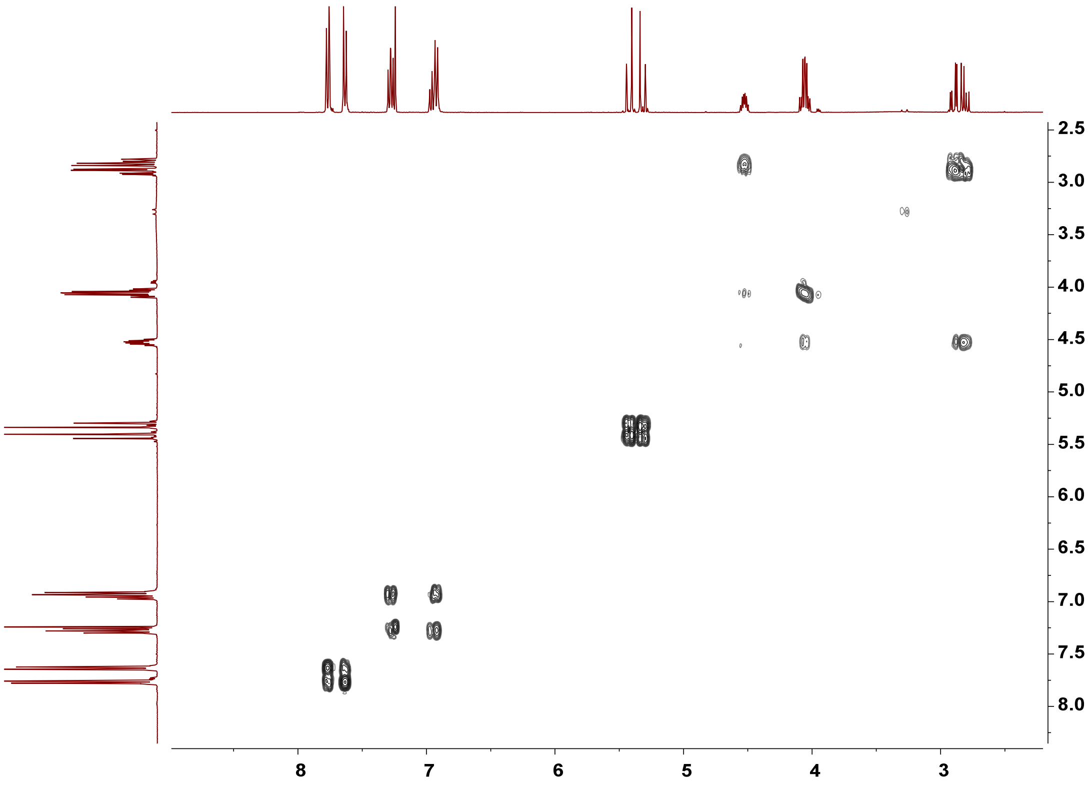

d)

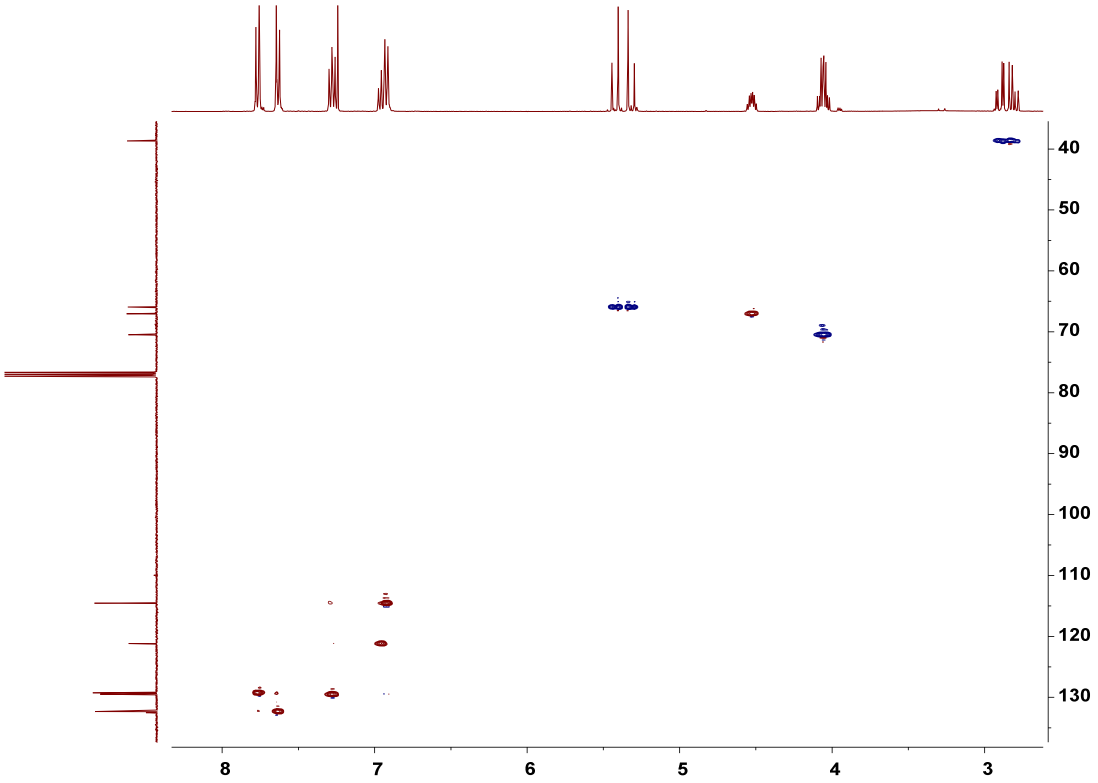

Figure S47. NMR spectra ($CDCl_3$) of *R*-**4e**: a) ^{1}H, b) ^{13}C, c) COSY and d) HSQC.

a)

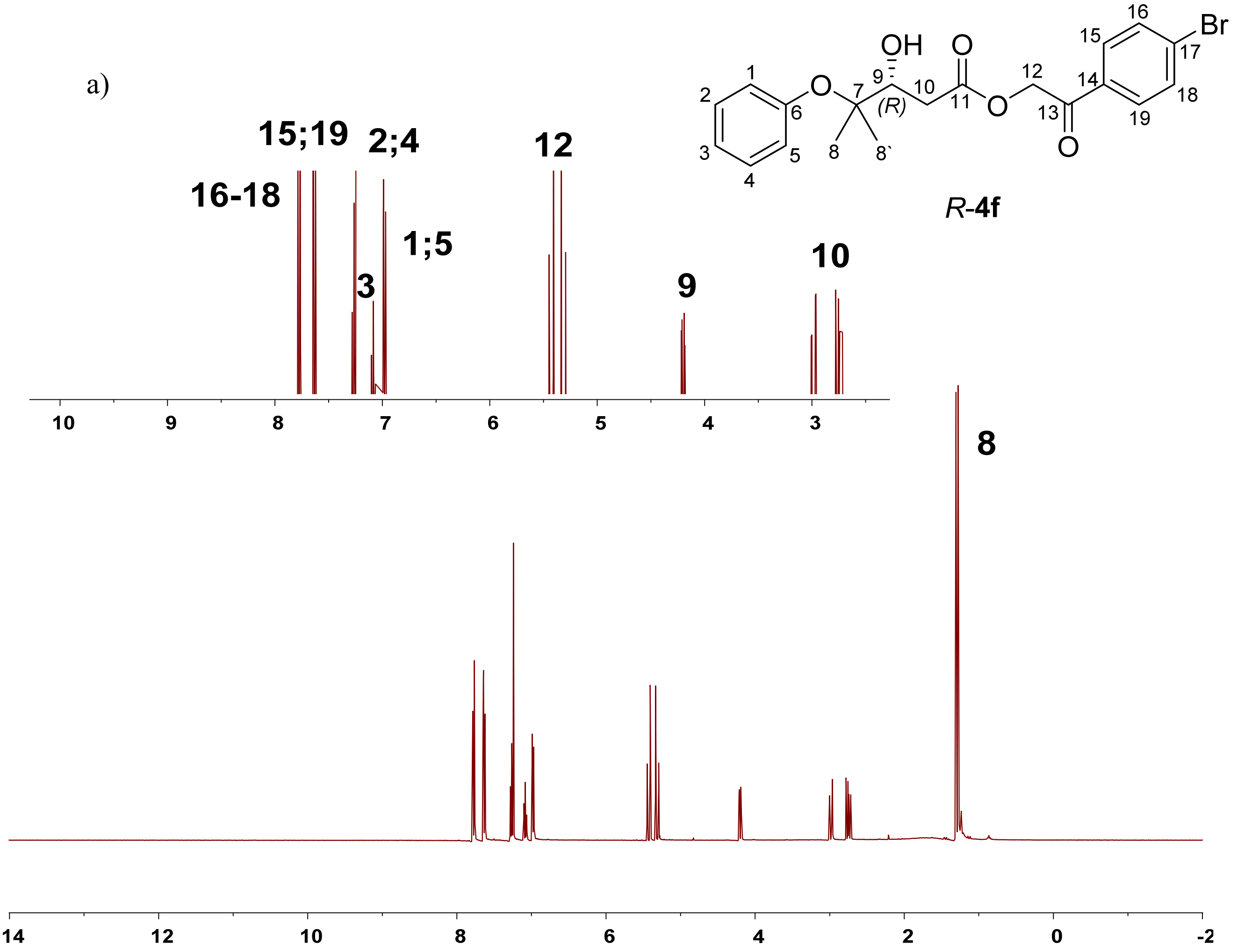
16-18
15;19
2;4
3
1;5
12
9
10
8
OH
O
Br
(R)
R-4f

b)

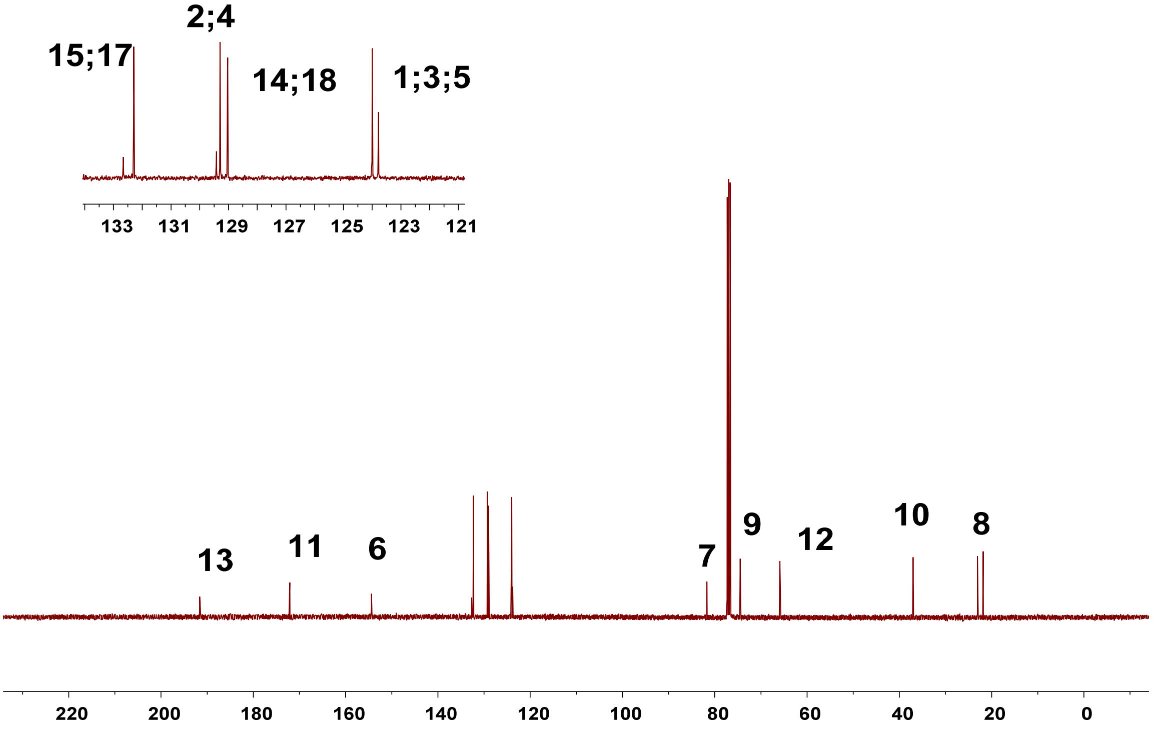
15;17
2;4
14;18
1;3;5
13
11
6
7
9
12
10
8

c)

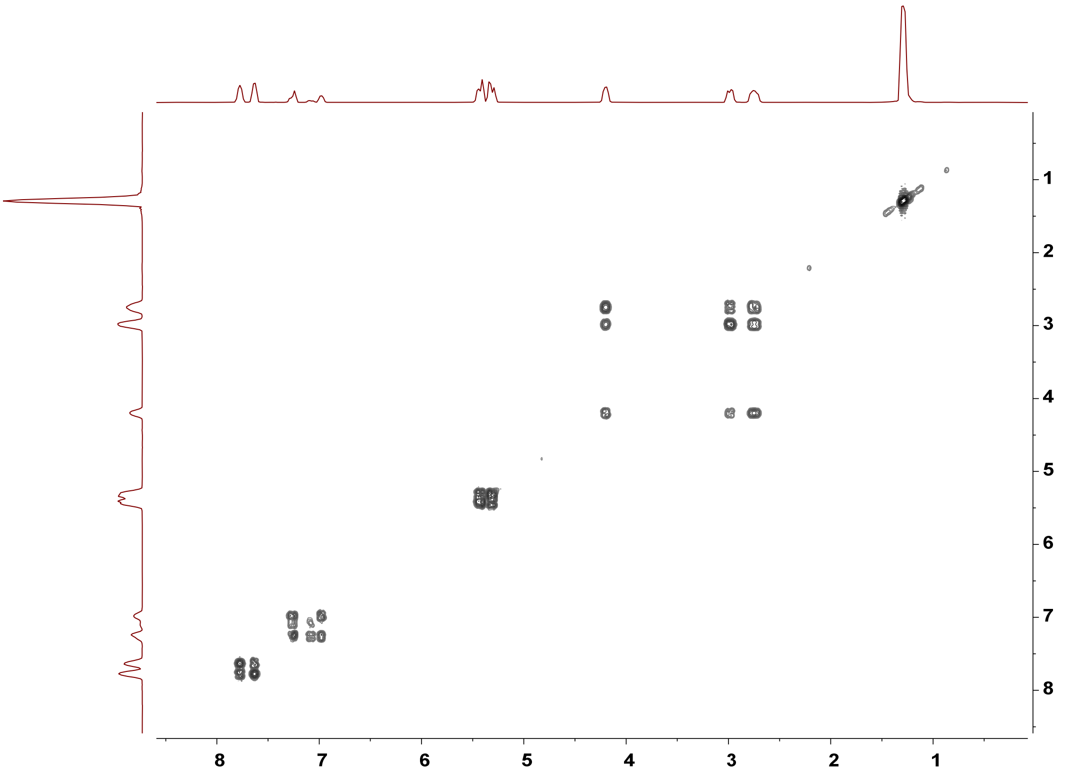

d)

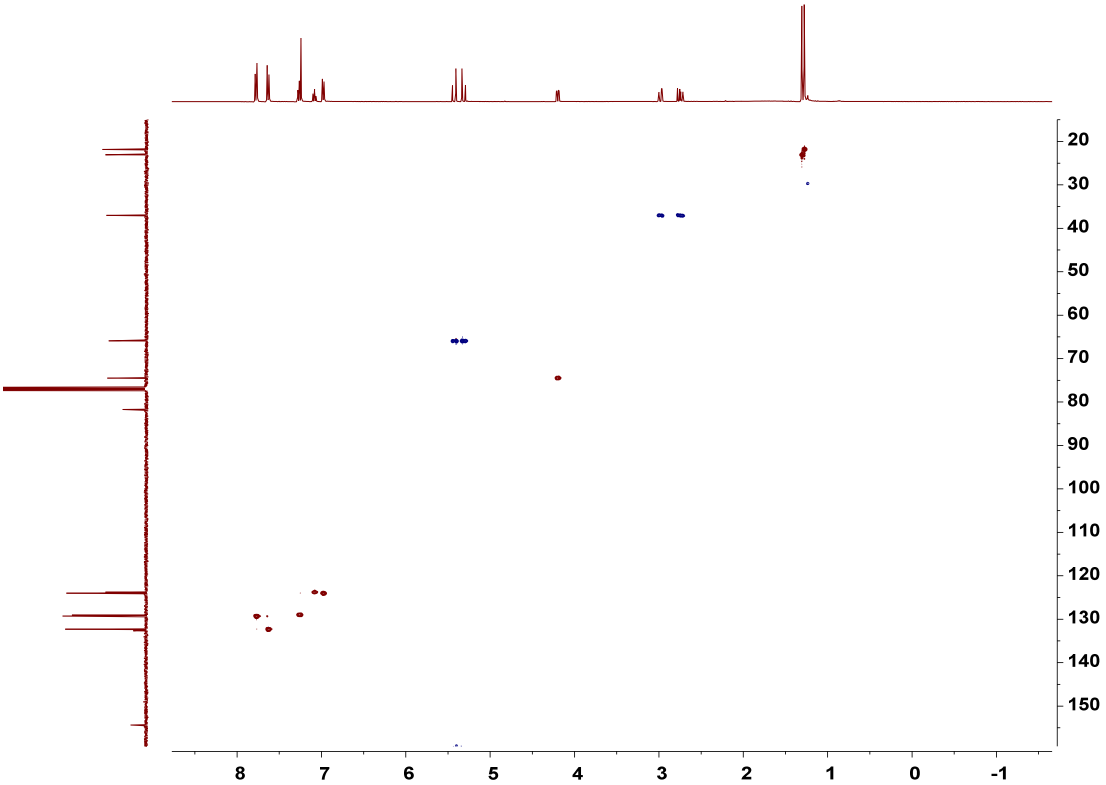

Figure S48. NMR spectra (CDCl3) of *R*-**4f**: a) ^{1}H, b) ^{13}C, c) COSY and d) HSQC.

a)

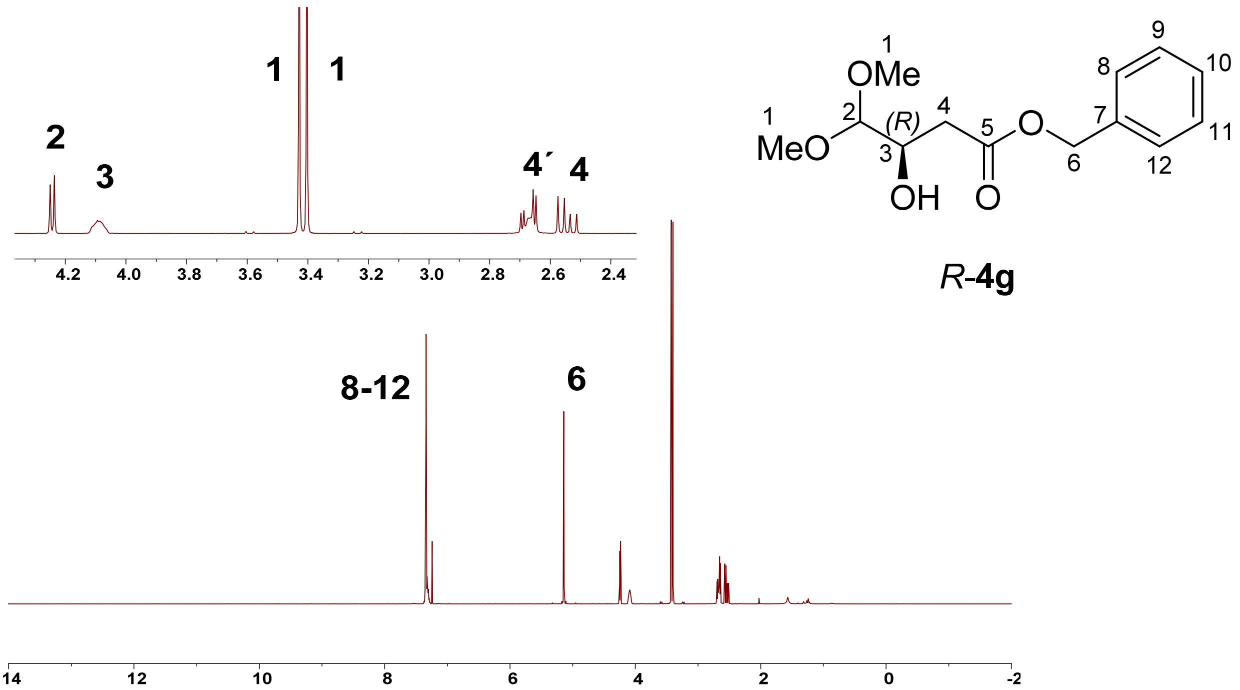

b)

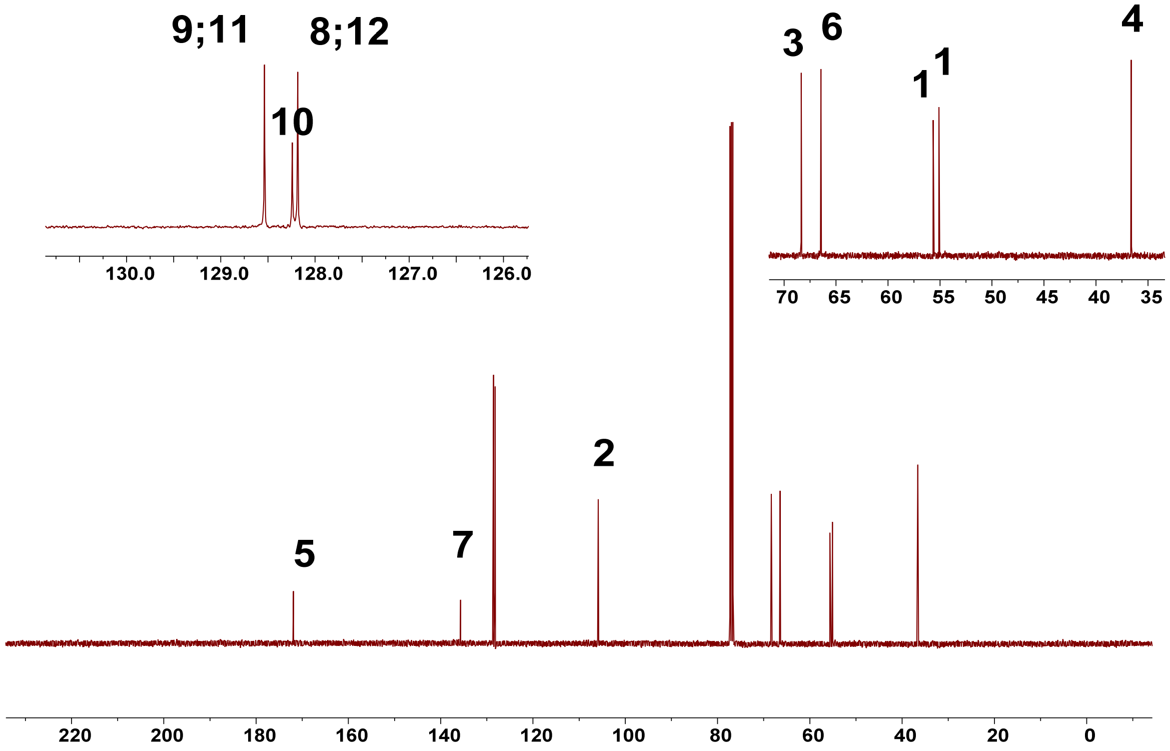

c)

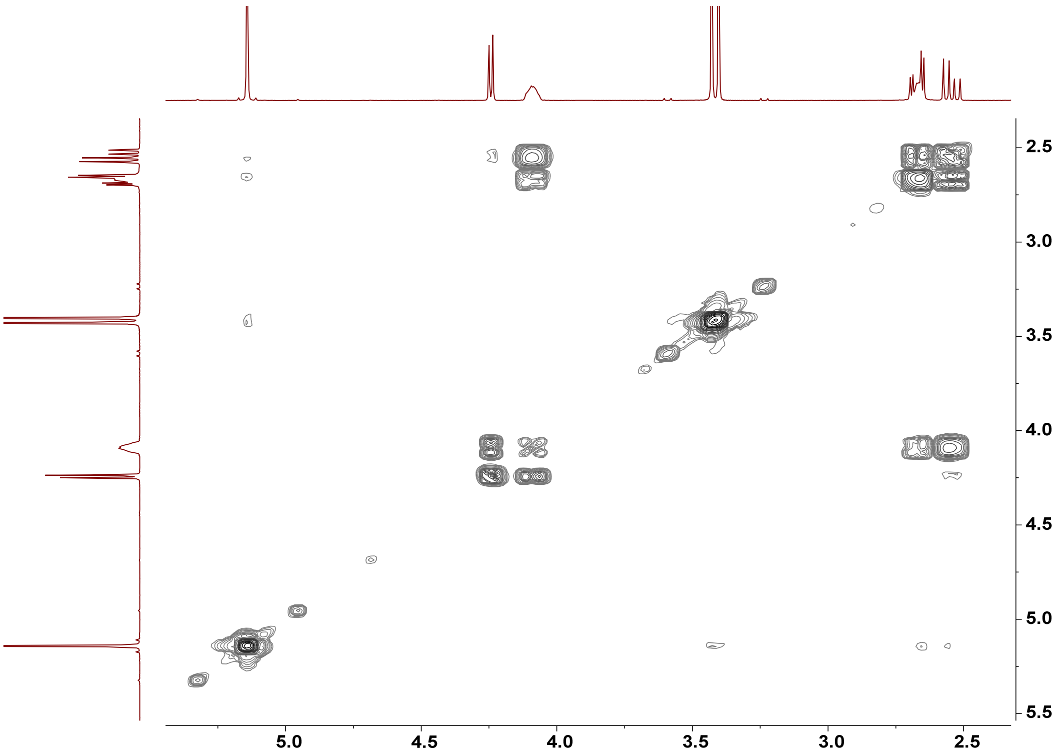

d)

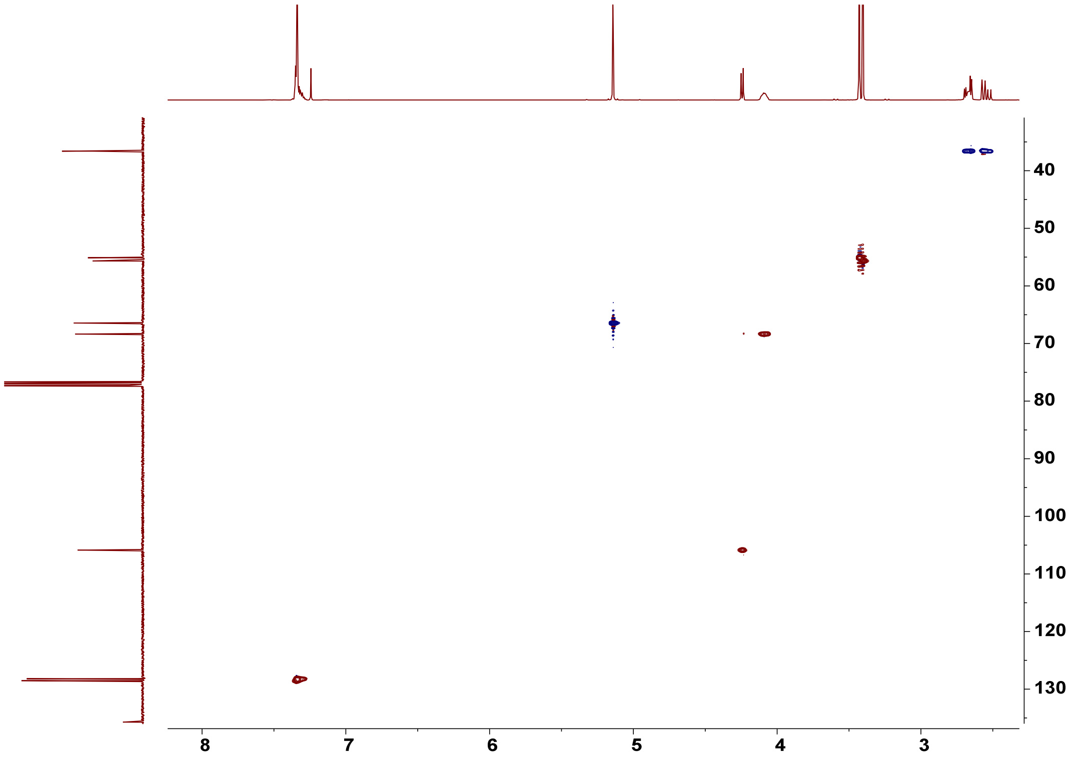

Figure S49. NMR spectra ($CDCl_3$) of *R*-**4g**: a) ^{1}H, b) ^{13}C, c) COSY and d) HSQC.

a)

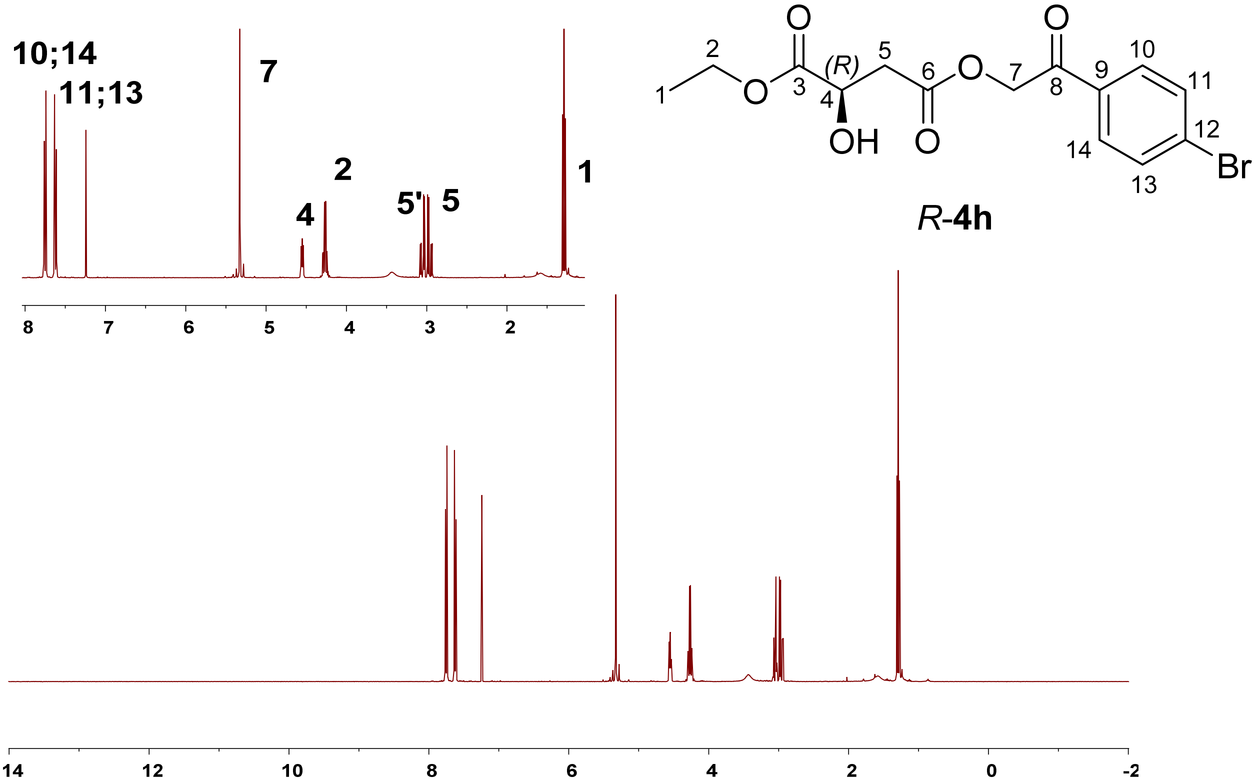

b)

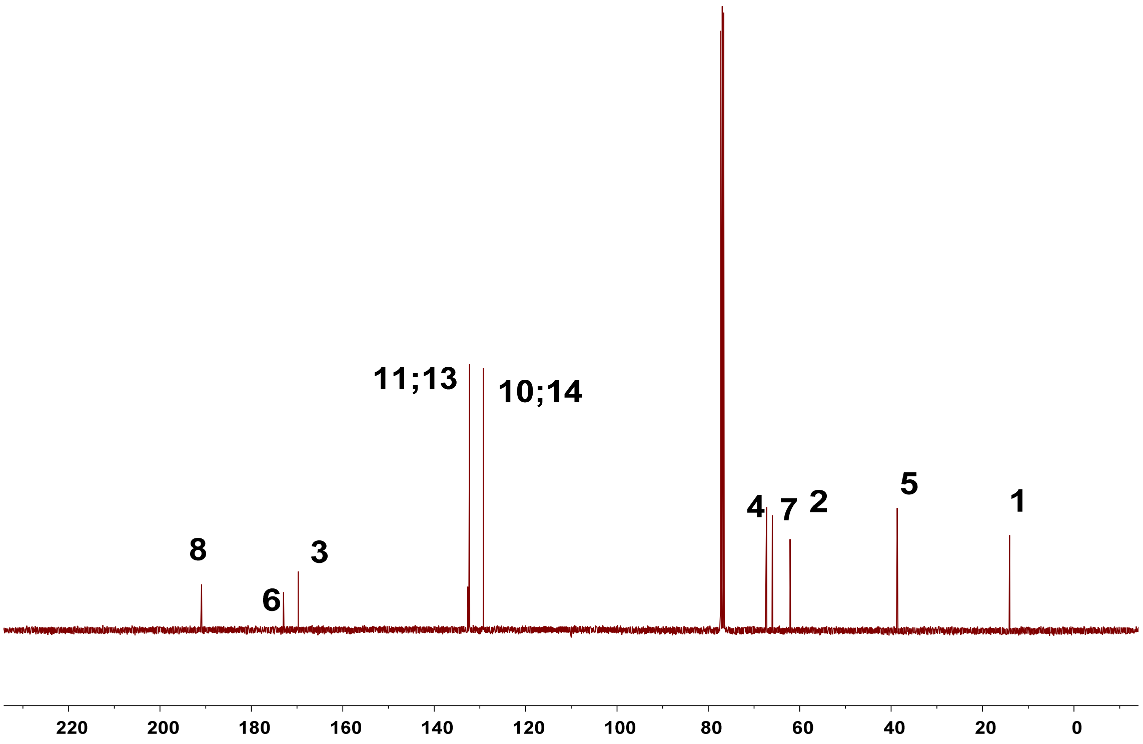

c)

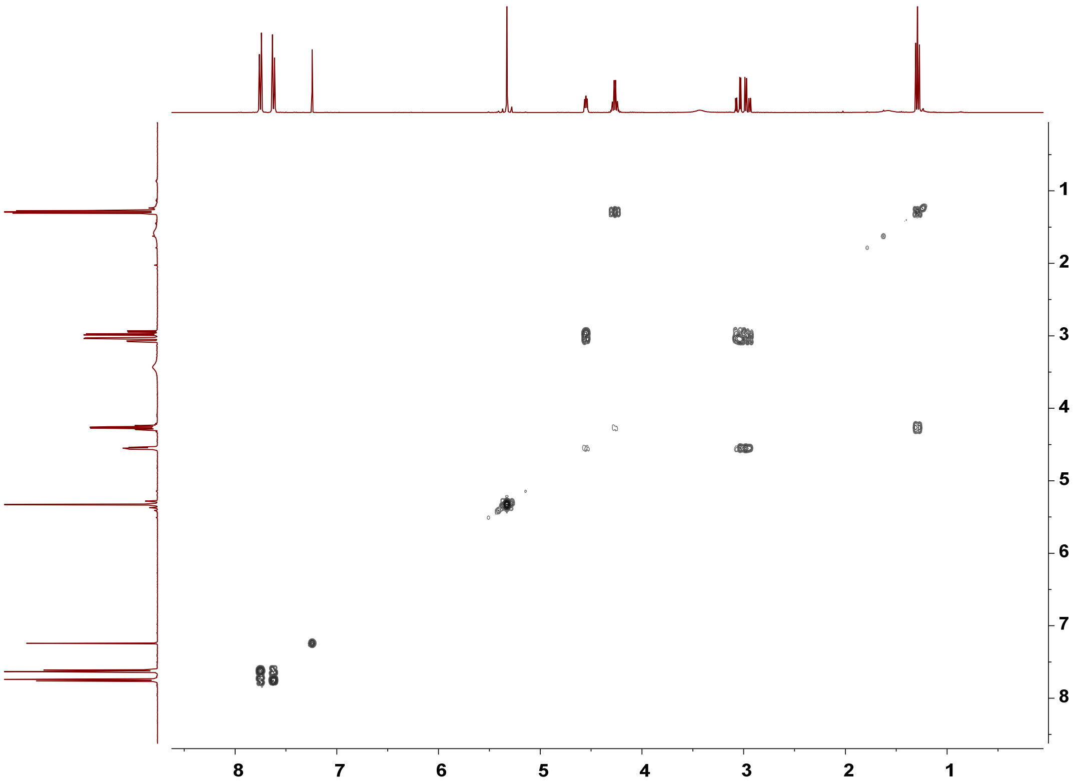

d)

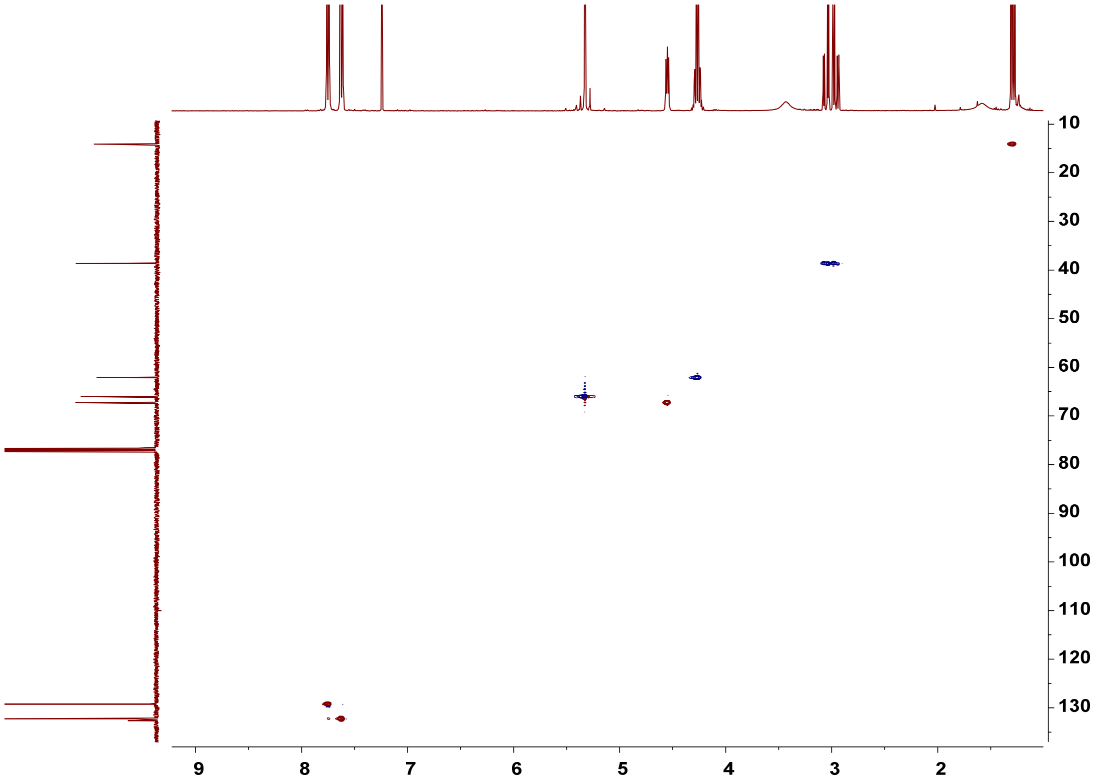

Figure S50. NMR spectra ($CDCl_3$) of *R*-**4h**: a) ^{1}H, b) ^{13}C, c) COSY and d) HSQC.

a)

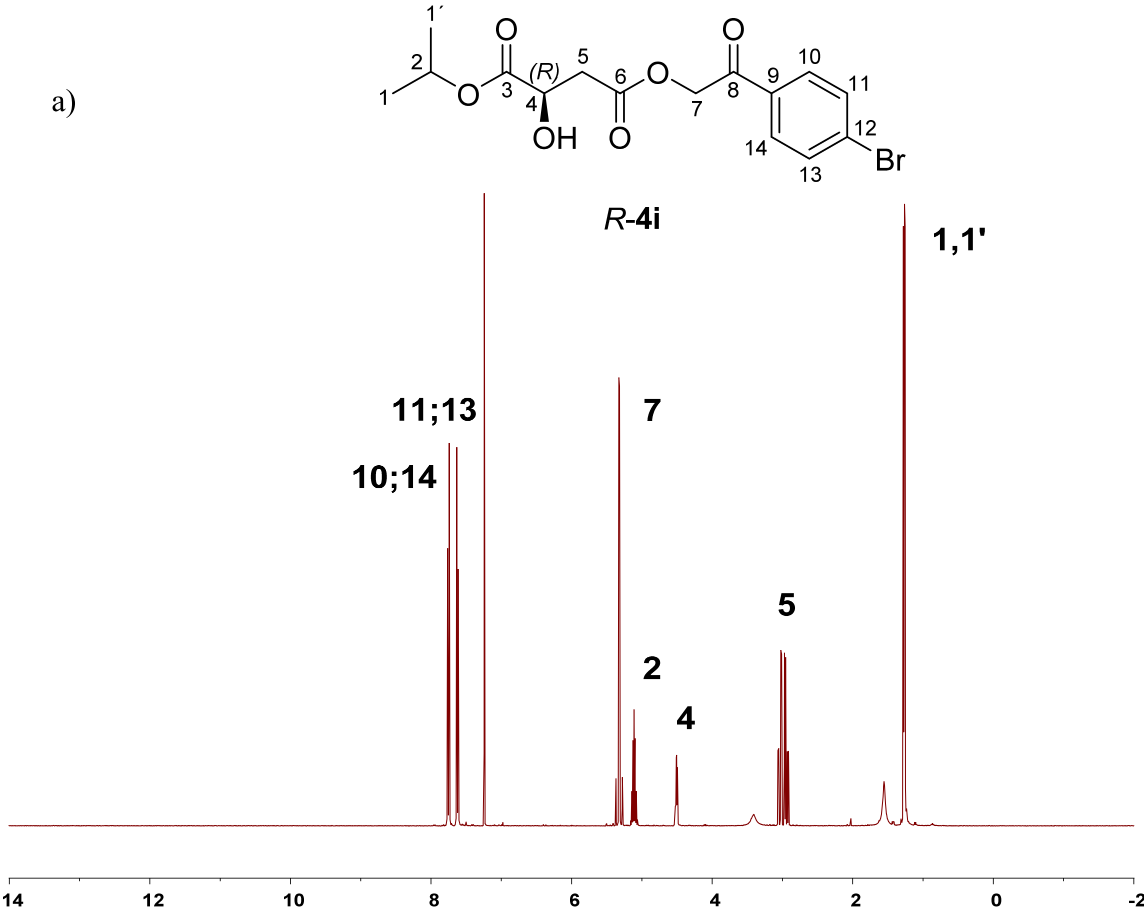

R-4i
1,1'
11;13
10;14
7
5
2
4
14
12
10
8
6
4
2
0
-2

b)

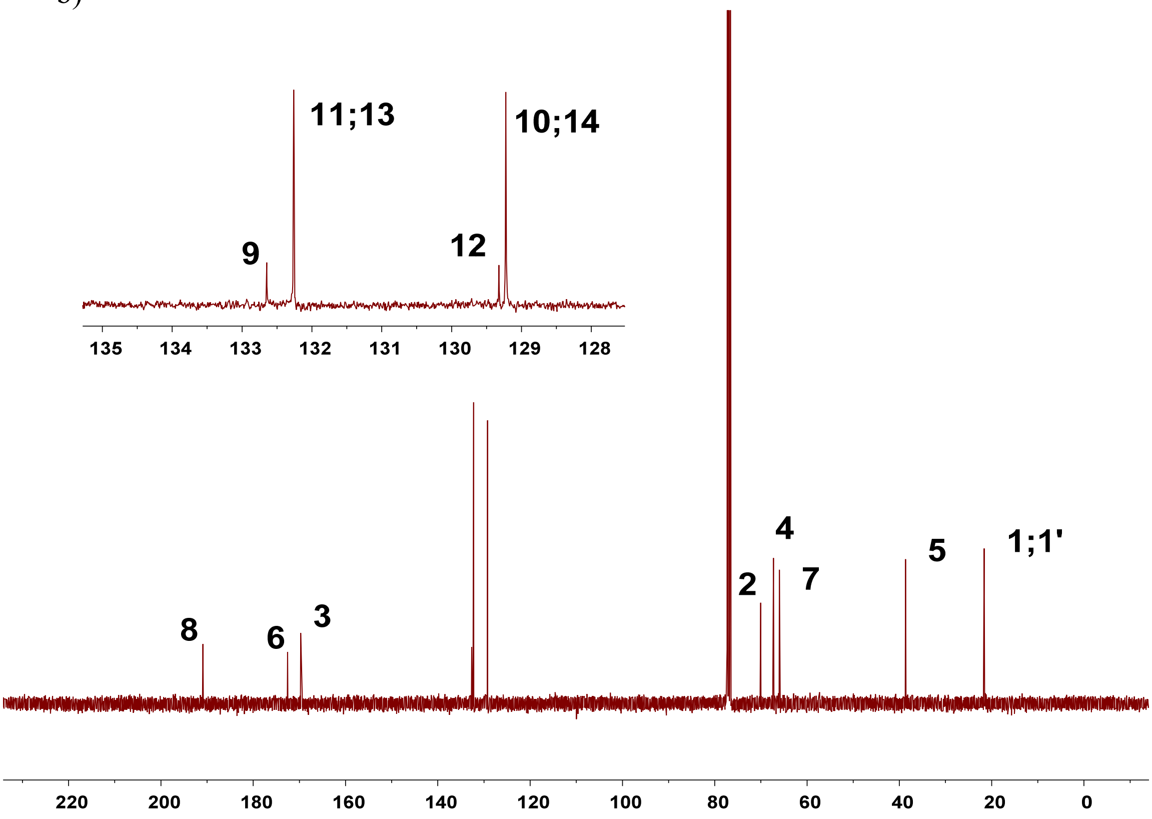

11;13
10;14
9
12
135
134
133
132
131
130
129
128
8
6
3
2
4
7
5
1;1'
220
200
180
160
140
120
100
80
60
40
20
0

c)

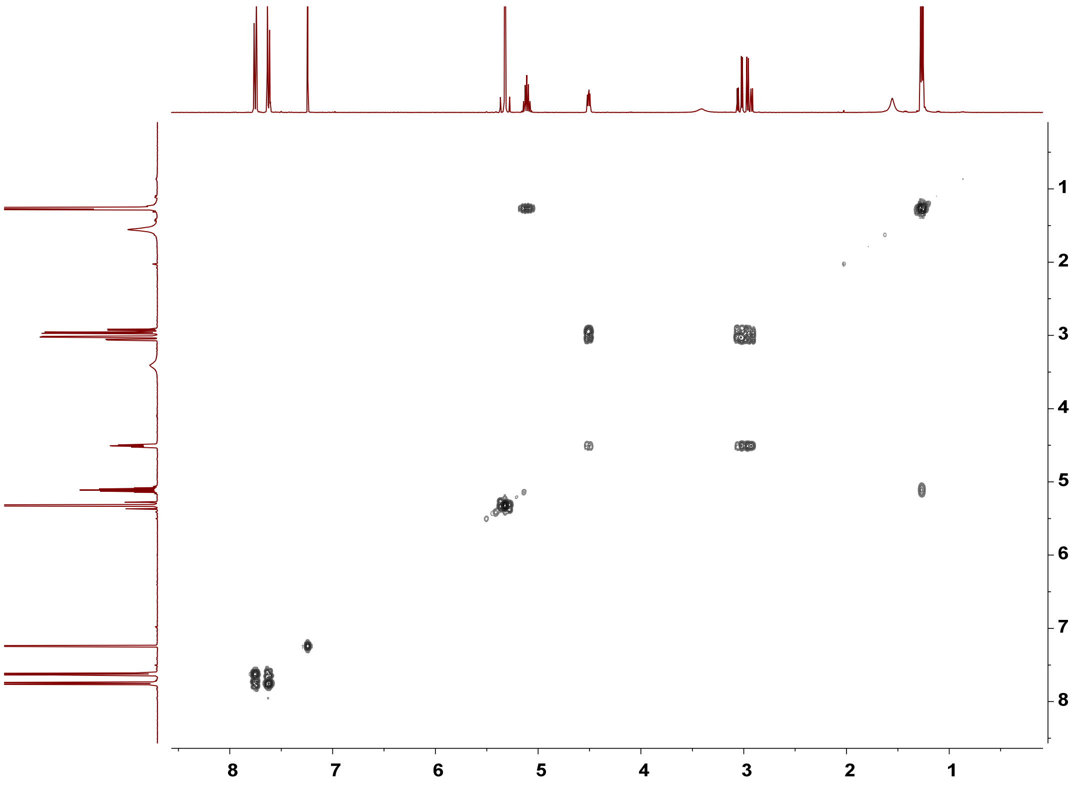

d)

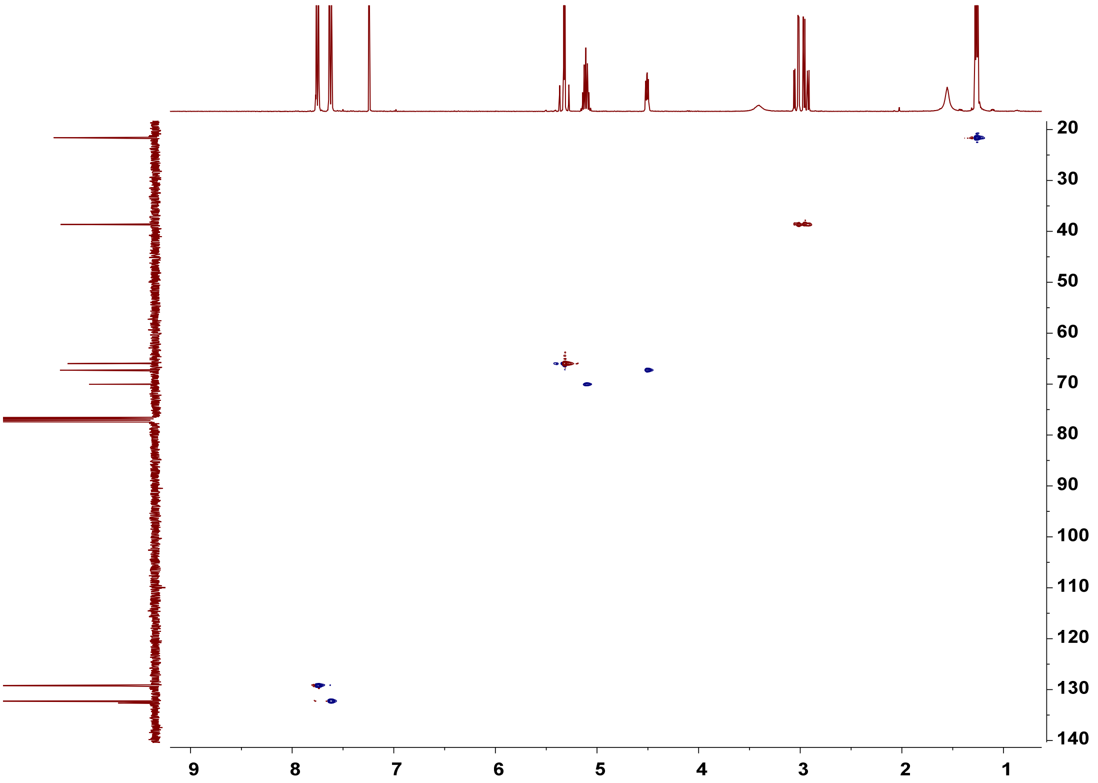

Figure S51. NMR spectra ($CDCl_3$) of *R*-**4i**: a) ^{1}H, b) ^{13}C, c) COSY and d) HSQC.

a)

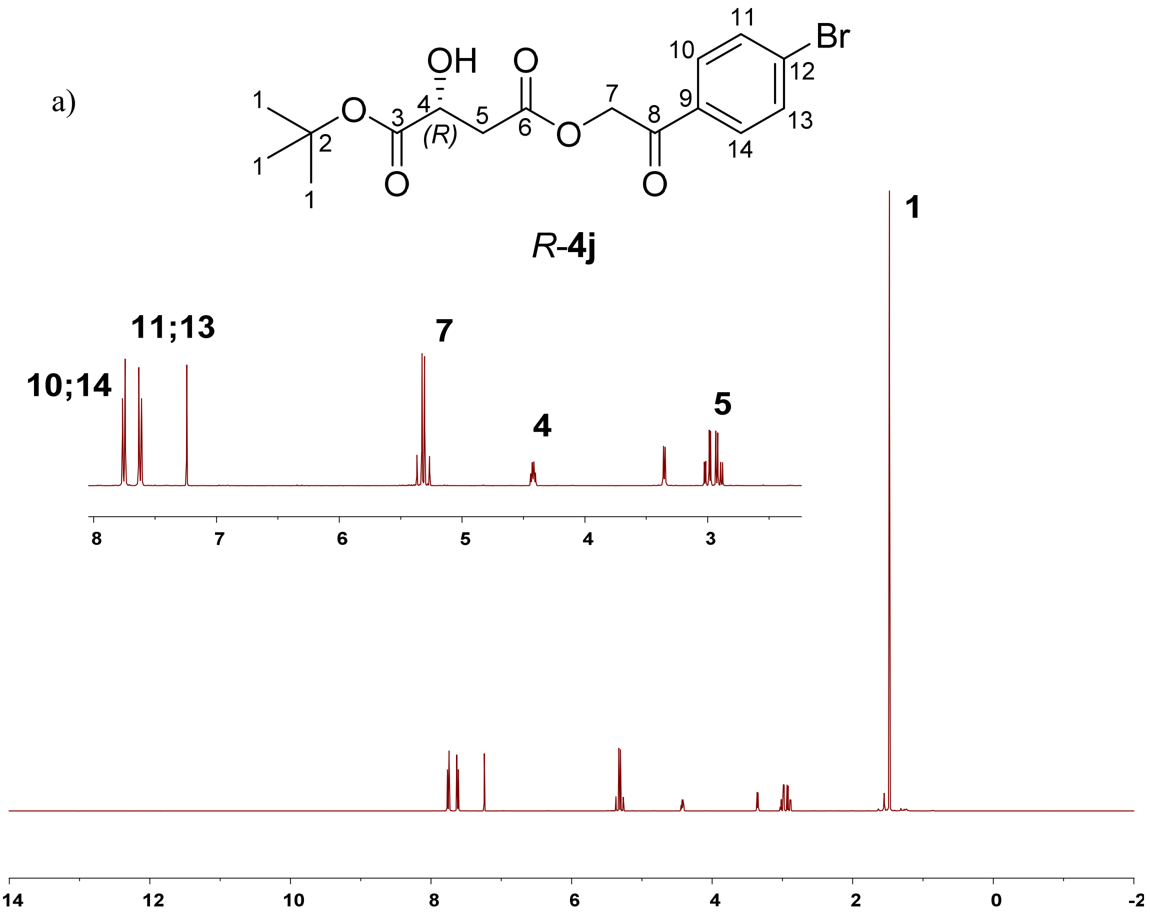
OH
O
O
O
O
O
O
Br
1
1
1
2
3
4
(R)
5
6
7
8
9
10
11
12
13
14
R-4j
1
10;14
11;13
7
4
5
8
7
6
5
4
3
14
12
10
8
6
4
2
0
-2

b)

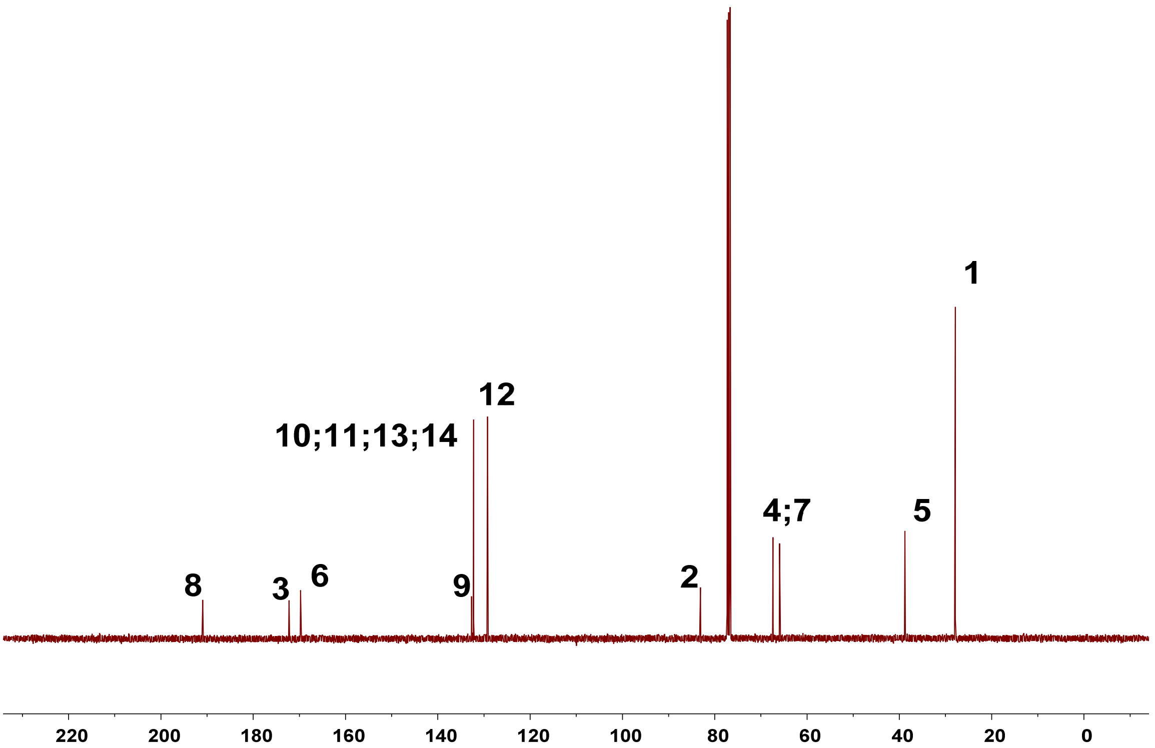
1
12
10;11;13;14
4;7
5
8
3
6
9
2
220
200
180
160
140
120
100
80
60
40
20
0

c)

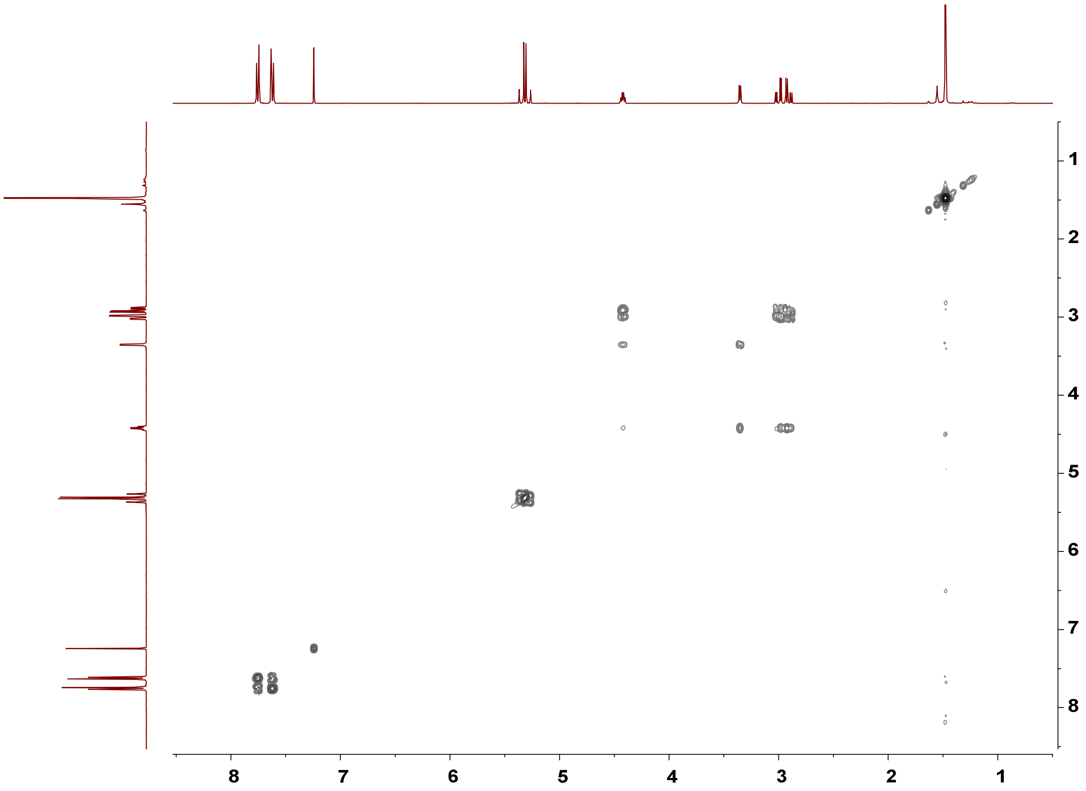

d)

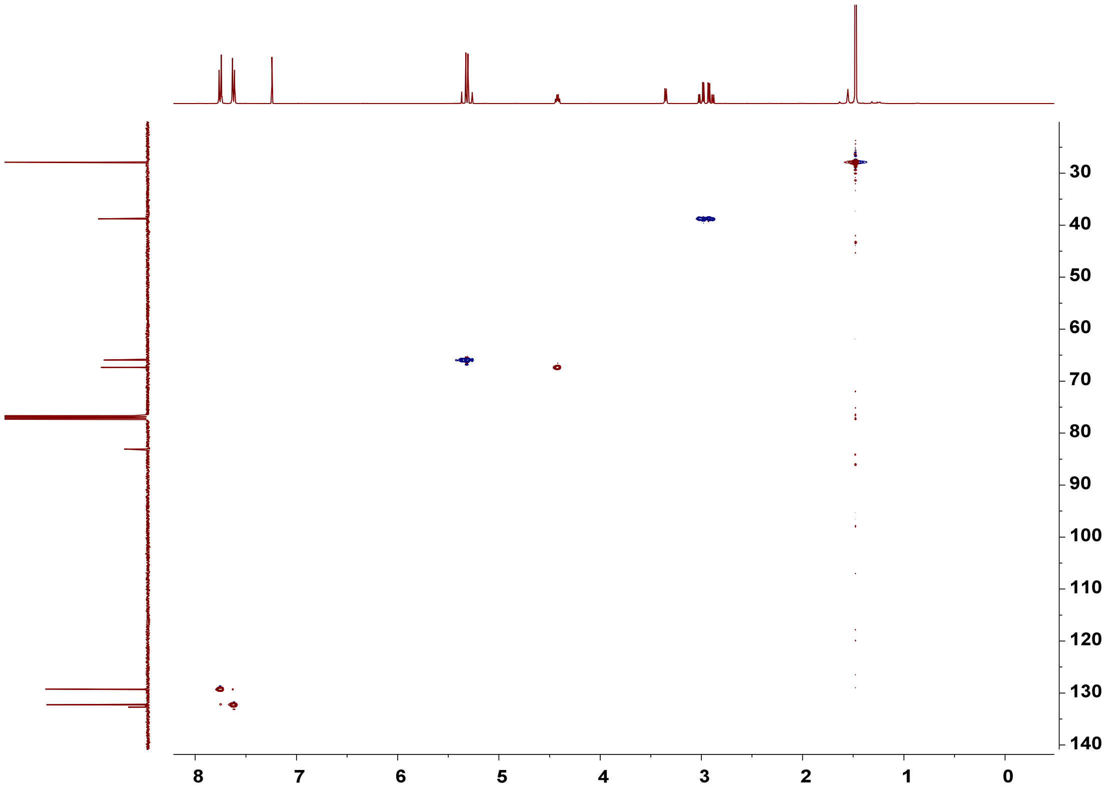

Figure S52. NMR spectra ($CDCl_3$) of *R*-**4j**: a) ^{1}H, b) ^{13}C, c) COSY and d) HSQC.

a)

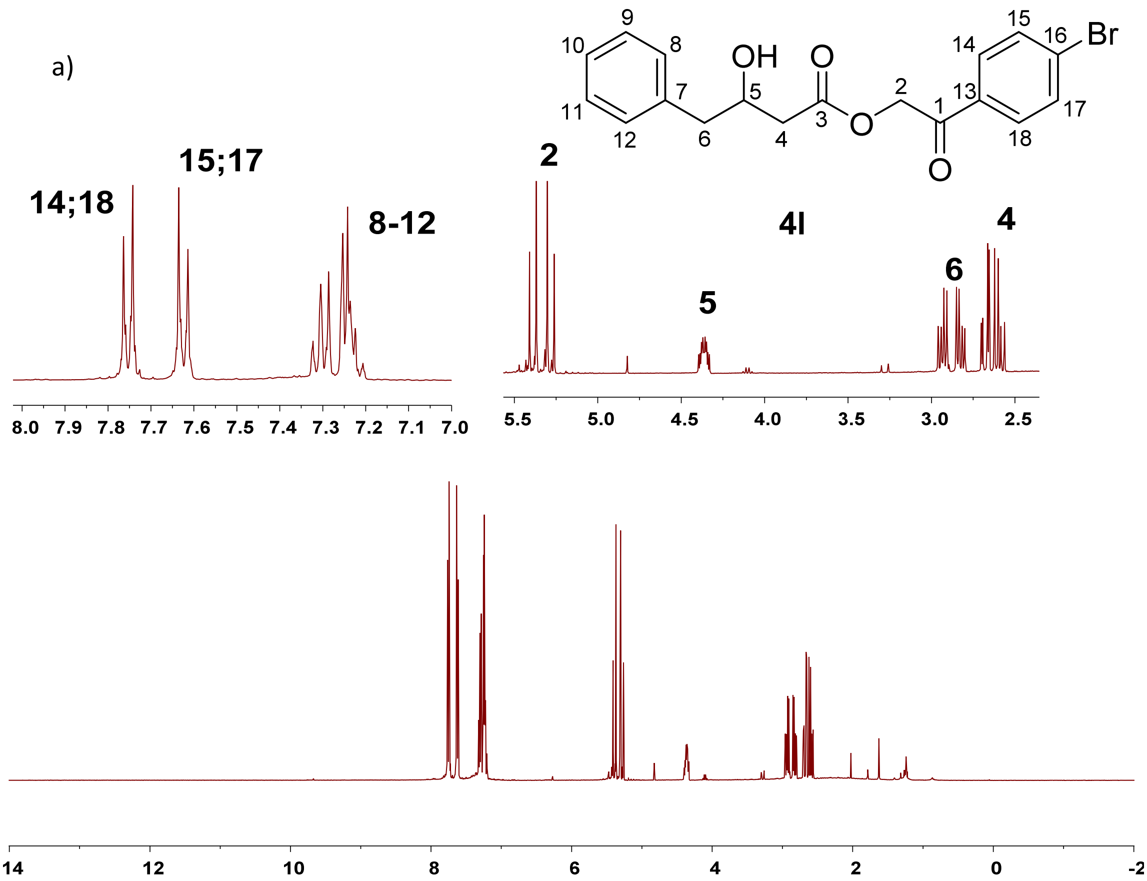
4l
Br
OH
O
O
O
14;18
15;17
8-12
2
5
6
4
8.0 7.9 7.8 7.7 7.6 7.5 7.4 7.3 7.2 7.1 7.0
5.5 5.0 4.5 4.0 3.5 3.0 2.5
14 12 10 8 6 4 2 0 -2

b)

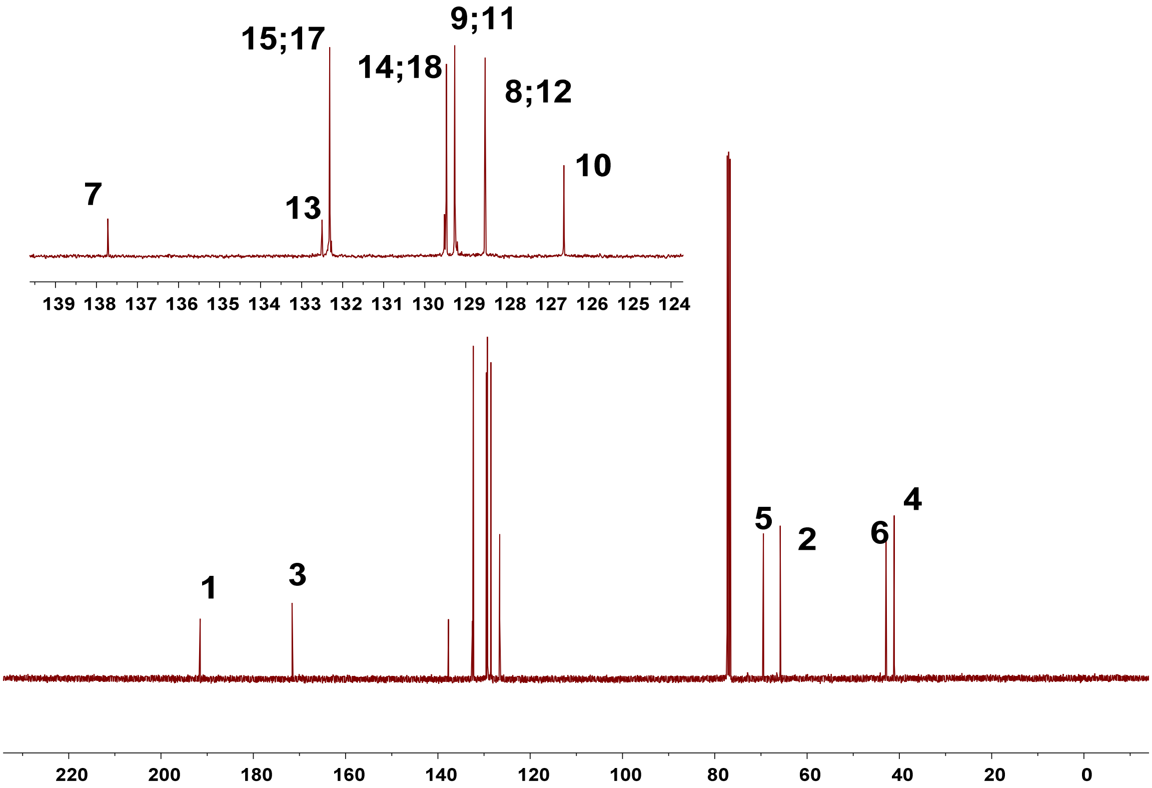
15;17
9;11
14;18
8;12
10
7
13
139 138 137 136 135 134 133 132 131 130 129 128 127 126 125 124
1
3
5
2
6
4
220 200 180 160 140 120 100 80 60 40 20 0

c)

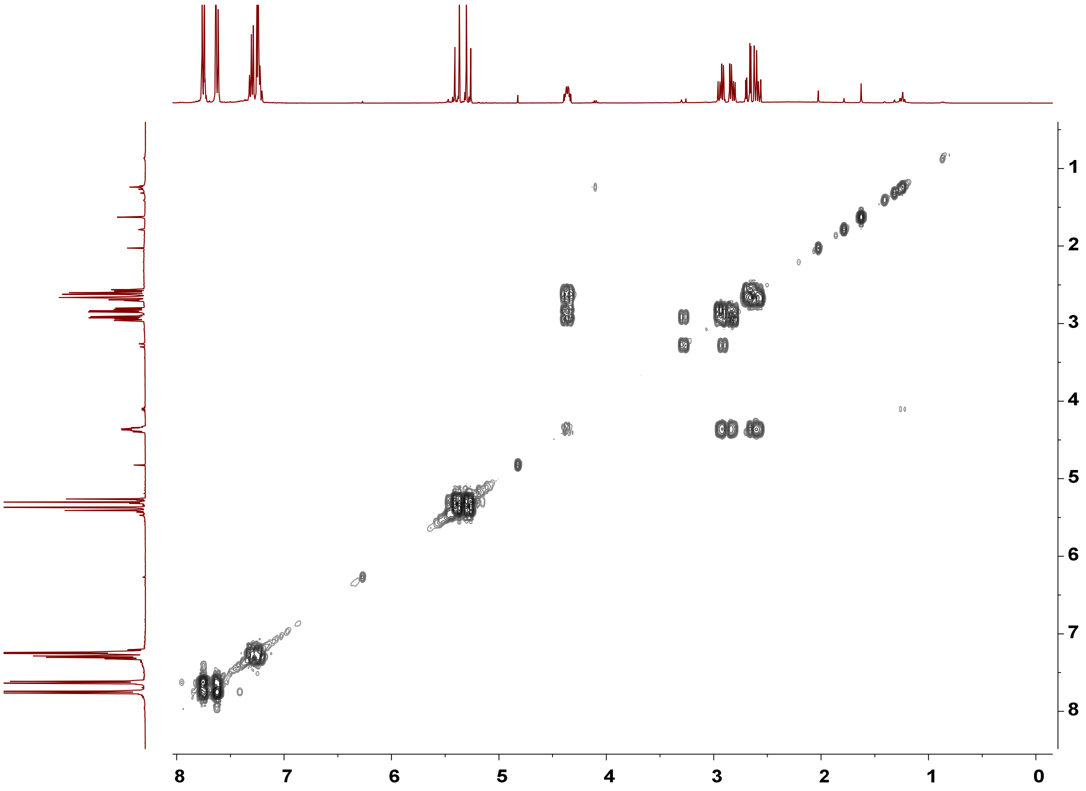

d)

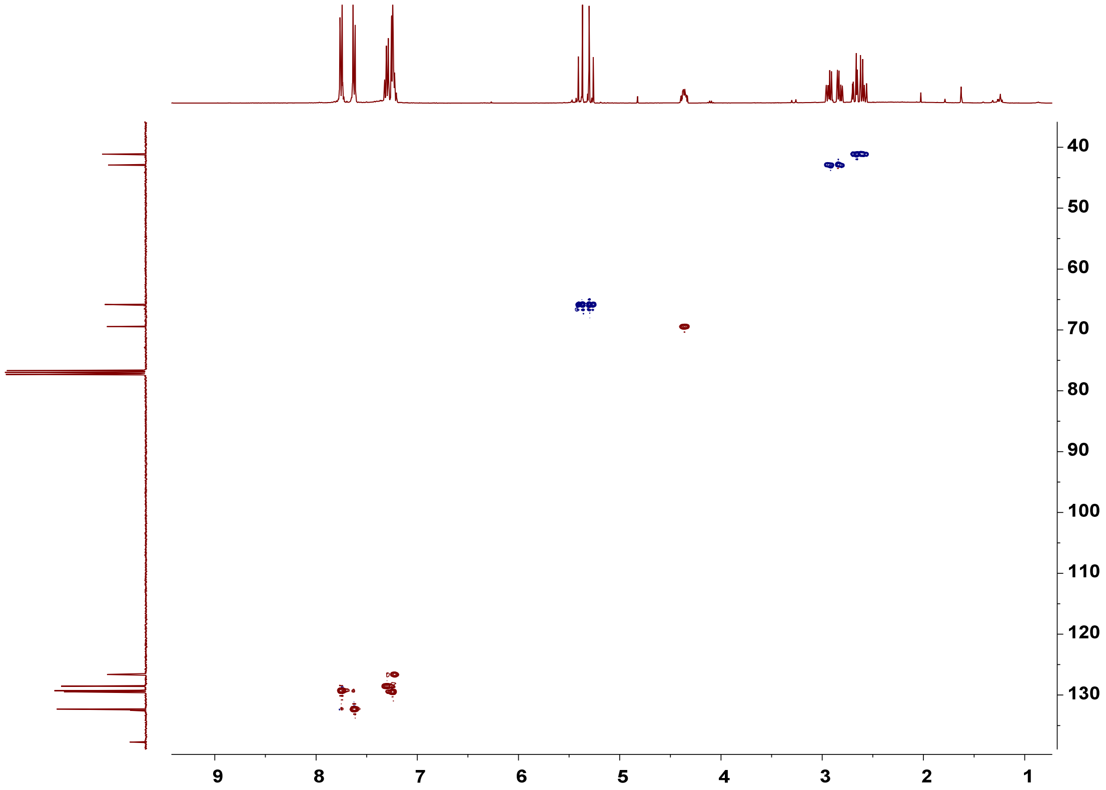

Figure S53. NMR spectra (CDCl3) of **4l**: a) ^{1}H, b) ^{13}C, c) COSY and d) HSQC.

a)

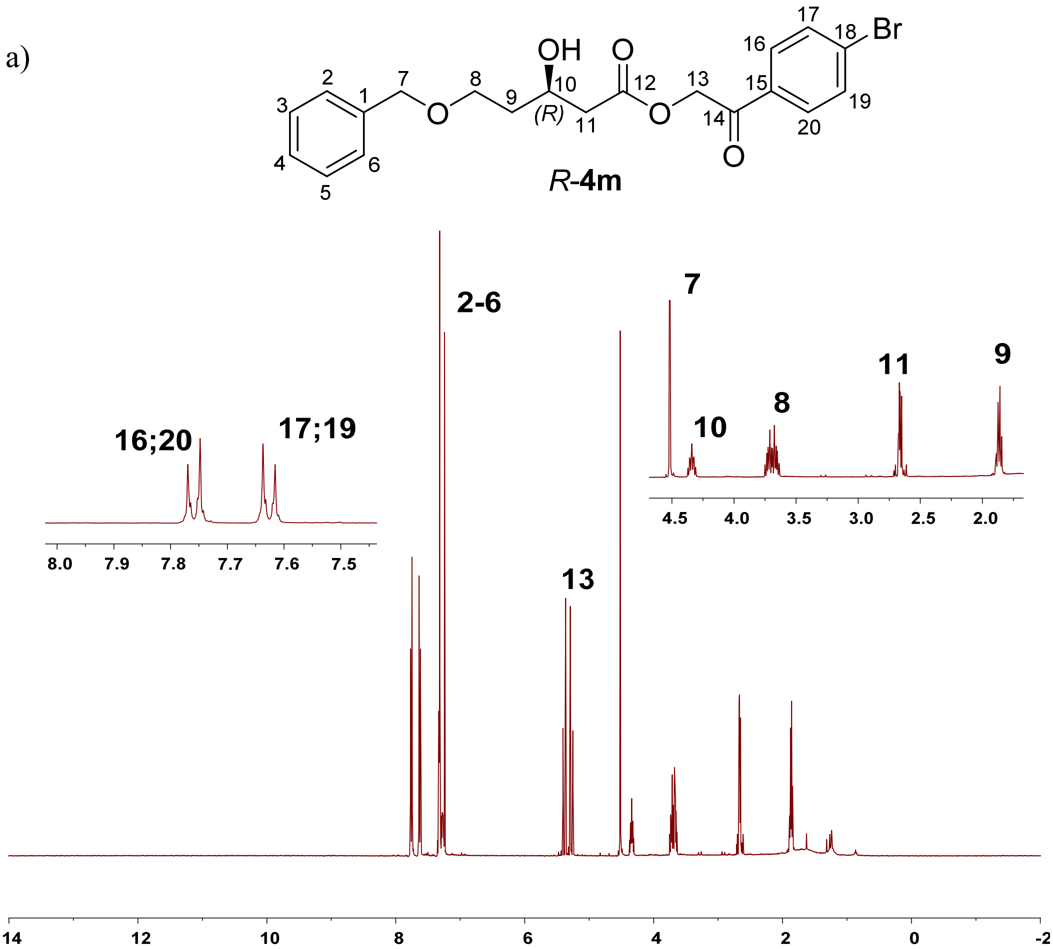

OH
O
Br
(R)
R-4m
2-6
7
10
8
11
9
16;20
17;19
13

b)

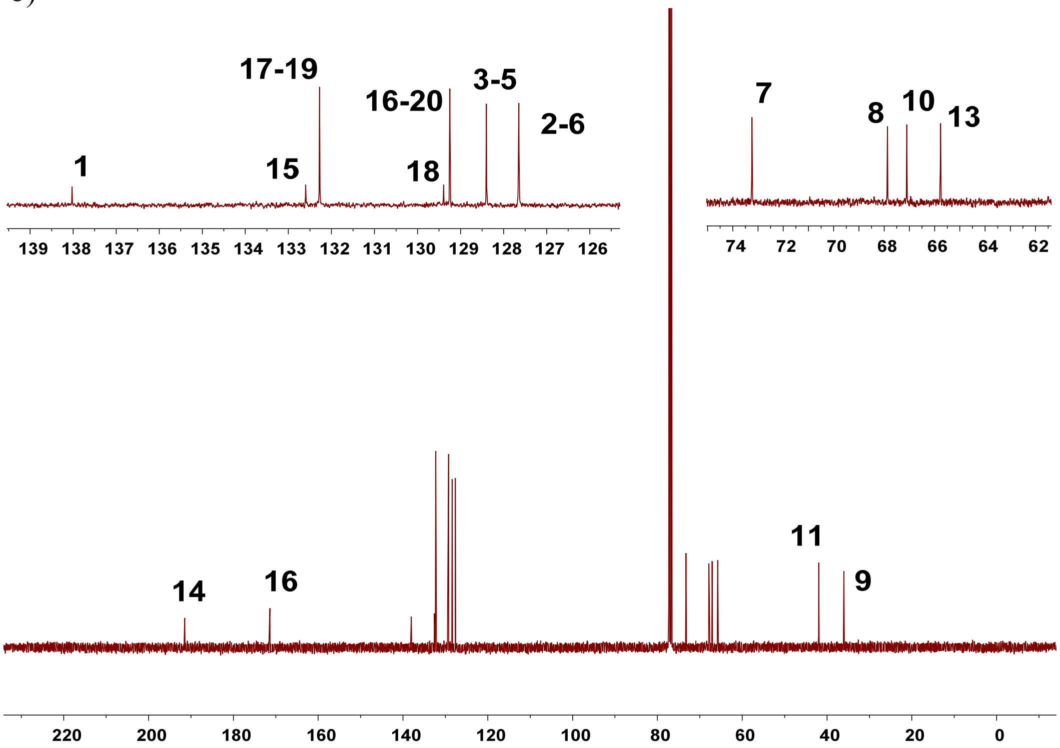

17-19
16-20
3-5
2-6
1
15
18
7
8
10
13
14
16
11
9

c)

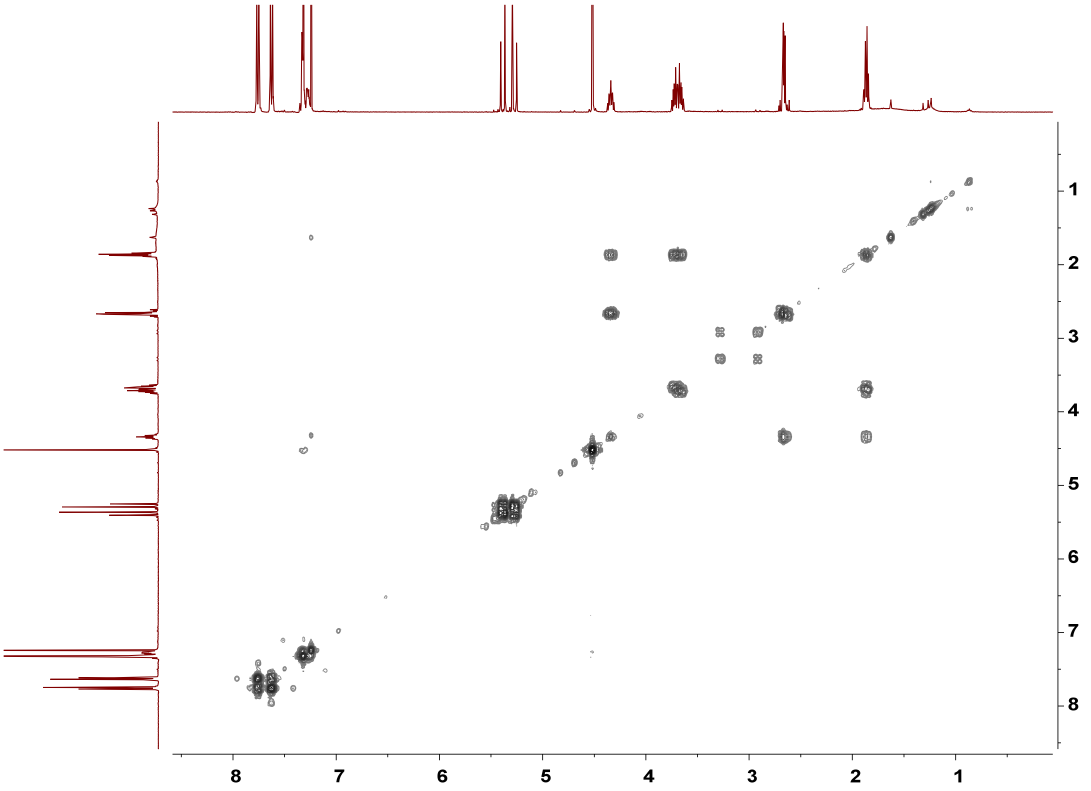

d)

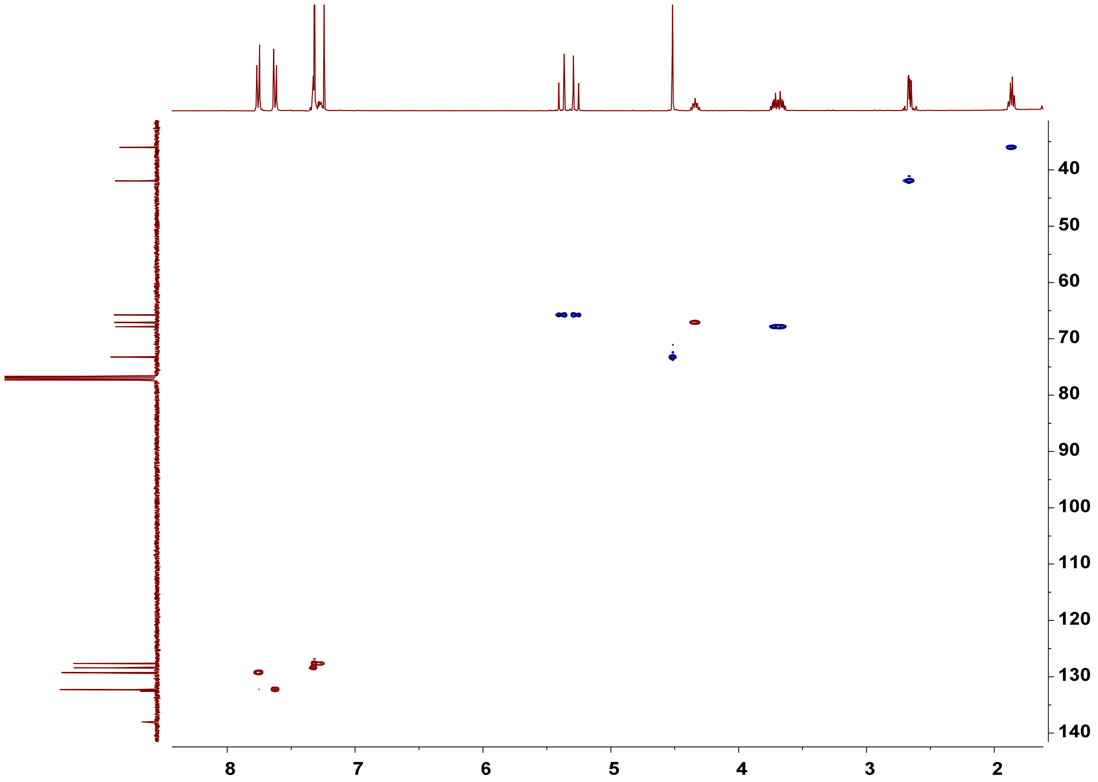

Figure S54. NMR spectra ($CDCl_3$) of *R*-**4m**: a) ^{1}H, b) ^{13}C, c) COSY and d) HSQC.

NMR spectra of α-amino-γ-butyrolactone derivatives.

a)

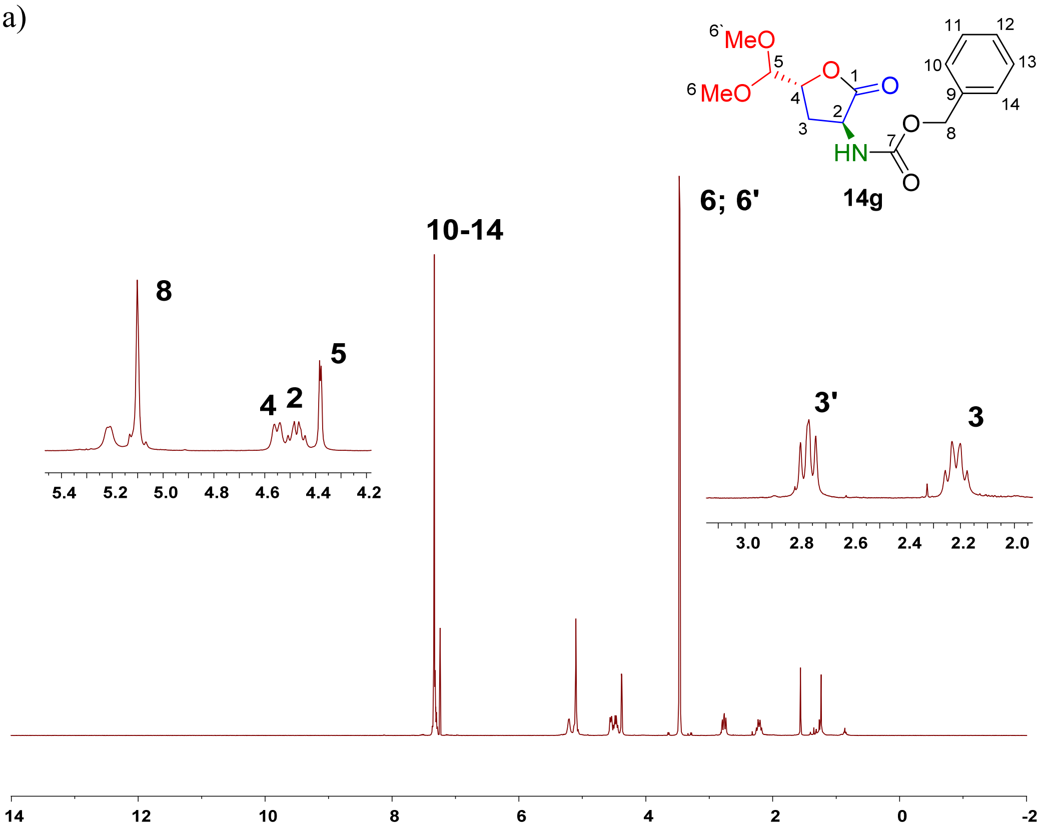

b)

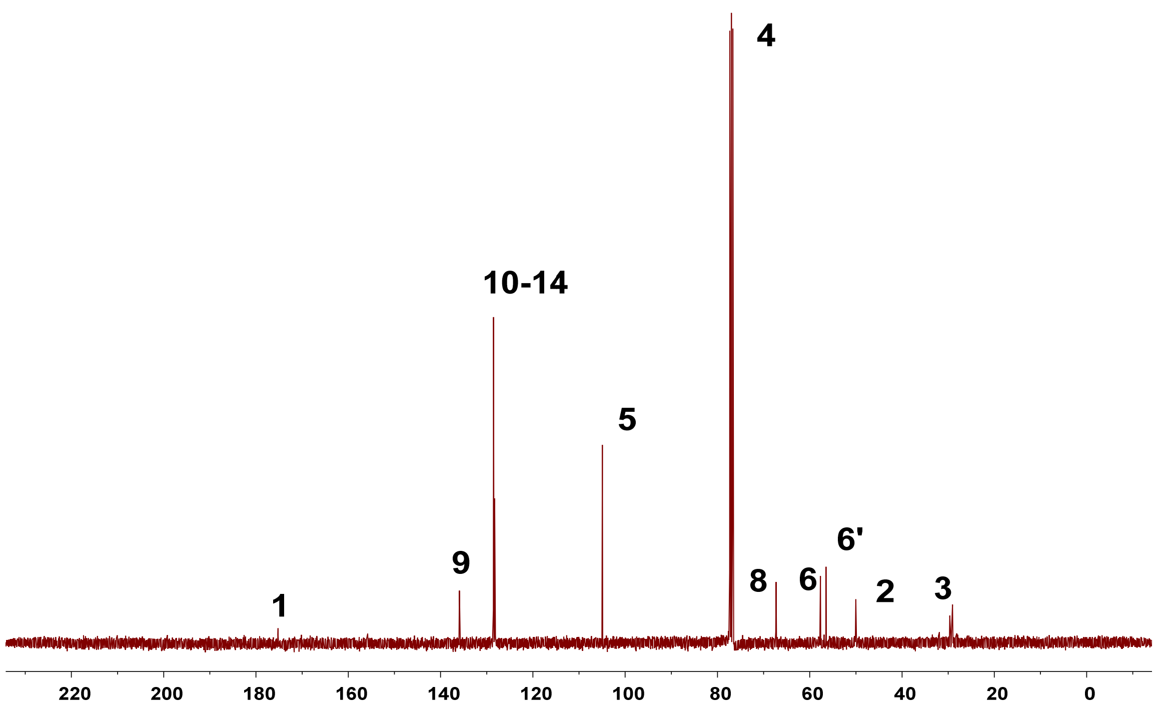

c)

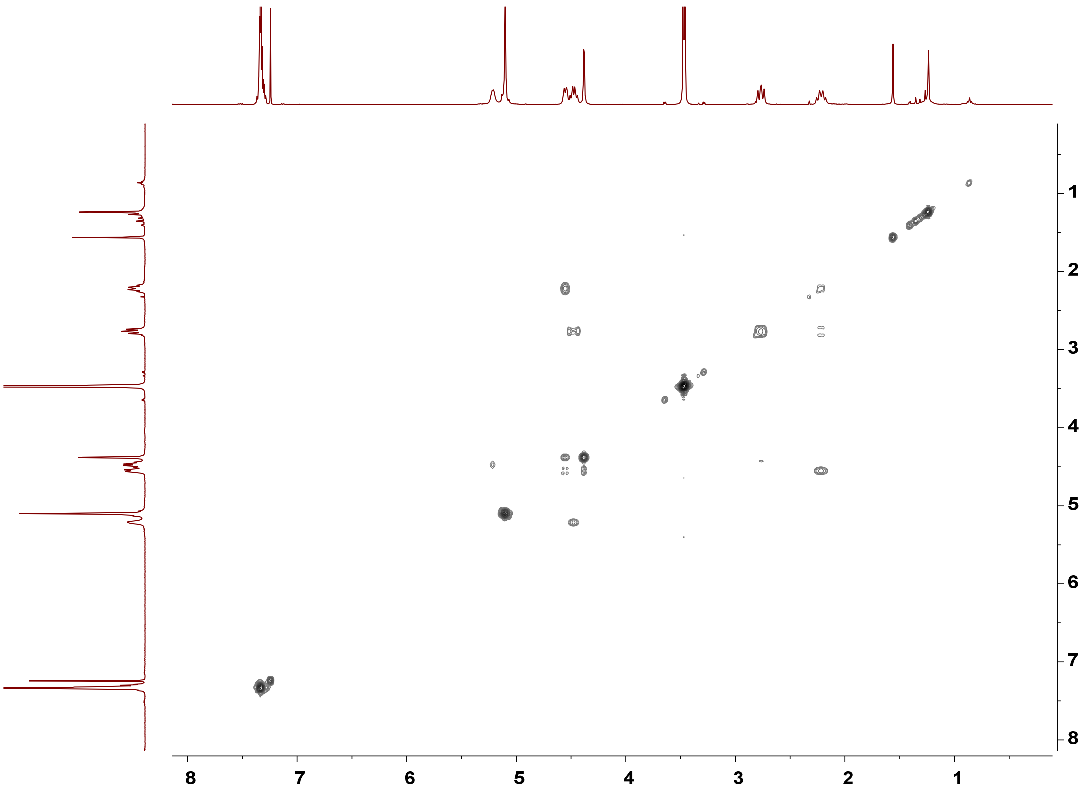
1
2
3
4
5
6
7
8
8
7
6
5
4
3
2
1

d)

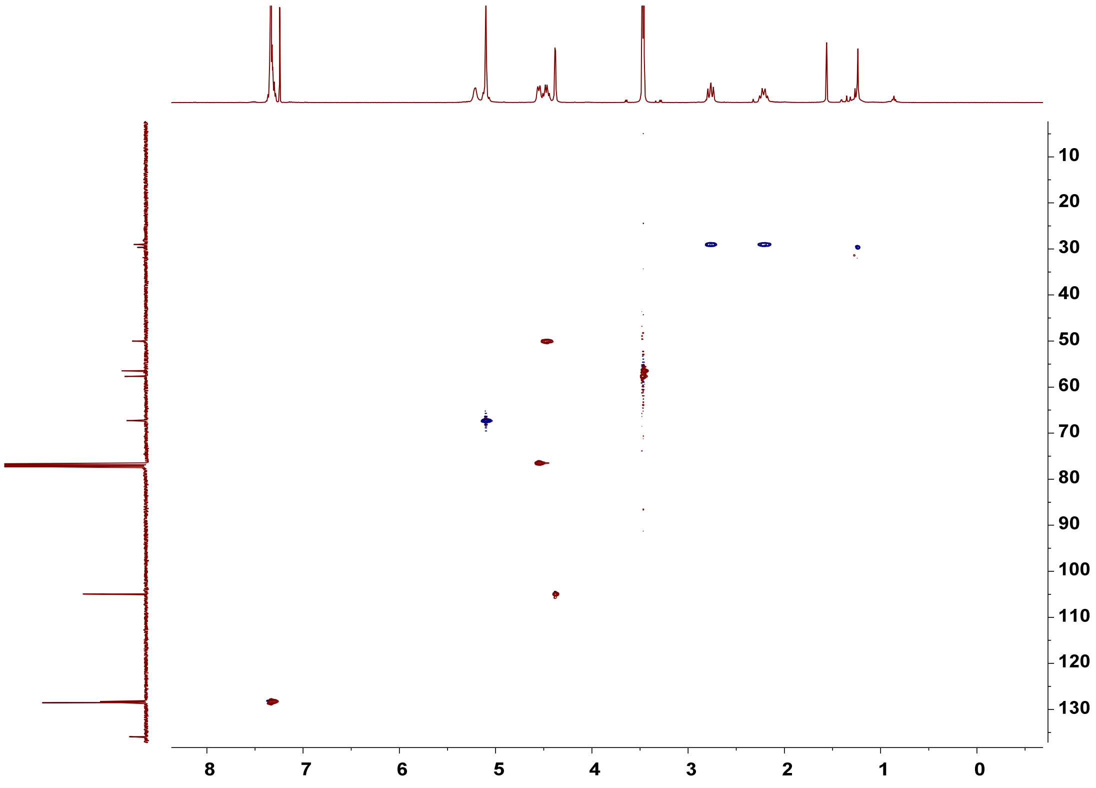
10
20
30
40
50
60
70
80
90
100
110
120
130
8
7
6
5
4
3
2
1
0

e)

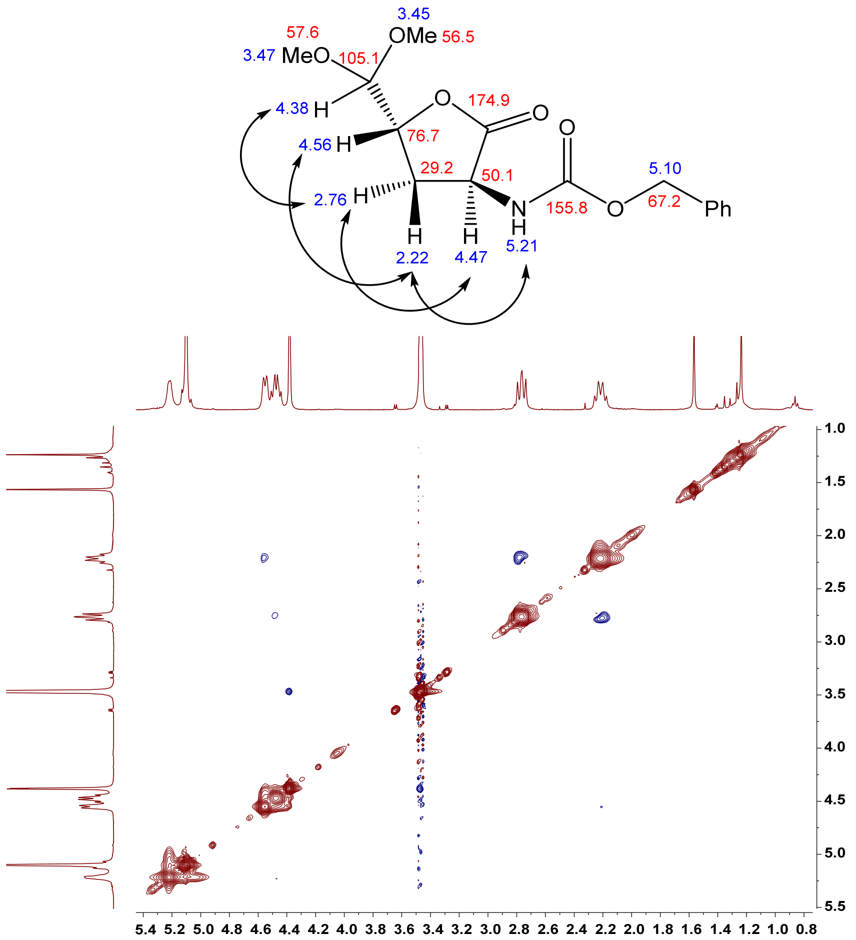

f)

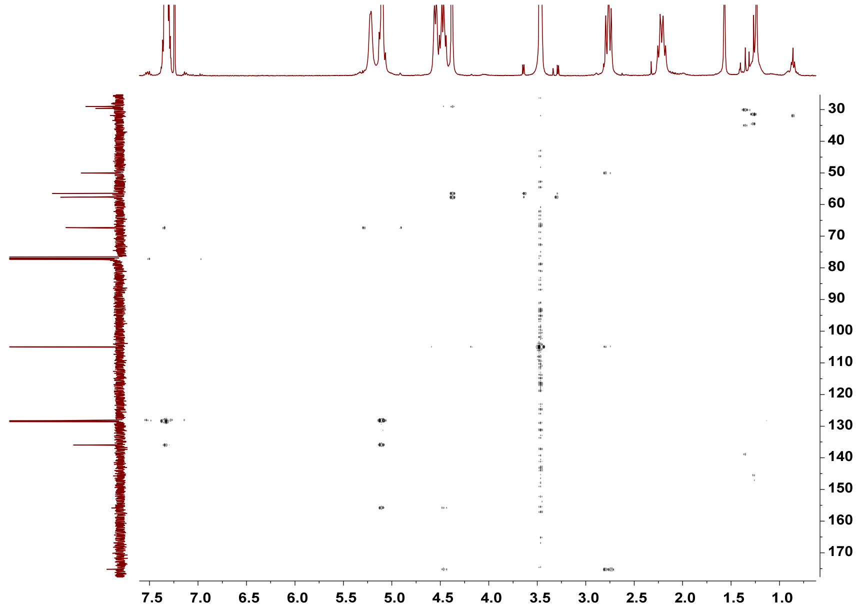

Figure S55. NMR spectra ($CDCl_3$) of **14g**: a) ^1H, b) ^{13}C, c) COSY, d) HSQC, e) NOESY and f) HMBC.

a)

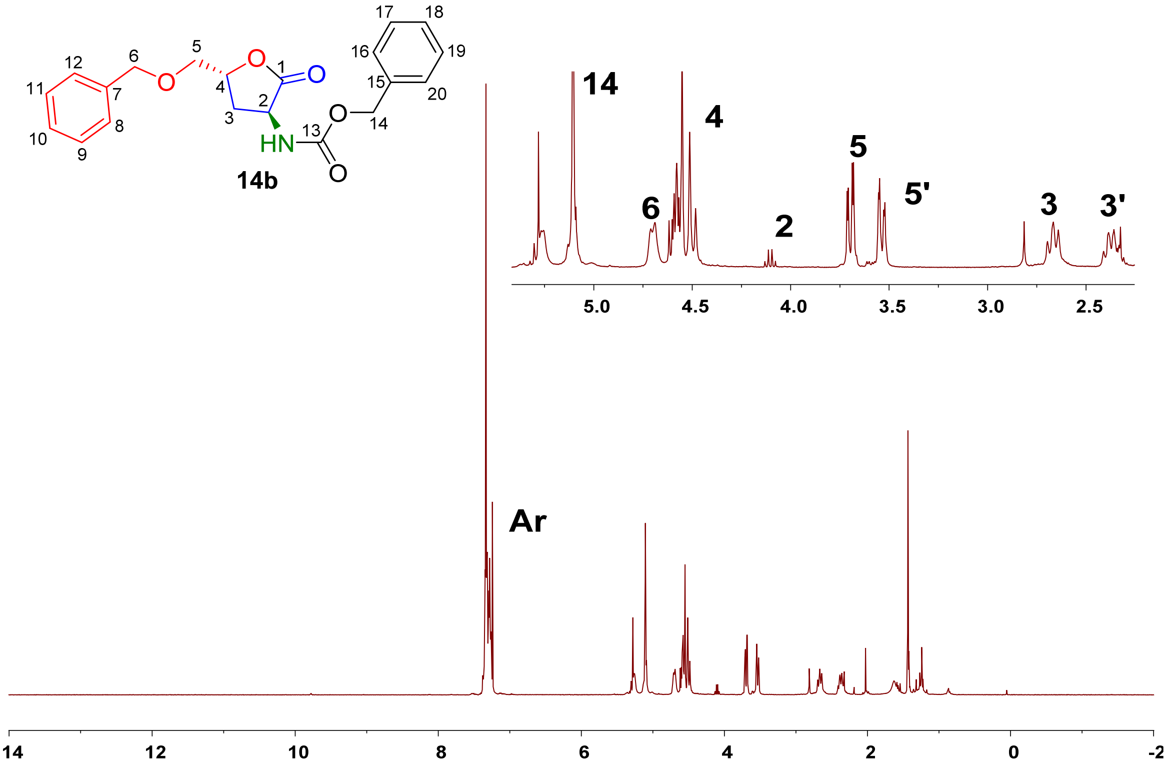

14b
HN
O
14
4
6
2
5
5'
3
3'
5.0
4.5
4.0
3.5
3.0
2.5
Ar
14
12
10
8
6
4
2
0
-2

b)

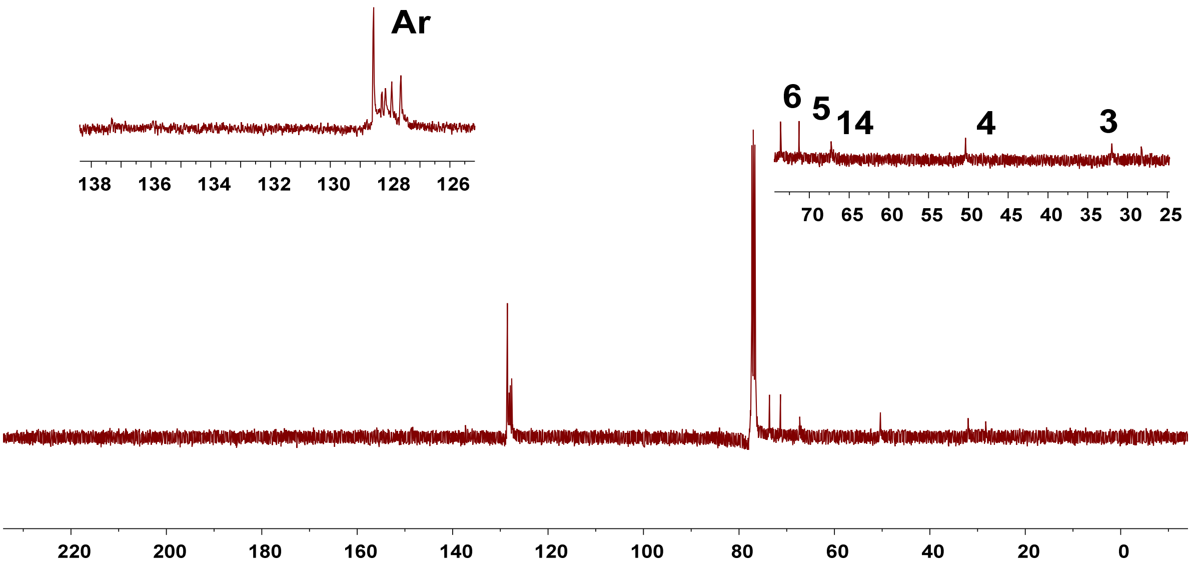

Ar
138
136
134
132
130
128
126
6
5
14
4
3
70
65
60
55
50
45
40
35
30
25
220
200
180
160
140
120
100
80
60
40
20
0

c)

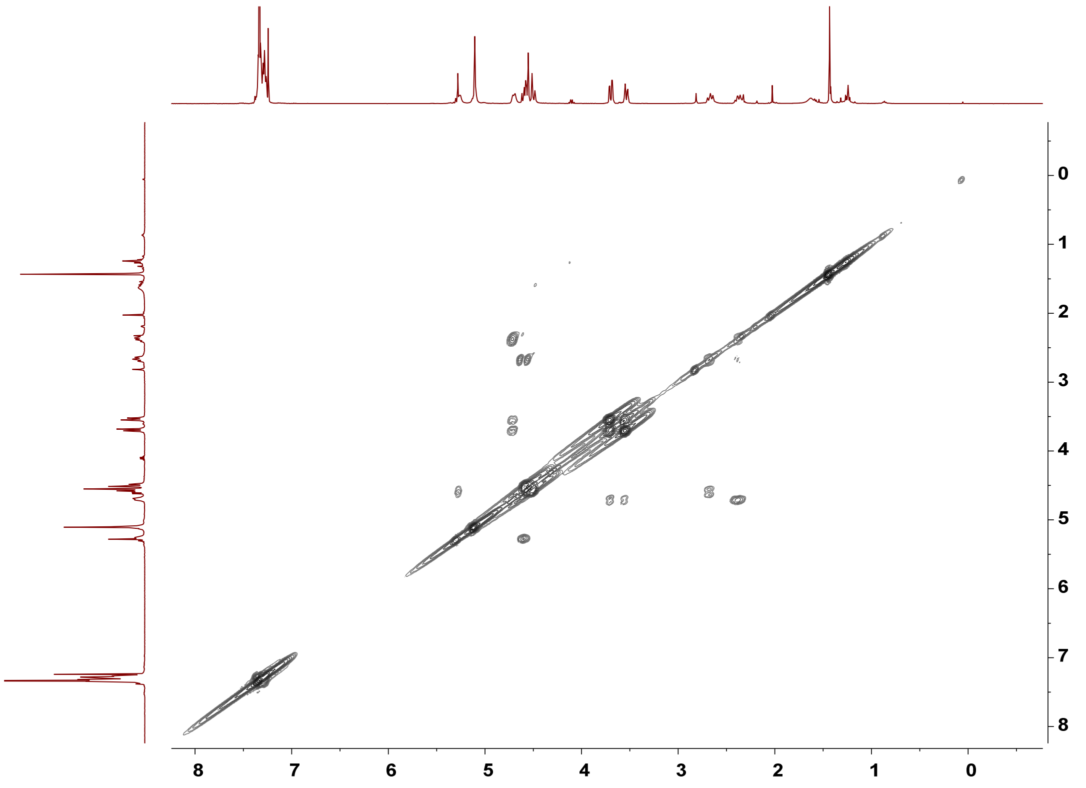
0
1
2
3
4
5
6
7
8
8
7
6
5
4
3
2
1
0

d)

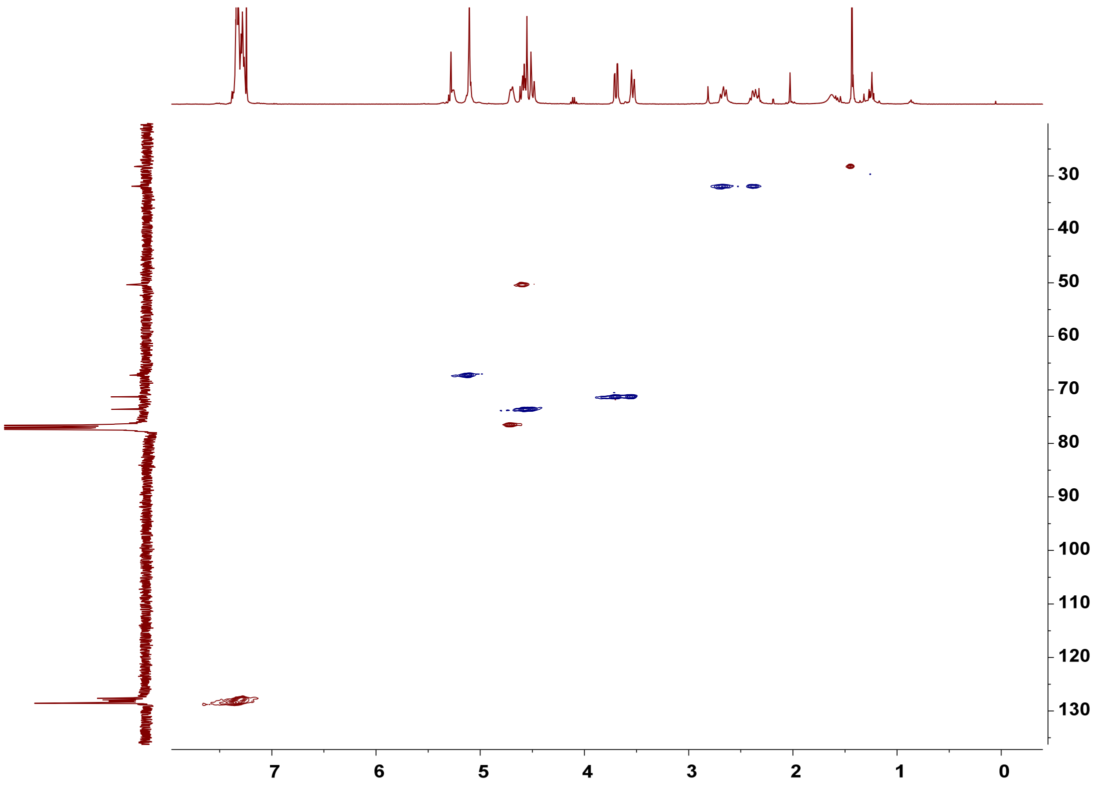
30
40
50
60
70
80
90
100
110
120
130
7
6
5
4
3
2
1
0

e)

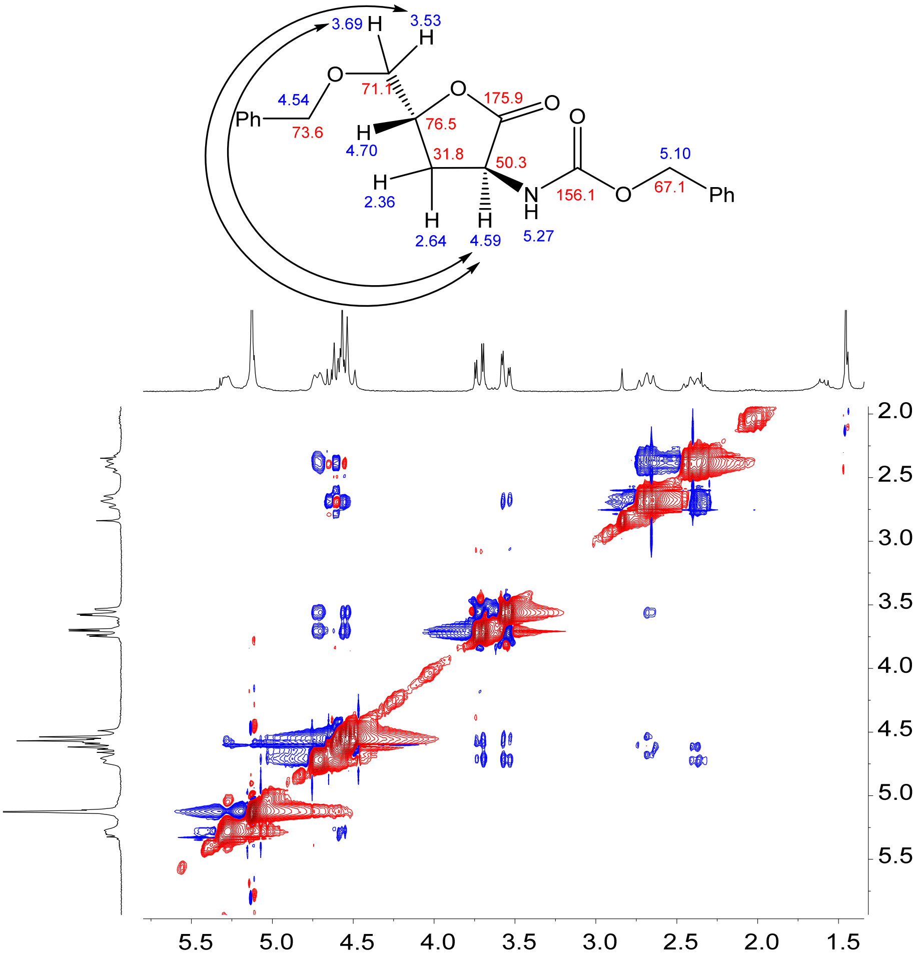

f)

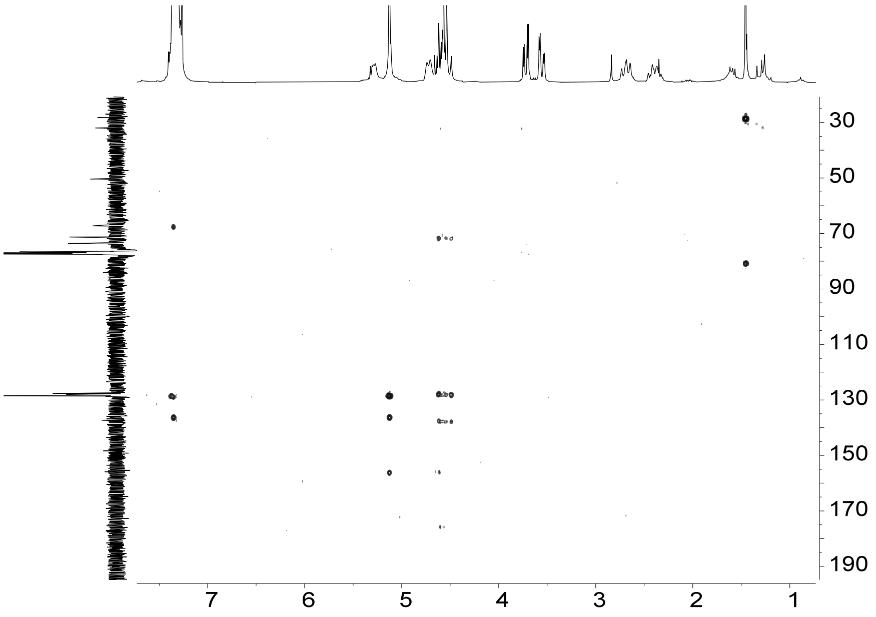

Figure S56. NMR spectra ($CDCl_3$) of **14b**: a) ^1H, b) ^{13}C, c) COSY, d) HSQC, e) NOESY and f) HMBC.

a)

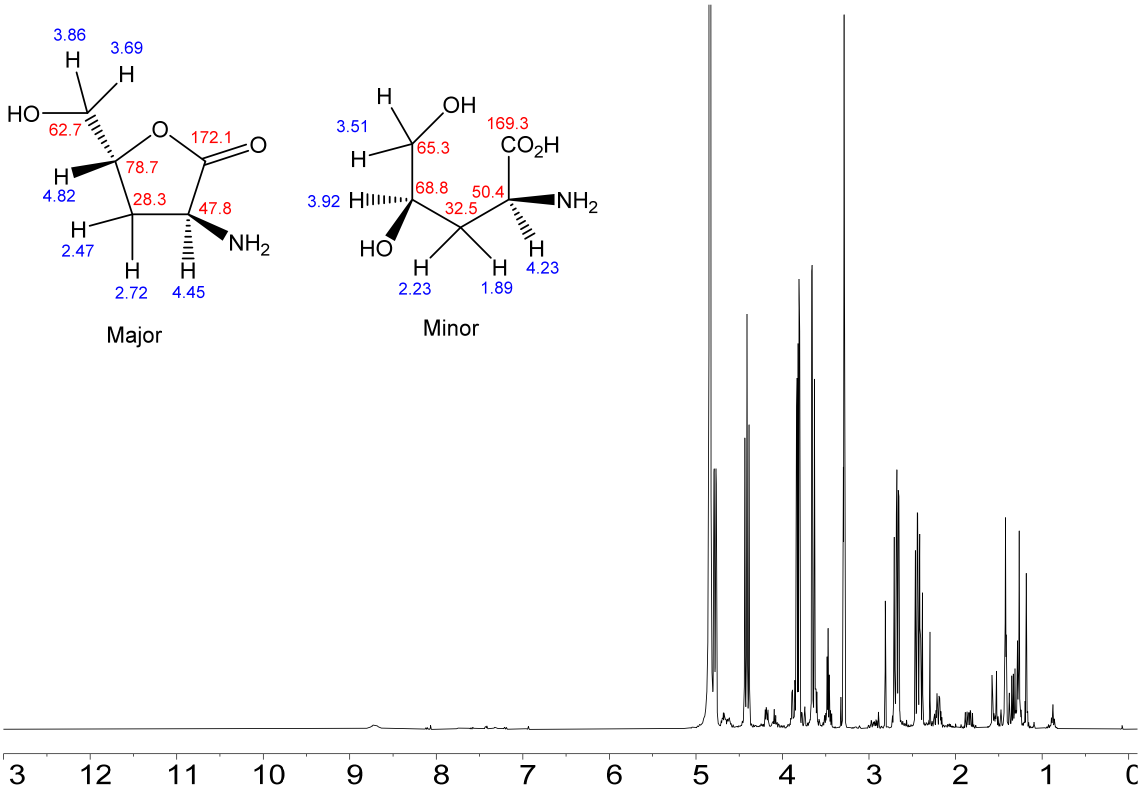
3.86
3.69
H
H
HO
62.7
O
172.1
O
H
78.7
4.82
28.3
47.8
H
NH2
2.47
H
H
2.72
4.45
Major
H
OH
3.51
169.3
65.3
CO2H
H
3.92
H
68.8
50.4
NH2
32.5
HO
H
H
H
4.23
2.23
1.89
Minor
3
12
11
10
9
8
7
6
5
4
3
2
1

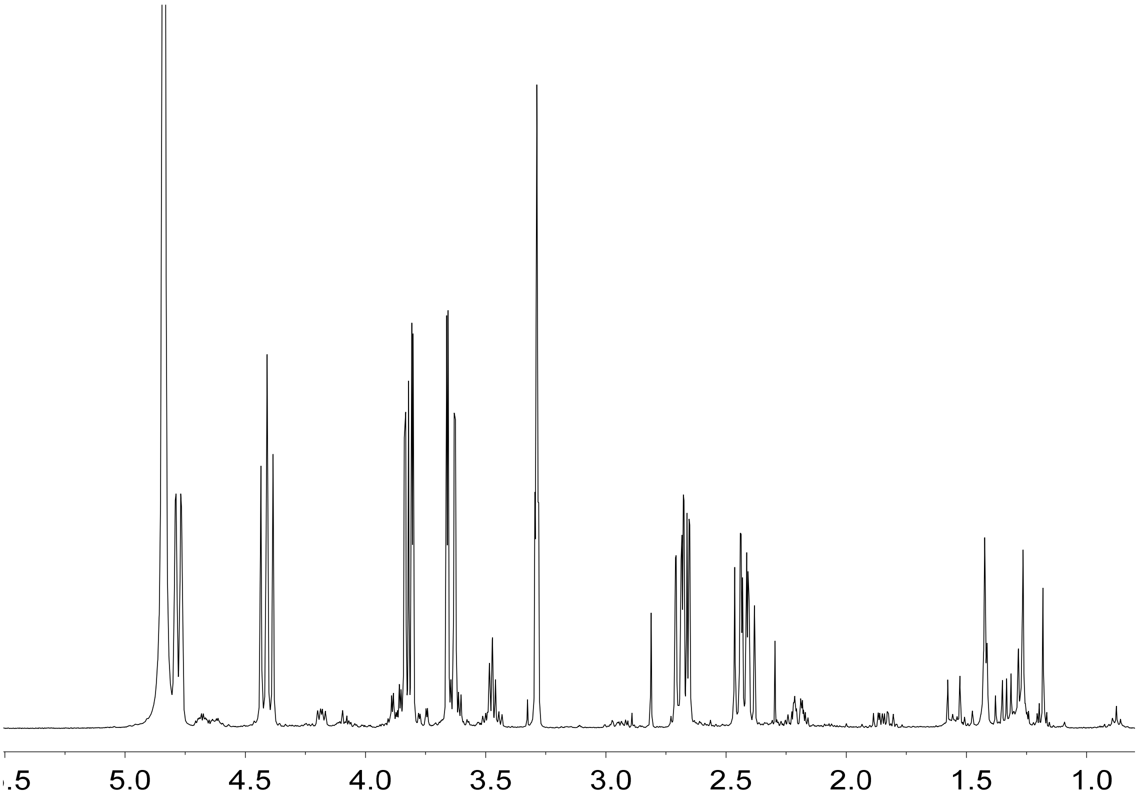
5.0
4.5
4.0
3.5
3.0
2.5
2.0
1.5
1.0

b)

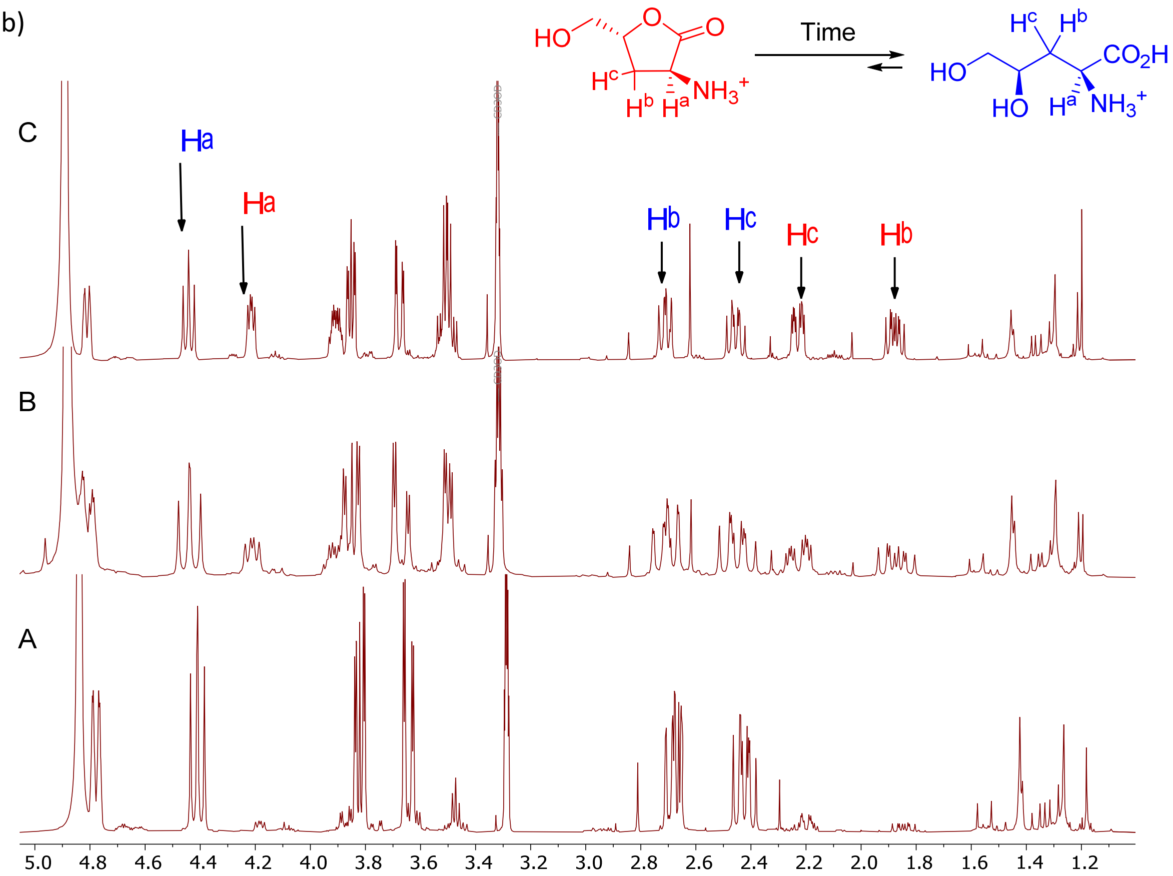

Time
C
B
A
Ha
Ha
Hb
Hc
Hc
Hb
5.0
4.8
4.6
4.4
4.2
4.0
3.8
3.6
3.4
3.2
3.0
2.8
2.6
2.4
2.2
2.0
1.8
1.6
1.4
1.2

c)

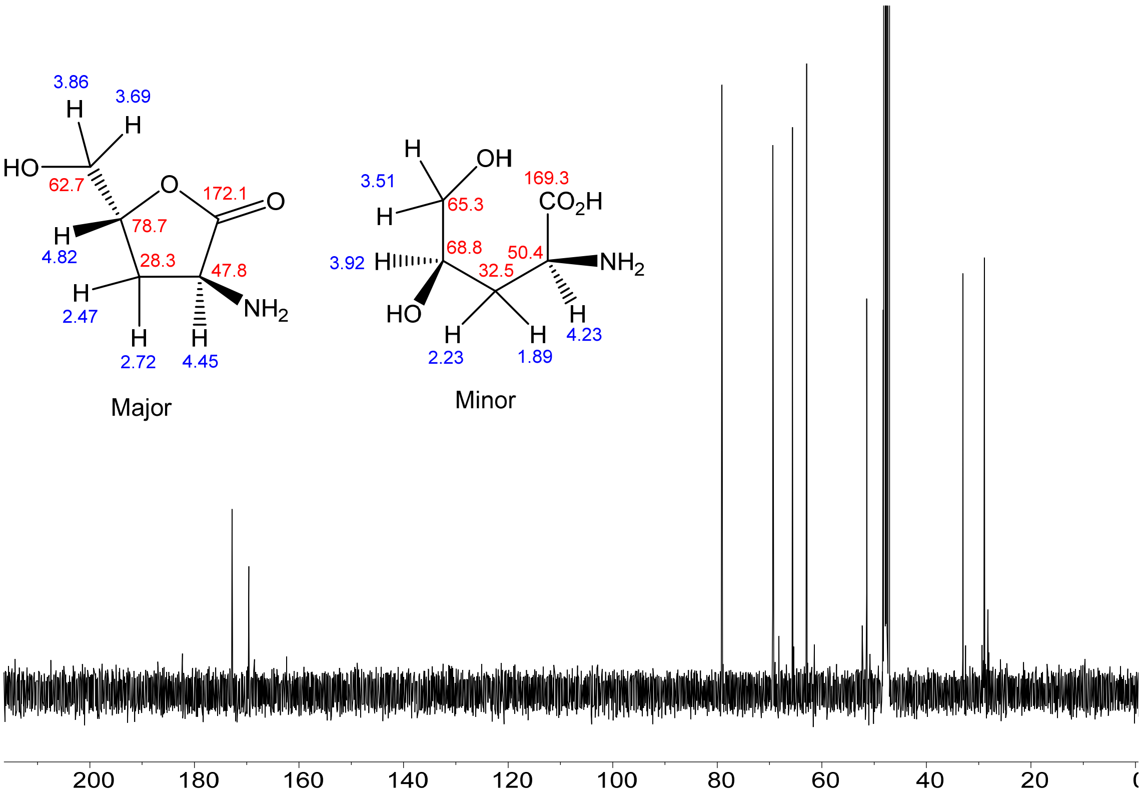

Major
Minor
3.86
3.69
62.7
172.1
78.7
4.82
28.3
47.8
2.47
2.72
4.45
3.51
169.3
65.3
3.92
68.8
50.4
32.5
2.23
1.89
4.23
200
180
160
140
120
100
80
60
40
20

d)

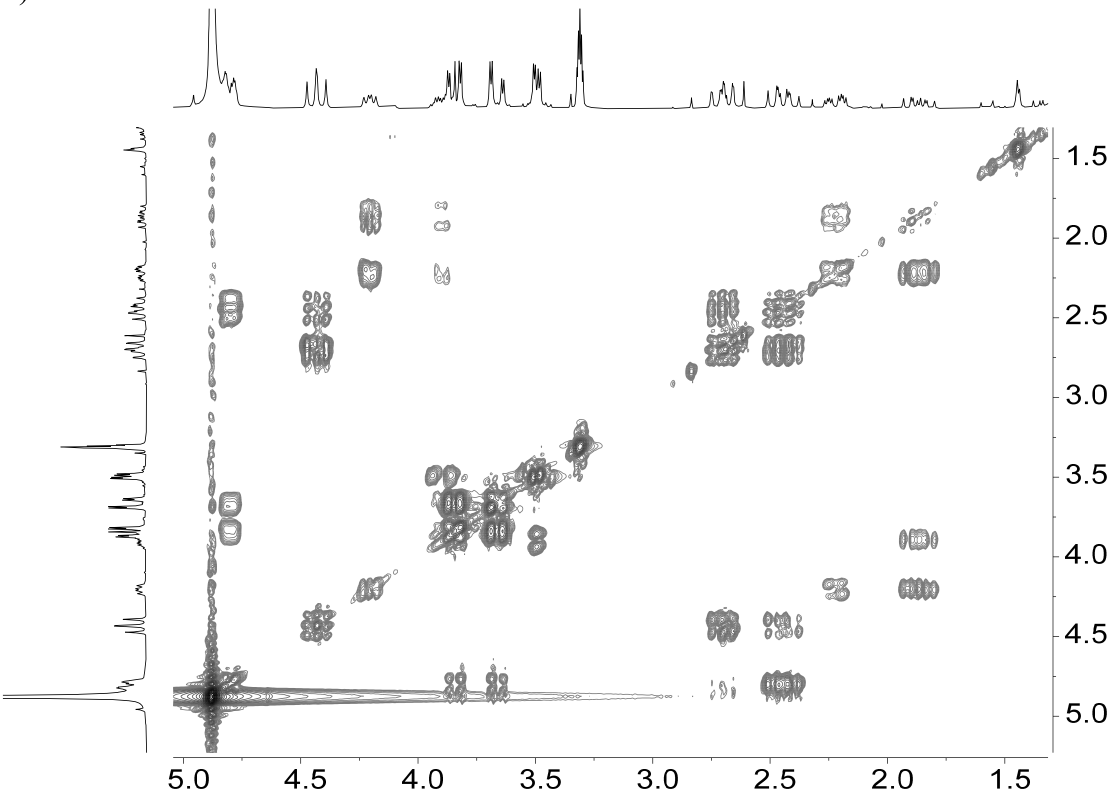

1.5
2.0
2.5
3.0
3.5
4.0
4.5
5.0
5.0
4.5
4.0
3.5
3.0
2.5
2.0
1.5

e)

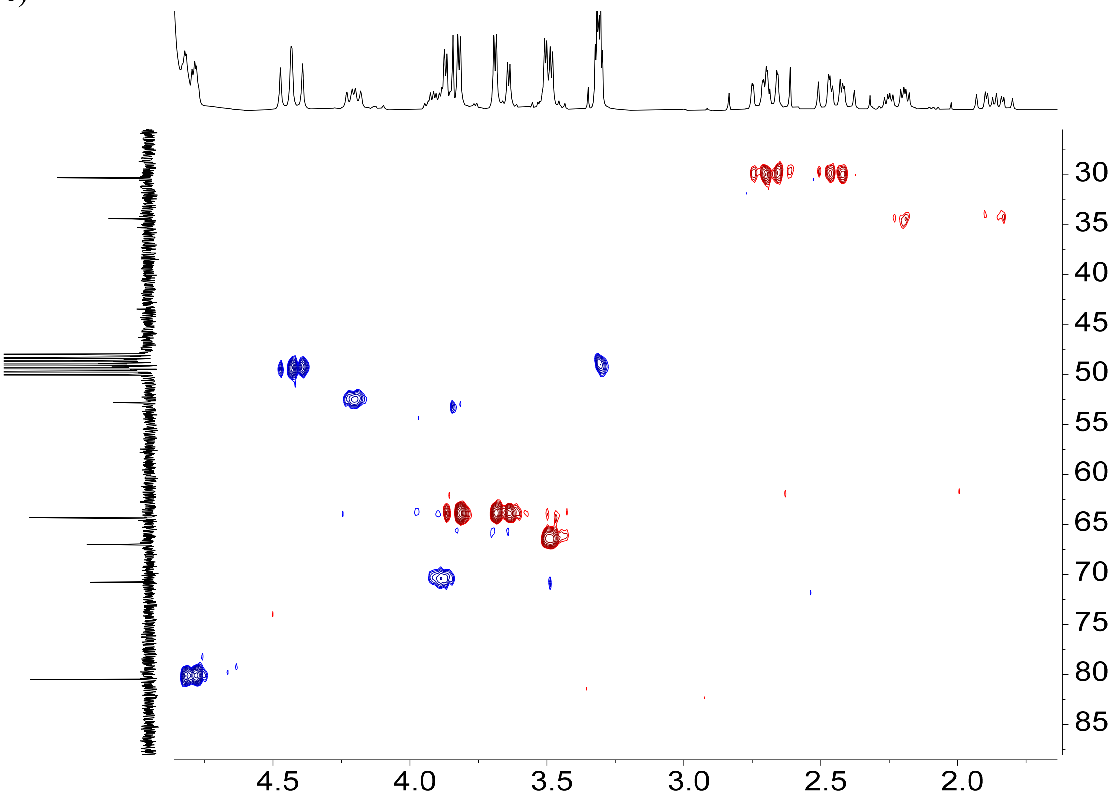

30
35
40
45
50
55
60
65
70
75
80
85
4.5
4.0
3.5
3.0
2.5
2.0

f)

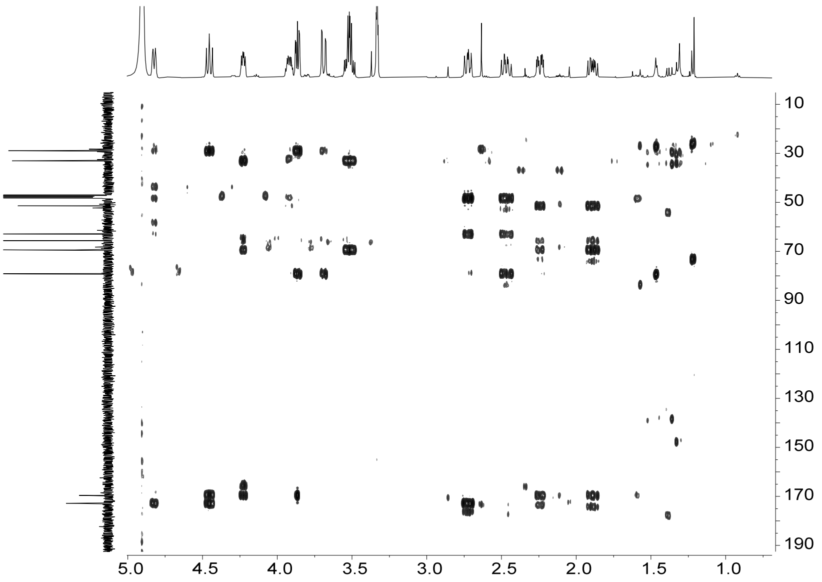

g)

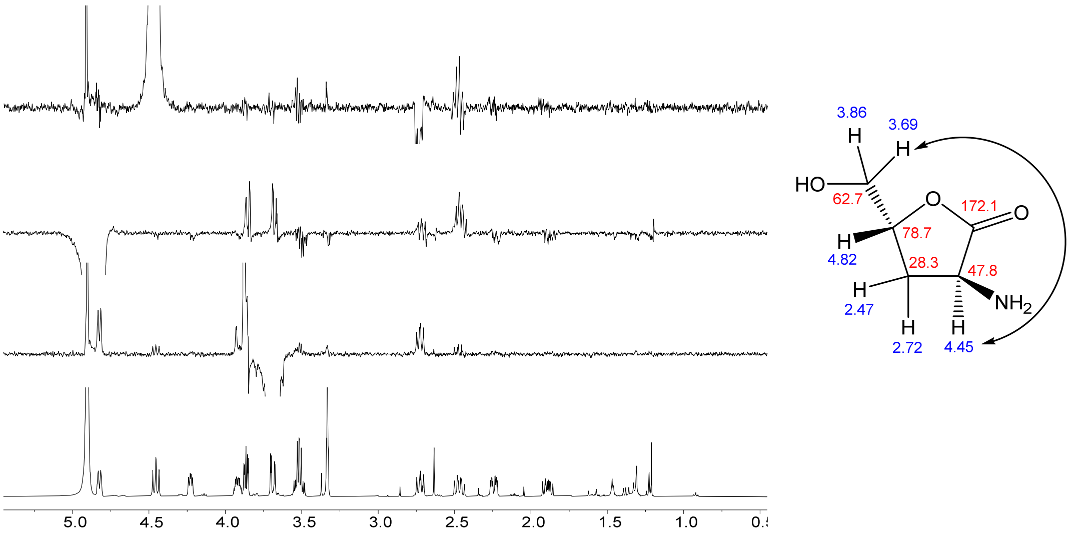

Figure S57. NMR spectra (CD3OD) of **14a**: a) ^{1}H (Initial spectra, 400 MHz), b) ^{1}H (The spectra of the sample changed with the time and it was because of the cyclic lactone is hydrolyzed: *i* (Initial spectra, 400 MHz) *ii* (Spectra after 3 months, 250 MHz) *iii* (Spectra after 4 months, 500 MHz) c) ^{13}C, d) COSY, e) HSQC, f) NOESY, g) HMBC (c-g 500 MHz) and g) Selective 1D nOe (500 MHz).

a)

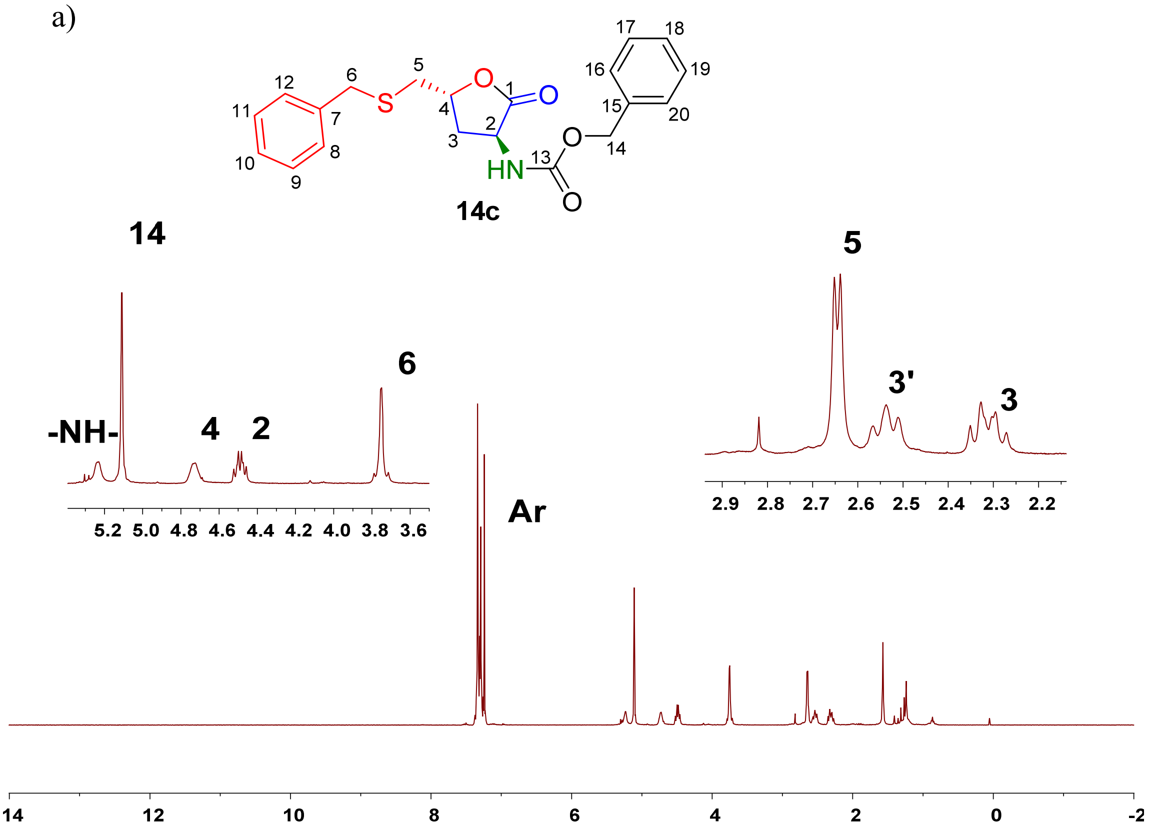

b)

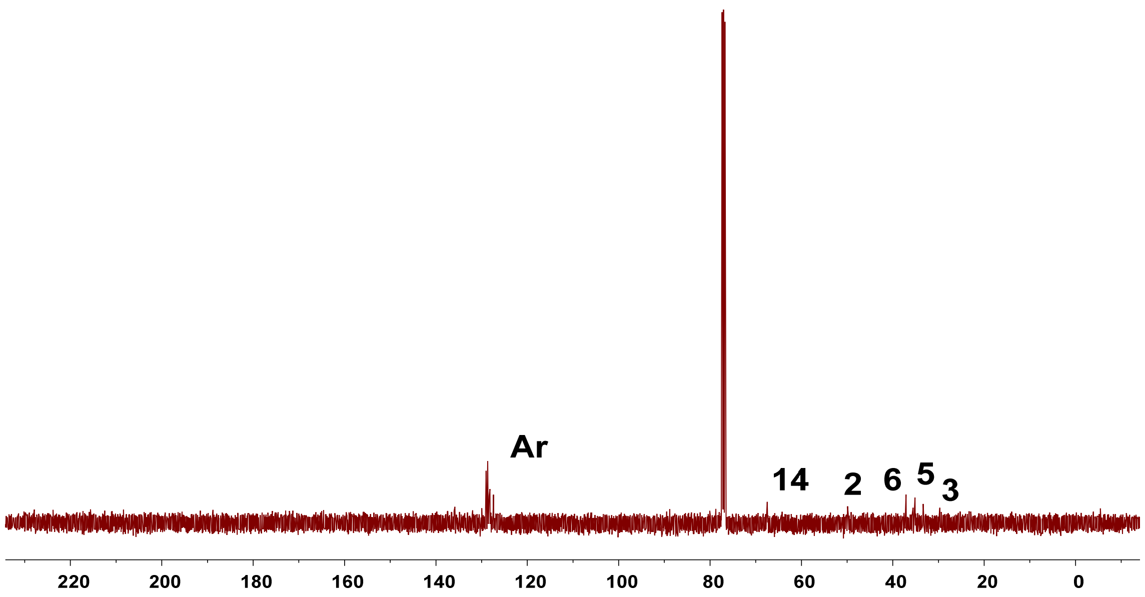

c)

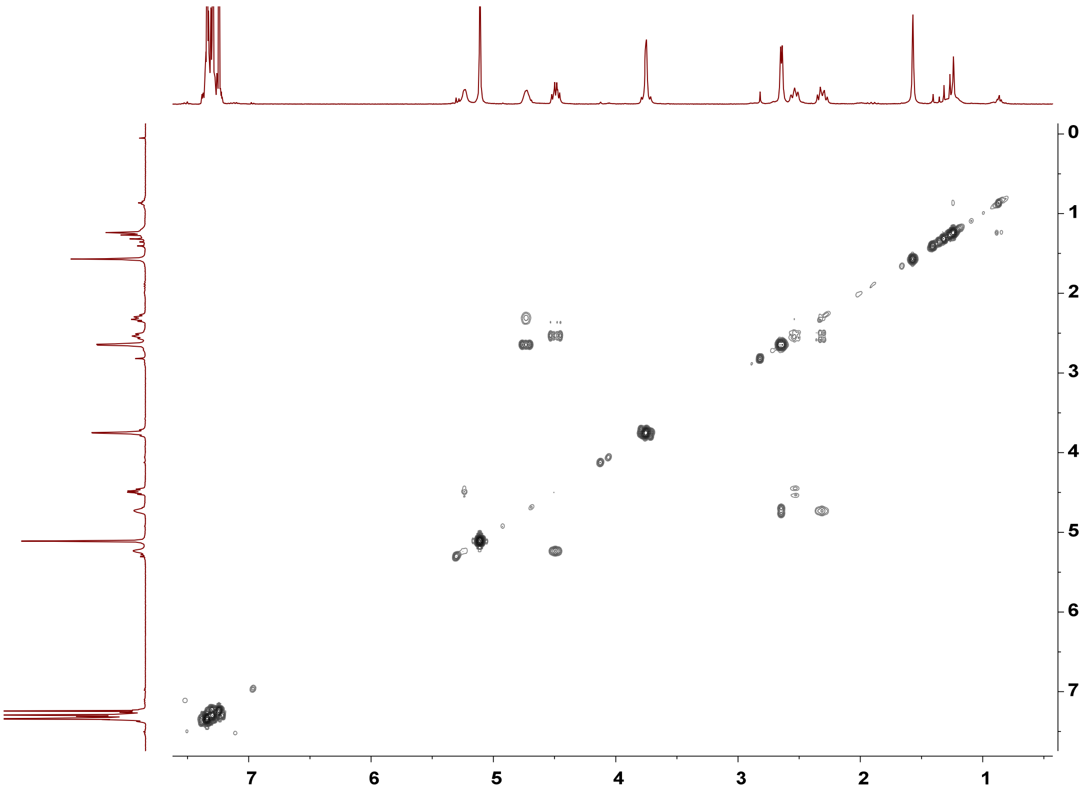

d)

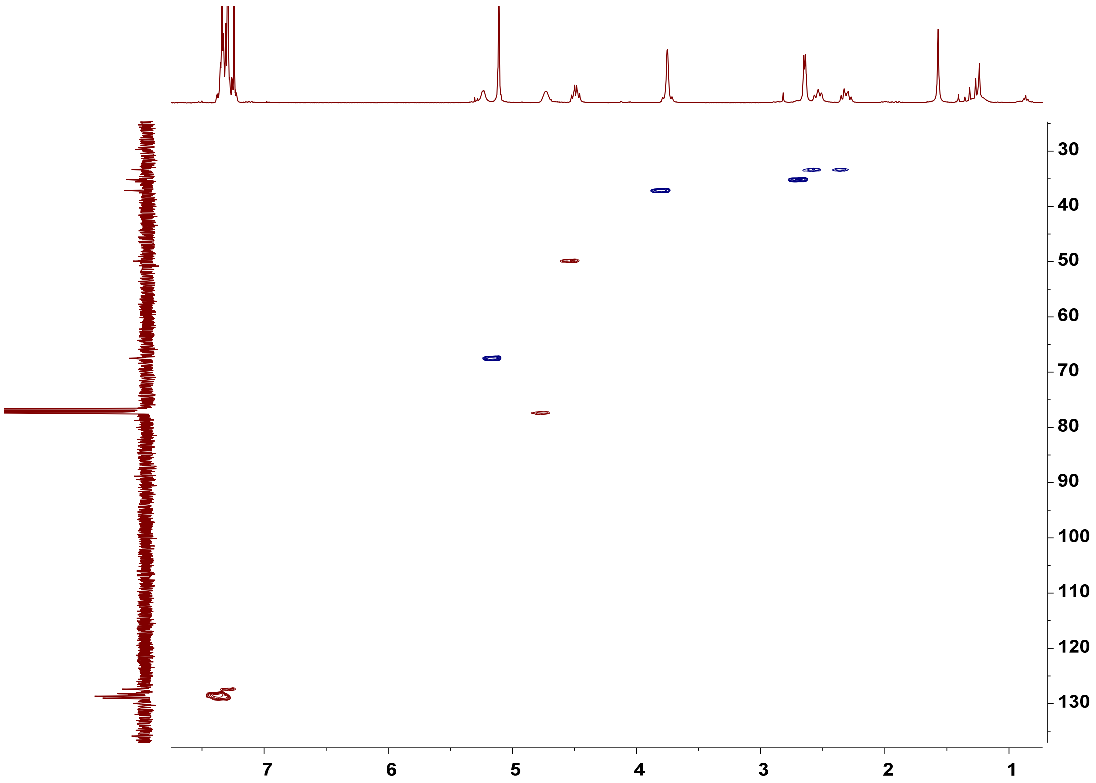

e)

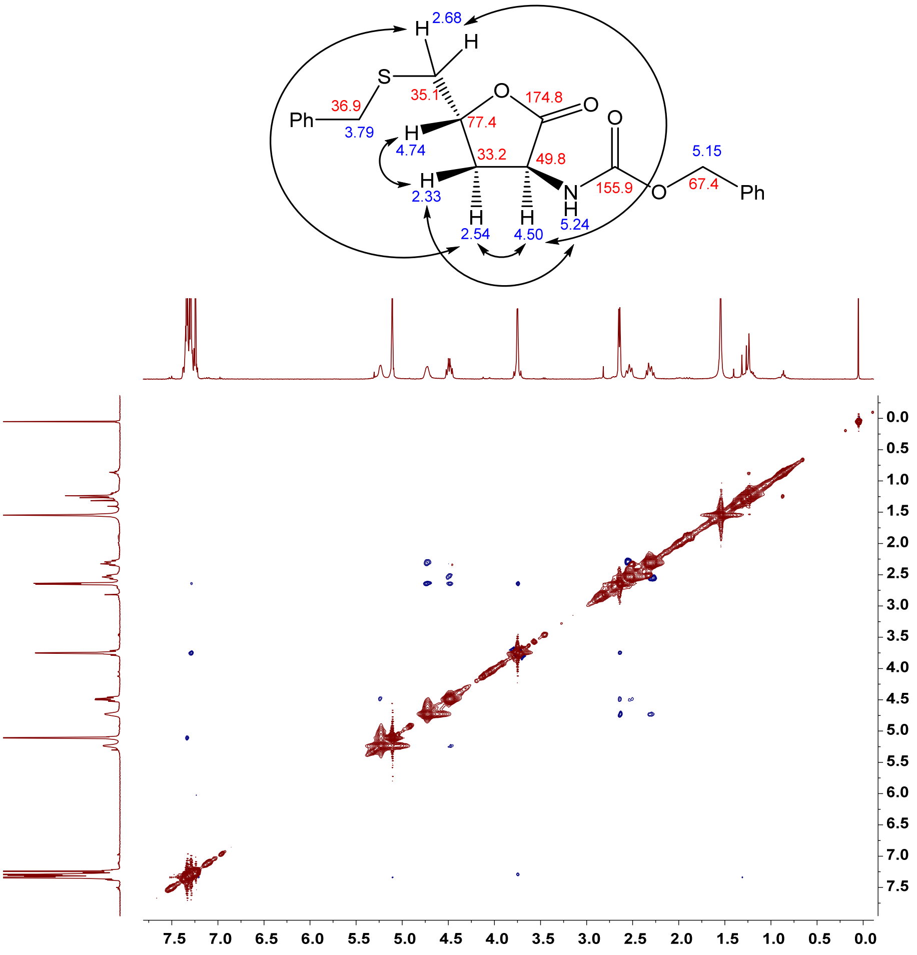

f)

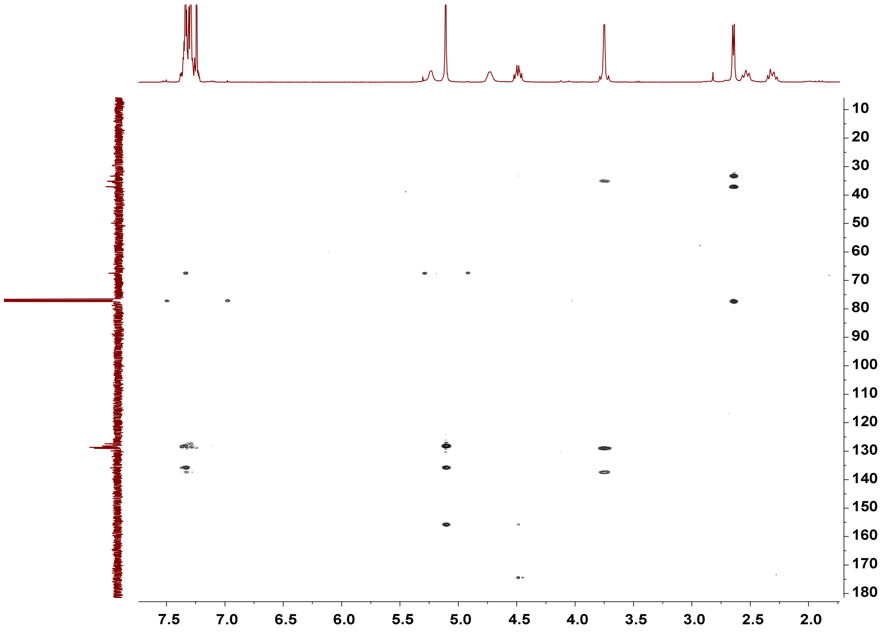

Figure S58. NMR spectra (CDCl3) of **14c**: a) ^{1}H, b)^{13}C, c) COSY, d) HSQC, e) NOESY and f) HMBC.

a)

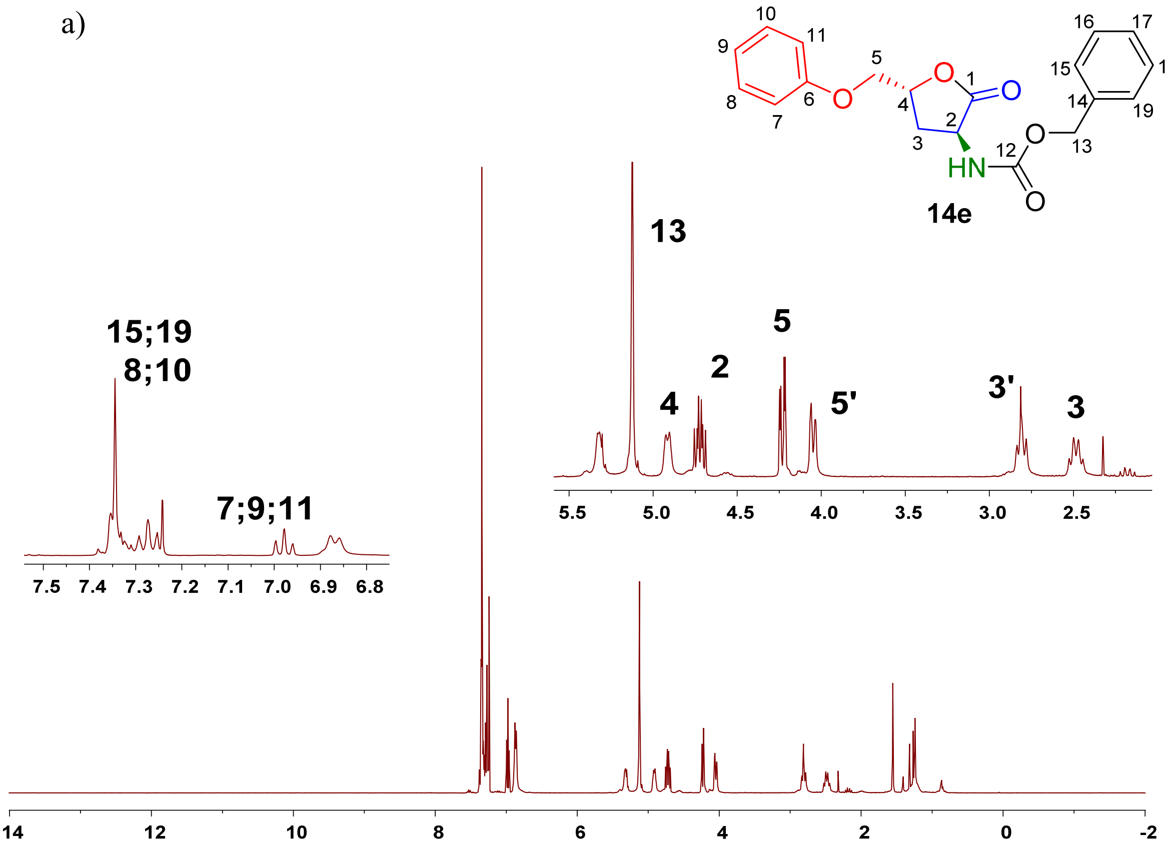

b)

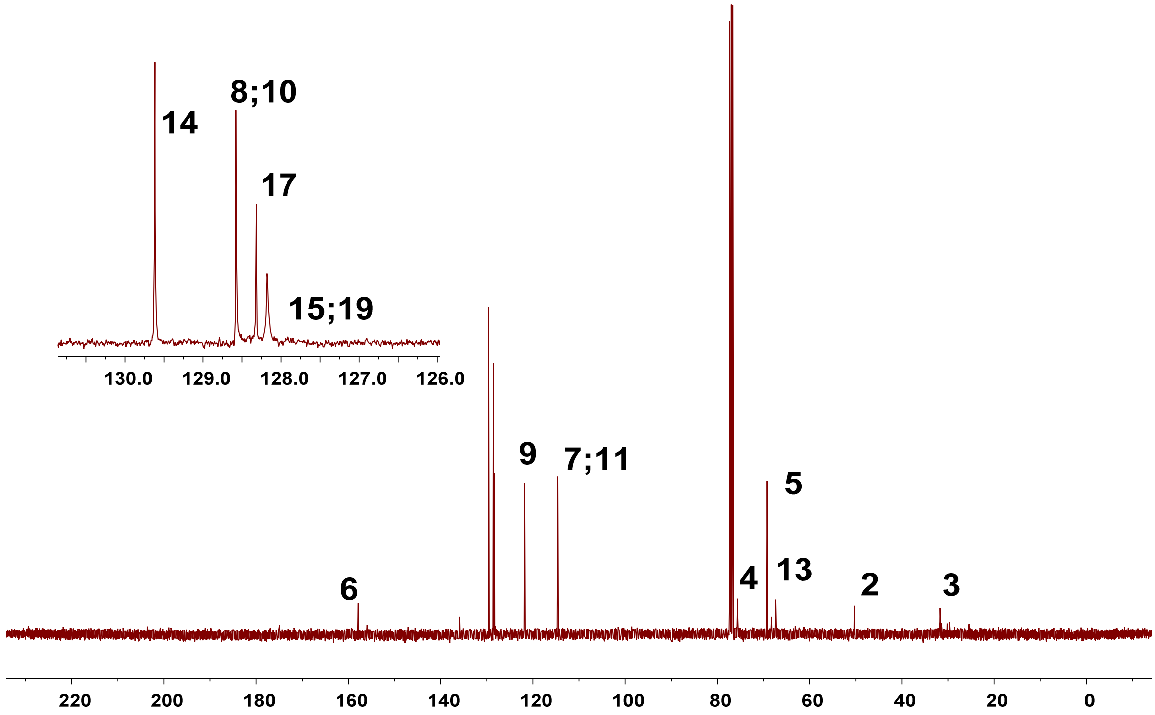

c)

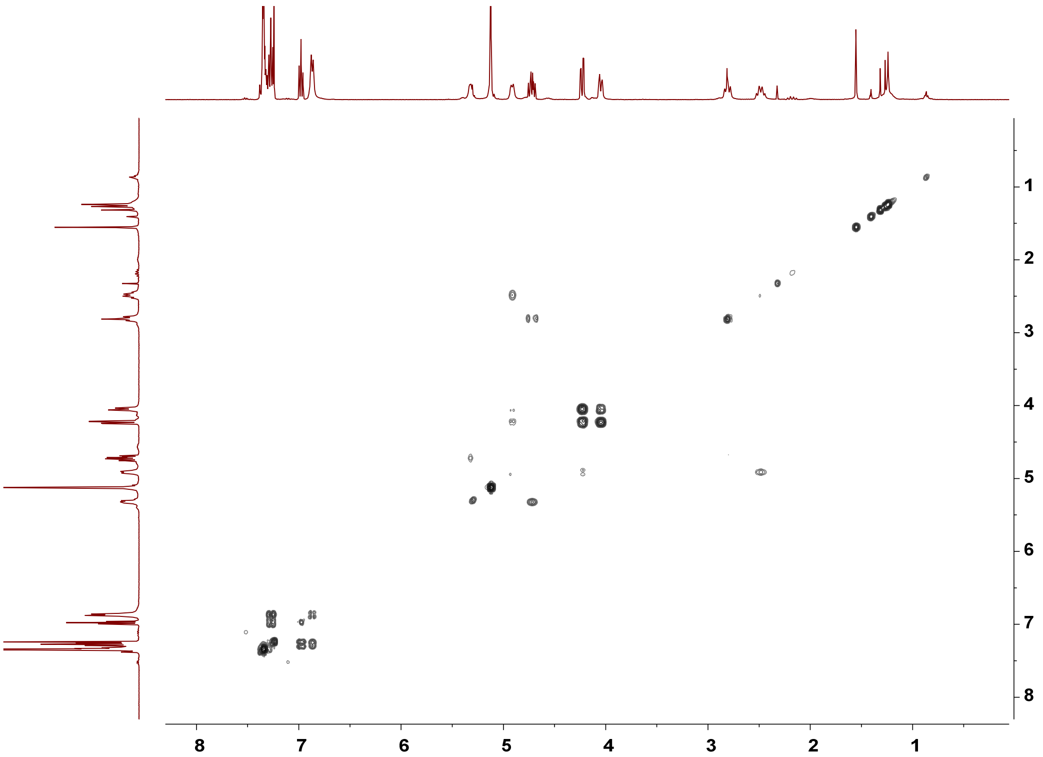
1
2
3
4
5
6
7
8
8
7
6
5
4
3
2
1

d)

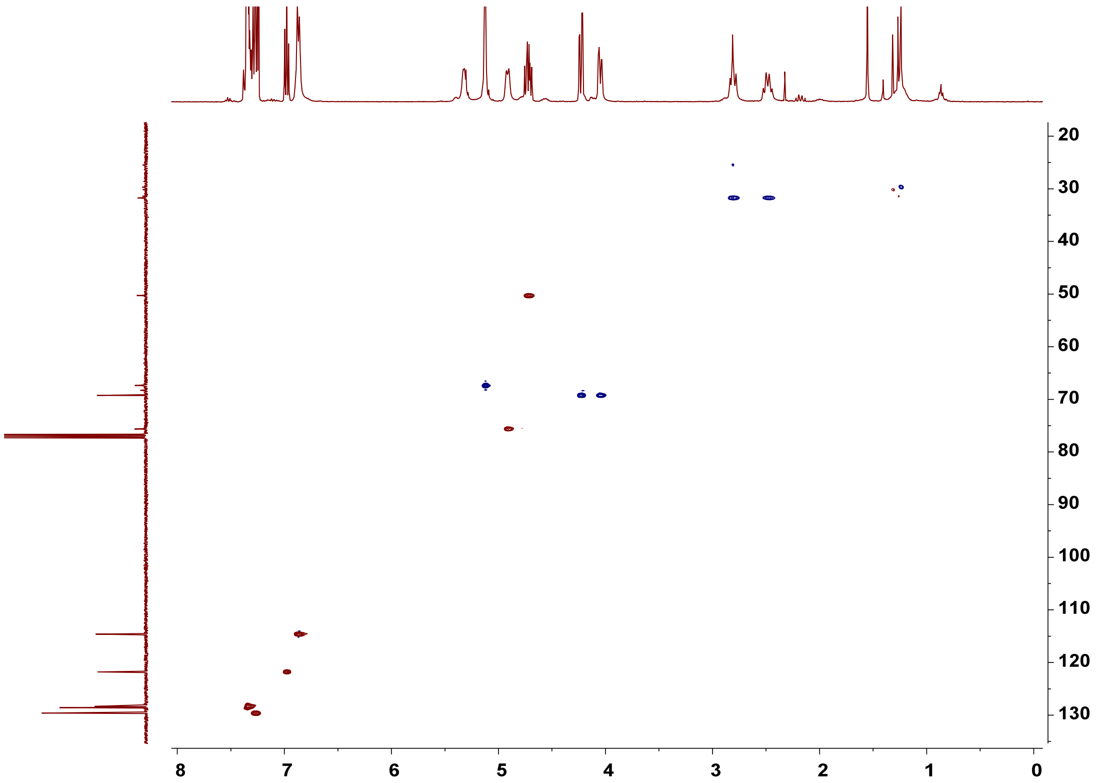
20
30
40
50
60
70
80
90
100
110
120
130
8
7
6
5
4
3
2
1
0

e)

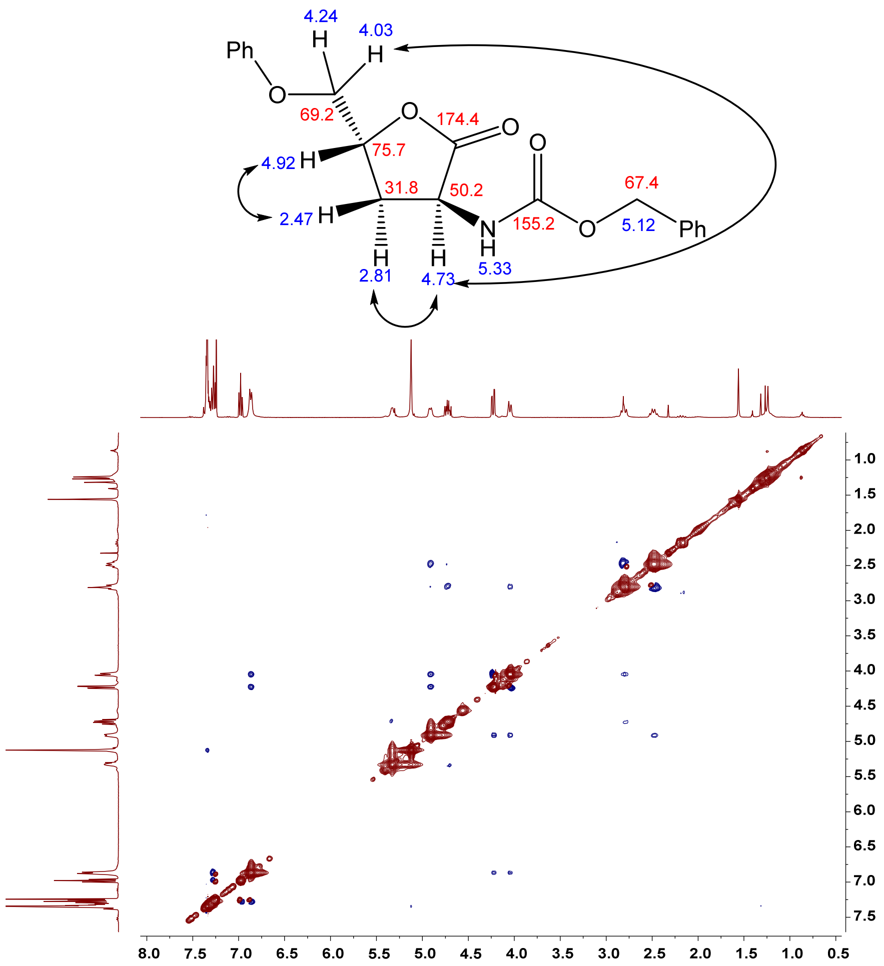

f)

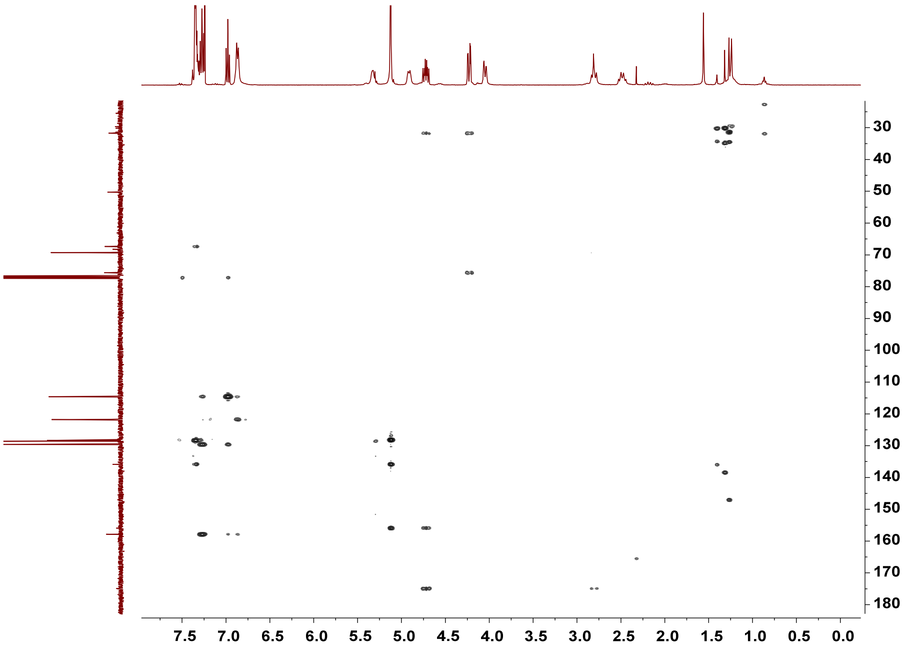

Figure S59. NMR spectra ($CDCl_3$) of **14e**: a) ^{1}H, b) ^{13}C, c) COSY, d) HSQC, e) NOESY and f) HMBC.

a)

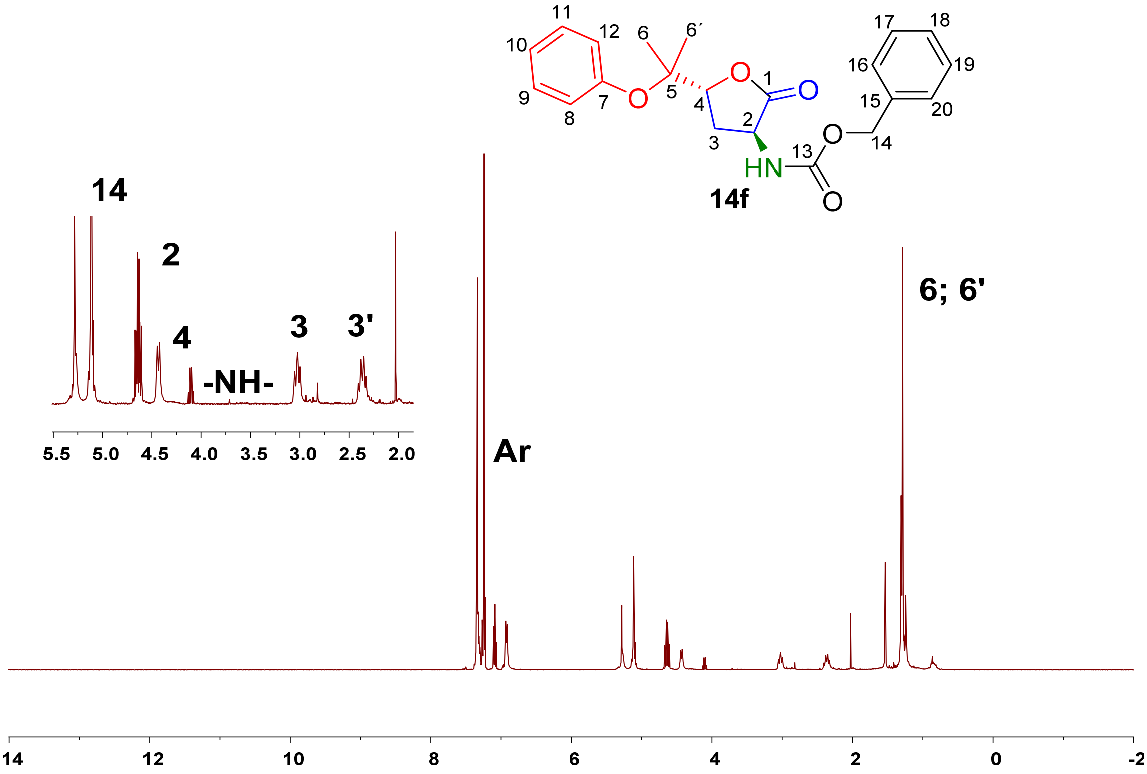

14f
14
2
4
-NH-
3
3'
Ar
6; 6'
5.5 5.0 4.5 4.0 3.5 3.0 2.5 2.0
14 12 10 8 6 4 2 0 -2

b)

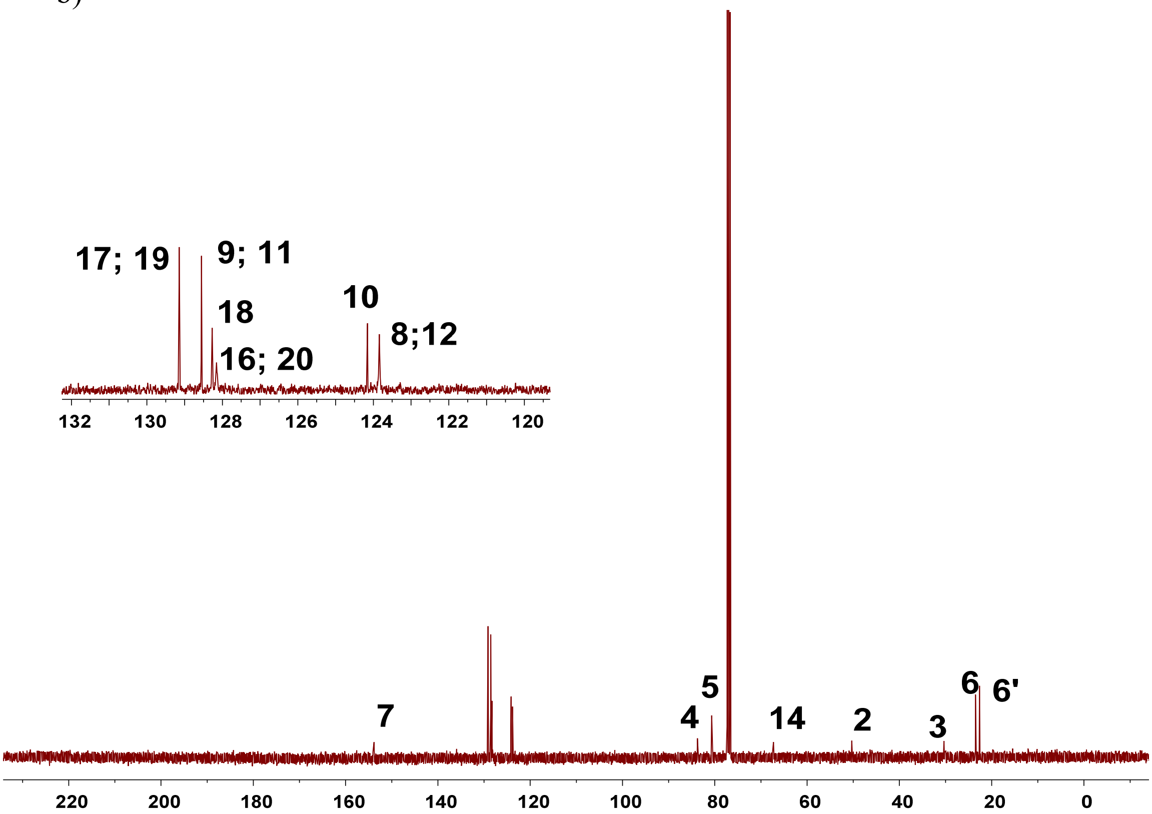

17; 19
9; 11
18
16; 20
10
8;12
132 130 128 126 124 122 120
7
4
5
14
2
3
6
6'
220 200 180 160 140 120 100 80 60 40 20 0

c)

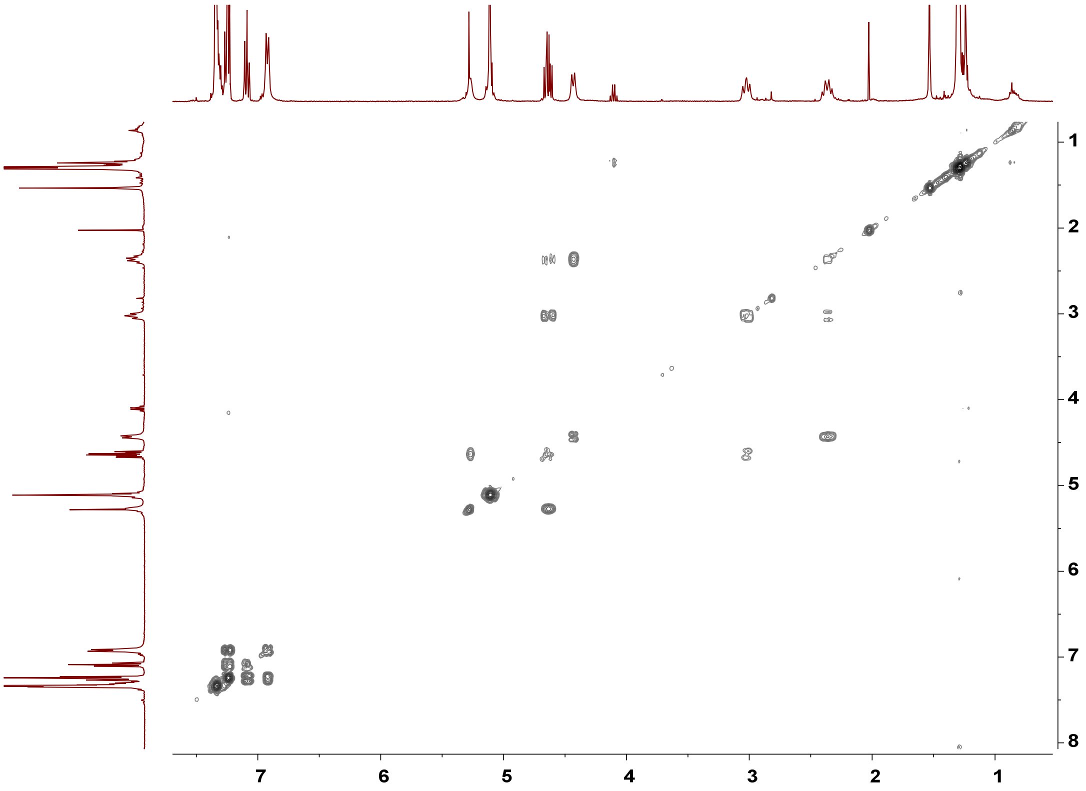
1
2
3
4
5
6
7
8
7
6
5
4
3
2
1

d)

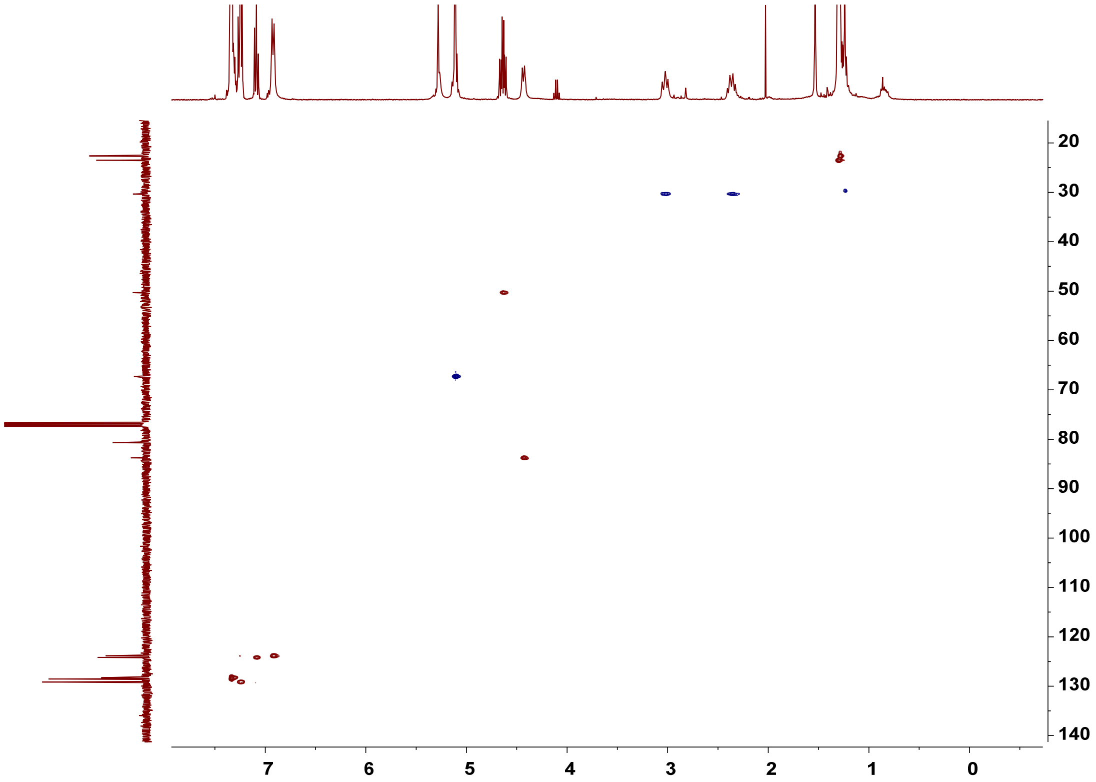
20
30
40
50
60
70
80
90
100
110
120
130
140
7
6
5
4
3
2
1
0

e)

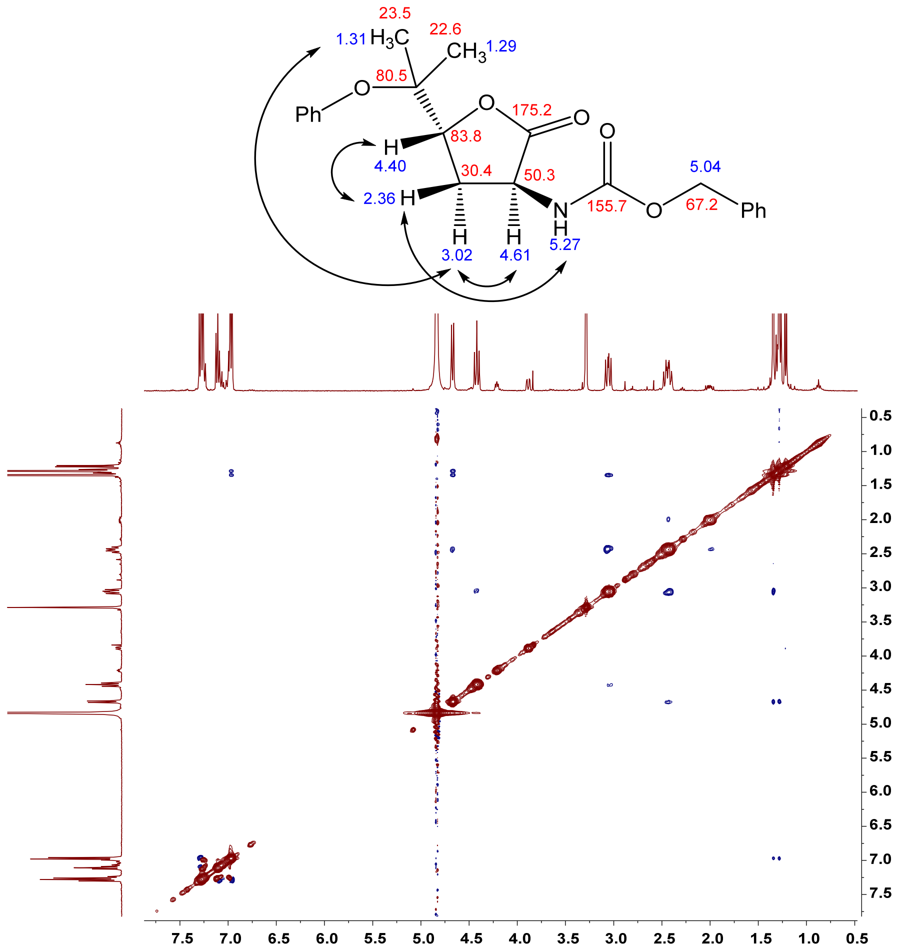

f)

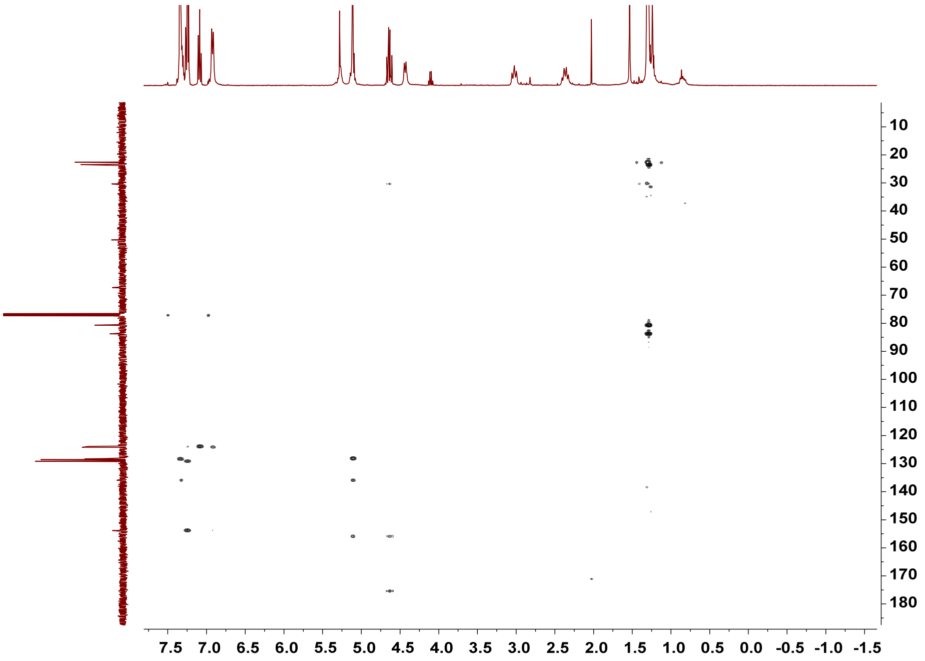

Figure S60. NMR spectra ($CDCl_3$) of **14f**: a) ^1H, b) ^{13}C, c) COSY, d) HSQC, e) NOESY and f) HMBC.

a)

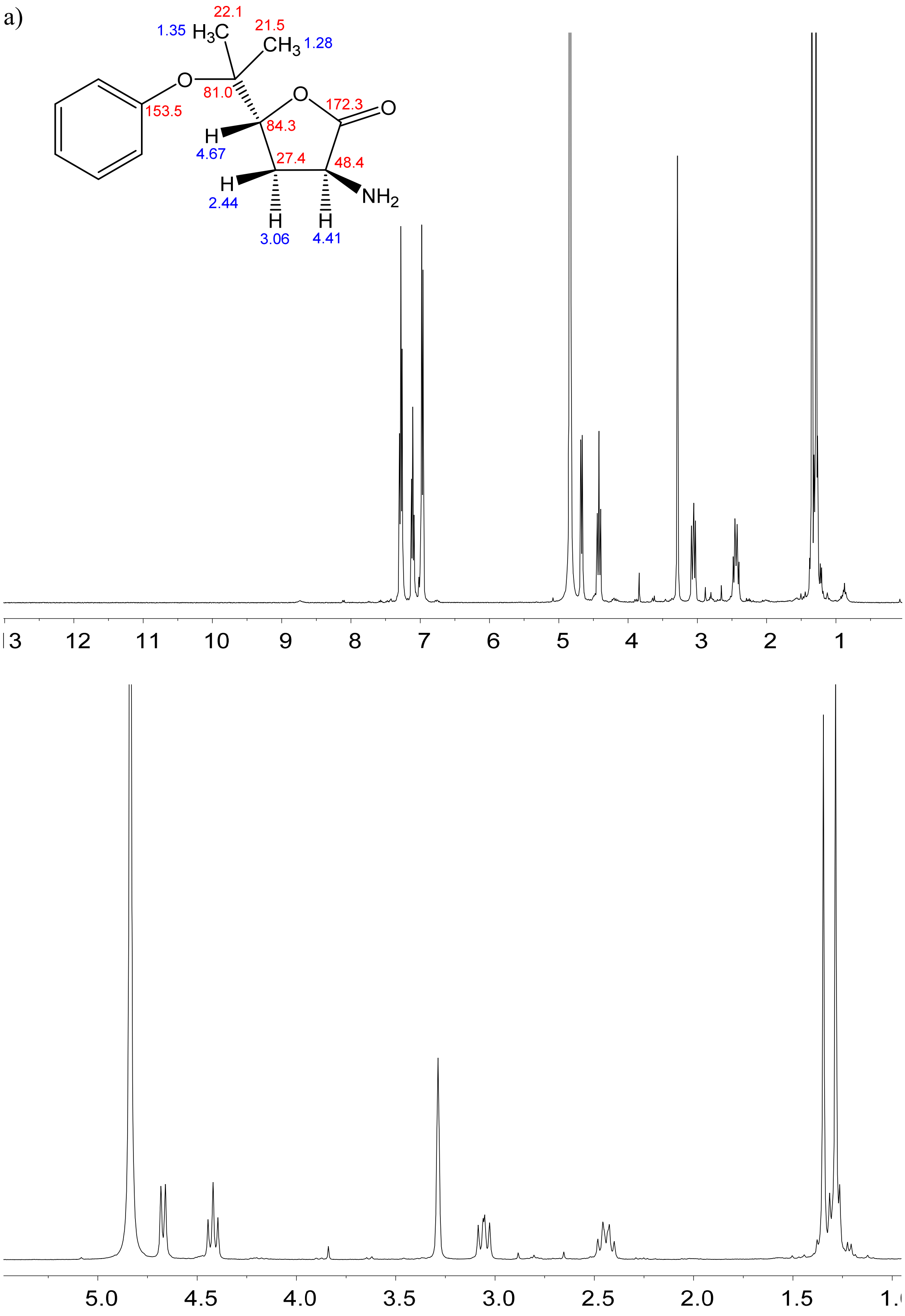
1.35 H_3C
22.1
21.5
CH_3 1.28
O
81.0
153.5
O
172.3
O
84.3
H
4.67
27.4
48.4
H
2.44
NH_2
H
3.06
H
4.41
13 12 11 10 9 8 7 6 5 4 3 2 1
5.0 4.5 4.0 3.5 3.0 2.5 2.0 1.5

b)

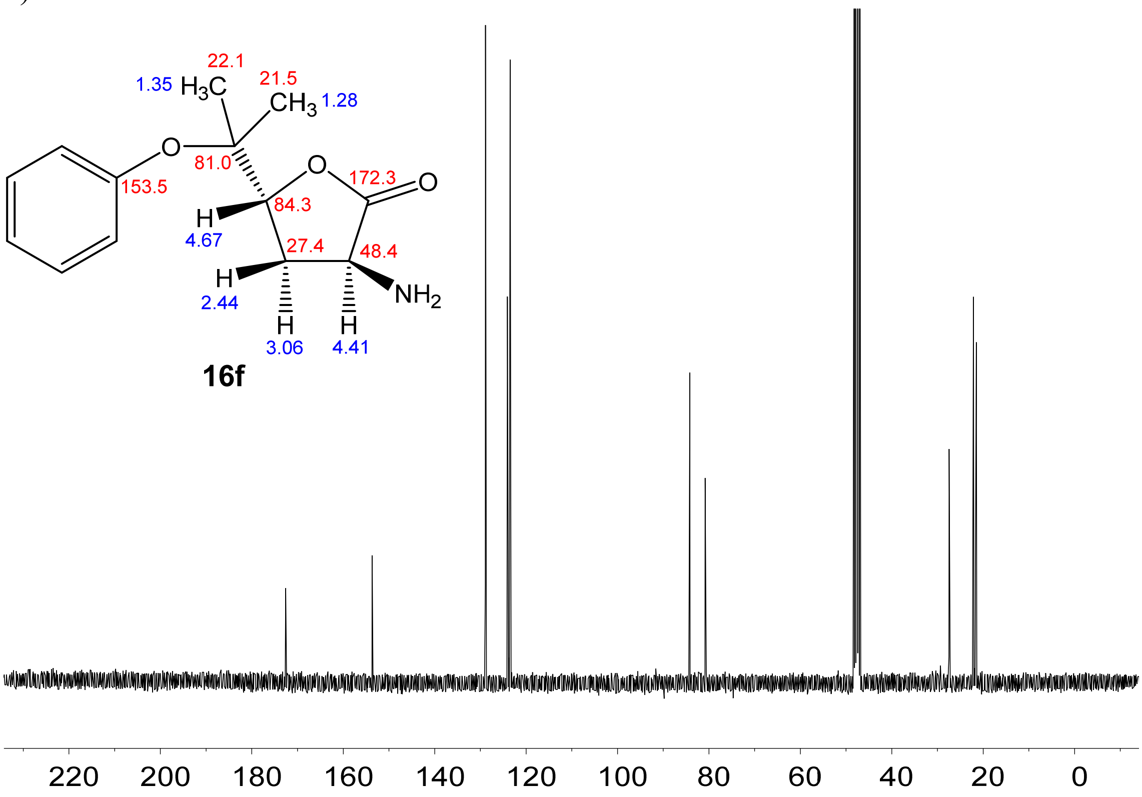

22.1
1.35 H3C
21.5
CH3 1.28
O
81.0
O
172.3
O
153.5
H
84.3
4.67
27.4
48.4
H
NH2
2.44
H
H
3.06
4.41
16f
220
200
180
160
140
120
100
80
60
40
20
0

c)

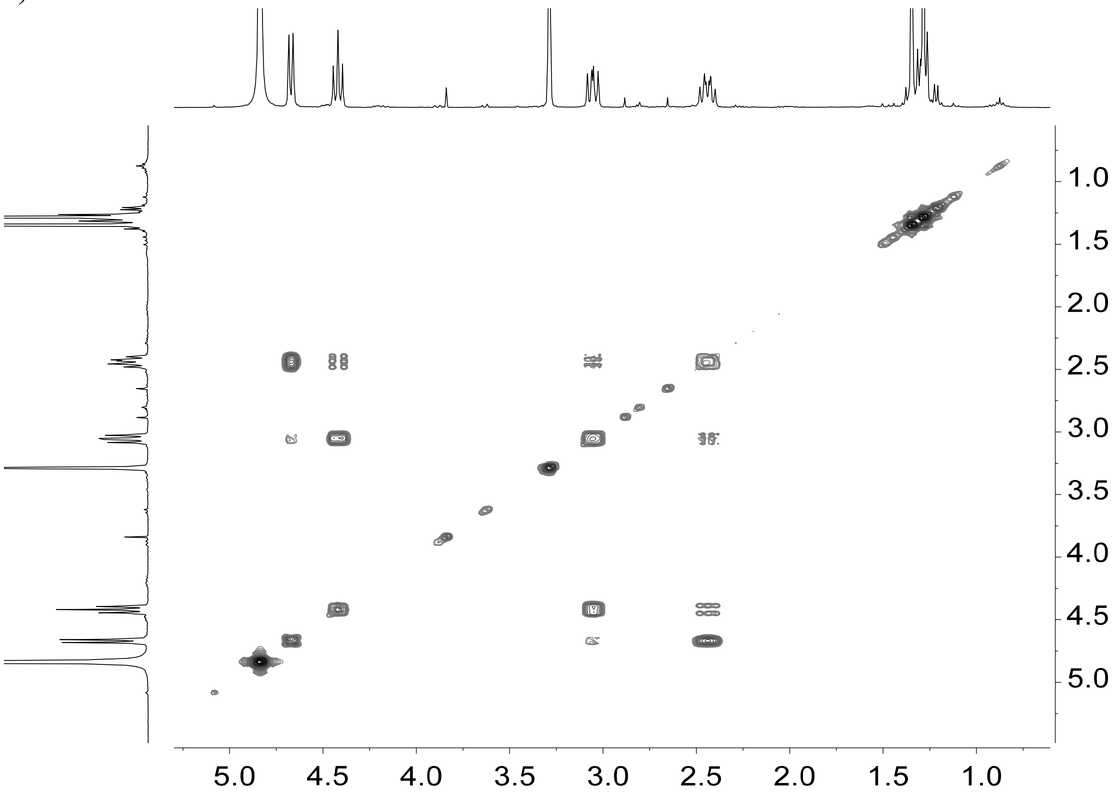

1.0
1.5
2.0
2.5
3.0
3.5
4.0
4.5
5.0
5.0
4.5
4.0
3.5
3.0
2.5
2.0
1.5
1.0

d)

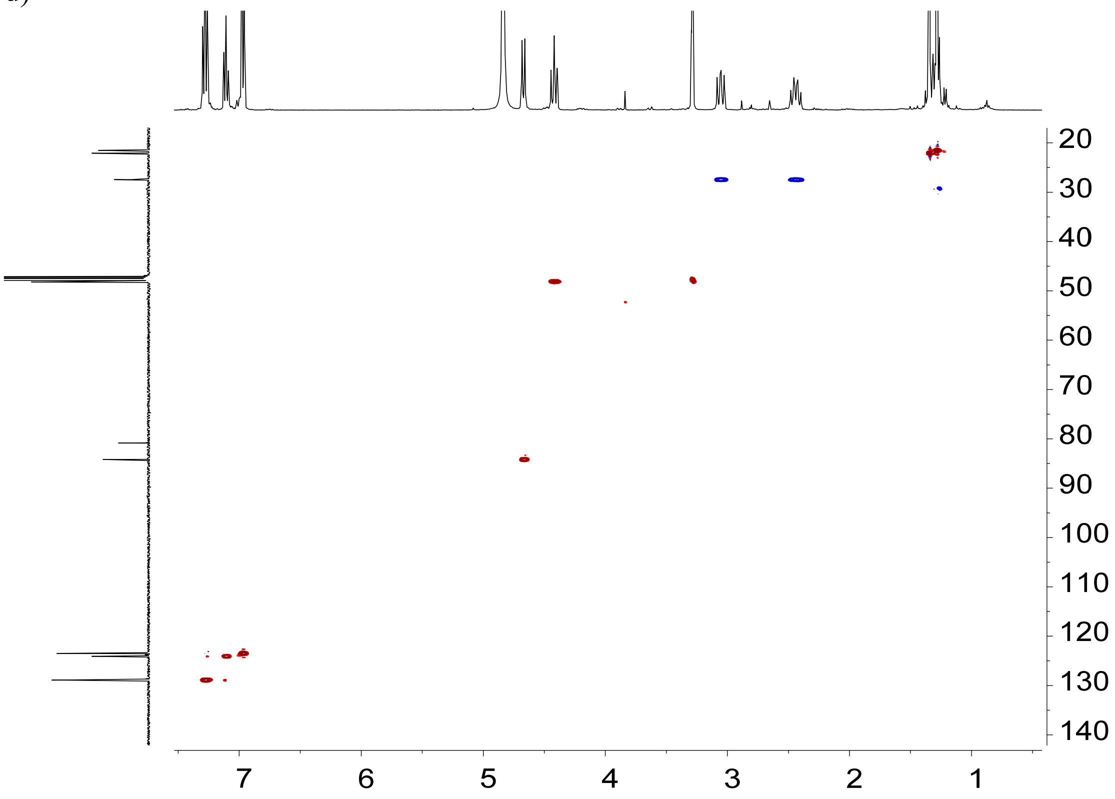

20
30
40
50
60
70
80
90
100
110
120
130
140
7
6
5
4
3
2
1

e)

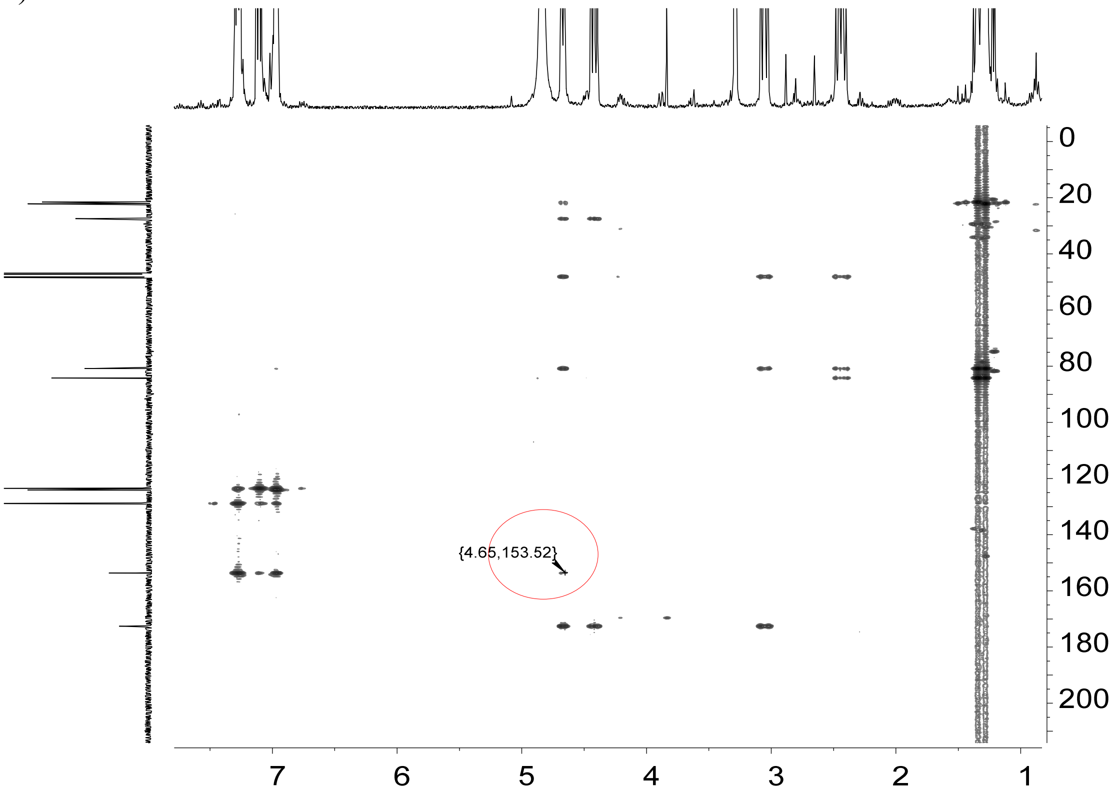

0
20
40
60
80
100
120
140
160
180
200
{4.65,153.52}
7
6
5
4
3
2
1

f)

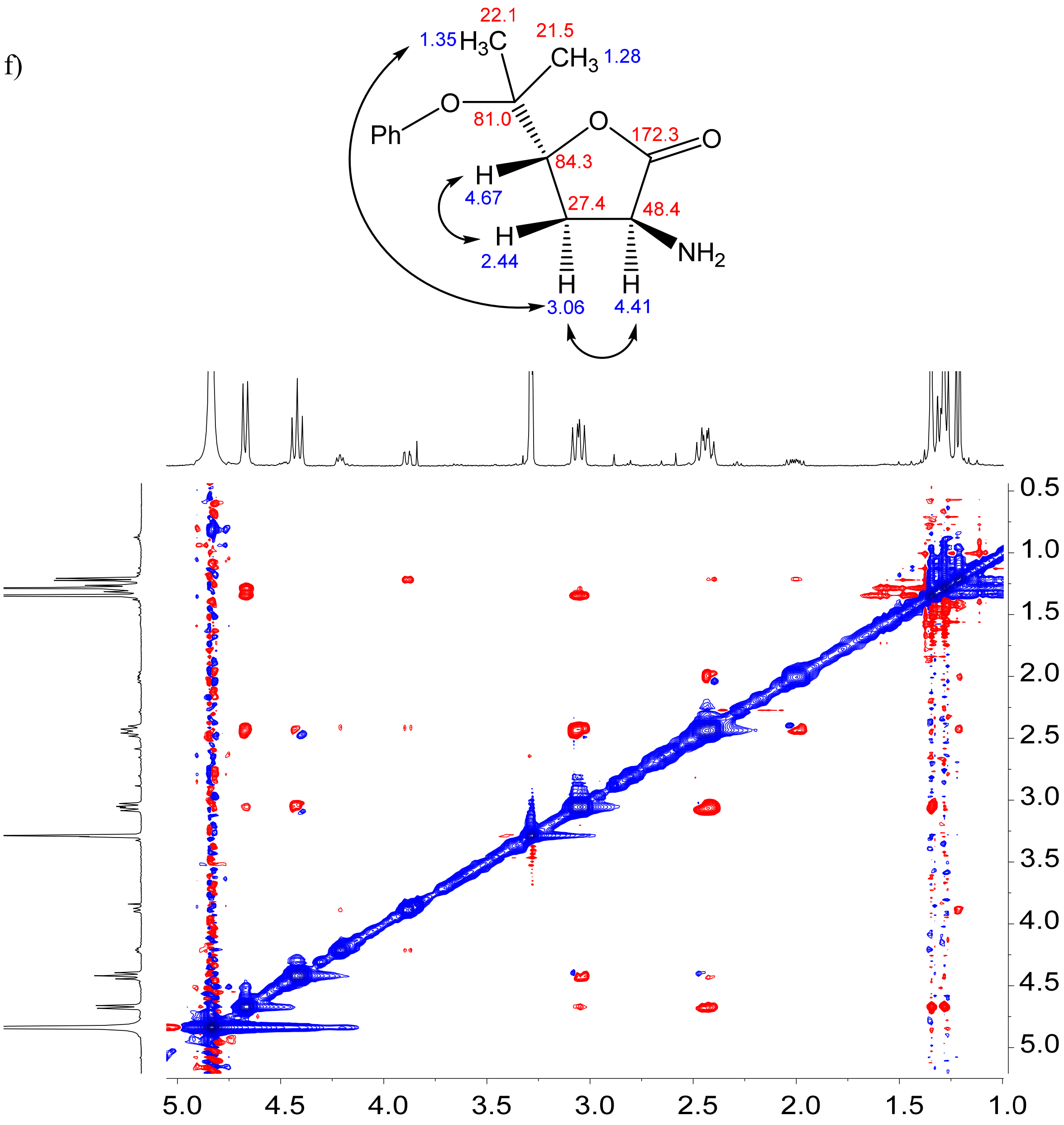

Figure S61. NMR spectra (CD_3OD) (i.e. Cbz removal) **16f** (i.e. unprotected **14f)**: a) ^1H, b) ^{13}C, c) COSY, d) HSQC, e) HMBC and f) NOESY.

a)

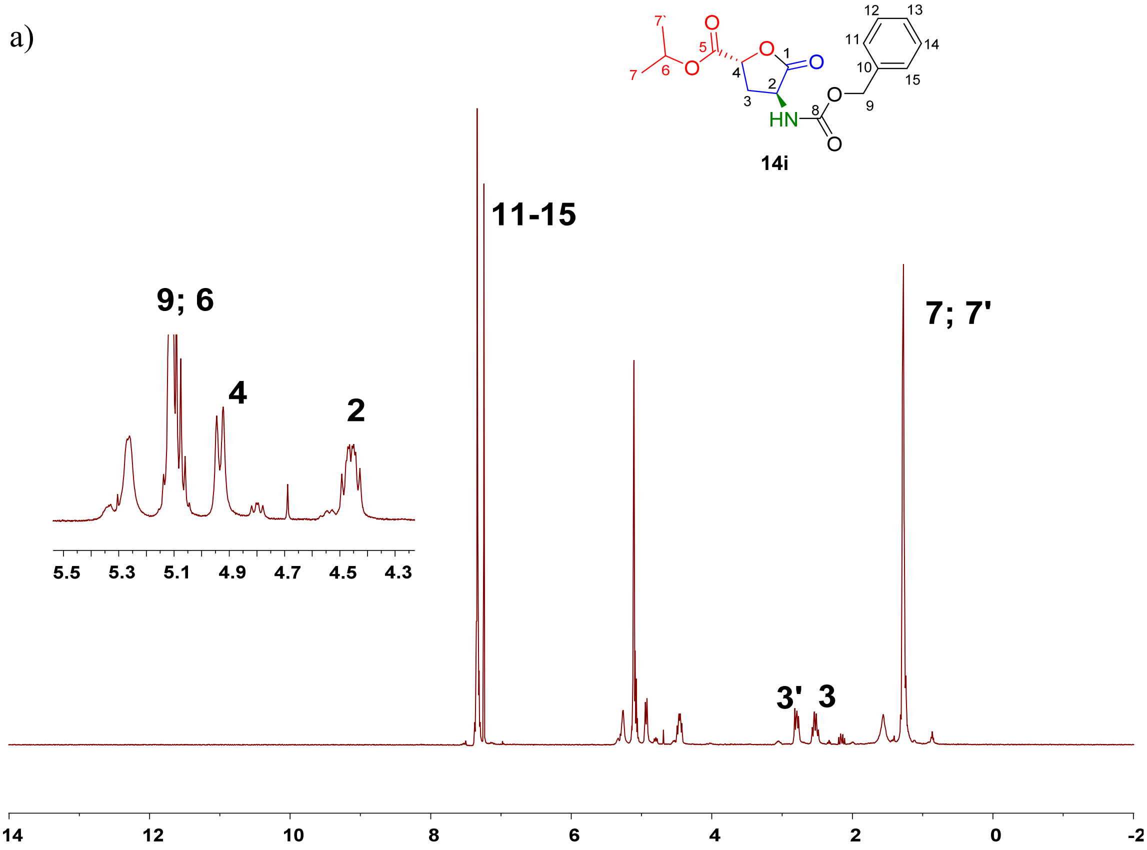
14i
11-15
9; 6
4
2
7; 7'
3' 3
5.5 5.3 5.1 4.9 4.7 4.5 4.3
14 12 10 8 6 4 2 0 -2

b)

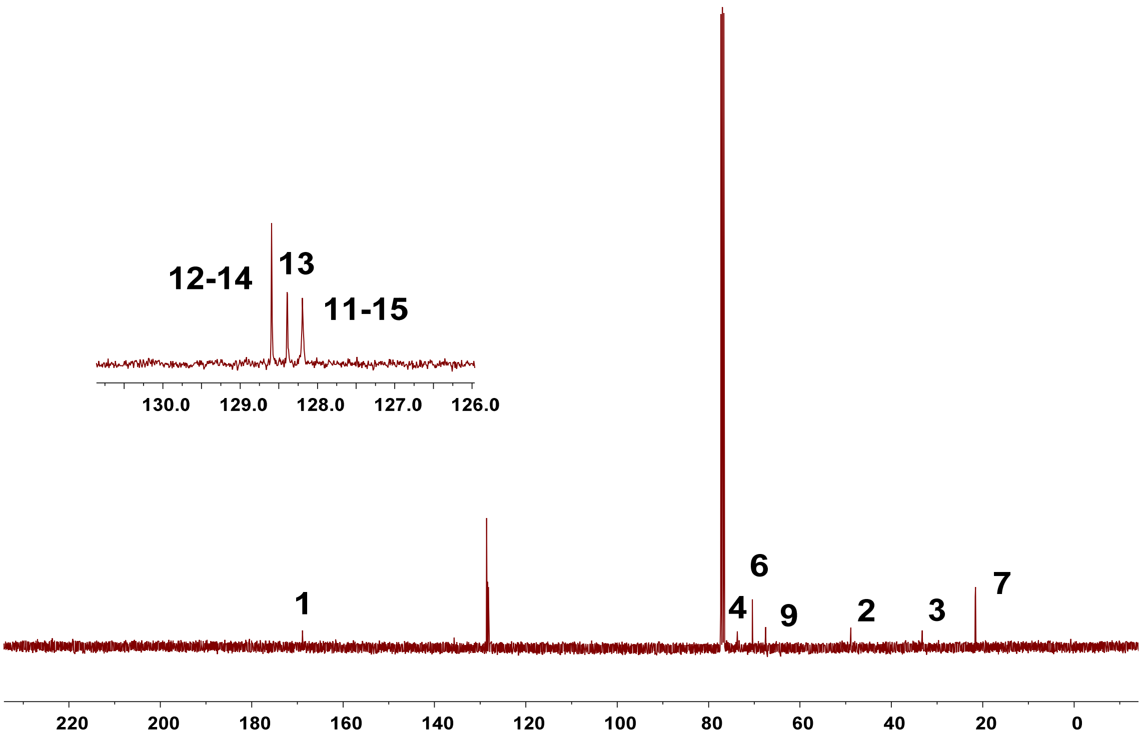
12-14
13
11-15
130.0 129.0 128.0 127.0 126.0
1
6
4
9
2
3
7
220 200 180 160 140 120 100 80 60 40 20 0

c)

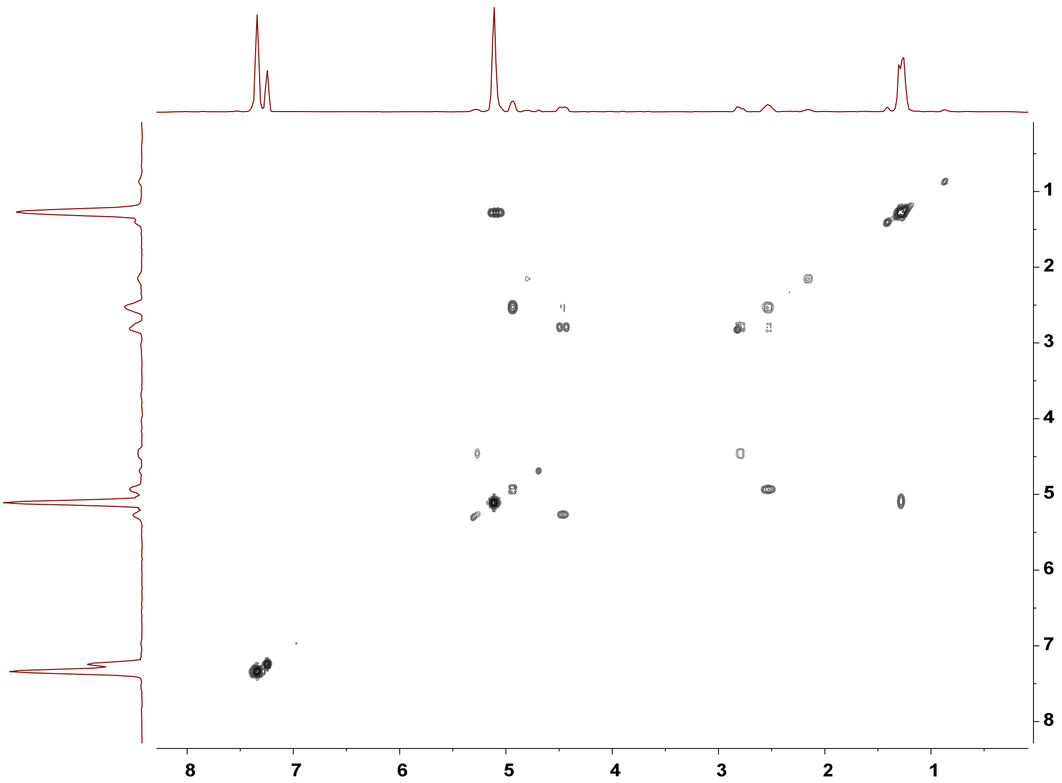

1
2
3
4
5
6
7
8
8
7
6
5
4
3
2
1

d)

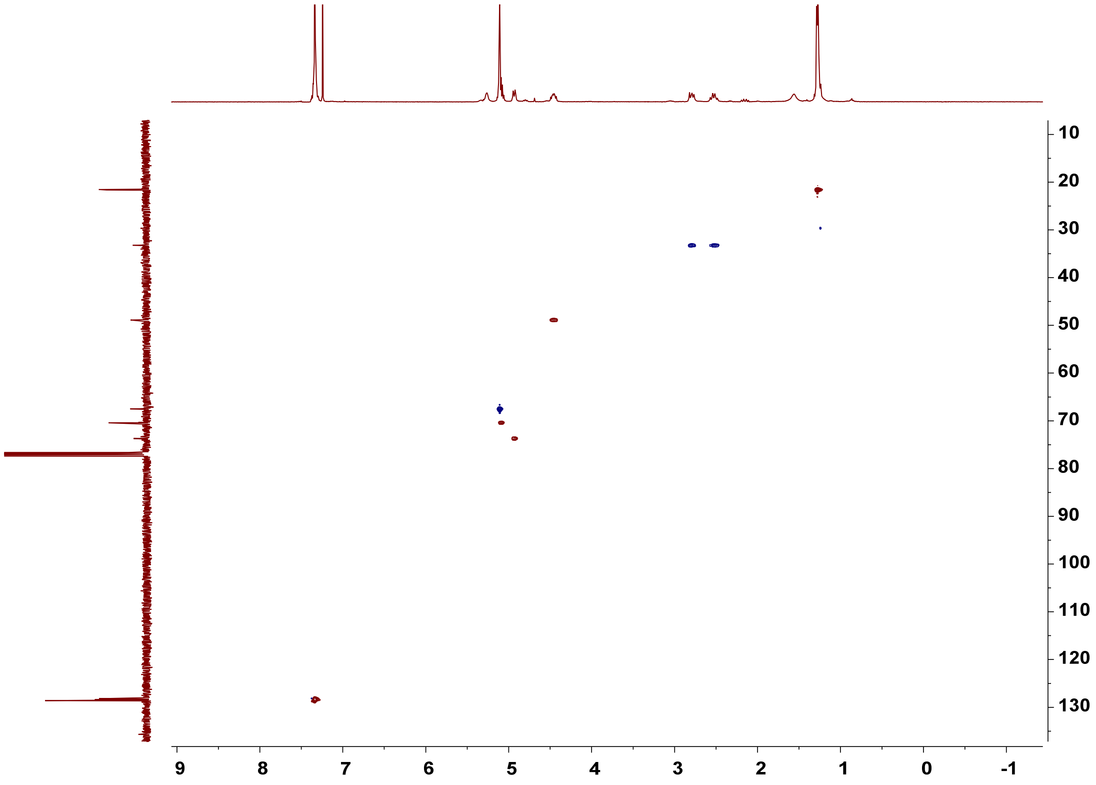

10
20
30
40
50
60
70
80
90
100
110
120
130
9
8
7
6
5
4
3
2
1
0
-1

e)

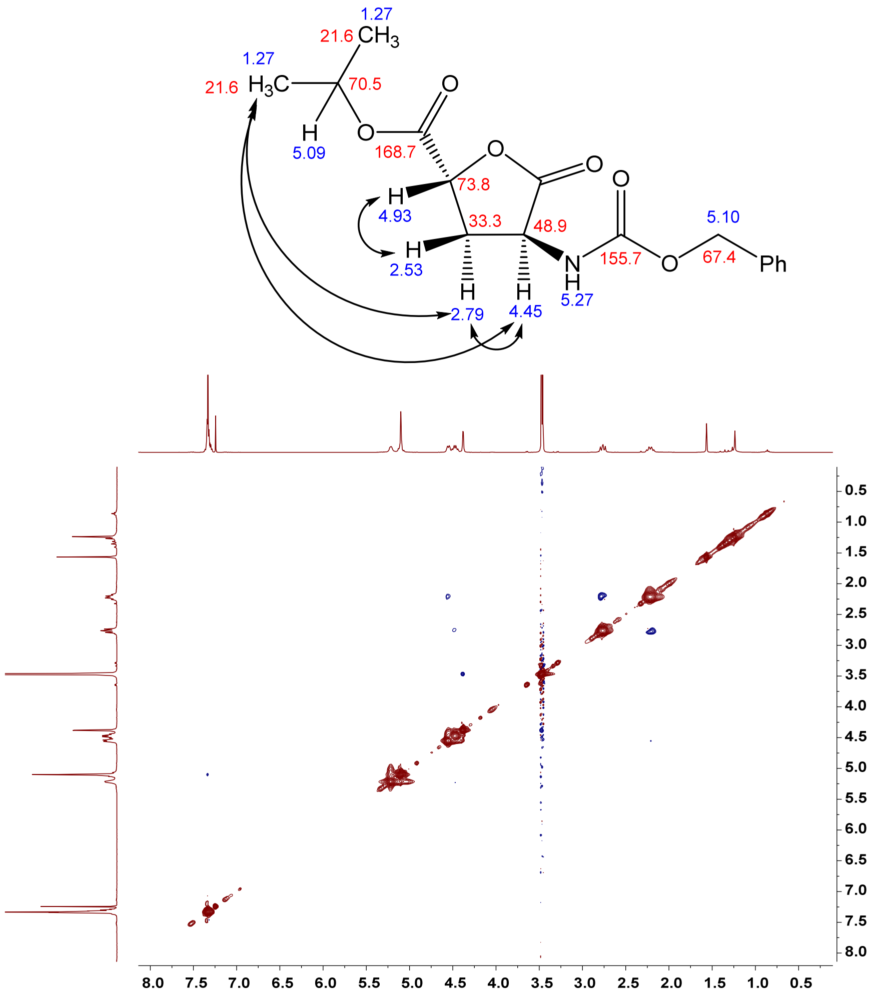

f)

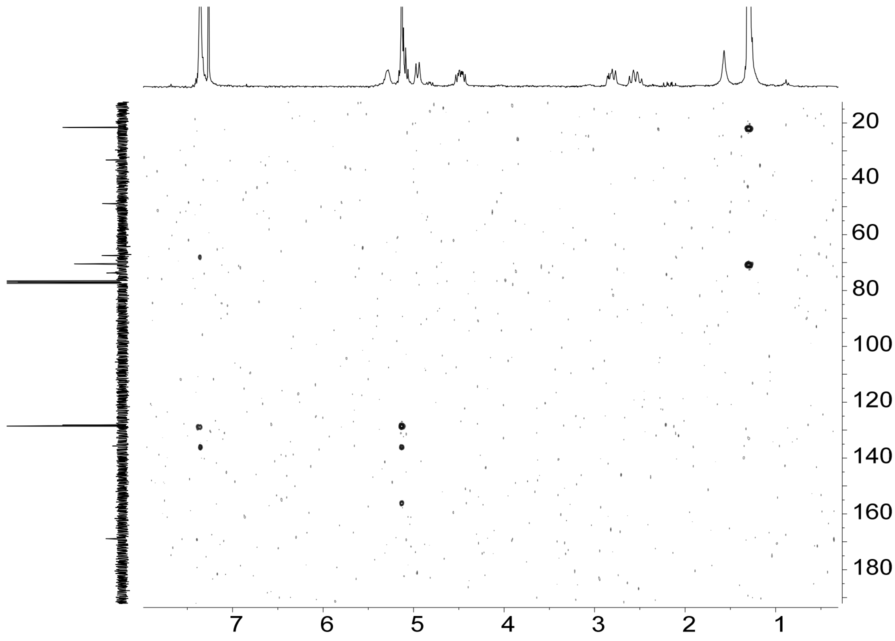

Figure S62. NMR spectra ($CDCl_3$) of **14i**: a) ^{1}H, b) ^{13}C, c) COSY, d) HSQC, e) NOESY and f) HMBC.

a)

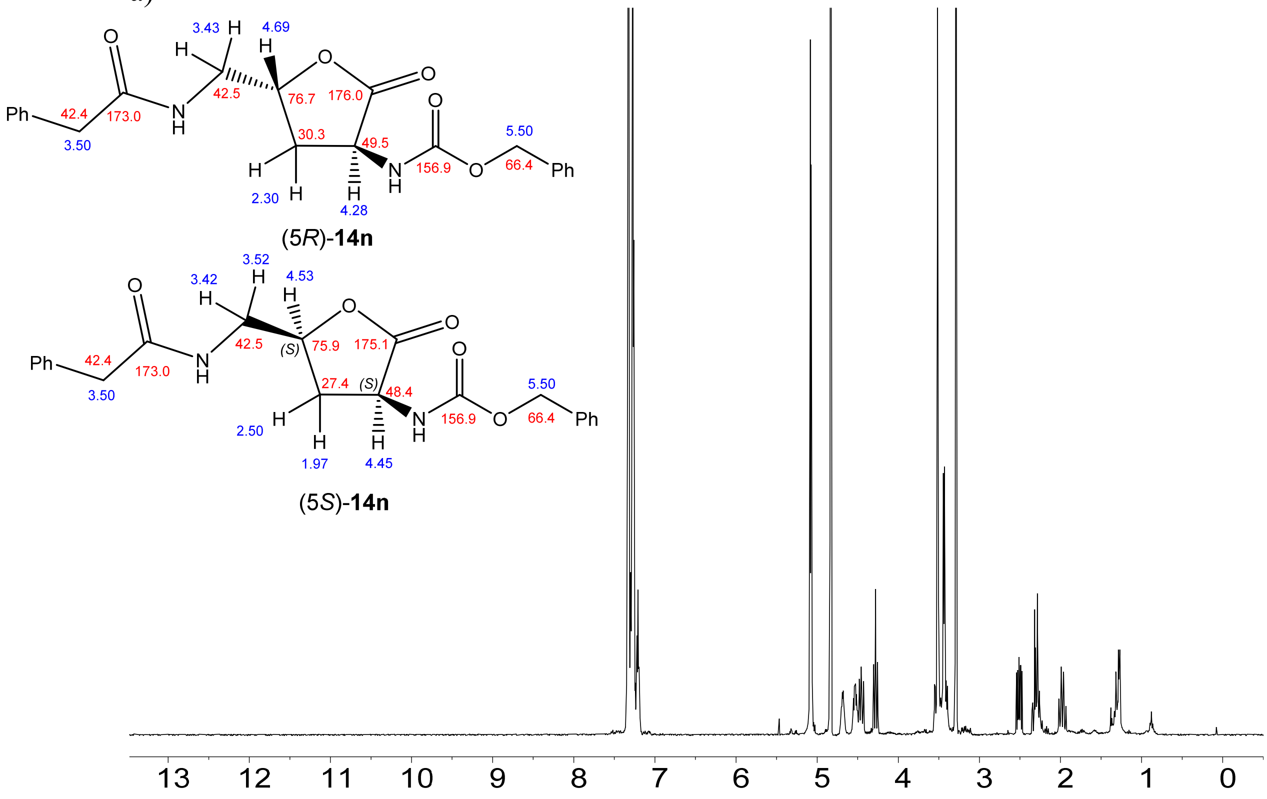
3.43
4.69
42.5
76.7
176.0
42.4
173.0
30.3
49.5
156.9
66.4
5.50
3.50
2.30
4.28
Ph
Ph
(5R)-14n
3.52
3.42
4.53
42.5
(S)
75.9
175.1
42.4
173.0
27.4
(S)
48.4
156.9
66.4
5.50
3.50
2.50
1.97
4.45
Ph
Ph
(5S)-14n
13 12 11 10 9 8 7 6 5 4 3 2 1 0

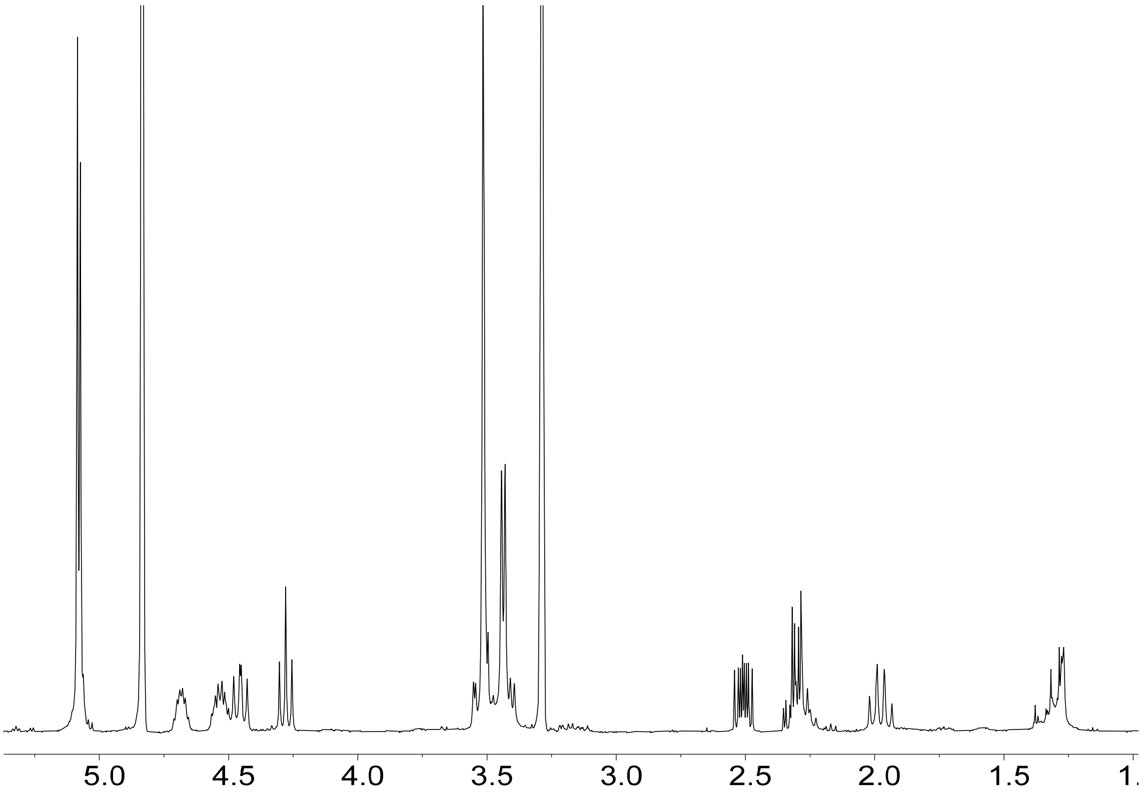
5.0 4.5 4.0 3.5 3.0 2.5 2.0 1.5

b)

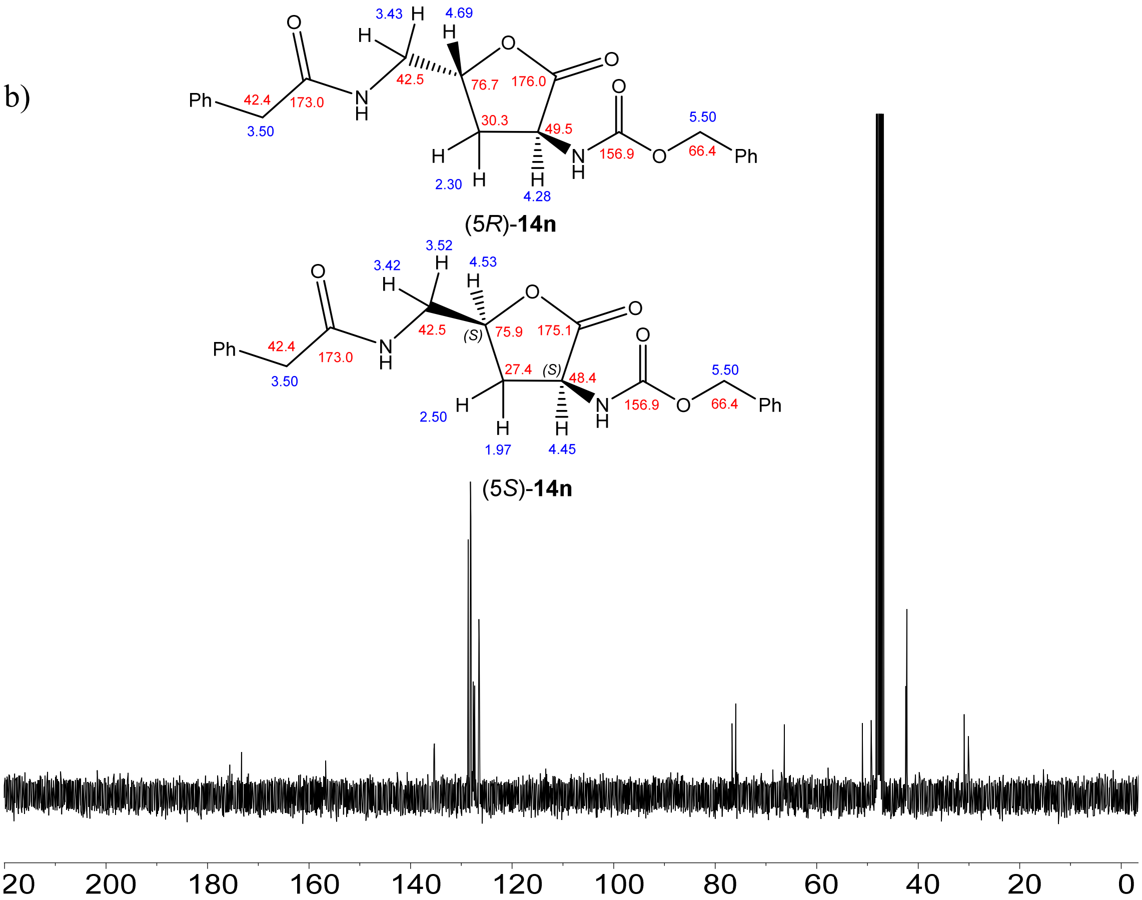
(5R)-14n
(5S)-14n
20 200 180 160 140 120 100 80 60 40 20 0

c)

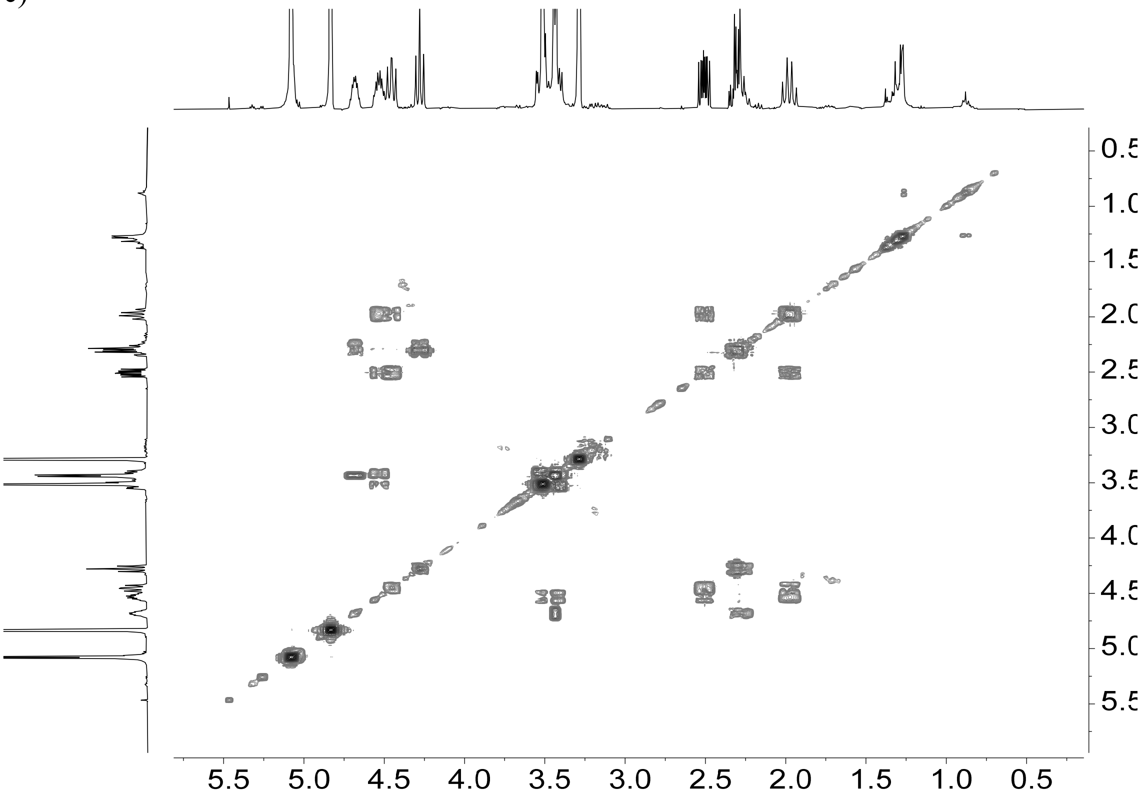
5.5 5.0 4.5 4.0 3.5 3.0 2.5 2.0 1.5 1.0 0.5

d)

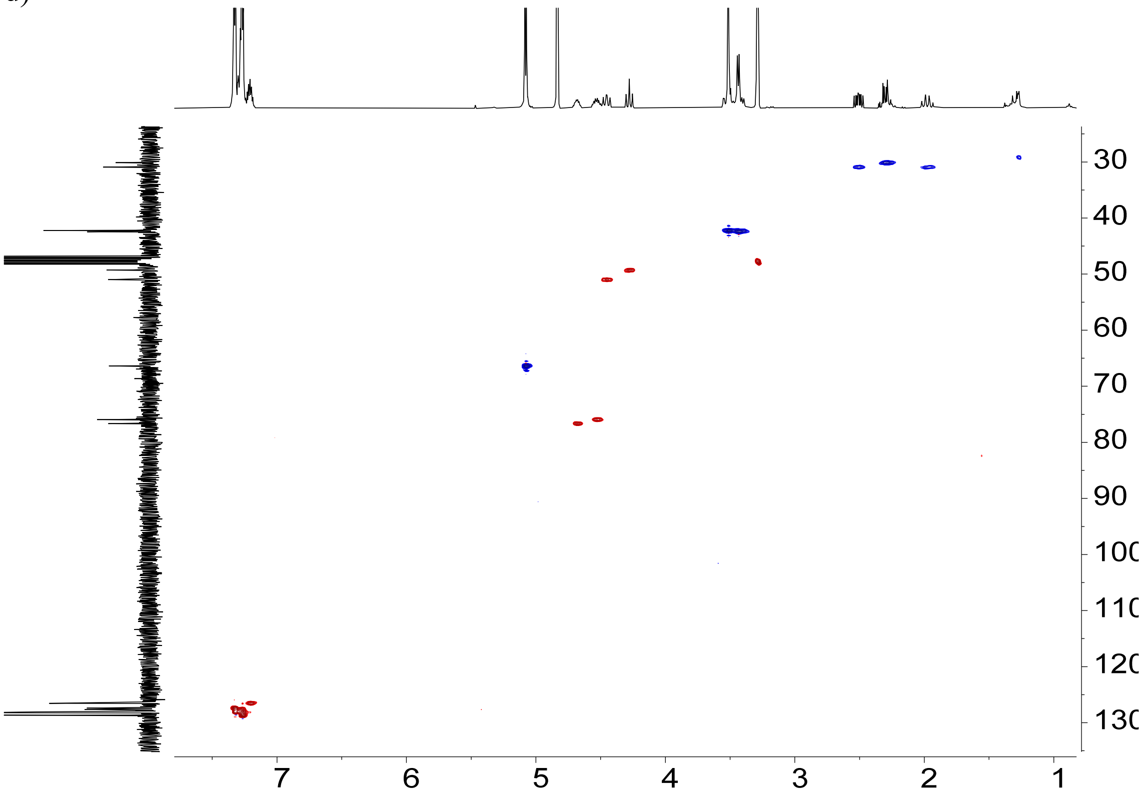
30
40
50
60
70
80
90
100
110
120
130
7
6
5
4
3
2
1

e)

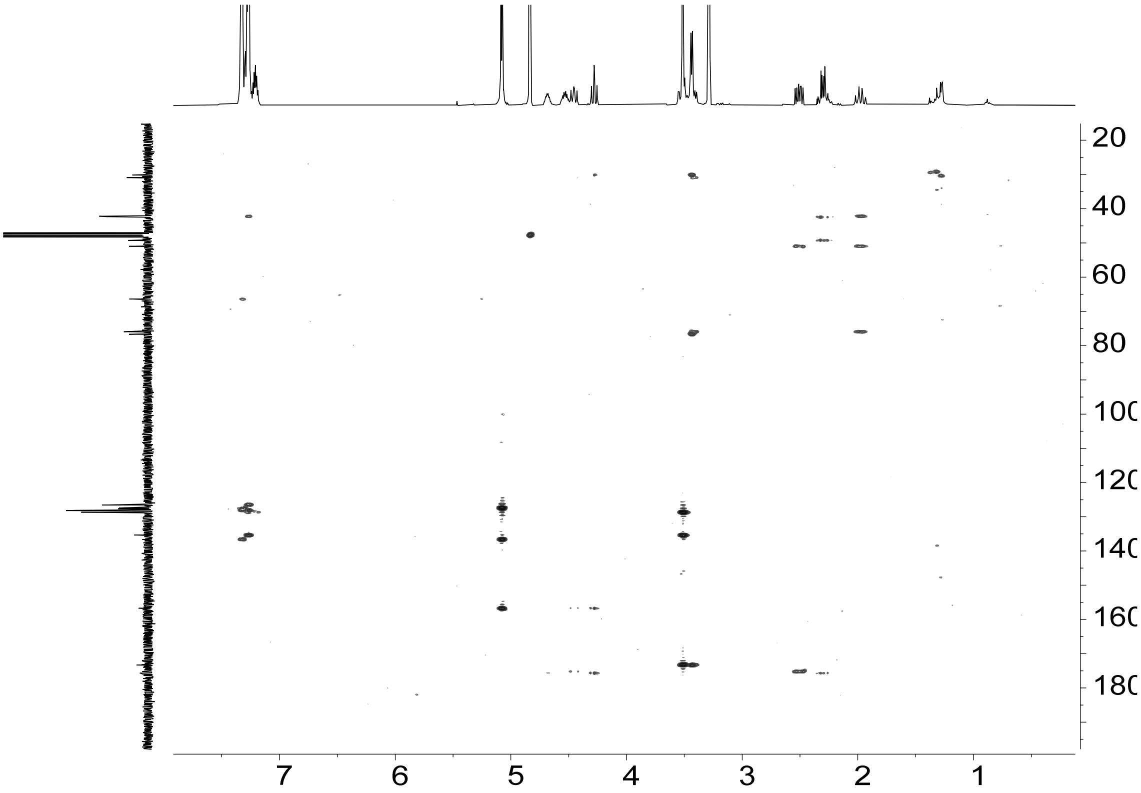
20
40
60
80
100
120
140
160
180
7
6
5
4
3
2
1

f)

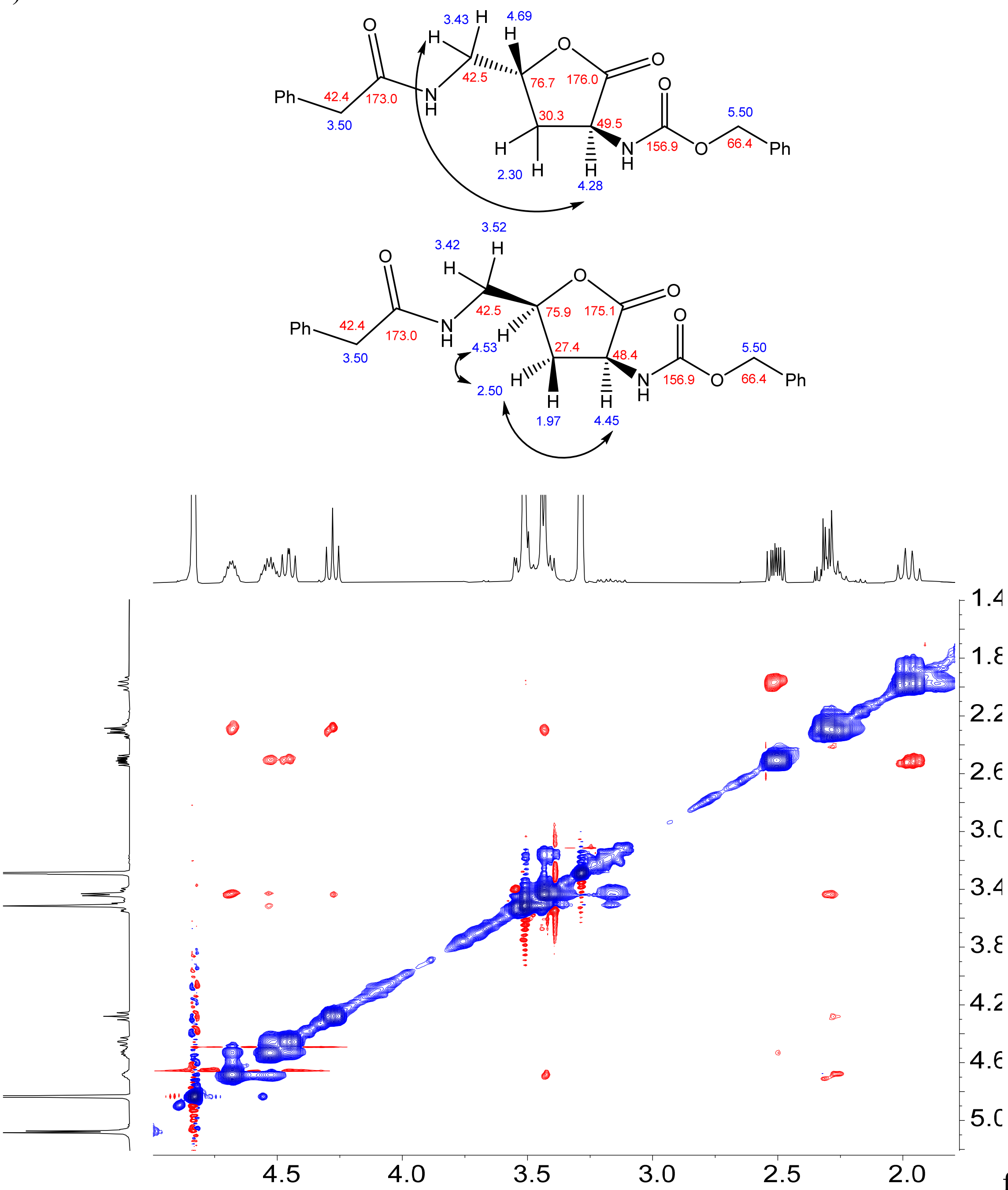

f)

Figure S63. NMR spectra (CD_3OD) of (5*R*/*S*)-**14n**: a) ^{1}H, b) ^{13}C, c) COSY, d) HSQC, e) HMBC and f) NOESY.

a)

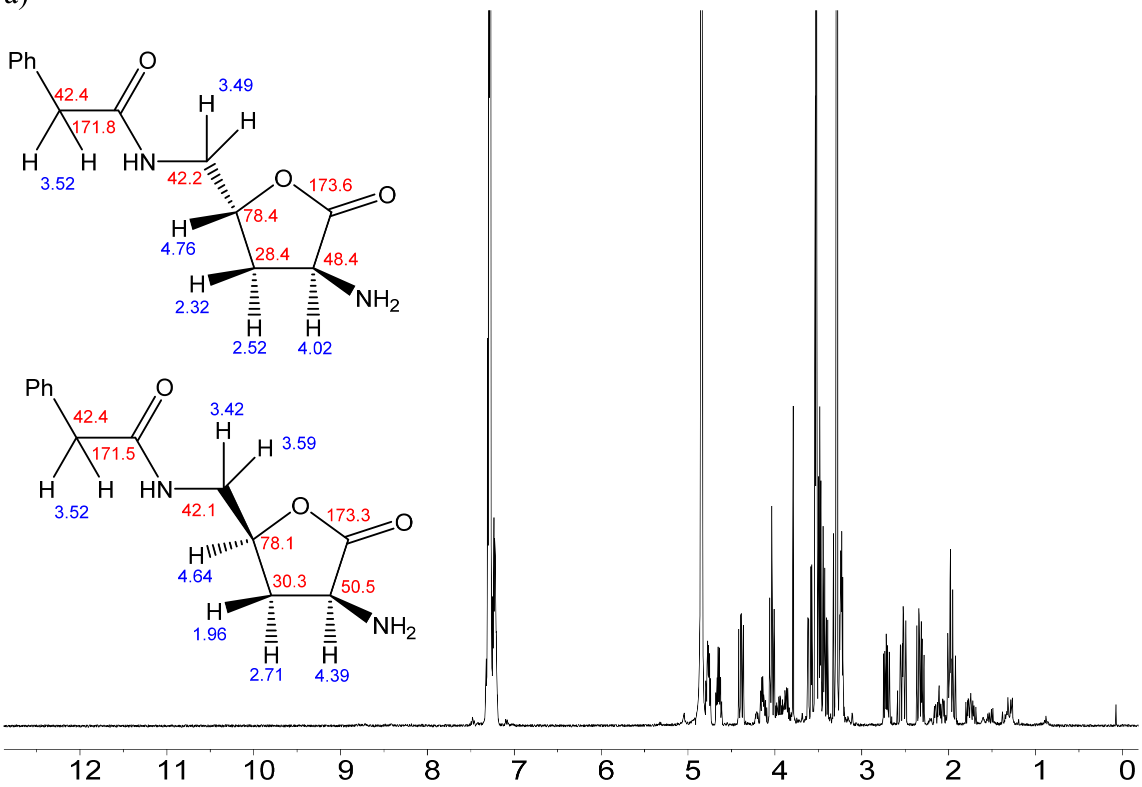

Ph
O
42.4
171.8
3.49
H
H
H
H
HN
3.52
42.2
O
173.6
O
H
78.4
4.76
28.4
48.4
H
NH$_2$
2.32
H
H
2.52
4.02
Ph
O
42.4
3.42
H
H
3.59
171.5
H
H
HN
3.52
42.1
O
173.3
O
H
78.1
4.64
30.3
50.5
H
NH$_2$
1.96
H
H
2.71
4.39
12
11
10
9
8
7
6
5
4
3
2
1
0

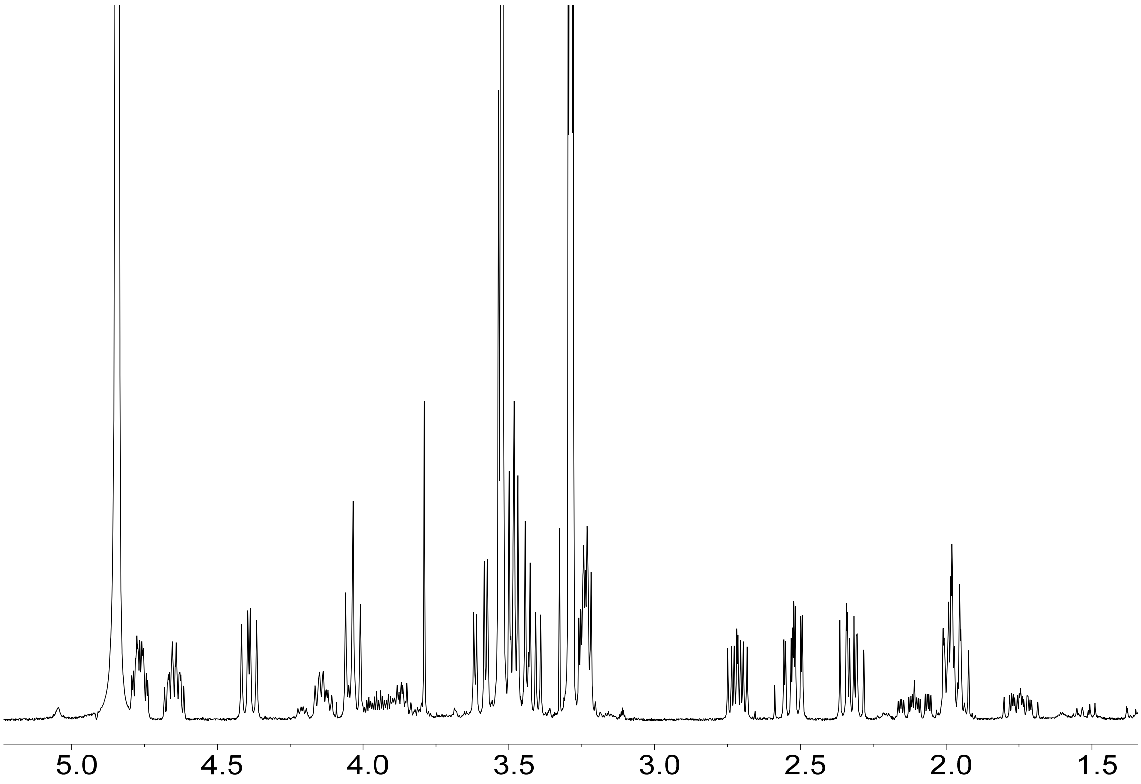

5.0
4.5
4.0
3.5
3.0
2.5
2.0
1.5

b)

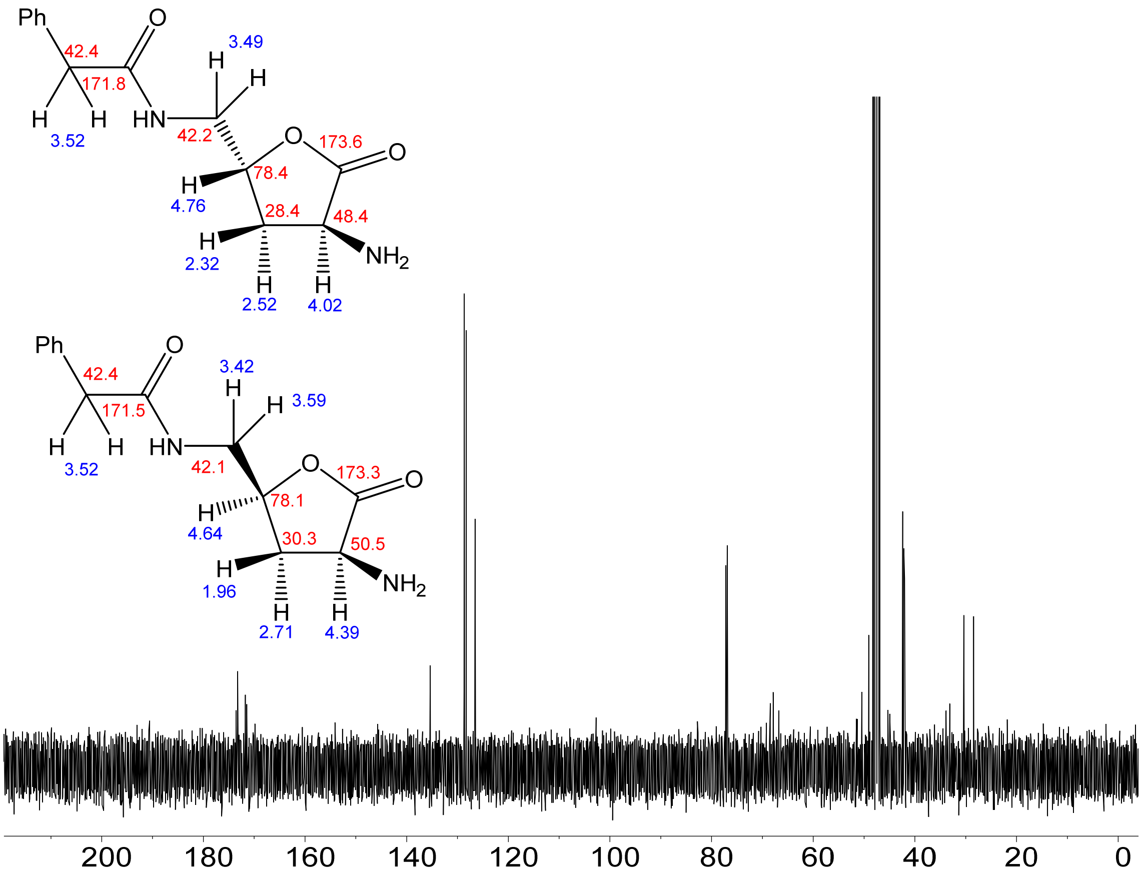
Ph
O
42.4
171.8
3.49
H
H
H
H
3.52
HN
42.2
O
173.6
O
78.4
H
4.76
28.4
48.4
H
2.32
NH2
H
2.52
H
4.02
Ph
O
42.4
171.5
3.42
H
H 3.59
H
H
3.52
HN
42.1
O
173.3
O
78.1
H
4.64
30.3
50.5
H
1.96
NH2
H
2.71
H
4.39
200
180
160
140
120
100
80
60
40
20
0

c)

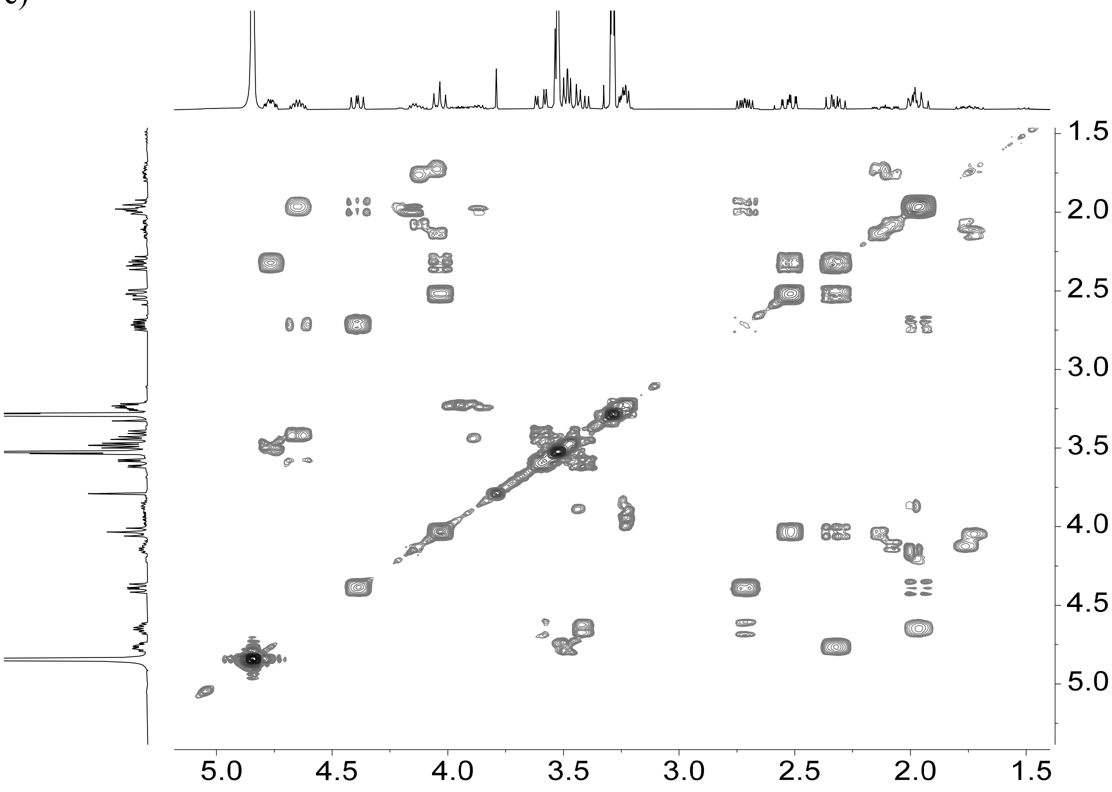
1.5
2.0
2.5
3.0
3.5
4.0
4.5
5.0
5.0
4.5
4.0
3.5
3.0
2.5
2.0
1.5

d)

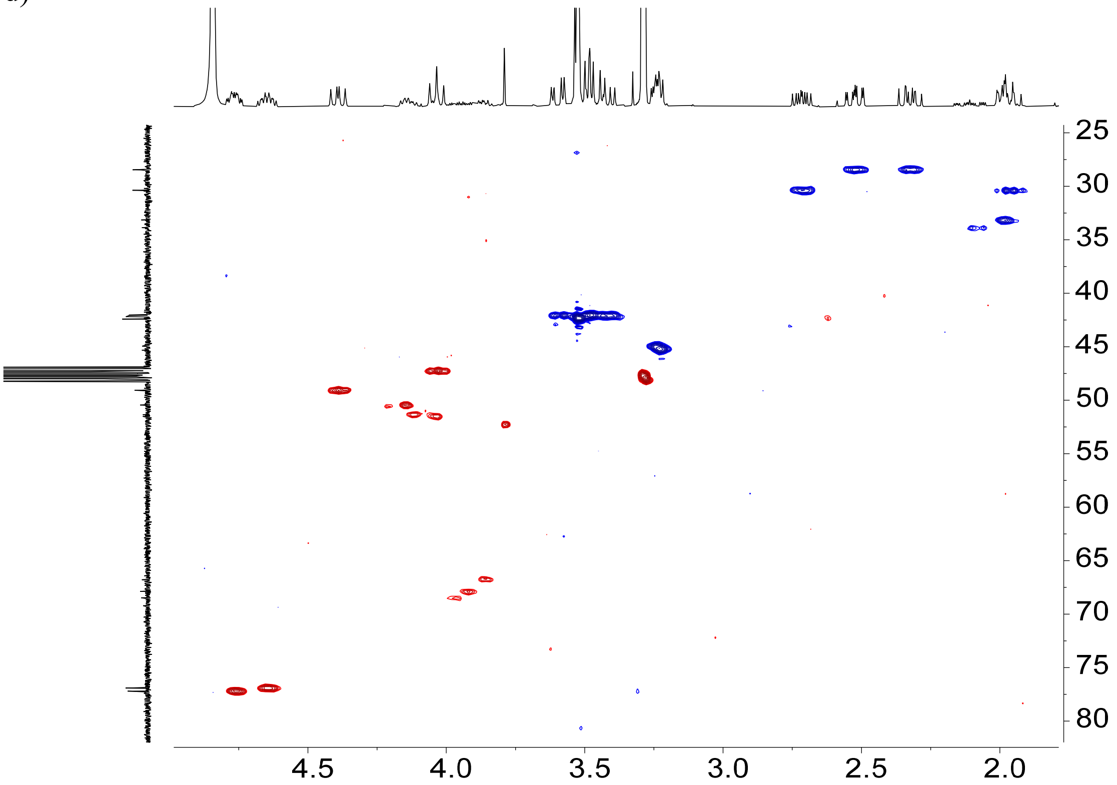

Figure S64. NMR spectra (CD_3OD) of (5*R*/*S*)-**17n** (i.e. Cbz-removal of (5*R*/*S*)-**14n** mixture**)**: a) ^1H, b) ^{13}C, c) COSY, d) HSQC.

a)

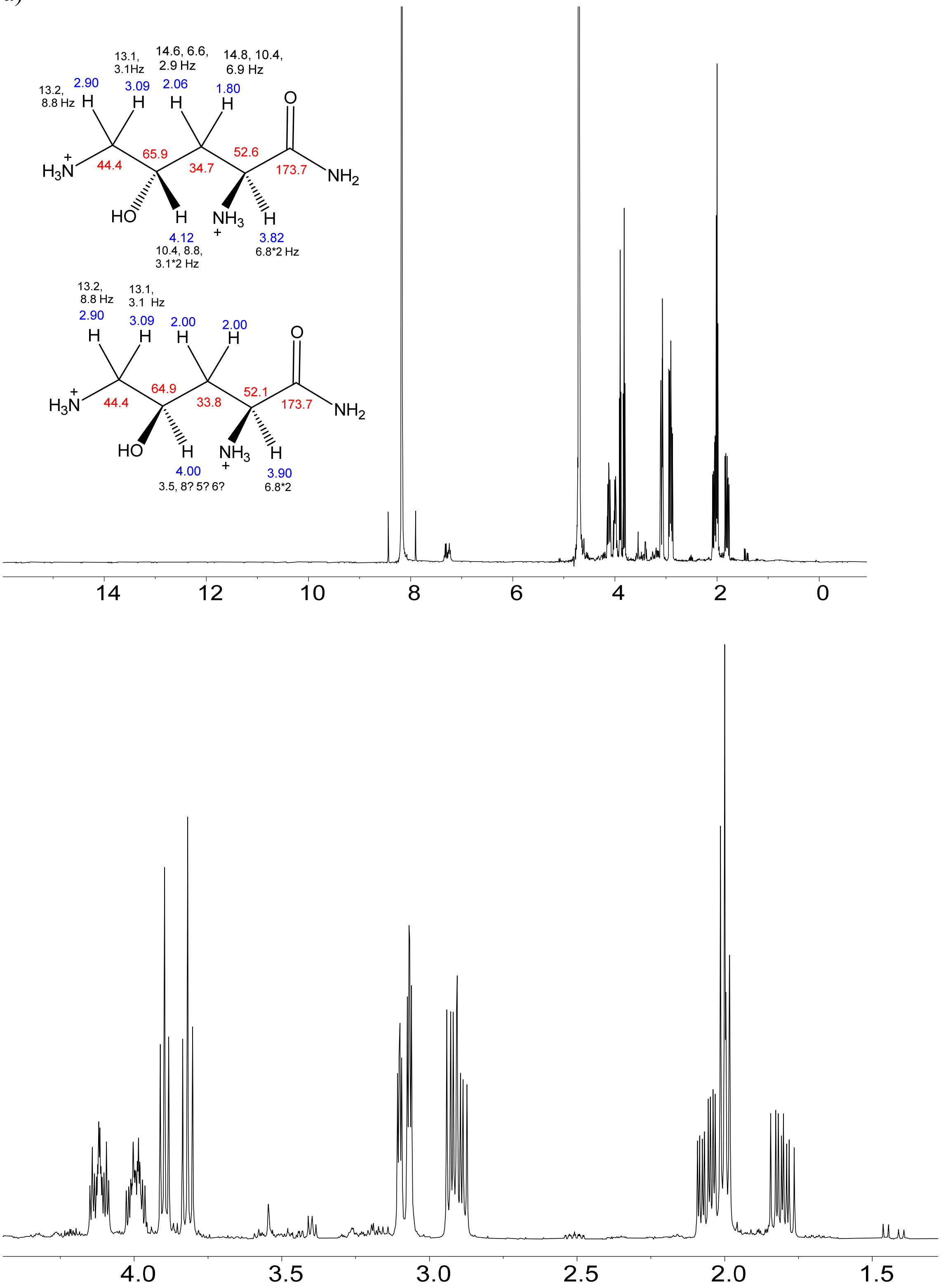

13.2, 8.8 Hz
2.90
13.1, 3.1Hz
3.09
14.6, 6.6, 2.9 Hz
2.06
14.8, 10.4, 6.9 Hz
1.80
65.9
44.4
34.7
52.6
173.7
4.12
10.4, 8.8, 3.1*2 Hz
3.82
6.8*2 Hz
13.2, 8.8 Hz
2.90
13.1, 3.1 Hz
3.09
2.00
2.00
64.9
44.4
33.8
52.1
173.7
4.00
3.5, 8? 5? 6?
3.90
6.8*2
14
12
10
8
6
4
2
0
4.0
3.5
3.0
2.5
2.0
1.5

b)

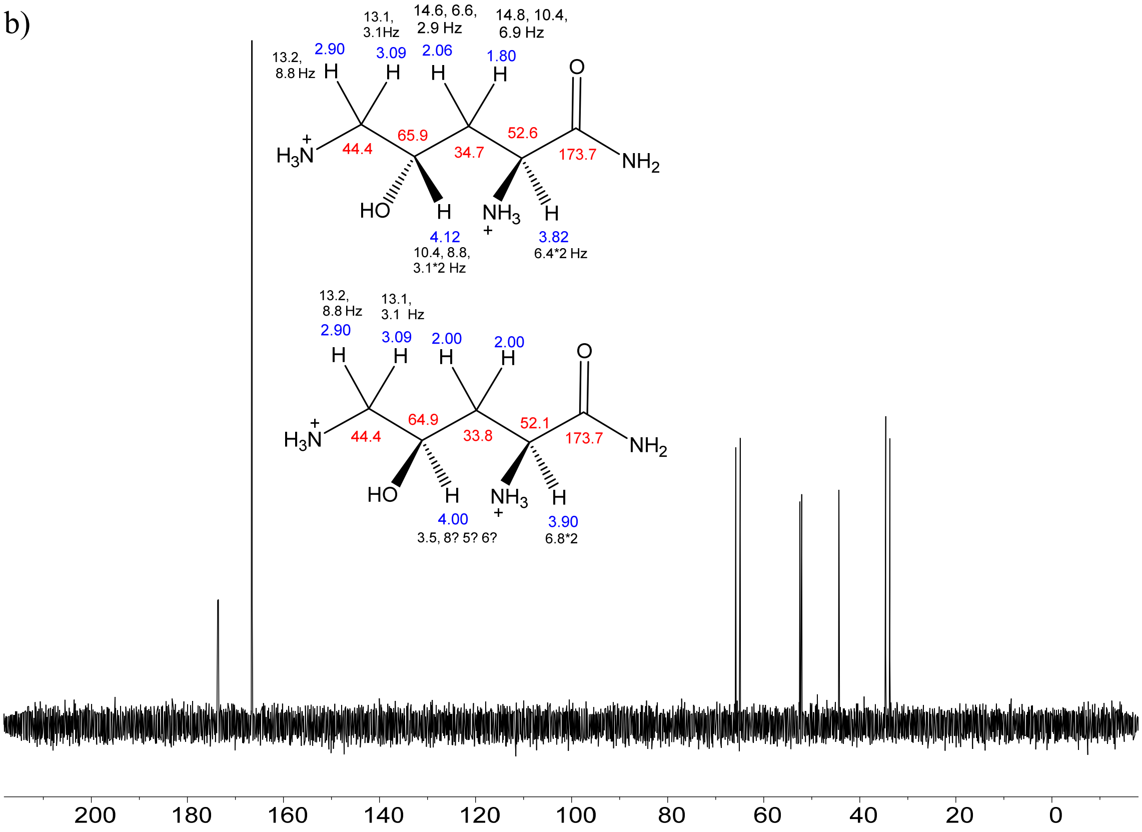
13.2, 8.8 Hz
2.90
13.1, 3.1Hz
3.09
14.6, 6.6, 2.9 Hz
2.06
14.8, 10.4, 6.9 Hz
1.80
44.4
65.9
34.7
52.6
173.7
4.12
10.4, 8.8, 3.1*2 Hz
3.82
6.4*2 Hz
13.2, 8.8 Hz
2.90
13.1, 3.1 Hz
3.09
2.00
2.00
44.4
64.9
33.8
52.1
173.7
4.00
3.5, 8? 5? 6?
3.90
6.8*2
200
180
160
140
120
100
80
60
40
20
0

c)

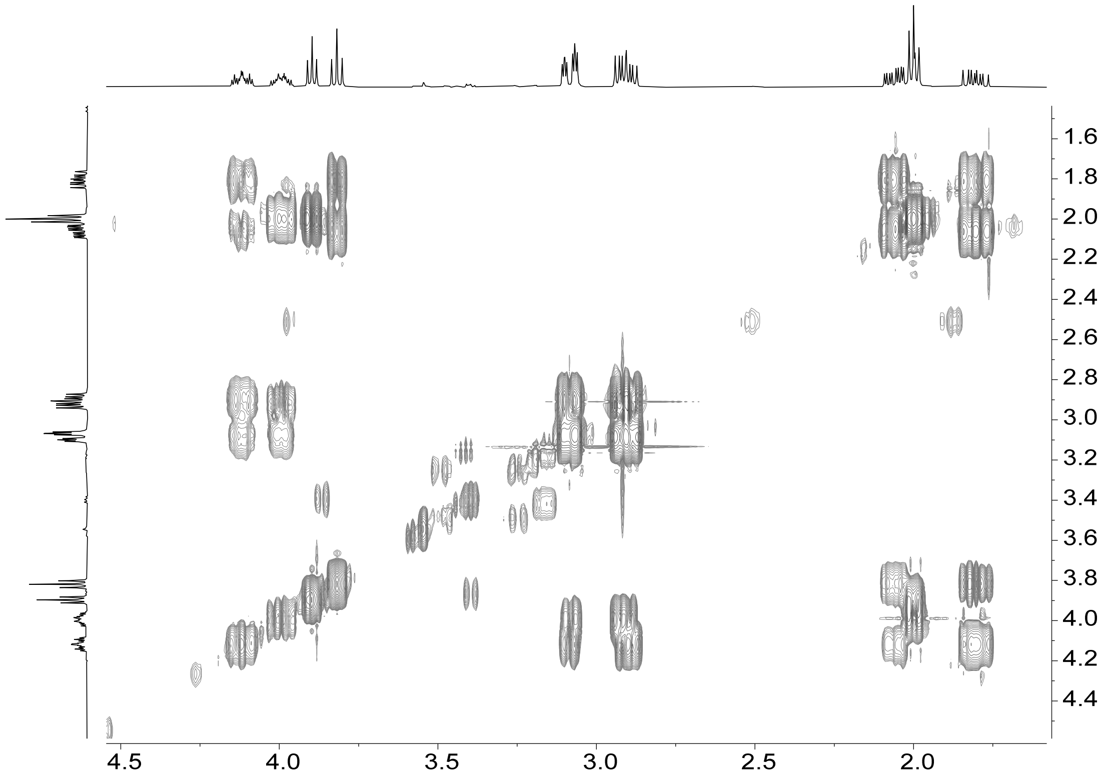
4.5
4.0
3.5
3.0
2.5
2.0
1.6
1.8
2.0
2.2
2.4
2.6
2.8
3.0
3.2
3.4
3.6
3.8
4.0
4.2
4.4

d)

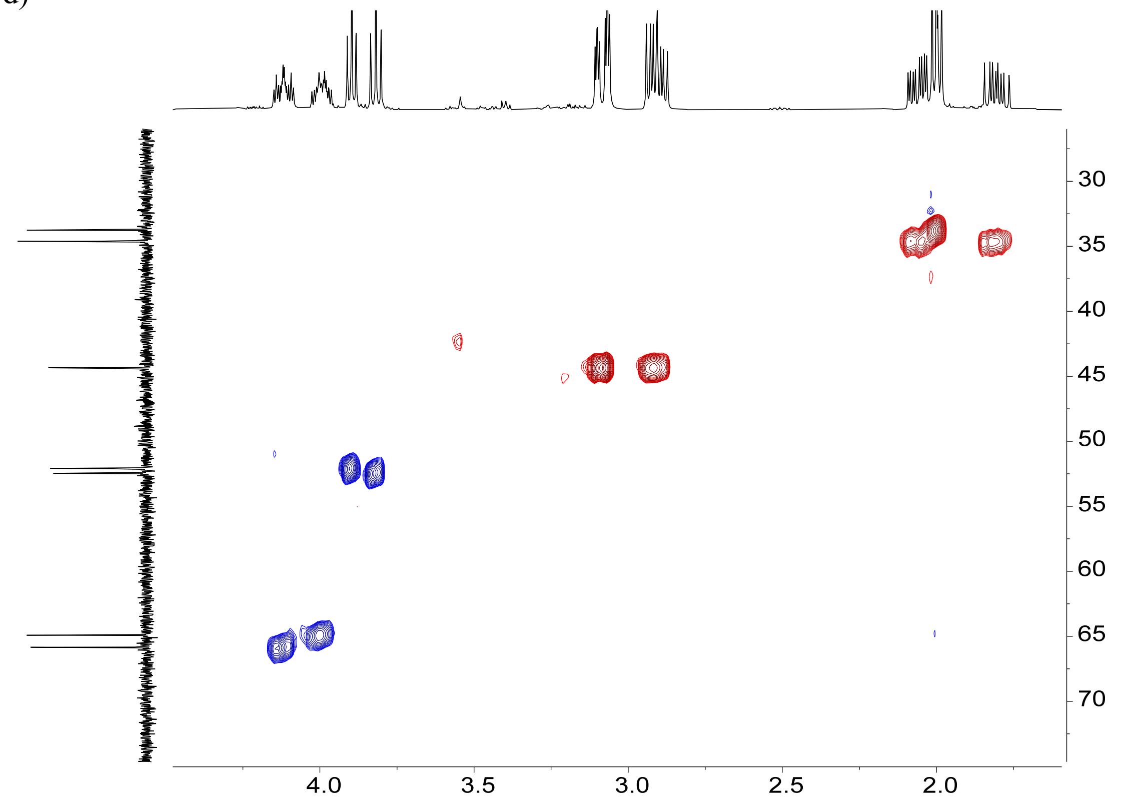
30
35
40
45
50
55
60
65
70
4.0
3.5
3.0
2.5
2.0

e)

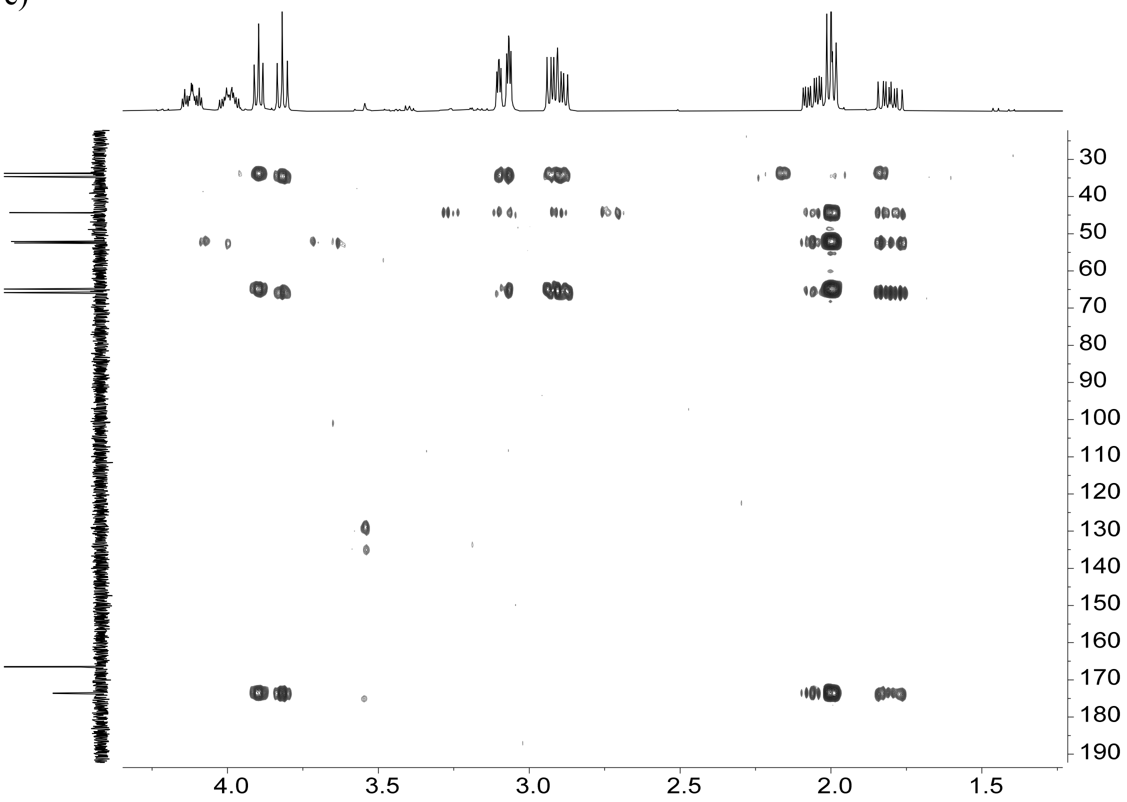
30
40
50
60
70
80
90
100
110
120
130
140
150
160
170
180
190
4.0
3.5
3.0
2.5
2.0
1.5

f)

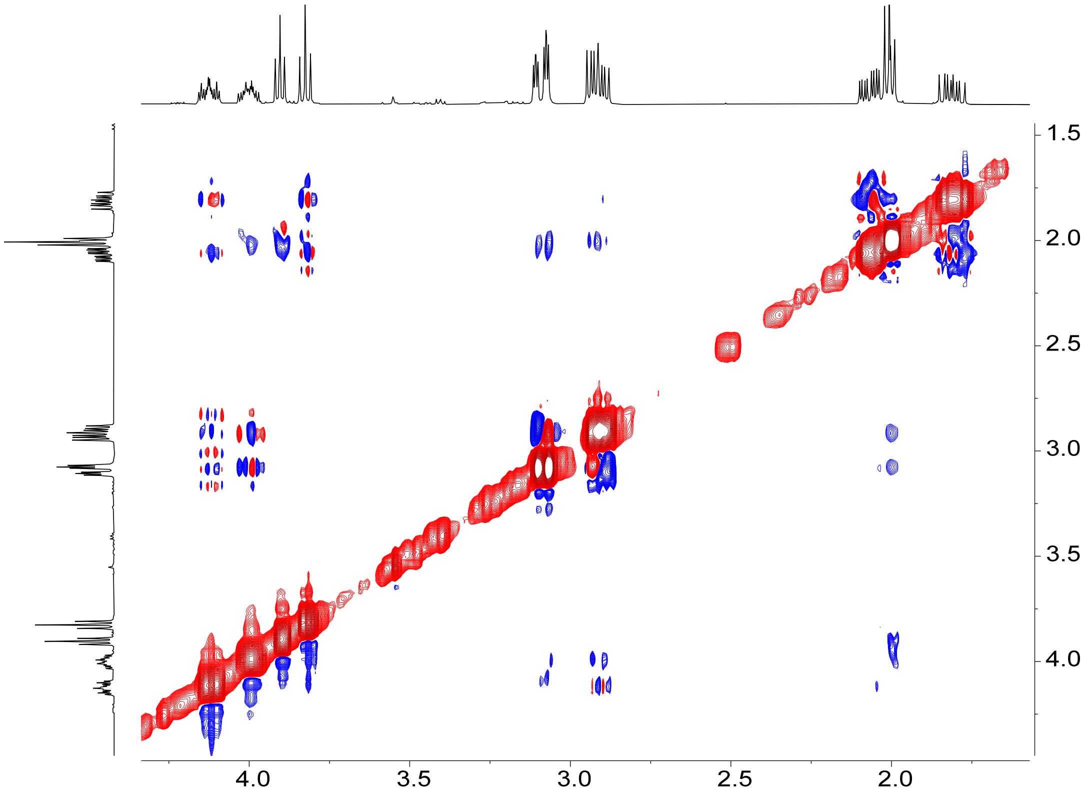

Figure S65. NMR spectra (D_2O) of (5*R*/*S*)-**18n** (i.e. Cbz and PheAc-removal of (5*R*/*S*)-**14n** mixture): a) ^{1}H, b) ^{13}C, c) COSY, d) HSQC, e) HMBC and f) NOESY.

a)

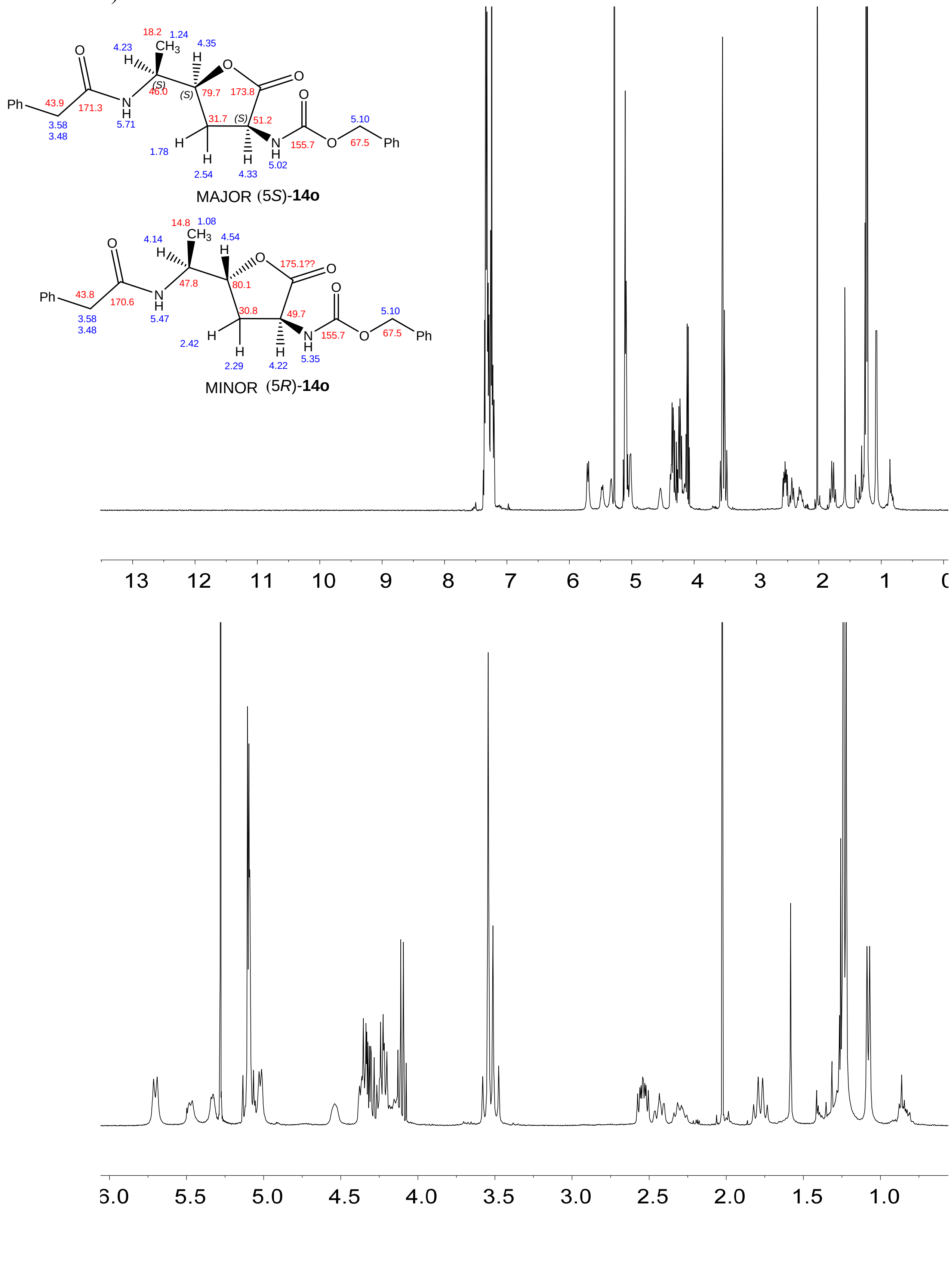

b)

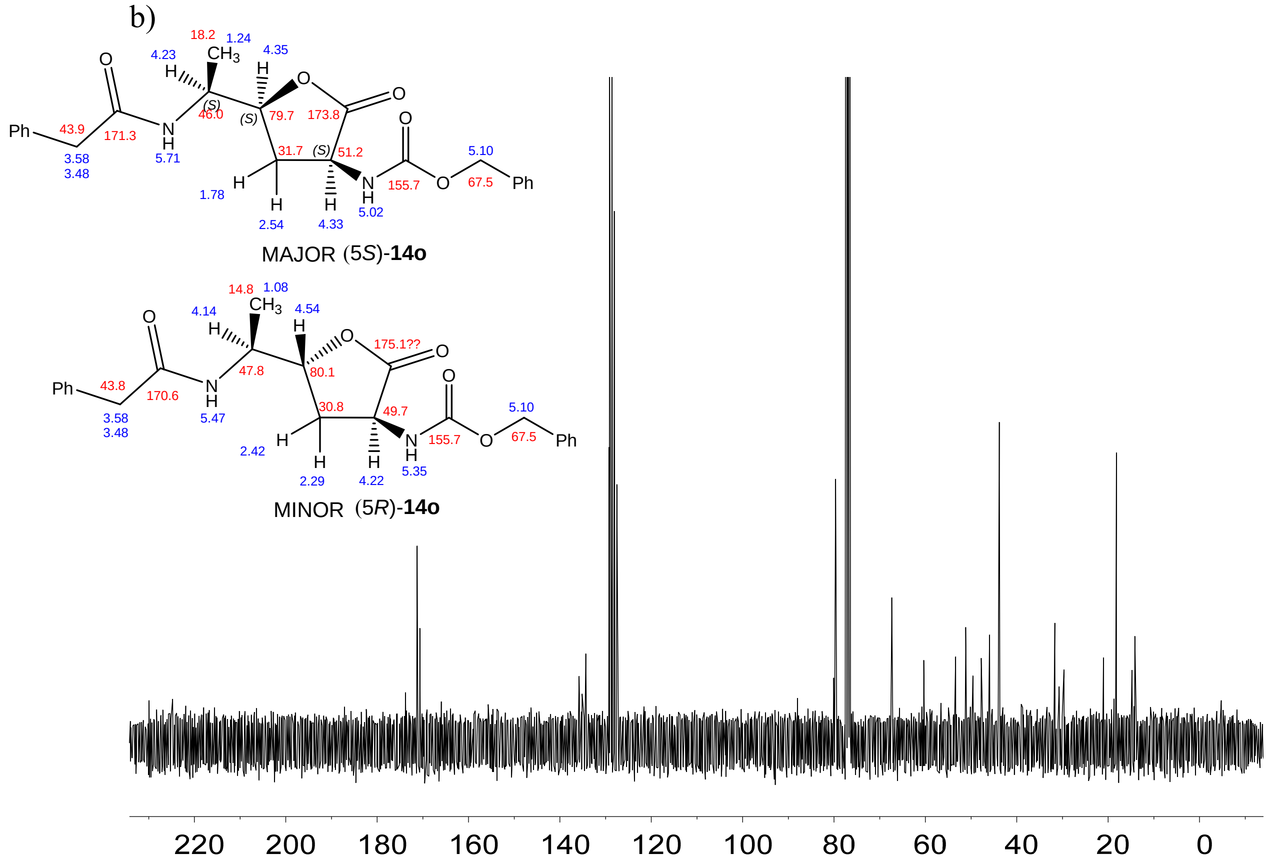
18.2
1.24
4.23
CH3
4.35
O
H
H
O
O
Ph
43.9
171.3
N
H
(S)
46.0
(S)
79.7
173.8
5.71
3.58
3.48
31.7
(S)
51.2
O
5.10
H
N
H
155.7
O
67.5
Ph
1.78
H
H
5.02
2.54
4.33
MAJOR (5S)-14o
14.8
1.08
4.14
CH3
4.54
O
H
H
O
175.1??
O
O
Ph
43.8
170.6
N
H
47.8
80.1
3.58
3.48
5.47
30.8
49.7
5.10
2.42
H
N
H
155.7
O
67.5
Ph
H
H
2.29
4.22
5.35
MINOR (5R)-14o
220
200
180
160
140
120
100
80
60
40
20
0

c)

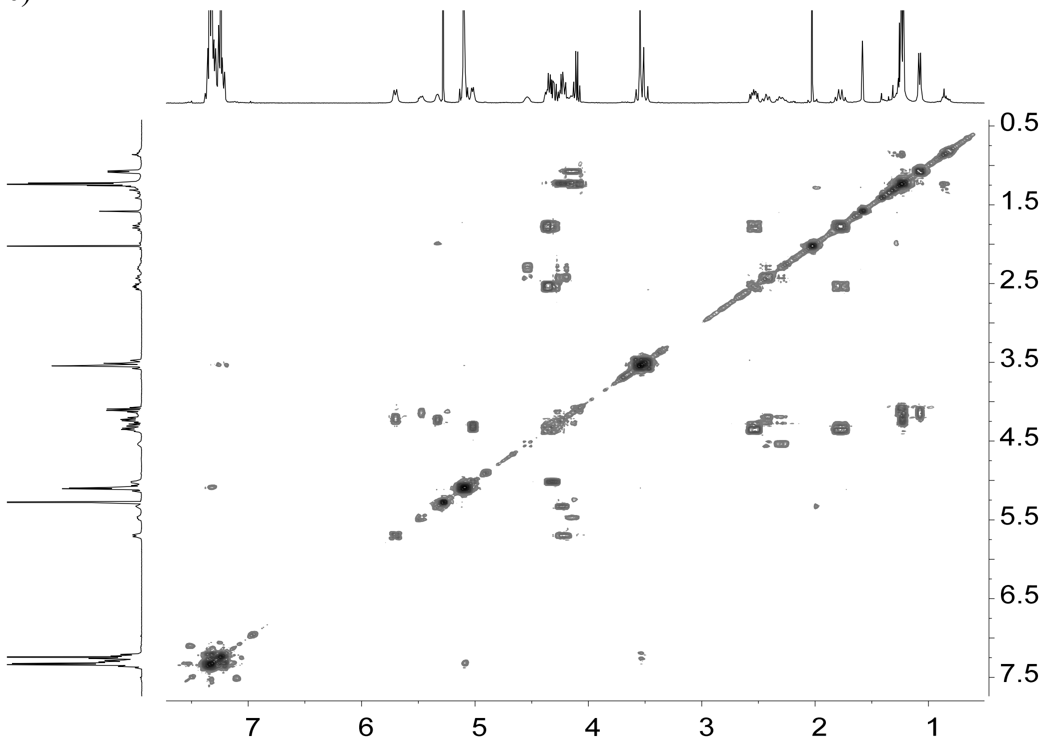
0.5
1.5
2.5
3.5
4.5
5.5
6.5
7.5
7
6
5
4
3
2
1

d)

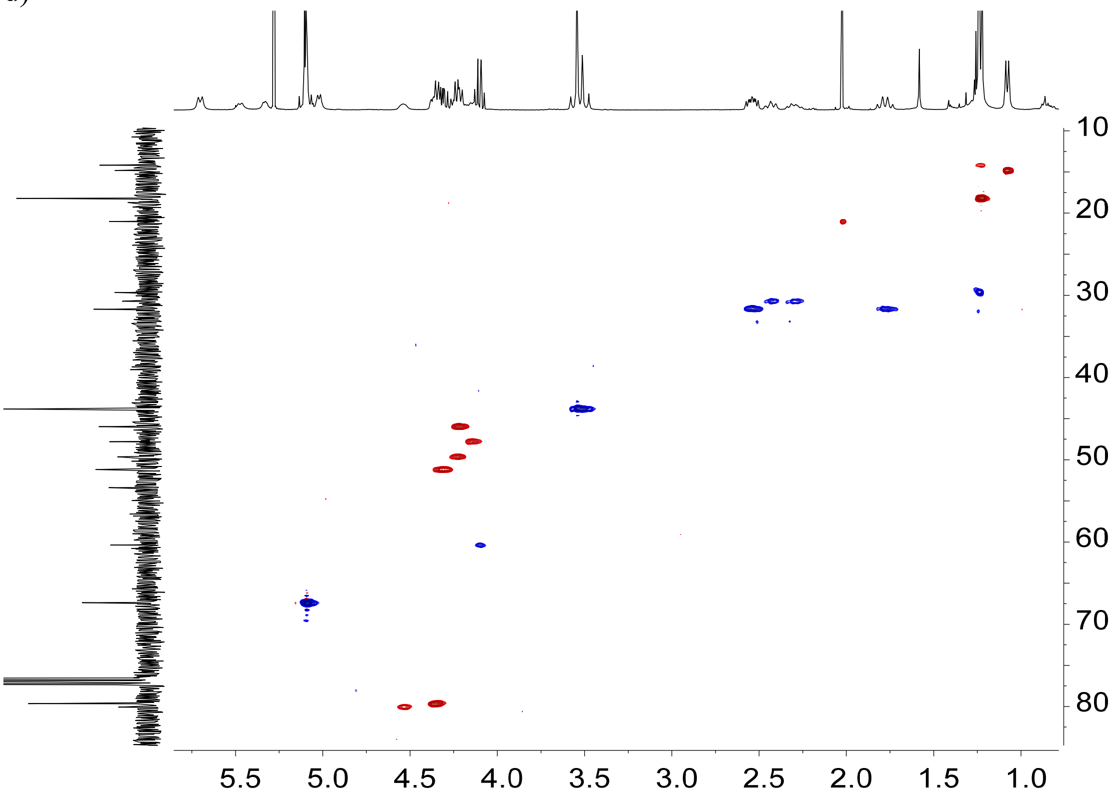

10
20
30
40
50
60
70
80
5.5
5.0
4.5
4.0
3.5
3.0
2.5
2.0
1.5
1.0

e)

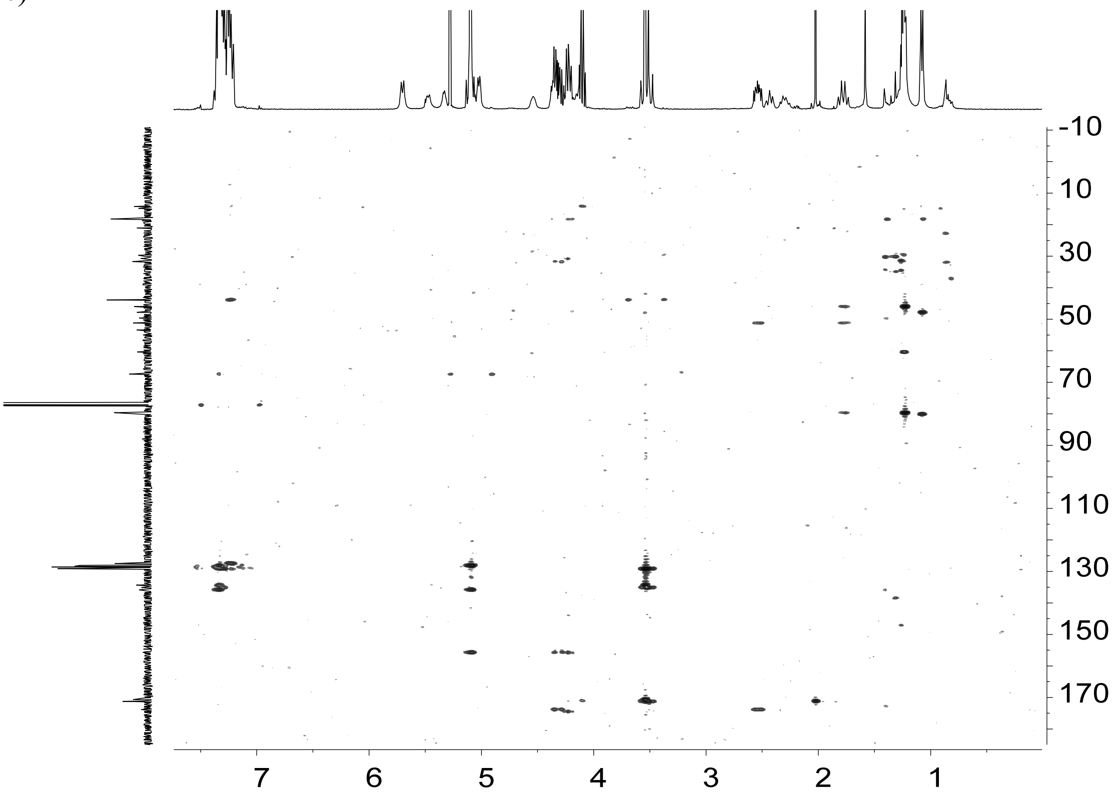

-10
10
30
50
70
90
110
130
150
170
7
6
5
4
3
2
1

f)

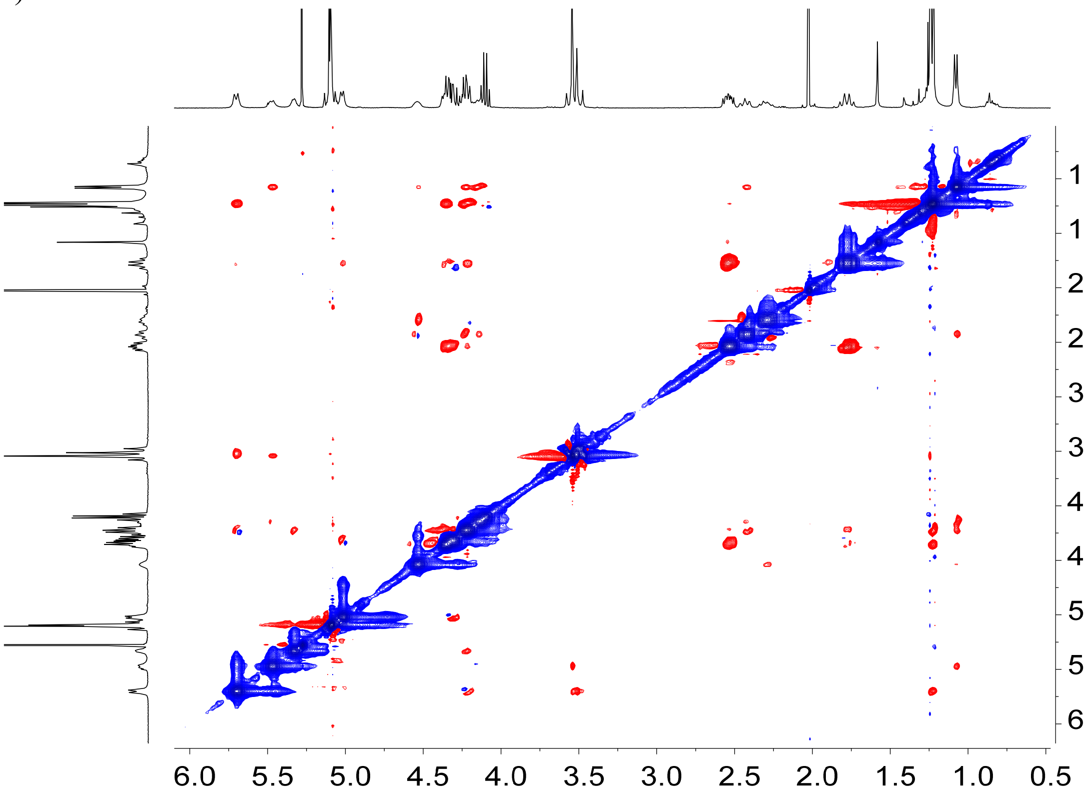

Figure S66. NMR spectra ($CDCl_3$) of (5*R*/*S*)-**14o** diasteromeric mixture: a) ^{1}H, b) ^{13}C, c) COSY, d) HSQC, e) HMBC and f) NOESY.

a)

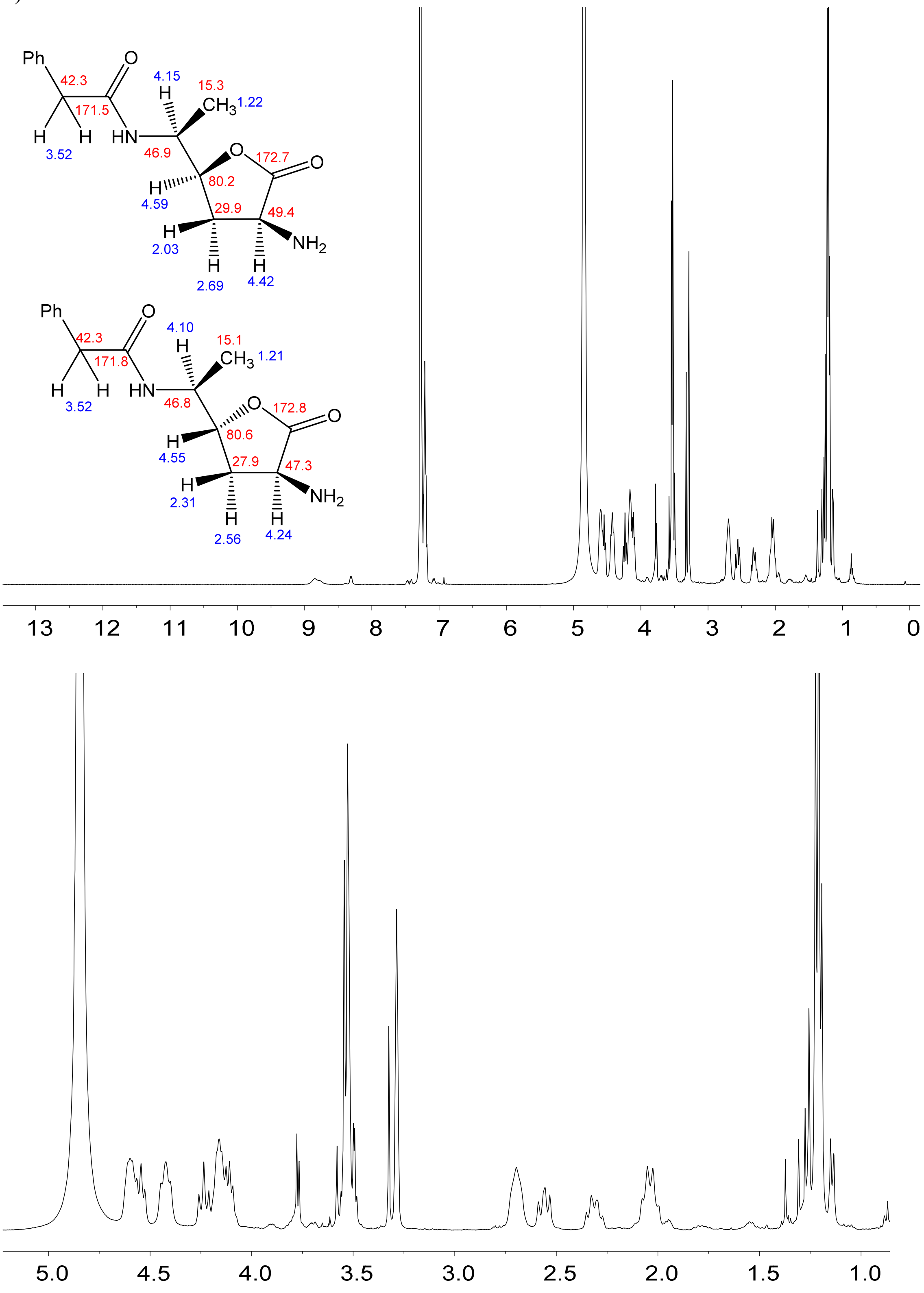
Ph
O
42.3
171.5
H
H
3.52
HN
46.9
4.15
H
15.3
CH3
1.22
O
172.7
O
H
80.2
4.59
29.9
49.4
H
2.03
H
2.69
H
4.42
NH2
Ph
O
42.3
171.8
H
H
3.52
HN
46.8
4.10
H
15.1
CH3
1.21
O
172.8
O
H
80.6
4.55
27.9
47.3
H
2.31
H
2.56
H
4.24
NH2
13
12
11
10
9
8
7
6
5
4
3
2
1
0
5.0
4.5
4.0
3.5
3.0
2.5
2.0
1.5
1.0

b)

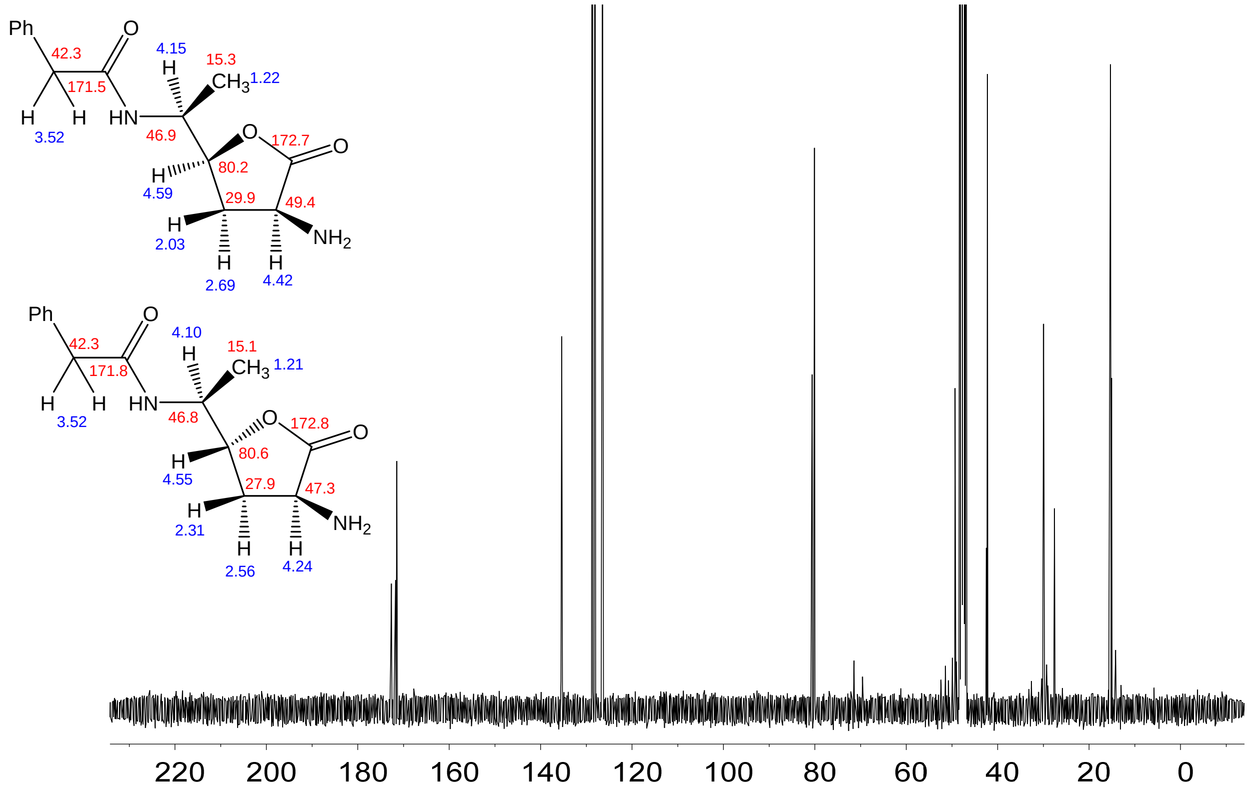
Ph
O
42.3
171.5
4.15
15.3
H
CH_3 1.22
H H HN
3.52
46.9
O
172.7
O
H
80.2
4.59
29.9
49.4
H
2.03
NH_2
H H
2.69 4.42
Ph
O
42.3
4.10
15.1
H
CH_3 1.21
171.8
H H HN
3.52
46.8
O
172.8
O
H
80.6
4.55
27.9
47.3
H
2.31
NH_2
H H
2.56 4.24
220 200 180 160 140 120 100 80 60 40 20 0

c)

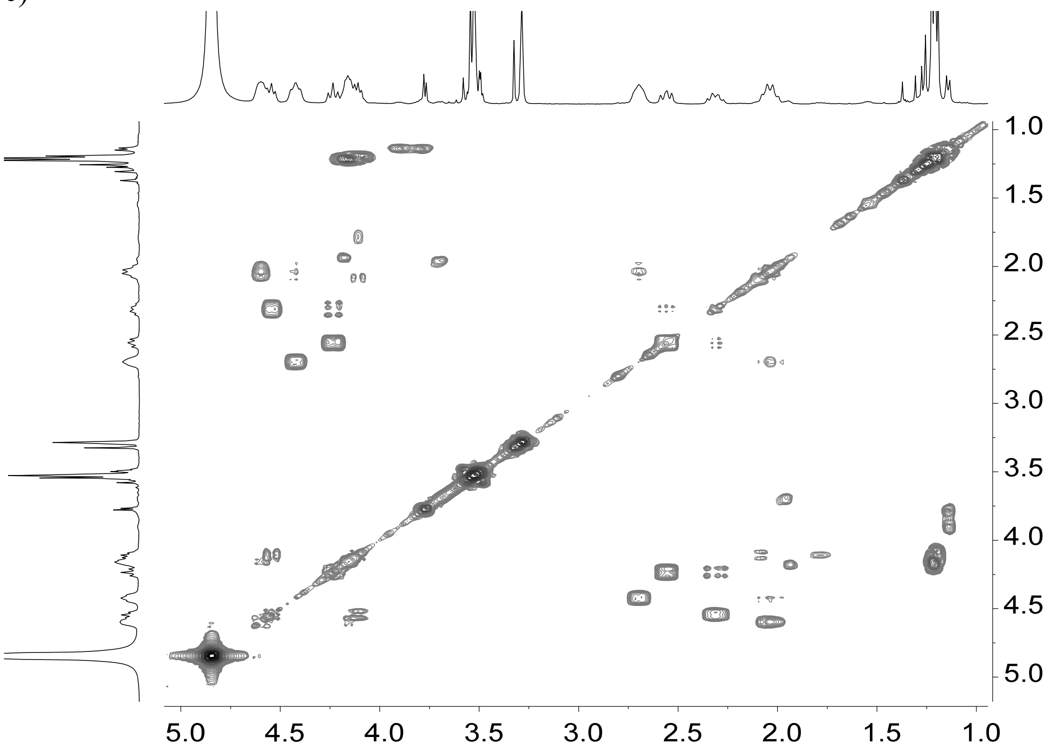
1.0
1.5
2.0
2.5
3.0
3.5
4.0
4.5
5.0
5.0 4.5 4.0 3.5 3.0 2.5 2.0 1.5 1.0

d)

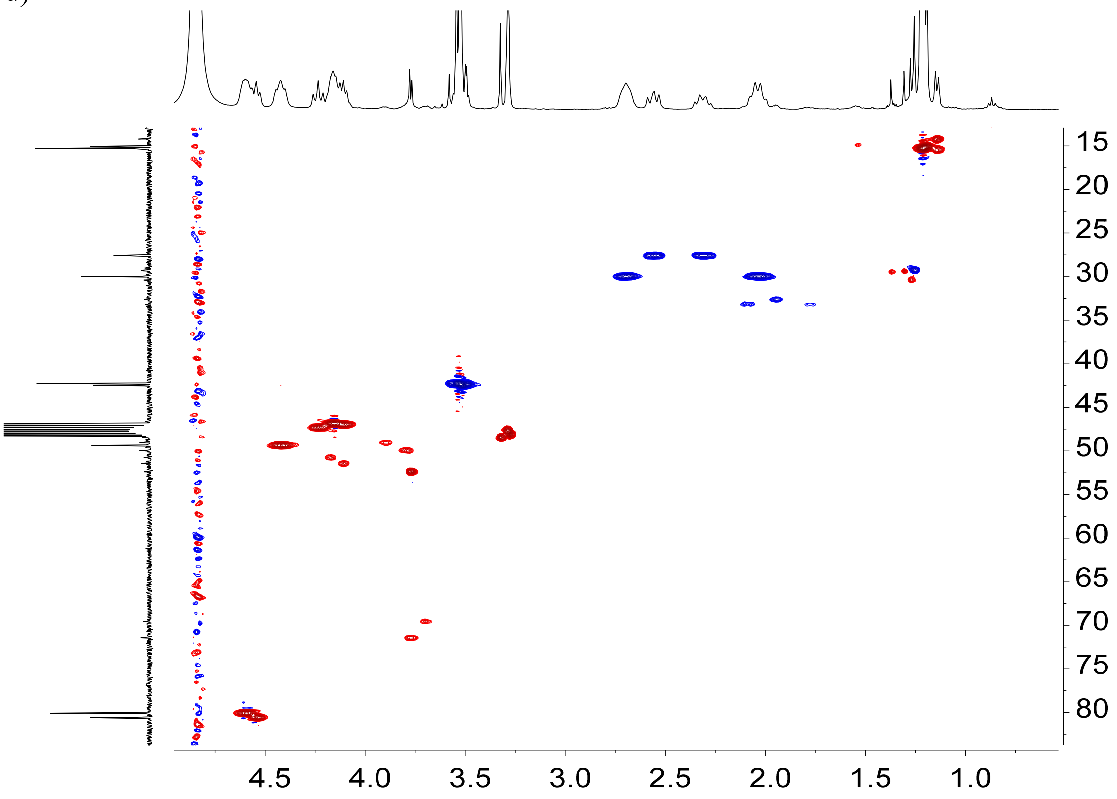

Figure S67. NMR spectra (CD_3OD) of (5*R*/*S*)-**17o** (i.e. Cbz-removal of (5*R*/*S*)-**14o** mixture): a) ^{1}H, b) ^{13}C, c) COSY, d) HSQC.

a)

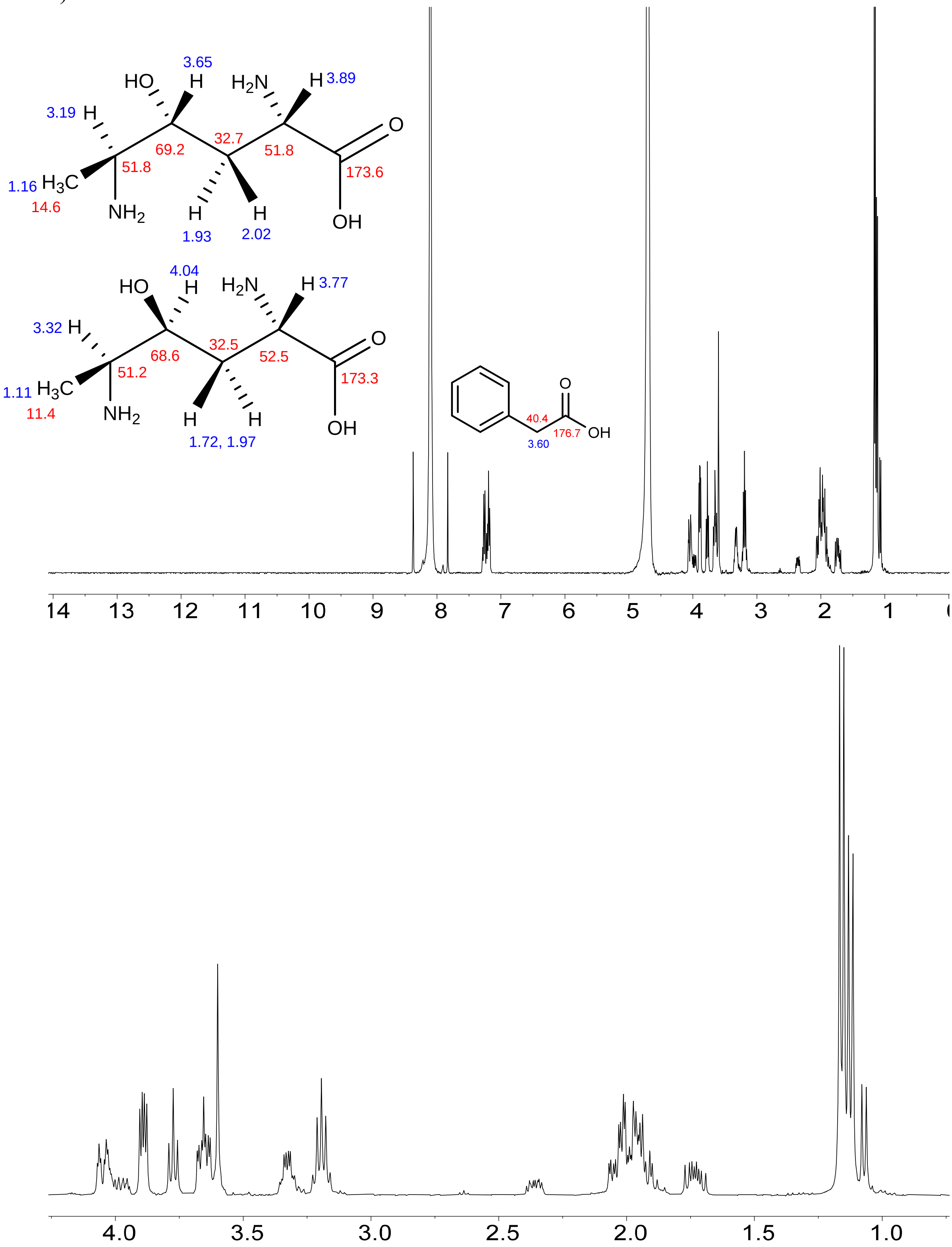
3.65
HO
H
H2N
H 3.89
3.19 H
O
69.2
32.7
51.8
51.8
173.6
1.16 H3C
14.6
NH2
H
H
OH
1.93
2.02
4.04
HO
H
H2N
H 3.77
3.32 H
O
32.5
68.6
52.5
51.2
173.3
1.11 H3C
11.4
NH2
H
H
OH
1.72, 1.97
O
40.4
176.7
OH
3.60
14
13
12
11
10
9
8
7
6
5
4
3
2
1
4.0
3.5
3.0
2.5
2.0
1.5
1.0

b)

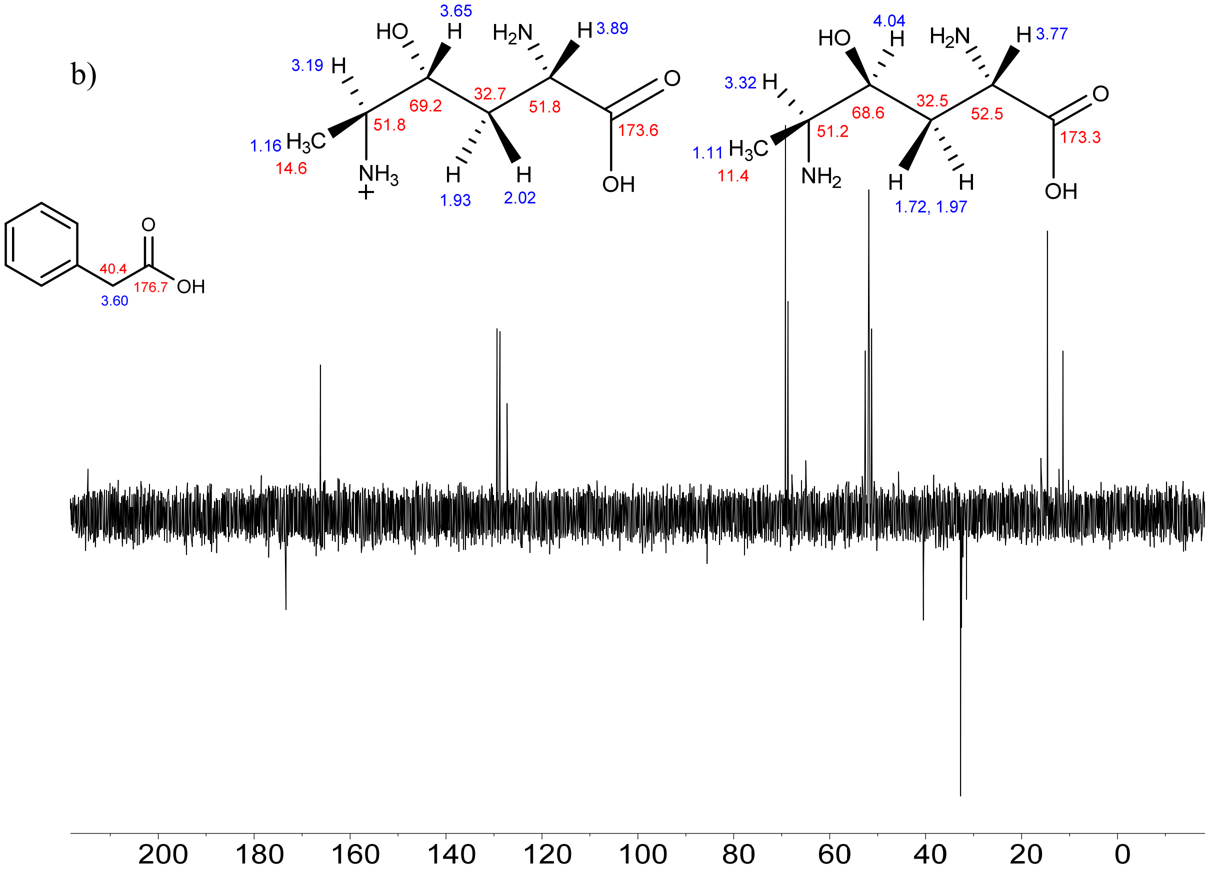

HO
H
3.65
H2N
H 3.89
3.19 H
69.2
32.7
51.8
O
1.16 H3C
51.8
173.6
14.6
NH3
+
H
H
1.93
2.02
OH
HO
4.04
H
H2N
H 3.77
3.32 H
68.6
32.5
52.5
O
51.2
173.3
1.11 H3C
11.4
NH2
H
H
1.72, 1.97
OH
O
40.4
176.7
OH
3.60
200
180
160
140
120
100
80
60
40
20
0

c)

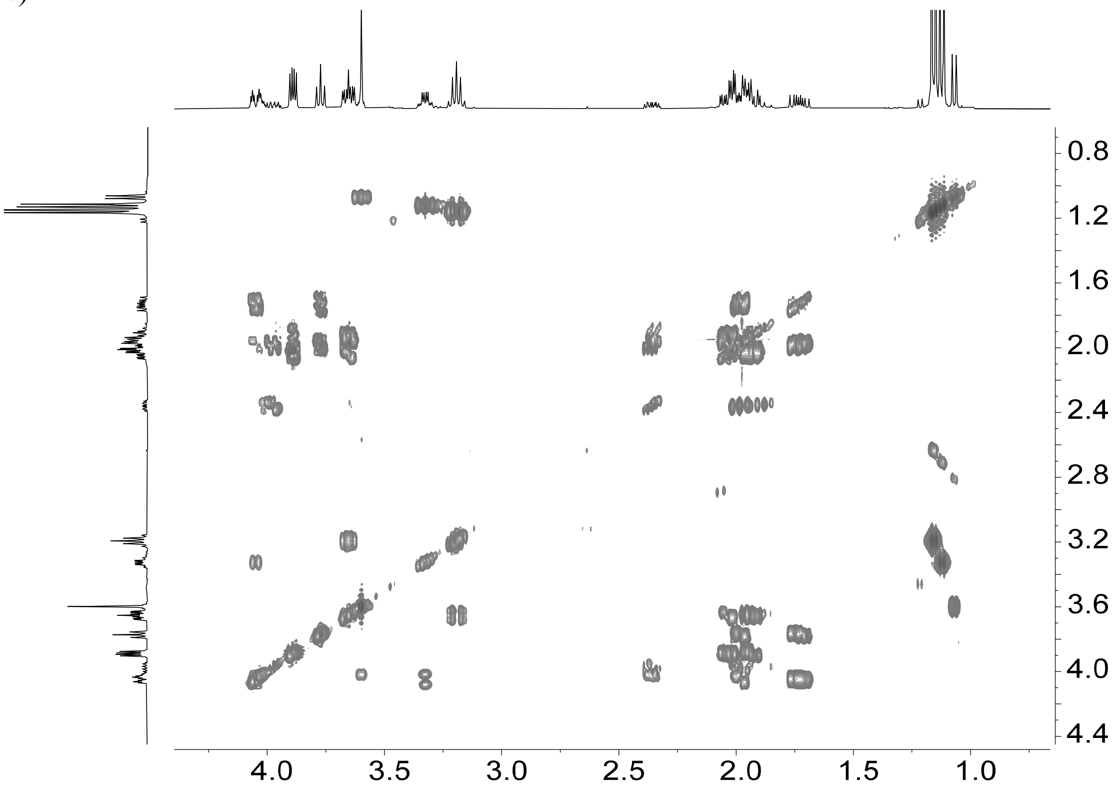

0.8
1.2
1.6
2.0
2.4
2.8
3.2
3.6
4.0
4.4
4.0
3.5
3.0
2.5
2.0
1.5
1.0

d)

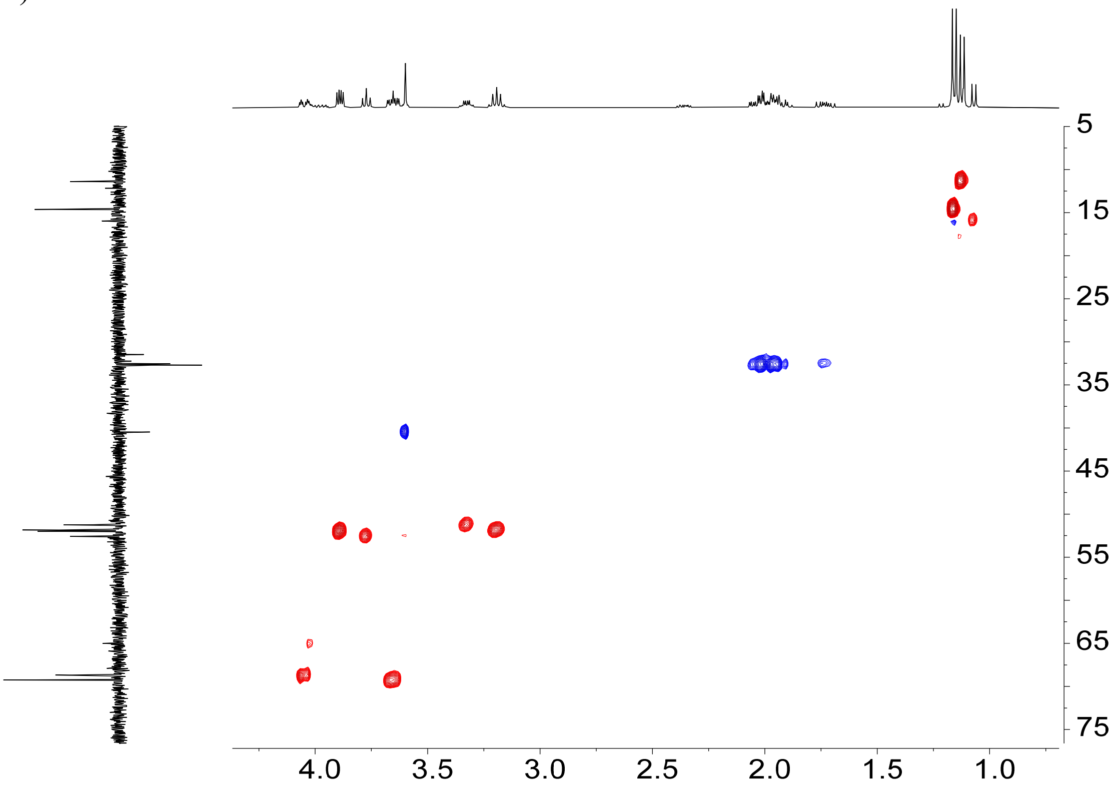

5
15
25
35
45
55
65
75
4.0
3.5
3.0
2.5
2.0
1.5
1.0

e)

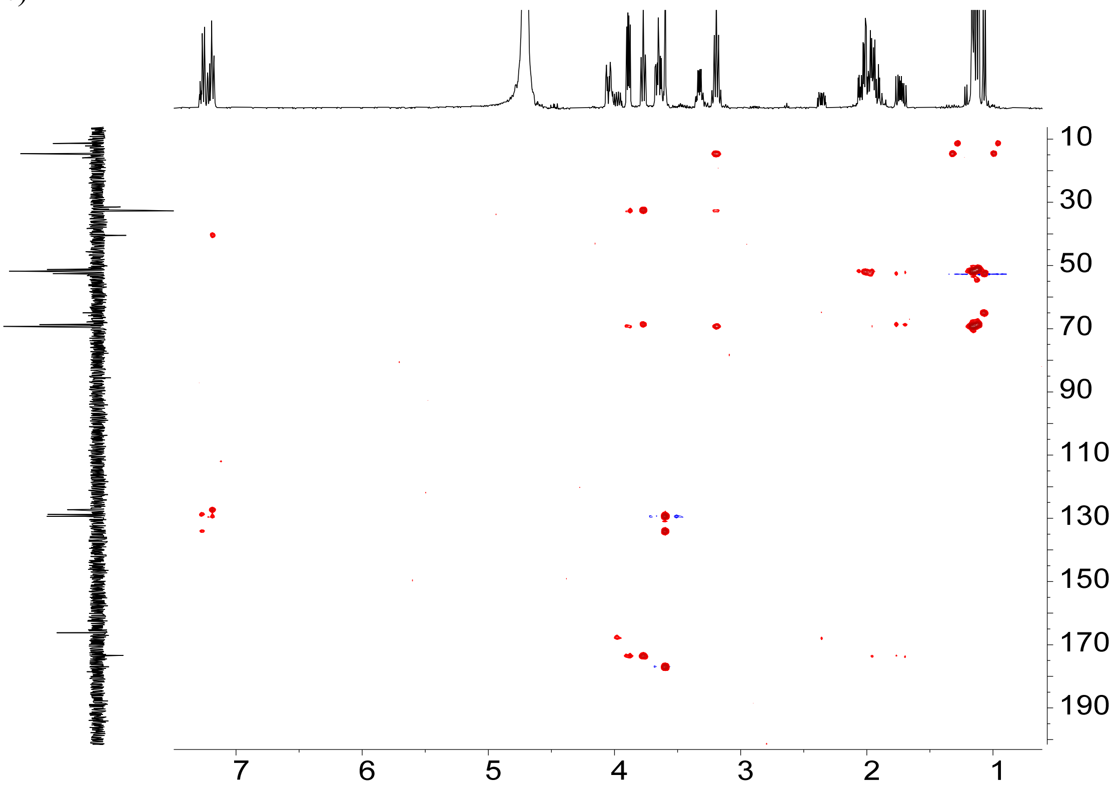

10
30
50
70
90
110
130
150
170
190
7
6
5
4
3
2
1

f)

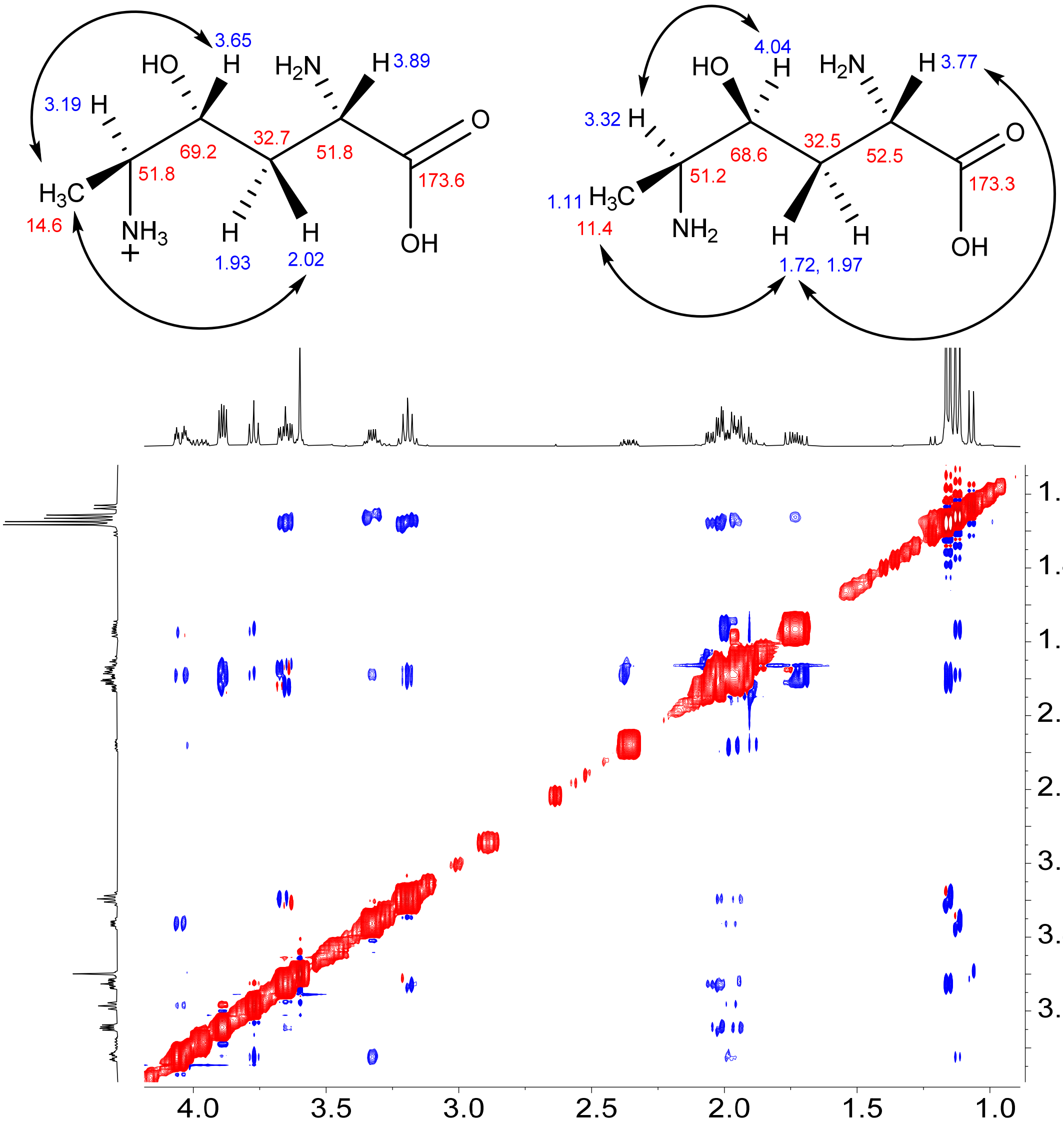

Figure S68. NMR spectra (D_2O) of (4*R*/*S*)-**14o** (i.e. Cbz and PheAc-removal of (5*R*/*S*)-**14o** mixture): a) ^{1}H, b) ^{13}C, c) COSY, d) HSQC, e) HMBC and f) NOESY.

a)

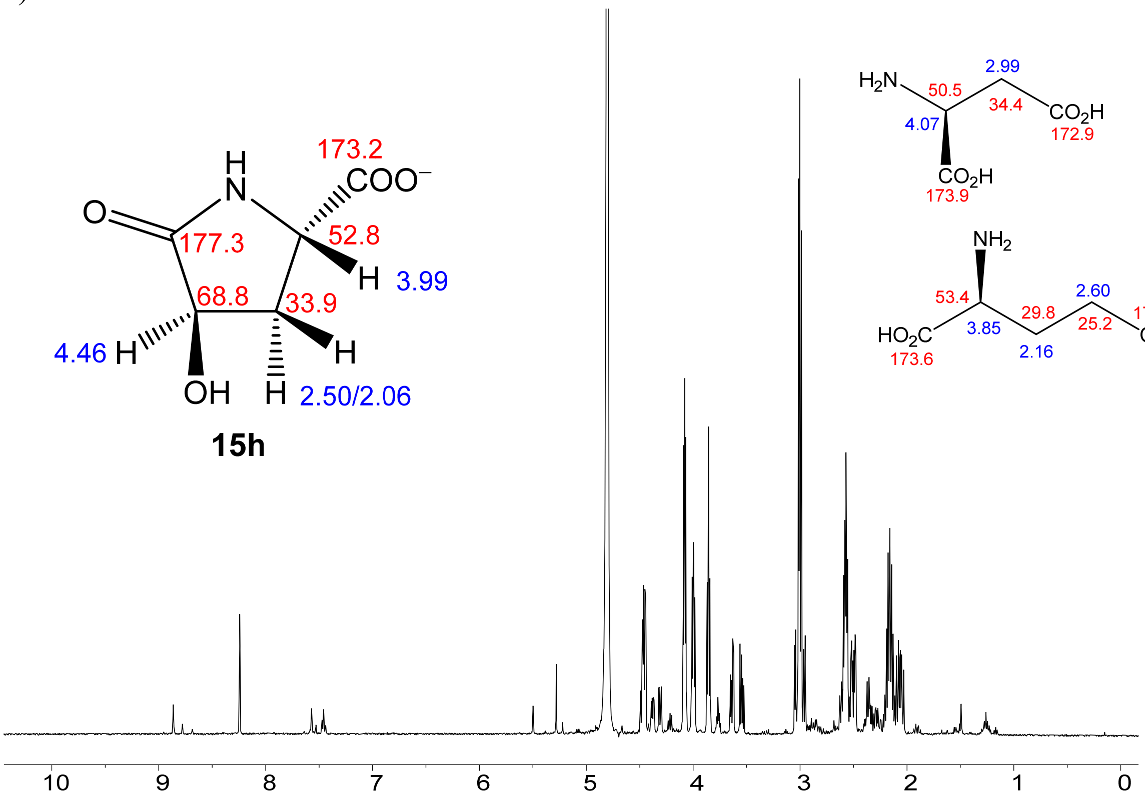
173.2
COO⁻
H
N
O
177.3
52.8
H 3.99
68.8
33.9
4.46 H
H
OH
H 2.50/2.06
15h
H2N
50.5
2.99
34.4
4.07
CO2H
172.9
CO2H
173.9
NH2
53.4
2.60
29.8
176.7
HO2C
3.85
25.2
CO2H
173.6
2.16
10
9
8
7
6
5
4
3
2
1
0

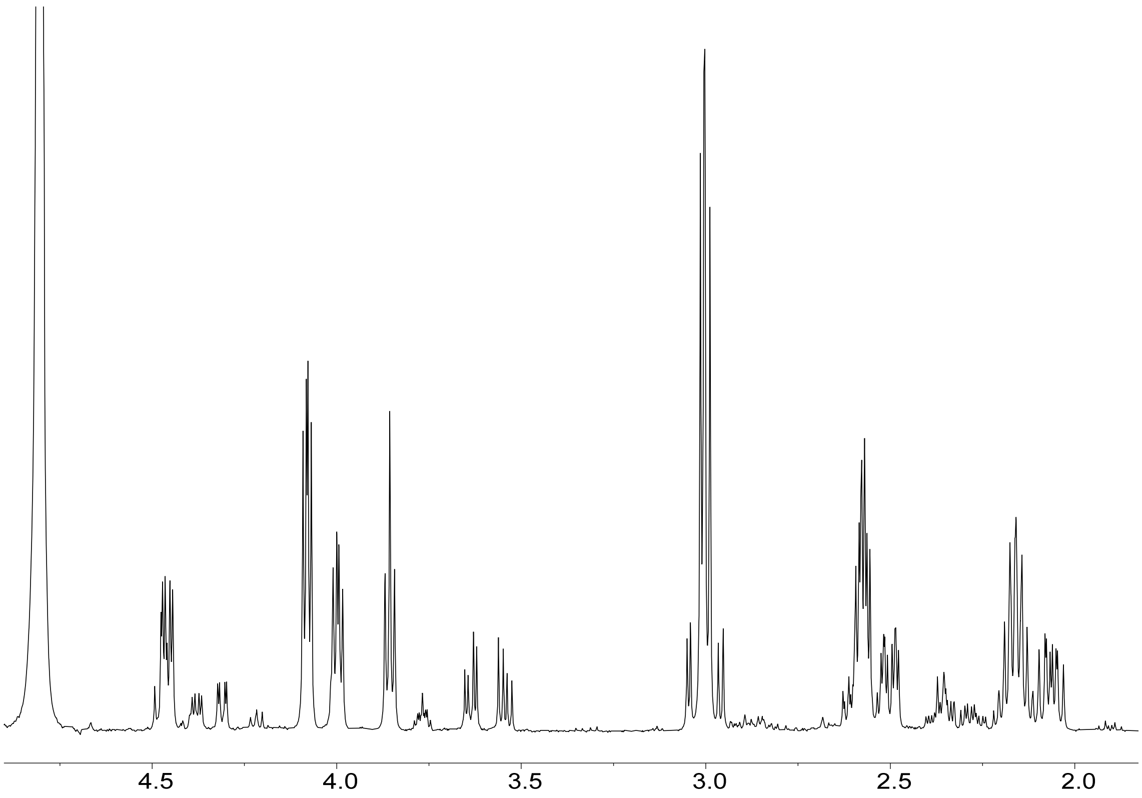
4.5
4.0
3.5
3.0
2.5
2.0

b)

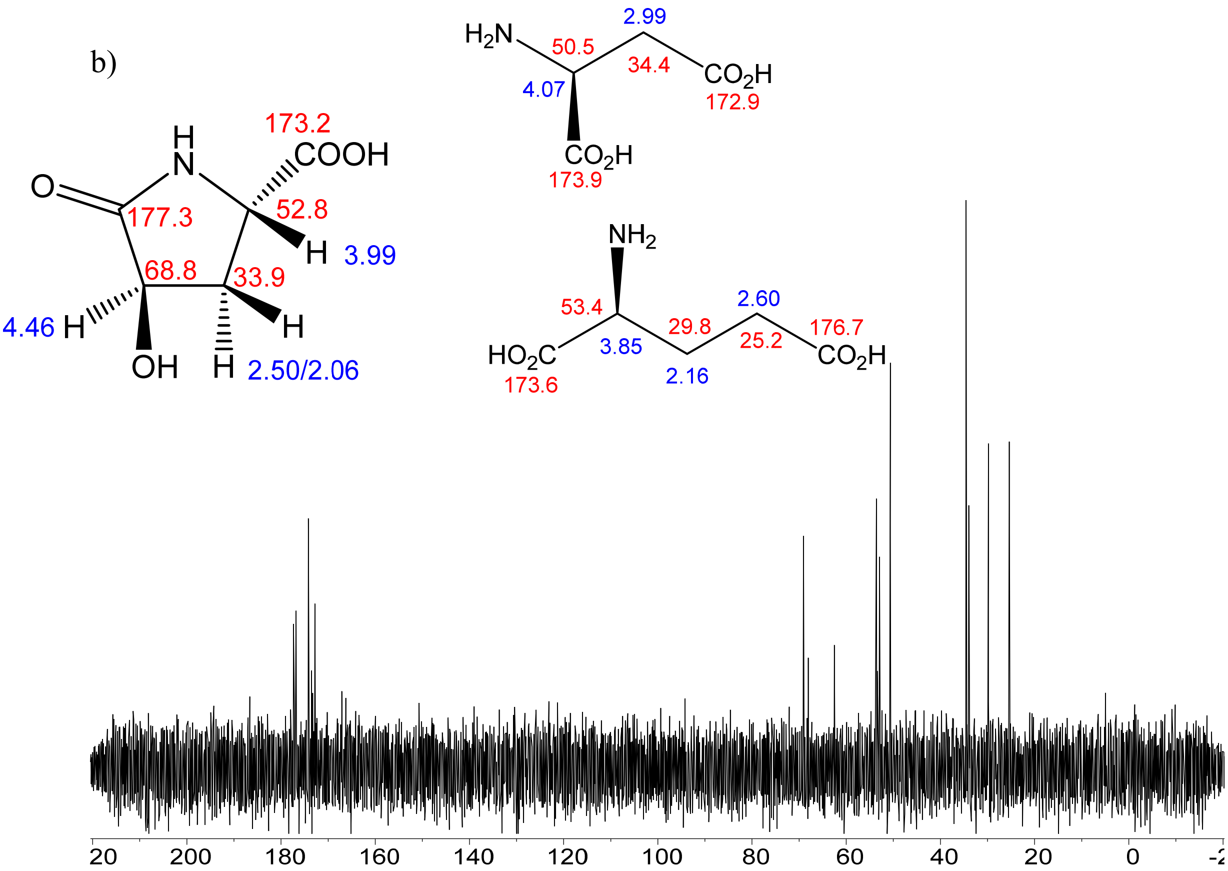
173.2
COOH
177.3
52.8
H 3.99
68.8
33.9
4.46 H
OH
H 2.50/2.06
H2N
2.99
50.5
34.4
4.07
CO2H
172.9
CO2H
173.9
NH2
53.4
2.60
29.8
176.7
25.2
HO2C
3.85
CO2H
173.6
2.16
20
200
180
160
140
120
100
80
60
40
20
0

c)

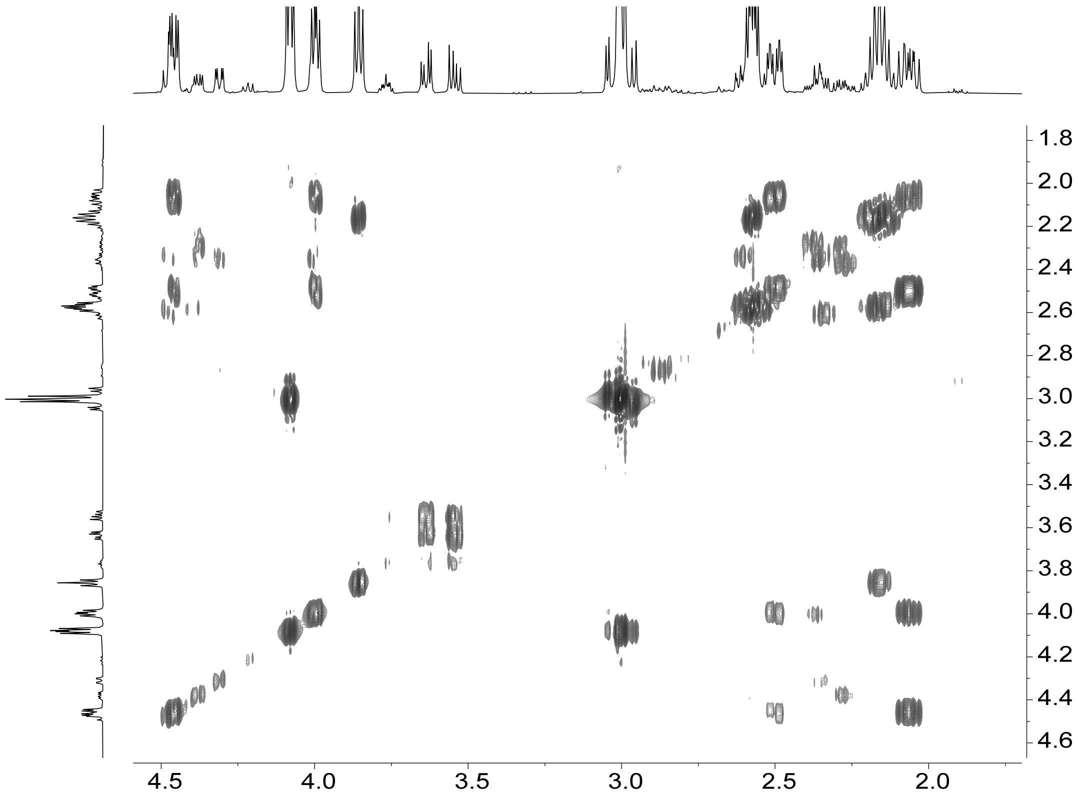
1.8
2.0
2.2
2.4
2.6
2.8
3.0
3.2
3.4
3.6
3.8
4.0
4.2
4.4
4.6
4.5
4.0
3.5
3.0
2.5
2.0

d)

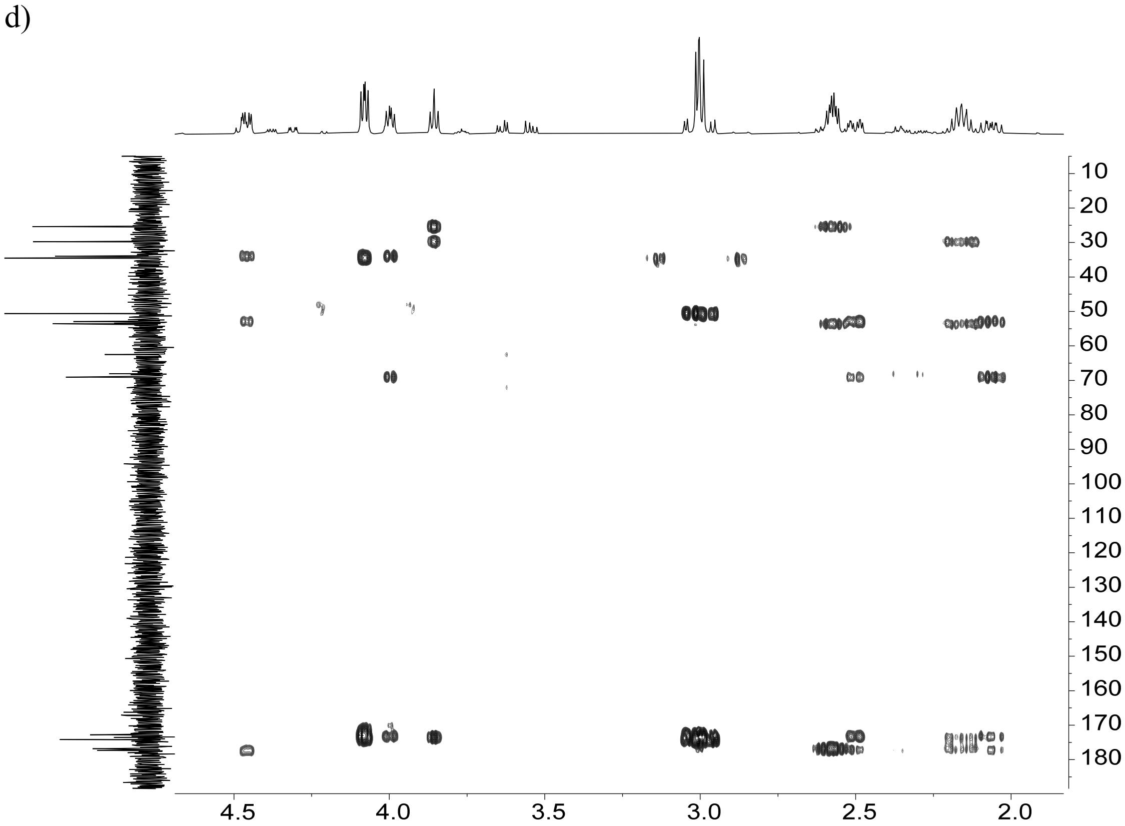

Figure S69. NMR spectra (D_2O) of **15h**: a) ^1H, b) ^{13}C, c) COSY, d) HMBC.

a)

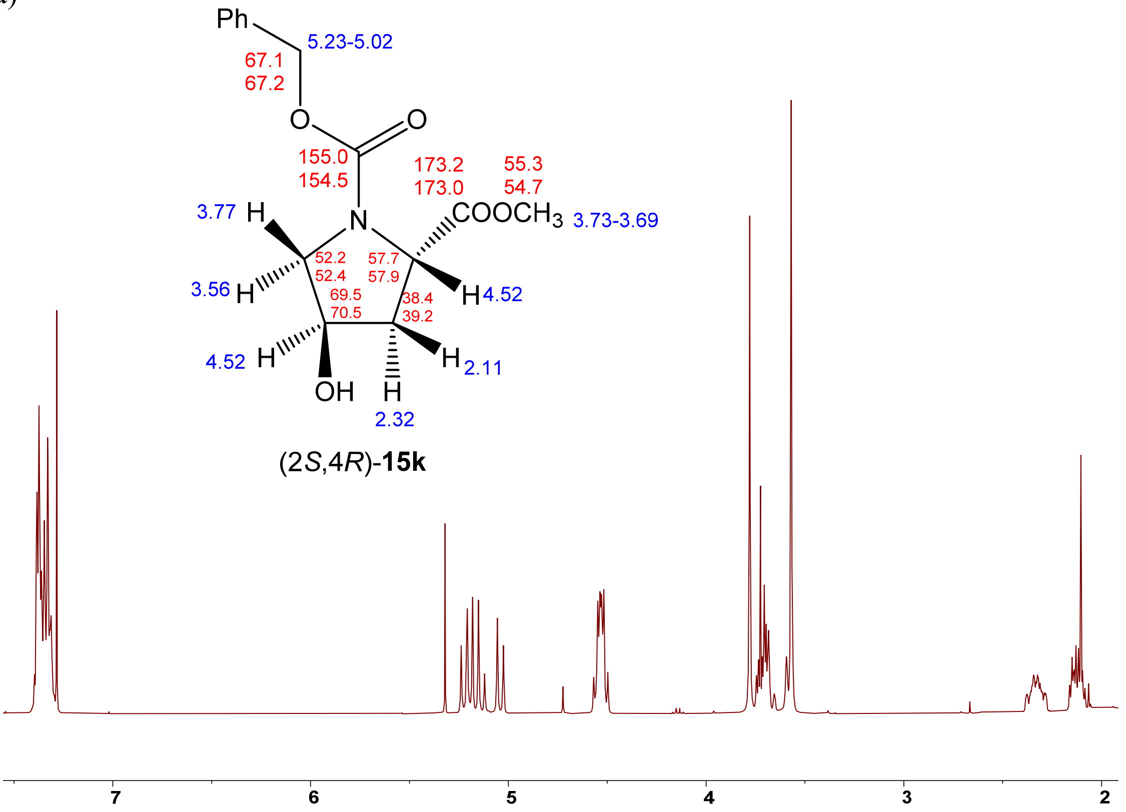
Ph
5.23-5.02
67.1
67.2
O
O
155.0
154.5
173.2
173.0
55.3
54.7
3.77 H
N
COOCH3 3.73-3.69
52.2
52.4
57.7
57.9
3.56 H
69.5
70.5
38.4
39.2
H4.52
4.52 H
H 2.11
OH
H
2.32
(2S,4R)-15k
7
6
5
4
3
2

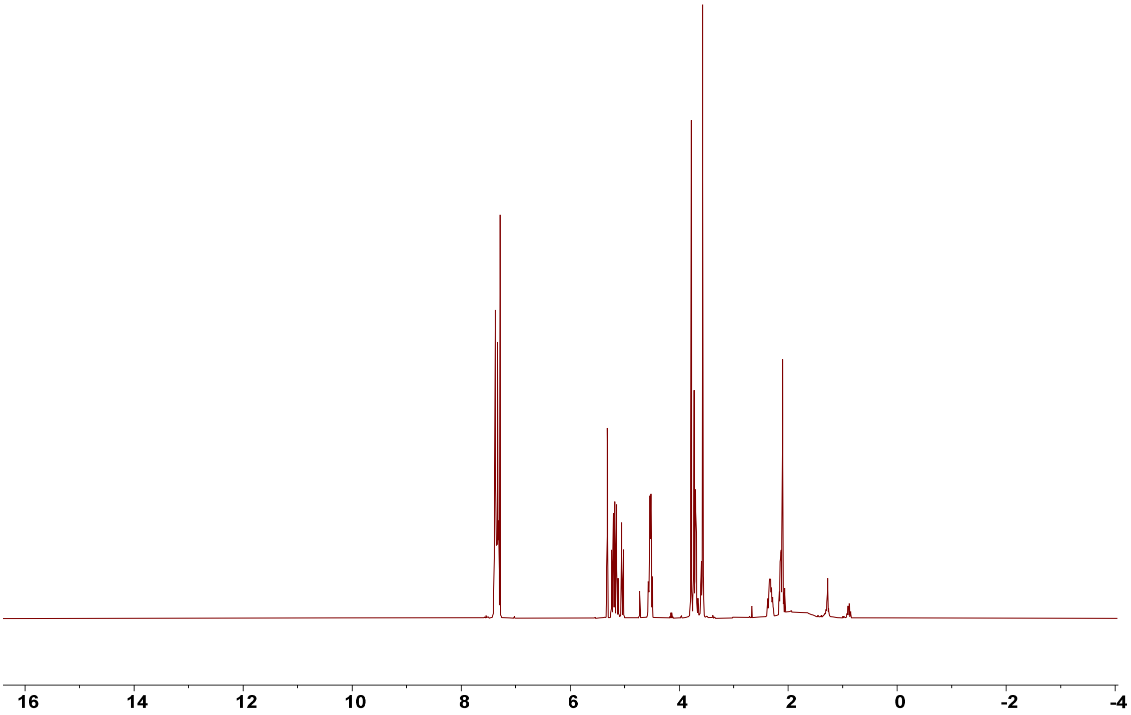
16
14
12
10
8
6
4
2
0
-2
-4

b)

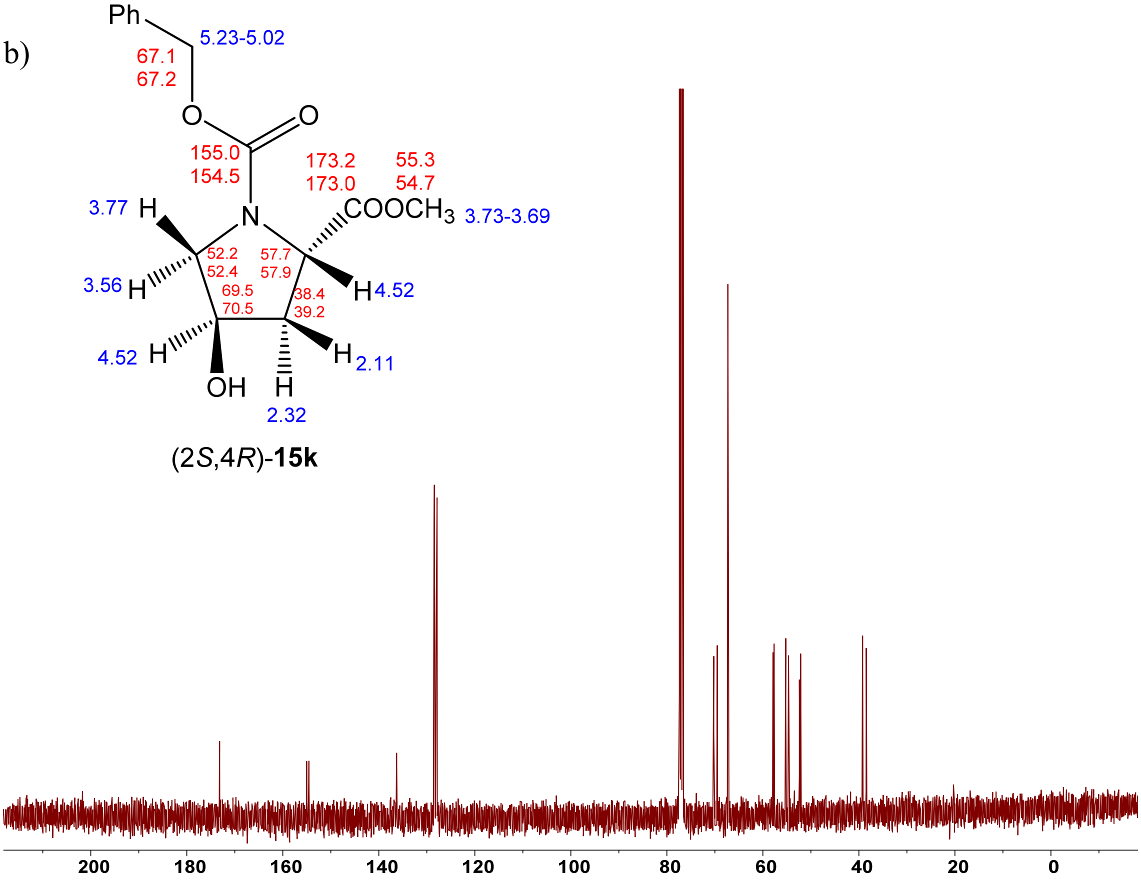

Ph
5.23-5.02
67.1
67.2
O
O
155.0
154.5
173.2
173.0
55.3
54.7
3.77 H
N
COOCH3 3.73-3.69
3.56 H
52.2
52.4
57.7
57.9
69.5
70.5
38.4
39.2
H4.52
4.52 H
H2.11
OH
H
2.32
(2S,4R)-15k
200
180
160
140
120
100
80
60
40
20
0

c)

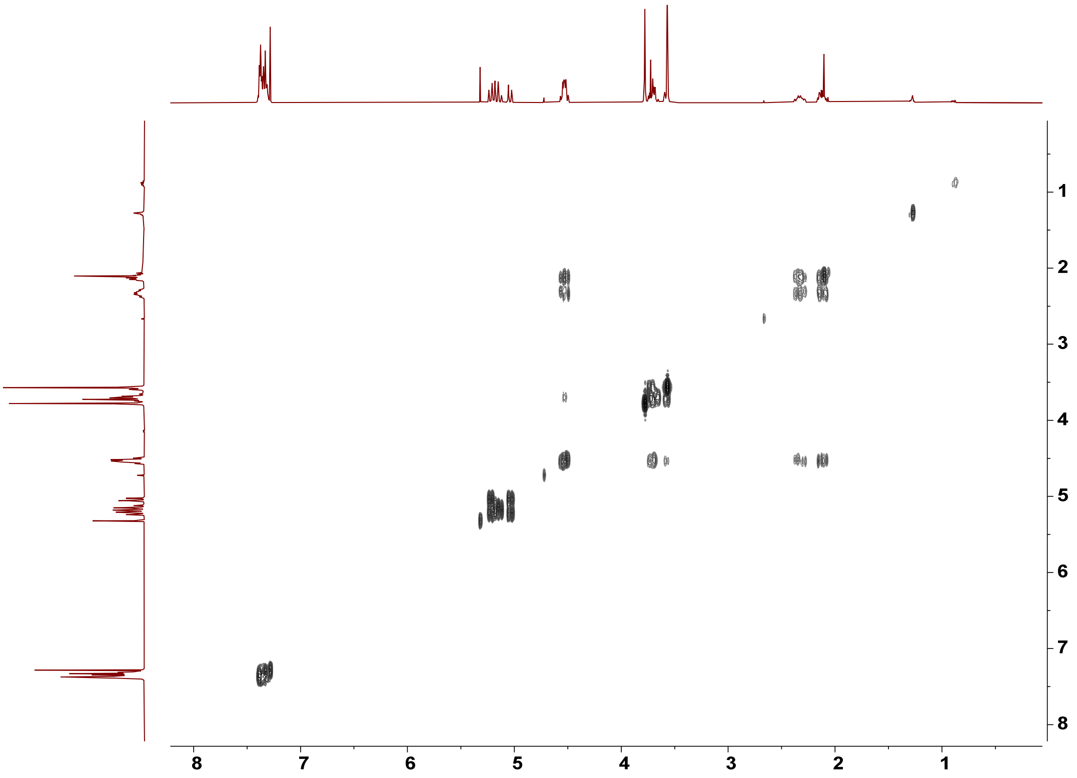

8
7
6
5
4
3
2
1

d)

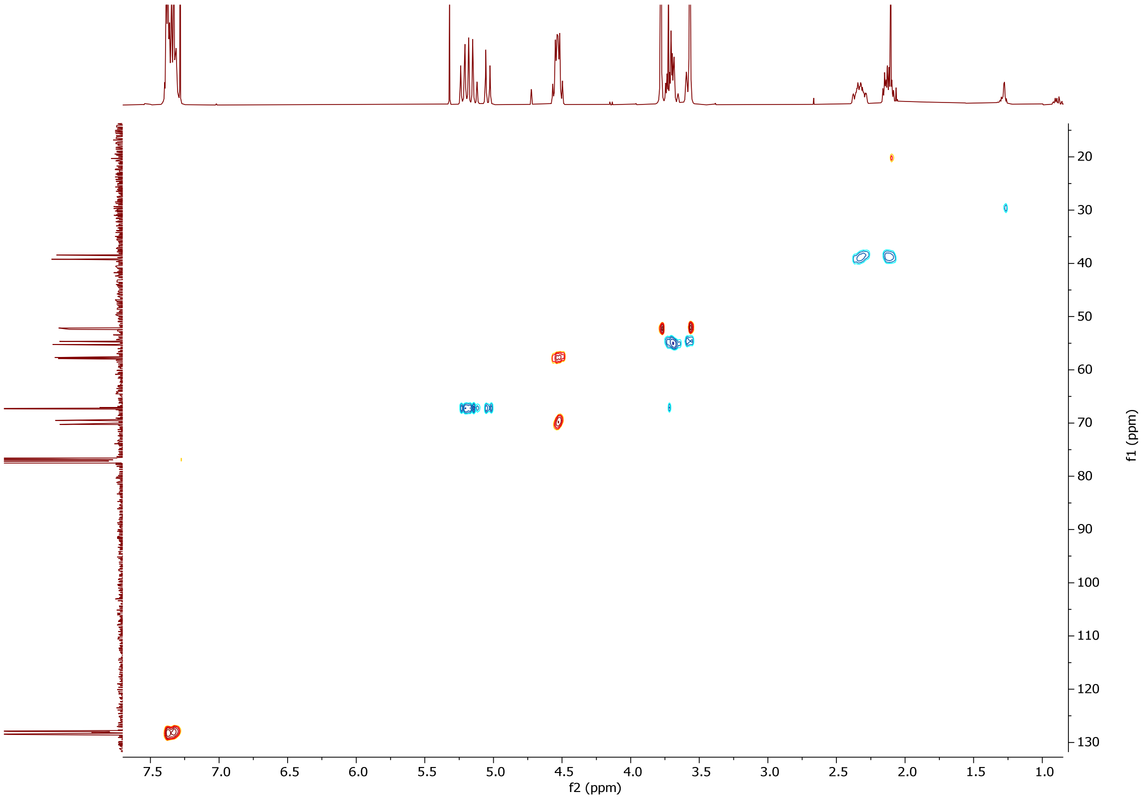

Figure S70. NMR spectra ($CDCl_3$) of (2*S*,4*R*)-**15k**, chemical synthesis: a) ^{1}H, b) ^{13}C, c) COSY, d) HSQC.

a)

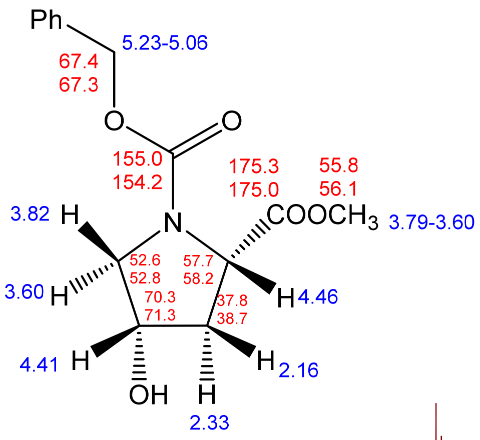
Ph
5.23-5.06
67.4
67.3
O
O
155.0
154.2
175.3
175.0
55.8
56.1
3.82 H
N
COOCH3 3.79-3.60
52.6
52.8
57.7
58.2
3.60 H
70.3
71.3
37.8
38.7
H 4.46
4.41 H
H 2.16
OH
H
2.33

(2*S*,4*S*)-**15k**

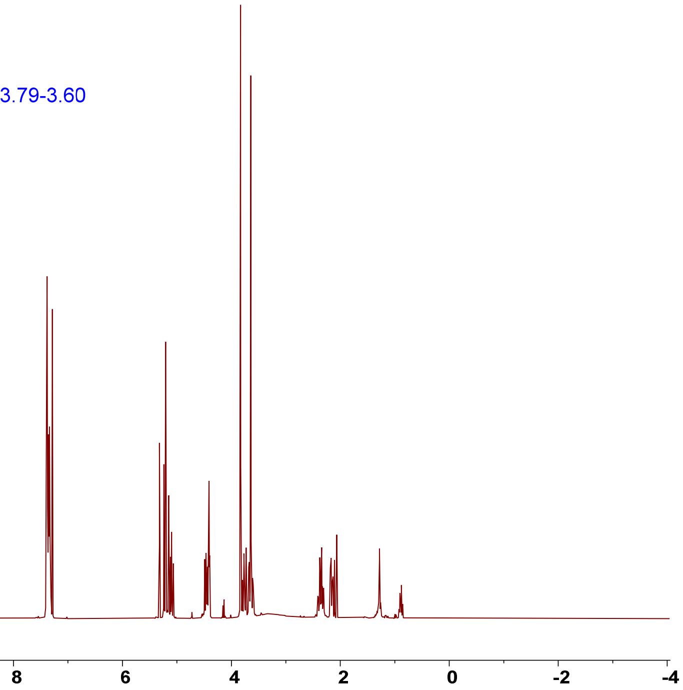
16
14
12
10
8
6
4
2
0
-2
-4

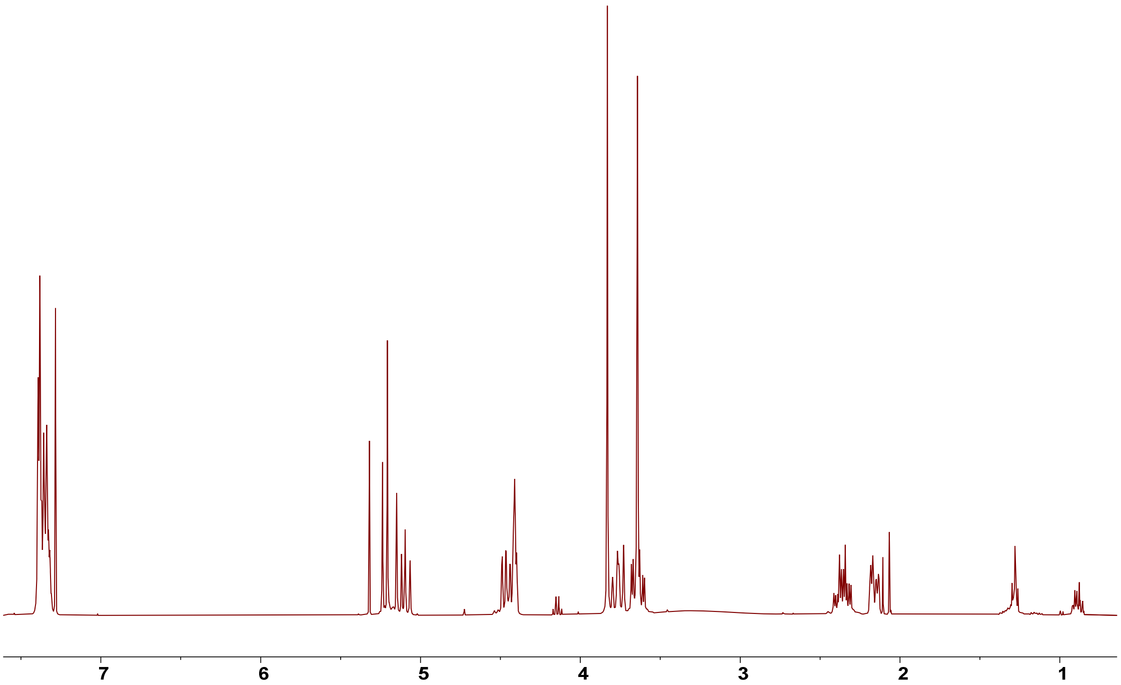
7
6
5
4
3
2
1

b)

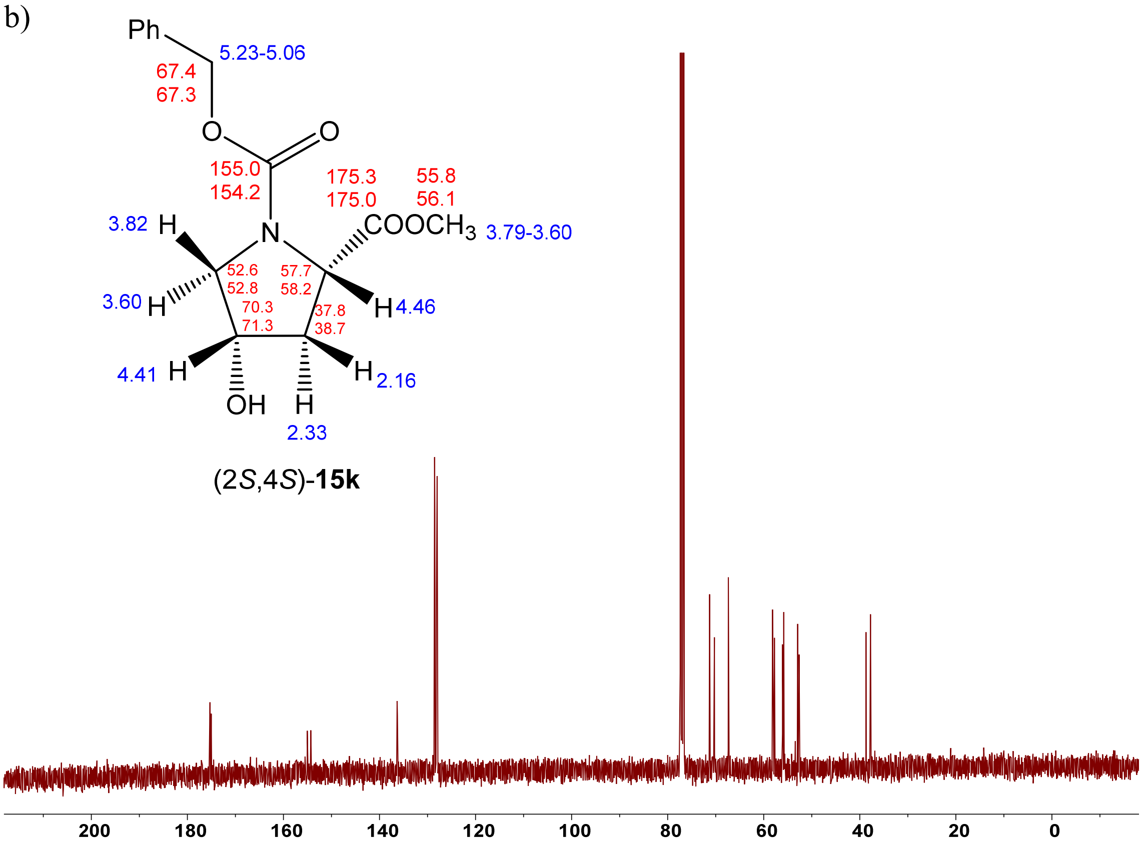
Ph
5.23-5.06
67.4
67.3
O
O
155.0
154.2
175.3
175.0
55.8
56.1
N
COOCH$_3$ 3.79-3.60
3.82 H
3.60 H
52.6
52.8
57.7
58.2
70.3
71.3
37.8
38.7
H 4.46
4.41 H
OH
H
H 2.16
2.33
(2*S*,4*S*)-**15k**
200
180
160
140
120
100
80
60
40
20
0

c)

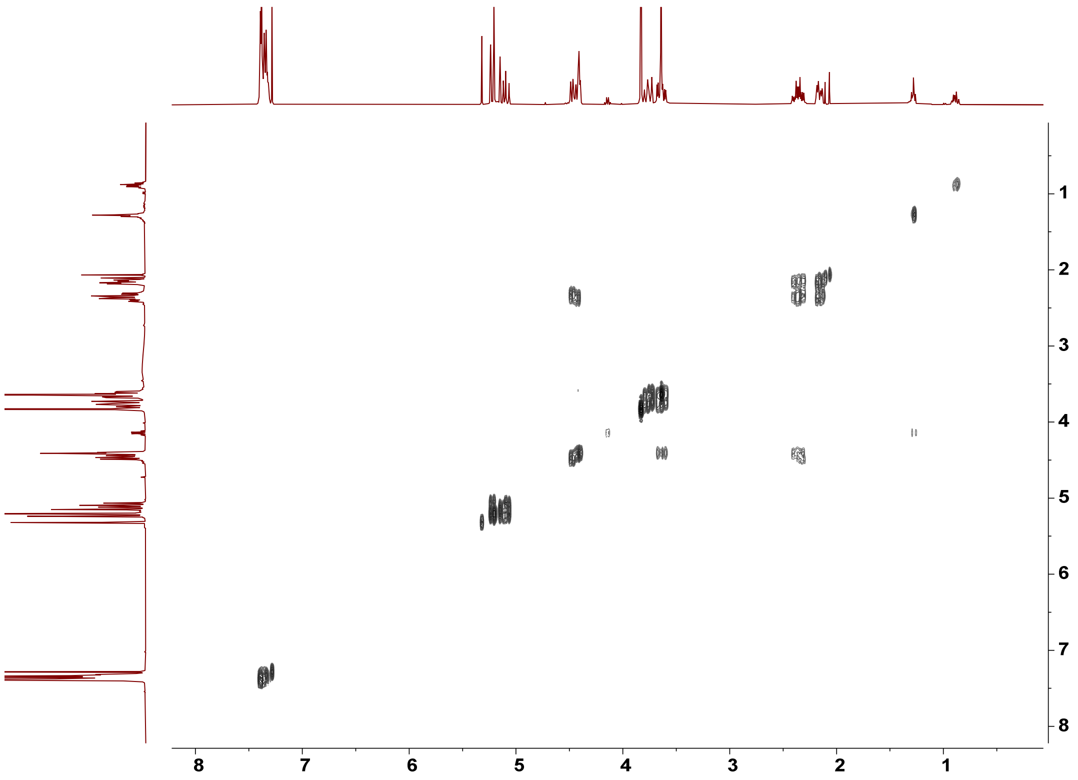
8
7
6
5
4
3
2
1

d)

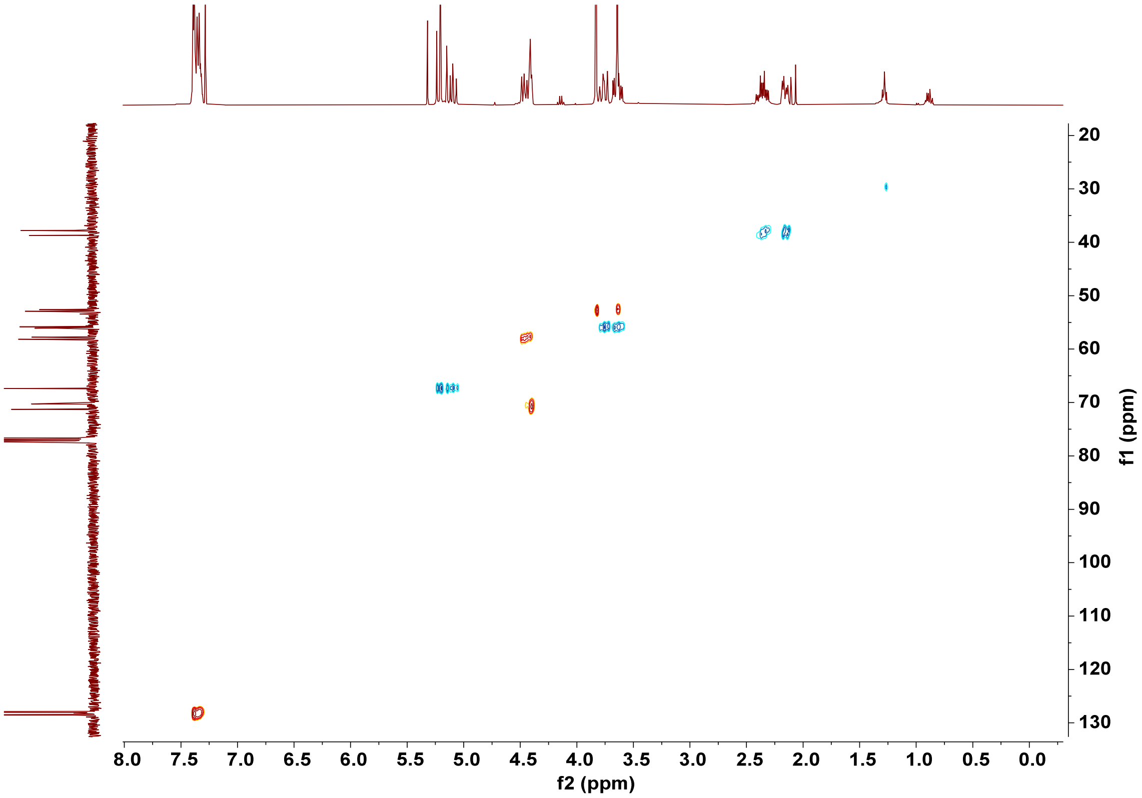

Figure S71. NMR spectra ($CDCl_3$) of (2*S*,4*S*)-**15k**, chemical synthesis: a) ^1H, b) ^{13}C, c) COSY, d) HSQC.

a)

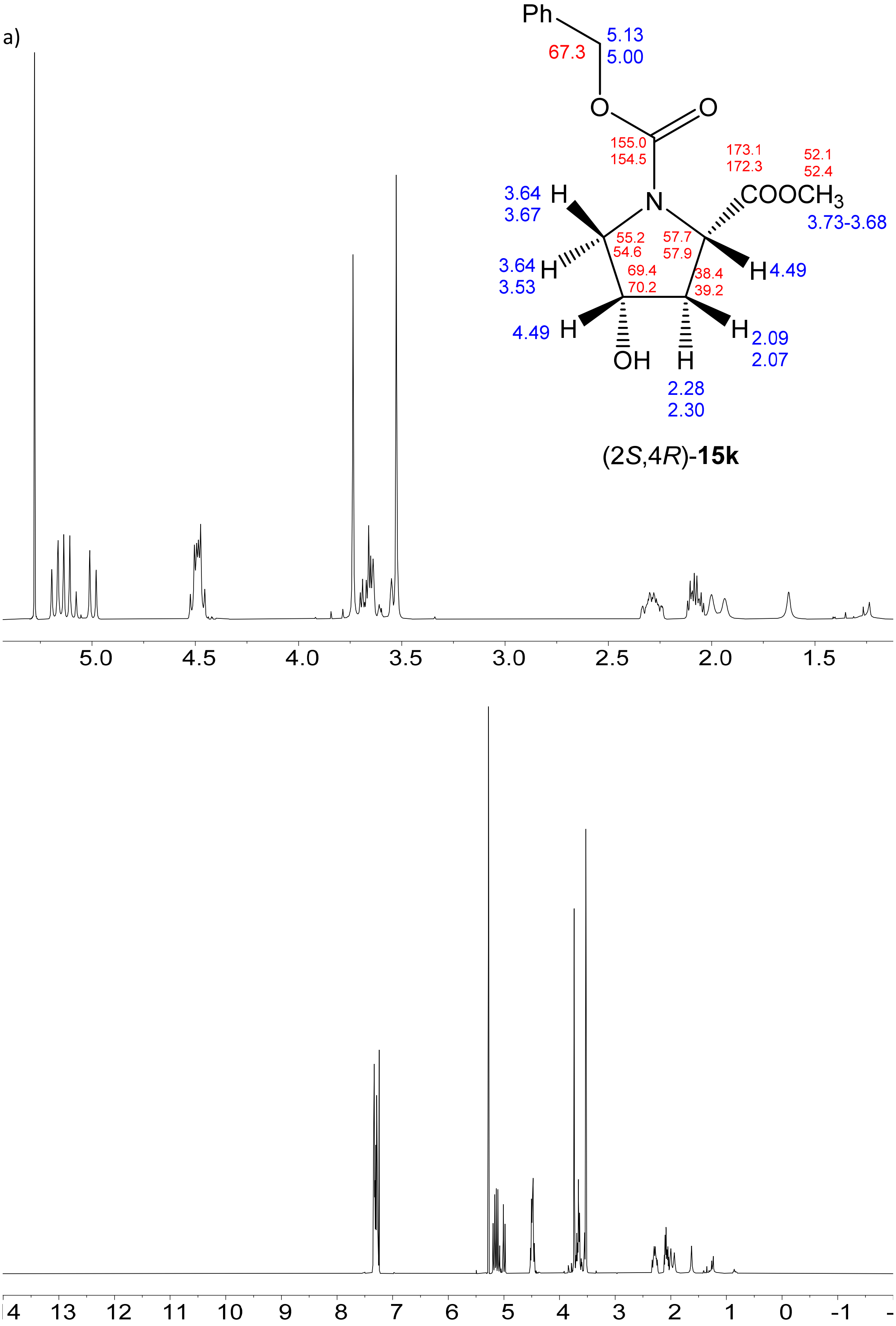

Ph
67.3
5.13
5.00
O
O
155.0
154.5
173.1
172.3
52.1
52.4
N
COOCH3
3.73-3.68
3.64
3.67
H
3.64
3.53
H
55.2
54.6
57.7
57.9
69.4
70.2
38.4
39.2
H 4.49
4.49 H
OH
H
H 2.09
2.07
2.28
2.30
(2S,4R)-15k
5.0
4.5
4.0
3.5
3.0
2.5
2.0
1.5
13
12
11
10
9
8
7
6
5
4
3
2
1
0
-1

b)

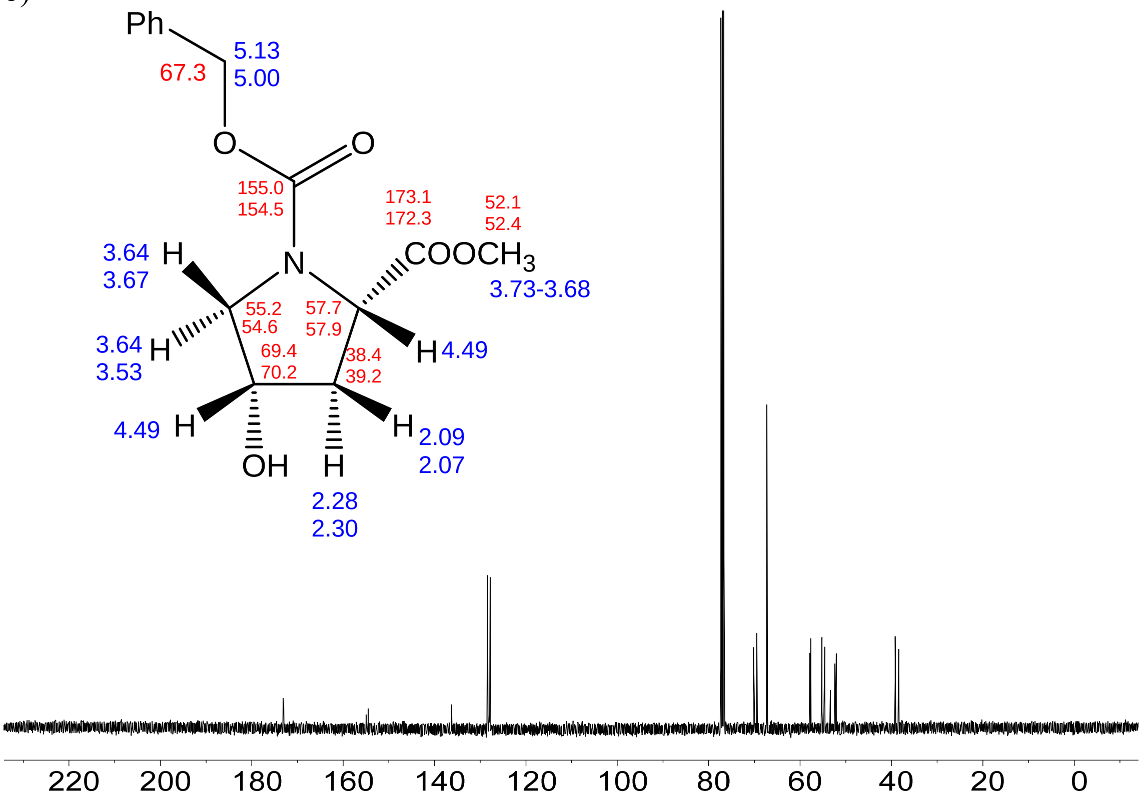
Ph
5.13
5.00
67.3
O
O
155.0
154.5
173.1
172.3
52.1
52.4
3.64 H
3.67
N
COOCH3
3.73-3.68
55.2
54.6
57.7
57.9
3.64 H
3.53
69.4
70.2
38.4
39.2
H 4.49
4.49 H
H 2.09
2.07
OH
H
2.28
2.30
220
200
180
160
140
120
100
80
60
40
20
0

c)

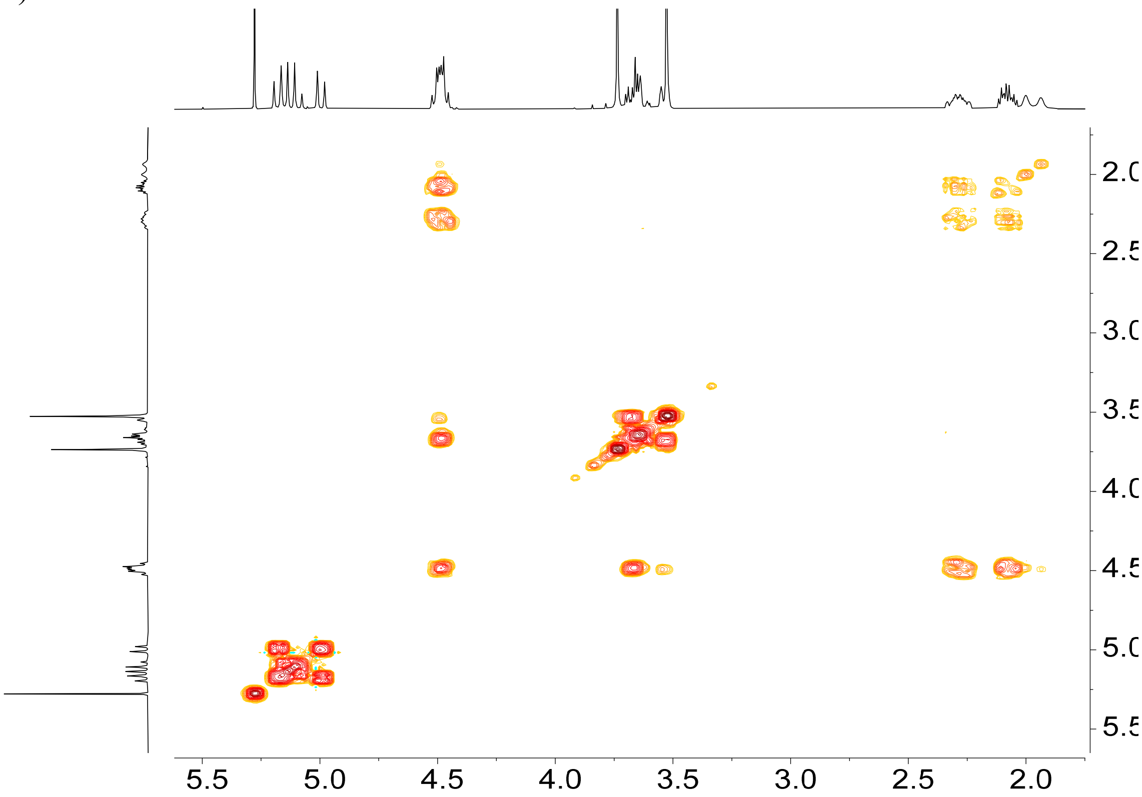
5.5
5.0
4.5
4.0
3.5
3.0
2.5
2.0

d)

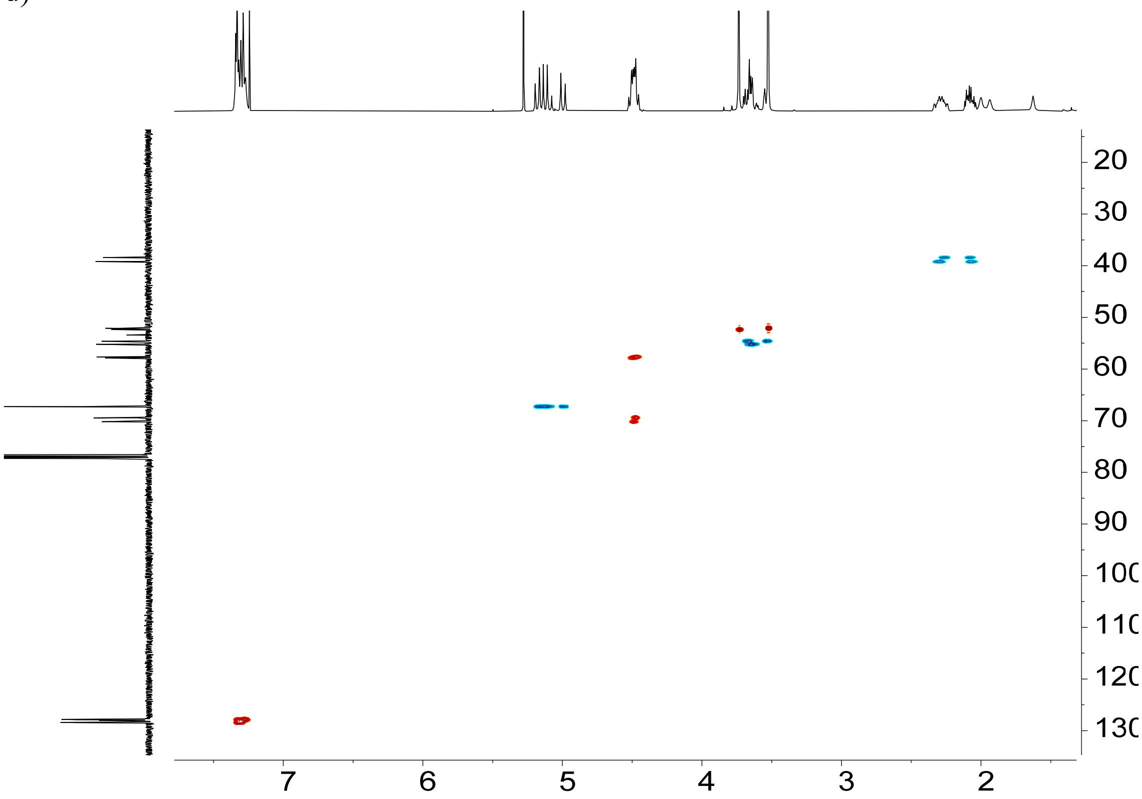

e)

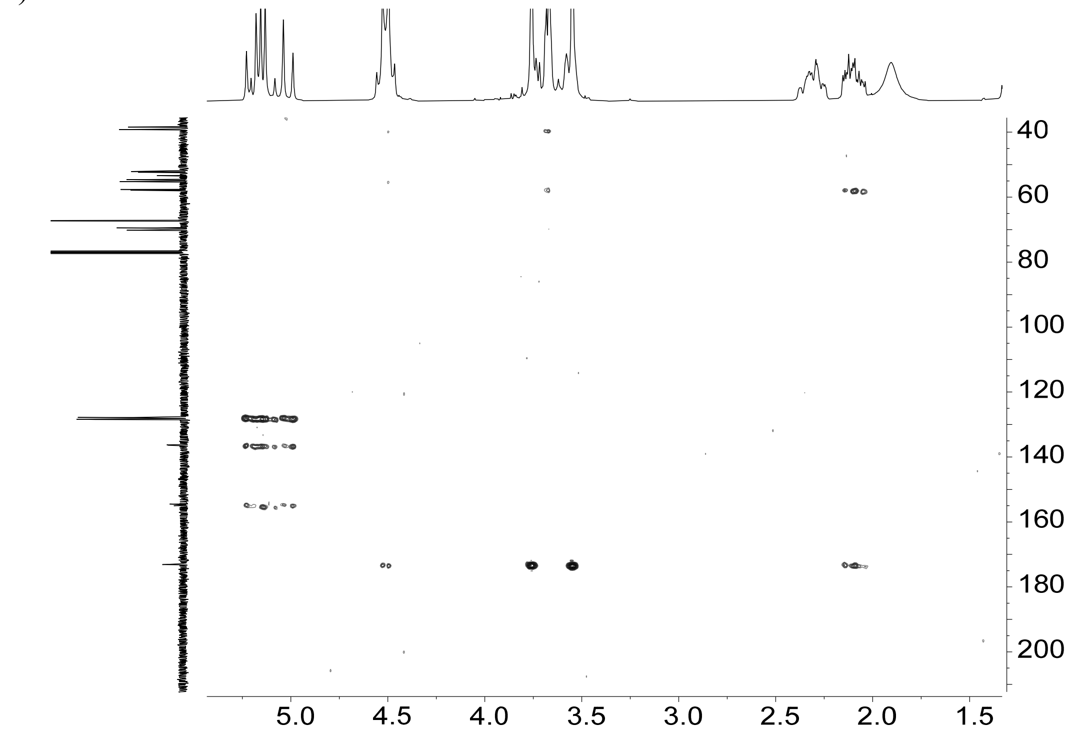

Figure S72. NMR spectra ($CDCl_3$) of (2*S*,4*R*)-**15k**, obtained via HBPA/BCAT/AspAT biocatalytic approach: a) ^{1}H, b) ^{13}C, c) COSY, d) HSQC, e) HMBC.

a)

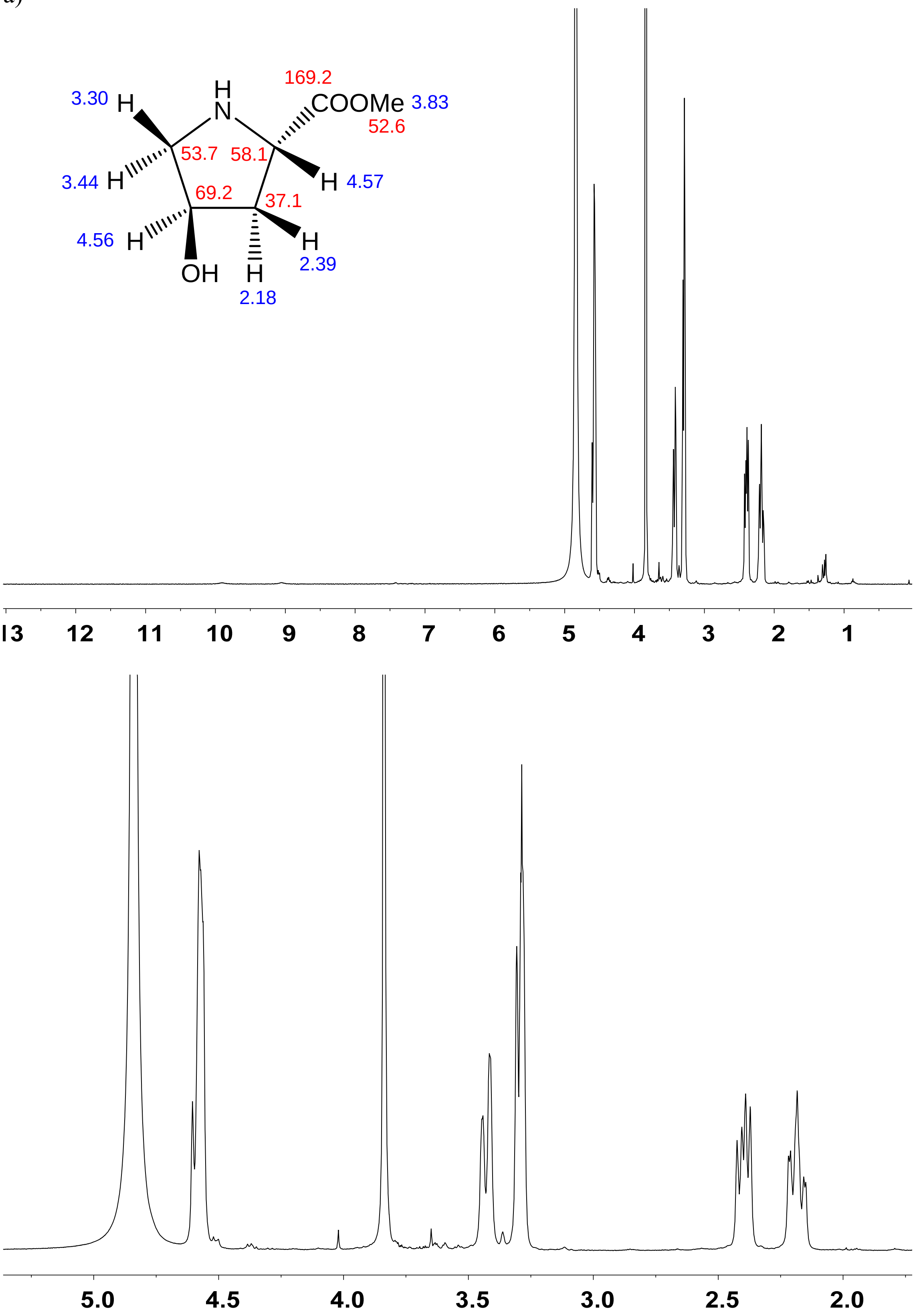

3.30 H
H
N
169.2
COOMe 3.83
52.6
53.7
58.1
3.44 H
H 4.57
69.2
37.1
4.56 H
H
2.39
OH
H
2.18
13 12 11 10 9 8 7 6 5 4 3 2 1
5.0 4.5 4.0 3.5 3.0 2.5 2.0

b)

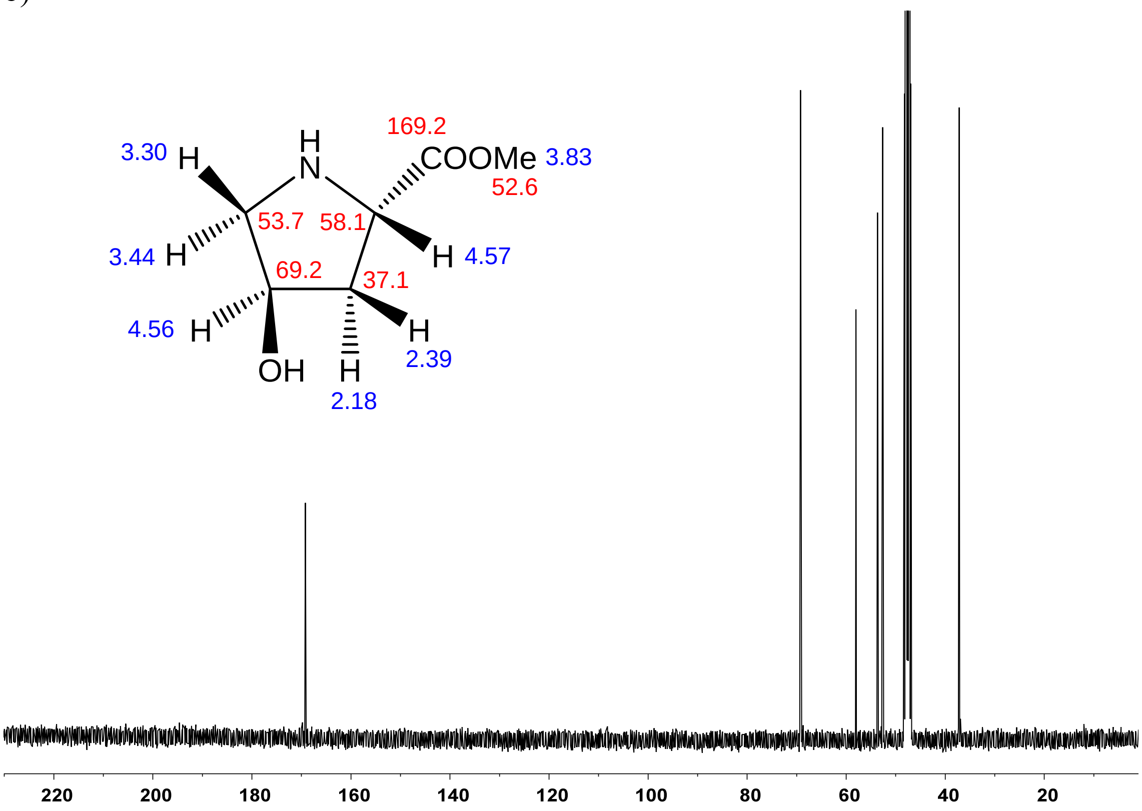

3.30
H
H
N
169.2
COOMe
3.83
52.6
3.44
H
53.7
58.1
H
4.57
69.2
37.1
4.56
H
H
OH
H
2.39
2.18
220
200
180
160
140
120
100
80
60
40
20

c)

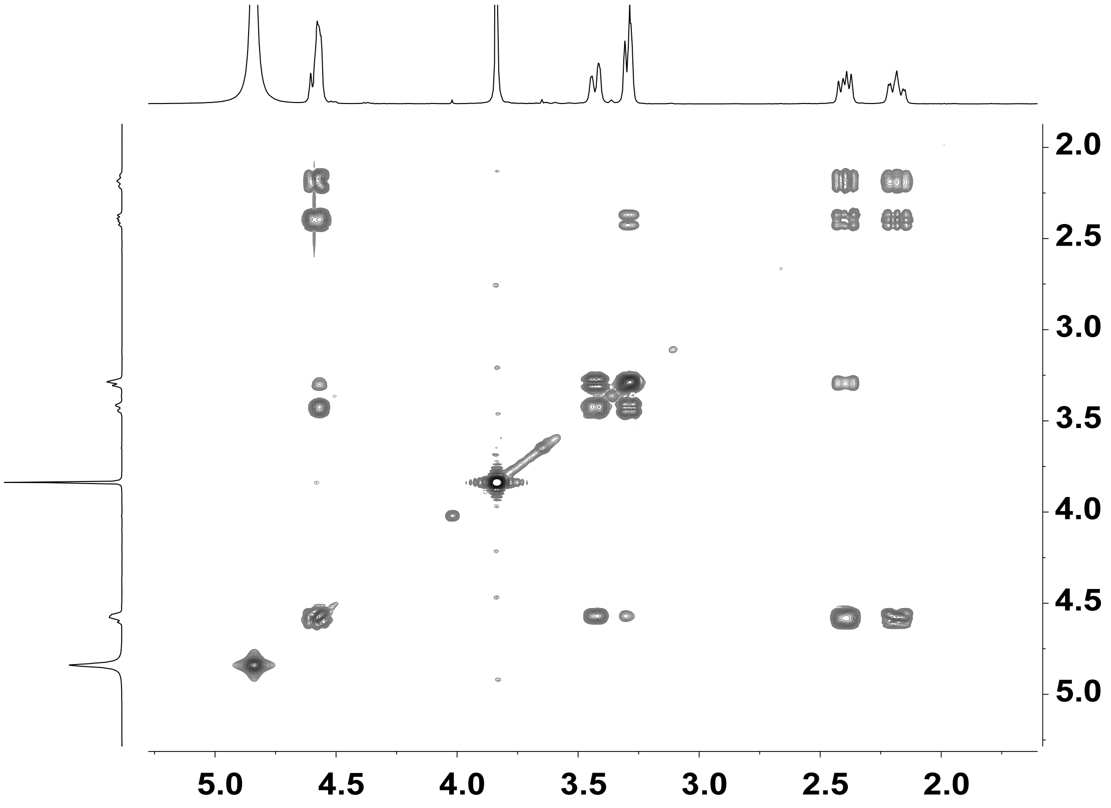

2.0
2.5
3.0
3.5
4.0
4.5
5.0
5.0
4.5
4.0
3.5
3.0
2.5
2.0

d)

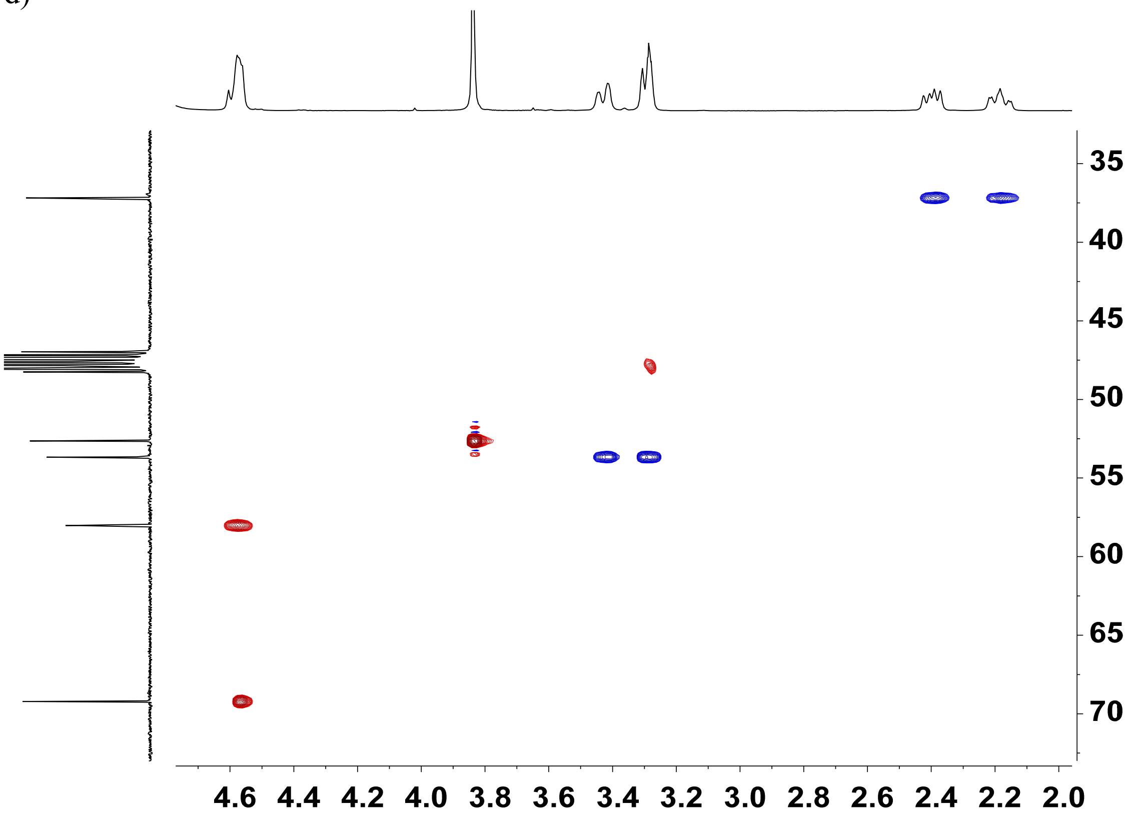

Figure S73. NMR spectra ($CDCl_3$) of **23k** (i.e. Cbz-removal of **15k**): a) ^{1}H, b) ^{13}C, c) COSY, and d) HSQC.

Chromatograms of the HPLC analysis on chiral stationary phases

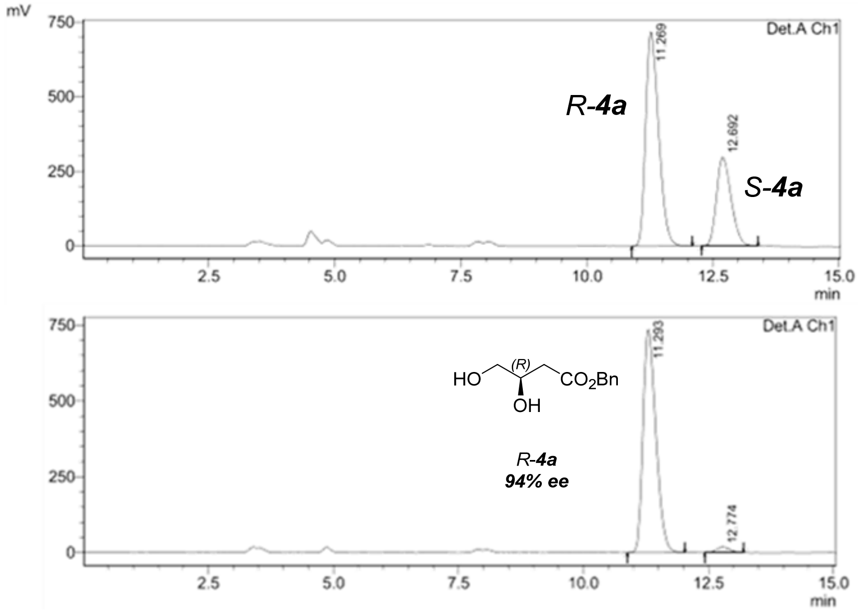

Figure S74: Chiral HPLC analysis chromatogram of *R*-**4a** synthetized with HBPA *wild-type* (down) and *rac*-**4a** synthetized with MBP-YFAU(Ni^{2+}) *wild-type* (see section Chiral HPLC analysis of 4-hydroxyesters). Conditions: CHIRALPACK® ID 46 x 250 mm column, 5 µm, flow rate 1 mL min^{-1} at 25 °C and UV detection (254 nm). Isocratic elution hexane:^{i}PrOH 80:20 (v/v).

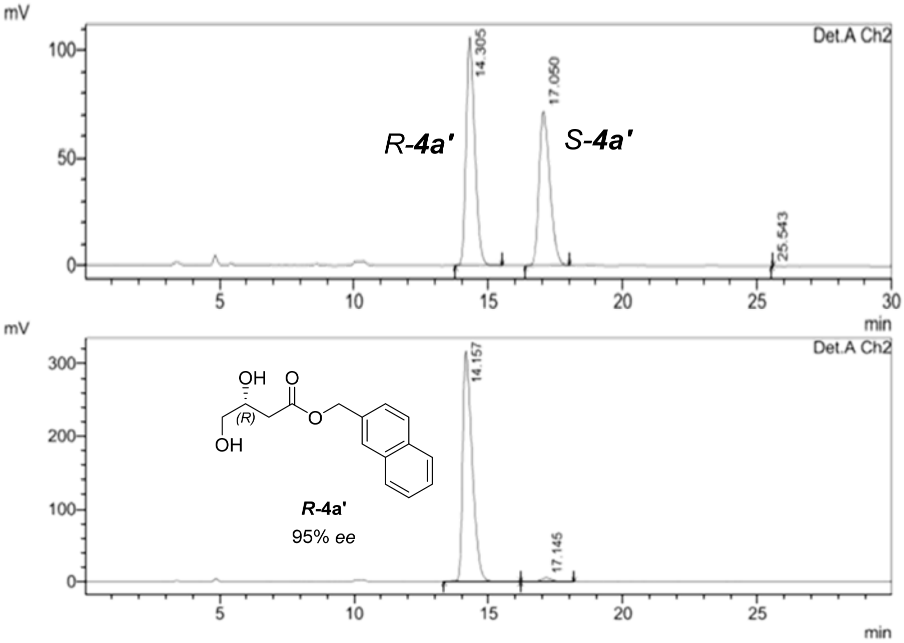

Figure S75: Chiral HPLC analysis chromatogram of *R*-**4a'** synthetized with HBPA *wild-type* (down) and *rac*-**4a** synthetized with MBP-YFAU(Ni^{2+}) *wild-type* type (see section

Chiral HPLC analysis of 4-hydroxyesters). Conditions: CHIRALPACK® ID 46 x 250 mm column, 5 µm, flow rate 1 mL min^{-1} at 25 °C and UV detection (254 nm). Isocratic elution hexane:^{i}PrOH 80:20 (v/v).

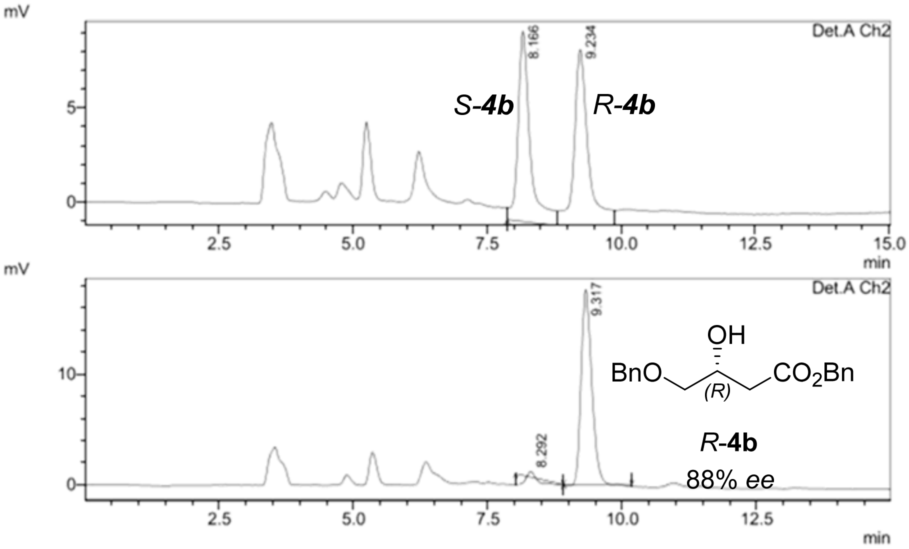

Figure S76: Chiral HPLC analysis chromatogram of *R*-**4b** synthetized with HBPA *wild-type* (down) and *rac*-**4b** synthetized with MBP-YFAU(Ni^{2+}) *wild-type* type (see section Chiral HPLC analysis of 4-hydroxyesters). Conditions: CHIRALPACK® IB 46 x 250 mm column, 5 µm, flow rate 1 mL min^{-1} at 25 °C and UV detection (254 nm). Isocratic elution hexane:^{i}PrOH 80:20 (v/v).

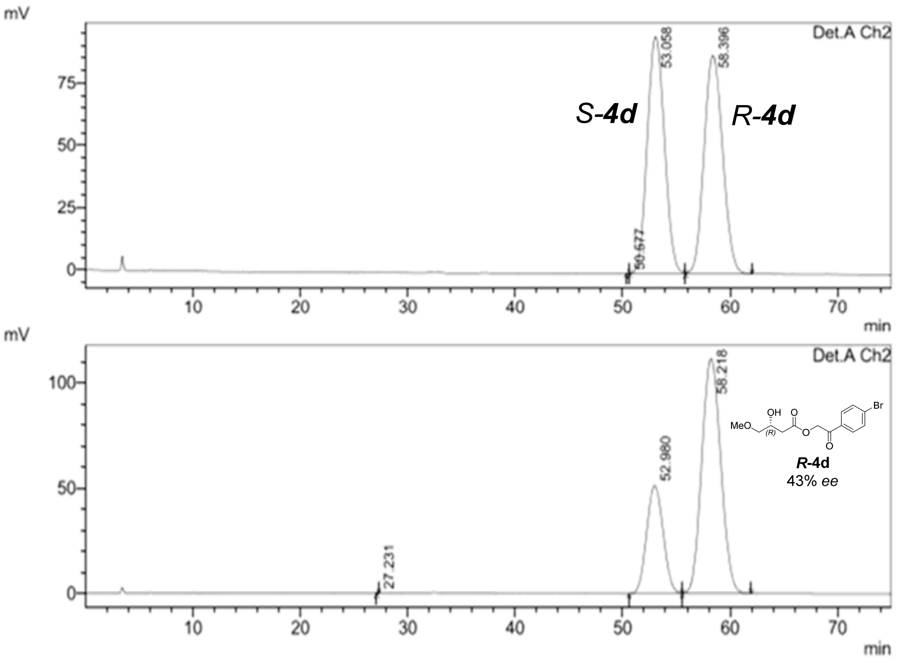

Figure S77: Chiral HPLC analysis chromatogram of *R*-**4d** synthetized with HBPA *wild-type* (down) and *rac*-**4d** synthetized with MBP-YFAU(Ni^{2+}) *wild-type* type (see section

Chiral HPLC analysis of 4-hydroxyesters). Conditions: CHIRALPACK® IC 46 x 250 mm column, 5 µm, flow rate 1 mL min^{-1} at 25 °C and UV detection (254 nm). Isocratic elution hexane:^{i}PrOH 80:20 (v/v).

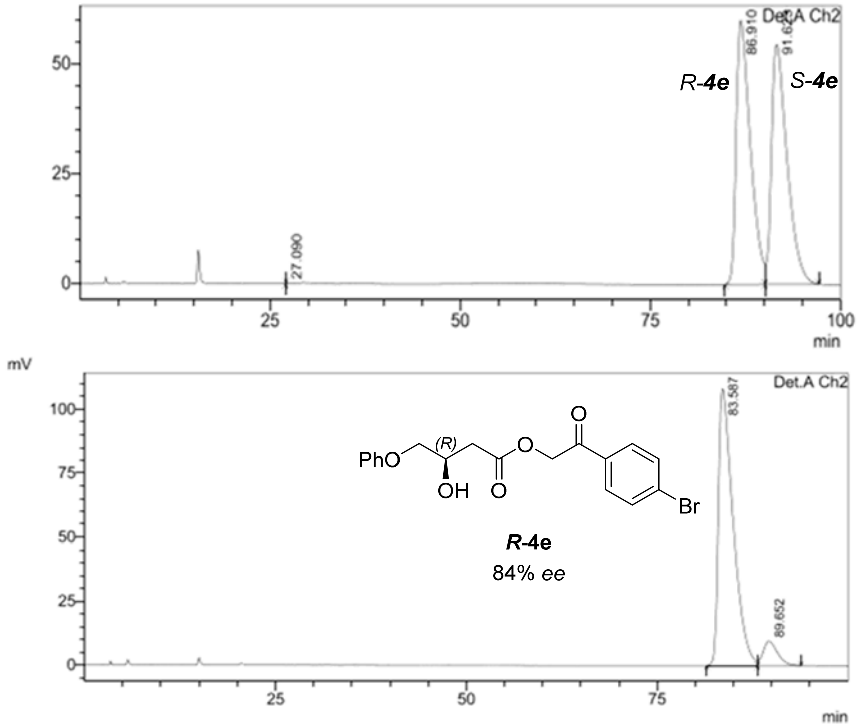

Figure S78: Chiral HPLC analysis chromatogram of *R*-**4e** synthetized with HBPA *wild-type* (down) and *rac*-**4e** synthetized with MBP-YFAU(Ni^{2+}) *wild-type* type (see section Chiral HPLC analysis of 4-hydroxyesters). Conditions: CHIRALPACK® IB 46 x 250 mm column, 5 µm, flow rate 1 mL min^{-1} at 25 °C and UV detection (254 nm). Isocratic elution hexane:^{i}PrOH 95:5 (v/v).

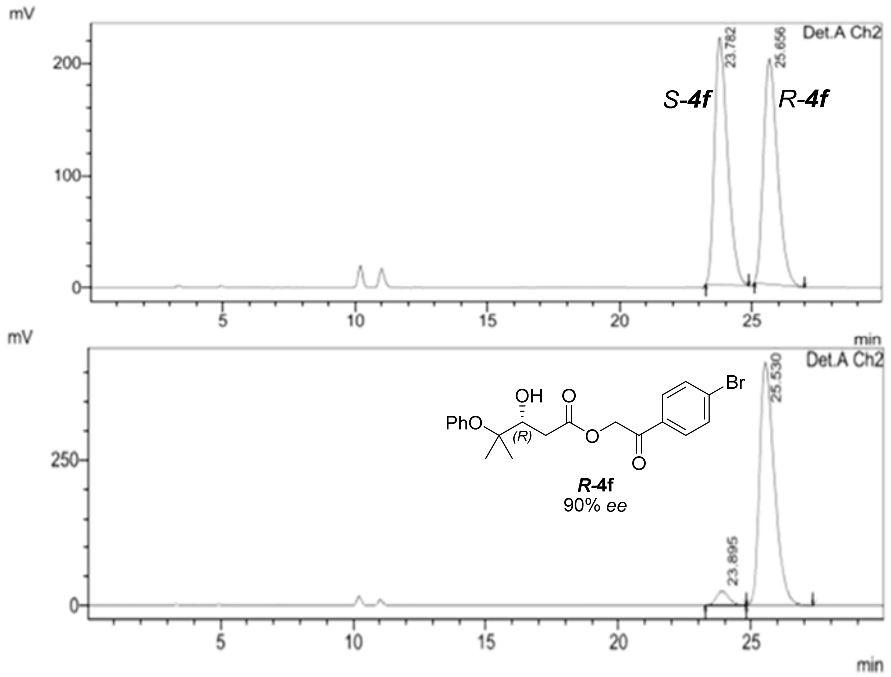

Figure S79: Chiral HPLC analysis chromatogram of *R*-**4f** synthetized with HBPA *wild-type* (down) and *rac*-**4f** synthetized with MBP-YFAU(Ni^{2+}) *wild-type* type (see section Chiral HPLC analysis of 4-hydroxyesters). Conditions: CHIRALPACK® IB 46 x 250 mm column, 5 µm, flow rate 1 mL min^{-1} at 25 °C and UV detection (254 nm). Isocratic elution hexane:^{i}PrOH 90:10 (v/v).

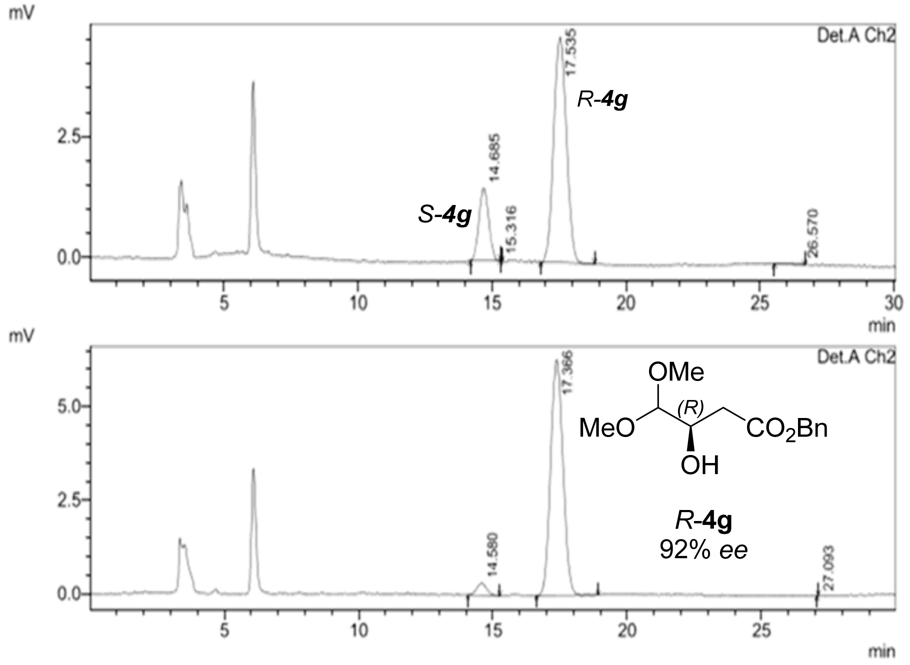

Figure S80: Chiral HPLC analysis chromatogram of *R*-**4g** synthetized with HBPA *wild-type* (down) and *rac*-**4g** synthetized with MBP-YFAU(Ni^{2+}) *wild-type* type (see section Chiral HPLC analysis of 4-hydroxyesters). Conditions: CHIRALPACK® IC 46 x 250

mm column, 5 μm, flow rate 1 mL min^{-1} at 25 °C and UV detection (254 nm). Isocratic elution hexane:^{i}PrOH 80:20 (v/v).

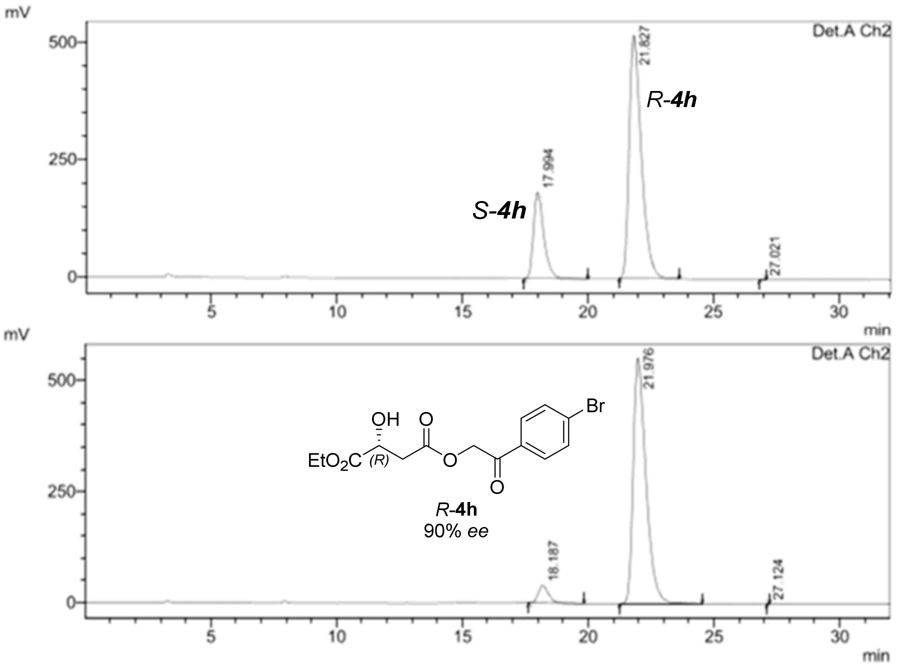

Figure S81: Chiral HPLC analysis chromatogram of *R*-**4h** synthetized with HBPA *wild-type* (down) and *rac*-**4h** synthetized with MBP-YFAU(Ni^{2+}) *wild-type* type (see section Chiral HPLC analysis of 4-hydroxyesters). Conditions: CHIRALPACK® IB 46 x 250 mm column, 5 μm, flow rate 1 mL min^{-1} at 25 °C and UV detection (254 nm). Isocratic elution hexane:^{i}PrOH 80:20 (v/v).

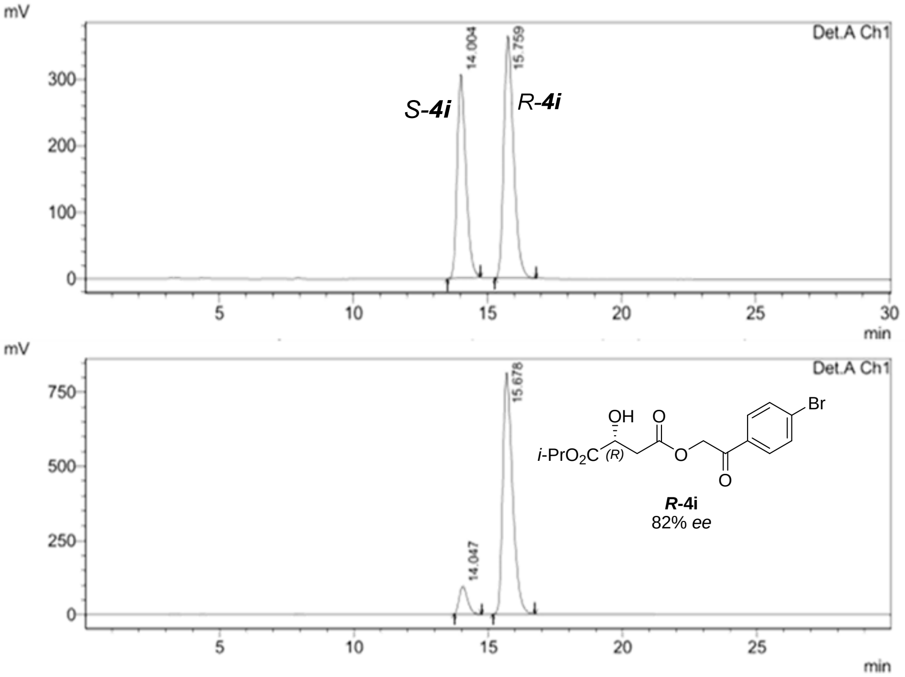

Figure S82: Chiral HPLC analysis chromatogram of *R*-**4i** synthetized with HBPA *wild-type* (down) and *rac*-**4i** synthetized with MBP-YFAU(Ni^{2+}) *wild-type* type (see section

Chiral HPLC analysis of 4-hydroxyesters). Conditions: CHIRALPACK® IB 46 x 250 mm column, 5 μm, flow rate 1 mL min^{-1} at 25 °C and UV detection (254 nm). Isocratic elution hexane:^{i}PrOH 80:20 (v/v).

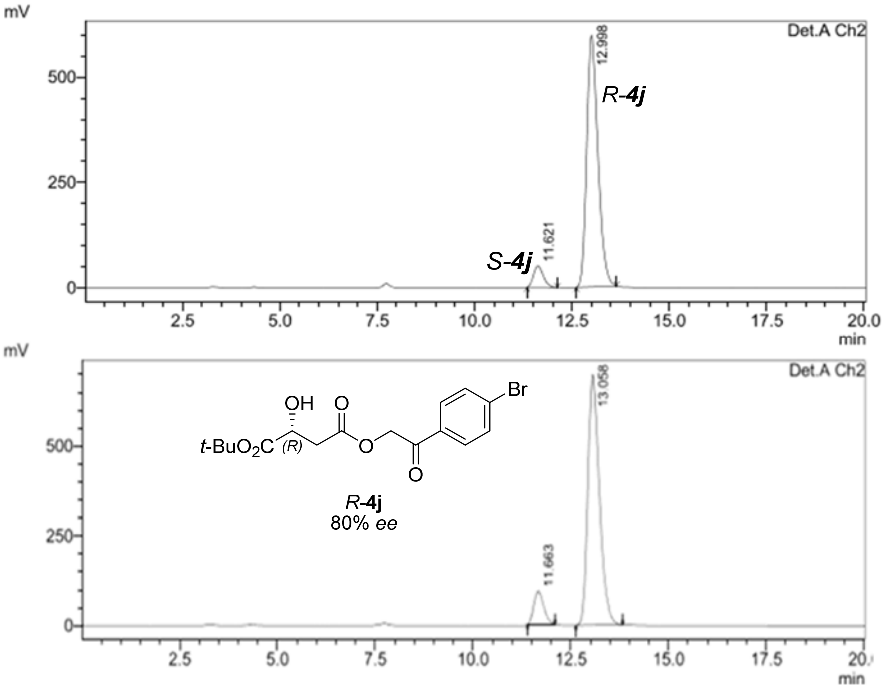

Figure S83: Chiral HPLC analysis chromatogram of ***R*-4j** synthetized with HBPA *wild-type* (down) and *rac*-**4j** synthetized with MBP-YFAU(Ni^{2+}) *wild-type* type (see section Chiral HPLC analysis of 4-hydroxyesters). Conditions: CHIRALPACK® IB 46 x 250 mm column, 5 μm, flow rate 1 mL min^{-1} at 25 °C and UV detection (254 nm). Isocratic elution hexane:^{i}PrOH 80:20 (v/v).

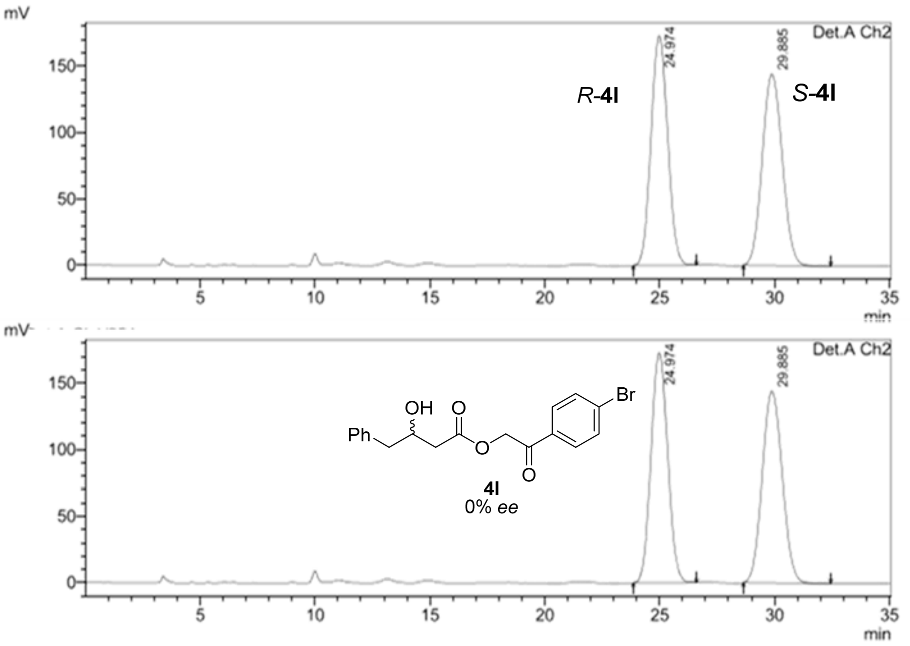

Figure S84: Chiral HPLC analysis chromatogram of *racemic* **4l** synthetized with HBPA *wild-type* (down) and *rac*-**4l** synthetized with MBP-YFAU(Ni^{2+}) *wild-type* type (see section Chiral HPLC analysis of 4-hydroxyesters). Conditions: CHIRALPACK® IC 46 x 250 mm column, 5 µm, flow rate 1 mL min^{-1} at 25 °C and UV detection (254 nm). Isocratic elution hexane:^{i}PrOH 80:20 (v/v).

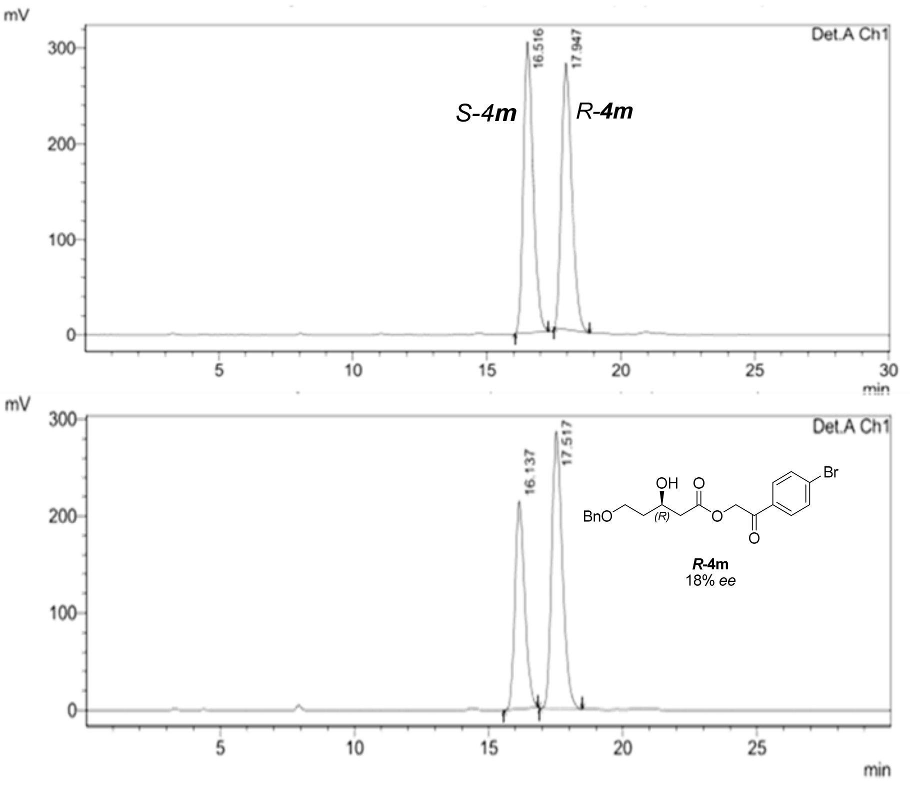

Figure S85: Chiral HPLC analysis chromatogram of *R*-**4m** synthetized with HBPA *wild-type* (down) and *rac*-**4m** synthetized with MBP-YFAU(Ni^{2+}) *wild-type* type (see section Chiral HPLC analysis of 4-hydroxyesters). Conditions: CHIRALPACK® IB 46 x 250 mm column, 5 μm, flow rate 1 mL min^{-1} at 25 °C and UV detection (254 nm). Isocratic elution hexane:^{i}PrOH 80:20 (v/v).

Computational Modeling

The crystal structure of HBPA has recently been solved (PDB 6DAO) together with the structure of its homolog *trans*-*o*-carboxybenzylidene pyruvate hydratase-aldolase (CBPA), the later crystalized alone (PDB 6DAN) and in complex with a substrate molecule (PDB 6DAQ).[14] The HBPA active site is located inside of a relatively deep and narrow cavity, where the ε-amino group of the essential Lys183 is at about 11 Å from the surface, with several aromatic residues flanking the entrance (H205, W224, F269 and F277) and predominantly polar residues (G64, T65, G67, Y155, N157 and N281) at the deeper end, close to the catalytic lysine (**Figure S86A, B**).

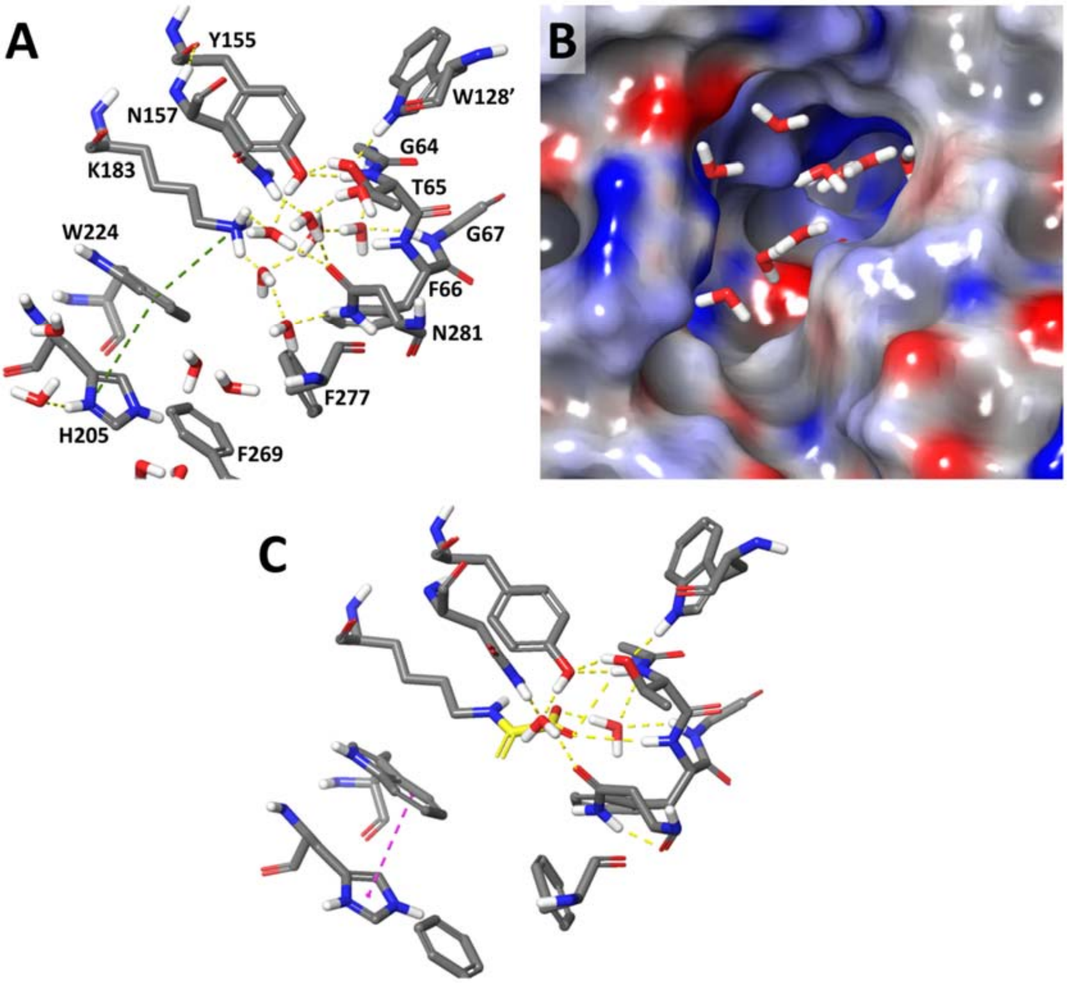

Figure S86. (A) Crystal structure of the active site of HBPA (chain A, PDB 6DAO[14]), showing the essential Lys residue (K183) and the residues that conform the cavity, and (B) its electrostatic potential mapped surface. (C) Model of the enamine pyruvate HBPA covalent complex. The pyruvate enamine is highlighted with yellow C-atoms. Two water molecules are also shown: one of them H-bound to the pyruvate carboxylate and the second one to residues Y155 and N157.

The structure of the CBPA complex (PDB 6DAQ) allows proposing that the covalently bound pyruvate enamine intermediate in HBPA adopts a similar configuration, with its carboxylate moiety oriented towards the pyruvoyl carboxylate binding motif (GXXGE, $G^{64}TFGE^{68}$ in HBPA), stabilized by H-bonds with the backbone amide NH groups of residues Thr65 and Phe66, and with a conserved water molecule which in turn is hydrogen bonded to the backbone amides of Gly64 and Gly67 (**Figure S86C**). The proposed enzymatic mechanism for HBPA (**Figure S87**) implies that Tyr155 could be the acid/base catalyst that protonates the carbonyl oxygen of the aldehyde electrophile, once the C-C

bond is formed.[14] Therefore, the incoming aldehyde should displace a water molecule that is H-bonded to Tyr155 and Asn157 in the HBPA crystal structure (Figure S85C), and adequately locate its carbonyl oxygen for protonation by Tyr155. Based on this hypothesis, structural models of the pre-reactive complexes of pyruvate-enamine bound HBPA, *wild-type* and H205A variant, with the different electrophiles assayed, approaching the enamine from its *re*- and *si*-faces, were built (Figures S87-S92). Similarly, models were built for the corresponding aldol adduct intermediates bound as imines to residue Lys183 (Figures S93-S98). According to the mechanism (Figure S87), these models have the Lys183 ε-nitrogen atom protonated and the phenol group of Tyr155 unprotonated.

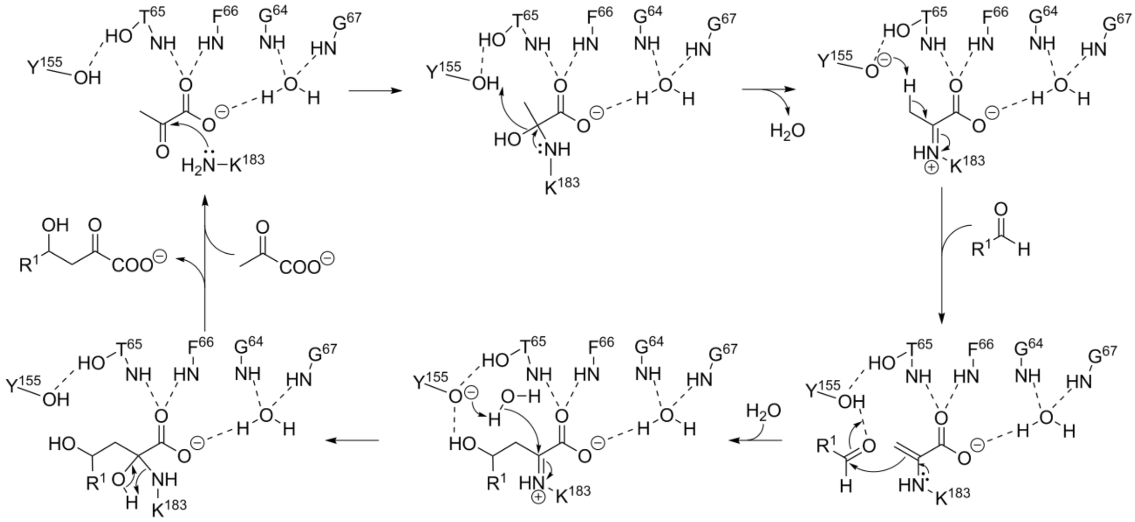

Figure S87. Proposed mechanism for HBPA.

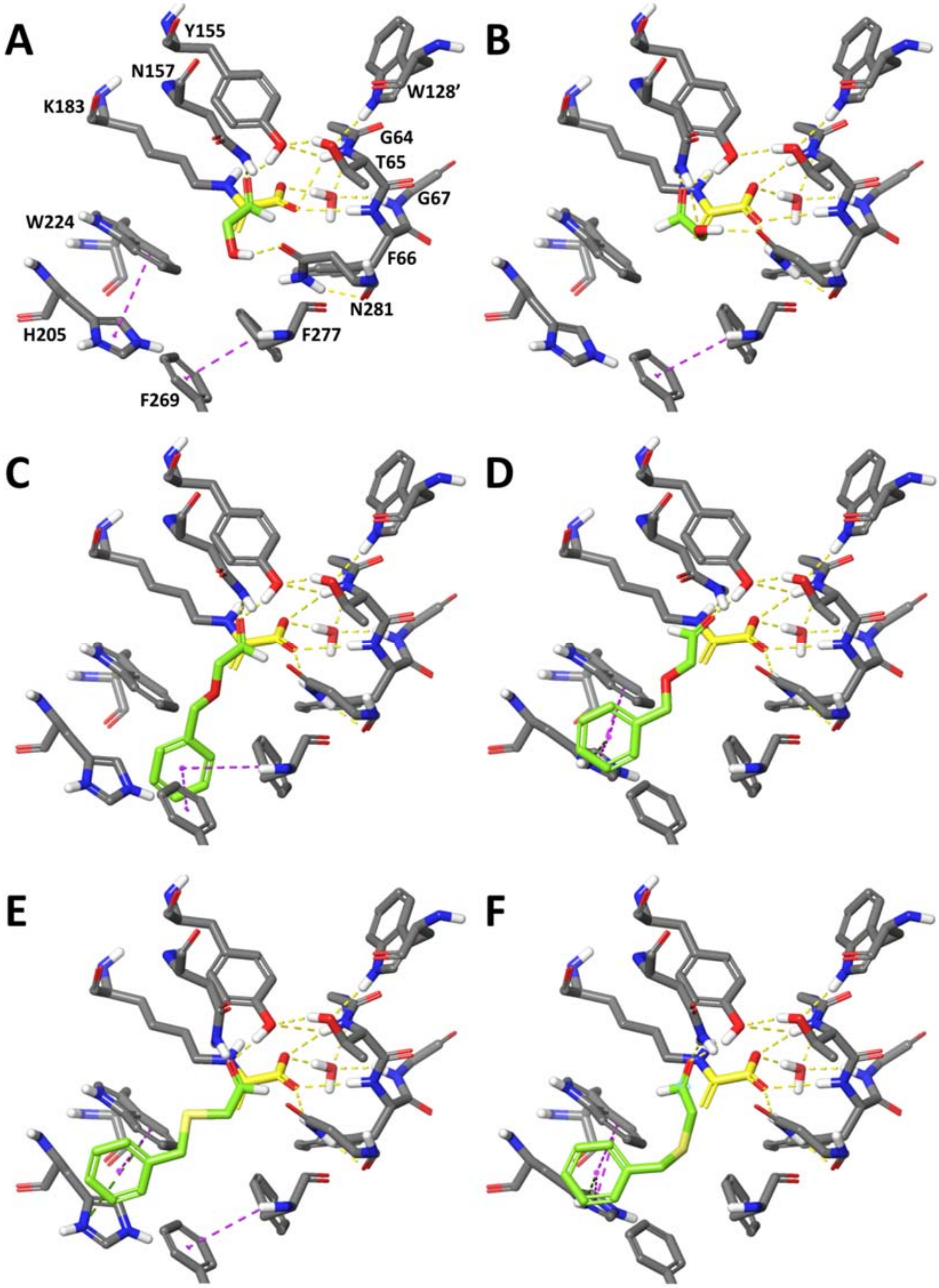

Figure S88. Models of the pre-reactive complexes of pyruvate-enamine bound *wild-type* HBPA with (**A**,**B**) **1a**, (**C**,**D**) **1b** and (**E**,**F**) **1c**. Electrophiles **1a-c** are shown approaching the enamine from their *re*- (**A**,**C**,**E**) or *si*-face (**B**,**D**,**F**). The pyruvate-enamine is shown with yellow C-atoms, the electrophile molecules with green C-atoms, and the interactions with dashed lines: H-bond in yellow, π-π stacking in magenta and π-cation in green.

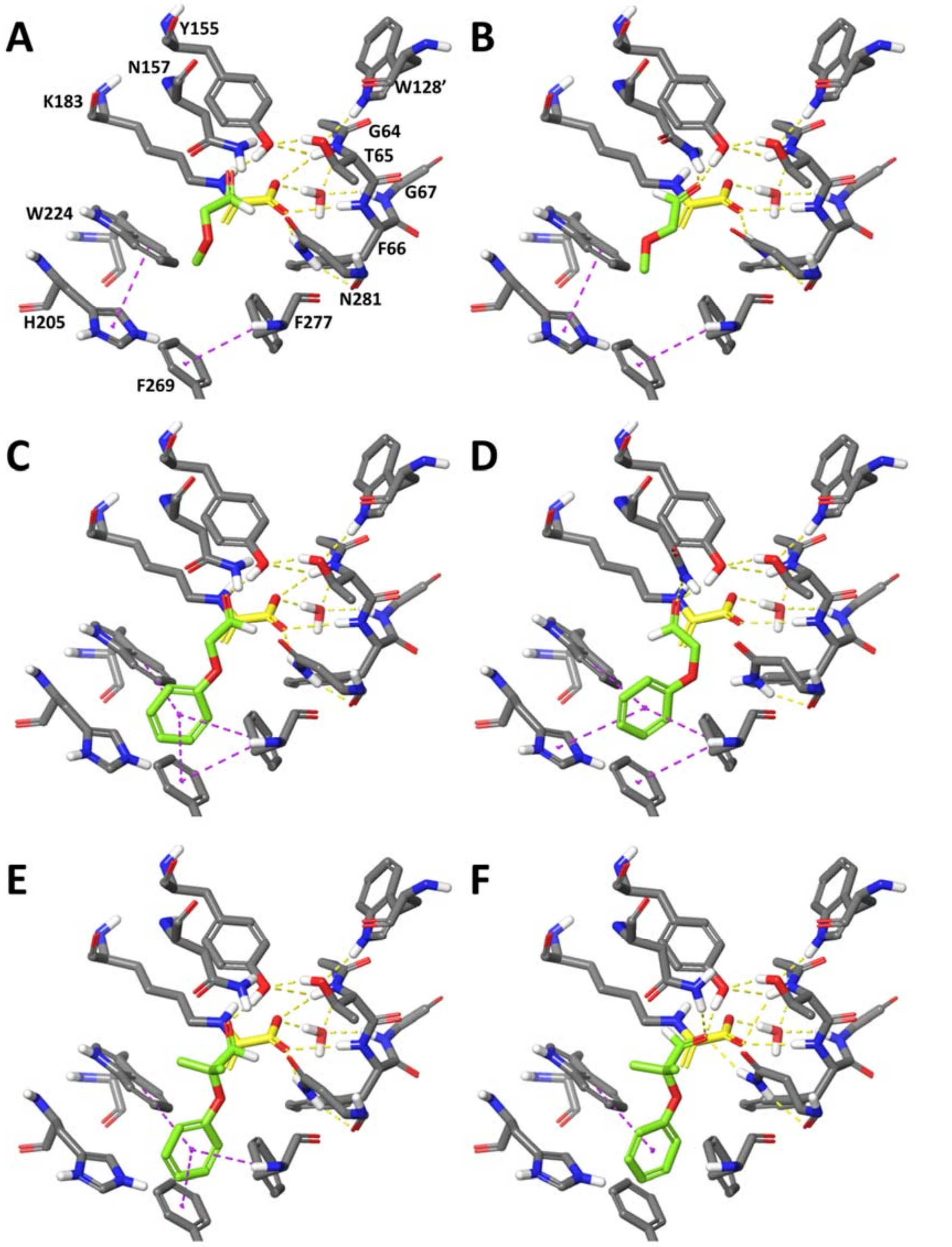

Figure S89. Models of the pre-reactive complexes of pyruvate-enamine bound *wild-type* HBPA with (**A**,**B**) **1d**, (**C**,**D**) **1e** and (**E**,**F**) **1f**. Electrophiles **1d-f** are shown approaching the enamine from their *re*- (**A**,**C**,**E**) or *si*-face (**B**,**D**,**F**). Representation details as in Figure S88.

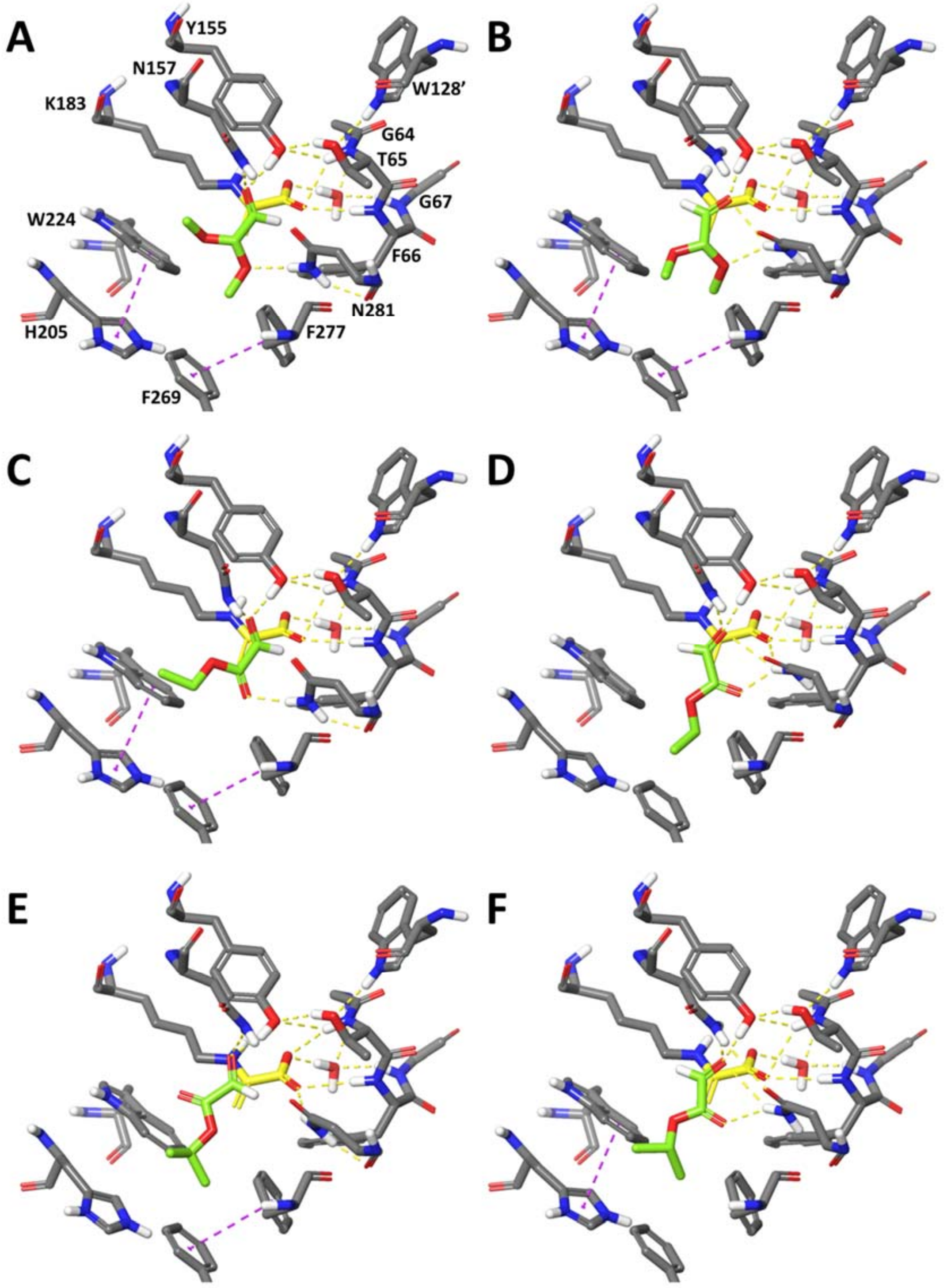

Figure S90. Models of the pre-reactive complexes of pyruvate-enamine bound *wild-type* HBPA with (**A**,**B**) **1g**, (**C**,**D**) **1h** and (**E**,**F**) **1i**. Electrophiles **1g-i** are shown approaching the enamine from their *re*- (**A**,**C**,**E**) or *si*-face (**B**,**D**,**F**). Representation details as in Figure S88.

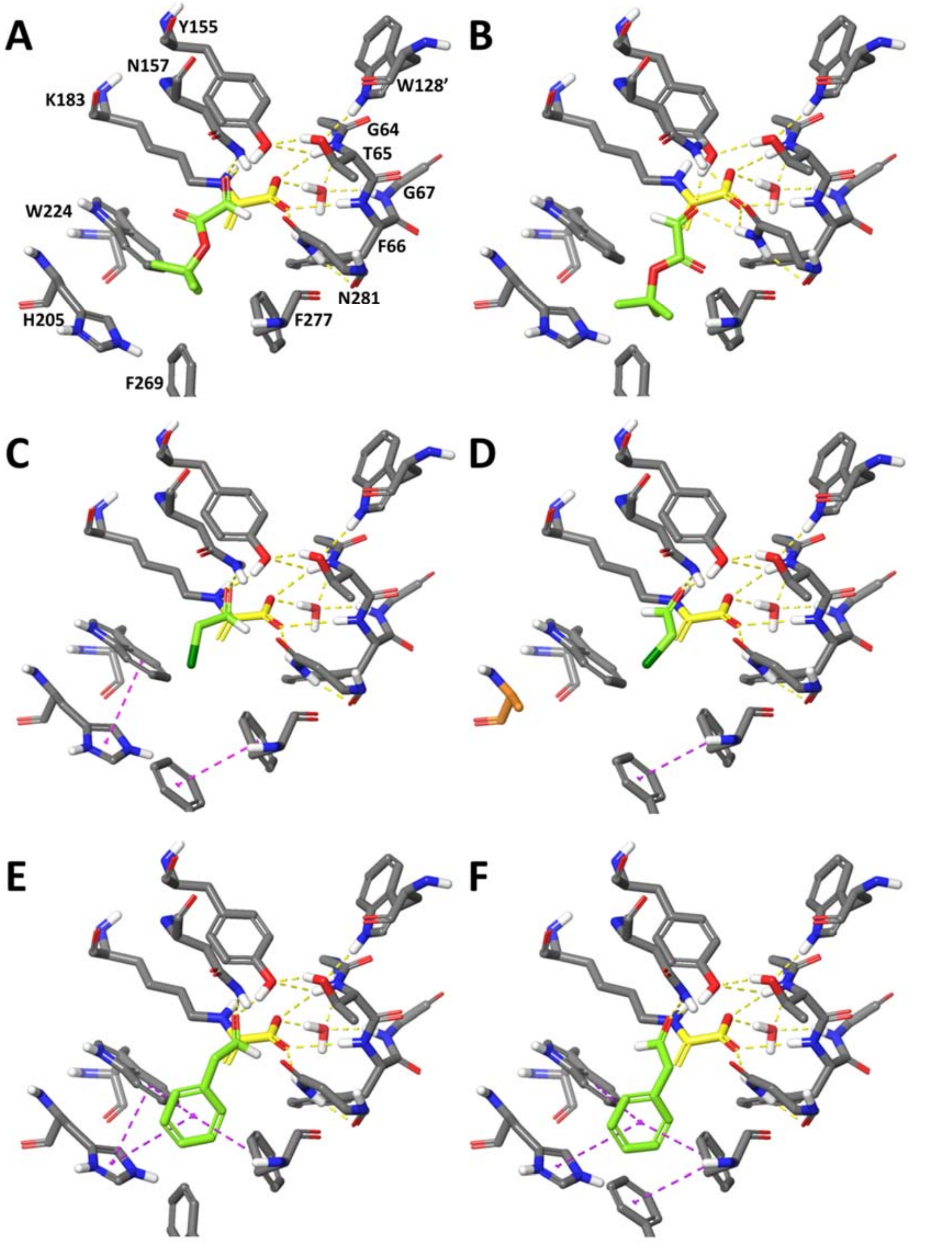

Figure S91. Models of the pre-reactive complexes of pyruvate-enamine bound *wild-type* HBPA with (**A**,**B**) **1j**, (**C**,**D**) **1k** and (**E**,**F**) **1l**. Electrophiles **1j-l** are shown approaching the enamine from their *re*- (**A**,**C**,**E**) or *si*-face (**B**,**D**,**F**). Representation details as in Figure S88.

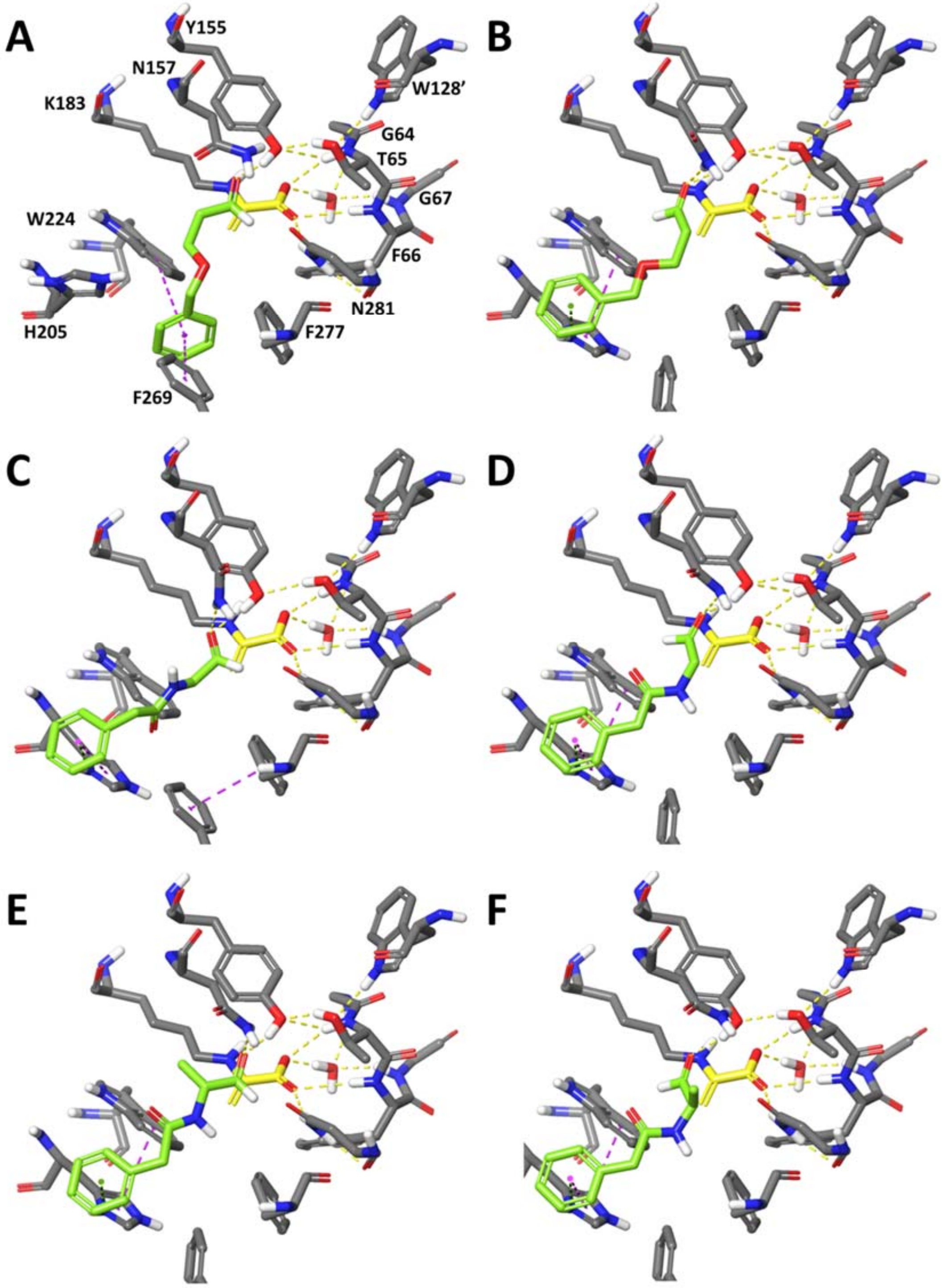

Figure S92. Models of the pre-reactive complexes of pyruvate-enamine bound *wild-type* HBPA with (**A**,**B**) **1m**, (**C**,**D**) **1n** and (**E**,**F**) **1o**. Electrophiles **1m-o** are shown approaching the enamine from their *re*- (**A**,**C**,**E**) or *si*-face (**B**,**D**,**F**). Representation details as in Figure S88.

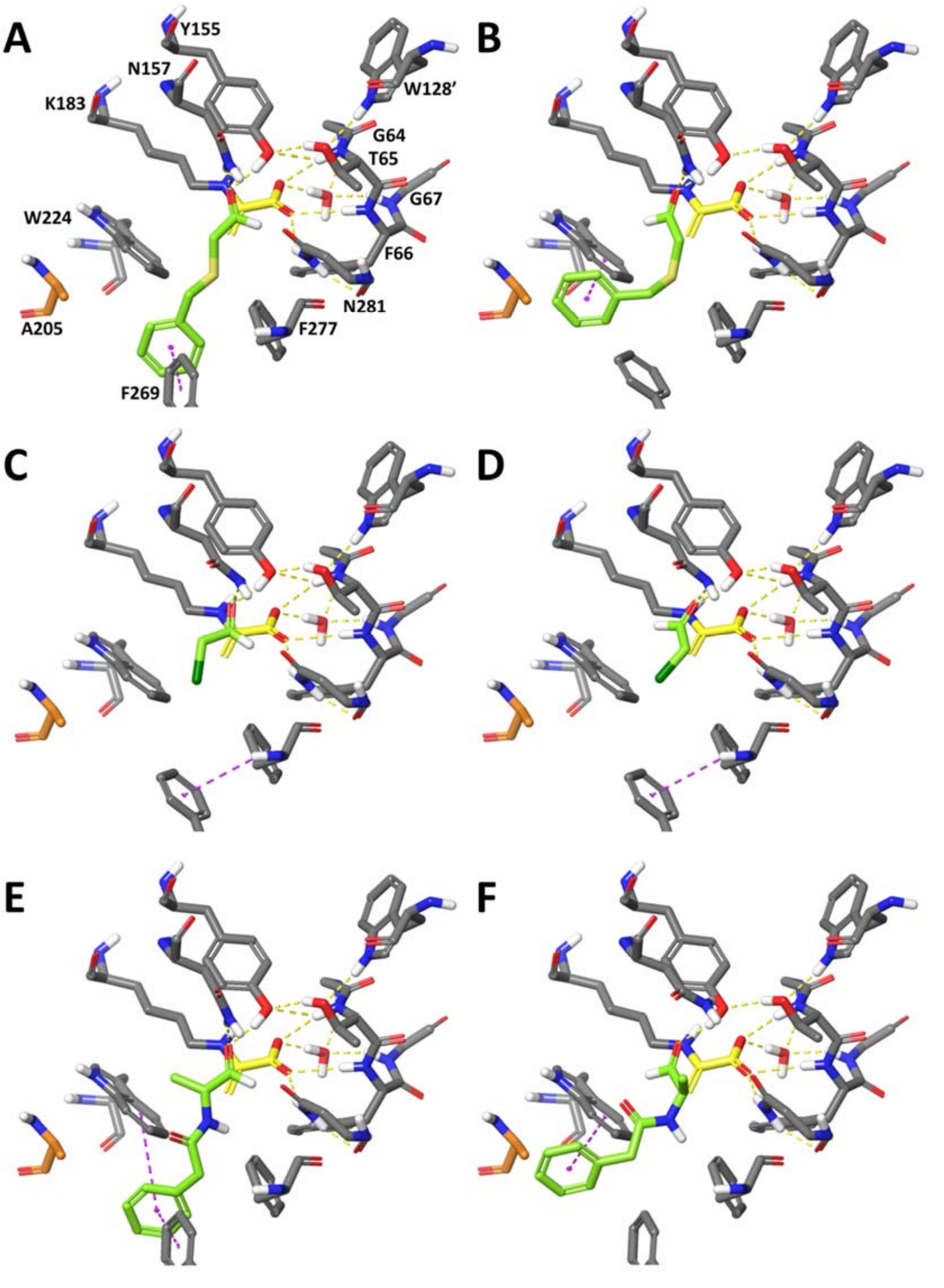

Figure S93. Models of the pre-reactive complexes of pyruvate-enamine bound HBPA H205A with (**A**,**B**) **1c**, (**C**,**D**) **1k** and (**E**,**F**) **1o**. Electrophiles **1m-o** are shown approaching the enamine from their *re*- (**A**,**C**,**E**) or *si*-face (**B**,**D**,**F**). The mutated residue A205 is highlighted in orange. The rest of representation details as in Figure S88.

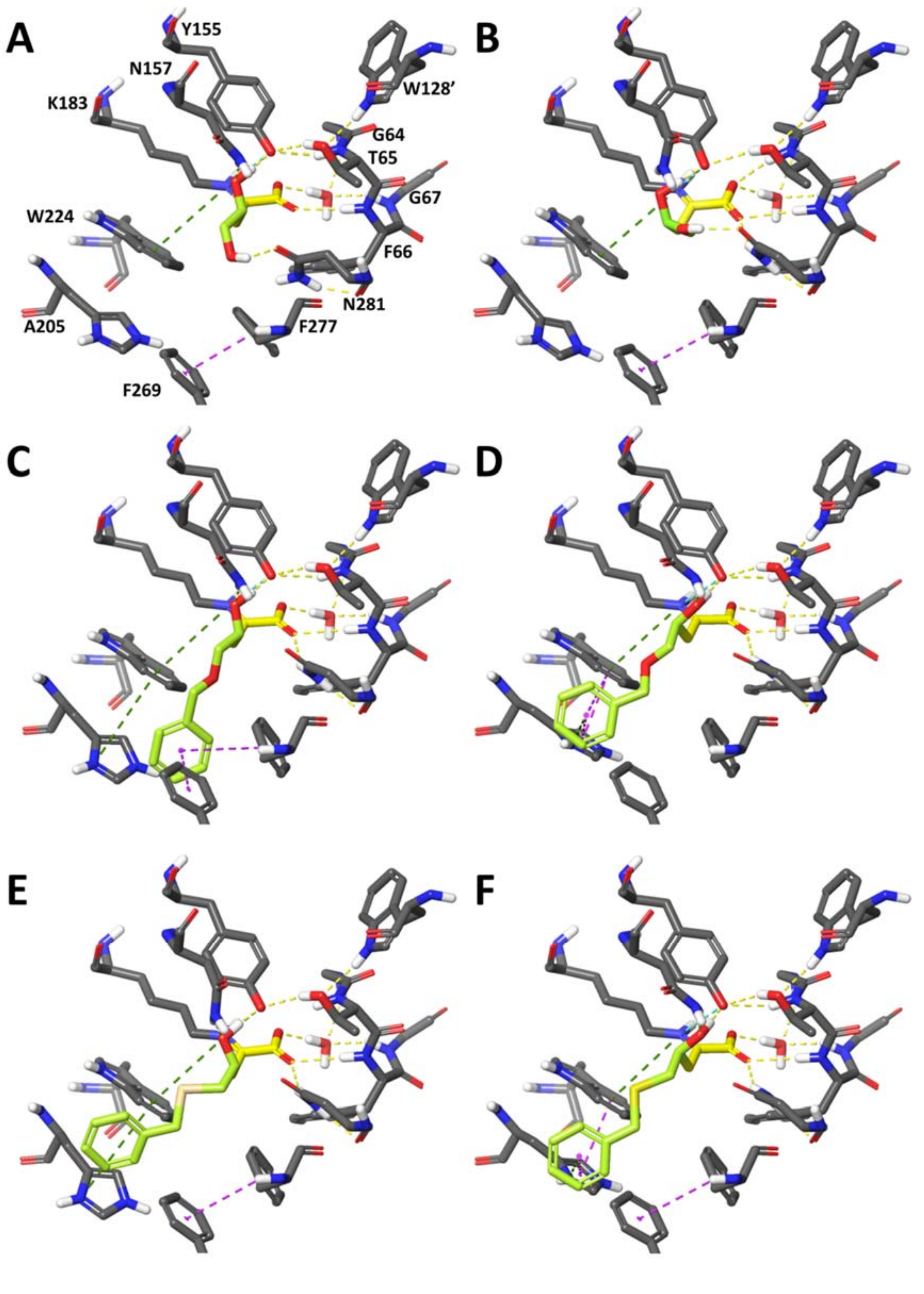

Figure S94. Models of HBPA complexes with the K183-bound aldol adduct imines derived from (**A**) (*R*)-**3a**, (**B**) (*S*)-**3a**, (**C**) (*R*)-**3b**, (**D**) (*S*)-**3b**, (**E**) (*R*)-**3c** and (**F**) (*S*)-**3c**. The pyruvate-derived moiety is shown with yellow C-atoms, the electrophile-derived moieties with green C-atoms, and the interactions with dashed lines: H-bond in yellow, salt bridges in cyan, π-π stacking in magenta and π-cation in green.

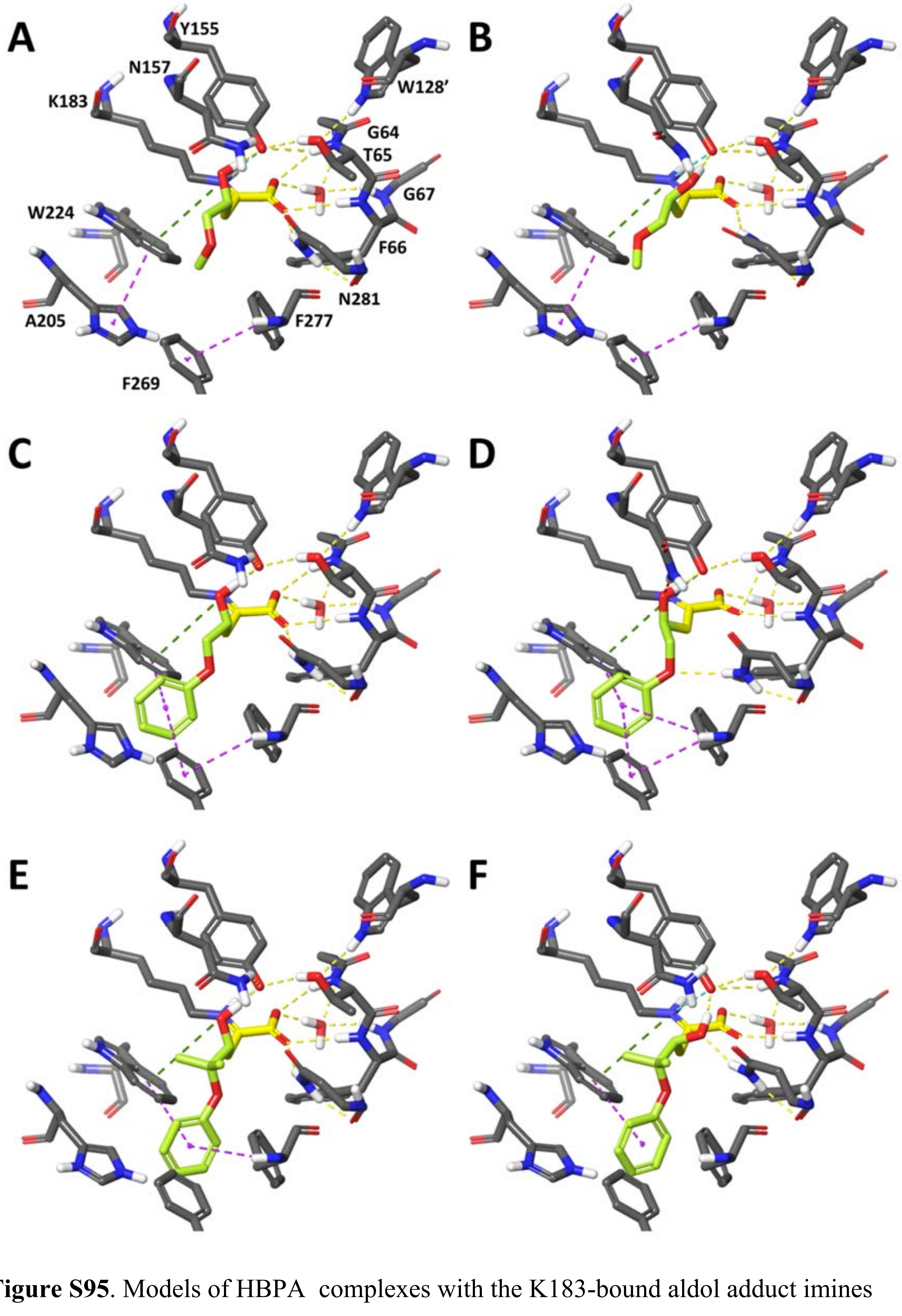

Figure S95. Models of HBPA complexes with the K183-bound aldol adduct imines derived from (**A**) (*R*)-**3d**, (**B**) (*S*)-**3d**, (**C**) (*R*)-**3e**, (**D**) (*S*)-**3e**, (**E**) (*R*)-**3f** and (**F**) (*S*)-**3f**. Representation details as in Figure S94.

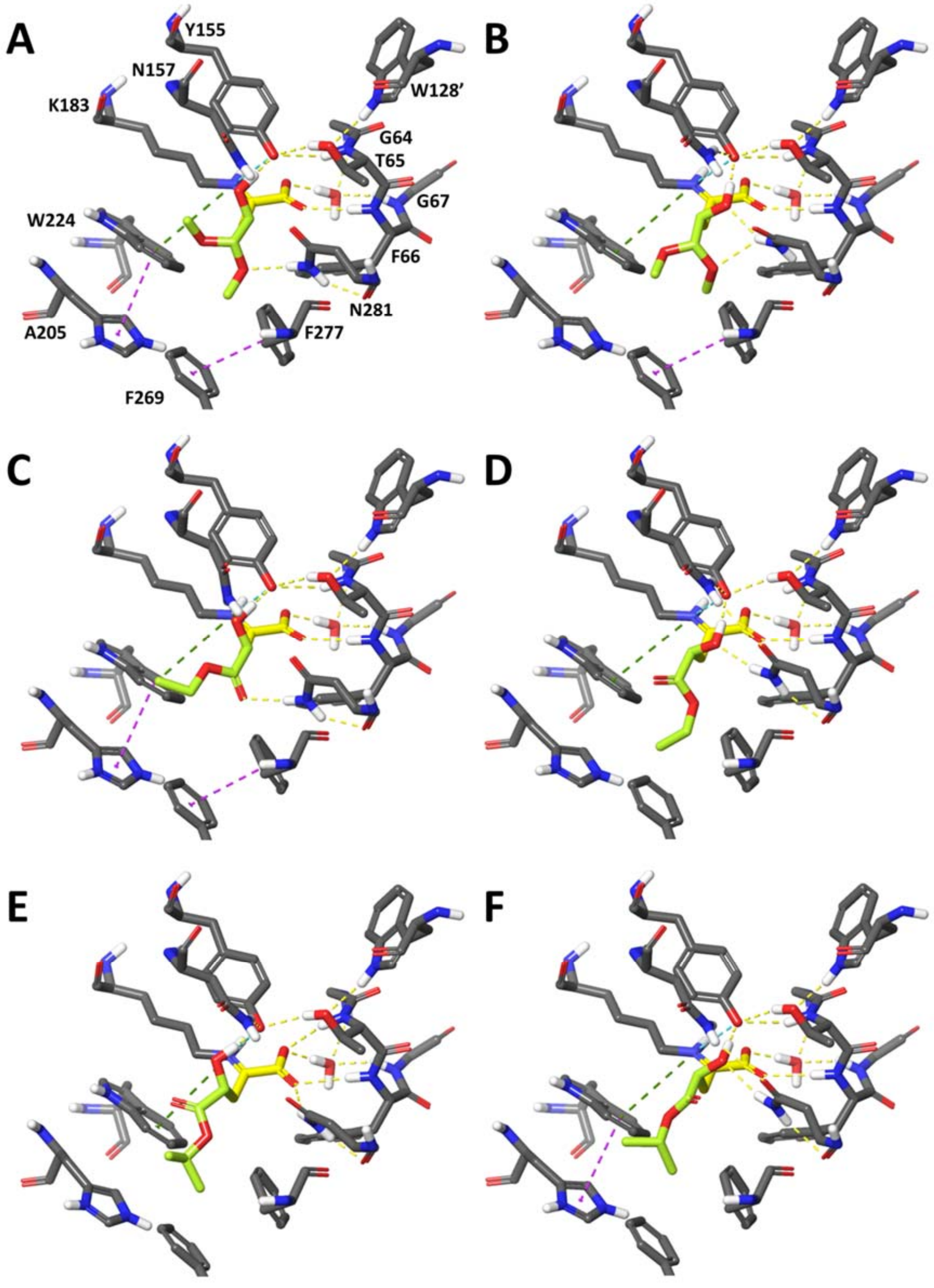

Figure S96. Models of HBPA complexes with the K183-bound aldol adduct imines derived from (**A**) (*R*)-**3g**, (**B**) (*S*)-**3g**, (**C**) (*R*)-**3h**, (**D**) (*S*)-**3h**, (**E**) (*R*)-**3i** and (**F**) (*S*)-**3i**. Representation details as in Figure S94.

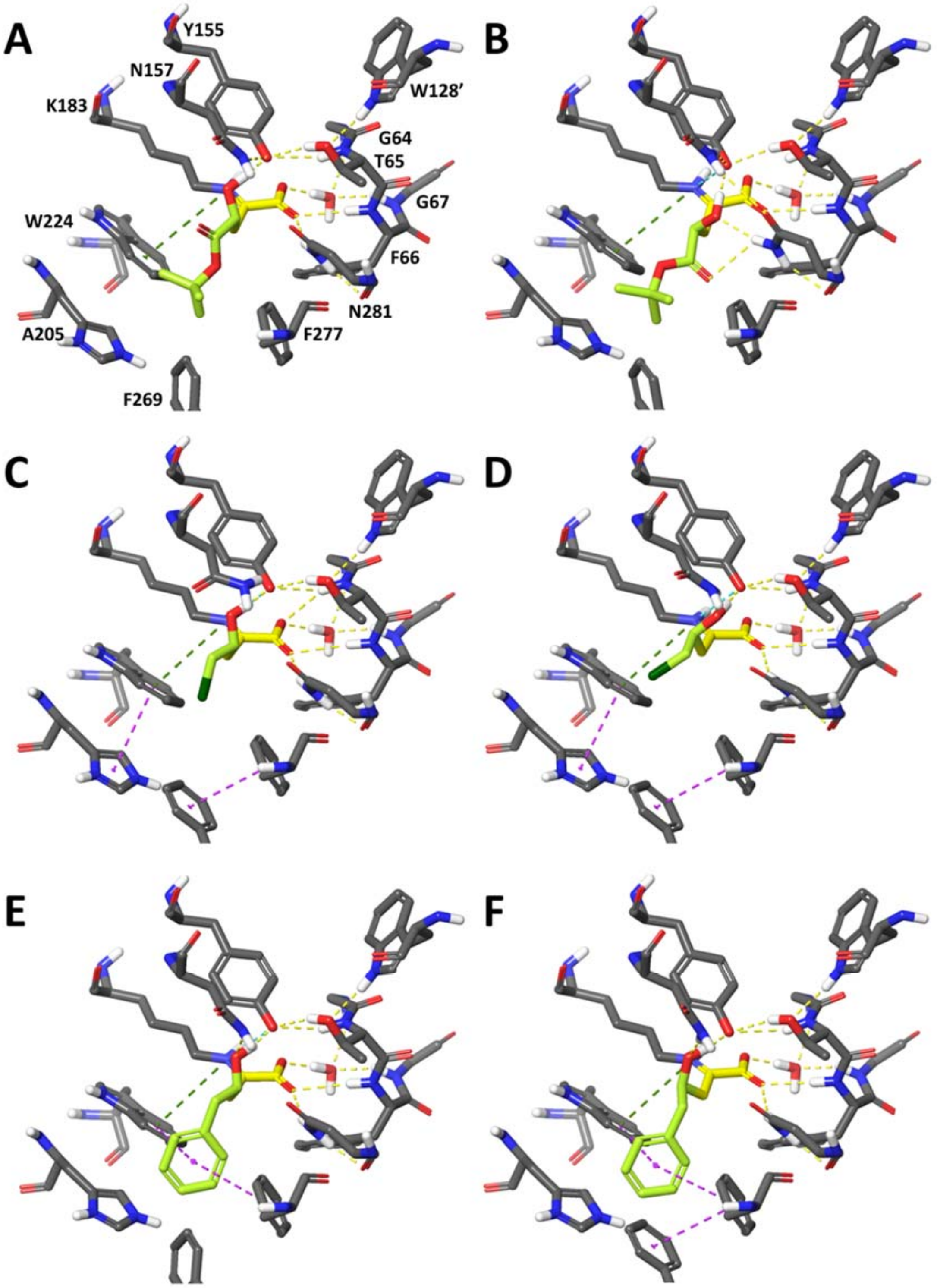

Figure S97. Models of HBPA complexes with the K183-bound aldol adduct imines derived from (**A**) (*R*)-**3j**, (**B**) (*S*)-**3j**, (**C**) (*R*)-**3k**, (**D**) (*S*)-**3k**, (**E**) (*S*)-**3l** and (**F**) (*R*)-**3l**. Representation details as in Figure S94.

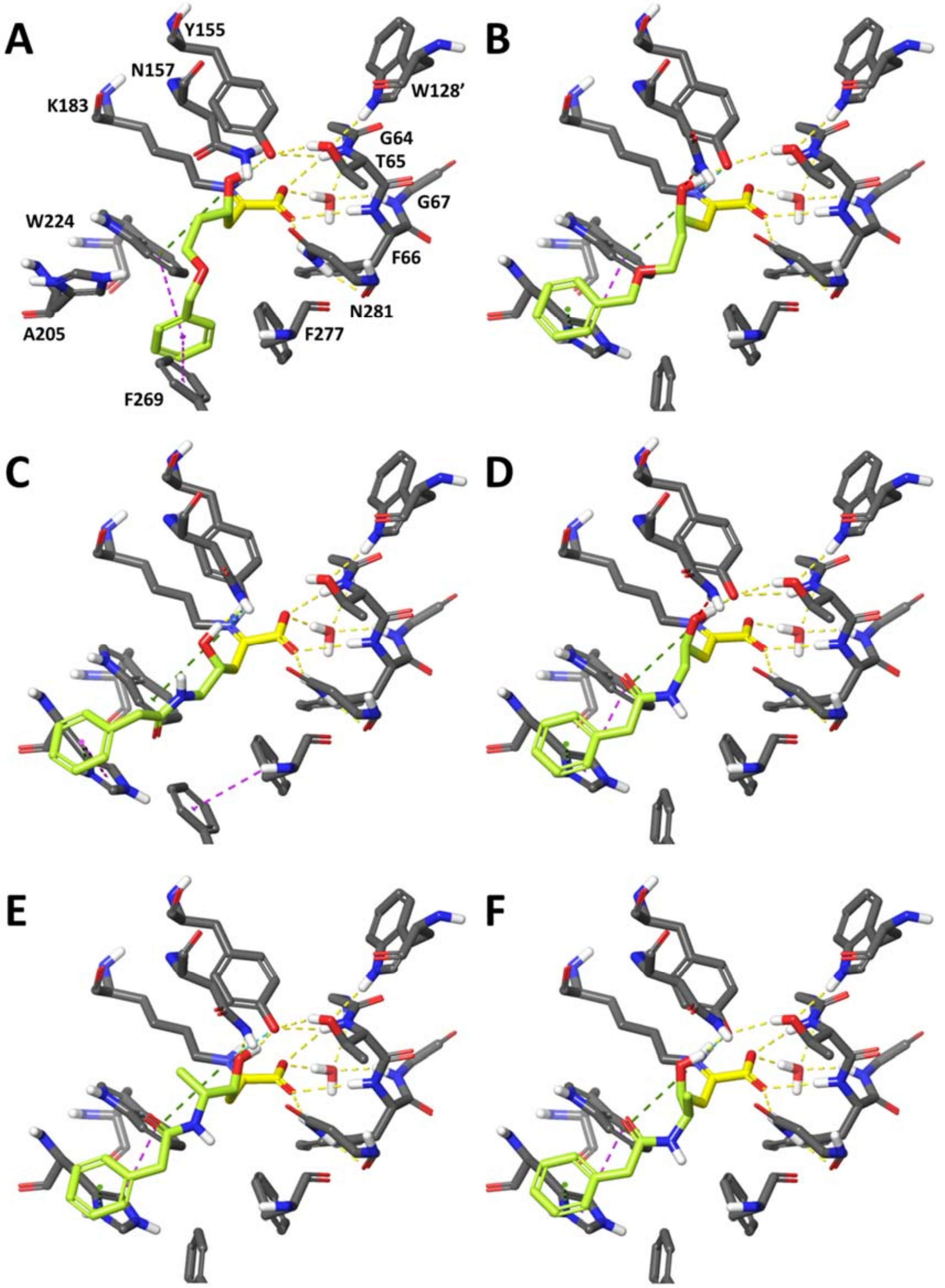

Figure S98. Models of HBPA complexes with the K183-bound aldol adduct imines derived from (**A**) (*R*)-**3m**, (**B**) (*S*)-**3m**, (**C**) (*R*)-**3n**, (**D**) (*S*)-**3n**, (**E**) (*R*)-**3o** and (**F**) (*S*)-**3o**. Representation details as in Figure S94.

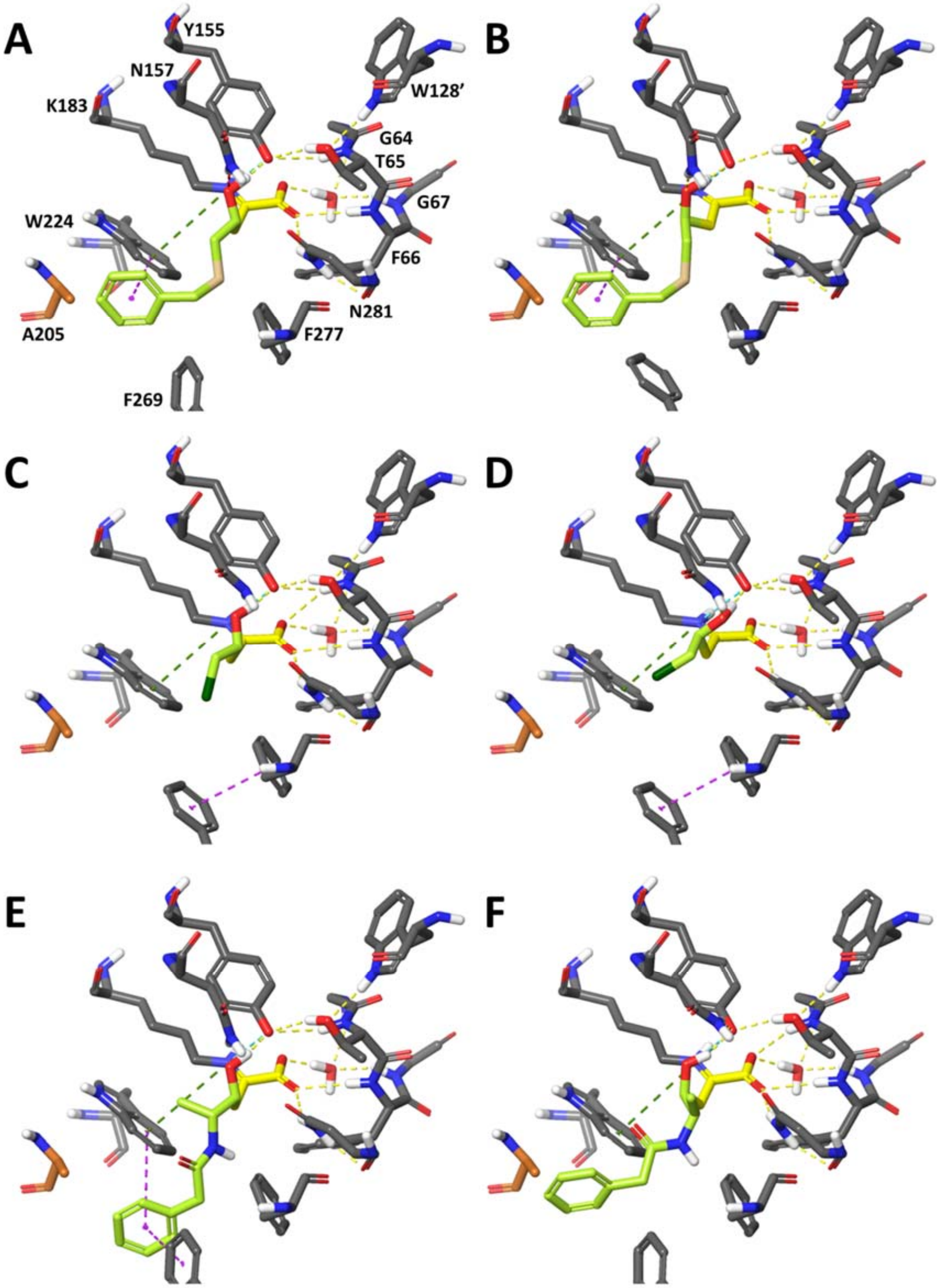

Figure S99. Models of HBPA H205A complexes with the K183-bound aldol adduct imines derived from (**A**) (*R*)-**3c**, (**B**) (*S*)-**3c**, (**C**) (*R*)-**3k**, (**D**) (*S*)-**3k**, (**E**) (*R*)-**3o** and (**F**) (*S*)-**3o**. The mutated residue A205 is highlighted in orange. The rest of representation details as in Figure S94.

Computational Methods.

Protein complexes were modeled with the package Schrödinger Suite 2020-2,[15] through its graphical interface Maestro.[16] The Protein Preparation Wizard[17] included in Maestro was used to prepare the protein structure by removing solvent molecules and ions, adding hydrogens, setting protonation states[18] and running a restrained minimization using the OPLS3 force-field.[19] The program MacroModel[17a] with the same force field and GB/SA water solvation conditions[20] was used for further molecular mechanics calculations. The program QSite[21] was used for the QM/MM calculations.

Molecular models of the pre-reactive pyruvate-enamine intermediate with the electrophiles bound into the active site of the HBPA protein were generated starting from the HBPA crystal structure (PDB 6DAO).[14] Mutations were introduced within Maestro, followed by restrained minimization. The structures of the bound pyruvate and electrophiles were built within Maestro, based on the structure of the ligand bound into the active site of the homolog CBPHA protein (PDB 6DAQ). The pyruvate was modeled as enamine bound to the essential K183 residue. The electrophiles were modeled approaching the enamine from their *re*- and *si*-faces. For that purpose, they were manually placed in the active site of HBPA and adequately oriented to satisfy the conditions of (i) having its C-atom at approximately 2.4 Å of the reactive enamine C-atom of pyruvate, and (ii) stablishing a hydrogen bond interaction between the aldehyde O-atom and the phenol and amide groups of Y155 and N157, respectively. The structures of the complexes were minimized with QSite at the DFT B3LYP/6-31G** level of theory, establishing a distance constraint between the two reactive C-atoms to keep them at a distance of 2.4 Å. The QM/MM boundary was defined by placement of hydrogen caps between the Cα and Cβ atoms of residues Y155, N157, K183 and N281 of HBPA. The pyruvate-enamine, a water molecule that is H-bound to the pyruvate carboxylate, and the electrophile molecules were also included as part of the QM region. All residues with atoms within 6 Å of the QM region were simultaneously optimized using the OPLS3 force-field,[19] while residues which were further away were kept frozen. A distance dependent dielectric ($\varepsilon = 78$) was used for calculating the electrostatic component of the MM energy. Furthermore, to find the best bound conformations for the more flexible electrophiles, a conformational search was performed using the mixed MCMM/LMCS method[22] implemented in MacroModel to find the best poses for the aldehyde R^1 substituents, while the carbonyl group and the rest of the system were kept frozen. Then,

the best conformers detected by this search were QM/MM reoptimized as above. Finally, the optimized structures were used to build the corresponding aldol adducts, bound as imines to residue Lys183. According to the mechanism, after C-C bond formation, the resulting intermediates should have the Lys183 ε-nitrogen atom protonated and the phenol group of Tyr155 unprotonated. These structural modifications were performed within Maestro and the resulting model structures were QM/MM reoptimized as above.

References.

1. (a) Calveras, J.; Egido-Gabás, M.; Gómez, L.; Casas, J.; Parella, T.; Joglar, J.; Bujons, J.; Clapés, P., Dihydroxyacetone phosphate aldolase-catalyzed synthesis of structurally diverse polyhydroxylated pyrrolidine derivatives and evaluation of their glycosidase inhibitory properties. *Chem. Eur. J.* **2009**, *15*, 7310-7328; (b) Concia, A. L.; Lozano, C.; Castillo, J. A.; Parella, T.; Joglar, J.; Clapés, P., D-Fructose-6-phosphate aldolase in organic synthesis: cascade chemical-enzymatic preparation of sugar-related polyhydroxylated compounds. *Chem. Eur. J.* **2009**, *15*, 3808-3816; (c) Garrabou, X.; Gomez, L.; Joglar, J.; Gil, S.; Parella, T.; Bujons, J.; Clapés, P., Structure-guided minimalist redesign of L-fuculose-1-phosphate aldolase active site. Expedient synthesis of novel polyhydroxylated pyrrolizidines and their inhibitory properties against glycosidases and intestinal disaccharidases *Chem. Eur. J.* **2010**, *16*, 10691-10706.

2. Hernández, K.; Parella, T.; Joglar, J.; Bujons, J.; Pohl, M.; Clapés, P., Expedient Synthesis of C-Aryl Carbohydrates by Consecutive Biocatalytic Benzoin and Aldol Reactions. *Chem. Eur. J.* **2015**, *21*, 3335-3346.

3. Hernández, K.; Gómez, A.; Joglar, J.; Bujons, J.; Parella, T.; Clapés, P., 2-Keto-3-Deoxy-L-Rhamnonate Aldolase (YfaU) as Catalyst in Aldol Additions of Pyruvate to Amino Aldehyde Derivatives. *Adv. Synth. Catal.* **2017**, *359*, 2090-2100.

4. Ferreira, T.; Rasb, W., ImageJ user guide: IJ 1.46 r. **2012**.

5. Eaton, R. W., trans-o-Hydroxybenzylidenepyruvate Hydratase-Aldolase as a Biocatalyst. *Appl. Environ. Microbiol.* **2000**, *66*, 2668-2672.

6. Mironov, G. G.; St-Jacques, A. D.; Mungham, A.; Eason, M. G.; Chica, R. A.; Berezovski, M. V., Bioanalysis for Biocatalysis: Multiplexed Capillary Electrophoresis–Mass Spectrometry Assay for Aminotransferase Substrate Discovery and Specificity Profiling. *J. Am. Chem. Soc.* **2013**, *135*, 13728-13736.

7. Keiko, N. A.; Stepanova, L. G.; Verochkina, E. A.; Chuvashev, Y. A., Synthesis and properties of alkylthioethanals. *ARKIVOC (Gainesville, FL, U. S.)* **2011**, 127-138.

8. Hon, Y.-S.; Wong, Y.-C.; Chang, C.-P.; Hsieh, C.-H., Tishchenko reactions of aldehydes promoted by diisobutylaluminum hydride and its application to the macrocyclic lactone formation. *Tetrahedron* **2007**, *63*, 11325-11340.

9. Kumar, S.; Pearson, A. L.; Pratt, R. F., Design, synthesis, and evaluation of α-ketoheterocycles as class C β-lactamase inhibitors. *Bioorg. Med. Chem.* **2001**, *9*, 2035-2044.

10. Zhang, P.-l.; Wang, G.; Liu, J.-s.; Xu, F.-q.; Zhao, Z.-z.; Wang, W.-x.; Wang, J.-t.; Wang, G.-k.; Wu, P.-y., Three new metabolites from the endophytic fungus

Climacocystis montana isolated from the root bark of Paeonia ostia. *Phytochemistry Letters* **2018**, *26*, 50-54.

11. Espelt, L.; Parella, T.; Bujons, J.; Solans, C.; Joglar, J.; Delgado, A.; Clapés, P., Stereoselective aldol additions catalyzed by dihydroxyacetone phosphate dependent aldolases in emulsion systems: preparation and structural characterization of linear and cyclic aminopolyols from aminoaldehydes. *Chem. Eur. J.* **2003**, *9*, 4887-4899.

12. White, J. D.; Hansen, J. D., Total Synthesis of (−)-7-Epicylindrospermopsin, a Toxic Metabolite of the Freshwater Cyanobacterium Aphanizomenon ovalisporum, and Assignment of Its Absolute Configuration. *J. Org. Chem.* **2005**, *70*, 1963-1977.

13. Sheldrick, G., A short history of SHELX. *Acta Crystallogr. Sect. A* **2008**, *64*, 112-122.

14. LeVieux, J. A.; Medellin, B.; Johnson, W. H.; Erwin, K.; Li, W.; Johnson, I. A.; Zhang, Y. J.; Whitman, C. P., Structural Characterization of the Hydratase-Aldolases, NahE and PhdJ: Implications for the Specificity, Catalysis, and N-Acetylneuraminate Lyase Subgroup of the Aldolase Superfamily. *Biochemistry* **2018**, *57*, 3524-3536.

15. Schrödinger *Release 2020-2: Schrödinger Suite 2020-2 Protein Preparation Wizard; Epik, Schrödinger, LLC, New York, NY, 2020; Impact, Schrödinger, LLC, New York, NY, 2020; Prime, Schrödinger, LLC, New York, NY, 2020.*, 2020.

16. Schrödinger *Release 2020-2: Maestro*, Schrödinger, LLC: New York, NY, 2020.

17. (a) Schrödinger *Release 2020-2: Macromodel*, Schrödinger, LLC: New York, NY, 2020; (b) Sastry, G. M.; Adzhigirey, M.; Day, T.; Annabhimoju, R.; Sherman, W., Protein and ligand preparation: parameters, protocols, and influence on virtual screening enrichments. *J. Comput. Aided Mol. Des.* **2013**, *27*, 221-234.

18. Olsson, M. H. M.; Søndergard, C. R.; Rostkowski, M.; Jensen, J. H., PROPKA3: Consistent Treatent of Internal and Surface Residues in Empirical pKa predictions. *J. Chem. Theor. Comput.* **2011**, *7*, 525-537.

19. Harder, E.; Damm, W.; Maple, J.; Wu, C.; Reboul, M.; Xiang, J. Y.; Wang, L.; Lupyan, D.; Dahlgren, M. K.; Knight, J. L.; Kaus, J. W.; Cerutti, D. S.; Krilov, G.; Jorgensen, W. L.; Abel, R.; Friesner, R. A., OPLS3: A Force Field Providing Broad Coverage of Drug-like Small Molecules and Proteins. *J Chem Theory Comput* **2016**, *12*, 281-296.

20. Still, W. C.; Tempczyk, A.; Hawley, R. C.; Hendrickson, T., Semianalytical Treatment of Solvation for Molecular Mechanics and Dynamics. *J. Am. Chem. Soc.* **1990**, *112*, 6127-6129.

21. (a) Schrödinger *Release 2020-2: QSite*, Schrödinger, LLC: New York, NY, 2020; (b) Murphy, R. B.; Philipp, D. M.; Friesner, R. A., A mixed quantum mechanics/molecular mechanics (QM/MM) method for large-scale modeling of chemistry in protein environments. *J. Comp. Chem.* **2000**, *21*, 1442-1457; (c) Philipp, D. M.; Friesner, R. A., Mixed ab initio QM/MM modeling using frozen orbitals and tests with alanine dipeptide and tetrapeptide. *J. Comp. Chem.* **1999**, *20*, 1468-1494.

22. Kolossvary, I.; Guida, W. C., Low-mode conformational search elucidated: application to $C_{39}H_{80}$ and flexible docking of 9-deazaguanine inhibitors into PNP. *J. Comput. Chem.* **1999**, *20*, 1671-1684.
